# Combination of Pseudo‐Natural Product Design and Formal Natural Product Ring Distortion Yields Stereochemically and Biologically Diverse Pseudo‐Sesquiterpenoid Alkaloids

**DOI:** 10.1002/anie.202106654

**Published:** 2021-08-19

**Authors:** Jie Liu, Jana Flegel, Felix Otte, Axel Pahl, Sonja Sievers, Carsten Strohmann, Herbert Waldmann

**Affiliations:** ^1^ Max Planck Institute of Molecular Physiology Department of Chemical Biology Otto-Hahn-Strasse 11 44227 Dortmund Germany; ^2^ Technical University Dortmund Faculty of Chemistry Chemical Biology Otto-Hahn-Strasse 6 44221 Dortmund Germany; ^3^ Technical University Dortmund Faculty of Chemistry Inorganic Chemistry Otto-Hahn-Strasse 6 44221 Dortmund Germany; ^4^ Compound Management and Screening Center Dortmund Germany

**Keywords:** cycloaddition, pseudo-natural products, ring distortion, stereodivergent synthesis

## Abstract

We describe the synthesis and biological evaluation of a new natural product‐inspired compound class obtained by combining the conceptually complementary pseudo‐natural product (pseudo‐NP) design strategy and a formal adaptation of the complexity‐to‐diversity ring distortion approach. Fragment‐sized α‐methylene‐sesquiterpene lactones, whose scaffolds can formally be viewed as related to each other or are obtained by ring distortion, were combined with alkaloid‐derived pyrrolidine fragments by means of highly selective stereocomplementary 1,3‐dipolar cycloaddition reactions. The resulting pseudo‐sesquiterpenoid alkaloids were found to be both chemically and biologically diverse, and their biological performance distinctly depends on both the structure of the sesquiterpene lactone‐derived scaffolds and the stereochemistry of the pyrrolidine fragment. Biological investigation of the compound collection led to the discovery of a novel chemotype inhibiting Hedgehog‐dependent osteoblast differentiation

## Graphical abstract

The combination of the conceptually complementary formal ring distortion‐ and pseudo‐natural product strategies yields novel natural product‐inspired bioactive pseudo‐sesquiterpenoid alkaloids.

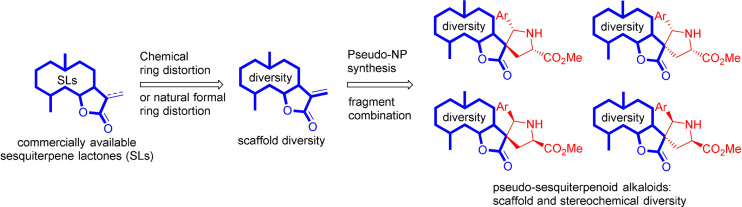

## Introduction

**Figure 1:**
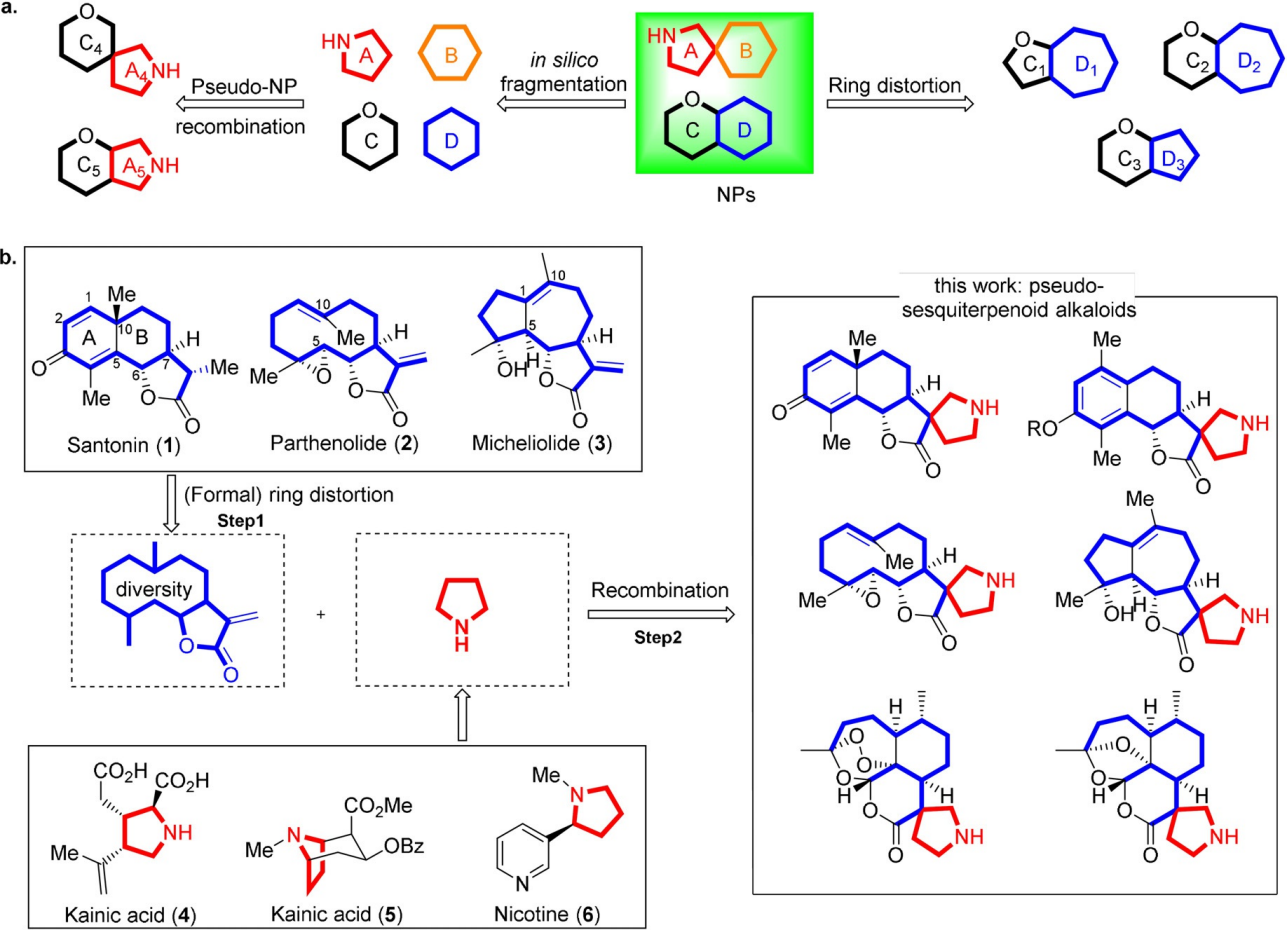
Combination of the (formal) ring distortion strategy with the pseudo‐NP principle for the synthesis of novel chemically diverse NP‐inspired compound classes with diverse biological performance. a) Previously reported synthetic strategies toward NP‐inspired compound collections. b) (Formal) ring distortion of sesquiterpene lactones (SLs) led to diverse scaffolds which were recombined with pyrrolidine fragments.

The design and synthesis of structurally complex and chemically diverse compound collections, encoding diverse biological activities are at the heart of chemical biology and drug discovery, and novel approaches are in high demand.^[1]^ Natural product (NP) structures harness biological relevance established in evolution and have served as inspiration for the discovery of novel bioactive compound classes^[2]^ for instance by late‐stage functionalization of NPs,^[3]^ function‐oriented synthesis (FOS)^[4]^ and biology‐oriented synthesis (BIOS).^[5]^ However, these strategies yield compound collections which explore chemical and biological space closely related to the parent NPs. For wider exploration of NP‐inspired chemical and biological space, ring distortion by means of the complexity‐to‐diversity approach[^6^, ^7^] and the development of pseudo‐natural products (pseudo‐NPs)[^8^, ^9^] have recently been introduced (Figure 1 a).

Ring distortion uses available natural products as starting points which are then subjected to different transformations resulting in chemical modulation of the underlying NP scaffolds to yield compound classes with different ring structure and bioactivity.^[7]^ In the design and synthesis of pseudo‐NPs, natural products are viewed as combinations of fragments into which they can be disassembled by means of cheminformatic methods,^[10]^ and they can themselves be fragment‐sized. Chemical recombination of NP‐fragments from different sources in unprecedented arrangements by means of complexity‐generating transformations will generate novel NP‐inspired compound classes with unexpected or new bioactivities.

We envisioned that combination of these two strategies would yield novel natural product‐inspired compound classes with diverse scaffolds and a priori endowed with bioactivity. On the one hand fragment‐sized NPs would be subjected to ring‐distortion transformations, which requires that scaffolds of natural products themselves need to be distorted by chemical transformations. This strategy could be complemented by an approach which can be regarded as formal adaptation of the ring distortion principle. In this alternative approach NPs are directly employed whose scaffolds can formally be regarded as formed by means of ring distortion reaction for instance in biosynthesis cascades (i.e. the ring distortion reaction would already have occurred in nature). Subsequent chemical combination with structurally unrelated NP‐fragments through complexity‐generating transformations would afford compound collections with pronounced scaffold‐ and stereochemical complexity as outlined in Figure 1.

Here we report a first exploration of this approach. We employed several fragment‐sized^[11]^ α‐methylene‐sesquiterpene lactones (SLs) whose different scaffolds can formally be viewed as related to each other by ring distortion (i.e., we took advantage of the natural diversity in this terpene class), or subjected them to ring distortion reactions. Stereocomplementary asymmetric 1,3‐dipolar cycloadditions of dehydrolactones obtained from the natural products with amino acid‐derived azomethine ylides resulted in the formation of stereoisomeric *spiro*‐pyrrolidine fragments, characteristic for pyrrolidine alkaloids (Figure 1). Cheminformatic analysis of the resulting pseudo‐sesquiterpenoid alkaloids and biological characterization in a high content morphological assay monitoring a wide range of bioactivity and in several assays monitoring different signaling pathways revealed that the novel NP‐inspired pseudo‐sesquiterpenoid alkaloids are both chemically and biologically performance diverse and define a novel chemotype inhibiting Hedgehog‐dependent osteoblast differentiation.

## Results and Discussion

### Development of Stereodivergent 1,3‐Dipolar Cycloadditions with Sesquiterpene Lactones

In order to explore the combination of ring distortion or the adapted approach as described above with pseudo‐NP design, we planned to combine different sesquiterpene lactone scaffolds with pyrrolidine fragments. Sesquiterpene lactones (SLs) are a diverse class of biologically active natural products with multiple bioactivities^[12]^ and many are fragment‐sized.^[11]^ Their scaffolds can be considered structurally related, and in a formal sense it is imaginable that these fragments could be converted into each other by means of ring‐distortion. For instance, santonin **1** contains a cyclohexadienone ring, whereas in structurally related parthenolide **2** C5 and C10 are not connected, and transannulation of parthenolide yields tricyclic micheliolide **3**, which can also be seen as a rearranged scaffold of santonin **1** (Figure 1 b).

Sesquiterpene lactones frequently embody or can be equipped with a reactive α‐methylene‐γ‐lactone which is a major determinant of bioactivity due to covalent reaction with biological nucleophiles.^[13]^ We reasoned that conversion of this reactive functional group into a new NP fragment by means of an appropriate complexity‐generating reaction would enable both, exploration of novel chemical space and new bioactivity. As complexity‐generating reaction, the asymmetric 1,3‐dipolar cycloaddition with azomethine ylides was chosen. This cycloaddition is a very reliable and powerful method for the incorporation of the pyrrolidine moiety,[^14^, ^15^] which occurs in numerous alkaloids, like kainic acid,^[16]^ cocaine^[17]^ and nicotine^[18]^ (Figure 1 b). For the asymmetric steering of these cycloadditions several efficient catalyst systems are available, but it has rarely been applied for the functionalization of natural products.^[19]^

Since stereogenic character and content parallel bioactivity^[20]^ it was planned to synthesize a stereochemically diverse collection, that is, to generate all possible stereoisomers of the newly formed pyrrolidine ring in a stereocomplementary synthesis approach. This task was considered particularly challenging because the complex chiral environment and diverse functional groups of NPs may complicate catalyst‐ and substrate‐mediated control of stereoselectivity. Thus, while stereoselective synthesis of one isomer frequently is achievable,^[21]^ stereodivergent synthesis of all isomers is much more demanding because of match‐mismatch effects^[22]^ and limited availability of catalysts in stereoisomeric forms.^[23]^ For the development of suitable reaction conditions, santonin **1** was selected and converted into the corresponding α‐methylene‐γ‐lactone **7** (see Supplementary Figure S2 and Table 1).^[19c]^ Use of AgOAc in the absence of any ligand as catalyst for the dipolar cycloadditions with the azomethine ylide generated from Schiff base **8 a** by deprotonation predominantly yielded *endo*‐isomers **9 a** and **10 a** (Table 1, entry 1). Triphenyl phosphine (PPh_3_) as ligand induced a small decrease in *endo* selectivity (Table 1, entry 2), and (*R*)‐Fesulphos **L2** ^[24]^ yielded moderate *endo* selectivity (Table 1, entry 5). We previously reported that hydrogen bonding between the ligand and dipolarophile can improve *endo* selectivity.^[25]^ Accordingly, use of amine‐substituted phosphine **L3** was explored and the corresponding catalyst indeed catalyzed the dipolar cycloaddition with good *endo/exo*‐ and excellent facial selectivity (Table 1, entry 6). When THF was used as the solvent, ligand **L3** (condition A) and its enantiomer (condition B) gave excellent *endo‐Si* selectivity (Table 1, entry 7; condition A, and entry 8; condition B). Thus, under these conditions the *endo* selectivity is controlled by the chiral catalyst and not the chirality of the dipolarophile.

**Table 1:** Development of the stereodivergent synthesis of santonin‐pyrrolidines.^[a]^ 
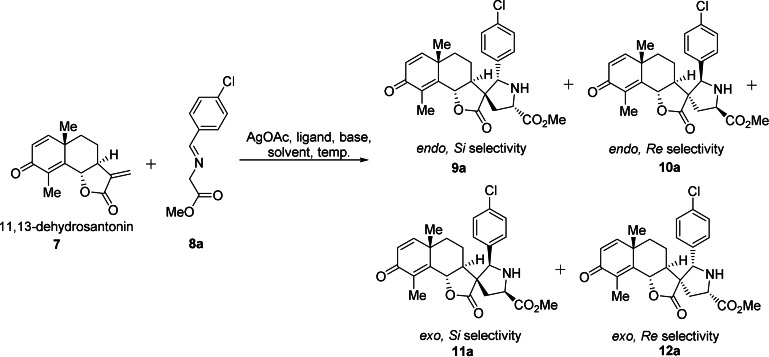

Entry	Ligand	Base	Solvent	*T*	Yield	d.r. (**9 a**:**10 a**:**11 a**:**12 a**)
1	–	Et_3_N	CH_2_Cl_2_	r.t.	74 %	72:23:04:01
2	PPh_3_	Et_3_N	CH_2_Cl_2_	r.t.	79 %	68:18:11:03
3	**L1**	Et_3_N	CH_2_Cl_2_	r.t.	93 %	21:06:71:02
4	*ent‐* **L1**	Et_3_N	CH_2_Cl_2_	r.t.	78 %	22:14:19:45
5	**L2**	Et_3_N	CH_2_Cl_2_	r.t.	88 %	60:05:09:26
6	**L3**	Et_3_N	CH_2_Cl_2_	r.t.	91 %	89:00:11:00
7	**L3**	Et_3_N	THF	r.t.	82 % (73 %)^[b]^	91:02:07:00
8	*ent*‐**L3**	Et_3_N	THF	0 °C	79 % (95 %)^[b]^	03:95:01:01
9	**L4**	Et_3_N	DCE	r.t.	92 % (89 %)^[b]^	00:00:100:00
10	*ent*‐**L4**	Et_3_N	CH_2_Cl_2_	r.t.	68 %	28:01:03:68
11^[c]^	**L5**	Cs_2_CO_3_	THF	r.t.	53 %	88:00:00:12
12^[c]^	**L5**	Cs_2_CO_3_	CHCl_3_	r.t.	77 %	13:00:02:86
13^[d]^	**L5**	Cs_2_CO_3_	CHCl_3_	0 °C	72 % (88 %)^[b]^	04:00:00:96
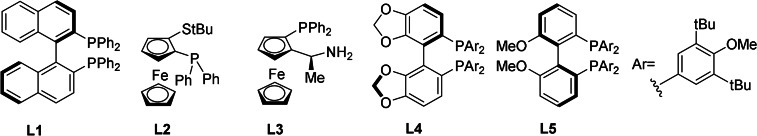						

[a] Dehydrosantonin **7** (0.10 mmol, 1.0 equiv), Schiff base **8 a** (0.15 mmol, 1.5 equiv) and base (0.02 mmol, 0.20 equiv) were reacted in solvent (0.10 M, 1 mL) with 5 mol % AgOAc and 6 mol % ligand for 24–48 hours. Yields for all isomers and d.r. (**9 a**:**10 a**:**11 a**:**12 a**) were determined from the reaction mixture by means of ^1^H NMR analysis with CH_2_Br_2_ as the internal standard. [b] The main isomer was purified by column chromatography; isolated yields of main isomers are shown in brackets. [c] 0.50 equiv Cs_2_CO_3_ was used. [d] 2.0 equiv Schiff base **8 a** was used.

However, in the presence of bulky phosphine ligand BINAP **L1** the *endo/exo* selectivity was reversed, and the (*R*)‐configured phosphine favored *Si* facial attack (**11 a**:**12 a**=71:2) (Table 1, entry 3). In comparison, the enantiomeric (*S*)‐BINAP (*ent*‐**L1**) induced only low *Re* facial selectivity (**11 a**:**12 a**=19:45) which suggests that the (*S*)‐configured phosphine ligand forms a mismatched pair with the substrate (Table 1, entry 4).

Based on these findings, bulky phosphine ligands were used for *exo* selective formation of the cycloadducts. Thus, in the presence of (*R*)‐DTBM Segphos **L4** as ligand in DCE as solvent, the reaction proceeded with almost quantitative *exo‐Si* selectivity (Table 1, entry 9; condition C). However, the enantiomeric ligand did not lead to satisfying *exo‐Re* facial selectivity (Table 1, entry 10). Exploration of several ligands revealed that (*S*)‐DTBM Biphep **L5** induced the highest diastereoselectivity (Table 1, entries 11–13; Supplementary Table S1). In these transformations choice of the right solvent is crucial to obtain high selectivity. In THF the *endo‐Re* attack is favored (Table 1, entry 11), however, the selectivity is switched to *exo‐Si* when chloroform is employed as the solvent (Table 1, entry 12). The solvent effects on the stereochemical course of 1,3‐dipolar cycloadditions using azomethine ylides were also observed in our recent report about dynamic catalytic highly enantioselective 1,3‐dipolar cycloaddition wherein thermodynamic and kinetic reaction control enabled the efficient synthesis of stereochemically diverse compound libraries using a unified catalyst system.^[26]^ Finally, lowering the temperature and increasing the amount of the Schiff base did not substantially influence yield and selectivity (Table 1, entry 13; condition D).

The chirality of the substrate has only a minor influence on the *endo* selectivity, such that with both enantiomers of **L3** *endo*‐cycloadducts are formed with high selectivity (Table 1, entries 7 and 8). However, in the *exo*‐selective transformations, the stereogenic character of the substrate introduces facial bias, probably due to the quaternary center embedded in the santonin scaffold (Supplementary Figure S1). When the dipole approaches the dipolarophile from the *Si* face, the methyl group attached to the quaternary center points away from the phosphine ligands, such that substrate and chiral ligand form a matched pair leading to excellent selectivity. Attack of the azomethine ylide to the *Re*‐face of the dipolarophile, on the contrary, is mismatched due to unfavorable interaction of the methyl group with the ylide, resulting in low *endo*/*exo* selectivity. Gratifyingly, tuning the solvents and using powerful ligands realized such a transformation.

### Asymmetric Synthesis of Pseudo‐Sesquiterpenoid Alkaloids

**Figure 2:**
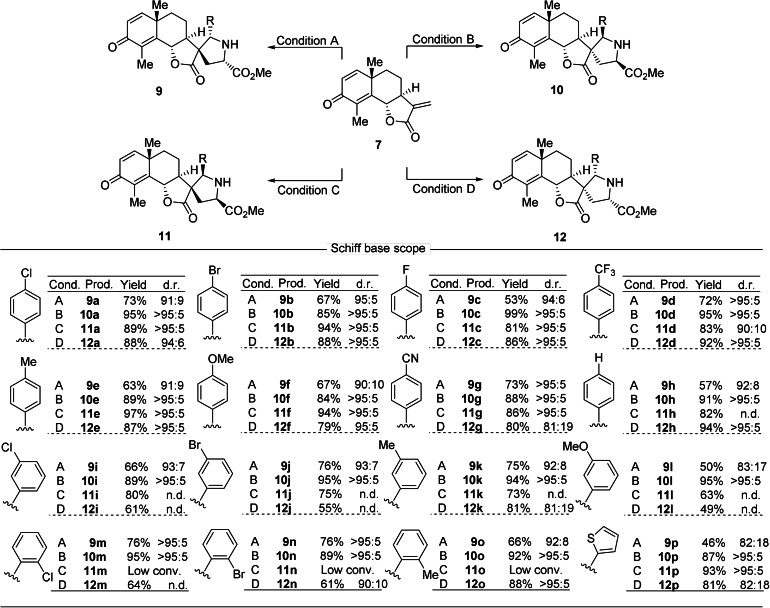
Substrate scope for the stereodivergent synthesis of santonin‐pyrrolidines. Condition A: 0.10 mmol dehydrosantonin and 0.15 mmol Schiff base, 0.02 mmol Et_3_N in 1 mL THF with 5 mol % AgOAc and 6 mol % **L3** at r.t. for 24–48 hours; Condition B: same as condition A but *ent*‐**L3** was used at 0 °C; Condition C: same as condition A except **L4** was used in 1 mL DCE; Condition D: 0.10 mmol dehydrosantonin and 0.20 mmol Schiff base, 0.05 mmol Cs_2_CO_3_ in 1 mL CHCl_3_ with 5 mol % AgOAc and 6 mol % **L5** at 0 °C for 24–48 hours. n.d.: not determined.

With the conditions for the stereodivergent synthesis of the four diastereomeric cycloadducts in hand, the scope of the stereoselective cycloaddition to dehydrosantonin **7** was explored (Figure 2). The transformation tolerates diverse substituents in the *para*‐ and *meta*‐ position of the phenyl group. In general, condition B delivers higher yield and diastereoselectivity compared to condition A. However, *ortho*‐substituted Schiff bases did not afford satisfying results (**11 m**–**11 o**) under condition C because of low conversion. Azomethine ylides derived from aliphatic aldehydes were not explored because they are not reactive in this transformation, presumably due to tautomerization of the imines. In total, 61 stereochemically diverse compounds were efficiently synthesized. The absolute configurations of **9 b**, **10 f** and **12 e** were unambiguously confirmed by X‐ray crystallographic analysis (see the Supporting Information, Figures S3–S5).

**Figure 3:**
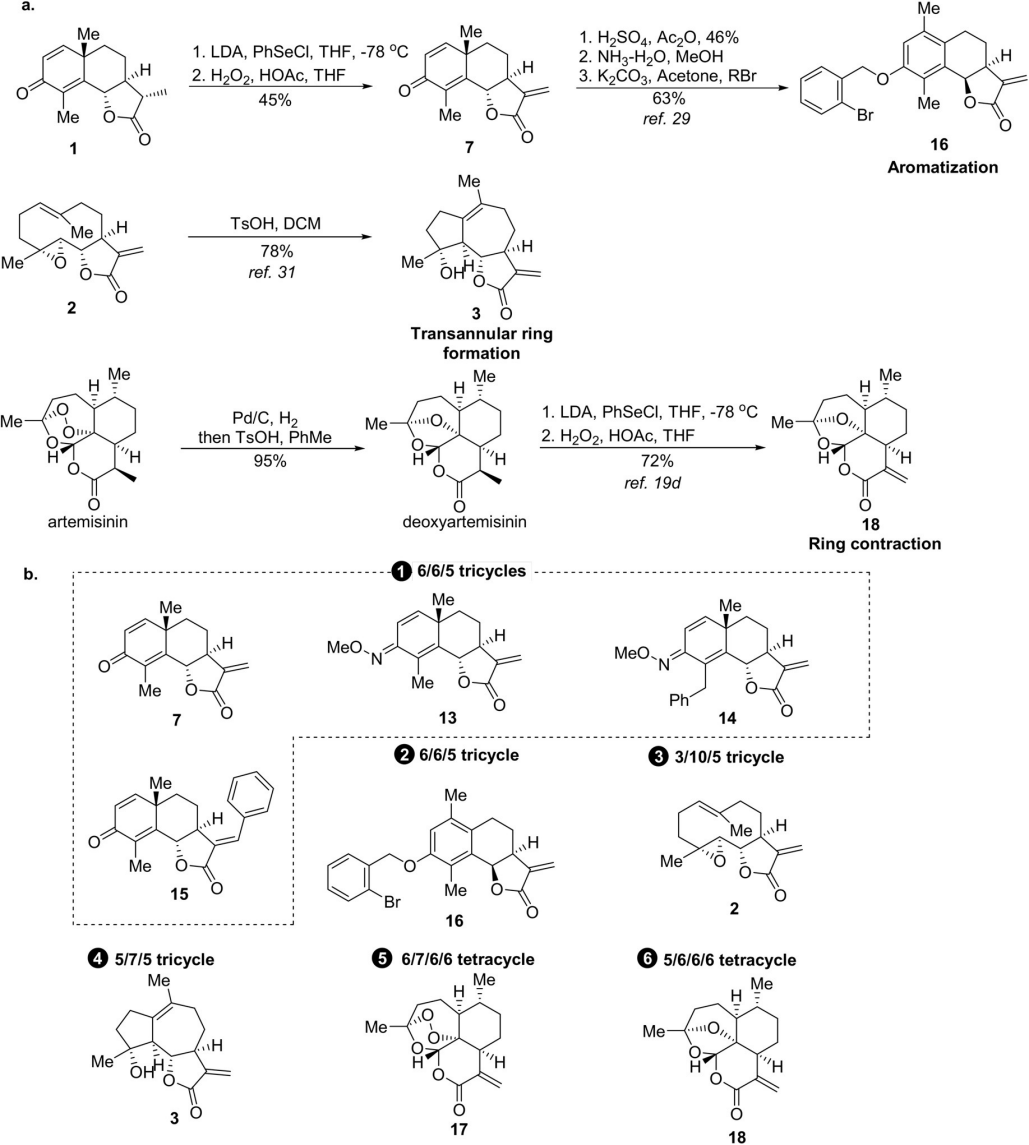
Sesquiterpene lactone‐derived compound classes employed in the synthesis of the pseudo‐sesquiterpenoid alkaloids. a) Ring distortion of commercially available sesquiterpene lactones. b) Structures of the nine lactones with six different scaffolds employed in the synthesis of the sesquiterpenoid alkaloid collection.

Encouraged by the wide scope of the asymmetric cycloaddition for the dipoles, the scope for different dipolarophiles was explored. To this end, santonin was subjected to different substitutions of the scaffold (Supplementary Figure S2), that is, oxime formation, C−H activation^[27]^ and Heck reaction^[28]^ to give analogs **13**, **14** and **15** respectively (Figure 3). Under acidic conditions, dehydrosantonin underwent a ring distortion reaction and rearranged to a phenol with stereoinversion of C6^[29]^ which was *O*‐alkylated to yield benzyl ether **16**. Aromatization of rings is considered a genuine ring distortion reaction as introduced by Hergenrother et al.[^6b^, ^30^]

In order to further explore the ring distortion concept, we included sesquiterpenoid lactones whose scaffolds can be formally viewed as related to santonin by ring distortion. Parthenolide **2** can be viewed as a ring‐opened analog of santonin. Transannular ring closure between the alkene and epoxide in parthenolide **2** results in formation of 5/7/5 tricyclic micheliolide **3** ^[31]^ (see Figure 3; Supplementary Figure S2). We also included artemisinin and its deoxy‐derivative and converted them to α‐methylene‐*δ*‐lactones **17** and **18** via a two‐step procedure, resulting in a ring contraction^[19d]^ (see Figure 3; Supplementary Figure S2).

Thus, for the synthesis of a collection of pseudo‐sesquiterpenoid alkaloids, three structurally related natural sesquiterpene lactones were subjected to different ring distortion reactions.

**Figure 4:**
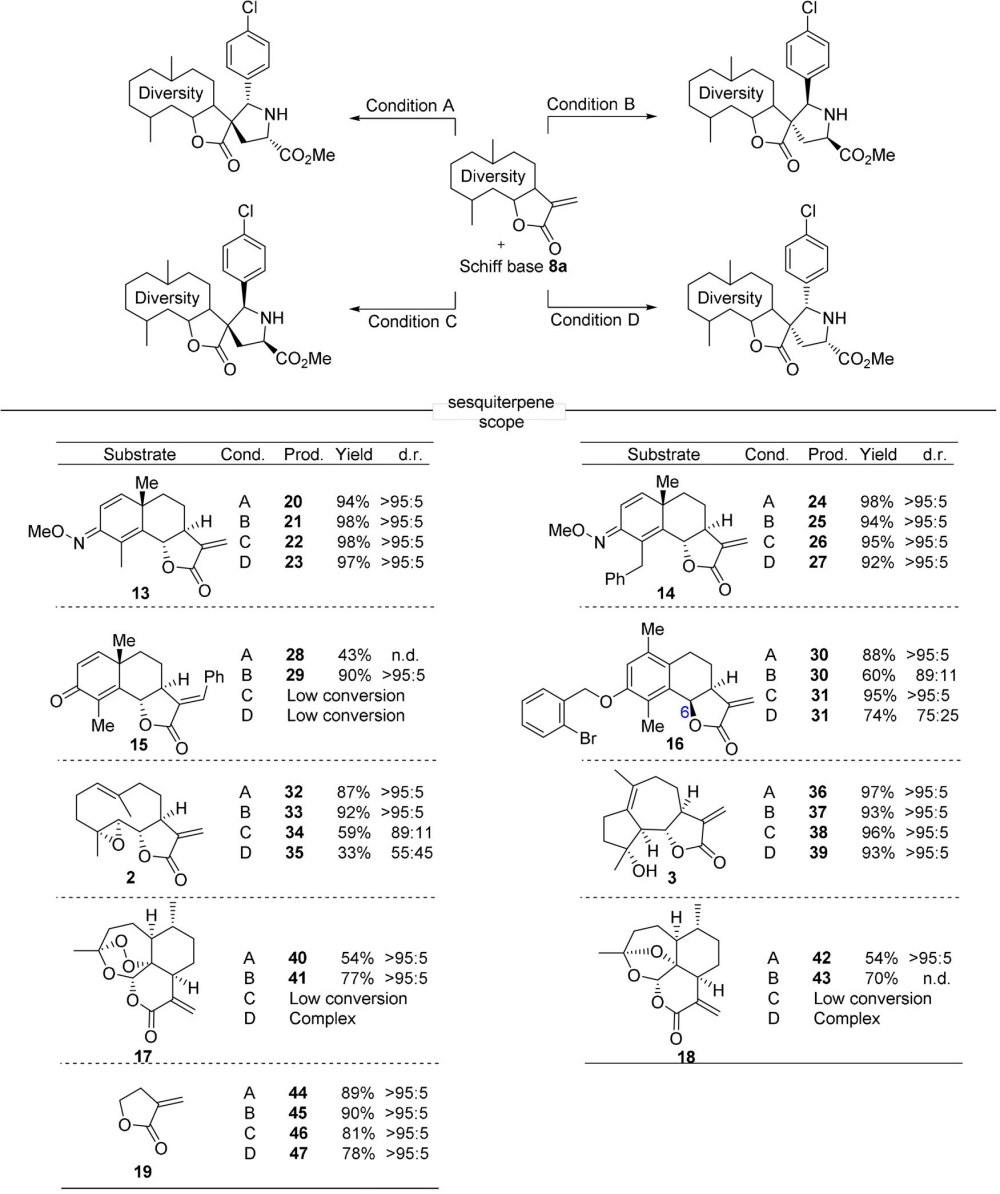
Substrate scope for diverse sesquiterpenes. All reactions were carried out under the conditions described above (Figure 2). 1,3‐dipolar cycloadditions with **15**–**18** were conducted on 0.05 mmol scale.

As shown in Figure 4 the dipolar cycloaddition also displays appreciable scope for different sesquiterpene lactones. Changes in ring A and B of dehydrosantonin **7** are well tolerated, and the oxime derivatives afforded the desired cycloadducts **20**–**23** and **24**–**27** in excellent yields and selectivity. Cycloadditions to the formally ring‐opened analog **2** and the rearranged scaffold **3** yielded very satisfying results (**32**–**35**; **36**–**39**). Even cycloaddition to the simplest lactone **19** afforded excellent stereoselectivity (**44**–**47**).^[32]^ However, the reaction was highly sensitive to modification of the γ‐lactone. Compound **15** with a phenyl ring attached to the methylene group showed low reactivity under the conditions for the *exo*‐selective reaction. Inversion of the stereocenter at C6 (**16**) confined the reaction to the *Si* face to yield cycloadducts **30** and **31** as the main isomers even under conditions B and D. The ring size of the lactone has a clear impact on the course of the reaction. Artemisinin derivatives **17** and **18** exclusively yielded *endo* products (**40**–**41**; **42**–**43**).

### Cheminformatic and Analysis of the Sesquiterpene Lactones and Pseudo‐Sesquiterpenoid Alkaloids

**Figure 5:**
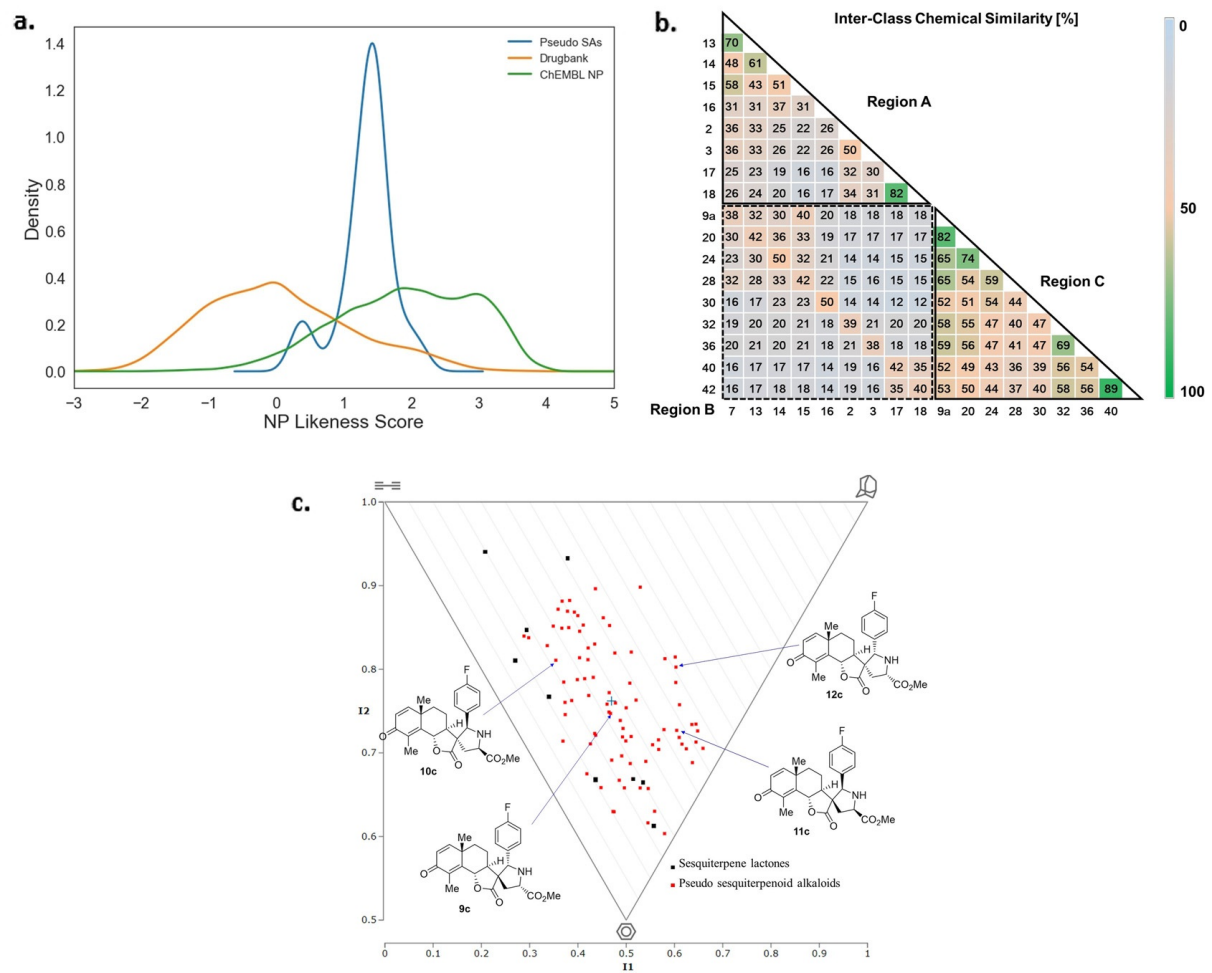
Characterization of the pseudo‐sesquiterpenoid alkaloids by means of cheminformatic. a) Comparison of the NP likeness scores calculated for the pseudo‐sesquiterpenoid alkaloids (Pseudo SAs) with the scores recorded for the NPs in the ChEMBL database and in Drugbank. b) Tanimoto cross chemical similarity comparison of the sublibraries. c) Analysis of the molecular shapes of the sesquiterpene lactones and the pseudo‐sesquiterpenoid alkaloids by means of a PMI plot.

For characterization of the 94 synthesized sesquiterpene lactones and pseudo‐sesquiterpenoid alkaloids the NP‐likeness score^[33]^ was computed using the open‐source software RDKit^[34]^ and compared to NPs in the ChEMBL database^[35]^ and compounds in the DrugBank,^[36]^ which characterize marketed and experimental drugs. The NP likeness score compares the frequency of occurrence of a given fragment in natural products with the frequency of occurrence in commercial compounds. Scores >0 indicate that the fragments are more common in natural products. Not unexpectedly, the synthesized sesquiterpene lactones directly obtained from natural products showed high scores. In comparison the scores recorded for the pseudo‐sesquiterpenoid alkaloids are lower, since their scaffolds result from fragment combinations and are novel (Supplementary Figure S6). However, the scores calculated for the pseudo‐sesquiterpenoid alkaloids are represented in the area characteristic for NPs (Figure 5 a). This result differs from the corresponding findings for pseudo natural product libraries, obtained by combination of different NP‐fragments which are more shifted to the area characteristic for the compounds in Drugbank.^[8a]^ This preliminary finding indicates that ring distortion of fragment‐sized NPs appears to retain a high degree of NP character in the combination with other NP‐fragments.

For analysis of compound class similarity, a representative group of the pseudo‐sesquiterpenoid alkaloids depicted in Figure 4 was analyzed by computing Tanimoto similarity of the Morgan fingerprints^[37]^ (for the structures of the compounds included in the analysis see Supplementary Figure S7). As shown in Figure 5 b, sesquiterpene lactones **2**, **3**, **7** and **13**–**18** display low cross similarity (Figure 5 b, region A), and cross‐similarity to the pseudo‐sesquiterpene alkaloids was low as well (Figure 5 b, region B). The intraclass chemical similarity of the pseudo‐sesquiterpenoid alkaloids **9 a** and **20**–**42** was higher (Figure 5 b, region C) probably because they share the same pyrrolidine fragment. Similarity was particularly high (89 %) for compounds **40** and **42** sharing almost the same scaffold.

NP likeness score and Tanimoto cross similarity do not distinguish stereoisomers. In order to analyze the incorporation of differently configured stereocenters in the compound collection, we calculated principal moments of inertia (PMI)^[38]^ (Figure 5 c). PMI plots are used to describe the molecular shape of lowest‐energy conformations and, therefore, they differentiate stereoisomers. The top‐left of the plot represents rod‐shaped compounds, the top‐right represents spheres and the bottom corner denotes disc‐shaped compounds. As shown in Figure 5 c, the pseudo‐sesquiterpenoid alkaloids (red dots) are broadly distributed in the PMI plot. On the contrary, most of the sesquiterpene lactones (black dots) resided along the rod‐disc side of the plot. Obviously, the recombination of the natural product scaffolds with the pyrrolidine moiety led to a shift to the sphere‐disc part of the PMI plot, representative for a higher degree in stereogenic character. Notably, even different stereoisomers occupy significantly different space in the plot, as exemplified for stereoisomers **9 c**–**12 c** (identified by blue arrows) in Figure 5 c. This analysis demonstrates that for the compound class analyzed here combination of ring distortion and pseudo NP synthesis yielded a stereochemically diverse compound collection that is structurally distinct from the guiding natural products which were subjected to ring distortion.

### Biological Performance Analysis of the Sesquiterpene Lactones and Pseudo‐Sesquiterpenoid Alkaloids

**Figure 6:**
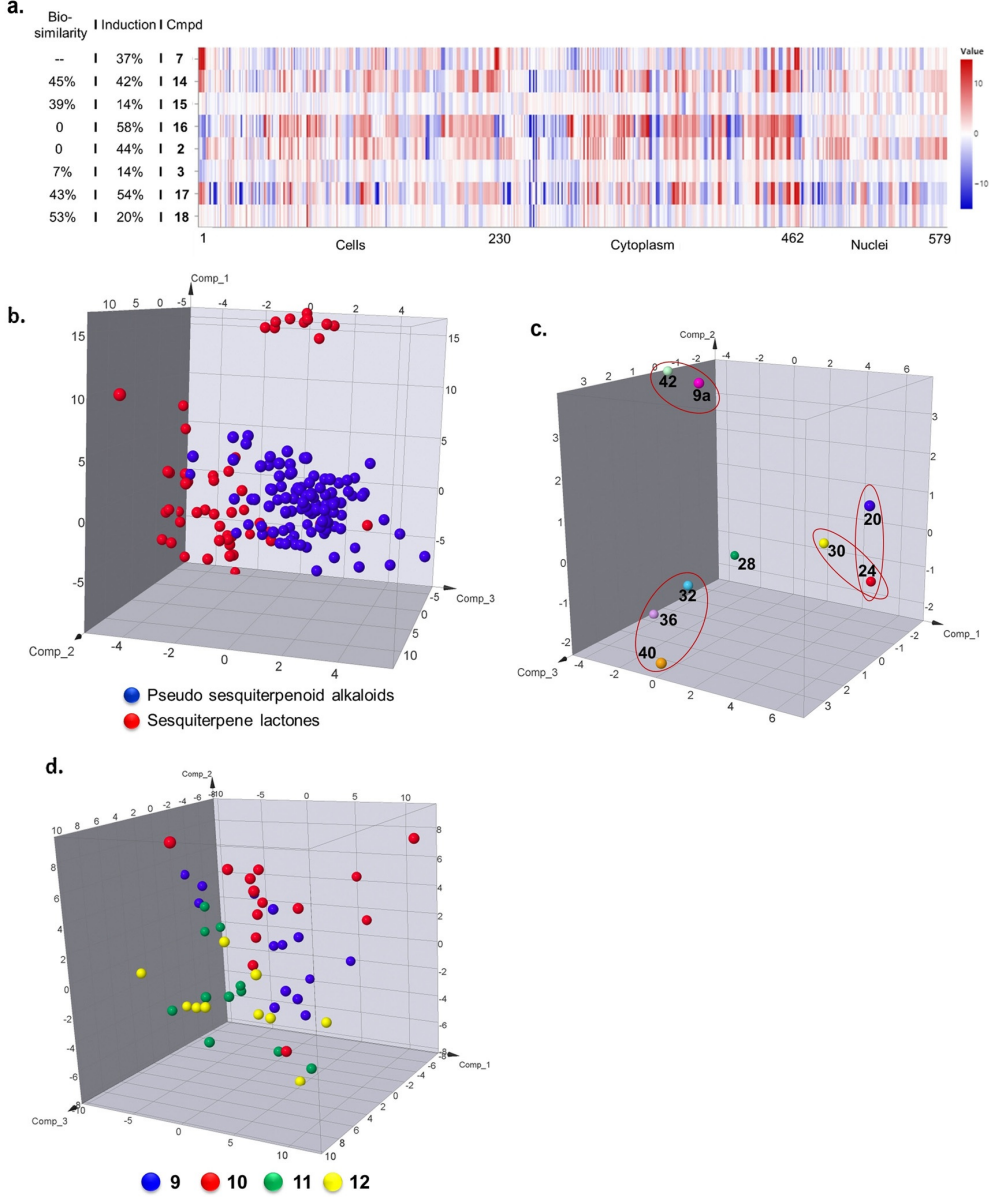
Characterization of the SLs and pseudo‐sesquiterpenoid alkaloids by means of high‐content morphological profiling in the cell painting assay. a) Cell painting assay fingerprint profiles for SL derivatives at 10 μM. Biosimilarity refers to the bioactivity similarity of the cell painting assay fingerprint profiles. The induction value is calculated based on the fraction of parameters (in %) that underwent significant changes (median absolute deviation [MAD] value) upon compound treatment of at least ± threefold of the median determined for the DMSO controls. b–d) Principal component analysis (PCA) of cell painting profiles. Unless otherwise noted, all analyses were based on active samples at all concentrations. b) Comparison between pseudo‐sesquiterpenoid alkaloids (PSA) and SLs. Expl. Var. PC1=40.9 %, PC2=13.3 %, PC3=7.9 %. c) Comparison of pseudo‐sesquiterpenoid alkaloids synthesized from diverse sesquiterpene scaffolds under 30 μM concentration. Compounds in red circles displayed >75 % cross biological similarity. Expl. Var. PC1=50.0 %, PC2=16.4 %, PC3=11.3 %. d) Comparison of stereoisomeric santonin‐pyrrolidines 9–12. Expl. Var. PC1=22.4 %, PC2=16.3 %, PC3=13.6 %.

Bioactivity of the sesquiterpene lactones **2**, **3**, **7** and **13**–**18** and the pseudo‐sesquiterpenoid alkaloids **9**–**12** and **20**–**47** was analyzed by means of the cell painting assay which monitors phenotypic changes in cells by employing six fluorescent dyes that selectively stain various compartments.^[39]^ Imaging via multi‐channel fluorescence microscopy identifies morphological changes condensed into 579 features to generate a characteristic profile. The number of significantly changed features in a profile relative to a DMSO control defines an induction value (see SI for more details). Biosimilarities of phenotypic profiles are calculated from the correlation distances between two profiles. Differences in morphological features can be observed among SL derivatives **2**, **3**, **7** and **14**–**18**, whose biosimilarities compared to dehydrosantonin **7** are below 60 % (Figure 6 a). We note that the α‐methylene‐γ‐lactone is considered a promiscuous covalent binder, potentially targeting multiple nucleophiles in proteins, and this reactivity is no longer present in the pseudo‐NPs. Thus, already in the design of the pseudo‐NP library described here, it could be expected that the biological performance of the synthesized pseudo‐NPs would be significantly different from the profiles for the guiding NPs indicating novel bioactivity (see also below).

Principal component analysis (PCA) was used to condense the phenotypic profiles of several compounds into three‐dimensional plots visualizing differences between compound classes by cluster formation. This approach has previously been used by Schreiber et al.^[40]^ and by us[^1b^, ^9b^] to demonstrate differences in cellular responses to stereo‐ and regioisomers.

Human osteosarcoma cells (U‐2 OS) were incubated with compounds at different concentrations (Supplementary Table S5) and analysis of the fingerprints revealed that the induction was concentration‐dependent (Supplementary Figure S8). Five compounds showed >5 % induction at 10 μM and at 50 μM 49 compounds induced significant morphological changes. To investigate the impact of fragment combination on bioactivity pattern, the sesquiterpene lactones (red dots) were compared with the corresponding pseudo‐sesquiterpenoid alkaloids (blue dots) by means of PCA (Figure 6 b) which revealed that the two compound classes collectively display distinctly different morphological profiles. This discovery is consistent with the notion that the α‐methylene lactone can serve as an electrophile to covalently bind biological nucleophiles. Combination with the pyrrolidine fragment abolishes this mode of action. Even in comparison with saturated sesquiterpene lactones, a relatively low cross biosimilarity was observed (Supplementary Figure 9 a,b).

The influence of the ring distortion was gleaned from the cross biosimilarity analysis for pseudo‐sesquiterpenoid alkaloids synthesized from diverse SLs (Supplementary Figure S9c). For compounds representing modifications based on santonin (compounds **20**, **24** and aromatized scaffold **30**), performance was relatively similar with cross biosimilarities >70 % (Figure 6 c; Supplementary Figure S9c). Notably **40** and **42** which shared the highest cross chemical similarity displayed distinctly different biological performance (Figure 6 c). However, **32**, **36** and **40** showed high cross biosimilarity (>75 %; red oval) even though they are structurally different (Figure 6 c; Supplementary Figure S9c). The corresponding sesquiterpene lactones **2**, **3** and **17** displayed highly different biological performance (Supplementary Figure S9d). After combination with the same pyrrolidine fragment, the resulting biological performance does not differentiate the influence of the SL scaffolds anymore. Pyrrolidine **44** itself did not induce any significant morphological changes even at 50 μM concentration (Supplementary Table S5). Therefore, the convergence of biological performance was not induced by a dominant effect of the pyrrolidine fragment.

In order to analyze the influence of different stereoisomers the pseudo‐sesquiterpenoid alkaloids derived from dehydrosantonin **1** were analyzed. The data revealed a separation between classes **9**–**12** (Figure 4 f; Supplementary Figure S10). The finding that cell painting can differentiate stereoisomers has been reported before.^[40]^ As mentioned above, compounds **32**, **36** and **40** displayed relatively high cross biosimilarity regardless of different sesquiterpene scaffolds. Very interestingly when different stereoisomers of these compounds (**33** vs. **41**; **35** vs. **39**) were compared, the influence of ring distortion was again visible leading to highly different biological performance (Supplementary Figure S9e,f).

### Identification of a Pseudo‐Sesquiterpenoid Alkaloid Inhibitor of Hedgehog‐Dependent Osteoblast Differentiation

In order to get more detailed insight into the bioactivity of the sesquiterpenoid alkaloids we subjected them to several cell‐based assays monitoring different cellular pathways and programs, including autophagy, T‐cell signaling as well as signal transduction through the Wnt and the Hedgehog (Hh) pathways. These investigations revealed a novel inhibitor of Hh‐dependent osteoblast differentiation.

**Figure 7:**
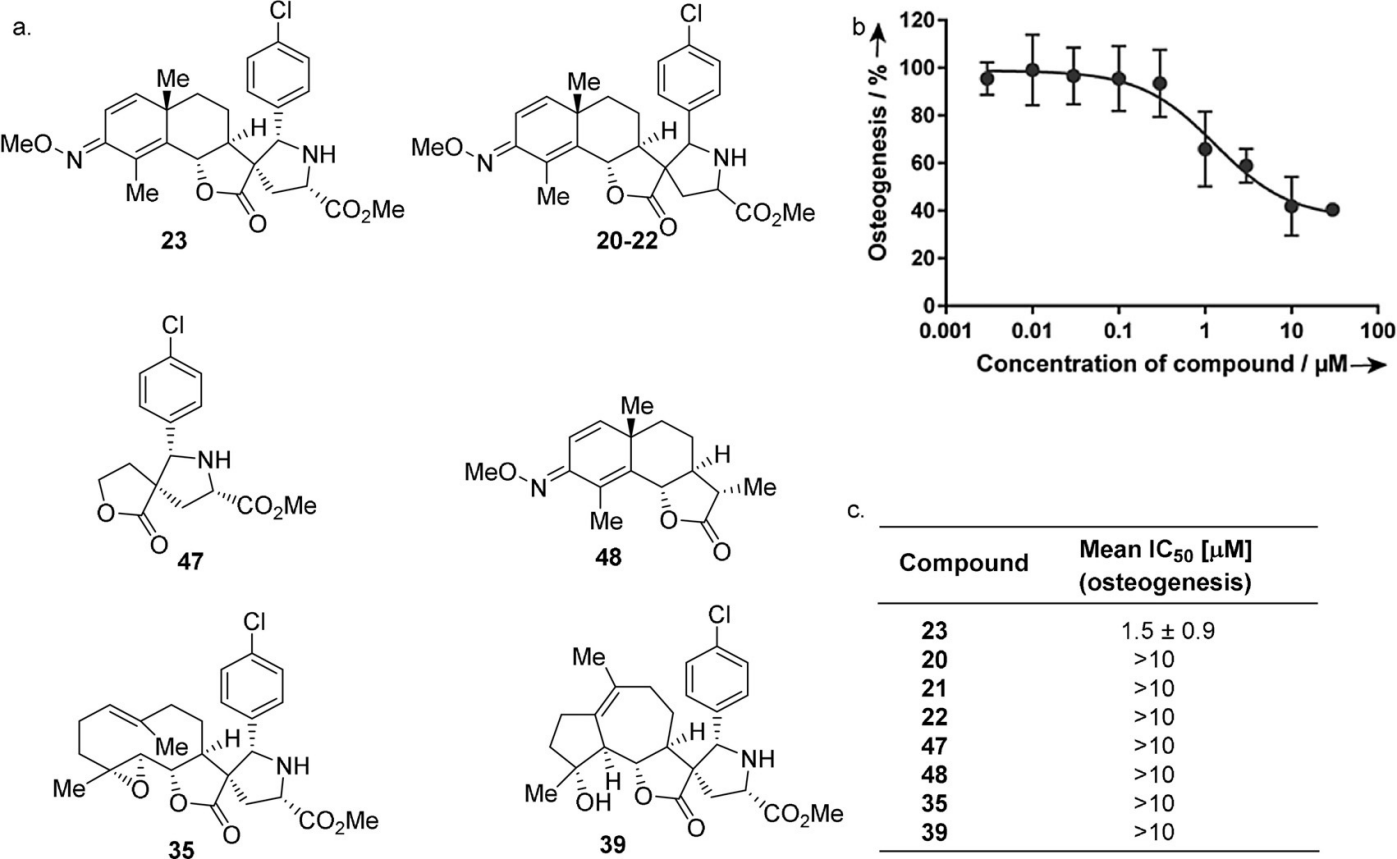
Pseudo‐sesquiterpenoid alkaloid **23** defines a new chemotype inhibiting Hedgehog‐dependent differentiation of multipotent murine mesenchymal progenitor stem cells into osteoblasts. a) Structures of compound **23** and its analogs. b) Hh‐dependent osteoblast differentiation assay. Data are mean values ±SD and representative of four biological replicates, each performed in three technical replicates. c) IC_50_ values of compounds depicted in (a).

Hh signaling is essential for embryonic and post‐embryonic development and regeneration.^[41]^ It has been linked to cancers like basal cell carcinoma and medulloblastoma,^[42]^ and novel Hh inhibitor classes are in high demand. To identify inhibitors of Hh signaling, we performed a Hh‐dependent osteoblast differentiation assay using C3H/10T1/2 cells (Figure 7).^[43]^ The pathway agonist purmorphamine was employed to induce osteoblast differentiation which leads to the expression of alkaline phosphatase as readout for pathway activity.^[44]^ This assay revealed that compound **23** inhibits Hh‐dependent osteoblast differentiation with half‐maximal inhibitory concentration (IC_50_) of 1.5±0.9 μM. Very notably, stereoisomeric pseudo‐sesquiterpenoid alkaloids **20**–**22** showed no inhibitory activity at 10 μM concentration. Under these conditions, also compounds **47** and **48** which are partial structures of the new inhibitor chemotype and pseudo‐sesquiterpenoid alkaloids **35** and **39** were inactive. The results indicate that combination of ring distortion and the pseudo‐NP strategy may enable the discovery of novel bioactive chemical matter in a more general sense.

## Conclusion

We describe the combination of the pseudo‐natural product principle with chemical ring distortion of NPs and a formal adaptation of the complexity‐to‐diversity strategy, to yield novel bioactive natural product‐inspired compound classes. In this new strategy, in general, on the one hand diverse scaffolds are formed from fragment‐sized NPs by subjecting them to ring‐distortion reactions. On the other hand, fragment‐sized NPs are directly employed (i.e. without separate ring distortion reaction) whose scaffolds can be regarded as formed by means of a ring distortion reaction, for example, through biosynthesis pathways. The second option is to apply ring distortion strategy which requires that chemical transformations should be used to distort the scaffolds of natural products. Subsequently, chemical fragment recombination through complexity‐generating transformations affords complex and diverse compound collections. We exemplify the new principle by the synthesis of a collection of pseudo‐sesquiterpenoid alkaloids obtained from fragment‐sized sesquiterpenes by means of stereocomplementary 1,3‐dipolar cycloadditions with azomethine ylides resulting in the highly stereoselective formation of pyrrolidine fragments. Cheminformatic and biological characterization revealed that the compound collection is stereochemically diverse and that this diversity is reflected in diverse biological performance, including the discovery of a novel chemotype inhibiting Hedgehog‐dependent differentiation of multipotent murine mesenchymal progenitor stem cells into osteoblasts.

Combination of these two complementary principles will give efficient access to new, natural product‐inspired, structurally complex compound classes, which are not accessible by currently described biosynthesis pathways. However, we note that silent, unexploited biosynthesis pathways exist,^[45]^ and that biosynthesis pathways can be reassembled, for example, in systems biology approaches.^[46]^ It is, therefore, possible that natural product scaffolds exist in nature which resemble the structures of the new NP‐inspired compound classes resulting from our approach thereby further validating it. In agreement with this notion, very recently and while we developed the principle, the isolation of the natural product vlasoulamine A was reported,^[47]^ whose scaffold is structurally related to pseudo‐sesquiterpenoid alkaloids **36**.

## Conflict of interest

The authors declare no conflict of interest.

## Supporting information

As a service to our authors and readers, this journal provides supporting information supplied by the authors. Such materials are peer reviewed and may be re‐organized for online delivery, but are not copy‐edited or typeset. Technical support issues arising from supporting information (other than missing files) should be addressed to the authors.

Supporting Information
