# Supplementary material for: Combination of Pseudo‐Natural Product Design and Formal Natural Product Ring Distortion Yields Stereochemically and Biologically Diverse Pseudo‐Sesquiterpenoid Alkaloids
Source: Angew Chem Int Ed Engl. 2021 Aug 19;60(39):21384–95. doi: 10.1002/anie.202106654 (PMC8518946; doi:10.1002/anie.202106654)
Supplement: Supplementary file 1 — Supporting Information [file ANIE-60-21384-s001.pdf]

Angewandte Chemie
Eine Zeitschrift der Gesellschaft Deutscher Chemiker

Supporting Information

Combination of Pseudo-Natural Product Design and Formal Natural Product Ring Distortion Yields Stereochemically and Biologically Diverse Pseudo-Sesquiterpenoid Alkaloids

*Jie Liu, Jana Flegel, Felix Otte, Axel Pahl, Sonja Sievers, Carsten Strohmann, and Herbert Waldmann**

anie_202106654_sm_miscellaneous_information.pdf

Table of Contents

1. Supplementary Figures and Tables 2

2. Cell painting methodology 22

3. Overview of all molecular structures 25

4. General information 29

5. Experimental details and analytic data for pseudo-sesquiterpenoid alkaloids 30

6. References 91

7. NMR spectra 92

    7.1 NMR spectra of santonin-pyrrolidines 92

    7.2 NMR spectra of SLs derivatives 153

    7.3 NMR spectra of diverse sesquiterpenoid alkaloids (**20-47**) 161

8. Biology experiment 222

1. Supplementary Figures and Tables

Table S1. Condition screening of stereodivergent synthesis of pseudo-sesquiterpenoid alkaloids.

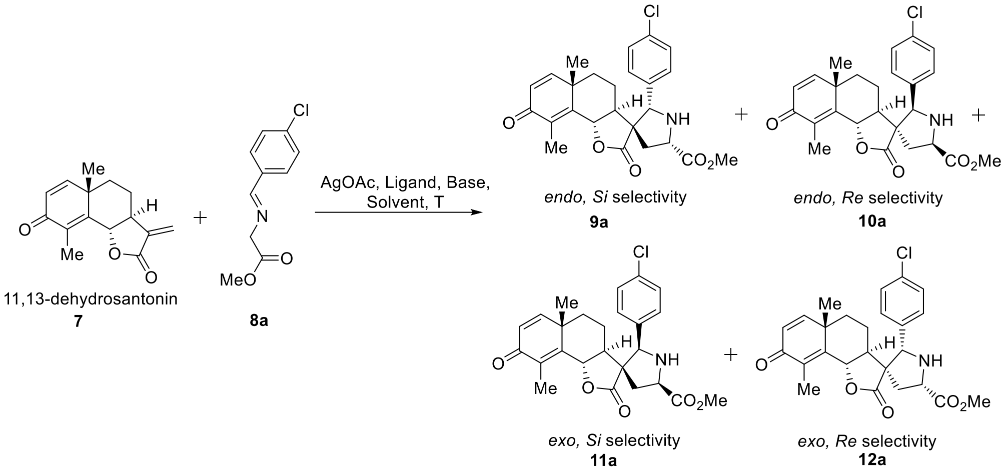

Entry	catalyst	ligand	solvent	Base	T	Yield [%][a]	d.r. [a] (**9a**:**10a**:**11a**:**12a**)
1	AgOAc	**none**	DCM	Et_3N	r.t.	74%	72:23:04:01
2	AgOAc	**PPh_3**	DCM	Et_3N	r.t.	79%	68:18:11:03
3	AgOAc	**L1**	DCM	Et_3N	r.t.	93%	21:06:71:02
4	AgOAc	***ent*-L1**	DCM	Et_3N	r.t.	78%	22:14:19:45
5	AgOAc	**L2**	DCM	Et_3N	r.t.	88%	60:05:09:26
6	AgOAc	**L3**	DCM	Et_3N	r.t.	91%	89:00:11:00
7	AgOAc	**L4**	DCM	Et_3N	r.t.	99%	08:02:89:00
8	$CuBF_4$	**L3**	DCM	Et_3N	r.t.	47%	67:20:10:03
9	AgOAc	**L3**	DCM	Et_3N	0 °C	99%	90:00:10:00
10	AgOAc	**L3**	DCM	Et_3N	-20 °C	29%	82:00:18:00
11	AgOAc	**L3**	THF	Et_3N	r.t.	82%(73%) [b]	91:02:07:00
12	AgOAc	**L3**	DCE	Et_3N	r.t.	78%	88:01:11:00
13	$CuBF_4$	**L4**	DCM	Et_3N	r.t.	75%	07:02:89:02
14	AgOAc	**L4**	THF	Et_3N	r.t.	83%	07:25:67:00
15	AgOAc	**L4**	DCE	Et_3N	r.t.	92%(89%) [b]	00:00:100:00
16	AgOAc	***ent*-L3**	DCM	Et_3N	r.t.	73%	07:89:01:02
17	$CuBF_4$	***ent*-L3**	DCM	Et_3N	r.t.	Low conv.	-

Table S1 (Continued). Condition screening of stereodivergent synthesis of pseudo sesquiterpenoid alkaloids.

Entry	catalyst	ligand	solvent	Base	T	Yield [%][a]	d.r. [a] (**9a**:**10a**:**11a**:**12a**)
18	AgOAc	***ent*-L3**	DCM	Et_3N	0 ºC	68%	00:94:04:02
19	AgOAc	***ent*-L3**	THF	Et_3N	0 ºC	79%(95%) [b]	03:95:01:01
20	AgOAc	***ent*-L4**	DCM	Et_3N	r.t.	68%	28:01:03:68
21	$CuBF_4$	***ent*-L4**	DCM	Et_3N	r.t.	40%	54:24:13:09
22	AgOAc	**L6**	DCM	Cs_2CO_3	r.t.	82%	38:07:05:51
23	AgOAc	**L7**	DCM	Cs_2CO_3	r.t.	82%	15:13:71:00
24	AgOAc	**L8**	DCM	Cs_2CO_3	r.t.	95%	43:28:17:12
25	AgOAc	**L9**	DCM	Cs_2CO_3	r.t.	66%	28:44:17:10
26	AgOAc	**L5**	DCM	Cs_2CO_3	r.t.	64%	24:00:01:75
27	AgOAc	**L5**	DCM	Cs_2CO_3	r.t.	64%	24:00:01:75
28	AgOAc	**L5**	PhMe	Cs_2CO_3	r.t.	62%	79:15:01:05
29	AgOAc	**L5**	DCE	Cs_2CO_3	r.t.	71%	35:00:04:61
30	AgOAc	**L5**	Et_2O	Cs_2CO_3	r.t.	79%	82:09:01:08
31	AgOAc	**L5**	THF	Cs_2CO_3	r.t.	53%	88:00:00:12
32	AgOAc	**L5**	$CHCl_3$	Cs_2CO_3	r.t.	77%	13:00:02:86
33	AgOAc	**L5**	CCl_4	Cs_2CO_3	r.t.	66%	40:03:04:53
34	AgOAc	**L5**	CH_3CN	Cs_2CO_3	r.t.	28%	48:00:02:50
35	AgOAc	**L5**	Acetone	Cs_2CO_3	r.t.	38%	54:00:02:44
36	AgOAc	**L5**	$CDCl_3$	Cs_2CO_3	r.t.	77%	11:00:03:86
37	AgOAc	**L5**	$CHCl_3$	Cs_2CO_3	0 ºC	48%	07:00:01:93
38[c]	AgOAc	**L5**	$CHCl_3$	Cs_2CO_3	0 ºC	72%(88%) [b	04:00:00:96
39[c]	AgOAc	***ent*-L4**	$CHCl_3$	Cs_2CO_3	0 ºC	68%	10:00:02:88

The reaction was performed on 0.1 mmol scale, Schiff base (1.5 eq.), catalyst (5% mol), ligand (6% mol), base (0.5 equiv.), solvent (1 mL), 24h. a. Determined by the crude ^1H NMR with CH_2Br_2 as the internal standard. Yields of all stereoisomers. b. Isolation yield of main isomer. c. 2.0 eq. of iminoester, 48 h.

Table S1 (Continued). Condition screening of stereodivergent synthesis of pseudo sesquiterpenoid alkaloids.

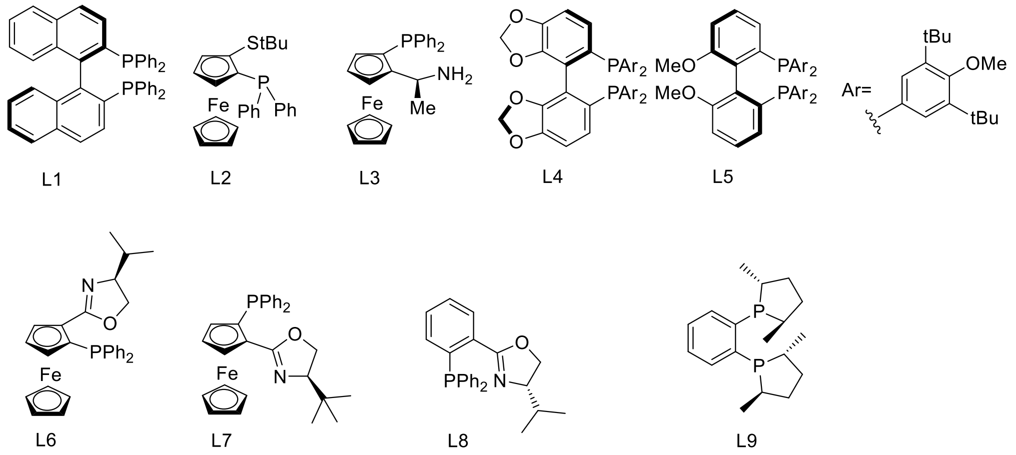

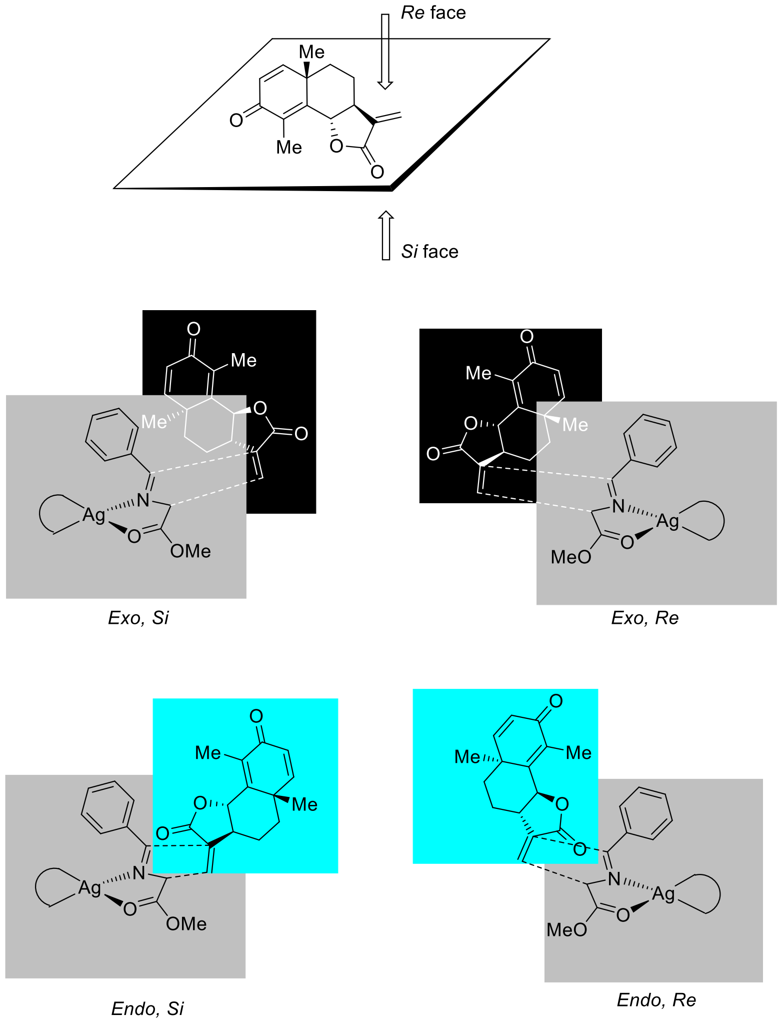

Figure S1. Proposed transition states of stereodivergent 1,3-DC. In the *exo*,*Re*-selectivity, the repulsion between quaternary methyl group on santonin scaffold and azomethine ylide made such a pathway less favored (mismatched cases).

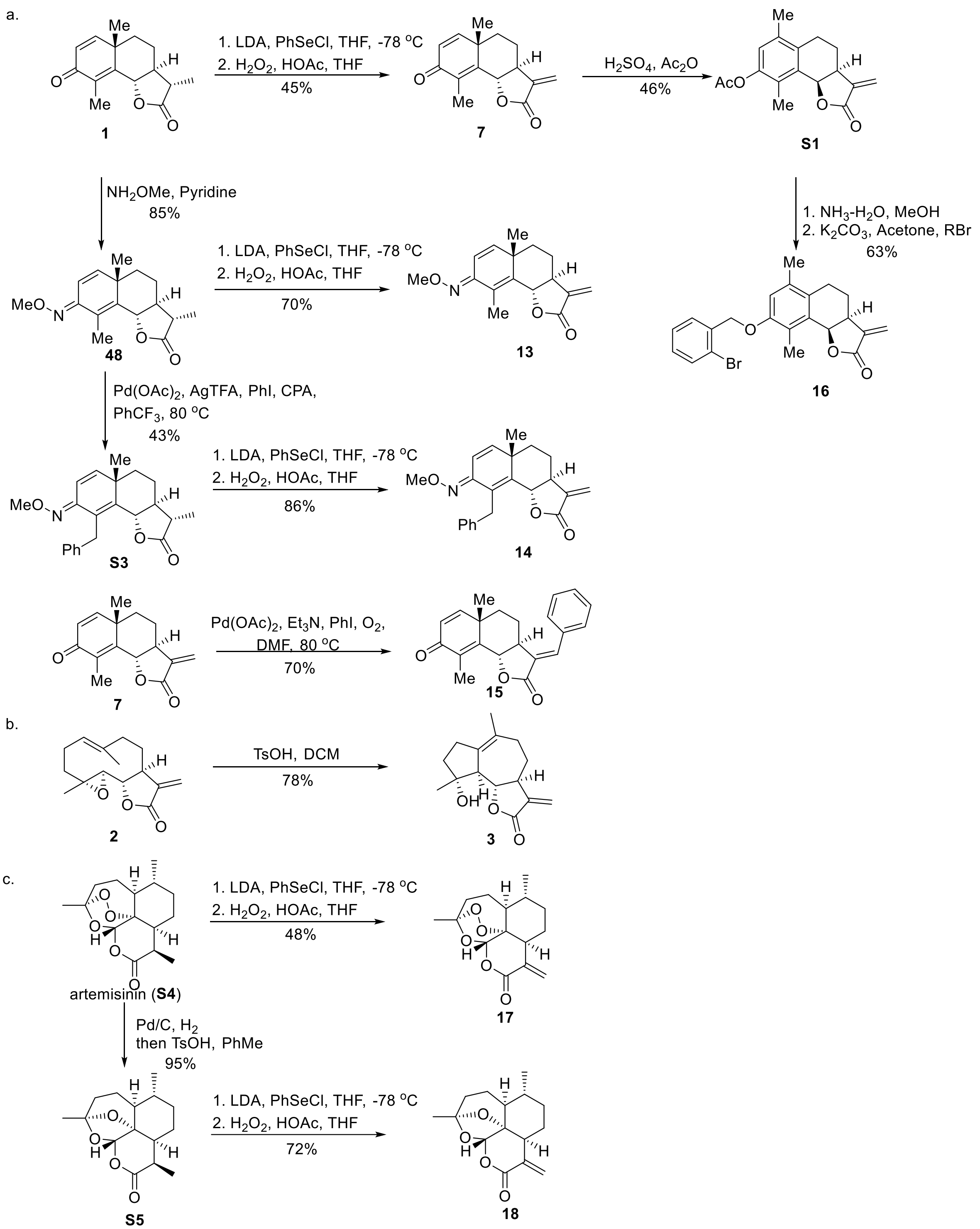

Figure S2. Synthetic pathways to SLs library. Three subgroups of SLs were synthesized from commercially available santonin, parthenolide and artemisinin respectively.

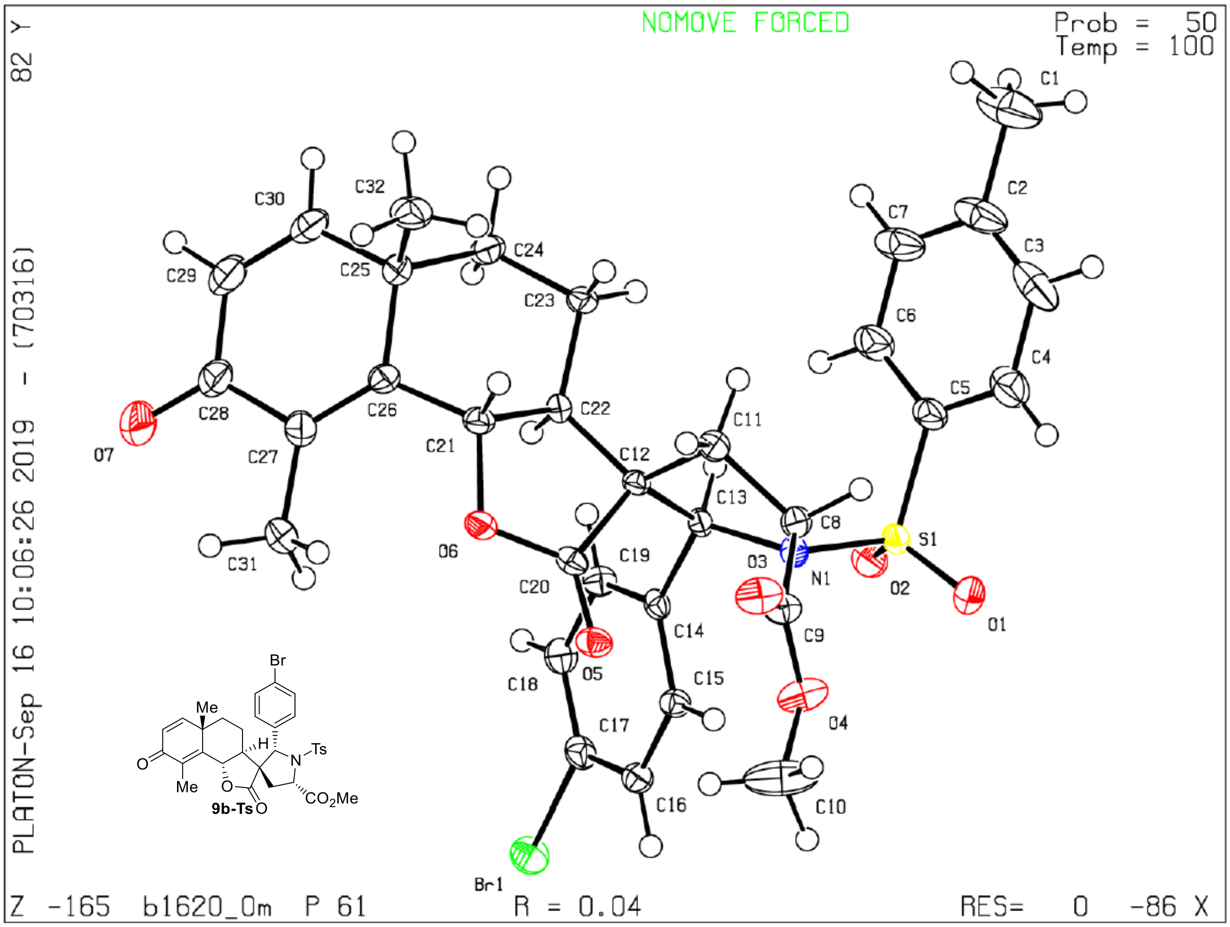

Figure S3. Crystal structure of the Ts protected cycloadduct **9b-Ts**. ORTEP plot of C32H32BrNO7S (M =654.55 g/mol) at the 50% probability level. See Supplementary Table S2 for additional details. Crystallographic data have been deposited at the Cambridge Crystallographic Data Centre and copies can be obtained on request, free of charge, by quoting the publication citation and the deposition number CCDC 2055830.

Table S2. Crystal data and structure refinement for **9b-Ts**.

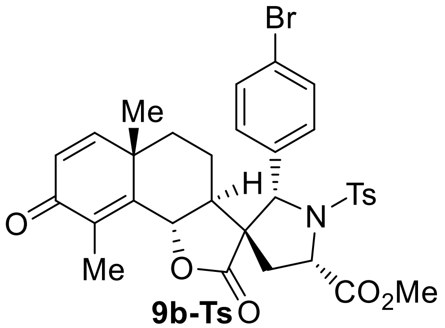

9b-Ts

Empirical formula	$C_{32}H_{32}BrNO_7S$
Formula weight	654.55
Temperature/K	100.0
Crystal system	hexagonal
Space group	$P6_1$
a/Å	16.1442(17)
b/Å	16.1442(17)
c/Å	22.1544(15)
α/°	90
β/°	90
γ/°	120
Volume/Å^3	5000.6(11)
Z	6
ρ_{calc}g/cm^3	1.304
μ/mm^{-1}	1.340
F(000)	2028.0
Crystal size/mm^3	0.436 × 0.239 × 0.132
Radiation	MoKα (λ = 0.71073)
2Θ range for data collection/°	5.828 to 57.994
Index ranges	$-22 \leqslant h \leqslant 22$, $-22 \leqslant k \leqslant 21$, $-30 \leqslant l \leqslant 29$
Reflections collected	50608
Independent reflections	8817 [R_{int} = 0.0389, R_{sigma} = 0.0439]
Data/restraints/parameters	8817/1/383
Goodness-of-fit on F^2	1.035
Final R indexes [I>=2σ (I)]	R_1 = 0.0374, wR_2 = 0.0978
Final R indexes [all data]	R_1 = 0.0408, wR_2 = 0.0999
Largest diff. peak/hole / e Å^{-3}	0.39/-0.30
Flack parameter	0.022(3)

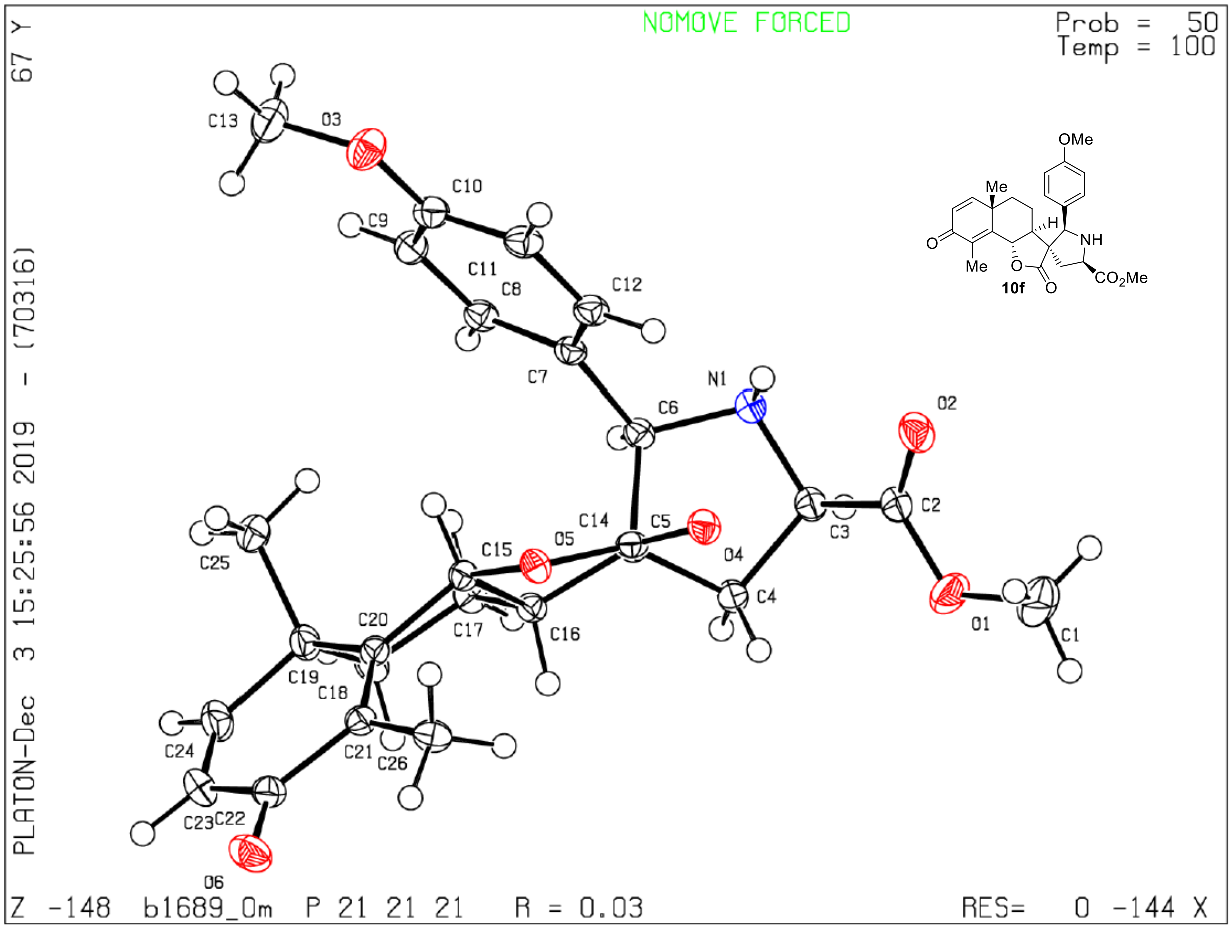

Figure S4. Crystal structure of the cycloadduct **10f**. ORTEP plot of C26H29NO6 (M =451.50 g/mol) at the 50% probability level. See Supplementary Table S3 for additional details. Crystallographic data have been deposited at the Cambridge Crystallographic Data Centre and copies can be obtained on request, free of charge, by quoting the publication citation and the deposition number CCDC 2055829.

Table S3. Crystal data and structure refinement for **10f**.

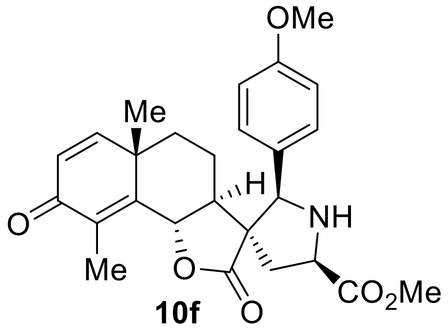

Empirical formula	$C_{26}H_{29}NO_6$
Formula weight	451.50
Temperature/K	100.0
Crystal system	orthorhombic
Space group	$P2_12_12_1$
a/Å	6.38220(10)
b/Å	12.9293(3)
c/Å	27.4615(6)
α/°	90
β/°	90
γ/°	90
Volume/Å^3	2266.05(8)
Z	4
ρ_{calc}g/cm^3	1.323
μ/mm^{-1}	0.768
F(000)	960.0
Crystal size/mm^3	0.678 × 0.048 × 0.04
Radiation	CuKα (λ = 1.54178)
2Θ range for data collection/°	6.436 to 159.68
Index ranges	$-8 \leqslant h \leqslant 8, -16 \leqslant k \leqslant 16, -35 \leqslant l \leqslant 33$
Reflections collected	75692
Independent reflections	4876 [R_{int} = 0.0279, R_{sigma} = 0.0091]
Data/restraints/parameters	4876/0/306
Goodness-of-fit on F^2	1.058
Final R indexes [I>=2σ (I)]	R_1 = 0.0270, wR_2 = 0.0718
Final R indexes [all data]	R_1 = 0.0272, wR_2 = 0.0721
Largest diff. peak/hole / e Å^{-3}	0.21/-0.17
Flack parameter	-0.02(3)

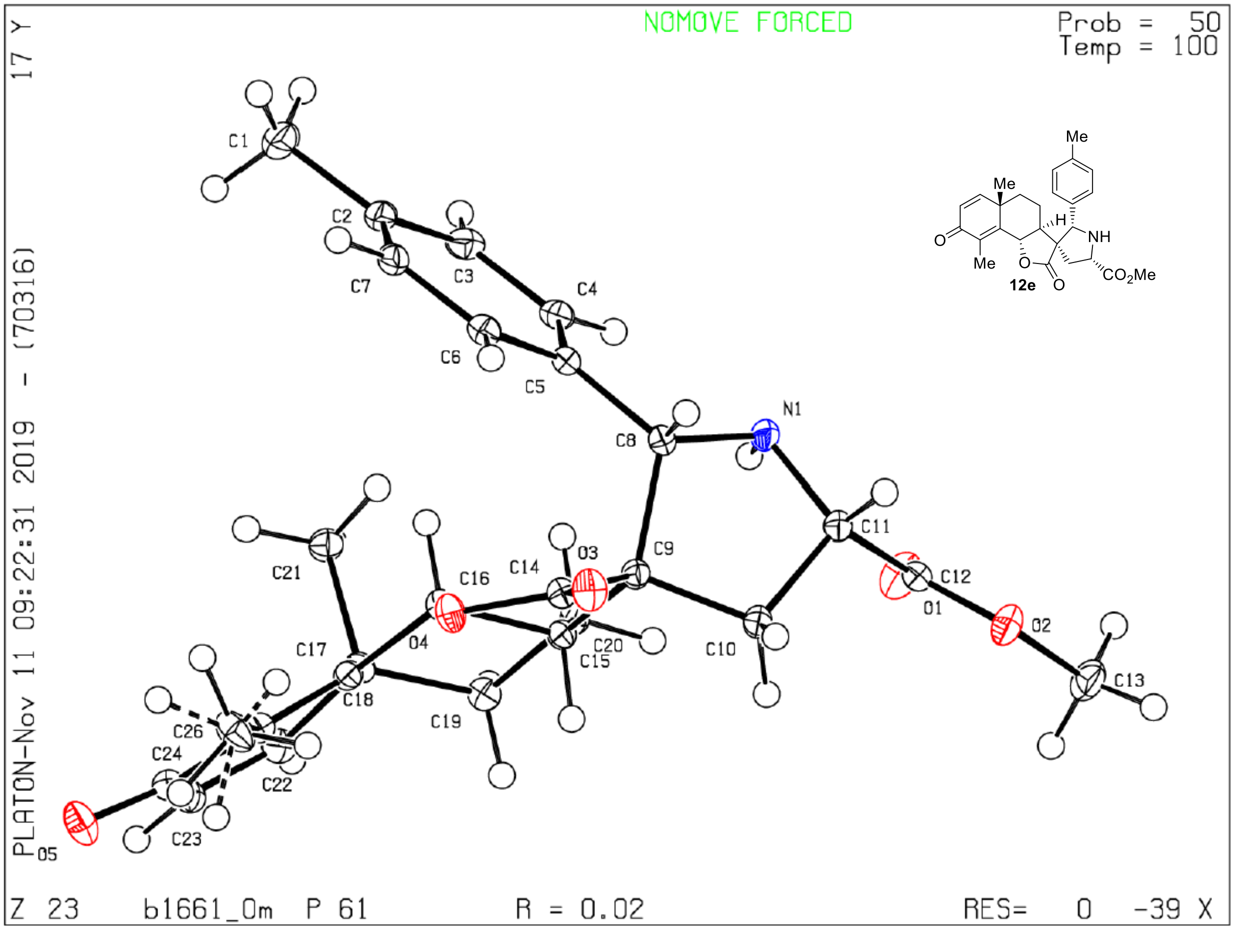

Figure S5. Crystal structure of the cycloadduct **12e**. ORTEP plot of C26H29NO5 (M =435.50 g/mol) at the 50% probability level. See Supplementary Table S4 for additional details. Crystallographic data have been deposited at the Cambridge Crystallographic Data Centre and copies can be obtained on request, free of charge, by quoting the publication citation and the deposition number CCDC 2055831.

Table S4. Crystal data and structure refinement for **12e**.

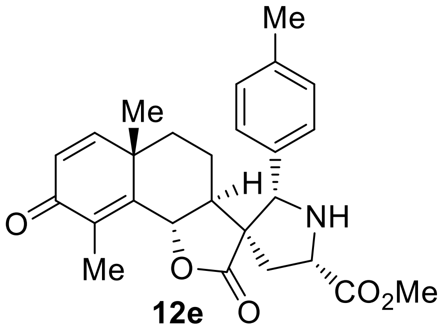

12e

Empirical formula	$C_{26}H_{29}NO_5$
Formula weight	435.50
Temperature/K	100.0
Crystal system	hexagonal
Space group	$P6_1$
a/Å	11.5596(2)
b/Å	11.5596(2)
c/Å	27.5631(6)
α/°	90
β/°	90
γ/°	120
Volume/Å^3	3189.66(13)
Z	6
ρ_{calc}g/cm^3	1.360
μ/mm^{-1}	0.762
F(000)	1392.0
Crystal size/mm^3	0.738 × 0.161 × 0.108
Radiation	CuKα (λ = 1.54178)
2Θ range for data collection/°	8.832 to 159.24
Index ranges	-14 ⩽ h ⩽ 14, -14 ⩽ k ⩽ 14, -30 ⩽ l ⩽ 34
Reflections collected	113217
Independent reflections	4514 [R_{int} = 0.0229, R_{sigma} = 0.0070]
Data/restraints/parameters	4514/1/310
Goodness-of-fit on F^2	1.038
Final R indexes [I>=2σ (I)]	R_1 = 0.0245, wR_2 = 0.0647
Final R indexes [all data]	R_1 = 0.0245, wR_2 = 0.0647
Largest diff. peak/hole / e Å^{-3}	0.18/-0.17
Flack parameter	-0.05(4)

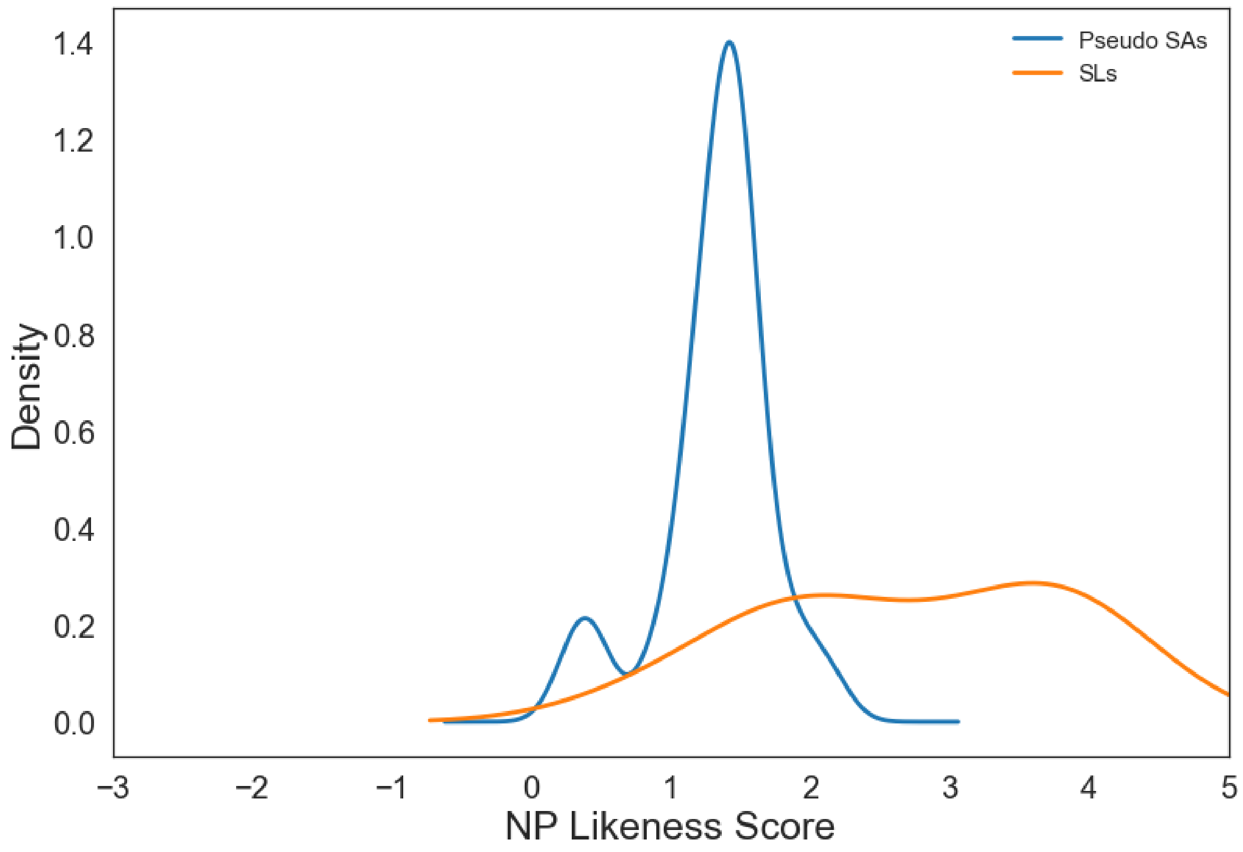

Figure S6. NP likeness scores of synthetic pseudo natural products. An obvious decrease of NP-likeness score was observed after incorporation of pyrrolidine moiety to the SLs.

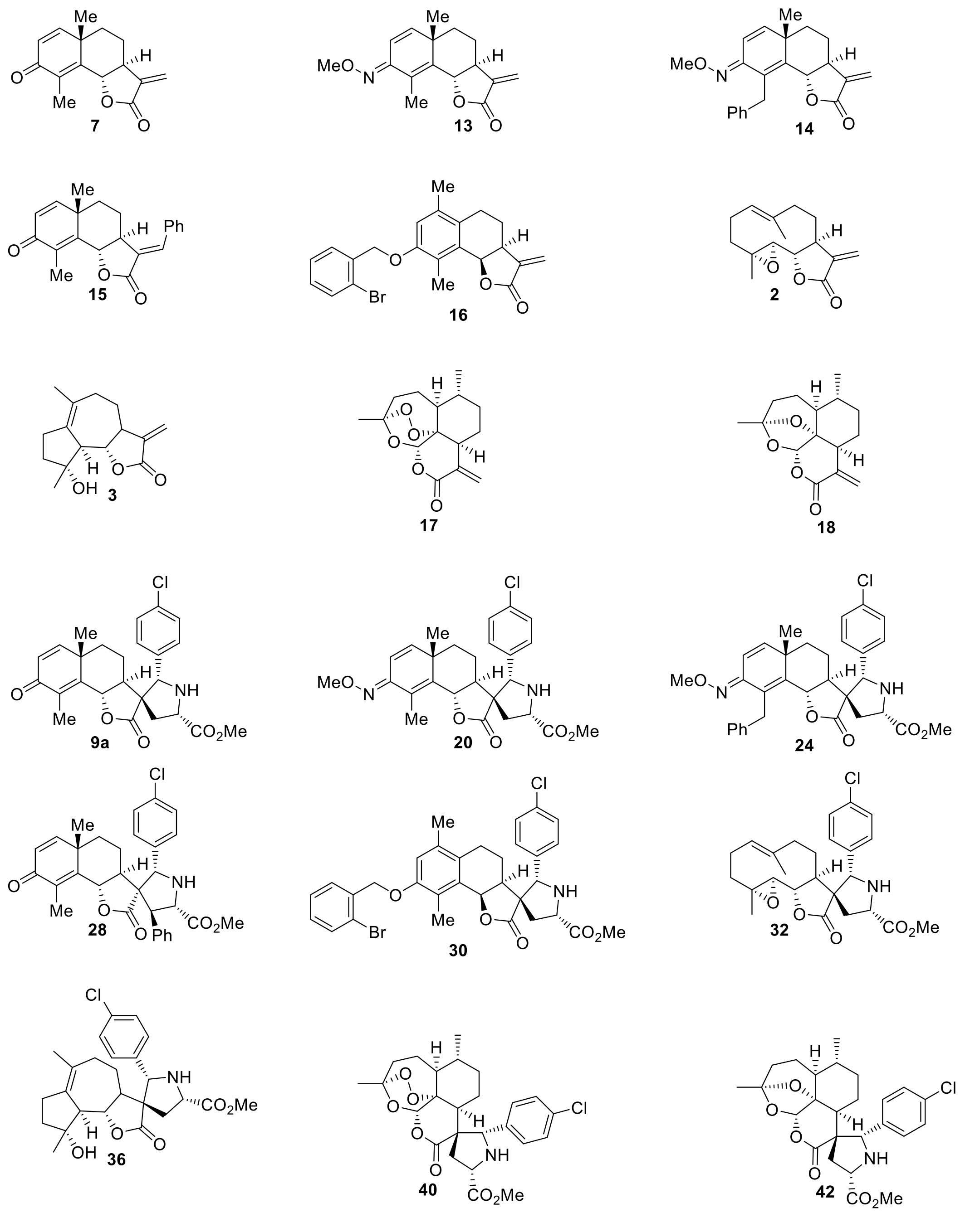

Figure S7. Compound list for the Tanimoto cross similarity analysis.

Table S5. Induction for all compounds. Some compounds were not tested (n.d.) because of the limited amount.

No.	Induction @ 10 µM	Induction @ 30 µM	Induction @ 50 µM	*No.*	Induction @ 10 µM	Induction @ 30 µM	Induction @ 50 µM
**7**	36.6	94.8	95.0	**2**	44.4	94.5	n.d.
**13**	89.6	95.5	92.4	**3**	14.2	70.6	93.6
**14**	41.8	92.1	94.6	**17**	53.7	95.3	94.1
**15**	13.5	29.7	18.7	**18**	19.7	44.0	34.9
**16**	58.5	92.4	73.1				
**9a**	0.2	9.5	11.9	**9i**	0.3	1.4	7.1
**9b**	0.0	9.3	10.7	**9j**	0.0	25.9	25.0
**9c**	0.0	0.9	2.1	**9k**	0.0	1.6	16.1
**9d**	0.3	14.3	10.7	**9l**	0.0	6.9	1.4
**9e**	0.0	0.2	0.3	**9m**	0.0	1.4	12.3
**9f**	0.2	11.1	3.3	**9n**	0.3	2.4	2.8
**9g**	0.0	1.0	10.4	**9o**	0.0	3.3	2.1
**9h**	0.0	2.6	1.7	**9p**	0.0	3.3	1.7
**10a**	1.6	2.6	15.0	**10i**	1.0	36.1	25.2
**10b**	0.7	2.6	4.1	**10j**	0.9	12.4	35.2
**10c**	4.7	0.3	4.8	**10k**	0.3	3.1	6.4
**10d**	0.5	0.3	2.1	**10l**	1.0	6.6	8.3
**10e**	1.4	11.1	7.1	**10m**	0.3	0.9	3.5
**10f**	n.d.	n.d.	n.d.	**10n**	0.9	5.7	8.3
**10g**	0.3	0.7	0.3	**10o**	0.7	1.2	6.2
**10h**	n.d.	n.d.	n.d.	**10p**	0.2	2.8	10.2
**11a**	0.2	3.5	2.4	**11i**	0.5	3.8	11.4
**11b**	1.0	16.6	16.6	**11j**	0.2	14.5	8.5
**11c**	0.0	0.3	2.4	**11k**	0.5	18.8	6.2
**11d**	0.3	3.3	6.9	**11l**	0.0	0.7	3.8
**11e**	0.0	0.9	4.5	**11p**	0.0	0.7	0.3
**11f**	0.5	8.5	5.9				
**11g**	0.0	3.3	6.7				
**11h**	0.3	2.1	0.7				

Table S5 (Continued). Induction for all compounds. Some compounds were not tested (n.d.) because of the limited amount.

No.	**Induction @ 10 µM**	**Induction @ 30 µM**	**Induction @ 50 µM**	*No.*	**Induction @ 10 µM**	**Induction @ 30 µM**	**Induction @ 50 µM**
**12a**	n.d.	n.d.	n.d.	**12i**	n.d.	n.d.	n.d.
**12b**	0.9	2.9	16.6	**12j**	3.3	7.3	25.9
**12c**	0.0	0.2	0.7	**12k**	0.7	5.7	12.3
**12d**	0.7	14.5	18.1	**12l**	0.5	0.7	4.1
**12e**	1.4	3.8	8.6	**12m**	0.0	2.1	6.7
**12f**	0.3	0.3	1.6	**12n**	0.3	4.1	6.7
**12g**	0.3	1.7	0.9	**12o**	0.3	0.5	1.9
**12h**	0.5	0.0	1.6	**12p**	0.2	0.0	1.2
**20**	0.9	6.9	24.5	**35**	0.3	1.4	10.2
**21**	0.3	3.6	21.1	**36**	0.7	16.6	22.5
**22**	6.4	22.8	39.7	**37**	0.2	1.9	2.8
**23**	5.0	42.1	64.1	**38**	0.3	0.2	1.6
**24**	4.8	11.7	12.3	**39**	0.0	5.4	0.7
**25**	1.7	23.3	30.6	**40**	0.3	n.d.	n.d.
**26**	7.8	26.9	28.0	**41**	0.0	26.4	20.4
**27**	7.8	30.1	35.4	**42**	1.0	10.0	3.6
**28**	1.4	12.1	13.1	**43**	0.0	2.9	11.7
**29**	0.5	5.2	14.2	**44**	0.0	0.0	0.7
**30**	2.8	5.7	10.9	**45**	0.0	0.0	0.3
**31**	14.5	13.0	15.5	**46**	0.3	0.9	0.0
**32**	1.2	15.9	22.3	**47**	0.0	0.2	0.0
**33**	1.9	3.1	14.9				
**34**	0.0	0.5	1.4				
Cell painting inductions of saturated sesquiterpene lactones							
**1**	0.0	0.0	0.0	**S4**	7.6	6.6	n.d.
**48**	1.6	2.4	7.6	**S5**	0.0	0.0	0.5
**S3**	0.0	1.0	2.2				

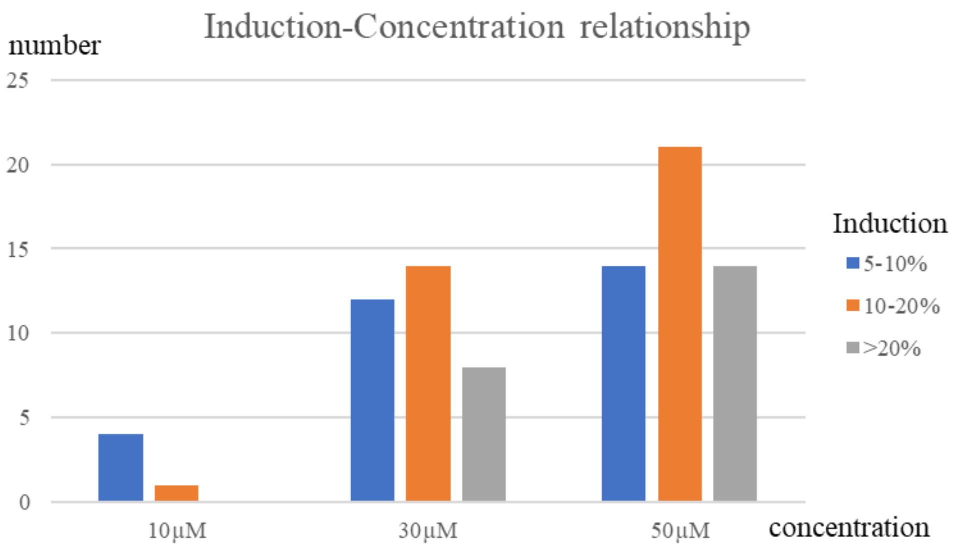

Figure S8. Cell painting induction analyses of pseudo sesquiterpenoid alkaloids. The induction value was concentration dependent, with more active values (>5%) under higher concentration.

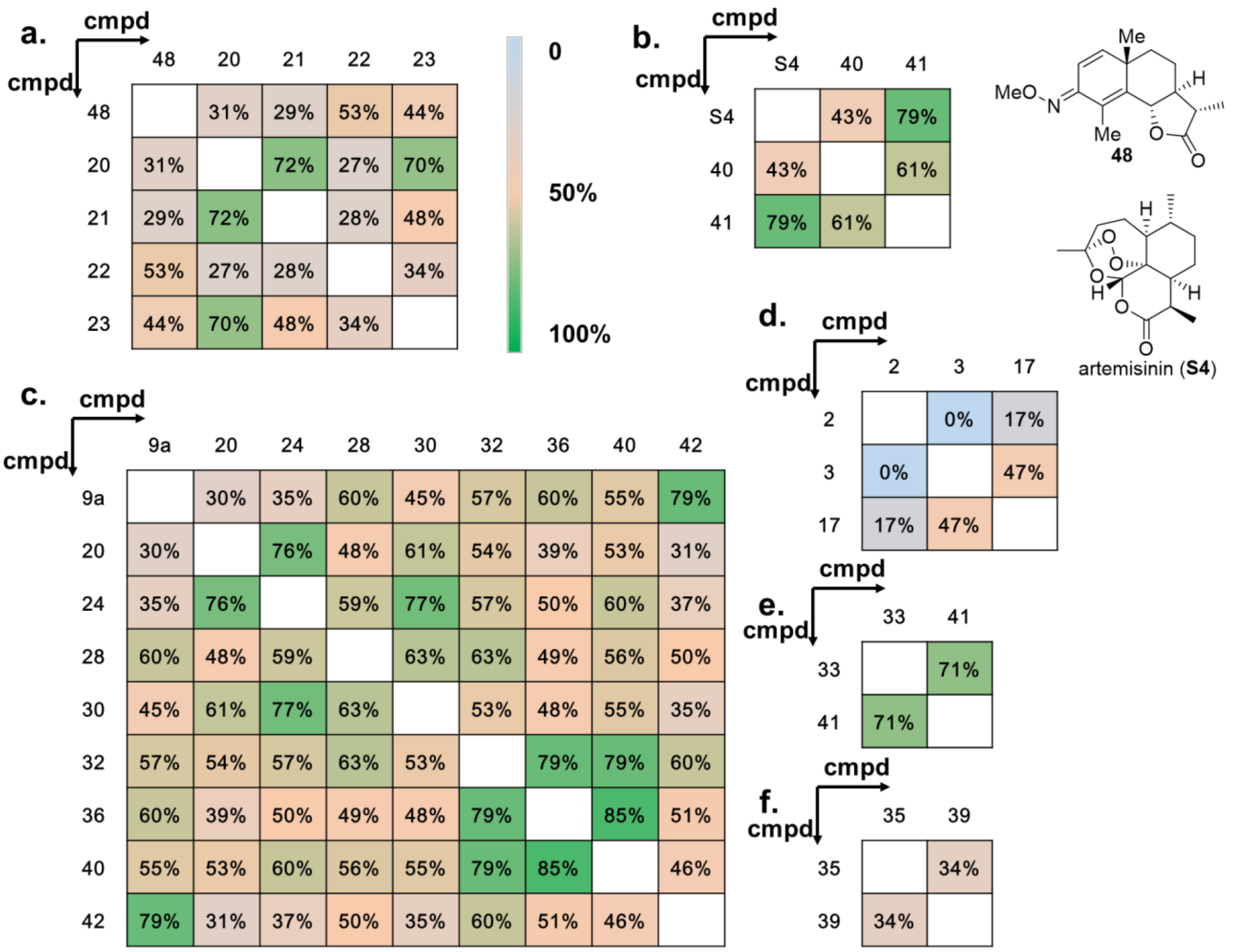

Figure S9. Cross biosimilarity between different pseudo sesquiterpenoid alkaloids with the same stereocenters of pyrrolidine fragment. Ring distortion played a significant role to lead to diverse biological performance. a. After recombination of pyrrolidine moiety, **20-23** displayed highly different biological performance compared to the saturated sesquiterpene lactone **48**. Induction scale: 5%-30%. b. After recombination of pyrrolidine moiety, **40** displayed highly different biological performance compared to the saturated artemisinin **S4**, while **41** led to relatively similar performance. Induction scale: 5%-30%. c. Diverse pseudo sesquiterpenoid alkaloids with different sesquiterpene scaffolds were compared together. Induction scale: 5%-30%. d. Cross biosimilarity of sesquiterpene lactones **2**, **3** and **17**. Induction scale: >5% at 10 µM. e. Cross biosimilarity of **33** and **41**. Induction scale: 5%-30%. f. Cross biosimilarity of **35** and **39**. Induction scale: 5%-30%.

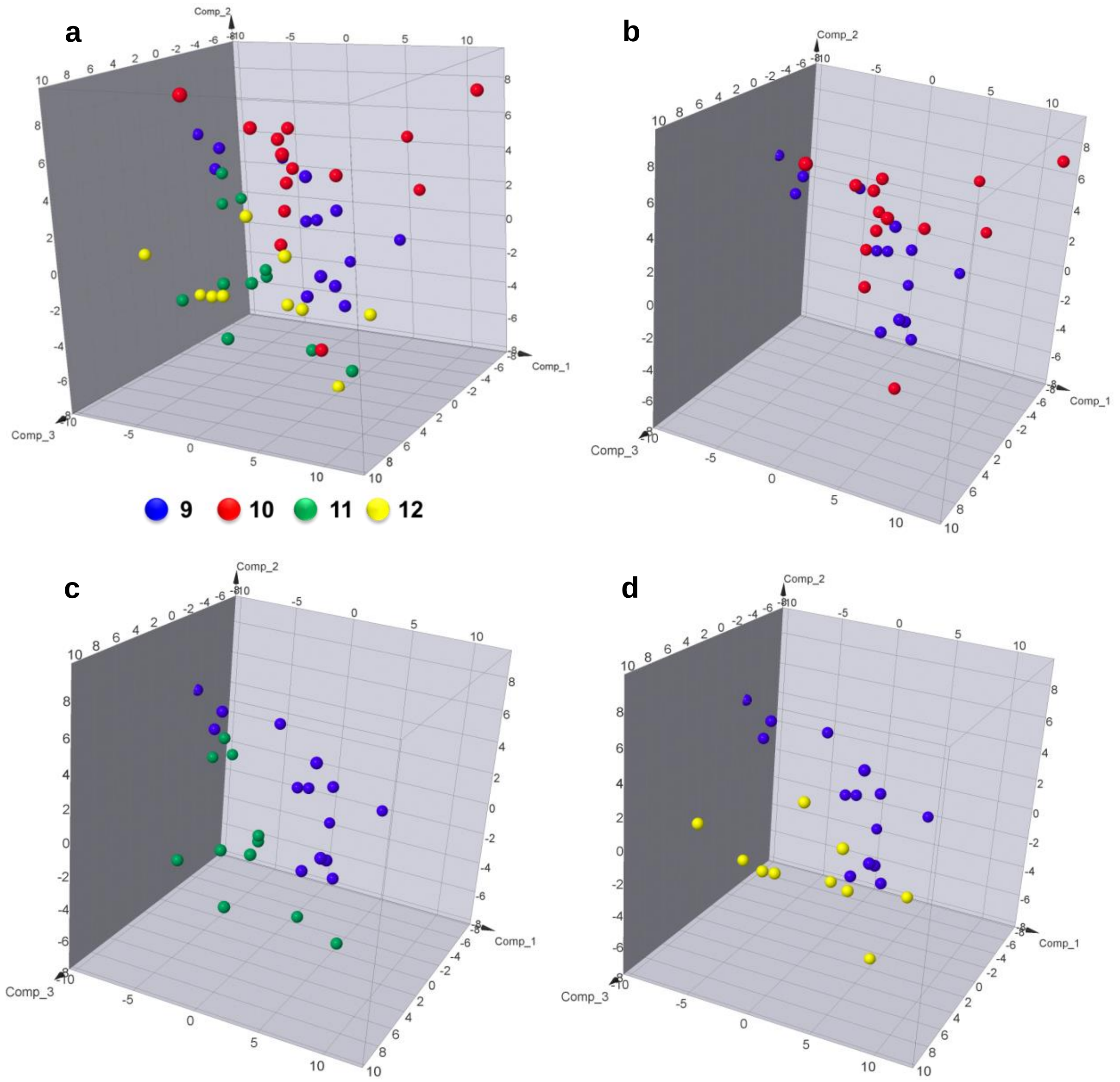

Figure S10. Santonin-pyrrolidine class **9**, **10**, **11** and **12** showed diverse biological performance. a. Principal component analysis of all stereoisomers based on santonin-pyrrolidine scaffold. b-g. Different pairs were shown based on Fig. S10a respectively to give a clear comparison.

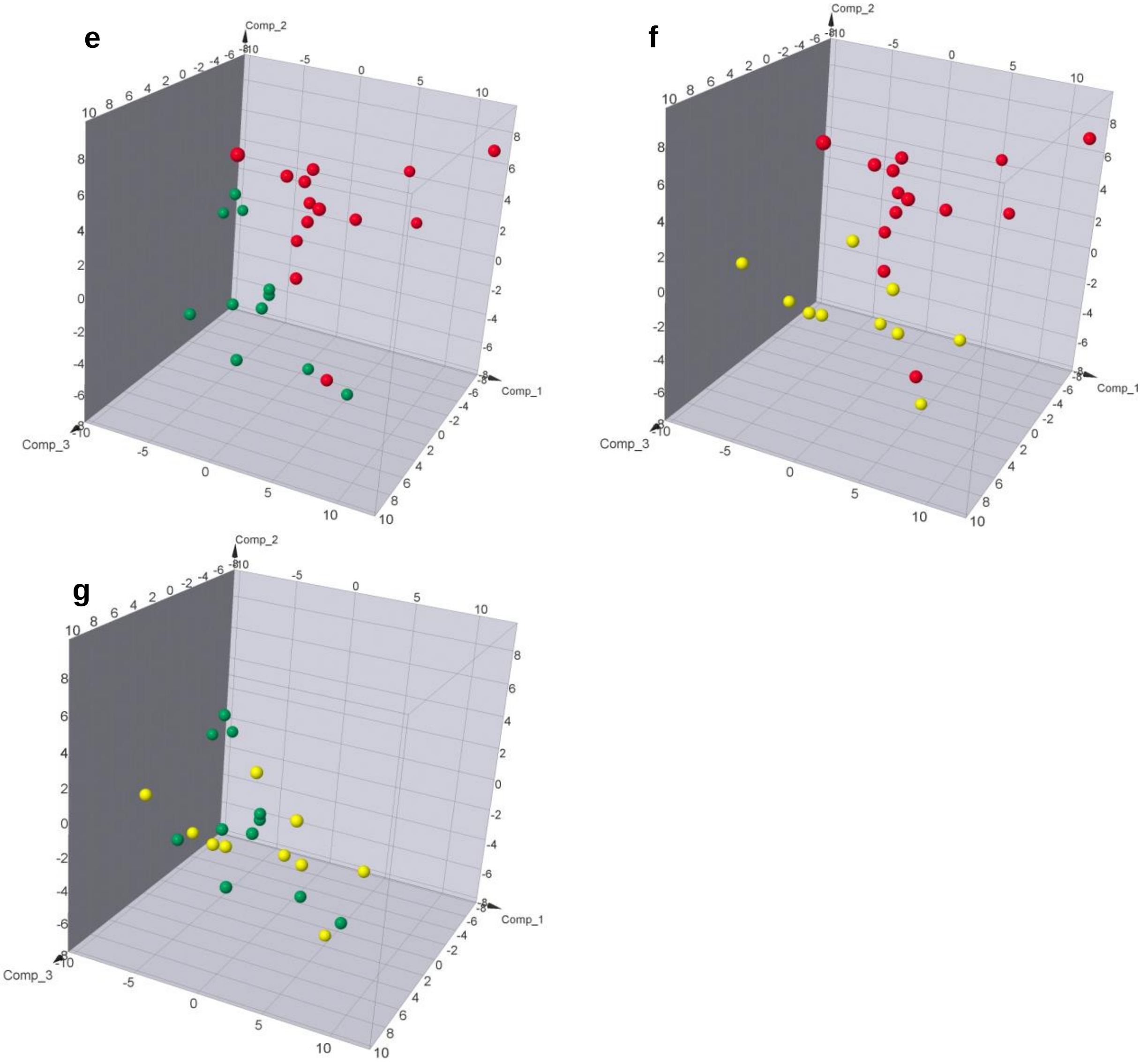

Figure S10 (Continued). Santonin-pyrrolidine class **9**, **10**, **11** and **12** showed diverse biological performance. a. Principal component analysis of all stereoisomers based on santonin-pyrrolidine scaffold. b-g. Different pairs were shown based on Fig. S10a respectively to give a clear comparison.

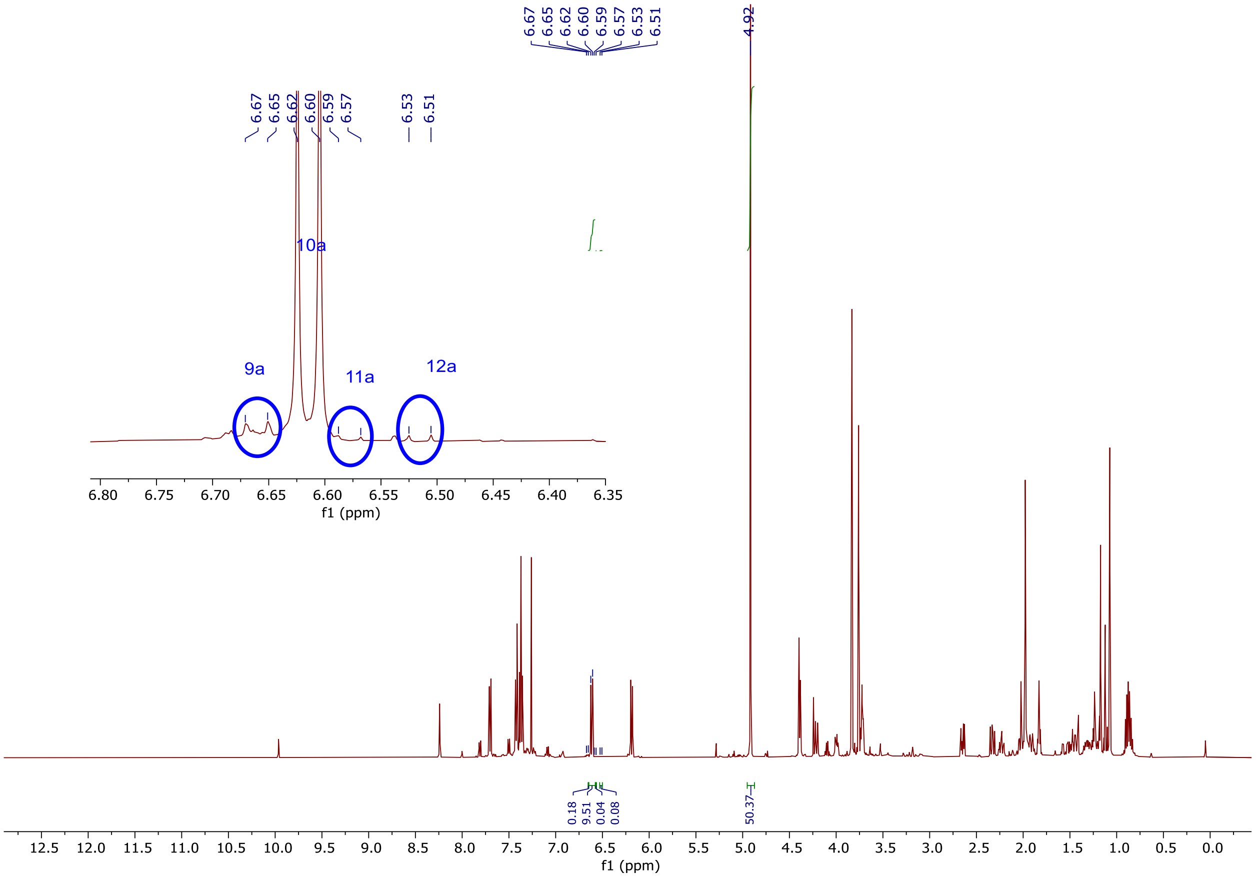

Figure S11. Representative crude ^{1}H NMR for entry 8 in Table 1.

2. Cell painting methodology

The described assay follows closely the method described by Bray et al[1].

Initially, 5 µl U2OS medium were added to each well of a 384-well plate (PerkinElmer CellCarrier-384 Ultra). Subsequently, U2OS cell were seeded with a density of 1600 cells per well in 20 µl medium. The plate was incubated for 10 min at the ambient temperature, followed by an additional 4 h incubation (37 °C, 5% CO_2). Compound treatment was performed with the Echo 520 acoustic dispenser (Labcyte) at final concentrations of 10 µM, 3 µM or 1 µM. Incubation with compound was performed for 20 h (37 °C, 5% CO2). Subsequently, mitochondria were stained with Mito Tracker Deep Red (Thermo Fisher Scientific, Cat. No. M22426). The Mito Tracker Deep Red stock solution (1 mM) was diluted to a final concentration of 100 nM in prewarmed medium. The medium was removed from the plate leaving 10 µl residual volume and 25 µl of the Mito Tracker solution were added to each well. The plate was incubated for 30 min in darkness (37 °C, 5% CO2). To fix the cells 7 µl of 18.5 % formaldehyde in PBS were added, resulting in a final formaldehyde concentration of 3.7 %. Subsequently, the plate was incubated for another 20 min in darkness (RT) and washed three times with 70 µl of PBS. (Biotek Washer Elx405). Cells were permeabilized by addition of 25 µl 0.1% Triton X-100 to each well, followed by 15 min incubation (RT) in darkness. The cells were washed three times with PBS leaving a final volume of 10 µl. To each well 25 µl of a staining solution were added, which contains 1% BSA, 50 µl Phalloidin (Alexa594 conjugate, Thermo Fisher Scientific, A12381), 25 µg/ml Concanavalin A (Alexa488 conjugate, Thermo Fisher Scientific, Cat. No. C11252), 50 µl/ml Hoechst 33342 (Sigma, Cat. No. B2261-25mg), 15 µl/ml WGA-Alexa594 conjugate (Thermo Fisher Scientific, Cat. No. W11262) and 0.3 µl/ml SYTO 14 solution (Thermo Fisher Scientific, Cat. No. S7576). The plate is incubated for 30 min (RT) in darkness and washed three times with 70 µl PBS. After the final washing step the PBS was not aspirated. The plates were sealed and centrifuged for 1 min at 500 rpm.

The plates were prepared in triplicates with shifted layouts to reduce plate effects and imaged using a Micro XL High-Content Screening System (Molecular Devices) in 5 channels (DAPI: Ex350-400/ Em410-480; FITC: Ex470-500/ Em510-540; Spectrum Gold: Ex520-545/ Em560-585; TxRed: Ex535-585/ Em600-650; Cy5: Ex605-650/ Em670-715) with 9 sites per well and 20x magnification (binning 2).

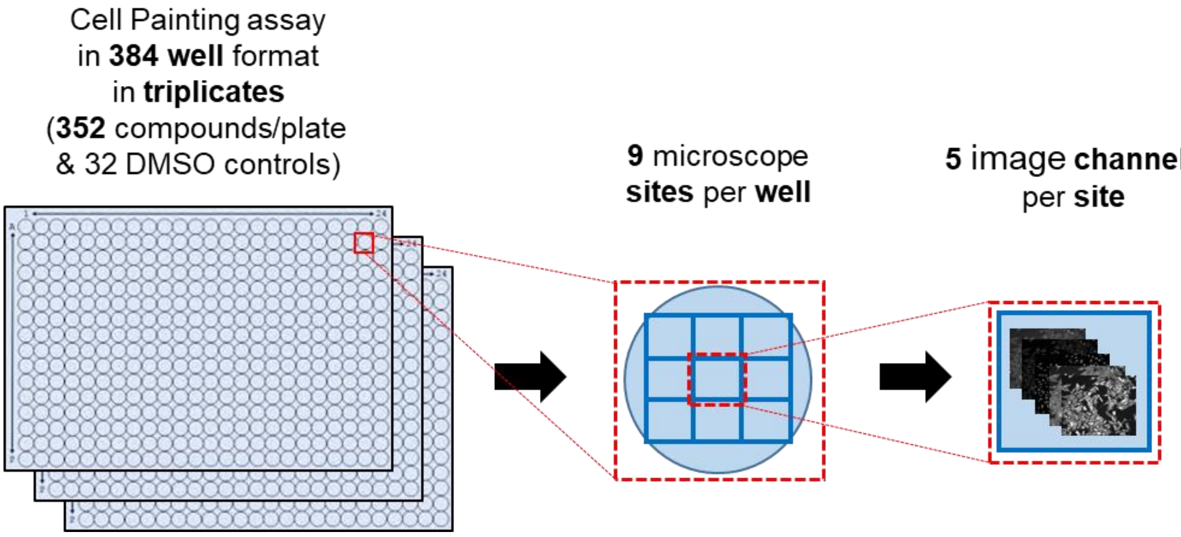

The generated images were processed with the *CellProfiler* package (https://cellprofiler.org/, version 3.0.0) on a computing cluster of the Max Planck Society to extract 1716 cell features (parameters) per microscope site. The data was then further aggregated as medians per well (9 sites -> 1 well), then over the three replicates.

Further analysis was performed with custom *Python* (https://www.python.org/) scripts using the *Pandas* (https://pandas.pydata.org/) and *Dask* (https://dask.org/) data processing libraries as well as the *Scientific Python* (https://scipy.org/) package (separate publication to follow).

From the total set of 1716 parameters a subset of highly reproducible and robust parameters was determined using the procedure described by Woehrmann et al[2]. in the following way: Two biological repeats of one plate containing reference compounds were analysed. For every parameter, its full profile over each whole plate was calculated. If the profiles from the two repeats showed a similarity >= 0.8 (see below), the parameter was added to the set.

This procedure was only performed once and resulted in a set of 579 robust parameters out of the total of 1716 that was used for all further analyses.

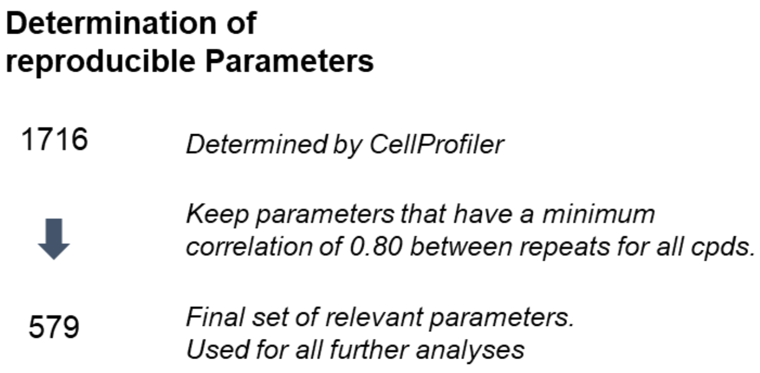

To determine the phenotypic profiles for each test compound Z-scores were then calculated for each parameter as how many times the Median Absolute Deviation (MAD) of the controls the measured parameter value of a test compound deviates from the Median of the controls:

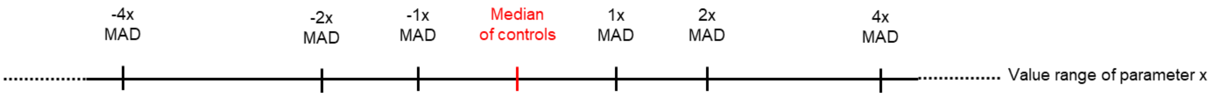

$$z\_score = \frac{value_{meas.} - Median_{Controls}}{MAD_{Controls}}$$

The phenotypic compound profile is then determined as the list of z-scores of all parameters for one compound.

In addition to the phenotypic profile, an induction value was determined for each compound as the fraction of significantly changed parameters, in percent:

$$Induction\ [\%] = \frac{number\ of\ parameters\ with\ abs.\ values > 3}{total\ number\ of\ parameters}$$

Similarities of phenotypic profiles were calculated from the correlation distances between two profiles (https://docs.scipy.org/doc/scipy/reference/generated/scipy.spatial.distance.correlation.html; Similarity = 1 - Correlation Distance) and the compounds with the most similar profiles were determined from a set of 3000 reference compounds that was also measured in the assay.

An example for two compounds with highly similar profiles (96% similarity):

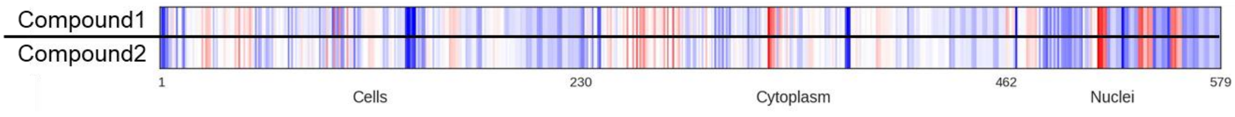

An example for two compounds with low similarity profiles (0% similarity):

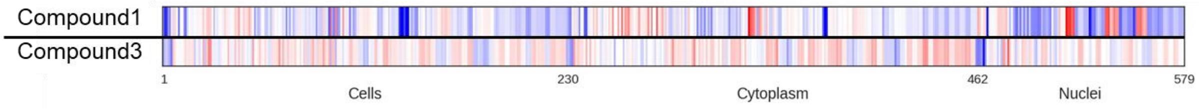

Each colored band represents one Z-score of a parameter.

Principal Component Analyses were performed with the full profiles using the PCA module from the *scikit-learn* package (https://scikit-learn.org/0.22/modules/generated/sklearn.decomposition.PCA.html; v0.22.1, last accessed 22-Apr-2020.)

3. Overview of all molecular structures

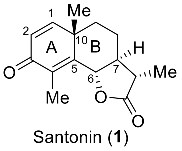

Santonin (**1**)

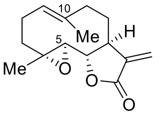

Parthenolide (**2**)

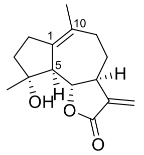

Micheliolide (**3**)

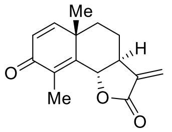

11,13-dehydrosantonin
7

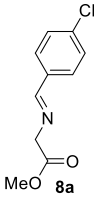

8a

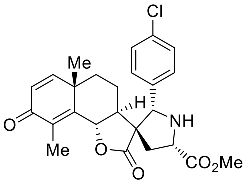

9a

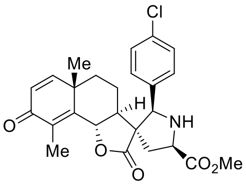

10a

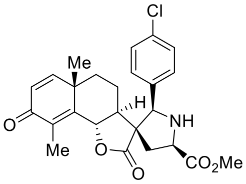

11a

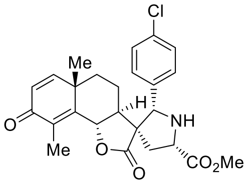

12a

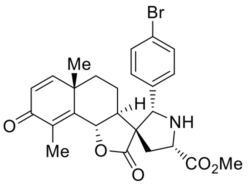

9b

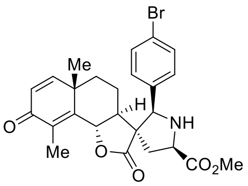

10b

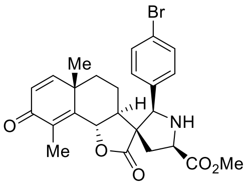

11b

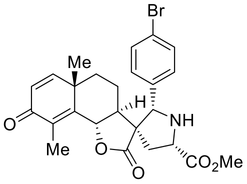

12b

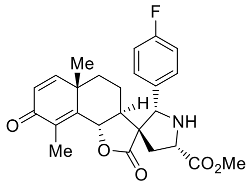

9c

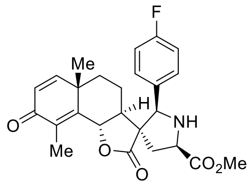

10c

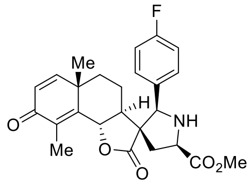

11c

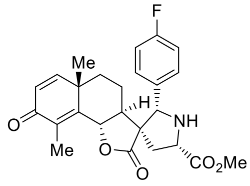

12c

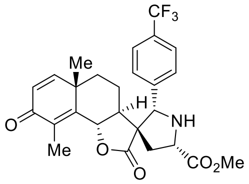

9d

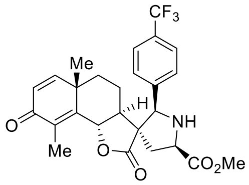

10d

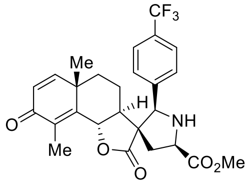

11d

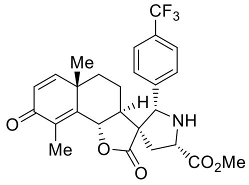

12d

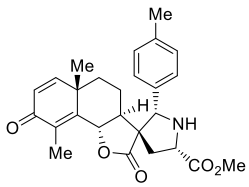

9e

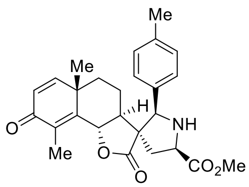

10e

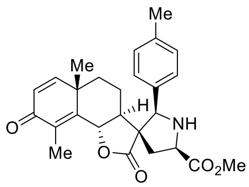

11e

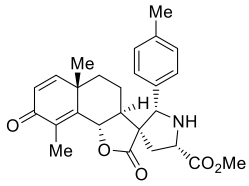

12e

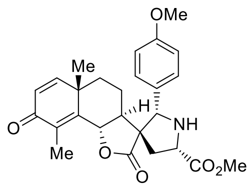

9f

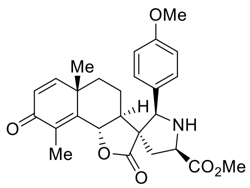

10f

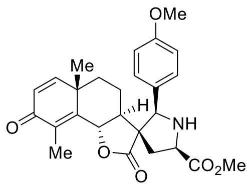

11f

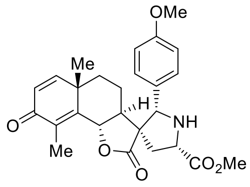

12f

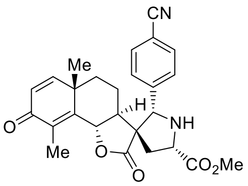

9g

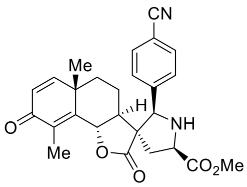

10g

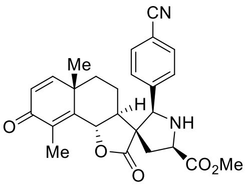

11g

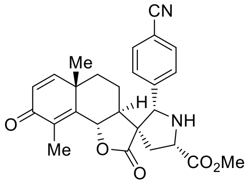

12g

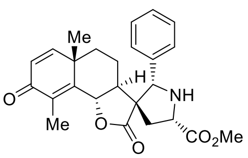

9h

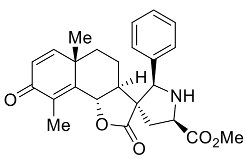

10h

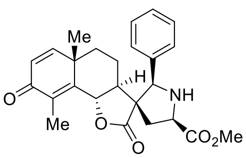

11h

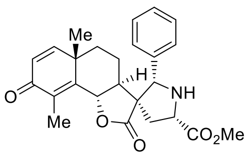

12h

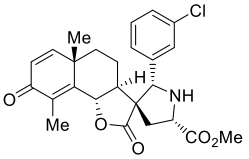

9i

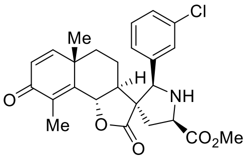

10i

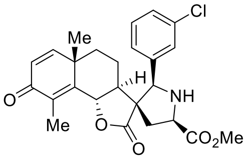

11i

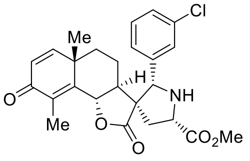

12i

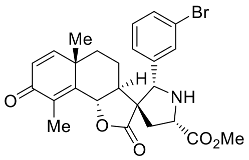

9j

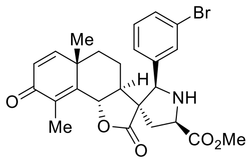

10j

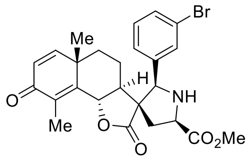

11j

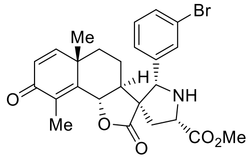

12j

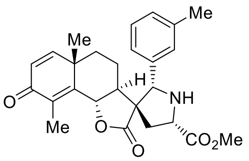

9k

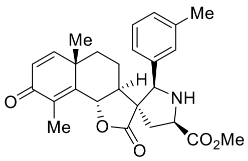

10k

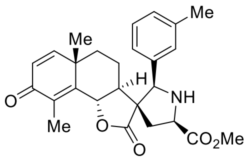

11k

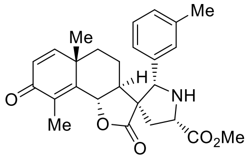

12k

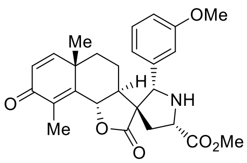

9l

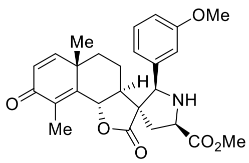

10l

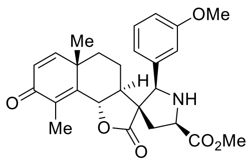

11l

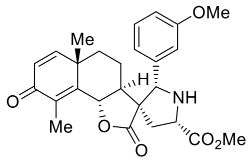

12l

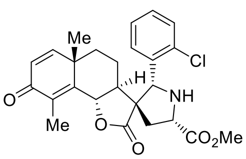

9m

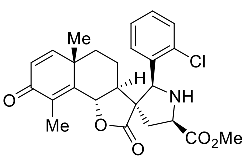

10m

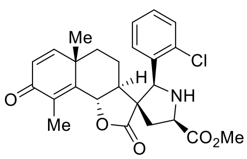

11m (n.r.)

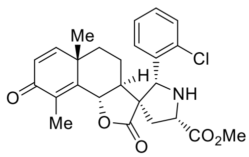

12m

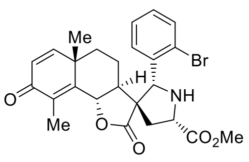
9n

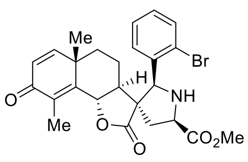
10n

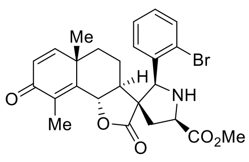
11n (n.r.)

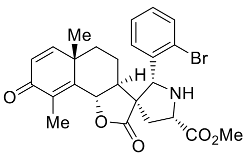
12n

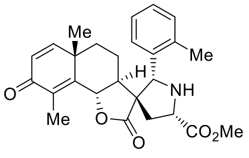
9o

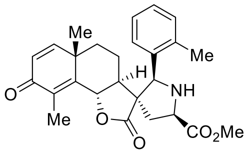
10o

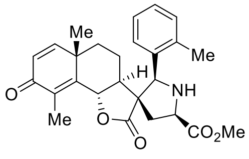
11o (n.r.)

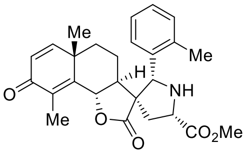
12o

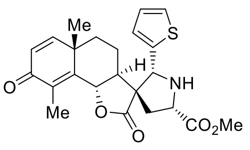
9p

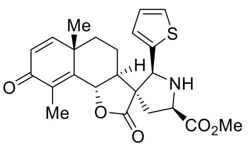
10p

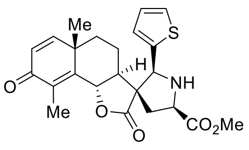
11p

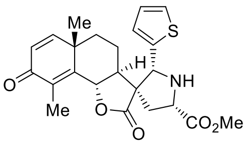
12p

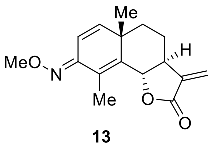
13

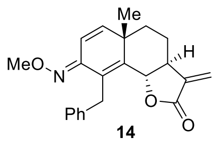
14

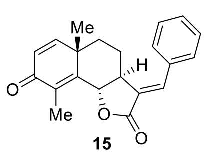
15

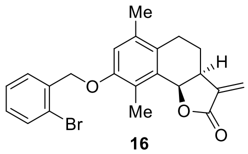
16

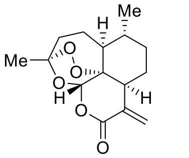
17

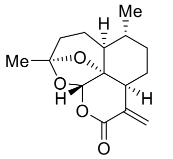
18

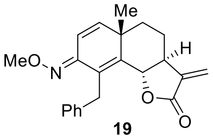
19

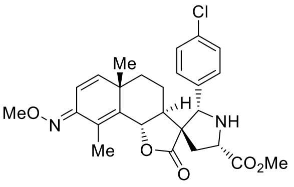
20

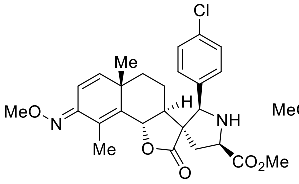
21

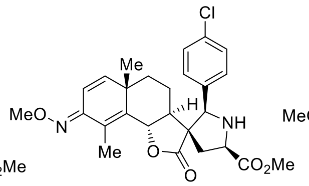
22

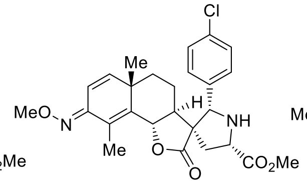
23

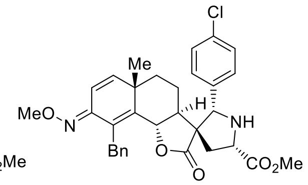
24

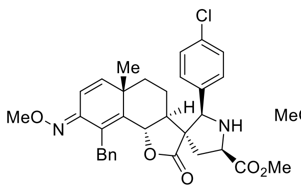
25

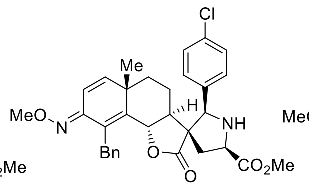
26

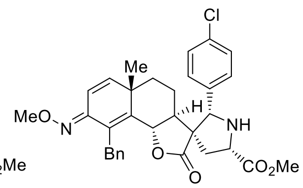
27

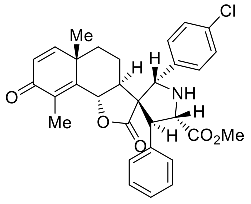
28

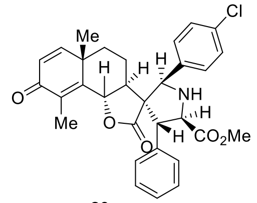

29

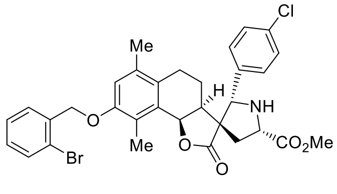

30

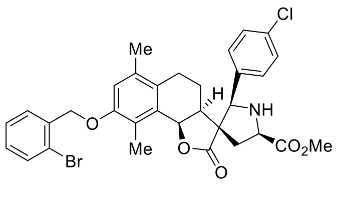

31

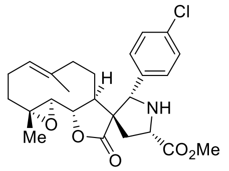

32

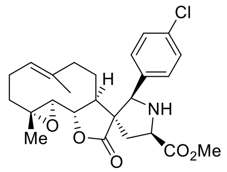

33

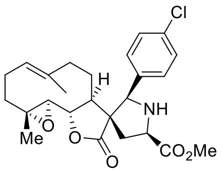

34

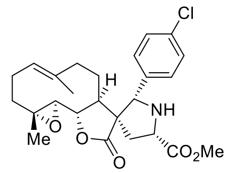

35

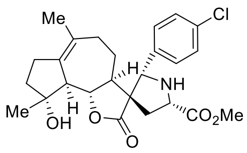

36

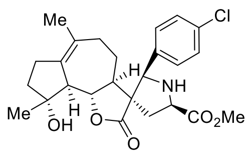

37

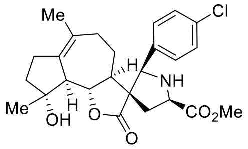

38

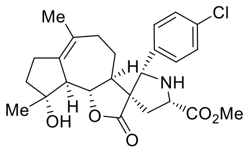

39

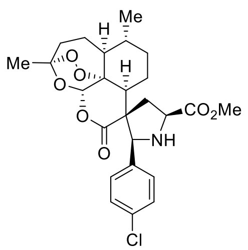

40

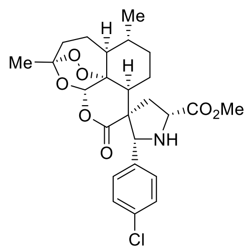

41

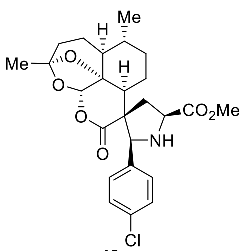

42

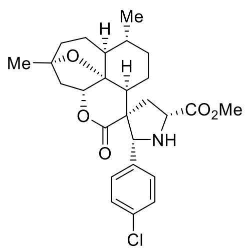

43

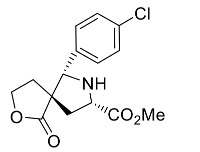

44

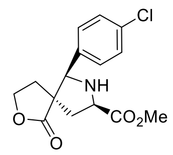

45

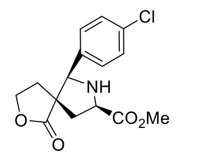

46

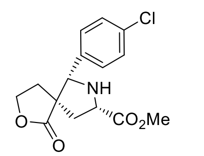

47

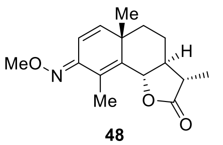

48

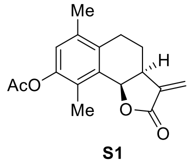

S1

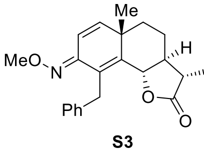

S3

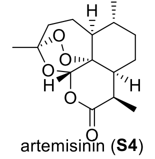

artemisinin (**S4**)

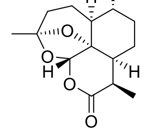

S5

4. General information

Unless otherwise noted, all commercially available compounds were used as provided without further purifications. Solvents for chromatography were technical grade. Analytical thin-layer chromatography (TLC) was performed on Merck silica gel aluminium plates with F-254 indicator. Compounds were visualized by irradiation with UV light or potassium permanganate staining. Column chromatography was performed using silica gel Merck 60 (particle size 0.040-0.063 mm) or aluminum oxide (activated, neutral, Brockmann I, Sigma-Aldrich).

^{1}H-NMR and ^{13}C-NMR were recorded on a *Bruker DRX400* (400 MHz), *Bruker DRX500* (500 MHz), *INOVA500* (500 MHz) and *Bruker DRX700* using CD_2Cl_2, $CDCl_3$ or DMSO-*d6* as solvent. Data are reported in the following order: chemical shift (δ) values are reported in ppm with the solvent resonance as internal standard (CD_2Cl_2: δ = 5.32 ppm for ^{1}H, δ = 53.84 ppm for ^{13}C; $CDCl_3$: δ = 7.26 ppm for ^{1}H, δ = 77.16 ppm for ^{13}C; DMSO-*d6*: δ = 2.50 ppm for ^{1}H, δ = 39.52 ppm for ^{13}C); multiplicities are indicated br s (broadened singlet), s (singlet), d (doublet), t (triplet), q (quartet), m (multiplet); coupling constants (J) are given in Hertz (Hz).

High resolution mass spectra were recorded on a *LTQ Orbitrap* mass spectrometer coupled to an *Accela HPLC*-System (HPLC column: *Hypersyl GOLD*, 50 mm x 1 mm, particle size 1.9 µm, ionization method: electron spray ionization).

Data collection for single crystal X-ray structure analyses was conducted on a *Bruker D8 Venture* four-circle diffractometer by *Bruker AXS GmbH* using a *PHOTON II* CPAD detector by *Bruker AXS GmbH*. X-ray radiation was generated by microfocus sources *IµS 3.0* Mo by *Incoatec GmbH* with HELIOS mirror optics and a single-hole collimator by *Bruker AXS GmbH*.

For the data collection, the programs APEX 3 Suite (v.2018.7-2) with the integrated programs SAINT (integration) and SADABS (adsorption correction) by Bruker AXS GmbH were used. Using Olex2[3], the structures were solved with the ShelXT[4] structure solution program using Intrinsic Phasing and refined with the XL[5] refinement package using Least Squares minimization.

Chemical yields refer to isolated substances. After the cycloaddition, the crude residue was analyzed by ^{1}H NMR to calculate the diastereoselectivity.

5. Experimental details and analytic data for pseudo-sesquiterpenoid alkaloids

5.1 Synthesis of diverse sesquiterpene lactones (SLs) derivatives

Compound **7**, **16**, **48**, **S1**, **S3**, **3**, **17**, **18** were synthesized according to the literature.

General procedure 1 for the synthesis of *α*-methylene-*γ*/*δ*-lactones:

A solution of freshly prepared lithium diisopropylamide (1.2 equiv.; 0.5 M in anhydrous THF) was added dropwise to the sesquiterpene derivatives (1.0 equiv.) in THF (0.1 M) under -78 ^{o}C and Ar protection. The whole reaction was stirred for 1 h before the addition of PhSeCl (1.2 equiv.) dissolved with few THF. When the starting material was full consumed monitored by TLC, aqueous NH_4Cl was added to quench the reaction and warm to room temperature. The solution was extracted with ethyl acetate (EA) for three times. The organic layer was combined, washed with brine, dried with Na_2SO_4 and evaporated after the filtration. The residue was then purified by column chromatography with elution of *n*-pentane and EA.

The *α*-selenation product (1.0 equiv.) was again dissolved in THF (0.1 M). Then AcOH (3.0 equiv.) and H_2O_2 (6.5 equiv.; 30% solution) were added sequentially at 0 ^{o}C. The reaction was stirred for 20 min before warmed to room temperature. When the starting material was full consumed monitored by TLC, aqueous $NaHCO_3$ was added to quench the reaction. The solution was extracted with ethyl acetate (EA) for three times. The organic layer was combined, washed with brine, dried with Na_2SO_4 and evaporated after the filtration. The residue was then purified by column chromatography with elution of *n*-pentane and EA.

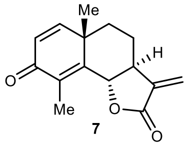

7

(3a*S*,5a*S*,9b*S*)-5a,9-dimethyl-3-methylene-3a,5,5a,9b-tetrahydronaphtho[1,2-*b*]furan-2,8(3*H*,4*H*)-dione

The title product compound **7** was prepared using **General Procedure 1** from santonin (2.46 g, 10 mmol) and isolated by column chromatography (1:1 *n*-pentane: Ethyl acetate) giving a solid (1.1 g, 4.50 mmol, 45% yield). The spectra were identical with the reported data[6].

^{1}H NMR (500 MHz, $CDCl_3$) δ 6.70 (d, *J* = 9.9 Hz, 1H), 6.30 – 6.19 (m, 2H), 5.56 (dd, *J* = 3.1, 1.3 Hz, 1H), 4.77 (dd, *J* = 11.6, 1.4 Hz, 1H), 2.75 – 2.64 (m, 1H), 2.23-2.18 (m, 1H), 2.16 (s, 3H), 1.93 (ddd, *J* = 13.5, 3.9, 2.2 Hz, 1H), 1.84 – 1.72 (m, 1H), 1.59 (td, J = 13.3, 4.5 Hz, 1H), 1.31 (s, 3H).

^{13}C NMR (126 MHz, $CDCl_3$) δ 186.4, 169.3, 154.9, 150.8, 137.6, 129.1, 126.1, 119.9, 81.6, 50.4, 41.5, 37.8, 25.3, 21.8, 11.0.

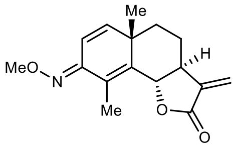

13

(3a*S*,5a*S*,9b*S*,*E*)-8-(methoxyimino)-5a,9-dimethyl-3-methylene-3a,4,5,5a,8,9b-hexahydronaphtho[1,2-*b*]furan-2(3*H*)-one

The title product compound **13** was prepared using **General Procedure 1** from reported compound **48** (300 mg, 1.09 mmol)[7] and isolated by column chromatography (1:1 *n*-pentane: Ethyl acetate) giving a solid (210 mg, 0.77 mmol, 70% yield).

^{1}H NMR (400 MHz, CDCl$_3$) δ 6.80 (d, *J* = 10.2 Hz, 1H), 6.17 (d, *J* = 3.2 Hz, 1H), 5.97 (d, *J* = 10.2 Hz, 1H), 5.49 (d, *J* = 3.1 Hz, 1H), 4.74 (dd, *J* = 11.4, 1.4 Hz, 1H), 3.92 (s, 3H), 2.61-2.70 (m, 1H), 2.25 – 2.06 (m, 4H), 1.82 – 1.66 (m, 2H), 1.59 – 1.48 (m, 1H), 1.21 (s, 3H).

^{13}C NMR (101 MHz, CDCl$_3$) δ 169.7, 149.8, 145.1, 138.3, 123.2, 119.1, 113.0, 82.5, 62.1, 50.3, 40.8, 38.2, 25.9, 22.4, 12.0.

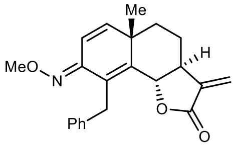

14

(3a*S*,5a*S*,9b*S*,*E*)-9-benzyl-8-(methoxyimino)-5a-methyl-3-methylene-3a,4,5,5a,8,9b-hexahydronaphtho[1,2-*b*]furan-2(3*H*)-one

The title product compound **14** was prepared using **General Procedure 1** from reported compound **S3** (300 mg, 0.85 mmol)[7] and isolated by column chromatography (1:1 *n*-pentane: Ethyl acetate) giving a solid (260 mg, 0.74 mmol, 86% yield).

^{1}H NMR (400 MHz, CDCl$_3$) δ 7.27 – 7.23 (m, 2H), 7.22 – 7.17 (m, 2H), 7.12 – 7.05 (m, 1H), 6.83 (d, *J* = 10.1 Hz, 1H), 6.13 (d, *J* = 3.3 Hz, 1H), 5.99 (d, *J* = 10.2 Hz, 1H), 5.44 (d, *J* = 3.0 Hz, 1H), 4.78 (d, *J* = 11.4 Hz, 1H), 4.28 (d, *J* = 14.7 Hz, 1H), 4.13 (d, *J* = 14.7 Hz, 1H), 3.90 (s, 3H), 2.58 – 2.50 (m, 1H), 2.16 – 2.08 (m, 1H), 1.83 – 1.67 (m, 2H), 1.63-1.55 (m, 1H), 1.27 (s, 3H).

^{13}C NMR (101 MHz, CDCl$_3$) δ 169.4, 148.6, 144.8, 142.2, 139.5, 138.1, 128.6, 128.0, 126.5, 125.3, 119.1, 113.3, 82.1, 62.3, 50.1, 41.1, 38.5, 30.3, 26.1, 22.5.

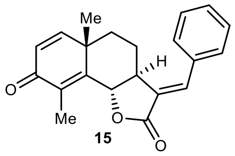

15

(3a*S*,5a*S*,9b*S*)-3-((*E*)-benzylidene)-5a,9-dimethyl-3a,5,5a,9b-tetrahydronaphtho[1,2-*b*]furan-2,8(3*H*,4*H*)-dione

A mixture of dehydrosantonin **7** (150 mg, 0.61 mmol), triethylamine (255 μL, 1.83 mmol), and iodobenzene (77 μL, 0.69 mmol) in DMF (6 mL) was treated with palladium(II) acetate (7 mg, 0.03 mmol) and then heated at 80 °C under air. After 24 h, the reaction mixture was allowed to cool to rt, water (5 mL) was added, and the resultant mixture was extracted with EA (10 mL x 3). The organics were dried over Na_2SO_4 and concentrated under reduced pressure. SiO_2 flash chromatography (2:1 *n*-pentane/EtOAc) afforded the title product **15** as a solid (137 mg, 0.43 mmol, 70%).

^{1}H NMR (400 MHz, CDCl$_3$) δ 7.74 (d, *J* = 3.4 Hz, 1H), 7.42 – 7.35 (m, 3H), 7.36 – 7.29 (m, 2H), 6.67 (d, *J* = 9.9 Hz, 1H), 6.25 (d, *J* = 9.9 Hz, 1H), 4.79 (dq, *J* = 11.3, 1.3 Hz, 1H), 3.03 (tt, *J* = 11.4, 3.2 Hz, 1H), 2.31 – 2.24 (m,

1H), 2.18 (d, J = 1.3 Hz, 3H), 1.79 (ddd, J = 13.2, 3.6, 2.4 Hz, 1H), 1.53 (td, J = 13.2, 3.8 Hz, 1H), 1.47-1.35 (m, 1H), 1.26 (d, J = 0.6 Hz, 3H).

^{13}C NMR (101 MHz, CDCl$_3$) δ 186.3, 170.8, 154.9, 151.2, 138.3, 133.6, 129.6, 129.5, 129.0, 128.5, 128.5, 126.0, 82.0, 49.8, 41.2, 38.2, 25.3, 22.1, 10.9.

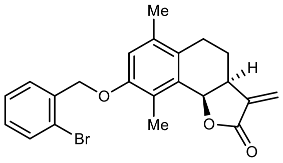

16

(3a*S*,9b*R*)-8-((2-bromobenzyl)oxy)-6,9-dimethyl-3-methylene-3a,4,5,9b-tetrahydronaphtho[1,2-*b*]furan-2(3*H*)-one

The tile compound **16** was synthesized from **7** according to the literature.[6] The spectra were identical with the reported data.

^{1}H NMR (400 MHz, CDCl$_3$) δ 7.64 – 7.56 (m, 2H), 7.36 (td, J = 7.5, 1.3 Hz, 1H), 7.20 (td, J = 7.7, 1.7 Hz, 1H), 6.80 (s, 1H), 6.31 (d, J = 2.1 Hz, 1H), 5.71 (d, J = 1.8 Hz, 1H), 5.63 (d, J = 6.7 Hz, 1H), 5.11 (s, 2H), 3.34-3.29 (m, 1H), 2.72 (ddd, J = 16.4, 6.2, 4.4 Hz, 1H), 2.52 (ddd, J = 16.4, 9.5, 4.4 Hz, 1H), 2.40 (s, 3H), 2.24 (s, 3H), 2.01-1.94 (m, 1H), 1.89 – 1.76 (m, 1H).

^{13}C NMR (101 MHz, CDCl$_3$) δ 170.4, 154.7, 140.2, 136.8, 134.2, 132.7, 130.8, 129.2, 129.0, 128.8, 127.7, 126.2, 122.3, 121.5, 115.1, 75.2, 70.0, 39.6, 26.3, 23.8, 20.1, 11.8.

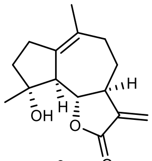

3

(3a*S*,9*R*,9a*S*,9b*S*)-9-hydroxy-6,9-dimethyl-3-methylene-3a,4,5,7,8,9,9a,9b-octahydroazuleno[4,5-*b*]furan-2(3*H*)-one

The tile compound **3** was synthesized from **2** according to the literature. The spectra were identical with the reported data[8].

^{1}H NMR (400 MHz, CDCl$_3$) δ 6.21 (d, J = 3.3 Hz, 1H), 5.50 (d, J = 3.1 Hz, 1H), 3.81 (dd, J = 10.6, 9.9 Hz, 1H), 2.73 (d, J = 10.9 Hz, 1H), 2.71 – 2.61 (m, 1H), 2.47 (s, 1H), 2.39 (dd, J = 16.6, 8.4 Hz, 1H), 2.29 – 2.14 (m, 3H), 2.13-2.06 (m, 1H), 1.88 – 1.73 (m, 2H), 1.69 (s, 3H), 1.40 – 1.32 (m, 1H), 1.30 (s, 3H).

^{13}C NMR (101 MHz, CDCl$_3$) δ 169.9, 139.0, 132.1, 131.0, 119.6, 84.6, 80.4, 58.9, 49.8, 38.5, 35.1, 30.3, 26.0, 24.1, 22.9.

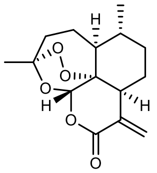

17

(3*R*,5a*S*,6*R*,8a*S*,12*S*,12a*R*)-3,6-dimethyl-9-methyleneoctahydro-12*H*-3,12-epoxy[1,2]dioxepino[4,3-*i*]isochromen-10(3*H*)-one

The title product compound **17** was prepared using **General Procedure 1** from artemisinin **S4** (140 mg, 0.50 mmol) and isolated by column chromatography (5:1 *n*-pentane: Ethyl acetate) giving a solid (68 mg, 0.24 mmol, 48% yield). The spectra were identical with the reported data[9].

^{1}H NMR (400 MHz, $CDCl_3$) δ 6.56 (d, *J* = 1.1 Hz, 1H), 5.99 (s, 1H), 5.66 (d, *J* = 1.1 Hz, 1H), 2.54 (dd, *J* = 13.6, 4.5 Hz, 1H), 2.40 (ddd, *J* = 14.8, 12.8, 3.9 Hz, 1H), 2.06 (ddd, *J* = 15.0, 4.7, 2.9 Hz, 1H), 2.00 – 1.94 (m, 1H), 1.78-1.72 (m, 2H), 1.61 – 1.40 (m, 7H), 1.24 – 1.14 (m, 1H), 1.01 (d, *J* = 5.7 Hz, 3H).

^{13}C NMR (101 MHz, $CDCl_3$) δ 162.8, 135.1, 130.5, 105.5, 93.6, 79.5, 50.3, 46.2, 37.9, 36.0, 33.8, 31.7, 25.5, 24.8, 20.0.

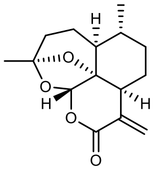

18

(3a*S*,3a1*R*,6*R*,6a*S*,9*S*,10a*R*)-6,9-dimethyl-3-methyleneoctahydro-10a*H*-3a1,9-epoxyoxepino[4,3,2-*ij*]isochromen-2(3*H*)-one

The title product compound **18** was prepared using **General Procedure 1** from reported compound **S5** (133 mg, 0.5 mmol) and isolated by column chromatography (5:1 *n*-pentane: Ethyl acetate) giving a solid (94.1 mg, 0.36 mmol, 72% yield). The spectra were identical with the reported data[9].

^{1}H NMR (500 MHz, $CDCl_3$) δ 6.42 (d, *J* = 1.3 Hz, 1H), 5.79 (s, 1H), 5.67 – 5.60 (m, 1H), 2.80 (dd, *J* = 13.3, 4.4 Hz, 1H), 1.90 (ddd, *J* = 8.5, 4.9, 1.9 Hz, 1H), 1.83 – 1.71 (m, 3H), 1.65 – 1.59 (m, 1H), 1.54 – 1.47 (m, 4H), 1.33 – 1.18 (m, 4H), 0.96 (d, *J* = 6.0 Hz, 3H).

^{13}C NMR (126 MHz, $CDCl_3$) δ 163.2, 135.5, 129.5, 110.1, 99.6, 82.5, 44.8, 44.5, 35.7, 34.0, 33.8, 31.0, 24.2, 22.1, 18.7.

5.2 Conditions for stereodivergent 1,3-dipolar cycloaddition

Condition A: Silver acetate (0.8 mg, 0.005 mmol, 0.05 equiv.) and chiral ligand **L3** (2.5 mg, 0.006 mmol, 0.06 equiv.) were dissolved in dry THF (0.5 mL). After stirring for 30 min, a solution of lactones (0.10 mmol, 1.0 equiv.) and the desired iminoester (0.15 mmol, 1.5 equiv.) in THF (0.5 mL) were added dropwise followed by the addition of Et_3N (2.8 µL, 0.20 mmol, 0.2 equiv.). The reaction was stirred at room temperature until full conversion of the starting material was observed by TLC. The reaction was passed through a short pad of silica and test the crude ^1H NMR to determine the diastereoselectivity. Then the reaction was purified by column chromatography using cyclohexane/EA mixtures. Unless otherwise specified, only main isomer was isolated.

Condition B: Silver acetate (0.8 mg, 0.005 mmol, 0.05 equiv.) and chiral ligand *ent*-**L3** (2.5 mg, 0.006 mmol, 0.06 equiv.) were dissolved in dry THF (0.5 mL). After stirring for 30 min, a solution of lactones (0.10 mmol, 1.0 equiv.) and the desired iminoester (0.15 mmol, 1.5 equiv.) in THF (0.5 mL) were added at 0 ^oC dropwise followed by the addition of Et_3N (2.8 µL, 0.20 mmol, 0.2 equiv.). The reaction was stirred until full conversion of the starting material was observed by TLC. The reaction was passed through a short pad of silica and test the crude ^1H NMR to determine the diastereoselectivity. Then the reaction was purified by column chromatography using cyclohexane/EA mixtures. Unless otherwise specified, only main isomer was isolated.

Condition C: Silver acetate (0.8 mg, 0.005 mmol, 0.05 equiv.) and chiral ligand **L4** (7.1 mg, 0.006 mmol, 0.06 equiv.) were dissolved in dry DCE (0.5 mL). After stirring for 30 min, a solution of lactones (0.10 mmol, 1.0 equiv.) and the desired iminoester (0.15 mmol, 1.5 equiv.) in DCE (0.5 mL) were added dropwise followed by the addition of Et_3N (2.8 µL, 0.20 mmol, 0.2 equiv.). The reaction was stirred until full conversion of the starting material was observed by TLC. The reaction was passed through a short pad of silica and test the crude ^1H NMR to determine the diastereoselectivity. Then the reaction was purified by column chromatography using cyclohexane/EA mixtures. Unless otherwise specified, only main isomer was isolated.

Condition D: Silver acetate (0.8 mg, 0.005 mmol, 0.05 equiv.) and chiral ligand **L5** (6.9 mg, 0.006 mmol, 0.06 equiv.) were dissolved in $CHCl_3$ (0.5 mL). After stirring for 30 min, a solution of lactones (0.10 mmol, 1.0 equiv.) and the desired iminoester (0.20 mmol, 2.0 equiv.) in $CHCl_3$ (0.5 mL) were added dropwise at 0 ^oC followed by the addition of Cs_2CO_3 (16.3 mg, 0.05 mmol, 0.5 equiv.). The reaction was stirred until full conversion of the starting material was observed by TLC. The reaction was passed through a short pad of silica and test the crude ^1H NMR to determine the diastereoselectivity. Then the reaction was purified by column chromatography using cyclohexane/EA mixtures. Unless otherwise specified, only main isomer was isolated.

5.3 Analytic data for pseudo sesquiterpenoid alkaloids

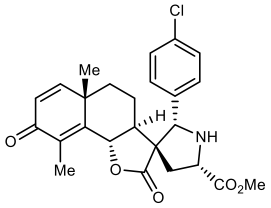

9a

Methyl (2'*S*,3*R*,3a*S*,5a*S*,5'*S*,9b*S*)-2'-(4-chlorophenyl)-5a,9-dimethyl-2,8-dioxo-3a,4,5,5a,8,9b-hexahydro-2*H*-spiro[naphtho[1,2-*b*]furan-3,3'-pyrrolidine]-5'-carboxylate

The title product compound **9a** was prepared in 0.10 mmol scale using **Condition A** and isolated by column chromatography (20:1 DCM: MeOH) giving an amorphous solid (35 mg, 0.07 mmol, 73% yield).

^{1}H NMR (500 MHz, $CDCl_3$) δ 7.34 – 7.28 (m, 4H), 6.67 (d, *J* = 9.9 Hz, 1H), 6.23 (d, *J* = 9.9 Hz, 1H), 4.78 (dd, *J* = 11.7, 1.5 Hz, 1H), 4.09 – 3.99 (m, 2H), 3.82 (s, 3H), 2.54 (dd, *J* = 13.5, 9.0 Hz, 1H), 2.46 (dd, *J* = 13.4, 5.4 Hz, 1H), 2.05 – 1.93 (m, 3H), 1.90 (d, *J* = 1.4 Hz, 3H), 1.88 – 1.79 (m, 1H), 1.53 (td, *J* = 13.1, 4.6 Hz, 1H), 1.30 (s, 3H).

^{13}C NMR (126 MHz, $CDCl_3$) δ 186.2, 175.9, 172.5, 154.5, 150.4, 134.5, 134.0, 129.7, 129.3, 128.9, 126.2, 80.0, 68.0, 58.7, 55.1, 52.8, 50.3, 41.1, 37.6, 33.8, 25.2, 19.4, 10.9.

HRMS(ESI): $[M+H]^+$ calcd. $C_{25}H_{27}NO_5Cl$ m/z 456.1572, found 456.1560.

IR (film, cm^{-1}): 2926, 2854, 1775, 1737, 1661, 1634, 1615, 1491, 1436, 1376, 1268, 1225, 1204, 1185, 1154, 1091, 1036, 1014, 991, 961, 906, 831.

$[\alpha]_D^{20}$ = -52.3 (c = 0.17, $CHCl_3$)

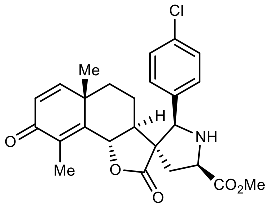

10a

Methyl (2'*R*,3*S*,3a*S*,5a*S*,5'*R*,9b*S*)-2'-(4-chlorophenyl)-5a,9-dimethyl-2,8-dioxo-3a,4,5,5a,8,9b-hexahydro-2*H*-spiro[naphtho[1,2-*b*]furan-3,3'-pyrrolidine]-5'-carboxylate

The title product compound **10a** was prepared in 0.10 mmol scale using **Condition B** and isolated by column chromatography (20:1 DCM: MeOH) giving an amorphous solid (43.3 mg, 0.09 mmol, 95% yield).

^{1}H NMR (500 MHz, $CDCl_3$) δ 7.42 (d, *J* = 8.3 Hz, 2H), 7.36 (d, *J* = 8.2 Hz, 2H), 6.62 (d, *J* = 9.9 Hz, 1H), 6.18 (d, *J* = 9.8 Hz, 1H), 4.39 (s, 1H), 4.21 (dd, *J* = 11.9, 1.5 Hz, 1H), 4.01-3.97 (m, 1H), 3.83 (s, 3H), 2.65 (dd, *J* = 13.6, 4.7 Hz, 1H), 2.32 (dd, *J* = 13.7, 9.5 Hz, 1H), 2.23 (td, *J* = 12.1, 3.5 Hz, 1H), 2.11 – 1.85 (m, 6H), 1.49 – 1.40 (m, 1H), 1.08 (s, 3H).

^{13}C NMR (126 MHz, $CDCl_3$) δ 186.2, 177.3, 173.4, 154.5, 150.6, 136.3, 135.1, 129.7, 129.2, 128.9, 126.1, 79.5, 65.8, 58.3, 56.7, 52.7, 52.5, 41.1, 38.6, 37.8, 24.8, 19.2, 11.0.

HRMS(ESI): $[M+H]^+$ calcd. $C_{25}H_{27}NO_5Cl$ m/z 456.1572, found 456.1562.

IR (film, cm^{-1}): 2959, 1774, 1732,1662, 1635, 1615, 1492.99, 1435, 1372, 1336, 1315, 1287, 1263,1219, 1195, 1169, 1149, 1094, 1064, 1041, 1013, 997, 962, 939, 919, 894, 839, 827.

$[\alpha]_D^{20}$ = -122.1 (c = 0.15, $CHCl_3$)

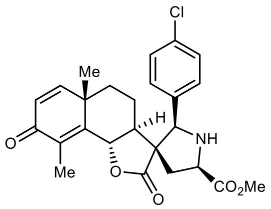

11a

Methyl (2'*R*,3*R*,3a*S*,5a*S*,5'*R*,9b*S*)-2'-(4-chlorophenyl)-5a,9-dimethyl-2,8-dioxo-3a,4,5,5a,8,9b-hexahydro-2*H*-spiro[naphtho[1,2-*b*]furan-3,3'-pyrrolidine]-5'-carboxylate

The title product compound **11a** was prepared in 0.10 mmol scale using **Condition C** and isolated by column chromatography (1:1 Cyclohexane: Ethyl acetate) giving an amorphous solid (40.8 mg, 0.09 mmol, 89% yield).

^{1}H NMR (700 MHz, $CDCl_3$) δ 7.34 – 7.26 (m, 4H), 6.57 (d, *J* = 9.9 Hz, 1H), 6.18 (d, *J* = 9.9 Hz, 1H), 4.82 – 4.70 (m, 2H), 4.21 (dd, *J* = 9.0, 7.7 Hz, 1H), 3.82 (s, 3H), 2.51 (dd, *J* = 13.4, 7.7 Hz, 1H), 2.37 – 2.27 (m, 1H), 2.04 (d, *J* = 1.5 Hz, 3H), 1.66 (ddd, *J* = 13.4, 3.7, 2.4 Hz, 1H), 1.63 – 1.49 (m, 2H), 1.31 – 1.27 (m, 2H), 1.22 (s, 3H), 1.10 (td, *J* = 12.9, 4.5 Hz, 1H).

^{13}C NMR (176 MHz, $CDCl_3$) δ 186.2, 178.3, 173.3, 154.8, 150.7, 137.4, 134.1, 129.3, 129.0, 128.5, 126.1, 79.9, 66.5, 57.1, 55.1, 52.5, 50.3, 41.1, 37.4, 31.2, 25.1, 20.1, 11.1.

HRMS(ESI): $[M+H]^+$ calcd. $C_{25}H_{27}NO_5Cl$ m/z 456.1572, found 456.1568.

IR (film, cm^{-1}): 2925, 1771, 1737, 1662, 1634, 1614, 1490 ,1454, 1437, 1376, 1274, 1192, 1153, 1139, 1090, 1042, 1014, 998, 968, 902, 832.

$[\alpha]_D^{20}$ = +84.0 (c = 0.15, $CHCl_3$)

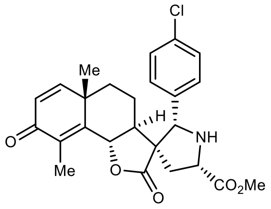

12a

Methyl (2'*S*,3*S*,3a*S*,5a*S*,5'*S*,9b*S*)-2'-(4-chlorophenyl)-5a,9-dimethyl-2,8-dioxo-3a,4,5,5a,8,9b-hexahydro-2*H*-spiro[naphtho[1,2-*b*]furan-3,3'-pyrrolidine]-5'-carboxylate

The title product compound **12a** was prepared in 0.10 mmol scale using **Condition D** and isolated by column chromatography (1:1 Cyclohexane: Ethyl acetate) giving an amorphous solid (40.0 mg, 0.09 mmol, 88% yield).

^{1}H NMR (600 MHz, CDCl$_3$) δ 7.52 (d, *J* = 7.9 Hz, 2H), 7.41 – 7.34 (m, 2H), 6.52 (d, *J* = 9.9 Hz, 1H), 6.15 (d, *J* = 9.9 Hz, 1H), 4.53 (s, 1H), 4.17 (ddd, *J* = 11.9, 10.6, 3.7 Hz, 2H), 3.80 (s, 3H), 3.01 (dd, *J* = 13.9, 10.7 Hz, 1H), 2.19 (dd, *J* = 13.9, 5.6 Hz, 1H), 2.05 (d, *J* = 1.4 Hz, 3H), 1.99 – 1.95 (m, 2H), 1.91 – 1.82 (m, 1H), 1.66 (ddd, *J* = 13.7, 4.1, 2.3 Hz, 1H), 1.20 (td, *J* = 13.1, 4.4 Hz, 1H), 0.65 (s, 3H).

^{13}C NMR (151 MHz, CDCl$_3$) δ 186.3, 178.5, 173.3, 154.9, 151.1, 135.1, 134.6, 129.1, 128.7, 128.6, 125.9, 79.7, 67.8, 56.8, 56.6, 54.9, 52.6, 41.2, 40.4, 38.5, 24.0, 18.2, 11.1.

HRMS(ESI): [M+H]$^+$ calcd. $C_{25}H_{27}NO_5Cl$ m/z 456.1572, found 456.1558.

IR (film, cm^{-1}): 2930, 1770, 1737, 1661, 1633, 16141491, 1437, 1406, 1377, 1271, 1201, 1186, 1162, 1091, 1040, 1013, 989, 954, 903, 878, 830.

[α]$_D^{20}$ = -100.0 (c = 0.10, CHCl$_3$)

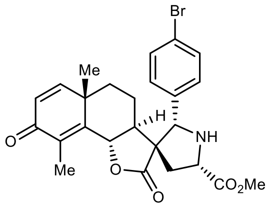

9b

Methyl (2'*S*,3*R*,3a*S*,5a*S*,5'*S*,9b*S*)-2'-(4-bromophenyl)-5a,9-dimethyl-2,8-dioxo-3a,4,5,5a,8,9b-hexahydro-2*H*-spiro[naphtho[1,2-*b*]furan-3,3'-pyrrolidine]-5'-carboxylate

The title product compound **9b** was prepared in 0.10 mmol scale using **Condition A** and isolated by column chromatography (20:1 DCM: MeOH) giving an amorphous solid (33.4 mg, 0.07 mmol, 67% yield).

^{1}H NMR (500 MHz, CDCl$_3$) δ 7.49 – 7.41 (m, 2H), 7.32 – 7.17 (m, 2H), 6.67 (d, *J* = 9.9 Hz, 1H), 6.23 (d, *J* = 9.8 Hz, 1H), 4.77 (dd, *J* = 11.6, 1.4 Hz, 1H), 4.04 (m, 2H), 3.81 (s, 3H), 2.54 (dd, *J* = 13.5, 9.0 Hz, 1H), 2.45 (dd, *J* = 13.4, 5.4 Hz, 1H), 2.03 – 1.92 (m, 3H), 1.89 (d, *J* = 1.4 Hz, 3H), 1.88-1.78 (m, 1H), 1.53 (td, *J* = 13.1, 4.6 Hz, 1H), 1.29 (s, 3H).

^{13}C NMR (126 MHz, CDCl$_3$) δ 186.2, 175.9, 172.5, 154.5, 150.4, 134.6, 131.8, 130.0, 129.3, 126.2, 122.7, 80.0, 67.9, 58.7, 55.0, 52.7, 50.3, 41.1, 37.5, 33.8, 25.2, 19.4, 10.9.

HRMS(ESI): [M+H]$^+$ calcd. $C_{25}H_{27}NO_5Br$ m/z 500.1067, found 500.1055.

IR (film, cm^{-1}): 2946, 2557, 2161, 2036, 1774, 1736, 1661, 1633, 1614, 1487, 1436, 1376, 1305, 1270, 1224, 1204, 1185, 1154, 1132, 1057, 1035, 1010, 991, 961, 905, 831.

[α]$_D^{20}$ = -91.4 (c = 0.11, CHCl$_3$)

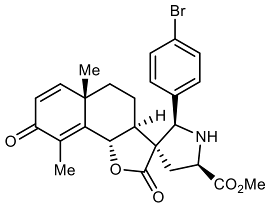

10b

Methyl (2'*R*,3*S*,3a*S*,5a*S*,5'*R*,9b*S*)-2'-(4-bromophenyl)-5a,9-dimethyl-2,8-dioxo-3a,4,5,5a,8,9b-hexahydro-2*H*-spiro[naphtho[1,2-*b*]furan-3,3'-pyrrolidine]-5'-carboxylate

The title product compound **10b** was prepared in 0.10 mmol scale using **Condition B** and isolated by column chromatography (20:1 DCM: MeOH) giving an amorphous solid (42.6 mg, 0.09 mmol, 85% yield).

^{1}H NMR (500 MHz, $CDCl_3$) δ 7.53 (d, *J* = 8.5 Hz, 2H), 7.36 (d, *J* = 8.5 Hz, 2H), 6.62 (d, *J* = 9.9 Hz, 1H), 6.20 (d, *J* = 9.9 Hz, 1H), 4.37 (s, 1H), 4.24 (dd, *J* = 11.9, 1.5 Hz, 1H), 3.99 (dd, *J* = 9.6, 5.2 Hz, 1H), 3.84 (s, 3H), 2.66 (dd, *J* = 13.6, 5.2 Hz, 1H), 2.32 (dd, *J* = 13.6, 9.6 Hz, 1H), 2.24 (td, *J* = 12.1, 4.0 Hz, 1H), 2.08 – 1.88 (m, 6H), 1.46 (td, *J* = 13.0, 4.9 Hz, 1H), 1.09 (s, 3H).

^{13}C NMR (126 MHz, $CDCl_3$) δ 186.2, 177.3, 173.4, 154.5, 150.6, 137.0, 132.6, 129.5, 129.0, 126.2, 123.3, 79.5, 77.5, 77.2, 76.8, 66.0, 58.5, 56.7, 52.8, 52.6, 41.2, 38.7, 37.9, 24.9, 19.2, 11.0.

HRMS(ESI): $[M+H]^+$ calcd. $C_{25}H_{27}NO_5Br$ m/z 500.1067, found 500.1053.

IR (film, cm^{-1}): 2960, 1773, 1732, 1661, 1635, 1615, 1490, 1435, 1372, 1336, 1314, 1292, 1263, 1218, 1194, 1168, 1149, 1115, 1094, 1064, 1041, 1009, 997, 962, 939, 919, 895, 840, 827, 806.

$[\alpha]_D^{20}$ = -129.3 (c = 0.14, $CHCl_3$)

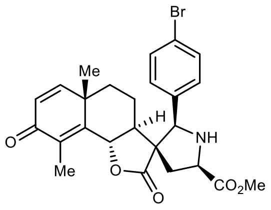

11b

Methyl (2'*R*,3*R*,3a*S*,5a*S*,5'*R*,9b*S*)-2'-(4-bromophenyl)-5a,9-dimethyl-2,8-dioxo-3a,4,5,5a,8,9b-hexahydro-2*H*-spiro[naphtho[1,2-*b*]furan-3,3'-pyrrolidine]-5'-carboxylate

The title product compound **11b** was prepared in 0.10 mmol scale using **Condition C** and isolated by column chromatography (1:1 Cyclohexane: Ethyl acetate) giving an amorphous solid (47.0 mg, 0.09 mmol, 94% yield).

^{1}H NMR (500 MHz, $CDCl_3$) δ 7.43 (d, *J* = 8.5 Hz, 2H), 7.26 (d, *J* = 8.5 Hz, 2H), 6.57 (d, *J* = 9.8 Hz, 1H), 6.19 (d, *J* = 9.8 Hz, 1H), 4.77 (dd, *J* = 11.3, 1.5 Hz, 1H), 4.73 (s, 1H), 4.21 (dd, *J* = 9.0, 7.6 Hz, 1H), 3.82 (s, 3H), 2.51 (dd, *J* = 13.4, 7.7 Hz, 1H), 2.32 (dd, *J* = 13.4, 9.0 Hz, 1H), 2.04 (d, *J* = 1.4 Hz, 3H), 1.67 (ddd, *J* = 13.5, 3.7, 2.3 Hz, 1H), 1.62 – 1.49 (m, 2H), 1.33 – 1.27 (m, 1H), 1.22 (s, 3H), 1.15 – 1.07 (m, 1H).

^{13}C NMR (126 MHz, $CDCl_3$) δ 186.2, 173.2, 154.8, 154.8, 150.7, 137.9, 131.9, 129.2, 128.8, 126.0, 122.2, 79.9, 77.4, 77.2, 76.9, 66.5, 57.1, 55.0, 52.6, 50.2, 41.1, 37.3, 31.2, 25.1, 20.1, 11.1.

HRMS(ESI): $[M+H]^+$ calcd. $C_{25}H_{27}NO_5Br$ m/z 500.1067, found 500.1061.

IR (film, cm^{-1}): 2948, 2160, 2032, 1772, 1737, 1662, 1635, 1487, 1437, 1274, 1193, 1042, 1010, 902, 832.

$[\alpha]_D^{20}$ = +75.3 (c = 0.09, $CHCl_3$)

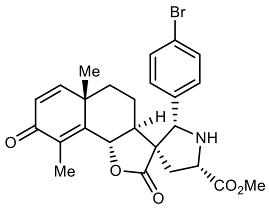

12b

Methyl (2'*S*,3*S*,3a*S*,5a*S*,5'*S*,9b*S*)-2'-(4-bromophenyl)-5a,9-dimethyl-2,8-dioxo-3a,4,5,5a,8,9b-hexahydro-2*H*-spiro[naphtho[1,2-*b*]furan-3,3'-pyrrolidine]-5'-carboxylate

The title product compound **12b** was prepared in 0.10 mmol scale using **Condition D** and isolated by column chromatography (1:1 Cyclohexane: Ethyl acetate) giving an amorphous solid (43.8 mg, 0.09 mmol, 88% yield).

^{1}H NMR (600 MHz, CDCl$_3$) δ 7.57 – 7.52 (m, 2H), 7.45 (d, *J* = 8.1 Hz, 2H), 6.52 (d, *J* = 9.9 Hz, 1H), 6.14 (d, *J* = 9.9 Hz, 1H), 4.50 (s, 1H), 4.20 – 4.11 (m, 2H), 3.79 (s, 3H), 3.00 (dd, *J* = 13.9, 10.7 Hz, 1H), 2.18 (dd, *J* = 13.9, 5.5 Hz, 1H), 2.05 (d, *J* = 1.4 Hz, 3H), 2.00 – 1.93 (m, 2H), 1.90 – 1.83 (m, 1H), 1.66 (ddd, *J* = 13.6, 4.1, 2.3 Hz, 1H), 1.19 (td, *J* = 13.0, 4.4 Hz, 1H), 0.65 (s, 3H).

^{13}C NMR (151 MHz, CDCl$_3$) δ 186.3, 178.5, 173.3, 155.0, 151.1, 135.8, 132.1, 129.0, 128.5, 125.9, 122.5, 79.7, 77.4, 77.2, 76.9, 67.9, 56.8, 56.5, 54.8, 52.6, 41.2, 40.5, 38.4, 23.9, 18.2, 11.1.

HRMS(ESI): $[M+H]^+$ calcd. $C_{25}H_{27}NO_5Br$ m/z 500.1067, found 500.1062.

IR (film, cm^{-1}): 1766, 1740, 1658, 1628, 1432, 1272, 1203, 1009, 844, 821.

$[\alpha]_D^{20}$ = -97.6 (c = 0.21, $CHCl_3$)

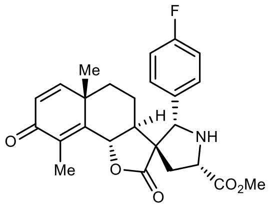

9c

Methyl (2'*S*,3*R*,3a*S*,5a*S*,5'*S*,9b*S*)-2'-(4-fluorophenyl)-5a,9-dimethyl-2,8-dioxo-3a,4,5,5a,8,9b-hexahydro-2*H*-spiro[naphtho[1,2-*b*]furan-3,3'-pyrrolidine]-5'-carboxylate

The title product compound **9c** was prepared in 0.10 mmol scale using **Condition A** and isolated by column chromatography (20:1 DCM: MeOH) giving an amorphous solid (23.3 mg, 0.05 mmol, 53% yield).

^{1}H NMR (500 MHz, CDCl$_3$) δ 7.43 – 7.31 (m, 2H), 7.06-6.98 (m, 2H), 6.67 (d, *J* = 9.9 Hz, 1H), 6.22 (d, *J* = 9.9 Hz, 1H), 4.78 (dd, *J* = 11.7, 1.5 Hz, 1H), 4.18 – 4.05 (m, 2H), 3.81 (s, 3H), 2.56 (dd, *J* = 13.5, 9.1 Hz, 1H), 2.48 (dd, *J* = 13.5, 5.0 Hz, 1H), 2.05 – 1.94 (m, 3H), 1.88 (d, *J* = 1.4 Hz, 3H), 1.83 (dd, *J* = 12.7, 4.0 Hz, 1H), 1.52 (ddd, *J* = 13.6, 13.1, 4.4 Hz, 1H), 1.29 (s, 3H).

^{13}C NMR (126 MHz, CDCl$_3$) δ 186.2, 176.1, 172.4, 162.9 (d, *J* = 247.8 Hz), 154.6, 150.4, 130.8 (d, *J* = 3.3 Hz), 130.1 (d, *J* = 8.2 Hz), 129.2, 126.2, 115.7 (d, *J* = 21.4 Hz), 80.1, 67.8, 58.6, 55.0, 52.8, 50.1, 41.2, 37.6, 33.7, 25.1, 19.5, 10.8.

HRMS(ESI): $[M+H]^+$ calcd. $C_{25}H_{27}NO_5F$ m/z 440.1868, found 440.1856.

IR (film, cm^{-1}): 3349, 2938, 2346, 1784, 1733, 1657, 1626, 1609, 1509, 1438, 1404, 1371, 1338, 1292, 1266, 1223, 1187, 1164, 1155, 1135, 1084, 1056, 1024, 993, 957, 901, 837.

$[\alpha]_D^{20}$ = -64.6 (c = 0.13, $CHCl_3$)

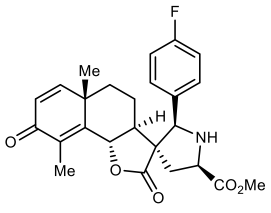

10c

Methyl (2'*R*,3*S*,3a*S*,5a*S*,5'*R*,9b*S*)-2'-(4-fluorophenyl)-5a,9-dimethyl-2,8-dioxo-3a,4,5,5a,8,9b-hexahydro-2*H*-spiro[naphtho[1,2-*b*]furan-3,3'-pyrrolidine]-5'-carboxylate

The title product compound **10c** was prepared in 0.10 mmol scale using **Condition B** and isolated by column chromatography (20:1 DCM: MeOH) giving an amorphous solid (43.7 mg, 0.10 mmol, 99% yield).

^{1}H NMR (500 MHz, $CDCl_3$) δ 7.53 – 7.40 (m, 2H), 7.12-7.05 (m, 2H), 6.61 (d, *J* = 9.9 Hz, 1H), 6.18 (d, *J* = 9.8 Hz, 1H), 4.38 (s, 1H), 4.18 (dd, *J* = 12.0, 1.4 Hz, 1H), 3.99 (dd, *J* = 9.7, 5.0 Hz, 1H), 3.83 (s, 3H), 2.64 (dd, *J* = 13.7, 5.1 Hz, 1H), 2.32 (dd, *J* = 13.7, 9.8 Hz, 1H), 2.24 (td, *J* = 12.3, 3.7 Hz, 1H), 2.09 – 2.03 (m, 1H), 1.99 – 1.84 (m, 5H), 1.44 (td, *J* = 13.2, 4.7 Hz, 1H), 1.06 (s, 3H).

^{13}C NMR (126 MHz, $CDCl_3$) δ 186.2, 177.5, 173.4, 163.0 (d, *J* = 248.8 Hz), 154.6, 150.7, 133.6 (d, *J* = 3.2 Hz), 129.6 (d, *J* = 8.2 Hz), 128.9, 126.1, 116.5 (d, *J* = 21.4 Hz), 79.4, 65.9, 58.3, 56.6, 52.7, 52.4, 41.1, 38.6, 37.8, 24.8, 19.1, 11.0.

HRMS(ESI): $[M+H]^+$ calcd. $C_{25}H_{27}NO_5F$ m/z 440.1868, found 440.1855.

IR (film, cm^{-1}): 2951, 2161, 1759, 1734, 1661, 1634, 1614, 1510, 1437, 1406, 1374, 1335, 1307, 1265, 1217, 1155, 1132, 1107, 1061, 1038, 991, 944, 918, 904, 836.

$[\alpha]_D^{20}$ = -99.2 (c = 0.13, $CHCl_3$)

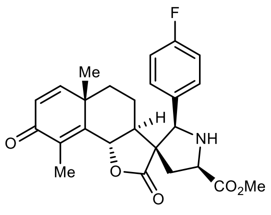

11c

Methyl (2'*R*,3*R*,3a*S*,5a*S*,5'*R*,9b*S*)-2'-(4-fluorophenyl)-5a,9-dimethyl-2,8-dioxo-3a,4,5,5a,8,9b-hexahydro-2*H*-spiro[naphtho[1,2-*b*]furan-3,3'-pyrrolidine]-5'-carboxylate

The title product compound **11c** was prepared in 0.10 mmol scale using **Condition C** and isolated by column chromatography (1:1 Cyclohexane: Ethyl acetate) giving an amorphous solid (35.4 mg, 0.08 mmol, 81% yield).

^{1}H NMR (700 MHz, CDCl$_3$) δ 7.41 – 7.33 (m, 2H), 7.03-6.98 (m, 2H), 6.57 (d, *J* = 9.9 Hz, 1H), 6.19 (d, *J* = 9.8 Hz, 1H), 4.80 – 4.74 (m, 2H), 4.22 (dd, *J* = 8.9, 7.7 Hz, 1H), 3.82 (s, 3H), 2.51 (dd, *J* = 13.4, 7.8 Hz, 1H), 2.32 (dd, *J* = 13.4, 8.9 Hz, 1H), 2.04 (d, *J* = 1.4 Hz, 3H), 1.66 (ddd, *J* = 13.4, 3.8, 2.4 Hz, 1H), 1.60 (dd, *J* = 12.3, 3.1 Hz, 1H), 1.54 (dd, *J* = 12.7, 3.9 Hz, 1H), 1.28 – 1.24 (m, 1H), 1.23 (s, 3H), 1.09 (td, *J* = 13.0, 4.5 Hz, 1H).

^{13}C NMR (176 MHz, CDCl$_3$) δ 186.3, 178.4, 173.5, 162.6 (d, *J* = 247.0 Hz), 154.8, 150.8, 134.7 (d, *J* = 3.1 Hz), 129.2, 128.7 (d, *J* = 8.0 Hz), 126.1, 115.7 (d, *J* = 21.3 Hz), 80.0, 66.5, 57.1, 55.1, 52.6, 50.3, 41.1, 37.5, 31.2, 25.1, 20.2, 11.1.

HRMS(ESI): $[M+H]^+$ calcd. $C_{25}H_{27}NO_5F$ m/z 440.1868, found 440.1864.

IR (film, cm^{-1}): 2950, 1771, 1738, 1662, 1634, 1614, 1509, 1438, 1377, 1275, 1223, 1154, 1140, 1043, 1016, 998, 968, 904, 833.

$[\alpha]_D^{20}$ = +30.0 (c = 0.15, CHCl$_3$)

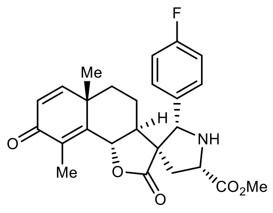

12c

Methyl (2'*S*,3*S*,3a*S*,5a*S*,5'*S*,9b*S*)-2'-(4-fluorophenyl)-5a,9-dimethyl-2,8-dioxo-3a,4,5,5a,8,9b-hexahydro-2*H*-spiro[naphtho[1,2-*b*]furan-3,3'-pyrrolidine]-5'-carboxylate

The title product compound **12c** was prepared in 0.10 mmol scale using **Condition D** and isolated by column chromatography (1:1 Cyclohexane: Ethyl acetate) giving an amorphous solid (37.6 mg, 0.09 mmol, 86% yield).

^{1}H NMR (500 MHz, CDCl$_3$) δ 7.62 – 7.48 (m, 2H), 7.14 – 7.05 (m, 2H), 6.52 (d, *J* = 9.9 Hz, 1H), 6.14 (d, *J* = 9.8 Hz, 1H), 4.51 (s, 1H), 4.19 – 4.09 (m, 2H), 3.79 (s, 3H), 3.00 (dd, *J* = 13.9, 10.8 Hz, 1H), 2.22 – 2.11 (m, 1H), 2.05 (d, *J* = 1.4 Hz, 3H), 2.00-1.93 (m, 2H), 1.89 (ddd, *J* = 14.0, 12.4, 3.9 Hz, 1H), 1.66 (ddd, *J* = 13.5, 3.9, 2.3 Hz, 1H), 1.20 (td, *J* = 12.9, 4.8 Hz, 1H), 0.64 (s, 3H).

^{13}C NMR (126 MHz, CDCl$_3$) δ 186.4, 178.6, 173.6, 162.6 (d, *J* = 248.7 Hz), 155.0, 151.3, 132.6 (d, *J* = 3.4 Hz), 128.9 (d, *J* = 7.8 Hz), 128.5, 125.9, 115.9 (d, *J* = 21.4 Hz), 79.6, 67.8, 56.9, 56.5, 54.8, 52.6, 41.2, 40.5, 38.4, 24.0, 18.1, 11.1.

HRMS(ESI): $[M+H]^+$ calcd. $C_{25}H_{27}NO_5F$ m/z 440.1868, found 440.1861.

IR (film, cm^{-1}): 2925, 2854, 1766, 1740, 1660, 1628, 1610, 1509, 1435, 1406, 1376, 1271, 1222, 1201, 1185, 1161, 1107, 1038, 988, 953, 902, 882, 834, 803.

$[\alpha]_D^{20}$ = -58.9 (c = 0.09, CHCl$_3$)

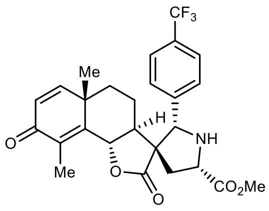

9d

Methyl (2'*S*,3*R*,3a*S*,5a*S*,5'*S*,9b*S*)-2'-(4- (trifluoromethyl)phenyl)-5a,9-dimethyl-2,8-dioxo-3a,4,5,5a,8,9b-hexahydro-2*H*-spiro[naphtho[1,2-*b*]furan-3,3'-pyrrolidine]-5'-carboxylate

The title product compound **9d** was prepared in 0.10 mmol scale using **Condition A** and isolated by column chromatography (20:1 DCM: MeOH) giving an amorphous solid (35.1 mg, 0.07 mmol, 72% yield).

^{1}H NMR (500 MHz, $CDCl_3$) δ 7.58 (d, *J* = 8.1 Hz, 2H), 7.51 (d, *J* = 8.1 Hz, 2H), 6.67 (d, *J* = 9.9 Hz, 1H), 6.22 (d, *J* = 9.9 Hz, 1H), 4.79 (dd, *J* = 11.6, 1.7 Hz, 1H), 4.16 (s, 1H), 4.06 (dd, *J* = 8.9, 5.6 Hz, 1H), 3.81 (s, 3H), 2.57 (dd, *J* = 13.5, 8.9 Hz, 1H), 2.46 (dd, *J* = 13.4, 5.6 Hz, 1H), 2.07 – 1.95 (m, 3H), 1.92 – 1.76 (m, 4H), 1.54 (ddd, *J* = 13.5, 13.0, 4.8 Hz, 1H), 1.29 (s, 3H).

^{13}C NMR (126 MHz, $CDCl_3$) δ 186.2, 175.8, 172.5, 154.5, 150.3, 139.9, 130.7 (q, *J* = 32.6 Hz), 129.3, 128.8, 126.2, 125.5 (q, *J* = 3.8 Hz), 124.0 (d, *J* = 272.2 Hz), 80.0, 67.9, 58.7, 55.2, 52.7, 50.6, 41.1, 37.5, 33.8, 25.1, 19.4, 10.9.

HRMS(ESI): $[M+H]^+$ calcd. $C_{26}H_{27}NO_5F_3$ m/z 490.1836, found 490.1819.

IR (film, cm^{-1}): 2936, 1774, 1738, 1662, 1634, 1617, 1437, 1379, 1324, 1269, 1225, 1162, 1116, 1068, 1017, 991, 906, 832.

$[\alpha]_D^{20}$ = -74.5 (c = 0.11, $CHCl_3$)

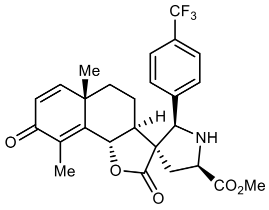

10d

Methyl (2'*R*,3*S*,3a*S*,5a*S*,5'*R*,9b*S*)-2'-(4- (trifluoromethyl)phenyl)-5a,9-dimethyl-2,8-dioxo-3a,4,5,5a,8,9b-hexahydro-2*H*-spiro[naphtho[1,2-*b*]furan-3,3'-pyrrolidine]-5'-carboxylate

The title product compound **10d** was prepared in 0.10 mmol scale using **Condition B** and isolated by column chromatography (20:1 DCM: MeOH) giving an amorphous solid (46.7 mg, 0.10 mmol, 95% yield).

^{1}H NMR (500 MHz, $CDCl_3$) δ 7.70 – 7.53 (m, 4H), 6.63 (d, *J* = 9.9 Hz, 1H), 6.19 (d, *J* = 9.9 Hz, 1H), 4.49 (s, 1H), 4.26 (dd, *J* = 12.1, 1.5 Hz, 1H), 4.02 (dd, *J* = 9.4, 5.7 Hz, 1H), 3.84 (s, 3H), 2.69 (dd, *J* = 13.6, 5.8 Hz, 1H), 2.33 (dd, *J* = 13.6, 9.4 Hz, 1H), 2.25 (td, *J* = 12.1, 4.0 Hz, 1H), 2.11 – 2.04 (m, 1H), 2.02 – 1.90 (m, 5H), 1.47 (td, *J* = 12.9, 4.8 Hz, 1H), 1.10 (s, 3H).

^{13}C NMR (126 MHz, $CDCl_3$) δ 186.2, 176.9, 173.4, 154.5, 150.4, 142.4, 131.4 (q, *J* = 32.9 Hz), 129.1, 128.4, 126.4 (q, *J* = 3.7 Hz), 126.1, 123.8 (d, *J* = 272.3 Hz), 79.4, 65.8, 58.5, 57.0, 52.9, 52.8, 41.1, 38.6, 37.8, 24.8, 19.4, 11.0.

HRMS(ESI): $[M+H]^+$ calcd. $C_{26}H_{27}NO_5F_3$ m/z 490.1836, found 490.1821.

IR (film, cm^{-1}): 2952, 1774, 1738, 1662, 1634, 1617, 1437, 1378, 1324, 1266, 1225, 1163, 1120, 1068, 1039, 1016, 993, 959, 906, 832.

$[\alpha]_D^{20}$ = -76.5 (c = 0.17, $CHCl_3$)

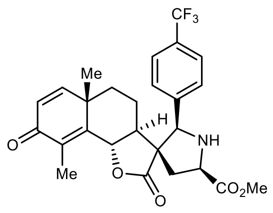

11d

Methyl (2'*R*,3*R*,3a*S*,5a*S*,5'*R*,9b*S*)-2'-(4- (trifluoromethyl)phenyl)-5a,9-dimethyl-2,8-dioxo-3a,4,5,5a,8,9b-hexahydro-2*H*-spiro[naphtho[1,2-*b*]furan-3,3'-pyrrolidine]-5'-carboxylate

The title product compound **11d** was prepared in 0.10 mmol scale using **Condition C** and isolated by column chromatography (1:1 Cyclohexane: Ethyl acetate) giving an amorphous solid (40.8 mg, 0.08 mmol, 83% yield).

^{1}H NMR (500 MHz, $CDCl_3$) δ 7.58 (d, *J* = 8.2 Hz, 2H), 7.52 (d, *J* = 8.1 Hz, 2H), 6.57 (d, *J* = 9.8 Hz, 1H), 6.19 (d, *J* = 9.9 Hz, 1H), 4.83 (s, 1H), 4.79 (dq, *J* = 11.1, 1.4 Hz, 1H), 4.24 (dd, *J* = 8.8, 7.8 Hz, 1H), 3.83 (s, 3H), 2.54 (dd, *J* = 13.4, 7.9 Hz, 1H), 2.34 (dd, *J* = 13.4, 8.8 Hz, 1H), 1.69 – 1.62 (m, 1H), 1.60 – 1.49 (m, 2H), 1.23 (m, 4H), 1.12 – 1.02 (m, 1H).

^{13}C NMR (126 MHz, $CDCl_3$) δ 186.2, 178.2, 173.2, 154.7, 150.5, 143.1, 130.6 (q, *J* = 32.6 Hz), 129.4, 127.6, 126.1, 125.7 (q, *J* = 3.7 Hz), 124.1 (q, *J* = 272.3 Hz), 79.9, 66.5, 57.1, 55.1, 52.6, 50.4, 41.0, 37.2, 31.1, 25.1, 20.1, 11.1.

HRMS(ESI): $[M+H]^+$ calcd. $C_{26}H_{27}NO_5F_3$ m/z 490.1836, found 490.1823.

IR (film, cm^{-1}): 2926, 2117, 1773, 1741, 1663, 1636, 1617, 1438, 1324, 1276, 1163, 1120, 1067, 1017, 902, 833.

$[\alpha]_D^{20}$ = +44.8 (c = 0.13, $CHCl_3$)

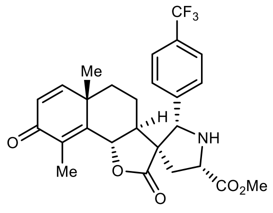

12d

Methyl (2'*S*,3*S*,3a*S*,5a*S*,5'*S*,9b*S*)-2'-(4- (trifluoromethyl)phenyl)-5a,9-dimethyl-2,8-dioxo-3a,4,5,5a,8,9b-hexahydro-2*H*-spiro[naphtho[1,2-*b*]furan-3,3'-pyrrolidine]-5'-carboxylate

The title product compound **12d** was prepared in 0.10 mmol scale using **Condition D** and isolated by column chromatography (1:1 Cyclohexane: Ethyl acetate) giving an amorphous solid (45.0 mg, 0.09 mmol, 92% yield).

^{1}H NMR (500 MHz, CDCl$_3$) δ 7.86 – 7.62 (m, 4H), 6.50 (d, *J* = 9.8 Hz, 1H), 6.13 (d, *J* = 9.9 Hz, 1H), 4.60 (s, 1H), 4.22 – 4.14 (m, 1H), 4.08 – 4.04 (m, 1H), 3.80 (s, 3H), 3.04 (dd, *J* = 13.9, 10.8 Hz, 1H), 2.20 (dd, *J* = 13.9, 5.5 Hz, 1H), 2.04 (d, *J* = 1.1 Hz, 3H), 2.01 – 1.94 (m, 2H), 1.88 – 1.75 (m, 1H), 1.64 (ddd, *J* = 13.7, 4.1, 2.2 Hz, 1H), 1.19 (td, *J* = 13.1, 4.3 Hz, 1H), 0.54 (s, 3H).

^{13}C NMR (126 MHz, CDCl$_3$) δ 186.3, 178.4, 173.3, 154.9, 150.9, 141.1, 130.0 (q, *J* = 32.9 Hz), 128.5, 127.8, 125.9, 125.8 (q, *J* = 3.7 Hz), 123.8 (q, *J* = 272.4 Hz), 79.7, 67.9, 56.8, 56.5, 55.0, 52.6, 41.1, 40.6, 38.4, 23.6, 18.2, 11.0.

HRMS(ESI): $[M+H]^+$ calcd. $C_{26}H_{27}NO_5F_3$ m/z 490.1836, found 490.1824.

IR (film, cm^{-1}): 2926, 1766, 1743, 1661, 1633, 1437, 1378, 1324, 1272, 1222, 1184, 1163, 1118, 1068, 1038, 1016, 989, 955, 913, 881, 832.

$[\alpha]_D^{20}$ = -50.0 (c = 0.21, CHCl$_3$)

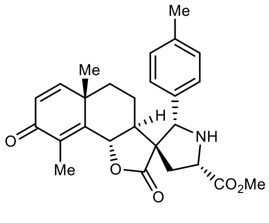

9e

Methyl (2'*S*,3*R*,3a*S*,5a*S*,5'*S*,9b*S*)-2'-(*p*-tolyl)-5a,9-dimethyl-2,8-dioxo-3a,4,5,5a,8,9b-hexahydro-2*H*-spiro[naphtho[1,2-*b*]furan-3,3'-pyrrolidine]-5'-carboxylate

The title product compound **9e** was prepared in 0.10 mmol scale using **Condition A** and isolated by column chromatography (20:1 DCM: MeOH) giving an amorphous solid (27.4 mg, 0.06 mmol, 63% yield).

^{1}H NMR (500 MHz, CDCl$_3$) δ 7.23 (d, *J* = 8.1 Hz, 2H), 7.16 – 7.10 (m, 2H), 6.67 (d, *J* = 9.9 Hz, 1H), 6.22 (d, *J* = 9.9 Hz, 1H), 4.76 (dd, *J* = 11.7, 1.3 Hz, 1H), 4.10 – 4.00 (m, 2H), 3.81 (s, 3H), 2.53 (dd, *J* = 13.5, 9.1 Hz, 1H), 2.45 (dd, *J* = 13.4, 5.2 Hz, 1H), 2.31 (s, 3H), 2.05 (td, *J* = 12.3, 3.4 Hz, 1H), 1.99 – 1.92 (m, 2H), 1.88 (d, *J* = 1.4 Hz, 3H), 1.86 – 1.77 (m, 1H), 1.52 (td, *J* = 13.1, 4.6 Hz, 1H), 1.29 (s, 3H).

^{13}C NMR (126 MHz, CDCl$_3$) δ 186.3, 176.3, 172.7, 154.6, 150.8, 138.4, 132.1, 129.4, 129.1, 128.1, 126.2, 80.1, 77.4, 68.6, 58.8, 55.1, 52.7, 50.0, 41.2, 37.7, 34.1, 25.1, 21.2, 19.5, 10.8.

HRMS(ESI): $[M+H]^+$ calcd. $C_{26}H_{30}NO_5$ m/z 436.2119, found 436.2108.

IR (film, cm^{-1}): 2924, 2160, 2036, 1777, 1737, 1659, 1627, 1611, 1514, 1434, 1404, 1377, 1338, 1303, 1267, 1211, 1189, 1163, 1141, 1088, 1056, 1026, 990, 960, 899, 842, 817.

$[\alpha]_D^{20}$ = -75.7 (c = 0.19, CHCl$_3$)

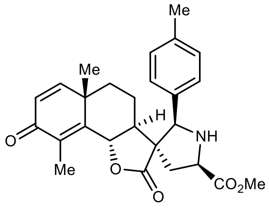

10e

Methyl (2'*R*,3*S*,3a*S*,5a*S*,5'*R*,9b*S*)-2'-(*p*-tolyl)-5a,9-dimethyl-2,8-dioxo-3a,4,5,5a,8,9b-hexahydro-2*H*-spiro[naphtho[1,2-*b*]furan-3,3'-pyrrolidine]-5'-carboxylate

The title product compound **10e** was prepared in 0.10 mmol scale using **Condition B** and isolated by column chromatography (20:1 DCM: MeOH) giving an amorphous solid (38.8 mg, 0.09 mmol, 89% yield).

^{1}H NMR (400 MHz, $CDCl_3$) δ 7.35 (d, *J* = 8.2 Hz, 2H), 7.19 (d, *J* = 7.8 Hz, 2H), 6.61 (d, *J* = 9.9 Hz, 1H), 6.17 (d, *J* = 9.9 Hz, 1H), 4.33 (s, 1H), 4.22 – 4.14 (m, 1H), 3.99 (dd, *J* = 9.9, 4.7 Hz, 1H), 3.84 (s, 3H), 2.63 (dd, *J* = 13.6, 4.7 Hz, 1H), 2.37 – 2.29 (m, 4H), 2.23 (td, *J* = 12.2, 3.4 Hz, 1H), 2.10 (td, *J* = 12.8, 4.0 Hz, 1H), 2.00 – 1.88 (m, 5H), 1.48 – 1.39 (m, 1H), 1.04 (s, 3H).

^{13}C NMR (101 MHz, $CDCl_3$) δ 186.3, 177.9, 173.5, 154.7, 151.1, 139.3, 134.3, 130.2, 128.7, 127.6, 126.0, 79.5, 66.7, 58.4, 56.6, 52.7, 52.3, 41.2, 38.9, 37.9, 24.7, 21.3, 19.0, 11.0.

HRMS(ESI): $[M+H]^+$ calcd. $C_{26}H_{30}NO_5$ m/z 436.2119, found 436.2115.

IR (film, cm^{-1}): 2927, 1773, 1732, 1661, 1635, 1615, 1518, 1436, 1373, 1343, 1319, 1310, 1267, 1219, 1169, 1149, 1116, 1095, 1064, 1041, 997, 961, 939, 894, 841, 823.

$[\alpha]_D^{20}$ = -119.0 (c = 0.10, $CHCl_3$)

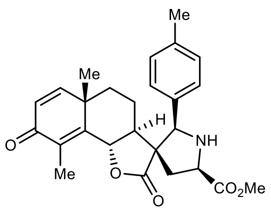

11e

Methyl (2'*R*,3*R*,3a*S*,5a*S*,5'*R*,9b*S*)-2'-(*p*-tolyl)-5a,9-dimethyl-2,8-dioxo-3a,4,5,5a,8,9b-hexahydro-2*H*-spiro[naphtho[1,2-*b*]furan-3,3'-pyrrolidine]-5'-carboxylate

The title product compound **11e** was prepared in 0.10 mmol scale using **Condition C** and isolated by column chromatography (1:1 Cyclohexane: Ethyl acetate) giving an amorphous solid (42.5 mg, 0.10 mmol, 97% yield).

^{1}H NMR (500 MHz, $CDCl_3$) δ 7.23 (d, *J* = 8.1 Hz, 2H), 7.10 (d, *J* = 8.1 Hz, 1H), 6.57 (d, *J* = 9.8 Hz, 1H), 6.17 (d, *J* = 9.8 Hz, 1H), 4.80 – 4.70 (m, 2H), 4.22 (dd, *J* = 9.0, 7.7 Hz, 1H), 3.82 (s, 3H), 2.53 (dd, *J* = 13.4, 7.6 Hz, 1H), 2.39 – 2.26 (m, 4H), 2.03 (d, *J* = 1.4 Hz, 3H), 1.69 – 1.48 (m, 3H), 1.41 – 1.34 (m, 1H), 1.22 (s, 3H), 1.08 (td, *J* = 12.9, 4.5 Hz, 1H).

^{13}C NMR (126 MHz, $CDCl_3$) δ 186.4, 178.6, 173.2, 155.0, 151.1, 138.1, 135.1, 129.5, 129.1, 126.9, 126.0, 79.9, 67.2, 57.2, 55.1, 52.6, 50.2, 41.1, 37.5, 31.4, 25.0, 21.3, 20.2, 11.0.

HRMS(ESI): $[M+H]^+$ calcd. $C_{26}H_{30}NO_5$ m/z 436.2119, found 436.2109.

IR (film, cm^{-1}): 3343, 2949, 2160, 2040, 1771, 1737, 1662, 1635, 1614, 1513, 1437, 1378, 1324, 1273, 1191, 1162, 1123, 1106, 1042, 1016, 997, 967, 902, 882, 832.

$[\alpha]_D^{20}$ = +66.7 (c = 0.14, $CHCl_3$)

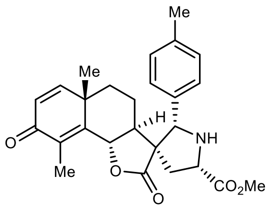

12e

Methyl (2'*S*,3*S*,3a*S*,5a*S*,5'*S*,9b*S*)-2'-(*p*-tolyl)-5a,9-dimethyl-2,8-dioxo-3a,4,5,5a,8,9b-hexahydro-2*H*-spiro[naphtho[1,2-*b*]furan-3,3'-pyrrolidine]-5'-carboxylate

The title product compound **12e** was prepared in 0.10 mmol scale using **Condition D** and isolated by column chromatography (1:1 Cyclohexane: Ethyl acetate) giving an amorphous solid (37.7 mg, 0.09 mmol, 87% yield).

^{1}H NMR (400 MHz, CDCl$_3$) δ 7.43 (d, *J* = 7.8 Hz, 2H), 7.24 – 7.15 (m, 2H), 6.50 (d, *J* = 9.9 Hz, 1H), 6.13 (d, *J* = 9.9 Hz, 1H), 4.50 (s, 1H), 4.22 – 4.08 (m, 2H), 3.80 (s, 3H), 3.02 (dd, *J* = 13.9, 10.8 Hz, 1H), 2.33 (s, 3H), 2.17 – 2.09 (m, 1H), 2.06 (d, *J* = 1.4 Hz, 3H), 2.04 – 1.90 (m, 3H), 1.69 – 1.60 (m, 1H), 1.24 – 1.12 (m, 1H), 0.58 (s, 3H).

^{13}C NMR (101 MHz, CDCl$_3$) δ 186.4, 178.8, 173.7, 155.1, 151.6, 138.5, 133.4, 129.5, 128.4, 127.1, 125.9, 79.6, 68.4, 57.1, 56.7, 55.0, 52.5, 41.2, 40.8, 38.6, 23.8, 21.2, 18.1, 11.1.

HRMS(ESI): $[M+H]^+$ calcd. $C_{26}H_{30}NO_5$ m/z 436.2119, found 436.2106.

IR (film, cm^{-1}): 3296, 2956, 1764, 1744, 1659, 1628, 1610, 1519, 1432, 1407, 1376, 1347, 1324, 1310, 1273, 1234, 1223, 1199, 1185, 1166, 1136, 1105, 1092, 1036, 1020, 989, 954, 932, 915, 900, 884, 855, 847, 836, 821.

$[\alpha]_D^{20}$ = -78.1 (c = 0.16, CHCl$_3$)

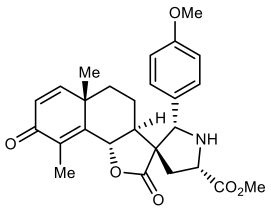

9f

Methyl (2'*S*,3*R*,3a*S*,5a*S*,5'*S*,9b*S*)-2'-(4-methoxyphenyl)-5a,9-dimethyl-2,8-dioxo-3a,4,5,5a,8,9b-hexahydro-2*H*-spiro[naphtho[1,2-*b*]furan-3,3'-pyrrolidine]-5'-carboxylate

The title product compound **9f** was prepared in 0.10 mmol scale using **Condition A** and isolated by column chromatography (20:1 DCM: MeOH) giving an amorphous solid (30.1 mg, 0.07 mmol, 67% yield).

^{1}H NMR (500 MHz, CDCl$_3$) δ 7.33 – 7.21 (m, 2H), 6.90 – 6.81 (m, 2H), 6.66 (d, *J* = 9.9 Hz, 1H), 6.21 (d, *J* = 9.9 Hz, 1H), 4.76 (dd, *J* = 11.9, 1.4 Hz, 1H), 4.04 (dd, *J* = 9.1, 5.1 Hz, 1H), 4.01 (s, 1H), 3.80 (s, 3H), 3.77 (s, 3H), 2.51 (dd, *J* = 13.5, 9.1 Hz, 1H), 2.44 (dd, *J* = 13.5, 5.1 Hz, 1H), 2.03 – 1.98 (m, 1H), 1.97 – 1.90 (m, 2H), 1.87 (d, *J* = 1.4 Hz, 3H), 1.81 (qd, *J* = 12.8, 4.0 Hz, 1H), 1.51 (td, *J* = 13.1, 4.7 Hz, 1H), 1.28 (s, 3H).

^{13}C NMR (126 MHz, CDCl$_3$) δ 186.3, 176.4, 172.7, 159.7, 154.7, 150.8, 129.4, 129.1, 127.1, 126.1, 114.0, 80.1, 68.4, 58.7, 55.3, 55.0, 52.7, 49.9, 41.2, 37.6, 34.0, 25.1, 19.5, 10.8.

HRMS(ESI): $[M+H]^+$ calcd. $C_{26}H_{30}NO_6$ m/z 452.2068, found 452.2056.

IR (film, cm^{-1}): 2936, 2161, 1772, 1737, 1661, 1634, 1613, 1515, 1437, 1377, 1247, 1224, 1182, 1131, 1058, 1032, 991, 961, 906, 831.

$[\alpha]_D^{20}$ = -86.7 (c = 0.12, $CHCl_3$)

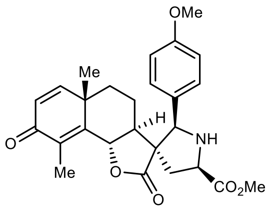

10f

Methyl (2'*R*,3*S*,3a*S*,5a*S*,5'*R*,9b*S*)-2'-(4-methoxyphenyl)-5a,9-dimethyl-2,8-dioxo-3a,4,5,5a,8,9b-hexahydro-2*H*-spiro[naphtho[1,2-*b*]furan-3,3'-pyrrolidine]-5'-carboxylate

The title product compound **10f** was prepared in 0.10 mmol scale using **Condition B** and isolated by column chromatography (20:1 DCM: MeOH) giving an amorphous solid (38.1 mg, 0.08 mmol, 84% yield).

^{1}H NMR (500 MHz, $CDCl_3$) δ 7.40 (d, *J* = 8.7 Hz, 2H), 6.91 (d, *J* = 8.7 Hz, 2H), 6.61 (d, *J* = 9.9 Hz, 1H), 6.18 (d, *J* = 9.9 Hz, 1H), 4.32 (s, 1H), 4.18 (dd, *J* = 11.9, 1.5 Hz, 1H), 3.98 (dd, *J* = 9.9, 4.7 Hz, 1H), 3.84 (s, 3H), 3.80 (s, 3H), 2.63 (dd, *J* = 13.6, 4.7 Hz, 1H), 2.33 (dd, *J* = 13.6, 9.9 Hz, 1H), 2.23 (td, *J* = 12.3, 3.5 Hz, 1H), 2.14 – 2.02 (m, 1H), 1.98 (d, *J* = 1.3 Hz, 3H), 1.97 – 1.88 (m, 2H), 1.43 (td, *J* = 13.2, 4.5 Hz, 1H), 1.05 (s, 3H).

^{13}C NMR (126 MHz, $CDCl_3$) δ 186.3, 178.0, 173.5, 160.2, 154.6, 151.1, 129.2, 128.9, 128.8, 126.1, 114.8, 79.5, 66.4, 58.3, 56.4, 55.4, 52.7, 52.3, 41.2, 38.8, 37.9, 24.8, 19.0, 11.0.

HRMS(ESI): $[M+H]^+$ calcd. $C_{26}H_{30}NO_6$ m/z 452.2068, found 452.2056.

IR (film, cm^{-1}): 2957, 1770, 1724, 1661, 1634, 1614, 1517, 1436, 1372, 1321, 1268, 1225, 1187, 1151, 1117, 1096, 1067, 1042, 1033, 990, 959, 940, 903, 890, 850, 840, 829, 822.

$[\alpha]_D^{20}$ = -92.4 (c = 0.21, $CHCl_3$)

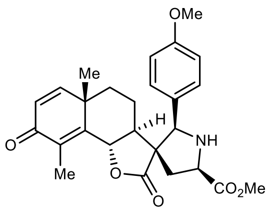

11f

Methyl (2'*R*,3*R*,3a*S*,5a*S*,5'*R*,9b*S*)-2'-(4-methoxyphenyl)-5a,9-dimethyl-2,8-dioxo-3a,4,5,5a,8,9b-hexahydro-2*H*-spiro[naphtho[1,2-*b*]furan-3,3'-pyrrolidine]-5'-carboxylate

The title product compound **11f** was prepared in 0.10 mmol scale using **Condition C** and isolated by column chromatography (1:1 Cyclohexane: Ethyl acetate) giving an amorphous solid (42.3 mg, 0.09 mmol, 94% yield).

^{1}H NMR (700 MHz, $CDCl_3$) δ 7.27 (d, *J* = 8.2 Hz, 2H), 6.84 (d, *J* = 8.3 Hz, 2H), 6.57 (d, *J* = 9.8 Hz, 1H), 6.18 (d, *J* = 9.8 Hz, 1H), 4.77 – 4.74 (m, 1H), 4.73 (s, 1H), 4.20 (t, *J* = 8.3 Hz, 1H), 3.82 (s, 3H), 3.78 (s, 3H), 2.51 (dd, *J* =

13.4, 7.6 Hz, 1H), 2.31 (dd, *J* = 13.4, 9.0 Hz, 1H), 2.08 – 1.98 (m, 3H), 1.69 – 1.61 (m, 2H), 1.54 (qd, *J* = 12.8, 4.0 Hz, 1H), 1.39 – 1.34 (m, 1H), 1.23 (s, 3H), 1.10 (td, *J* = 13.2, 4.6 Hz, 1H).

^{13}C NMR (176 MHz, $CDCl_3$) δ 186.4, 178.7, 173.5, 159.5, 154.9, 151.1, 130.6, 129.1, 128.2, 126.0, 114.1, 79.9, 66.9, 57.2, 55.4, 55.2, 52.5, 50.2, 41.1, 37.6, 31.4, 25.1, 20.2, 11.1.

HRMS(ESI): $[M+H]^+$ calcd. $C_{26}H_{30}NO_6$ m/z 452.2068, found 452.2064.

IR (film, cm^{-1}): 2927, 2250, 1771, 1738, 1662, 1634, 1612, 1512, 1455, 1438, 1378, 1325, 1247, 1227, 1181, 1139, 1106, 1079, 1034, 1018, 997, 967, 903, 832.

$[\alpha]_D^{20}$ = +50.6 (c = 0.16, $CHCl_3$)

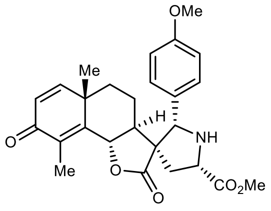

12f

Methyl (2'*S*,3*S*,3a*S*,5a*S*,5'*S*,9b*S*)-2'-(4-methoxyphenyl)-5a,9-dimethyl-2,8-dioxo-3a,4,5,5a,8,9b-hexahydro-2*H*-spiro[naphtho[1,2-*b*]furan-3,3'-pyrrolidine]-5'-carboxylate

The title product compound **12f** was prepared in 0.10 mmol scale using **Condition D** and isolated by column chromatography (1:1 Cyclohexane: Ethyl acetate) giving an amorphous solid (35.6 mg, 0.08 mmol, 79% yield).

^{1}H NMR (500 MHz, $CDCl_3$) δ 7.45 (d, *J* = 8.2 Hz, 2H), 6.90 (d, *J* = 9.0 Hz, 2H), 6.51 (d, *J* = 9.9 Hz, 1H), 6.13 (d, *J* = 9.8 Hz, 1H), 4.48 (s, 1H), 4.29 – 4.05 (m, 2H), 3.78 (s, 3H+3H), 2.99 (dd, *J* = 13.9, 10.7 Hz, 1H), 2.13 (dd, *J* = 13.9, 5.7 Hz, 1H), 2.04 (d, *J* = 1.4 Hz, 3H), 1.95 (dd, *J* = 8.2, 3.8 Hz, 3H), 1.69 – 1.60 (m, 1H), 1.25 – 1.13 (m, 2H), 0.62 (s, 3H).

^{13}C NMR (126 MHz, $CDCl_3$) δ 186.5, 178.9, 173.6, 159.7, 155.1, 151.6, 128.3, 128.3, 125.8, 114.2, 79.6, 68.1, 56.9, 56.6, 55.5, 54.8, 52.5, 41.2, 40.5, 38.5, 24.0, 18.0, 11.0.

HRMS(ESI): $[M+H]^+$ calcd. $C_{26}H_{30}NO_6$ m/z 452.2068, found 452.2064.

IR (film, cm^{-1}): 3297, 2920, 2850, 1762, 1743, 1659, 1631, 1611, 1584, 1517, 1434, 1408, 1376, 1332, 1312, 1274, 1256, 1234, 1222, 1182, 1167, 1136, 1110, 1031, 1018, 989, 952, 930, 915, 901, 883, 833, 822.

$[\alpha]_D^{20}$ = -95.5 (c = 0.20, $CHCl_3$)

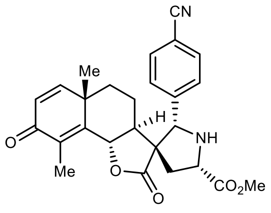

9g

Methyl (2'*S*,3*R*,3a*S*,5a*S*,5'*S*,9b*S*)-2'-(4-cyanophenyl)-5a,9-dimethyl-2,8-dioxo-3a,4,5,5a,8,9b-hexahydro-2*H*-spiro[naphtho[1,2-*b*]furan-3,3'-pyrrolidine]-5'-carboxylate

The title product compound **9g** was prepared in 0.10 mmol scale using **Condition A** and isolated by column chromatography (20:1 DCM: MeOH) giving an amorphous solid (32.7 mg, 0.07 mmol, 73% yield).

^{1}H NMR (500 MHz, CDCl$_3$) δ 7.62 (d, *J* = 8.4 Hz, 2H), 7.53 (d, *J* = 8.5 Hz, 2H), 6.68 (d, *J* = 9.9 Hz, 1H), 6.23 (d, *J* = 9.9 Hz, 1H), 4.80 (dq, *J* = 11.8, 1.4 Hz, 1H), 4.19 (s, 1H), 4.05 (dd, *J* = 8.7, 5.9 Hz, 1H), 3.81 (s, 3H), 2.58 (dd, *J* = 13.4, 8.7 Hz, 1H), 2.46 (dd, *J* = 13.4, 5.9 Hz, 1H), 2.06 – 1.96 (m, 4H), 1.89 (m, 4H), 1.61 – 1.50 (m, 1H), 1.31 (s, 3H).

^{13}C NMR (126 MHz, CDCl$_3$) δ 186.1, 175.5, 172.3, 154.5, 150.1, 141.5, 132.3, 129.4, 129.1, 126.2, 118.6, 112.5, 79.9, 67.8, 58.7, 55.3, 52.8, 51.0, 41.1, 37.4, 33.7, 25.2, 19.3, 10.9.

HRMS(ESI): $[M+H]^+$ calcd. $C_{26}H_{27}N_2O_5$ m/z 447.1915, found 447.1903.

IR (film, cm^{-1}): 2932, 2227, 1774, 1735, 1661, 1633, 1612, 1504, 1437, 1374, 1305, 1267, 1225, 1205, 1185, 1155, 1133, 1025, 991, 961, 906, 832.

$[\alpha]_D^{20}$ = -82.2 (c = 0.14, $CHCl_3$)

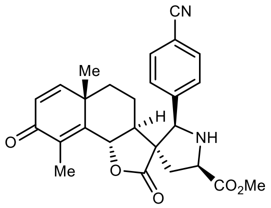

10g

Methyl (2'*R*,3*S*,3a*S*,5a*S*,5'*R*,9b*S*)-2'-(4-cyanophenyl)-5a,9-dimethyl-2,8-dioxo-3a,4,5,5a,8,9b-hexahydro-2*H*-spiro[naphtho[1,2-*b*]furan-3,3'-pyrrolidine]-5'-carboxylate

The title product compound **10g** was prepared in 0.10 mmol scale using **Condition B** and isolated by column chromatography (20:1 DCM: MeOH) giving an amorphous solid (39.3 mg, 0.09 mmol, 88% yield).

^{1}H NMR (500 MHz, CDCl$_3$) δ 7.68 (d, *J* = 8.5 Hz, 2H), 7.61 (d, *J* = 8.4 Hz, 2H), 6.64 (d, *J* = 9.9 Hz, 1H), 6.21 (d, *J* = 9.9 Hz, 1H), 4.52 (s, 1H), 4.37 – 4.30 (m, 1H), 4.03 (dd, *J* = 9.1, 6.3 Hz, 1H), 3.84 (s, 3H), 2.70 (dd, *J* = 13.5, 6.3 Hz, 1H), 2.31 (dd, *J* = 13.7, 9.1 Hz, 1H), 2.28 – 2.20 (m, 1H), 2.06 (dd, *J* = 12.4, 8.7 Hz, 1H), 2.02 – 1.91 (m, 5H), 1.49 (m, 1H), 1.15 (s, 3H).

^{13}C NMR (126 MHz, CDCl$_3$) δ 186.1, 176.4, 173.4, 154.4, 150.2, 144.2, 133.0, 129.3, 128.7, 126.2, 118.3, 113.1, 79.4, 65.4, 58.6, 57.4, 53.2, 52.8, 41.1, 38.4, 37.7, 24.9, 19.6, 11.0.

HRMS(ESI): $[M+H]^+$ calcd. $C_{26}H_{27}N_2O_5$ m/z 447.1915, found 447.1903.

IR (film, cm^{-1}): 3376, 2929, 2227, 1774, 1737, 1661, 1633, 1610, 1504, 1437, 1377, 1305, 1265, 1202, 1181, 1155, 1118, 1093, 1041, 993, 959, 905, 833.

$[\alpha]_D^{20}$ = -88.8 (c = 0.21, $CHCl_3$)

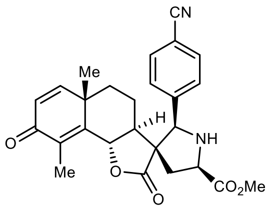

11g

Methyl (2'*R*,3*R*,3a*S*,5a*S*,5'*R*,9b*S*)-2'-(4-cyanophenyl)-5a,9-dimethyl-2,8-dioxo-3a,4,5,5a,8,9b-hexahydro-2*H*-spiro[naphtho[1,2-*b*]furan-3,3'-pyrrolidine]-5'-carboxylate

The title product compound **11g** was prepared in 0.10 mmol scale using **Condition C** and isolated by column chromatography (1:1 Cyclohexane: Ethyl acetate) giving an amorphous solid (38.6 mg, 0.09 mmol, 86% yield).

^{1}H NMR (700 MHz, CDCl$_3$) δ 7.62 (d, *J* = 8.6 Hz, 2H), 7.54 (d, *J* = 8.0 Hz, 2H), 6.57 (d, *J* = 9.8 Hz, 1H), 6.19 (d, *J* = 9.9 Hz, 1H), 4.82 (s, 1H), 4.80 – 4.76 (m, 1H), 4.25 (t, *J* = 8.3 Hz, 1H), 3.83 (s, 3H), 2.52 (dd, *J* = 13.5, 7.9 Hz, 1H), 2.34 (dd, *J* = 13.4, 8.7 Hz, 1H), 2.05 (d, *J* = 1.5 Hz, 3H), 1.69 – 1.63 (m, 1H), 1.56 – 1.53 (m, 2H), 1.23 (s, 3H), 1.17 – 1.12 (m, 1H), 1.06 (td, *J* = 12.5, 4.4 Hz, 1H).

^{13}C NMR (176 MHz, CDCl$_3$) δ 186.1, 178.0, 173.1, 154.6, 150.3, 144.7, 132.6, 129.4, 128.1, 126.1, 118.6, 112.4, 80.0, 66.4, 57.2, 55.3, 52.6, 50.4, 41.0, 37.3, 31.1, 25.1, 20.1, 11.1.

HRMS(ESI): $[M+H]^+$ calcd. $C_{26}H_{27}N_2O_5$ m/z 447.1915, found 447.1905.

IR (film, cm^{-1}): 2925, 2227, 1771, 1738, 1662, 1634, 1611, 1504, 1438, 1377, 1276, 1201, 1153, 1141, 1106, 1080, 1043, 1018, 998, 968, 903, 883, 833.

$[\alpha]_D^{20}$ = -32.7 (c = 0.25, $CHCl_3$)

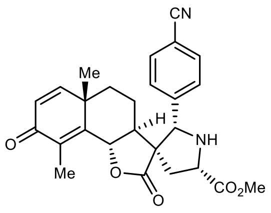

12g

Methyl (2'*S*,3*S*,3a*S*,5a*S*,5'*S*,9b*S*)-2'-(4-cyanophenyl)-5a,9-dimethyl-2,8-dioxo-3a,4,5,5a,8,9b-hexahydro-2*H*-spiro[naphtho[1,2-*b*]furan-3,3'-pyrrolidine]-5'-carboxylate

The title product compound **12g** was prepared in 0.10 mmol scale using **Condition D** and isolated by column chromatography (1:1 Cyclohexane: Ethyl acetate) giving an amorphous solid (35.9 mg, 0.08 mmol, 80% yield).

^{1}H NMR (400 MHz, CDCl$_3$) δ 7.75-7.67 (m, 4H), 6.51 (d, *J* = 9.9 Hz, 1H), 6.15 (d, *J* = 9.9 Hz, 1H), 4.57 (s, 1H), 4.17 (dd, *J* = 10.7, 5.4 Hz, 1H), 4.07 (dd, *J* = 12.2, 1.5 Hz, 1H), 3.79 (s, 3H), 3.02 (dd, *J* = 13.9, 10.7 Hz, 1H), 2.22 (dd, *J* = 13.9, 5.4 Hz, 1H), 2.05 (d, *J* = 1.4 Hz, 3H), 1.98 (dd, *J* = 14.5, 3.7 Hz, 2H), 1.77 – 1.63 (m, 2H), 1.20 (ddd, *J* = 9.0, 8.5, 3.7 Hz, 1H), 0.60 (s, 3H).

^{13}C NMR (101 MHz, CDCl$_3$) δ 186.2, 178.1, 173.3, 154.7, 150.7, 142.9, 132.6, 128.7, 128.2, 126.0, 118.2, 112.5, 79.7, 67.9, 56.8, 56.4, 55.1, 52.6, 41.1, 40.5, 38.4, 24.0, 18.4, 11.0.

HRMS(ESI): [M+H]$^+$ calcd. $C_{26}H_{27}N_2O_5$ m/z 447.1915, found 447.1908.

IR (film, cm^{-1}): 2925, 2854, 2228, 1771, 1739, 1662, 1634, 1610, 1504, 1437, 1406, 1378, 1271, 1201, 1185, 1161, 1106, 1040, 1018, 989, 959, 907, 880, 831.

$[\alpha]_D^{20}$ = -82.7 (c = 0.14, $CHCl_3$)

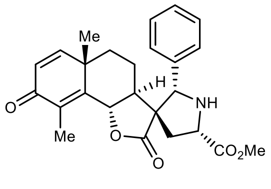

9h

Methyl (2'*S*,3*R*,3a*S*,5a*S*,5'*S*,9b*S*)-2'-phenyl-5a,9-dimethyl-2,8-dioxo-3a,4,5,5a,8,9b-hexahydro-2*H*-spiro[naphtho[1,2-*b*]furan-3,3'-pyrrolidine]-5'-carboxylate

The title product compound **9h** was prepared in 0.10 mmol scale using **Condition A** and isolated by column chromatography (20:1 DCM: MeOH) giving an amorphous solid (24.1 mg, 0.06 mmol, 57% yield).

^{1}H NMR (500 MHz, $CDCl_3$) δ 7.37 – 7.28 (m, 5H), 6.67 (d, *J* = 9.9 Hz, 1H), 6.23 (d, *J* = 9.9 Hz, 1H), 4.77 (dd, *J* = 11.9, 1.5 Hz, 1H), 4.11 – 4.04 (m, 2H), 3.82 (s, 3H), 2.55 (dd, *J* = 13.5, 9.1 Hz, 1H), 2.47 (dd, *J* = 13.4, 5.2 Hz, 1H), 2.11 – 2.04 (m, 2H), 2.00 – 1.93 (m, 2H), 1.87 (d, *J* = 1.4 Hz, 3H), 1.86 – 1.78 (m, 1H), 1.54 (ddd, *J* = 13.7, 13.3, 4.6 Hz, 1H), 1.30 (s, 3H).

^{13}C NMR (126 MHz, $CDCl_3$) δ 186.3, 176.2, 172.7, 154.6, 150.7, 135.2, 129.2, 128.7, 128.7, 128.3, 126.2, 80.1, 68.8, 58.9, 55.1, 52.7, 50.1, 41.2, 37.7, 34.1, 25.1, 19.6, 10.8.

HRMS(ESI): [M+H]$^+$ calcd. $C_{25}H_{28}NO_5$ m/z 422.1962, found 422.1955.

IR (film, cm^{-1}): 2938, 2162, 1787, 1732, 1657, 1626, 1610, 1495, 1438, 1404, 1385, 1338, 1292, 1267, 1223, 1189, 1164, 1131, 1091, 1072, 1056, 1020, 991, 957, 900, 844, 820.

$[\alpha]_D^{20}$ = -81.2 (c = 0.17, $CHCl_3$)

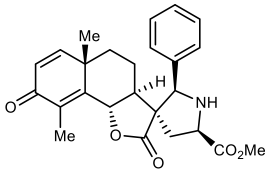

10h

Methyl (2'*R*,3*S*,3a*S*,5a*S*,5'*R*,9b*S*)-2'-phenyl-5a,9-dimethyl-2,8-dioxo-3a,4,5,5a,8,9b-hexahydro-2*H*-spiro[naphtho[1,2-*b*]furan-3,3'-pyrrolidine]-5'-carboxylate

The title product compound **10h** was prepared in 0.10 mmol scale using **Condition B** and isolated by column chromatography (20:1 DCM: MeOH) giving an amorphous solid (38.3 mg, 0.09 mmol, 91% yield).

^{1}H NMR (500 MHz, $CDCl_3$) δ 7.53 – 7.46 (m, 2H), 7.46 – 7.32 (m, 3H), 6.61 (d, *J* = 9.9 Hz, 1H), 6.18 (d, *J* = 9.9 Hz, 1H), 4.36 (s, 1H), 4.14 (dd, *J* = 11.9, 1.5 Hz, 1H), 4.01 (dd, *J* = 9.9, 4.7 Hz, 1H), 3.85 (s, 3H), 2.65 (dd, *J* = 13.6,

4.7 Hz, 1H), 2.34 (dd, *J* = 13.6, 9.9 Hz, 1H), 2.24 (td, *J* = 12.3, 3.5 Hz, 1H), 2.14 – 2.03 (m, 1H), 1.97 (d, *J* = 1.5 Hz, 3H), 1.96 – 1.84 (m, 2H), 1.44 (td, *J* = 13.1, 4.5 Hz, 1H), 1.03 (s, 3H).

^{13}C NMR (126 MHz, CDCl$_3$) δ 186.3, 177.8, 173.5, 154.6, 151.0, 137.5, 129.5, 129.4, 128.8, 127.8, 126.0, 79.4, 67.0, 58.5, 56.7, 52.7, 52.3, 41.2, 38.9, 37.9, 24.7, 19.0, 11.0.

HRMS(ESI): $[M+H]^+$ calcd. $C_{25}H_{28}NO_5$ m/z 422.1962, found 422.1949.

IR (film, cm^{-1}): 3351, 2930, 2156, 1771, 1729, 1663, 1636, 1616, 1495, 1455, 1433, 1405, 1376, 1322, 1268, 1226, 1207, 1182, 1153, 1111, 1095, 1061, 1041, 992, 960, 941, 902, 882, 836.

$[\alpha]_D^{20}$ = -103.6 (c = 0.28, $CHCl_3$)

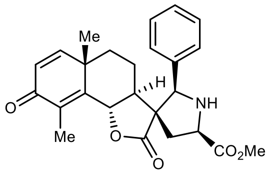

11h

Methyl (2'*R*,3*R*,3a*S*,5a*S*,5'*R*,9b*S*)-2'-phenyl-5a,9-dimethyl-2,8-dioxo-3a,4,5,5a,8,9b-hexahydro-2*H*-spiro[naphtho[1,2-*b*]furan-3,3'-pyrrolidine]-5'-carboxylate

The title product compound **11h** was prepared in 0.10 mmol scale using **Condition C** and isolated by column chromatography (1:1 Cyclohexane: Ethyl acetate) giving an amorphous solid (34.7 mg, 0.08 mmol, 82% yield).

^{1}H NMR (700 MHz, CDCl$_3$) δ 7.38 – 7.34 (m, 2H), 7.32-7.25 (m, 3H), 6.55 (d, *J* = 9.8 Hz, 1H), 6.16 (d, *J* = 9.8 Hz, 1H), 4.79 – 4.73 (m, 2H), 4.22 (dd, *J* = 8.8, 7.9 Hz, 1H), 3.82 (s, 3H), 2.51 (dd, *J* = 13.4, 7.9 Hz, 1H), 2.32 (dd, *J* = 13.4, 8.8 Hz, 1H), 2.03 (d, *J* = 1.4 Hz, 3H), 1.66 – 1.58 (m, 2H), 1.51 (qd, *J* = 12.9, 4.0 Hz, 1H), 1.25 – 1.20 (m, 4H), 1.05 (td, *J* = 13.2, 4.6 Hz, 1H).

^{13}C NMR (176 MHz, CDCl$_3$) δ 186.3, 178.6, 173.4, 154.9, 151.0, 138.9, 129.1, 128.7, 128.3, 127.0, 126.0, 79.9, 67.1, 57.2, 55.2, 52.5, 50.3, 41.1, 37.5, 31.3, 25.0, 20.1, 11.0.

HRMS(ESI): $[M+H]^+$ calcd. $C_{25}H_{28}NO_5$ m/z 422.1962, found 422.1950.

IR (film, cm^{-1}): 2950, 1771, 1738, 1661, 1634, 1614, 1493, 1454, 1437, 1378, 1274, 1192, 1153, 1139, 1055, 1017, 997, 967, 902, 833.

$[\alpha]_D^{20}$ = +37.8 (c = 0.19, $CHCl_3$)

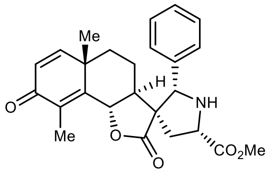

12h

Methyl (2'*S*,3*S*,3a*S*,5a*S*,5'*S*,9b*S*)-2'-phenyl-5a,9-dimethyl-2,8-dioxo-3a,4,5,5a,8,9b-hexahydro-2*H*-spiro[naphtho[1,2-*b*]furan-3,3'-pyrrolidine]-5'-carboxylate

The title product compound **12h** was prepared in 0.10 mmol scale using **Condition D** and isolated by column chromatography (1:1 Cyclohexane: Ethyl acetate) giving an amorphous solid (39.8 mg, 0.09 mmol, 94% yield).

^{1}H NMR (500 MHz, CDCl$_3$) δ 7.55 (d, *J* = 7.5 Hz, 2H), 7.38 (dd, *J* = 8.3, 7.0 Hz, 2H), 7.35 – 7.28 (m, 1H), 6.50 (d, *J* = 9.9 Hz, 1H), 6.12 (d, *J* = 9.8 Hz, 1H), 4.53 (s, 1H), 4.20 – 4.09 (m, 2H), 3.79 (s, 3H), 3.02 (dd, *J* = 13.8, 10.8 Hz, 1H), 2.14 (dd, *J* = 13.9, 5.6 Hz, 1H), 2.05 (d, *J* = 1.4 Hz, 3H), 1.99 – 1.92 (m, 3H), 1.64 (dd, *J* = 13.5, 3.0 Hz, 1H), 1.17 (ddd, *J* = 13.2, 8.7, 6.7 Hz, 1H), 0.55 (s, 3H).

^{13}C NMR (126 MHz, CDCl$_3$) δ 186.4, 178.8, 173.7, 155.1, 151.5, 136.6, 128.9, 128.6, 128.3, 127.1, 125.8, 79.6, 68.5, 57.1, 56.6, 54.9, 52.5, 41.2, 40.8, 38.5, 23.9, 18.1, 11.0.

HRMS(ESI): $[M+H]^+$ calcd. $C_{25}H_{28}NO_5$ m/z 422.1962, found 422.1947.

IR (film, cm^{-1}): 2929, 1765, 1742, 1661, 1633, 1613, 1453, 1435, 1377, 1311, 1272, 1223, 1199, 1183, 1162, 1107, 1039, 989, 953, 914, 832.

$[\alpha]_D^{20}$ = -56.9 (c = 0.16, CHCl$_3$)

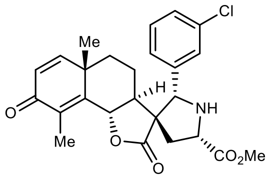

9i

Methyl (2'*S*,3*R*,3a*S*,5a*S*,5'*S*,9b*S*)-2'-(3-chlorophenyl)-5a,9-dimethyl-2,8-dioxo-3a,4,5,5a,8,9b-hexahydro-2*H*-spiro[naphtho[1,2-*b*]furan-3,3'-pyrrolidine]-5'-carboxylate

The title product compound **9i** was prepared in 0.10 mmol scale using **Condition A** and isolated by column chromatography (20:1 DCM: MeOH) giving an amorphous solid (30.0 mg, 0.07 mmol, 66% yield).

^{1}H NMR (500 MHz, CDCl$_3$) δ 7.37 (s, 1H), 7.28 – 7.23 (m, 3H), 6.67 (d, *J* = 9.9 Hz, 1H), 6.23 (d, *J* = 9.9 Hz, 1H), 4.78 (dd, *J* = 11.7, 1.4 Hz, 1H), 4.05 (s, 1H), 4.03 (dd, *J* = 8.9, 5.6 Hz, 1H), 3.81 (s, 3H), 2.54 (dd, *J* = 13.5, 8.9 Hz, 1H), 2.44 (dd, *J* = 13.4, 5.7 Hz, 1H), 2.07 – 2.01 (m, 1H), 1.99 – 1.92 (m, 2H), 1.91 (d, *J* = 1.4 Hz, 3H), 1.84 (qd, *J* = 13.2, 4.3 Hz, 1H), 1.55 (ddd, *J* = 13.5, 13.1, 4.6 Hz, 1H), 1.29 (s, 3H).

^{13}C NMR (126 MHz, CDCl$_3$) δ 186.2, 175.9, 172.4, 154.6, 150.4, 137.8, 134.5, 129.9, 129.3, 128.9, 128.6, 126.4, 126.2, 80.0, 67.9, 58.7, 55.0, 52.7, 50.5, 41.1, 37.5, 33.9, 25.2, 19.4, 10.9.

HRMS(ESI): $[M+H]^+$ calcd. $C_{25}H_{27}NO_5Cl$ m/z 456.1572, found 456.1569.

IR (film, cm^{-1}): 3328, 2937, 2349, 2161, 1783, 1732, 1658, 1630, 1611, 1597, 1573, 1438, 1403, 1379, 1339, 1291, 1269, 1225, 1209, 1186, 1165, 1133, 1093, 1076, 1022, 990, 959, 900, 840.

$[\alpha]_D^{20}$ = -79.6 (c = 0.25, CHCl$_3$)

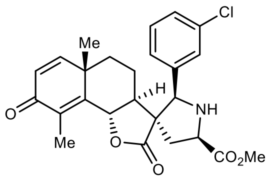

10i

Methyl (2'*R*,3*S*,3a*S*,5a*S*,5'*R*,9b*S*)-2'-(3-chlorophenyl)-5a,9-dimethyl-2,8-dioxo-3a,4,5,5a,8,9b-hexahydro-2*H*-spiro[naphtho[1,2-*b*]furan-3,3'-pyrrolidine]-5'-carboxylate

The title product compound **10i** was prepared in 0.10 mmol scale using **Condition B** and isolated by column chromatography (20:1 DCM: MeOH) giving an amorphous solid (40.4 mg, 0.09 mmol, 89% yield).

^{1}H NMR (500 MHz, $CDCl_3$) δ 7.46 (s, 1H), 7.41 – 7.31 (m, 3H), 6.63 (d, *J* = 9.9 Hz, 1H), 6.19 (d, *J* = 9.9 Hz, 1H), 4.36 (s, 1H), 4.24 (dd, *J* = 11.8, 1.5 Hz, 1H), 4.00 (dd, *J* = 9.7, 5.3 Hz, 1H), 3.84 (s, 3H), 2.66 (dd, *J* = 13.6, 5.3 Hz, 1H), 2.41 – 2.19 (m, 2H), 2.12 – 1.90 (m, 7H), 1.51-1.41 (m, 1H), 1.11 (s, 3H).

^{13}C NMR (126 MHz, $CDCl_3$) δ 186.2, 177.1, 173.3, 154.6, 150.7, 140.1, 135.1, 131.0, 129.5, 129.0, 128.4, 126.1, 125.6, 79.4, 66.0, 58.4, 56.9, 52.7, 52.6, 41.2, 38.6, 37.8, 24.6, 19.2, 11.0.

HRMS(ESI): $[M+H]^+$ calcd. $C_{25}H_{27}NO_5Cl$ m/z 456.1572, found 456.1560.

IR (film, cm^{-1}): 2928, 1774, 1737, 1661, 1634, 1614, 1597, 1573, 1436, 1377, 1306, 1265, 1202, 1181, 1154, 1097, 1039, 992, 959, 906, 833.

$[\alpha]_D^{20}$ = -105.3 (c = 0.15, $CHCl_3$)

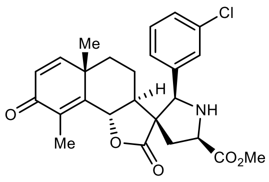

11i

Methyl (2'*R*,3*R*,3a*S*,5a*S*,5'*R*,9b*S*)-2'-(3-chlorophenyl)-5a,9-dimethyl-2,8-dioxo-3a,4,5,5a,8,9b-hexahydro-2*H*-spiro[naphtho[1,2-*b*]furan-3,3'-pyrrolidine]-5'-carboxylate

The title product compound **11i** was prepared in 0.10 mmol scale using **Condition C** and isolated by column chromatography (1:1 Cyclohexane: Ethyl acetate) giving an amorphous solid (36.6 mg, 0.08 mmol, 80% yield).

^{1}H NMR (700 MHz, CD_2Cl_2) δ 7.47 (s, 1H), 7.29 – 7.18 (m, 3H), 6.59 (d, *J* = 9.8 Hz, 1H), 6.13 (d, *J* = 9.8 Hz, 1H), 4.79 (d, *J* = 11.4 Hz, 1H), 4.72 (s, 1H), 4.21 (t, *J* = 8.3 Hz, 1H), 3.80 (s, 3H), 2.49 (dd, *J* = 13.4, 8.0 Hz, 1H), 2.32 (dd, *J* = 13.4, 8.7 Hz, 1H), 2.00 (s, 3H), 1.69 – 1.61 (m, 2H), 1.55 – 1.50 (m, 1H), 1.22 (s, 3H), 1.19 (ddd, *J* = 13.4, 5.4, 2.9 Hz, 1H), 1.09 (td, *J* = 13.2, 4.6 Hz, 1H).

^{13}C NMR (176 MHz, CD_2Cl_2) δ 186.2, 178.5, 173.5, 155.3, 151.3, 142.1, 134.9, 130.3, 129.0, 128.5, 127.5, 126.0, 125.9, 80.3, 66.7, 57.5, 55.5, 52.6, 50.6, 41.4, 37.6, 31.5, 27.3, 25.1, 20.4, 11.0.

HRMS(ESI): $[M+H]^+$ calcd. $C_{25}H_{27}NO_5Cl$ m/z 456.1572, found 456.1563.

IR (film, cm^{-1}): 3342, 2949, 2160, 1770, 1737, 1662, 1634, 1615, 1597, 1572, 1436, 1377, 1272, 1193, 1153, 1140, 1076, 1043, 1018, 998, 969, 903, 833.

$[\alpha]_D^{20}$ = +72.5 (c = 0.12, $CHCl_3$)

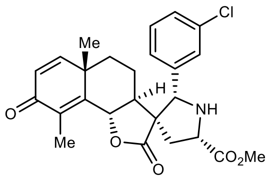

12i

Methyl (2'*S*,3*S*,3a*S*,5a*S*,5'*S*,9b*S*)-2'-(3-chlorophenyl)-5a,9-dimethyl-2,8-dioxo-3a,4,5,5a,8,9b-hexahydro-2*H*-spiro[naphtho[1,2-*b*]furan-3,3'-pyrrolidine]-5'-carboxylate

The title product compound **12i** was prepared in 0.10 mmol scale using **Condition D** and isolated by column chromatography (1:1 Cyclohexane: Ethyl acetate) giving an amorphous solid (27.9 mg, 0.06 mmol, 61% yield).

^{1}H NMR (700 MHz, $CDCl_3$) δ 7.68 (s, 1H), 7.41 – 7.32 (m, 3H), 6.53 (d, *J* = 9.9 Hz, 1H), 6.16 (d, *J* = 9.9 Hz, 1H), 4.59 (s, 1H), 4.24 (dd, *J* = 10.6, 5.9 Hz, 1H), 4.20 (d, *J* = 12.1 Hz, 1H), 3.82 (s, 3H), 3.01 (dd, *J* = 13.9, 10.6 Hz, 1H), 2.24 (dd, *J* = 13.9, 5.9 Hz, 1H), 2.07 (d, *J* = 1.4 Hz, 3H), 2.02 – 1.95 (m, 2H), 1.84 (qd, *J* = 13.3, 3.9 Hz, 1H), 1.69 (ddd, *J* = 13.7, 4.0, 2.3 Hz, 1H), 1.21 (td, *J* = 13.2, 4.5 Hz, 1H), 0.67 (s, 3H).

^{13}C NMR (176 MHz, $CDCl_3$) δ 186.3, 178.3, 172.9, 154.9, 151.0, 135.3, 130.4, 129.0, 128.7, 127.4, 126.0, 125.7, 79.7, 67.7, 56.7, 56.6, 54.9, 52.8, 41.2, 40.1, 38.5, 23.8, 18.2, 11.1.

HRMS(ESI): $[M+H]^+$ calcd. $C_{25}H_{27}NO_5Cl$ m/z 456.1572, found 456.1559.

IR (film, cm^{-1}): 2924, 1771, 1739, 1661, 1634, 1614, 1597, 1572, 1436, 1377, 1273, 1201, 1162, 1106, 1078, 1039, 988, 906.63, 832.

$[\alpha]_D^{20}$ = -97.6 (c = 0.13, $CHCl_3$)

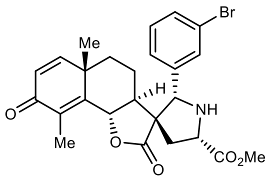

9j

Methyl (2'*S*,3*R*,3a*S*,5a*S*,5'*S*,9b*S*)-2'-(3-bromophenyl)-5a,9-dimethyl-2,8-dioxo-3a,4,5,5a,8,9b-hexahydro-2*H*-spiro[naphtho[1,2-*b*]furan-3,3'-pyrrolidine]-5'-carboxylate

The title product compound **9j** was prepared in 0.10 mmol scale using **Condition A** and isolated by column chromatography (20:1 DCM: MeOH) giving an amorphous solid (38.0 mg, 0.08 mmol, 76% yield).

^{1}H NMR (500 MHz, $CDCl_3$) δ 7.53 (s, 1H), 7.43 (d, *J* = 7.9 Hz, 1H), 7.32 (d, *J* = 7.8 Hz, 1H), 7.20 (t, *J* = 7.9 Hz, 1H), 6.68 (d, *J* = 9.9 Hz, 1H), 6.24 (d, *J* = 9.9 Hz, 1H), 4.79 (dd, *J* = 11.8, 1.4 Hz, 1H), 4.08 – 3.99 (m, 2H), 3.81 (s, 3H), 2.55 (dd, *J* = 13.4, 8.9 Hz, 1H), 2.44 (dd, *J* = 13.4, 5.7 Hz, 1H), 2.08 – 2.00 (m, 1H), 1.96 (ddd, *J* = 14.4, 4.1,

2.4 Hz, 2H), 1.92 (d, *J* = 1.4 Hz, 3H), 1.84 (qd, *J* = 13.1, 4.2 Hz, 1H), 1.55 (ddd, *J* = 13.5, 13.1, 4.6 Hz, 1H), 1.30 (s, 3H).

^{13}C NMR (126 MHz, CDCl$_3$) δ 186.2, 175.9, 172.4, 154.6, 150.4, 138.1, 131.8, 131.5, 130.2, 129.4, 126.8, 126.2, 122.7, 80.0, 67.9, 58.7, 55.0, 52.7, 50.6, 41.1, 37.5, 33.9, 25.2, 19.4, 10.9.

HRMS(ESI): $[M+H]^+$ calcd. $C_{25}H_{27}NO_5Br$ m/z 500.1067, found 500.1054.

IR (film, cm^{-1}): 3340, 2989, 2947, 2803, 2234, 2123, 1789, 1749, 1731, 1658, 1628, 1612, 1570, 1429, 1405, 1386, 1335, 1305, 1276, 1264, 1226, 1205, 1190, 1144, 1134, 1094, 1072, 1057, 1023, 1013, 988, 962, 949, 912, 902, 846.

$[\alpha]_D^{20}$ = -55.8 (c = 0.19, $CHCl_3$)

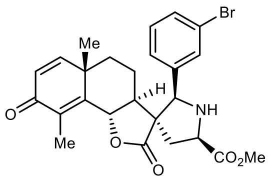

10j

Methyl (2'*R*,3*S*,3a*S*,5a*S*,5'*R*,9b*S*)-2'-(3-bromophenyl)-5a,9-dimethyl-2,8-dioxo-3a,4,5,5a,8,9b-hexahydro-2*H*-spiro[naphtho[1,2-*b*]furan-3,3'-pyrrolidine]-5'-carboxylate

The title product compound **10j** was prepared in 0.10 mmol scale using **Condition B** and isolated by column chromatography (20:1 DCM: MeOH) giving an amorphous solid (47.5 mg, 0.09 mmol, 95% yield).

^{1}H NMR (400 MHz, CDCl$_3$) δ 7.61 (s, 1H), 7.49 (d, *J* = 7.9 Hz, 1H), 7.45 (d, *J* = 8.0 Hz, 1H), 7.30 (t, *J* = 7.9 Hz, 1H), 6.63 (d, *J* = 9.9 Hz, 1H), 6.20 (d, *J* = 9.9 Hz, 1H), 4.35 (s, 1H), 4.24 (dd, *J* = 11.8, 1.4 Hz, 1H), 4.00 (dd, *J* = 9.7, 5.2 Hz, 1H), 3.85 (s, 3H), 2.67 (dd, *J* = 13.6, 5.2 Hz, 1H), 2.36 – 2.20 (m, 2H), 2.14 – 1.91 (m, 7H), 1.46 (td, *J* = 13.1, 4.8 Hz, 1H), 1.12 (s, 3H).

^{13}C NMR (101 MHz, CDCl$_3$) δ 186.2, 177.1, 173.3, 154.6, 150.7, 140.4, 132.5, 131.4, 131.3, 129.0, 126.1, 126.0, 123.1, 79.4, 66.0, 58.5, 56.9, 52.8, 52.6, 41.2, 38.6, 37.9, 24.7, 19.3, 11.0.

HRMS(ESI): $[M+H]^+$ calcd. $C_{25}H_{27}NO_5Br$ m/z 500.1067, found 500.1056.

IR (film, cm^{-1}): 2928, 2248, 1774, 1737, 1661, 1634, 1614, 1569, 1436, 1377, 1306, 1264, 1202, 1181, 1154, 1096, 1039, 995, 959, 905, 833.

$[\alpha]_D^{20}$ = -85.6 (c = 0.20, $CHCl_3$)

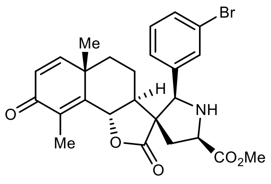

11j

Methyl (2'*R*,3*R*,3a*S*,5a*S*,5'*R*,9b*S*)-2'-(3-bromophenyl)-5a,9-dimethyl-2,8-dioxo-3a,4,5,5a,8,9b-hexahydro-2*H*-spiro[naphtho[1,2-*b*]furan-3,3'-pyrrolidine]-5'-carboxylate

The title product compound **11j** was prepared in 0.10 mmol scale using **Condition C** and isolated by column chromatography (1:1 Cyclohexane: Ethyl acetate) giving an amorphous solid (37.3 mg, 0.07 mmol, 75% yield).

^{1}H NMR (700 MHz, CDCl$_3$) δ 7.61 (s, 1H), 7.41 (d, *J* = 7.9 Hz, 1H), 7.25 (d, *J* = 7.9 Hz, 1H), 7.18 (t, *J* = 7.8 Hz, 1H), 6.58 (d, *J* = 9.8 Hz, 1H), 6.19 (d, *J* = 9.8 Hz, 1H), 4.78 (dd, *J* = 11.5, 1.5 Hz, 1H), 4.74 (s, 1H), 4.22 (dd, *J* = 8.9, 7.8 Hz, 1H), 3.83 (s, 3H), 2.52 (dd, *J* = 13.4, 7.8 Hz, 1H), 2.32 (dd, *J* = 13.4, 8.8 Hz, 1H), 2.05 (d, *J* = 1.4 Hz, 3H), 1.71 – 1.65 (m, 1H), 1.61 (td, *J* = 11.7, 11.1, 2.8 Hz, 1H), 1.56 (dd, *J* = 12.7, 3.8 Hz, 1H), 1.28 (dt, *J* = 5.0, 2.4 Hz, 1H), 1.23 (s, 3H), 1.10 (td, *J* = 13.0, 4.5 Hz, 1H).

^{13}C NMR (176 MHz, CDCl$_3$) δ 186.3, 178.2, 173.2, 154.8, 150.7, 141.4, 131.6, 130.4, 130.1, 129.3, 126.1, 126.0, 123.1, 80.0, 66.5, 57.2, 55.1, 52.6, 50.4, 41.1, 37.4, 31.1, 25.1, 20.2, 11.1.

HRMS(ESI): $[M+H]^+$ calcd. $C_{25}H_{27}NO_5Br$ m/z 500.1067, found 500.1061.

IR (film, cm^{-1}): 2946, 2248, 1771, 1736, 1661, 1634, 1614, 1568, 1436, 1376, 1272, 1193, 1153, 1140, 1043, 1018, 996, 968, 904, 832.

$[\alpha]_D^{20}$ = +73.2 (c = 0.24, $CHCl_3$)

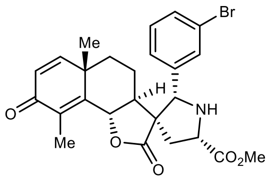

12j

Methyl (2'*S*,3*S*,3a*S*,5a*S*,5'*S*,9b*S*)-2'-(3-bromophenyl)-5a,9-dimethyl-2,8-dioxo-3a,4,5,5a,8,9b-hexahydro-2*H*-spiro[naphtho[1,2-*b*]furan-3,3'-pyrrolidine]-5'-carboxylate

The title product compound **12j** was prepared in 0.10 mmol scale using **Condition D** and isolated by column chromatography (1:1 Cyclohexane: Ethyl acetate) giving an amorphous solid (27.4 mg, 0.05 mmol, 55% yield).

^{1}H NMR (500 MHz, CDCl$_3$) δ 7.83 (s, 1H), 7.52 – 7.45 (m, 1H), 7.44 – 7.35 (m, 1H), 7.29 – 7.23 (m, 1H), 6.53 (d, *J* = 9.9 Hz, 1H), 6.15 (d, *J* = 9.9 Hz, 1H), 4.49 (s, 1H), 4.20 – 4.08 (m, 2H), 3.80 (s, 3H), 2.99 (dd, *J* = 13.9, 10.7 Hz, 1H), 2.18 (dd, *J* = 13.9, 5.5 Hz, 1H), 2.06 (d, *J* = 1.4 Hz, 3H), 2.03 – 1.83 (m, 3H), 1.69 (ddd, *J* = 13.5, 3.8, 2.3 Hz, 1H), 1.20 (td, *J* = 12.9, 4.7 Hz, 1H), 0.66 (s, 3H).

^{13}C NMR (126 MHz, CDCl$_3$) δ 186.4, 178.5, 173.5, 155.0, 151.2, 139.5, 131.7, 130.6, 130.2, 128.5, 126.1, 125.9, 123.2, 79.6, 67.8, 56.9, 56.5, 54.9, 52.6, 41.2, 40.2, 38.5, 23.7, 18.2, 11.1.

HRMS(ESI): $[M+H]^+$ calcd. $C_{25}H_{27}NO_5Br$ m/z 500.1067, found 500.1062.

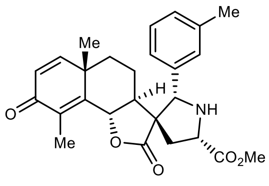

9k

Methyl (2'*S*,3*R*,3a*S*,5a*S*,5'*S*,9b*S*)-2'-(*m*-tolyl)-5a,9-dimethyl-2,8-dioxo-3a,4,5,5a,8,9b-hexahydro-2*H*-spiro[naphtho[1,2-*b*]furan-3,3'-pyrrolidine]-5'-carboxylate

The title product compound **9k** was prepared in 0.10 mmol scale using **Condition A** and isolated by column chromatography (20:1 DCM: MeOH) giving an amorphous solid (32.7 mg, 0.08 mmol, 75% yield).

^{1}H NMR (500 MHz, $CDCl_3$) δ 7.24 – 7.16 (m, 2H), 7.14 – 7.06 (m, 2H), 6.67 (d, *J* = 9.9 Hz, 1H), 6.23 (d, *J* = 9.9 Hz, 1H), 4.77 (dd, *J* = 11.8, 1.4 Hz, 1H), 4.12 – 4.01 (m, 2H), 3.82 (s, 3H), 2.54 (dd, *J* = 13.4, 9.1 Hz, 1H), 2.45 (dd, *J* = 13.4, 5.3 Hz, 1H), 2.33 (s, 3H), 2.07 (td, *J* = 12.3, 3.4 Hz, 1H), 2.00 – 1.91 (m, 2H), 1.90 (d, *J* = 1.4 Hz, 3H), 1.82 (ddd, *J* = 13.2, 12.7, 4.3 Hz, 1H), 1.53 (ddd, *J* = 13.6, 13.1, 4.6 Hz, 1H), 1.29 (s, 3H).

^{13}C NMR (126 MHz, $CDCl_3$) δ 186.3, 176.3, 172.6, 154.6, 150.7, 138.3, 135.2, 129.5, 129.2, 128.8, 128.5, 126.2, 125.3, 80.1, 68.7, 58.8, 55.0, 52.7, 50.1, 41.2, 37.6, 34.2, 25.1, 21.7, 19.5, 10.9.

HRMS(ESI): $[M+H]^+$ calcd. $C_{26}H_{30}NO_5$ m/z 436.2119, found 436.2114.

IR (film, cm^{-1}): 2935, 1766, 1738, 1660, 1633, 1613, 1430, 1381, 1319, 1270, 1190, 1158, 1135, 1112, 1092, 1057, 1031, 988, 966, 942, 902, 883, 856, 838.

$[\alpha]_D^{20}$ = -72.4 (c = 0.11, $CHCl_3$)

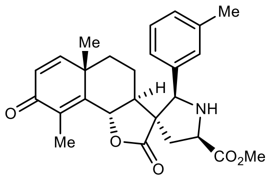

10k

Methyl (2'*R*,3*S*,3a*S*,5a*S*,5'*R*,9b*S*)-2'-(*m*-tolyl)-5a,9-dimethyl-2,8-dioxo-3a,4,5,5a,8,9b-hexahydro-2*H*-spiro[naphtho[1,2-*b*]furan-3,3'-pyrrolidine]-5'-carboxylate

The title product compound **10k** was prepared in 0.10 mmol scale using **Condition B** and isolated by column chromatography (20:1 DCM: MeOH) giving an amorphous solid (40.9 mg, 0.09 mmol, 94% yield).

^{1}H NMR (400 MHz, $CDCl_3$) δ 7.31 – 7.25 (m, 3H), 7.16 (d, *J* = 5.7 Hz, 1H), 6.61 (d, *J* = 9.9 Hz, 1H), 6.17 (d, *J* = 9.8 Hz, 1H), 4.31 (s, 1H), 4.15 (dd, *J* = 11.7, 1.5 Hz, 1H), 4.00 (dd, *J* = 10.0, 4.7 Hz, 1H), 3.84 (s, 3H), 2.63 (dd, *J* = 13.6, 4.7 Hz, 1H), 2.38 – 2.31 (m, 4H), 2.23 (td, *J* = 12.2, 3.3 Hz, 1H), 2.11 (qd, *J* = 12.9, 4.1 Hz, 1H), 2.02 – 1.88 (m, 5H), 1.48 – 1.37 (m, 1H), 1.04 (s, 3H).

^{13}C NMR (101 MHz, $CDCl_3$) δ 186.3, 177.8, 173.5, 154.6, 151.1, 139.2, 137.3, 130.2, 129.4, 128.7, 128.4, 126.1, 124.8, 79.4, 67.0, 58.4, 56.7, 52.7, 52.3, 41.2, 38.9, 37.9, 24.5, 21.6, 19.0, 11.0.

HRMS(ESI): $[M+H]^+$ calcd. $C_{26}H_{30}NO_5$ m/z 436.2119, found 436.2106.

IR (film, cm^{-1}): 2929, 1771, 1737, 1662, 1634, 1614, 1436, 1405, 1377, 1309, 1268, 1221, 1203, 1183, 1154, 1096, 1038, 991, 961, 907, 881, 833.

$[\alpha]_D^{20}$ = -70.0 (c = 0.16, $CHCl_3$)

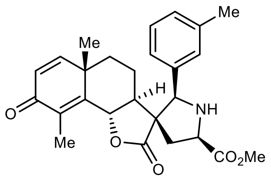

11k

Methyl (2'*R*,3*R*,3a*S*,5a*S*,5'*R*,9b*S*)-2'-(*m*-tolyl)-5a,9-dimethyl-2,8-dioxo-3a,4,5,5a,8,9b-hexahydro-2*H*-spiro[naphtho[1,2-*b*]furan-3,3'-pyrrolidine]-5'-carboxylate

The title product compound **11k** was prepared in 0.10 mmol scale using **Condition C** and isolated by column chromatography (1:1 Cyclohexane: Ethyl acetate) giving an amorphous solid (31.7 mg, 0.07 mmol, 73% yield).

^{1}H NMR (700 MHz, $CDCl_3$) δ 7.18 (t, *J* = 7.7 Hz, 1H), 7.15-7.12 (m, 2H), 7.06 (dd, *J* = 7.4, 1.8 Hz, 1H), 6.56 (d, *J* = 9.9 Hz, 1H), 6.16 (d, *J* = 9.8 Hz, 1H), 4.74 (dd, *J* = 11.7, 1.5 Hz, 1H), 4.72 (s, 1H), 4.20 (dd, *J* = 9.0, 7.7 Hz, 1H), 3.82 (s, 3H), 2.51 (dd, *J* = 13.4, 7.7 Hz, 1H), 2.33 – 2.27 (m, 4H), 2.02 (d, *J* = 1.4 Hz, 3H), 1.67 – 1.58 (m, 2H), 1.52 (qd, *J* = 12.8, 3.9 Hz, 1H), 1.34 – 1.30 (m, 1H), 1.21 (s, 3H), 1.05 (td, *J* = 13.2, 4.6 Hz, 1H).

^{13}C NMR (176 MHz, $CDCl_3$) δ 186.3, 178.6, 173.4, 154.9, 151.1, 138.6, 138.5, 129.1, 129.0, 128.6, 127.6, 126.0, 124.0, 79.9, 67.3, 57.2, 55.1, 52.5, 50.2, 41.1, 37.5, 31.4, 25.0, 21.6, 20.2, 11.0.

HRMS(ESI): $[M+H]^+$ calcd. $C_{26}H_{30}NO_5$ m/z 436.2119, found 436.2106.

IR (film, cm^{-1}): 2950, 1771, 1738, 1662, 1634, 1613, 1454, 1437, 1378, 1274, 1191, 1162, 1139, 1108, 1042, 1019, 991, 968, 908, 833.

$[\alpha]_D^{20}$ = +40.5 (c = 0.19, $CHCl_3$)

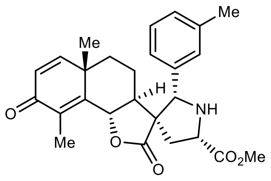

12k

Methyl (2'*S*,3*S*,3a*S*,5a*S*,5'*S*,9b*S*)-2'-(*m*-tolyl)-5a,9-dimethyl-2,8-dioxo-3a,4,5,5a,8,9b-hexahydro-2*H*-spiro[naphtho[1,2-*b*]furan-3,3'-pyrrolidine]-5'-carboxylate

The title product compound **12k** was prepared in 0.10 mmol scale using **Condition D** and isolated by column chromatography (1:1 Cyclohexane: Ethyl acetate) giving an amorphous solid (35.1 mg, 0.08 mmol, 81% yield).

^{1}H NMR (500 MHz, $CDCl_3$) δ 7.44 – 7.23 (m, 3H), 7.13 (d, *J* = 7.5 Hz, 1H), 6.51 (d, *J* = 9.8 Hz, 1H), 6.14 (d, *J* = 9.9 Hz, 1H), 4.48 (s, 1H), 4.21 – 4.06 (m, 2H), 3.80 (d, *J* = 1.4 Hz, 3H), 3.01 (ddd, *J* = 14.3, 10.7, 1.8 Hz, 1H), 2.36 (s, 3H), 2.13 (dd, *J* = 13.8, 5.5 Hz, 1H), 2.06 (s, 3H), 2.06 – 1.89 (m, 3H), 1.72 – 1.61 (m, 1H), 1.18 (td, *J* = 12.8, 4.7 Hz, 1H), 0.58 (s, 3H).

^{13}C NMR (126 MHz, $CDCl_3$) δ 186.5, 178.9, 173.8, 155.1, 151.6, 138.7, 136.6, 129.3, 128.9, 128.3, 127.6, 125.9, 124.3, 79.6, 68.5, 57.2, 56.6, 55.0, 52.5, 41.2, 40.8, 38.6, 23.6, 21.6, 18.0, 11.1.

HRMS(ESI): $[M+H]^+$ calcd. $C_{26}H_{30}NO_5$ m/z 436.2119, found 436.2115.

IR (film, cm^{-1}): 3325, 2919, 2850, 2363, 2160, 2033, 1770, 1736, 1662, 1635, 1613, 1489, 1437, 1378, 1308, 1271, 1186, 1162, 1106, 1039, 989, 938, 899, 832, 805.

$[\alpha]_D^{20}$ = -56.7 (c = 0.09, $CHCl_3$)

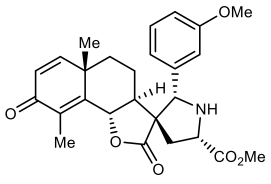

9l

Methyl (2'*S*,3*R*,3a*S*,5a*S*,5'*S*,9b*S*)-2'-(3-methoxyphenyl)-5a,9-dimethyl-2,8-dioxo-3a,4,5,5a,8,9b-hexahydro-2*H*-spiro[naphtho[1,2-*b*]furan-3,3'-pyrrolidine]-5'-carboxylate

The title product compound **9l** was prepared in 0.10 mmol scale using **Condition A** and isolated by column chromatography (20:1 DCM: MeOH) giving an amorphous solid (22.6 mg, 0.05 mmol, 50% yield).

^{1}H NMR (500 MHz, $CDCl_3$) δ 7.23 (t, *J* = 7.9 Hz, 1H), 6.97 (s, 1H), 6.90 (d, *J* = 7.7 Hz, 1H), 6.83 (d, *J* = 8.2 Hz, 1H), 6.67 (d, *J* = 9.9 Hz, 1H), 6.23 (d, *J* = 9.8 Hz, 1H), 4.77 (dd, *J* = 11.8, 1.6 Hz, 1H), 4.09 – 4.01 (m, 2H), 3.81 (s, 3H), 3.79 (s, 3H), 2.53 (dd, *J* = 13.5, 9.1 Hz, 1H), 2.46 (dd, *J* = 13.4, 5.1 Hz, 1H), 2.08 (td, *J* = 12.3, 3.5 Hz, 1H), 2.00 – 1.92 (m, 2H), 1.90 (s, 3H), 1.82 (qd, *J* = 13.1, 4.3 Hz, 1H), 1.53 (td, *J* = 13.1, 4.7 Hz, 1H), 1.29 (s, 3H).

^{13}C NMR (126 MHz, $CDCl_3$) δ 186.3, 176.2, 172.7, 159.7, 154.6, 150.7, 136.9, 129.6, 129.2, 126.2, 120.5, 114.3, 113.8, 80.1, 68.6, 58.8, 55.4, 55.1, 52.7, 50.1, 41.2, 37.6, 34.0, 25.1, 19.5, 10.9.

HRMS(ESI): $[M+H]^+$ calcd. $C_{26}H_{30}NO_6$ m/z 452.2068, found 452.2054.

IR (film, cm^{-1}): 2951, 1768, 1739, 1660, 1633, 1612, 1585, 1489, 1456, 1432, 1381, 1275, 1267, 1261, 1202, 1190, 1156, 1135, 1111, 1091, 1031, 988, 965, 940, 902, 882, 856, 837.

$[\alpha]_D^{20}$ = -81.5 (c = 0.14, $CHCl_3$)

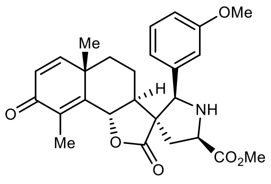

10l

Methyl (2'*R*,3*S*,3a*S*,5a*S*,5'*R*,9b*S*)-2'-(3-methoxyphenyl)-5a,9-dimethyl-2,8-dioxo-3a,4,5,5a,8,9b-hexahydro-2*H*-spiro[naphtho[1,2-*b*]furan-3,3'-pyrrolidine]-5'-carboxylate

The title product compound **10l** was prepared in 0.10 mmol scale using **Condition B** and isolated by column chromatography (20:1 DCM: MeOH) giving an amorphous solid (42.9 mg, 0.10 mmol, 95% yield).

^{1}H NMR (400 MHz, $CDCl_3$) δ 7.30 (t, *J* = 8.2 Hz, 1H), 7.07 – 7.02 (m, 2H), 6.88 (d, *J* = 8.3 Hz, 1H), 6.61 (d, *J* = 9.9 Hz, 1H), 6.18 (d, *J* = 9.8 Hz, 1H), 4.33 (s, 1H), 4.25 – 4.18 (m, 1H), 4.00 (dd, *J* = 9.9, 4.8 Hz, 1H), 3.84 (s, 3H), 3.80 (s, 3H), 2.64 (dd, *J* = 13.6, 4.8 Hz, 1H), 2.32 (dd, *J* = 13.6, 9.8 Hz, 1H), 2.23 (td, *J* = 12.2, 3.4 Hz, 1H), 2.16 – 1.88 (m, 7H), 1.44 (dt, *J* = 13.1, 6.7 Hz, 1H), 1.06 (s, 3H).

^{13}C NMR (101 MHz, CDCl$_3$) δ 186.3, 177.8, 173.5, 160.4, 154.6, 151.1, 139.2, 130.5, 128.8, 126.1, 120.0, 114.9, 113.2, 79.4, 66.9, 58.5, 56.7, 55.4, 52.7, 52.4, 41.2, 38.8, 37.9, 24.7, 19.0, 11.0.

HRMS(ESI): $[M+H]^+$ calcd. $C_{26}H_{30}NO_6$ m/z 452.2068, found 452.2054.

IR (film, cm^{-1}): 2935, 1772, 1737, 1661, 1634, 1611, 1585, 1489, 1455, 1437, 1377, 1267, 1223, 1202, 1181, 1154, 1096, 1037, 992, 961, 907, 880, 833.

$[\alpha]_D^{20}$ = -71.1 (c = 0.18, CHCl$_3$)

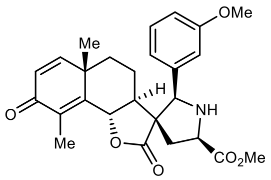

11l

Methyl (2'*R*,3*R*,3a*S*,5a*S*,5'*R*,9b*S*)-2'-(3-methoxyphenyl)-5a,9-dimethyl-2,8-dioxo-3a,4,5,5a,8,9b-hexahydro-2*H*-spiro[naphtho[1,2-*b*]furan-3,3'-pyrrolidine]-5'-carboxylate

The title product compound **11l** was prepared in 0.10 mmol scale using **Condition C** and isolated by column chromatography (1:1 Cyclohexane: Ethyl acetate) giving an amorphous solid (28.2 mg, 0.06 mmol, 63% yield).

^{1}H NMR (500 MHz, CDCl$_3$) δ 7.21 (t, *J* = 7.9 Hz, 1H), 6.96 (s, 1H), 6.90 (d, *J* = 7.8 Hz, 1H), 6.79 (d, *J* = 8.2 Hz, 1H), 6.56 (d, *J* = 9.8 Hz, 1H), 6.17 (d, *J* = 9.8 Hz, 1H), 4.75 (dd, *J* = 11.8, 1.4 Hz, 1H), 4.72 (s, 1H), 4.21 (dd, *J* = 8.8, 7.8 Hz, 1H), 3.81 (s, 3H), 3.76 (s, 3H), 2.51 (dd, *J* = 13.4, 7.8 Hz, 1H), 2.30 (dd, *J* = 13.4, 8.9 Hz, 1H), 2.03 (s, 3H), 1.70 – 1.61 (m, 2H), 1.52 (qd, *J* = 12.8, 3.8 Hz, 1H), 1.31 – 1.25 (m, 1H), 1.21 (s, 3H), 1.08 (td, *J* = 13.1, 4.5 Hz, 1H).

^{13}C NMR (126 MHz, CDCl$_3$) δ 186.3, 178.6, 173.4, 159.9, 154.9, 151.0, 140.6, 129.8, 129.0, 126.0, 119.3, 113.7, 112.5, 79.9, 67.0, 57.2, 55.4, 55.1, 52.5, 50.2, 41.1, 37.5, 31.2, 25.0, 20.2, 11.0.

HRMS(ESI): $[M+H]^+$ calcd. $C_{26}H_{30}NO_6$ m/z 452.2068, found 452.2055.

IR (film, cm^{-1}): 3339, 2949, 2526, 2160, 2024, 1770, 1738, 1661, 1634, 1611, 1584, 1488, 1454, 1437, 1378, 1314, 1274, 1192, 1157, 1044, 1018, 995, 969, 947, 902, 833.

$[\alpha]_D^{20}$ = +57.2 (c = 0.22, CHCl$_3$)

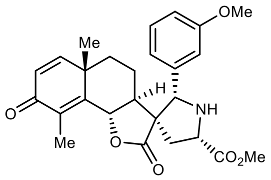

12l

Methyl (2'*S*,3*S*,3a*S*,5a*S*,5'*S*,9b*S*)-2'-(3-methoxyphenyl)-5a,9-dimethyl-2,8-dioxo-3a,4,5,5a,8,9b-hexahydro-2*H*-spiro[naphtho[1,2-*b*]furan-3,3'-pyrrolidine]-5'-carboxylate

The title product compound **12l** was prepared in 0.10 mmol scale using **Condition D** and isolated by column chromatography (1:1 Cyclohexane: Ethyl acetate) giving an amorphous solid (22.2 mg, 0.05 mmol, 49% yield).

^{1}H NMR (500 MHz, CDCl$_3$) δ 7.30 (t, *J* = 8.0 Hz, 1H), 7.17-7.07 (m, 2H), 6.86 (d, *J* = 8.2 Hz, 1H), 6.52 (d, *J* = 9.9 Hz, 1H), 6.15 (d, *J* = 9.9 Hz, 1H), 4.49 (s, 1H), 4.20 (dd, *J* = 11.4, 1.6 Hz, 1H), 4.14 (dd, *J* = 10.8, 5.5 Hz, 1H), 3.81 (s, 3H), 3.80 (s, 3H), 3.01 (dd, *J* = 13.9, 10.8 Hz, 1H), 2.15 (dd, *J* = 13.9, 5.6 Hz, 1H), 2.10 – 1.91 (m, 6H), 1.67 (ddd, *J* = 13.6, 3.9, 2.3 Hz, 1H), 1.19 (td, *J* = 13.0, 4.6 Hz, 1H), 0.63 (s, 3H).

^{13}C NMR (126 MHz, CDCl$_3$) δ 186.5, 178.9, 173.7, 160.0, 155.1, 151.6, 138.4, 130.1, 128.4, 125.9, 119.4, 113.9, 113.0, 79.7, 68.4, 57.1, 56.6, 55.6, 55.0, 52.5, 41.3, 40.6, 38.6, 23.9, 18.2, 11.1.

HRMS(ESI): [M+H]$^+$ calcd. $C_{26}H_{30}NO_6Cl$ m/z 452.2068, found 452.2064.

IR (film, cm^{-1}): 2923, 2851, 2161, 1770, 1737, 1661, 1634, 1610, 1584, 1491, 1454, 1437, 1378, 1272, 1187, 1160, 1107, 1039, 989, 960, 898, 832.

$[\alpha]_D^{20}$ = -66.7 (c = 0.15, CHCl$_3$)

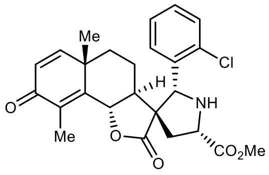

9m

Methyl (2'*S*,3*R*,3a*S*,5a*S*,5'*S*,9b*S*)-2'-(2-chlorophenyl)-5a,9-dimethyl-2,8-dioxo-3a,4,5,5a,8,9b-hexahydro-2*H*-spiro[naphtho[1,2-*b*]furan-3,3'-pyrrolidine]-5'-carboxylate

The title product compound **9m** was prepared in 0.10 mmol scale using **Condition A** and isolated by column chromatography (20:1 DCM: MeOH) giving an amorphous solid (34.5 mg, 0.08 mmol, 76% yield).

^{1}H NMR (600 MHz, CDCl$_3$) δ 7.88 (d, *J* = 7.9 Hz, 1H), 7.33 – 7.28 (m, 2H), 7.22 (t, *J* = 7.6 Hz, 1H), 6.66 (d, *J* = 9.9 Hz, 1H), 6.21 (d, *J* = 9.9 Hz, 1H), 4.81 – 4.69 (m, 2H), 4.11 (dd, *J* = 9.1, 5.7 Hz, 1H), 3.81 (s, 3H), 2.57 (dd, *J* = 13.5, 9.1 Hz, 1H), 2.43 (dd, *J* = 13.4, 5.7 Hz, 1H), 2.19 (td, *J* = 12.2, 3.4 Hz, 1H), 2.10 (ddt, *J* = 10.4, 4.3, 2.3 Hz, 1H), 1.95 – 1.88 (m, 4H), 1.82 (qd, *J* = 12.7, 3.9 Hz, 1H), 1.51 (td, *J* = 13.2, 4.5 Hz, 1H), 1.28 (s, 3H).

^{13}C NMR (151 MHz, CDCl$_3$) δ 186.3, 175.8, 172.8, 154.7, 150.8, 134.0, 133.9, 130.2, 129.6, 129.1, 127.4, 126.1, 79.9, 63.6, 58.6, 55.6, 52.7, 50.5, 41.2, 37.6, 33.7, 25.2, 19.9, 10.8.

HRMS(ESI): [M+H]$^+$ calcd. $C_{25}H_{27}NO_5Cl$ m/z 456.1572, found 456.1565.

IR (film, cm^{-1}): 3387, 2925, 2160, 1775, 1719, 1658, 1630, 1609, 1439, 1401, 1383, 1327, 1303, 1289, 1266, 1225, 1202, 1183, 1164, 1150, 1117, 1093, 1076, 1026, 989, 959, 902, 835.

$[\alpha]_D^{20}$ = -67.5 (c = 0.12, CHCl$_3$)

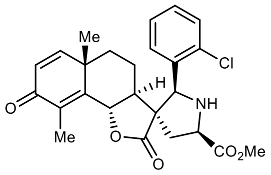

10m

Methyl (2'*R*,3*S*,3a*S*,5a*S*,5'*R*,9b*S*)-2'-(2-chlorophenyl)-5a,9-dimethyl-2,8-dioxo-3a,4,5,5a,8,9b-hexahydro-2*H*-spiro[naphtho[1,2-*b*]furan-3,3'-pyrrolidine]-5'-carboxylate

The title product compound **10m** was prepared in 0.10 mmol scale using **Condition B** and isolated by column chromatography (20:1 DCM: MeOH) giving an amorphous solid (43.3 mg, 0.09 mmol, 95% yield).

^{1}H NMR (400 MHz, CDCl$_3$) δ 7.73 (d, *J* = 8.2 Hz, 1H), 7.36 (dd, *J* = 7.4, 6.4 Hz, 2H), 7.30 – 7.24 (m, 1H), 6.66 (d, *J* = 9.9 Hz, 1H), 6.22 (d, *J* = 9.8 Hz, 1H), 5.07 (s, 1H), 4.77 (dd, *J* = 11.6, 1.5 Hz, 1H), 4.01 (t, *J* = 8.2 Hz, 1H), 3.83 (s, 3H), 2.75 (dd, *J* = 13.3, 8.5 Hz, 1H), 2.37 – 2.14 (m, 3H), 2.09 – 1.92 (m, 5H), 1.56 – 1.46 (m, 1H), 1.25 (s, 3H).

^{13}C NMR (101 MHz, CDCl$_3$) δ 186.3, 176.5, 173.4, 154.9, 151.0, 136.6, 132.5, 130.0, 129.8, 129.1, 128.2, 126.1, 79.4, 60.6, 59.0, 57.6, 54.9, 52.7, 41.3, 39.3, 37.9, 24.9, 20.1, 11.1.

HRMS(ESI): $[M+H]^+$ calcd. $C_{25}H_{27}NO_5Cl$ m/z 456.1572, found 456.1557.

IR (film, cm^{-1}): 2929, 1774, 1737, 1662, 1634, 1614, 1438, 1405, 1377, 1304, 1265, 1226, 1202, 1179, 1163, 1111, 1042, 994, 959, 903, 833.

$[\alpha]_D^{20}$ = -66.7 (c = 0.23, $CHCl_3$)

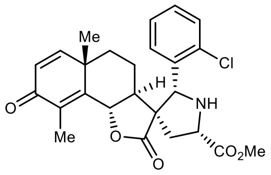

12m

Methyl (2'*S*,3*S*,3a*S*,5a*S*,5'*S*,9b*S*)-2'-(2-chlorophenyl)-5a,9-dimethyl-2,8-dioxo-3a,4,5,5a,8,9b-hexahydro-2*H*-spiro[naphtho[1,2-*b*]furan-3,3'-pyrrolidine]-5'-carboxylate

The title product compound **12m** was prepared in 0.10 mmol scale using **Condition D** and isolated by column chromatography (20:1 DCM: MeOH) giving an amorphous solid (29.4 mg, 0.06 mmol, 64% yield).

^{1}H NMR (500 MHz, CDCl$_3$) δ 8.03 (d, *J* = 7.9 Hz, 1H), 7.40 (d, *J* = 7.8 Hz, 1H), 7.34 (t, *J* = 7.6 Hz, 1H), 7.29 (t, *J* = 7.6 Hz, 1H), 6.51 (d, *J* = 9.9 Hz, 1H), 6.16 (d, *J* = 9.9 Hz, 1H), 4.97 (s, 1H), 4.77 (dd, *J* = 12.2, 1.7 Hz, 1H), 4.31 (t, *J* = 8.6 Hz, 1H), 3.80 (s, 3H), 2.73 (dd, *J* = 13.4, 8.3 Hz, 1H), 2.30 (dd, *J* = 13.5, 8.8 Hz, 1H), 2.11 (s, 3H), 1.99 (td, *J* = 12.7, 3.6 Hz, 1H), 1.66 – 1.57 (m, 2H), 1.36 (dd, *J* = 13.2, 3.8 Hz, 1H), 1.15 (td, *J* = 13.0, 4.1 Hz, 1H), 0.73 (s, 3H).

^{13}C NMR (126 MHz, CDCl$_3$) δ 186.4, 179.2, 173.3, 155.0, 151.3, 137.0, 133.5, 131.1, 130.0, 129.8, 129.1, 127.2, 125.9, 80.1, 63.1, 57.0, 55.9, 53.9, 52.5, 41.3, 39.3, 38.3, 23.9, 18.0, 11.4.

HRMS(ESI): $[M+H]^+$ calcd. $C_{25}H_{27}NO_5Cl$ m/z 456.1572, found 456.1563.

IR (film, cm^{-1}): 2925, 2853, 1773, 1739, 1661, 1633, 1614, 1438, 1404, 1378, 1274, 1202, 1185, 1157, 1106, 1035, 990, 954, 907, 832.

$[\alpha]_D^{20}$ = -28.7 (c = 0.36, $CHCl_3$)

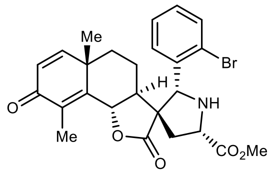

9n

Methyl (2'*S*,3*R*,3a*S*,5a*S*,5'*S*,9b*S*)-2'-(2-bromophenyl)-5a,9-dimethyl-2,8-dioxo-3a,4,5,5a,8,9b-hexahydro-2*H*-spiro[naphtho[1,2-*b*]furan-3,3'-pyrrolidine]-5'-carboxylate

The title product compound **9n** was prepared in 0.10 mmol scale using **Condition A** and isolated by column chromatography (20:1 DCM: MeOH) giving an amorphous solid (38.2 mg, 0.08 mmol, 76% yield).

^{1}H NMR (600 MHz, $CDCl_3$) δ 7.89 (d, *J* = 8.0 Hz, 1H), 7.51 (d, *J* = 8.0 Hz, 1H), 7.36 (t, *J* = 7.6 Hz, 1H), 7.16 (dd, *J* = 7.9, 7.3 Hz, 1H), 6.66 (d, *J* = 9.9 Hz, 1H), 6.22 (d, *J* = 9.9 Hz, 1H), 4.82 – 4.76 (m, 1H), 4.75 (s, 1H), 4.12 (dd, *J* = 9.0, 5.7 Hz, 1H), 3.82 (s, 3H), 2.56 (dd, *J* = 13.5, 9.1 Hz, 1H), 2.44 (dd, *J* = 13.4, 5.7 Hz, 1H), 2.25 (td, *J* = 12.1, 3.3 Hz, 1H), 2.21 – 2.14 (m, 1H), 1.97 – 1.88 (m, 4H), 1.82 (qd, *J* = 12.7, 3.9 Hz, 1H), 1.53 (td, *J* = 13.2, 4.5 Hz, 1H), 1.29 (s, 3H).

^{13}C NMR (151 MHz, $CDCl_3$) δ 186.3, 175.8, 172.9, 154.7, 150.9, 135.7, 132.9, 130.6, 130.0, 129.1, 128.0, 126.1, 125.0, 79.9, 66.3, 58.7, 55.7, 52.7, 50.6, 41.2, 37.6, 33.7, 25.2, 20.2, 10.8.

HRMS(ESI): $[M+H]^+$ calcd. $C_{25}H_{27}NO_5Br$ m/z 500.1067, found 500.1055.

IR (film, cm^{-1}): 3386, 2940, 1775, 1719, 1658, 1630, 1610, 1439, 1378, 1304, 1289, 1266, 1225, 1202, 1183, 1164, 1140, 1075, 1023, 989, 959, 902, 835.

$[\alpha]_D^{20}$ = -48.4 (c = 0.23, $CHCl_3$)

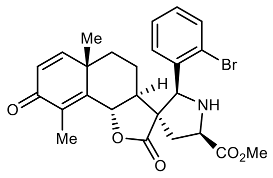

10n

Methyl (2'*R*,3*S*,3a*S*,5a*S*,5'*R*,9b*S*)-2'-(2-bromophenyl)-5a,9-dimethyl-2,8-dioxo-3a,4,5,5a,8,9b-hexahydro-2*H*-spiro[naphtho[1,2-*b*]furan-3,3'-pyrrolidine]-5'-carboxylate

The title product compound **10n** was prepared in 0.10 mmol scale using **Condition B** and isolated by column chromatography (20:1 DCM: MeOH) giving an amorphous solid (44.3 mg, 0.09 mmol, 89% yield).

^{1}H NMR (400 MHz, $CDCl_3$) δ 7.71 (d, *J* = 8.0 Hz, 1H), 7.55 (d, *J* = 8.1 Hz, 1H), 7.39 (t, *J* = 7.6 Hz, 1H), 7.22 – 7.14 (m, 1H), 6.66 (d, *J* = 9.9 Hz, 1H), 6.21 (d, *J* = 9.9 Hz, 1H), 5.03 (s, 1H), 4.87 (dd, *J* = 11.7, 1.5 Hz, 1H), 4.01 (t, *J* = 8.3 Hz, 1H), 3.82 (s, 3H), 2.77 (dd, *J* = 13.3, 8.8 Hz, 1H), 2.41 – 2.28 (m, 1H), 2.28 – 2.11 (m, 2H), 2.06 – 1.93 (m, 5H), 1.50 (td, *J* = 13.1, 12.7, 4.6 Hz, 1H), 1.26 (s, 3H).

^{13}C NMR (101 MHz, CDCl$_3$) δ 186.3, 176.3, 173.4, 154.8, 151.0, 138.2, 133.2, 130.4, 130.3, 129.1, 128.7, 126.0, 123.2, 79.4, 63.5, 59.0, 57.7, 55.1, 52.7, 41.3, 39.4, 37.9, 25.0, 20.4, 11.1.

HRMS(ESI): $[M+H]^+$ calcd. $C_{25}H_{27}NO_5Br$ m/z 500.1067, found 500.1060.

IR (film, cm^{-1}): 2930, 1773.74, 1736, 1661.78, 1633.70, 1614, 1567, 1436, 1405, 1376, 1304.72, 1274, 1263, 1225.68, 1201, 1179, 1163, 1113, 1042.71, 994, 959, 902, 832.67.

$[\alpha]_D^{20}$ = -63.5 (c = 0.26, $CHCl_3$)

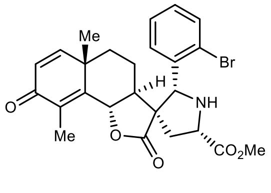

12n

Methyl (2'*S*,3*S*,3a*S*,5a*S*,5'*S*,9b*S*)-2'-(2-bromophenyl)-5a,9-dimethyl-2,8-dioxo-3a,4,5,5a,8,9b-hexahydro-2*H*-spiro[naphtho[1,2-*b*]furan-3,3'-pyrrolidine]-5'-carboxylate

The title product compound **12n** was prepared in 0.10 mmol scale using **Condition D** and isolated by column chromatography (20:1 DCM: MeOH) giving an amorphous solid (30.3 mg, 0.06 mmol, 61% yield).

^{1}H NMR (400 MHz, CDCl$_3$) δ 8.01 (d, *J* = 7.9 Hz, 1H), 7.58 (d, *J* = 8.0 Hz, 1H), 7.38 (dd, *J* = 8.5, 7.2 Hz, 1H), 7.21 (dd, *J* = 8.0, 7.3 Hz, 1H), 6.51 (d, *J* = 9.9 Hz, 1H), 6.16 (d, *J* = 9.9 Hz, 1H), 5.00 (s, 1H), 4.96 (dd, *J* = 12.2, 1.5 Hz, 1H), 4.40 (dd, *J* = 9.4, 7.7 Hz, 1H), 3.80 (s, 3H), 2.61 (dd, *J* = 13.3, 7.7 Hz, 1H), 2.43 – 2.31 (m, 1H), 2.12 (s, 3H), 2.06 – 1.95 (m, 1H), 1.57 (dd, *J* = 9.4, 2.3 Hz, 1H), 1.51 (ddd, *J* = 9.5, 3.8, 3.0 Hz, 1H), 1.19 – 1.09 (m, 2H), 0.81 (s, 3H).

^{13}C NMR (101 MHz, CDCl$_3$) δ 186.4, 179.2, 173.3, 155.0, 151.4, 139.3, 133.3, 131.8, 130.2, 129.2, 127.9, 126.0, 123.8, 80.3, 65.0, 57.1, 55.1, 54.3, 52.5, 41.3, 38.8, 38.3, 24.1, 18.2, 11.4.

HRMS(ESI): $[M+H]^+$ calcd. $C_{25}H_{27}NO_5Br$ m/z 500.1067, found 500.1057.

IR (film, cm^{-1}): 2927, 1772, 1739, 1661, 1633, 1614, 1437, 1404, 1378, 1275, 1202, 1184, 1156, 1106, 1038, 991, 950, 906, 879, 832.

$[\alpha]_D^{20}$ = -24.0 (c = 0.13, $CHCl_3$)

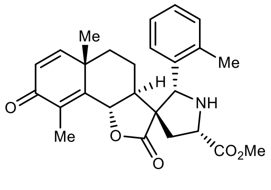

9o

Methyl (2'*S*,3*R*,3a*S*,5a*S*,5'*S*,9b*S*)-2'-(*o*-tolyl)-5a,9-dimethyl-2,8-dioxo-3a,4,5,5a,8,9b-hexahydro-2*H*-spiro[naphtho[1,2-*b*]furan-3,3'-pyrrolidine]-5'-carboxylate

The title product compound **9o** was prepared in 0.10 mmol scale using **Condition A** and isolated by column chromatography (20:1 DCM: MeOH) giving an amorphous solid (28.8 mg, 0.07 mmol, 66% yield).

^{1}H NMR (500 MHz, CDCl$_3$) δ 7.67 (d, *J* = 7.7 Hz, 1H), 7.25 – 7.11 (m, 3H), 6.65 (d, *J* = 9.9 Hz, 1H), 6.21 (d, *J* = 9.8 Hz, 1H), 4.77 (dd, *J* = 11.5, 1.5 Hz, 1H), 4.42 (s, 1H), 4.14 (dd, *J* = 8.4, 5.5 Hz, 1H), 3.82 (s, 3H), 2.56 – 2.46 (m, 2H), 2.26 (s, 3H), 2.01 – 1.89 (m, 6H), 1.88 – 1.78 (m, 1H), 1.45 (td, *J* = 13.3, 4.6 Hz, 1H), 1.29 (s, 3H).

^{13}C NMR (126 MHz, CDCl$_3$) δ 186.2, 176.5, 172.9, 154.5, 150.7, 136.6, 133.7, 130.9, 129.1, 128.3, 127.6, 126.7, 126.1, 80.1, 77.4, 77.2, 76.9, 63.9, 58.7, 55.2, 52.7, 49.7, 41.1, 37.6, 34.2, 25.2, 20.2, 20.2, 10.8.

HRMS(ESI): [M+H]$^+$ calcd. $C_{26}H_{30}NO_5$ m/z 436.2119, found 436.2108.

IR (film, cm^{-1}): 2933, 2118, 1771, 1737, 1661, 1634, 1615, 1436, 1378, 1307, 1268, 1203, 1184, 1165, 1153, 1141, 110, 1037, 994, 960, 907, 832.

$[\alpha]_D^{20}$ = -60.0 (c = 0.27, CHCl$_3$)

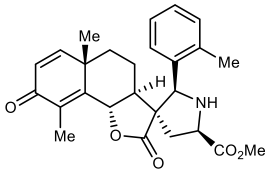

10o

Methyl (2'*R*,3*S*,3a*S*,5a*S*,5'*R*,9b*S*)-2'-(*o*-tolyl)-5a,9-dimethyl-2,8-dioxo-3a,4,5,5a,8,9b-hexahydro-2*H*-spiro[naphtho[1,2-*b*]furan-3,3'-pyrrolidine]-5'-carboxylate

The title product compound **10o** was prepared in 0.10 mmol scale using **Condition B** and isolated by column chromatography (20:1 DCM: MeOH) giving an amorphous solid (40.2 mg, 0.09 mmol, 92% yield).

^{1}H NMR (400 MHz, CDCl$_3$) δ 7.53 (dd, *J* = 7.4, 1.6 Hz, 1H), 7.29 – 7.17 (m, 3H), 6.60 (d, *J* = 9.9 Hz, 1H), 6.19 (d, *J* = 9.9 Hz, 1H), 4.57 (s, 1H), 4.30 (dd, *J* = 11.9, 1.5 Hz, 1H), 4.01 (dd, *J* = 9.4, 5.8 Hz, 1H), 3.84 (s, 3H), 2.68 (dd, *J* = 13.4, 5.8 Hz, 1H), 2.49 (s, 3H), 2.36 – 2.19 (m, 2H), 2.03 (s, 3H), 2.02 – 1.82 (m, 3H), 1.51-1.40 (m, 1H), 1.00 (s, 3H).

^{13}C NMR (101 MHz, CDCl$_3$) δ 186.2, 178.1, 173.4, 154.6, 150.8, 135.9, 135.4, 131.3, 129.0, 128.8, 127.6, 126.8, 126.1, 79.5, 61.8, 58.7, 56.7, 53.0, 52.7, 41.1, 39.6, 37.9, 24.6, 19.9, 19.7, 11.1.

HRMS(ESI): [M+H]$^+$ calcd. $C_{26}H_{30}NO_5$ m/z 436.2119, found 436.2108.

IR (film, cm^{-1}): 2933, 2250, 1770, 1736, 1662, 1634, 1614, 1492, 1437, 1404, 1377, 1303, 1264, 1202, 1179, 1154, 1112, 1037, 992, 959, 905, 832.

$[\alpha]_D^{20}$ = -80.0 (c = 0.21, CHCl$_3$)

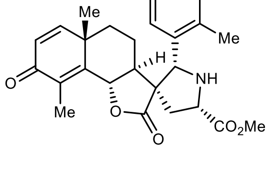

12o

Methyl (2'*S*,3*S*,3a*S*,5a*S*,5'*S*,9b*S*)-2'-(*o*-tolyl)-5a,9-dimethyl-2,8-dioxo-3a,4,5,5a,8,9b-hexahydro-2*H*-spiro[naphtho[1,2-*b*]furan-3,3'-pyrrolidine]-5'-carboxylate

The title product compound **12o** was prepared in 0.10 mmol scale using **Condition D** and isolated by column chromatography (20:1 DCM: MeOH) giving an amorphous solid (38.5 mg, 0.09 mmol, 88% yield).

^{1}H NMR (400 MHz, $CDCl_3$) δ 8.08 – 7.97 (m, 1H), 7.26 – 7.18 (m, 3H), 6.51 (d, *J* = 9.9 Hz, 1H), 6.14 (d, *J* = 9.9 Hz, 1H), 4.76 (s, 1H), 4.29 (dd, *J* = 11.6, 1.5 Hz, 1H), 4.17 (dd, *J* = 9.6, 7.3 Hz, 1H), 3.80 (s, 3H), 2.94 (dd, *J* = 13.7, 9.6 Hz, 1H), 2.39 (s, 3H), 2.23 (dd, *J* = 13.7, 7.4 Hz, 1H), 2.16 – 2.03 (m, 4H), 1.99-1.89 (m, 2H), 1.66 (d, *J* = 13.4 Hz, 1H), 1.17 (td, *J* = 13.0, 4.0 Hz, 1H), 0.57 (s, 3H).

^{13}C NMR (101 MHz, $CDCl_3$) δ 186.4, 179.4, 173.0, 155.2, 151.3, 136.9, 134.8, 131.7, 128.9, 128.9, 128.6, 125.9, 125.9, 79.9, 64.9, 57.3, 56.8, 53.5, 52.6, 41.3, 39.8, 38.4, 23.8, 19.6, 17.9, 11.3.

HRMS(ESI): $[M+H]^+$ calcd. $C_{26}H_{30}NO_5$ m/z 436.2119, found 436.2106.

IR (film, cm^{-1}): 1775, 1732, 1662, 1432, 1373, 1244, 1154, 1098, 1079, 1030, 984, 954, 890.

$[\alpha]_D^{20}$ = -52.1 (c = 0.28, $CHCl_3$)

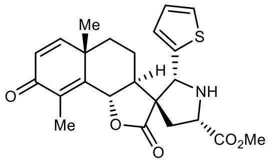

9p

Methyl (2'*S*,3*R*,3a*S*,5a*S*,5'*S*,9b*S*)-2'-(thiophen-2-yl)-5a,9-dimethyl-2,8-dioxo-3a,4,5,5a,8,9b-hexahydro-2*H*-spiro[naphtho[1,2-*b*]furan-3,3'-pyrrolidine]-5'-carboxylate

The title product compound **9p** was prepared in 0.10 mmol scale using **Condition A** and isolated by column chromatography (20:1 DCM: MeOH) giving an amorphous solid (19.6 mg, 0.05 mmol, 46% yield).

^{1}H NMR (600 MHz, $CDCl_3$) δ 7.24 (d, *J* = 5.1 Hz, 1H), 7.08 (d, *J* = 3.6 Hz, 1H), 6.98 (dd, *J* = 5.1, 3.6 Hz, 1H), 6.67 (d, *J* = 9.9 Hz, 1H), 6.24 (d, *J* = 9.9 Hz, 1H), 4.81 (dd, *J* = 11.9, 1.5 Hz, 1H), 4.31 (s, 1H), 4.07 (dd, *J* = 9.2, 5.2 Hz, 1H), 3.80 (s, 3H), 2.57 – 2.44 (m, 2H), 2.20 (td, *J* = 12.2, 3.5 Hz, 1H), 1.98 (s, 3H), 1.95 (ddd, *J* = 13.5, 4.0, 2.3 Hz, 1H), 1.92 – 1.87 (m, 1H), 1.80 (qd, *J* = 12.8, 3.9 Hz, 1H), 1.53 (td, *J* = 13.1, 4.6 Hz, 1H), 1.30 (s, 3H).

^{13}C NMR (151 MHz, $CDCl_3$) δ 186.3, 176.5, 172.3, 154.6, 150.7, 137.8, 129.2, 127.3, 126.5, 126.2, 125.6, 80.4, 64.6, 58.9, 55.2, 52.8, 50.0, 41.2, 37.8, 34.0, 25.1, 19.8, 10.8.

HRMS(ESI): $[M+H]^+$ calcd. $C_{23}H_{26}NO_5S$ m/z 428.1526, found 428.1524.

IR (film, cm^{-1}): 3337, 2931, 2855, 2160, 1791, 1731, 1657, 1626, 1610, 1437, 1404, 1386, 1336, 1293, 1264, 1223, 1198, 1166, 1143, 1129, 1117, 1088, 1054, 1043, 1019, 992, 955, 902, 878, 842.

$[\alpha]_D^{20}$ = -63.2 (c = 0.22, $CHCl_3$)

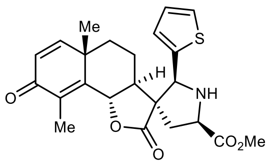

10p

Methyl (2'*R*,3*S*,3a*S*,5a*S*,5'*R*,9b*S*)-2'-(thiophen-2-yl)-5a,9-dimethyl-2,8-dioxo-3a,4,5,5a,8,9b-hexahydro-2*H*-spiro[naphtho[1,2-*b*]furan-3,3'-pyrrolidine]-5'-carboxylate

The title product compound **10p** was prepared in 0.10 mmol scale using **Condition B** and isolated by column chromatography (20:1 DCM: MeOH) giving an amorphous solid (37.2 mg, 0.09 mmol, 87% yield).

^{1}H NMR (400 MHz, CDCl$_3$) δ 7.31 – 7.25 (m, 1H), 7.23 (d, *J* = 3.7 Hz, 1H), 7.05 (dd, *J* = 5.1, 3.6 Hz, 1H), 6.62 (d, *J* = 9.9 Hz, 1H), 6.19 (d, *J* = 9.9 Hz, 1H), 4.59 (s, 1H), 4.34 (dd, *J* = 11.7, 1.4 Hz, 1H), 3.97 (dd, *J* = 10.0, 4.8 Hz, 1H), 3.83 (s, 3H), 2.66 (dd, *J* = 13.7, 4.8 Hz, 1H), 2.44 – 2.22 (m, 2H), 2.16 – 2.05 (m, 1H), 2.00 (s, 3H), 1.97 – 1.88 (m, 2H), 1.51 – 1.39 (m, 1H), 1.11 (s, 3H).

^{13}C NMR (101 MHz, CDCl$_3$) δ 186.3, 177.7, 173.0, 154.7, 151.1, 139.6, 128.8, 128.4, 126.0, 126.0, 125.4, 79.8, 61.6, 58.1, 56.7, 52.7, 52.0, 41.2, 38.8, 37.9, 24.9, 18.9, 11.0.

HRMS(ESI): $[M+H]^+$ calcd. $C_{23}H_{26}NO_5S$ m/z 428.1526, found 428.1517.

IR (film, cm^{-1}): 2928, 1772, 1736, 1661, 1633, 1614, 1436, 1378, 1308, 1270, 1220, 1202, 1155, 1106, 1037, 991, 957, 902, 832.

$[\alpha]_D^{20}$ = -103.1 (c = 0.26, $CHCl_3$)

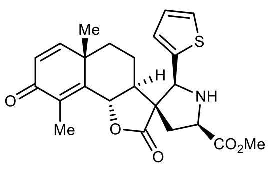

11p

Methyl (2'*R*,3*R*,3a*S*,5a*S*,5'*R*,9b*S*)-2'-(thiophen-2-yl)-5a,9-dimethyl-2,8-dioxo-3a,4,5,5a,8,9b-hexahydro-2*H*-spiro[naphtho[1,2-*b*]furan-3,3'-pyrrolidine]-5'-carboxylate

The title product compound **11p** was prepared in 0.10 mmol scale using **Condition C** and isolated by column chromatography (1:1 Cyclohexane: Ethyl acetate) giving an amorphous solid (39.8 mg, 0.09 mmol, 93% yield).

^{1}H NMR (500 MHz, CDCl$_3$) δ 7.20 (dd, *J* = 5.0, 1.3 Hz, 1H), 6.96 (dt, *J* = 7.2, 3.5 Hz, 2H), 6.59 (d, *J* = 9.8 Hz, 1H), 6.19 (d, *J* = 9.8 Hz, 1H), 5.00 (s, 1H), 4.79 (d, *J* = 11.7 Hz, 1H), 4.20 (t, *J* = 8.3 Hz, 1H), 3.81 (s, 3H), 2.55 (dd, *J* = 13.4, 7.8 Hz, 1H), 2.31 (dd, *J* = 13.4, 8.7 Hz, 1H), 2.06 (s, 3H), 1.79 (td, *J* = 12.1, 3.5 Hz, 1H), 1.69 (dd, *J* = 13.5, 3.2 Hz, 1H), 1.55 (qd, *J* = 12.9, 3.8 Hz, 1H), 1.35 (d, *J* = 15.6 Hz, 1H), 1.24 (s, 3H), 1.18 (td, *J* = 13.2, 4.5 Hz, 1H).

^{13}C NMR (126 MHz, CDCl$_3$) δ 186.3, 178.1, 172.9, 154.9, 151.0, 143.3, 129.2, 127.5, 126.0, 125.2, 124.9, 80.0, 63.5, 57.2, 55.3, 52.5, 50.4, 41.1, 37.6, 30.7, 25.1, 20.2, 11.1.

HRMS(ESI): $[M+H]^+$ calcd. $C_{23}H_{26}NO_5S$ m/z 428.1526, found 428.1514.

IR (film, cm^{-1}): 2948, 2160, 2032, 1770, 1738, 1661, 1633, 1614, 1437, 1378, 1307, 1270, 1193, 1054, 1016, 996, 967, 902, 832.

$[\alpha]_D^{20}$ = +34.1 (c = 0.21, $CHCl_3$)

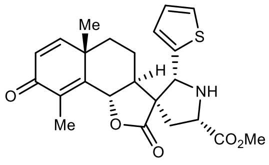

12p

Methyl (2'*S*,3*S*,3a*S*,5a*S*,5'*S*,9b*S*)-2'-(thiophen-2-yl)-5a,9-dimethyl-2,8-dioxo-3a,4,5,5a,8,9b-hexahydro-2*H*-spiro[naphtho[1,2-*b*]furan-3,3'-pyrrolidine]-5'-carboxylate

The title product compound **12p** was prepared in 0.10 mmol scale using **Condition D** and isolated by column chromatography (1:1 Cyclohexane: Ethyl acetate) giving an amorphous solid (34.6 mg, 0.08 mmol, 81% yield).

^{1}H NMR (500 MHz, CDCl$_3$) δ 7.30 (d, *J* = 5.0 Hz, 1H), 7.19 (d, *J* = 3.5 Hz, 1H), 7.03 (dd, *J* = 4.7, 3.6 Hz, 1H), 6.55 (d, *J* = 9.9 Hz, 1H), 6.16 (dt, *J* = 9.9, 1.2 Hz, 1H), 4.74 (s, 1H), 4.47 (dd, *J* = 11.9, 1.3 Hz, 1H), 4.16 (dd, *J* = 10.6, 5.5 Hz, 1H), 3.79 (s, 3H), 2.94 (dd, *J* = 13.1, 10.7 Hz, 1H), 2.20 (dd, *J* = 13.9, 5.6 Hz, 1H), 2.14 – 1.90 (m, 6H), 1.71 (ddd, *J* = 13.5, 4.0, 2.3 Hz, 1H), 1.29 – 1.19 (m, 2H), 0.79 (s, 3H).

^{13}C NMR (126 MHz, CDCl$_3$) δ 186.5, 178.2, 173.4, 155.2, 151.5, 140.5, 128.5, 127.6, 125.9, 125.7, 125.3, 79.8, 64.6, 56.7, 56.5, 55.2, 52.6, 41.3, 39.9, 38.5, 24.2, 18.3, 11.1.

HRMS(ESI): $[M+H]^+$ calcd. $C_{23}H_{26}NO_5S$ m/z 428.1526, found 428.1523.

IR (film, cm^{-1}): 3325, 2921, 2851, 1770, 1737, 1661, 1634, 1614, 1436, 1377, 1273, 1186, 1162, 1106, 1038, 990, 957, 901, 831.

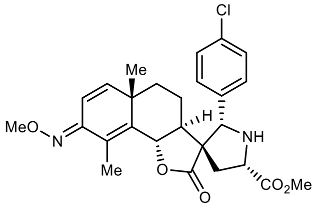

20

Methyl (2'*S*,3*R*,3a*S*,5a*S*,5'*S*,9b*S*,*E*)-2'-(4-chlorophenyl)-8-(methoxyimino)-5a,9-dimethyl-2-oxo-3a,4,5,5a,8,9b-hexahydro-2*H*-spiro[naphtho[1,2-*b*]furan-3,3'-pyrrolidine]-5'-carboxylate

The title product compound **20** was prepared in 0.10 mmol scale using **Condition A** and isolated by column chromatography (20:1 DCM: MeOH) giving an amorphous solid (45.5 mg, 0.09 mmol, 94% yield).

^{1}H NMR (500 MHz, CDCl$_3$) δ 7.34-7.28 (m, 4H), 6.78 (d, *J* = 10.1 Hz, 1H), 5.95 (d, *J* = 10.1 Hz, 1H), 4.77 (dd, *J* = 11.6, 1.6 Hz, 1H), 4.06-4.02 (m, 2H), 3.89 (s, 3H), 3.80 (s, 3H), 2.52 (dd, *J* = 13.4, 8.8 Hz, 1H), 2.41 (dd, *J* = 13.4, 5.0 Hz, 1H), 1.99 (ddd, *J* = 12.0, 10.5, 5.5 Hz, 1H), 1.93-1.86 (m, 4H), 1.84 – 1.73 (m, 2H), 1.54 – 1.45 (m, 1H), 1.20 (s, 3H).

^{13}C NMR (126 MHz, CDCl$_3$) δ 176.5, 172.6, 149.7, 144.8, 137.9, 134.4, 134.2, 129.7, 128.8, 123.5, 113.2, 80.9, 67.8, 62.1, 58.8, 55.0, 52.7, 50.2, 40.5, 38.0, 33.9, 25.8, 19.9, 12.0.

HRMS(ESI): $[M+H]^+$ calcd. $C_{26}H_{30}N_2O_5Cl$ m/z 485.1838, found 485.1830.

IR (film, cm^{-1}): 2936, 1774, 1737, 1597, 1492, 1436, 1374, 1314, 1269, 1225, 1202, 1166, 1111, 1092, 1038, 1014, 988, 904, 870, 833.

$[\alpha]_D^{20}$ = -3.3 (c = 0.15, $CHCl_3$)

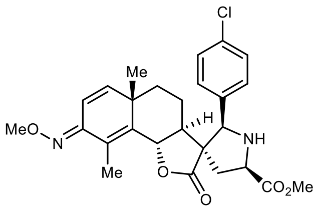

21

Methyl (2'*R*,3*S*,3a*S*,5a*S*,5'*R*,9b*S*,*E*)-2'-(4-chlorophenyl)-8-(methoxyimino)-5a,9-dimethyl-2-oxo-3a,4,5,5a,8,9b-hexahydro-2*H*-spiro[naphtho[1,2-*b*]furan-3,3'-pyrrolidine]-5'-carboxylate

The title product compound **21** was prepared in 0.10 mmol scale using **Condition B** and isolated by column chromatography (20:1 DCM: MeOH) giving an amorphous solid (47.6 mg, 0.10 mmol, 98% yield).

^{1}H NMR (500 MHz, $CDCl_3$) δ 7.42 (d, *J* = 8.6 Hz, 2H), 7.36 (d, *J* = 8.5 Hz, 2H), 6.75 (d, *J* = 10.1 Hz, 1H), 5.90 (d, *J* = 10.2 Hz, 1H), 4.38 (s, 1H), 4.24 (dd, *J* = 11.7, 1.6 Hz, 1H), 3.99 (dd, *J* = 9.6, 5.2 Hz, 1H), 3.89 (s, 3H), 3.84 (s, 3H), 2.65 (dd, *J* = 13.5, 5.0 Hz, 1H), 2.31 (dd, *J* = 13.6, 9.5 Hz, 1H), 2.21 (td, *J* = 12.2, 3.6 Hz, 1H), 2.03 – 1.89 (m, 5H), 1.75 (ddd, *J* = 13.6, 3.9, 2.2 Hz, 1H), 1.43 (td, *J* = 13.1, 4.6 Hz, 1H), 1.00 (s, 3H).

^{13}C NMR (126 MHz, $CDCl_3$) δ 177.7, 173.1, 149.7, 144.6, 137.9, 135.9, 135.3, 129.7, 129.3, 123.2, 113.1, 80.5, 65.7, 62.1, 58.4, 56.6, 52.9, 52.5, 40.5, 38.5, 38.3, 25.5, 19.8, 12.1.

HRMS(ESI): $[M+H]^+$ calcd. $C_{26}H_{30}N_2O_5Cl$ m/z 485.1838, found 485.1833.

IR (film, cm^{-1}): 2932, 1770, 1737, 1491, 1436, 1374, 1316, 1266, 1204, 1155, 1093, 1037, 1014, 988, 944, 902, 868, 835.

$[\alpha]_D^{20}$ = -90.3 (c = 0.15, $CHCl_3$)

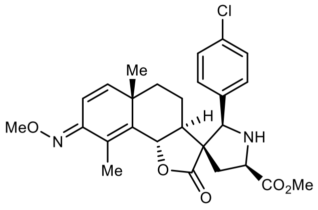

22

Methyl (2'*R*,3*R*,3a*S*,5a*S*,5'*R*,9b*S*,*E*)-2'-(4-chlorophenyl)-8-(methoxyimino)-5a,9-dimethyl-2-oxo-3a,4,5,5a,8,9b-hexahydro-2*H*-spiro[naphtho[1,2-*b*]furan-3,3'-pyrrolidine]-5'-carboxylate

The title product compound **22** was prepared in 0.10 mmol scale using **Condition C** and isolated by column chromatography (1:1 Cyclohexane: Ethyl acetate) giving an amorphous solid (47.5 mg, 0.10 mmol, 98% yield).

^{1}H NMR (500 MHz, $CDCl_3$) δ 7.35 – 7.25 (m, 4H), 6.73 (d, *J* = 10.1 Hz, 1H), 5.85 (d, *J* = 10.2 Hz, 1H), 4.76 (dd, *J* = 11.3, 1.5 Hz, 1H), 4.73 (s, 1H), 4.21 (t, *J* = 8.3 Hz, 1H), 3.90 (s, 3H), 3.81 (s, 3H), 2.48 (dd, *J* = 13.4, 7.9 Hz, 1H),

2.29 (dd, *J* = 13.3, 8.8 Hz, 1H), 2.06 (s, 3H), 1.59 (td, *J* = 11.7, 3.2 Hz, 1H), 1.54 – 1.44 (m, 2H), 1.24 – 1.18 (m, 1H), 1.13 (s, 3H), 1.11 – 1.04 (m, 1H).

^{13}C NMR (126 MHz, $CDCl_3$) δ 178.8, 173.3, 149.9, 145.0, 138.1, 137.5, 134.0, 128.9, 128.6, 123.3, 113.0, 80.8, 77.4, 66.5, 62.1, 57.2, 55.0, 52.5, 50.1, 40.4, 37.8, 31.2, 25.8, 20.7, 12.2.

HRMS(ESI): $[M+H]^+$ calcd. $C_{26}H_{30}N_2O_5Cl$ m/z 485.1838, found 485.1832.

IR (film, cm^{-1}): 2937, 1770, 1739, 1490, 1437, 1373, 1326, 1275, 1199, 1151, 1090, 1047, 1014, 951, 913, 888, 870, 839.

$[\alpha]_D^{20}$ = +152.4 (c = 0.11, $CHCl_3$)

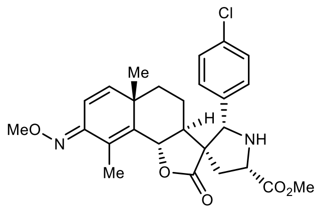

23

Methyl (2'*S*,3*S*,3a*S*,5a*S*,5'*S*,9b*S*,*E*)-2'-(4-chlorophenyl)-8-(methoxyimino)-5a,9-dimethyl-2-oxo-3a,4,5,5a,8,9b-hexahydro-2*H*-spiro[naphtho[1,2-*b*]furan-3,3'-pyrrolidine]-5'-carboxylate

The title product compound **23** was prepared in 0.10 mmol scale using **Condition D** and isolated by column chromatography (1:1 Cyclohexane: Ethyl acetate) giving an amorphous solid (47.1 mg, 0.10 mmol, 97% yield).

^{1}H NMR (500 MHz, $CDCl_3$) δ 7.48 (d, *J* = 8.1 Hz, 2H), 7.41 – 7.33 (m, 2H), 6.69 (d, *J* = 10.2 Hz, 1H), 5.80 (d, *J* = 10.2 Hz, 1H), 4.48 (s, 1H), 4.17 (dd, *J* = 10.5, 1.4 Hz, 1H), 3.88 (s, 3H), 3.79 (s, 3H), 2.99 (dd, *J* = 13.8, 10.7 Hz, 1H), 2.15 (dd, *J* = 13.9, 5.8 Hz, 1H), 2.06 (s, 3H), 1.95 (td, *J* = 12.6, 3.5 Hz, 1H), 1.91 – 1.85 (m, 1H), 1.79 (td, *J* = 13.0, 3.9 Hz, 1H), 1.48 (ddd, *J* = 13.4, 3.9, 2.3 Hz, 1H), 1.17 (td, *J* = 13.0, 4.7 Hz, 1H), 0.56 (s, 3H).

^{13}C NMR (126 MHz, $CDCl_3$) δ 179.1, 173.5, 149.9, 145.1, 138.7, 135.5, 134.3, 129.0, 128.6, 122.6, 112.8, 80.6, 67.8, 62.0, 57.0, 56.7, 54.9, 52.6, 40.7, 40.5, 38.9, 24.7, 18.7, 12.1.

HRMS(ESI): $[M+H]^+$ calcd. $C_{26}H_{30}N_2O_5Cl$ m/z 485.1838, found 485.1832.

IR (film, cm^{-1}): 2927, 1769, 1739, 1492, 1437, 1380, 1275, 1201, 1165, 1091, 1038, 1014, 987, 955, 908, 869, 826.

$[\alpha]_D^{20}$ = -60.0 (c = 0.17, $CHCl_3$)

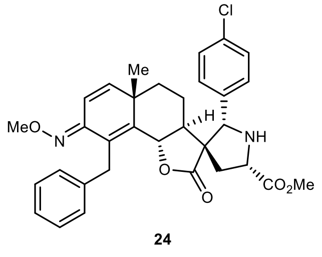

24

Methyl (2'*S*,3*R*,3a*S*,5a*S*,5'*S*,9b*S*,*E*)-9-benzyl-2'-(4-chlorophenyl)-8-(methoxyimino)-5a-methyl-2-oxo-3a,4,5,5a,8,9b-hexahydro-2*H*-spiro[naphtho[1,2-*b*]furan-3,3'-pyrrolidine]-5'-carboxylate

The title product compound **24** was prepared in 0.10 mmol scale using **Condition A** and isolated by column chromatography (20:1 DCM: MeOH) giving an amorphous solid (55.0 mg, 0.10 mmol, 98% yield).

^{1}H NMR (500 MHz, CDCl$_3$) δ 7.27 – 7.18 (m, 4H), 7.07 – 7.00 (m, 1H), 6.97 (dd, *J* = 8.3, 6.7 Hz, 2H), 6.91 (dd, *J* = 8.1, 1.5 Hz, 2H), 6.78 (d, *J* = 10.1 Hz, 1H), 5.95 (d, *J* = 10.1 Hz, 1H), 4.81 (d, *J* = 11.6 Hz, 1H), 4.05 – 3.95 (m, 4H), 3.83 (s, 3H), 3.79 (s, 3H), 2.50 (dd, *J* = 13.5, 9.1 Hz, 1H), 2.41 (dd, *J* = 13.4, 4.8 Hz, 1H), 1.99 – 1.72 (m, 4H), 1.58 (ddd, *J* = 14.0, 13.6, 4.8 Hz, 1H), 1.25 (s, 3H).

^{13}C NMR (126 MHz, CDCl$_3$) δ 175.9, 172.6, 148.4, 144.4, 141.5, 138.9, 134.3, 134.0, 129.7, 128.8, 128.1, 127.7, 126.6, 125.1, 113.4, 80.6, 67.6, 62.2, 58.6, 54.7, 52.7, 49.9, 40.7, 38.0, 33.8, 30.4, 26.1, 19.8.

HRMS(ESI): $[M+H]^+$ calcd. $C_{32}H_{34}N_2O_5Cl$ m/z 561.2151, found 561.2146.

IR (film, cm^{-1}): 2937, 1774, 1737, 1663, 1600, 1494, 1452, 1436, 1374, 1314, 1266, 1223, 1186, 1154, 1092, 1052, 1014, 908, 895, 823.

$[\alpha]_D^{20}$ = -8.0 (c = 0.18, $CHCl_3$)

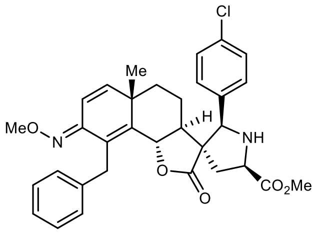

25

Methyl (2'*R*,3*S*,3a*S*,5a*S*,5'*R*,9b*S*,*E*)-9-benzyl-2'-(4-chlorophenyl)-8-(methoxyimino)-5a-methyl-2-oxo-3a,4,5,5a,8,9b-hexahydro-2*H*-spiro[naphtho[1,2-*b*]furan-3,3'-pyrrolidine]-5'-carboxylate

The title product compound **25** was prepared in 0.10 mmol scale using **Condition B** and isolated by column chromatography (20:1 DCM: MeOH) giving an amorphous solid (52.6 mg, 0.09 mmol, 94% yield).

^{1}H NMR (500 MHz, CDCl$_3$) δ 7.38 – 7.33 (m, 4H), 7.18 – 7.11 (m, 2H), 7.10 – 7.04 (m, 3H), 6.79 (d, *J* = 10.1 Hz, 1H), 5.93 (d, *J* = 10.2 Hz, 1H), 4.28 (s, 1H), 4.21 – 4.16 (m, 2H), 3.93 – 3.90 (m, 1H), 3.86 (s, 3H), 3.83 (s, 3H), 2.29 (dd, *J* = 13.5, 5.3 Hz, 1H), 2.08 (dd, *J* = 13.6, 9.5 Hz, 1H), 2.04 (s, 1H), 1.99 – 1.83 (m, 3H), 1.77 (d, *J* = 13.2 Hz, 1H), 1.48-1.41 (m, 1H), 1.04 (s, 3H).

^{13}C NMR (126 MHz, CDCl$_3$) δ 177.1, 173.4, 148.7, 144.6, 142.3, 139.3, 136.5, 134.9, 129.5, 129.2, 128.5, 127.8, 126.3, 125.1, 113.3, 80.0, 65.7, 62.2, 58.4, 56.4, 52.6, 51.8, 40.8, 38.8, 38.7, 29.9, 25.4, 19.9.

HRMS(ESI): $[M+H]^+$ calcd. $C_{32}H_{34}N_2O_5Cl$ m/z 561.2151, found 561.2146.

IR (film, cm^{-1}): 2936, 1770, 1737, 1663, 1600, 1493, 1452, 1435, 1374, 1316, 1265, 1221, 1181, 1154, 1093, 1051, 1031, 1014, 959, 941, 909, 893, 860, 824.

$[\alpha]_D^{20}$ = -122.5 (c = 0.12, $CHCl_3$)

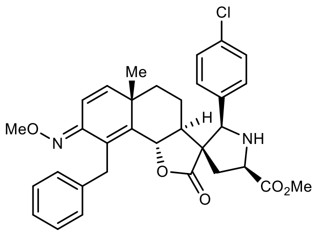

26

Methyl (2'*R*,3*R*,3a*S*,5a*S*,5'*R*,9b*S*,*E*)-9-benzyl-2'-(4-chlorophenyl)-8-(methoxyimino)-5a-methyl-2-oxo-3a,4,5,5a,8,9b-hexahydro-2*H*-spiro[naphtho[1,2-*b*]furan-3,3'-pyrrolidine]-5'-carboxylate

The title product compound **26** was prepared in 0.10 mmol scale using **Condition C** and isolated by column chromatography (1:1 Cyclohexane: Ethyl acetate) giving an amorphous solid (53.4 mg, 0.10 mmol, 95% yield).

^{1}H NMR (500 MHz, CDCl$_3$) δ 7.24 – 7.17 (m, 4H), 7.17 – 7.12 (m, 1H), 7.11 – 7.06 (m, 4H), 6.76 (d, J = 10.1 Hz, 1H), 5.87 (d, J = 10.1 Hz, 1H), 4.75 (d, J = 10.8 Hz, 1H), 4.45 (s, 1H), 4.22 – 4.14 (m, 2H), 4.03 (d, J = 14.9 Hz, 1H), 3.84 (s, 3H), 3.79 (s, 3H), 2.37 (dd, J = 13.3, 8.3 Hz, 1H), 2.22 (dd, J = 13.3, 8.5 Hz, 1H), 1.54 – 1.40 (m, 3H), 1.18 (s, 3H), 1.14 – 1.04 (m, 2H).

^{13}C NMR (126 MHz, CDCl$_3$) δ 178.4, 173.4, 148.7, 144.8, 142.3, 139.5, 137.7, 133.6, 128.7, 128.5, 128.4, 127.9, 126.2, 125.2, 113.1, 80.6, 66.2, 62.2, 57.2, 54.6, 52.5, 49.7, 40.7, 38.3, 31.2, 30.3, 25.6, 20.8.

HRMS(ESI): $[M+H]^+$ calcd. $C_{32}H_{34}N_2O_5Cl$ m/z 561.2151, found 561.2146.

IR (film, cm^{-1}): 2938, 1771, 1738, 1663, 1600, 1492, 1452, 1437, 1375, 1327, 1275, 1195, 1154, 1089, 1052, 1031, 1013, 909, 886, 846.

$[\alpha]_D^{20}$ = +176.5 (c = 0.12, $CHCl_3$)

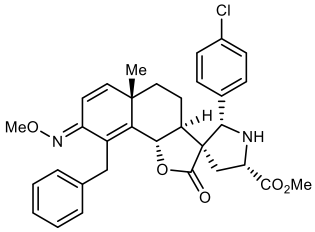

27

Methyl (2'*S*,3*S*,3a*S*,5a*S*,5'*S*,9b*S*,*E*)-9-benzyl-2'-(4-chlorophenyl)-8-(methoxyimino)-5a-methyl-2-oxo-3a,4,5,5a,8,9b-hexahydro-2*H*-spiro[naphtho[1,2-*b*]furan-3,3'-pyrrolidine]-5'-carboxylate

The title product compound **27** was prepared in 0.10 mmol scale using **Condition D** and isolated by column chromatography (1:1 Cyclohexane: Ethyl acetate) giving an amorphous solid (51.6 mg, 0.09 mmol, 92% yield).

^{1}H NMR (500 MHz, CDCl$_3$) δ 7.43 (d, J = 8.1 Hz, 2H), 7.38 – 7.31 (m, 2H), 7.21 - 7.16 (m, 2H), 7.14 – 7.06 (m, 3H), 6.73 (d, J = 10.1 Hz, 1H), 5.83 (d, J = 10.2 Hz, 1H), 4.37 (s, 1H), 4.24 (d, J = 15.0 Hz, 1H), 4.13 (d, J = 11.7 Hz, 1H), 4.04 (dd, J = 10.8, 5.6 Hz, 1H), 3.97 (d, J = 15.0 Hz, 1H), 3.84 (s, 3H), 3.77 (s, 3H), 2.73 (dd, J = 13.9, 10.7 Hz, 1H), 1.85 – 1.65 (m, 4H), 1.54 – 1.48 (m, 1H), 1.20 (td, J = 12.7, 5.0 Hz, 1H), 0.61 (s, 3H).

^{13}C NMR (126 MHz, CDCl$_3$) δ 178.6, 173.6, 148.8, 145.0, 142.4, 140.0, 135.6, 134.2, 125.6, 125.1, 113.0, 80.3, 67.8, 62.2, 56.5, 56.4, 54.6, 52.5, 40.9, 40.8, 39.4, 30.1, 24.5, 18.8.

HRMS(ESI): [M+H]$^+$ calcd. $C_{32}H_{34}N_2O_5Cl$ m/z 561.2151, found 561.2146.

IR (film, cm^{-1}): 2936, 1770, 1739, 1663, 1600, 1493, 1452, 1436, 1275, 1198, 1164, 1117, 1091, 1051, 1031, 1012.89, 986, 958, 909, 887, 825.

[α]$_D^{20}$ = -72.8 (c = 0.18, $CHCl_3$)

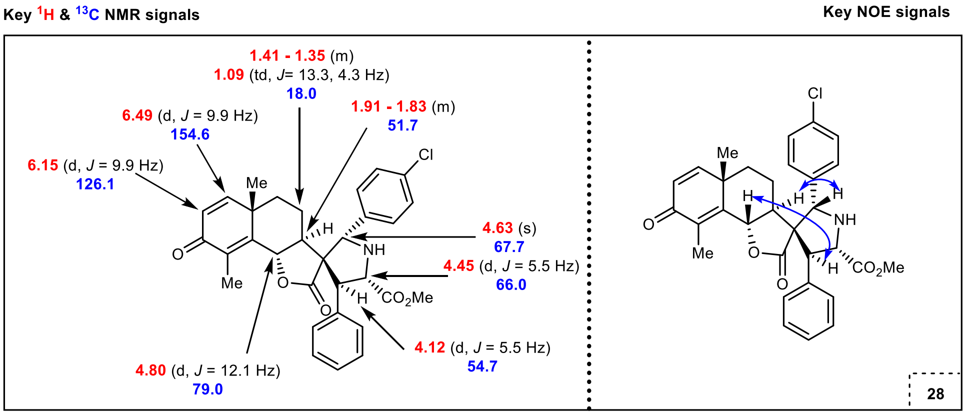

Methyl (2'*S*,3*R*,3a*S*,4'*S*,5a*S*,5'*S*,9b*S*)-2'-(4-chlorophenyl)-5a,9-dimethyl-2,8-dioxo-4'-phenyl-3a,4,5,5a,8,9b-hexahydro-2*H*-spiro[naphtho[1,2-*b*]furan-3,3'-pyrrolidine]-5'-carboxylate

The title product compound **28** was prepared from substrate **15** (16.0 mg, 0.05 mmol) using **Condition A** and isolated by column chromatography (20:1 DCM: MeOH) giving an amorphous solid (15.2 mg, 0.03 mmol, 57% yield) as a mixture of diastereomers (**28** + **29**; 3.17:1). The yield for the main isomer **28** is 43%.

^{1}H NMR (500 MHz, $CDCl_3$) δ 7.52 – 7.31 (m, 9H), 6.49 (d, *J* = 9.9 Hz, 1H), 6.15 (d, *J* = 9.9 Hz, 1H), 4.80 (dd, *J* = 12.1, 1.6 Hz, 1H), 4.63 (s, 1H), 4.45 (d, *J* = 5.5 Hz, 1H), 4.12 (d, *J* = 5.5 Hz, 1H), 3.81 (s, 3H), 1.92 (s, 3H), 1.91 – 1.83 (m, 1H), 1.55 (ddd, *J* = 13.5, 3.9, 2.3 Hz, 1H), 1.41 – 1.35 (m, 1H), 1.09 (td, *J* = 13.3, 4.3 Hz, 1H), 0.87 (s, 3H), 0.80 (td, *J* = 13.3, 3.9 Hz, 1H).

^{13}C NMR (126 MHz, $CDCl_3$) δ 186.2, 176.2, 154.6, 150.3, 138.6, 135.0, 129.9, 129.2, 126.1, 79.0, 67.7, 66.0, 59.6, 54.7, 53.2, 51.7, 41.1, 37.9, 24.4, 18.0, 11.2.

HRMS(ESI): [M+H]$^+$ calcd. $C_{31}H_{31}NO_5Cl$ m/z 532.1885, found 532.1879.

IR (film, cm^{-1}): 2922, 1772, 1738, 1662, 1635, 1493, 1454, 1436, 1375, 1159, 1091, 1055, 1014, 904, 831.

[α]$_D^{20}$ = -25.0 (c = 0.06, $CHCl_3$)

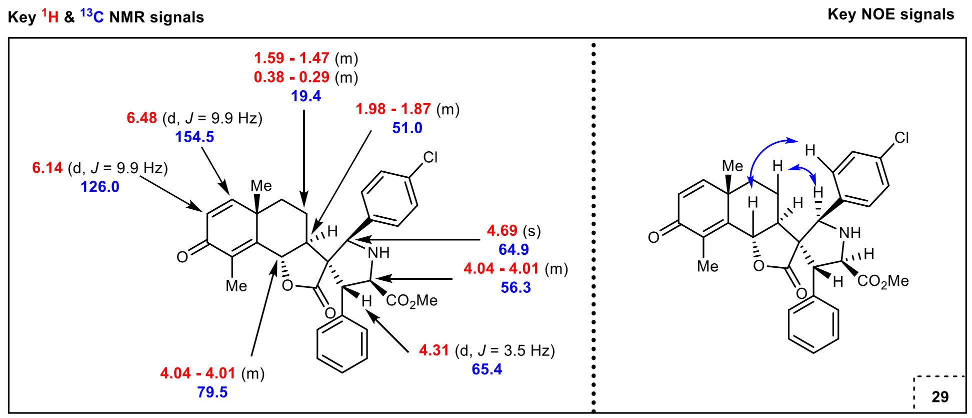

Methyl (2'*R*,3*S*,3a*S*,4'*R*,5a*S*,5'*R*,9b*S*)-2'-(4-chlorophenyl)-5a,9-dimethyl-2,8-dioxo-4'-phenyl-3a,4,5,5a,8,9b-hexahydro-2*H*-spiro[naphtho[1,2-*b*]furan-3,3'-pyrrolidine]-5'-carboxylate

The title product compound **29** was prepared from substrate **15** (16.0 mg, 0.05 mmol) using **Condition B** and isolated by column chromatography (20:1 DCM: MeOH) giving an amorphous solid (23.9 mg, 0.04 mmol, 90% yield).

^{1}H NMR (500 MHz, $CDCl_3$) δ 7.49 (d, *J* = 8.5 Hz, 2H), 7.43 – 7.30 (m, 7H), 6.48 (d, *J* = 9.9 Hz, 1H), 6.14 (d, *J* = 9.8 Hz, 1H), 4.69 (s, 1H), 4.31 (d, *J* = 3.5 Hz, 1H), 4.07 – 3.98 (m, 2H), 3.86 (s, 3H), 2.02 – 1.87 (m, 4H), 1.59 – 1.47 (m, 2H), 0.96 – 0.84 (m, 4H), 0.38 – 0.29 (m, 1H).

^{13}C NMR (126 MHz, $CDCl_3$) δ 186.2, 177.5, 173.4, 154.5, 150.7, 138.5, 136.0, 135.5, 129.9, 129.5, 129.2, 128.9, 128.5, 128.3, 126.0, 79.4, 65.4, 64.9, 60.3, 56.3, 53.1, 51.0, 40.9, 37.7, 24.7, 19.3, 11.0.

HRMS(ESI): $[M+H]^+$ calcd. $C_{31}H_{31}NO_5Cl$ m/z 532.1885, found 532.1880.

IR (film, cm^{-1}): 2925, 1771, 1736, 1662, 1635, 1615, 1492, 1454, 1435, 1406, 1376, 1307, 1264, 1226, 1184, 1157, 1093, 1055, 1013, 996, 941, 903, 832.

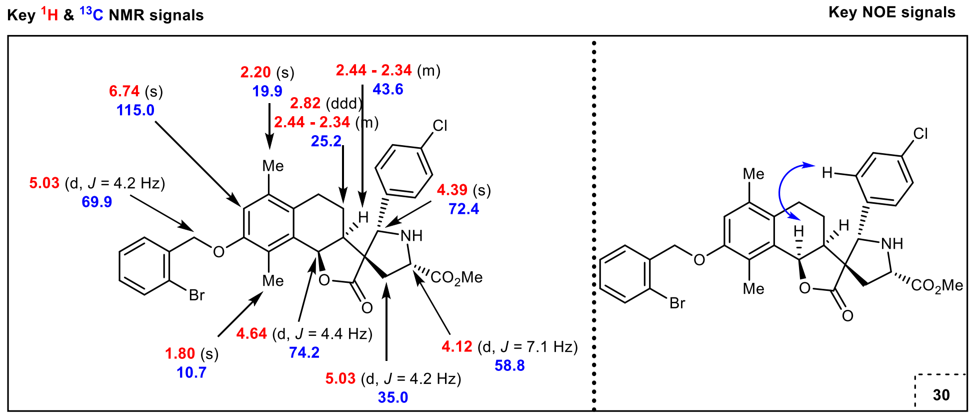

Methyl (2'*S*,3*R*,3a*S*,5'*S*,9b*R*)-8-((2-bromobenzyl)oxy)-2'-(4-chlorophenyl)-6,9-dimethyl-2-oxo-3a,4,5,9b-tetrahydro-2*H*-spiro[naphtho[1,2-*b*]furan-3,3'-pyrrolidine]-5'-carboxylate

The title product compound **30** was prepared from substrate **16** (20.7 mg, 0.05 mmol) using **Condition A** and isolated by column chromatography (20:1 DCM: MeOH) giving an amorphous solid (27.6 mg, 0.04 mmol, 88% yield).

^{1}H NMR (600 MHz, CDCl$_3$) δ 7.56 (dd, *J* = 8.0, 1.2 Hz, 1H), 7.53 (dd, *J* = 7.7, 1.7 Hz, 1H), 7.44 (d, *J* = 8.2 Hz, 2H), 7.38 (d, *J* = 8.1 Hz, 2H), 7.33 (td, *J* = 7.5, 1.2 Hz, 1H), 7.18 (td, *J* = 7.7, 1.7 Hz, 1H), 6.74 (s, 1H), 5.03 (d, *J* = 4.2 Hz, 2H), 4.64 (d, *J* = 4.4 Hz, 1H), 4.39 (s, 1H), 4.12 (d, *J* = 7.1 Hz, 1H), 3.87 (s, 3H), 2.82 (d, *J* = 14.5 Hz, 1H), 2.62 (dd, *J* = 13.5, 4.2 Hz, 1H), 2.51 (dd, *J* = 13.5, 9.5 Hz, 1H), 2.44 – 2.34 (m, 2H), 2.20 (s, 3H), 2.02 – 1.96 (m, 1H), 1.80 (s, 3H), 1.49-1.41 (m, 1H), 0.94 – 0.85 (m, 1H).

^{13}C NMR (151 MHz, CDCl$_3$) δ 177.8, 173.5, 171.3, 154.6, 136.7, 136.2, 135.3, 134.2, 132.6, 129.5, 129.5, 129.2, 129.1, 128.7, 128.4, 127.7, 125.9, 122.2, 115.0, 74.2, 72.4, 69.9, 61.1, 60.5, 58.8, 52.8, 43.6, 35.0, 25.2, 22.1, 21.2, 19.9, 14.3, 10.7.

HRMS(ESI): [M+H]$^+$ calcd. $C_{32}H_{32}NO_5ClBr$ m/z 624.1147, found 624.1145.

IR (film, cm^{-1}): 3356, 2926, 2852, 2160, 1762, 1738, 1601, 1484, 1437, 1384, 1353, 1339, 1304, 1212, 1194, 1172, 1132, 1112, 1087, 1045, 1028, 1012, 984, 945, 920, 862, 840.

[α]$_D^{20}$ = -36.3 (c = 0.25, CHCl$_3$)

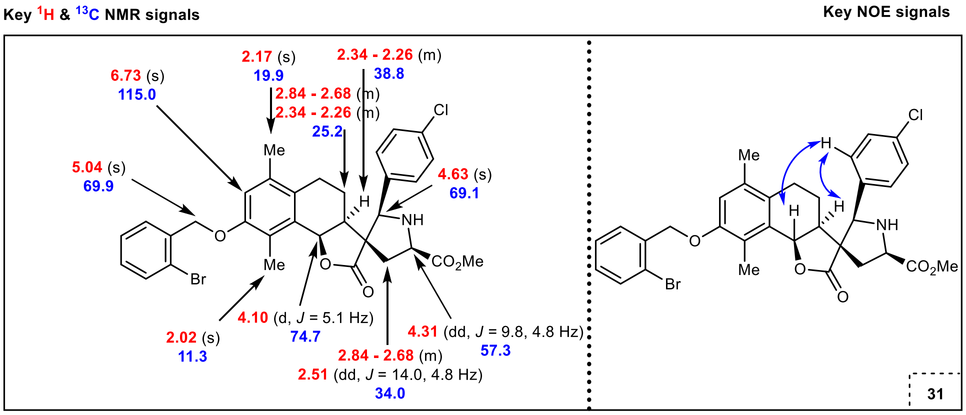

Methyl (2'*R*,3*R*,3a*S*,5'*R*,9b*R*)-8-((2-bromobenzyl)oxy)-2'-(4-chlorophenyl)-6,9-dimethyl-2-oxo-3a,4,5,9b-tetrahydro-2*H*-spiro[naphtho[1,2-*b*]furan-3,3'-pyrrolidine]-5'-carboxylate

The title product compound **31** was prepared from substrate **16** (20.7 mg, 0.05 mmol) using **Condition C** and isolated by column chromatography (3:1 *n*-pentane: EA) giving an amorphous solid (29.6 mg, 0.05 mmol, 95% yield).

^{1}H NMR (700 MHz, CDCl$_3$) δ 7.60 – 7.49 (m, 4H), 7.37 – 7.31 (m, 3H), 7.18 (td, *J* = 7.6, 1.7 Hz, 1H), 6.73 (s, 1H), 5.04 (s, 2H), 4.63 (s, 1H), 4.31 (dd, *J* = 9.8, 4.8 Hz, 1H), 4.10 (d, *J* = 5.1 Hz, 1H), 3.84 (s, 3H), 2.84 – 2.68 (m, 2H), 2.51 (dd, *J* = 14.0, 4.8 Hz, 1H), 2.34 – 2.26 (m, 2H), 2.17 (s, 3H), 2.02 (s, 3H), 1.85 – 1.80 (m, 1H), 1.32 – 1.27 (m, 1H).

^{13}C NMR (151 MHz, CDCl$_3$) δ 178.7, 174.3, 154.6, 136.7, 136.4, 134.8, 134.1, 132.6, 129.9, 129.2, 129.2, 128.8, 128.6, 128.5, 127.7, 126.0, 122.2, 115.0, 74.7, 70.0, 69.1, 59.4, 57.3, 52.7, 38.8, 34.0, 25.2, 22.2, 19.9, 11.3.

HRMS(ESI): [M+H]$^+$ calcd. $C_{32}H_{32}NO_5ClBr$ m/z 624.1147, found 624.1145.

IR (film, cm^{-1}): 2925, 1745, 1602, 1486, 1437, 1413, 1381, 1335, 1306, 1281, 1201, 1169, 1111, 1090, 1059, 1046, 1026, 1013, 977, 916, 872, 824.

[α]$_D^{20}$ = +13.2 (c = 0.24, CHCl$_3$)

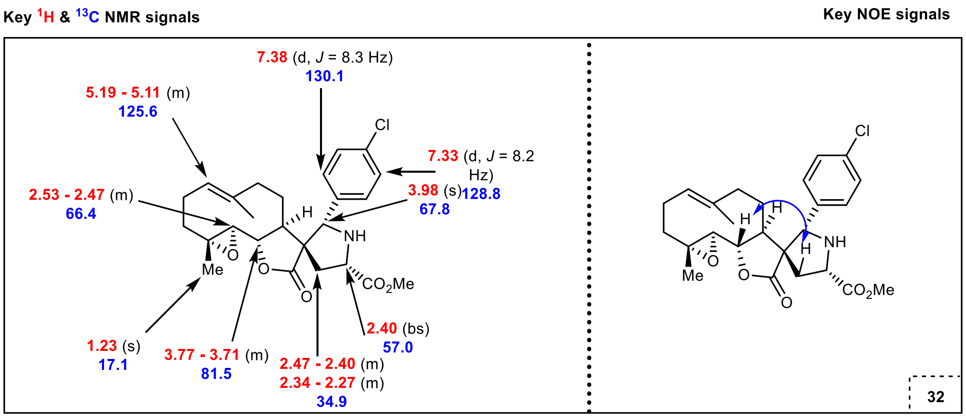

Methyl (2'*S*,3*R*,3a*S*,5'*S*,9a*R*,10a*R*,10b*S*,*E*)-2'-(4-chlorophenyl)-6,9a-dimethyl-2-oxo-3a,4,5,8,9,9a,10a,10b-octahydro-2*H*-spiro[oxireno[2',3':9,10]cyclodeca[1,2-*b*]furan-3,3'-pyrrolidine]-5'-carboxylate

The title product compound **32** was prepared in 0.10 mmol scale using **Condition A** and isolated by column chromatography (30:1 DCM: MeOH) giving an amorphous solid (40.2 mg, 0.09 mmol, 87% yield).

^{1}H NMR (500 MHz, $CDCl_3$) δ 7.38 (d, *J* = 8.3 Hz, 2H), 7.33 (d, *J* = 8.2 Hz, 2H), 5.19 – 5.11 (m, 1H), 4.06 – 3.95 (m, 2H), 3.77 – 3.71 (m, 4H), 2.55 – 2.40 (m, 3H), 2.39 – 2.26 (m, 2H), 2.13 – 2.01 (m, 2H), 1.91 (dd, *J* = 15.0, 6.8 Hz, 1H), 1.70 (s, 3H), 1.67 – 1.56 (m, 1H), 1.23 (s, 3H), 1.12 (td, *J* = 13.0, 5.9 Hz, 1H).

^{13}C NMR (126 MHz, $CDCl_3$) δ 207.2, 176.3, 172.5, 134.5, 134.0, 133.7, 130.1, 128.8, 125.6, 81.5, 67.8, 66.4, 61.6, 58.9, 57.0, 52.7, 47.5, 41.4, 36.7, 34.9, 31.1, 26.0, 24.0, 17.1, 17.0.

HRMS(ESI): $[M+H]^+$ calcd. $C_{25}H_{31}NO_5Cl$ m/z 460.1885, found 460.1881.

IR (film, cm^{-1}): 2926, 2855, 2346, 2158, 2048, 1957, 1765, 1491, 1437, 1387, 1317, 1292, 1270, 1204, 1092, 1074, 1045, 1012, 976, 940, 913, 892, 871, 824.

$[\alpha]_D^{20}$ = +12.3 (c = 0.16, $CHCl_3$)

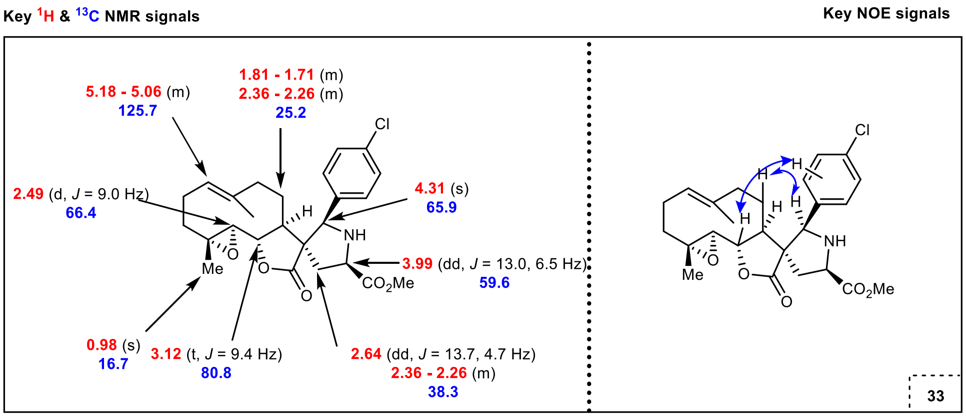

Methyl (2'*R*,3*S*,3a*S*,5'*R*,9a*R*,10a*R*,10b*S*,*E*)-2'-(4-chlorophenyl)-6,9a-dimethyl-2-oxo-3a,4,5,8,9,9a,10a,10b-octahydro-2*H*-spiro[oxireno[2',3':9,10]cyclodeca[1,2-*b*]furan-3,3'-pyrrolidine]-5'-carboxylate

The title product compound **33** was prepared in 0.10 mmol scale using **Condition B** and isolated by column chromatography (30:1 DCM: MeOH) giving an amorphous solid (42.3 mg, 0.09 mmol, 92% yield).

^{1}H NMR (500 MHz, CDCl$_3$) δ 7.33-7.27 (m, 4H), 5.18 – 5.06 (m, 1H), 4.31 (s, 1H), 3.99 (dd, *J* = 10.2, 5.0 Hz, 1H), 3.83 (s, 3H), 3.12 (t, *J* = 9.4 Hz, 1H), 2.64 (dd, *J* = 13.7, 4.7 Hz, 1H), 2.49 (d, *J* = 9.0 Hz, 1H), 2.45 (dd, *J* = 13.0, 6.5 Hz, 1H), 2.36 – 2.26 (m, 3H), 2.15 – 1.95 (m, 4H), 1.81 – 1.71 (m, 1H), 1.68 (s, 3H), 1.10 (td, *J* = 13.0, 5.8 Hz, 1H), 0.98 (s, 3H).

^{13}C NMR (126 MHz, CDCl$_3$) δ 177.4, 173.1, 135.1, 135.0, 133.9, 129.3, 129.1, 125.7, 80.8, 66.4, 65.9, 61.4, 59.6, 58.4, 52.7, 50.2, 41.8, 38.3, 36.7, 25.2, 24.0, 16.9, 16.7.

HRMS(ESI): $[M+H]^+$ calcd. $C_{25}H_{31}NO_5Cl$ m/z 460.1885, found 460.1880.

IR (film, cm^{-1}): 2929, 2861, 1761, 1738, 1492, 1435, 1386, 1316, 1201, 1152, 1113, 1092, 1071, 1044, 1014, 1004, 984, 940, 910, 873, 824.

$[\alpha]_D^{20}$ = +21.9 (c = 0.16, $CHCl_3$)

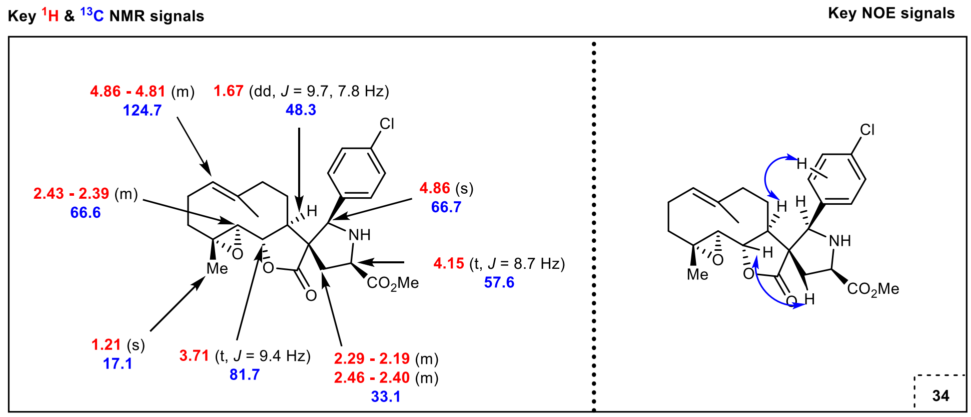

Methyl (2'*R*,3*R*,3a*S*,5'*R*,9a*R*,10a*R*,10b*S*,*E*)-2'-(4-chlorophenyl)-6,9a-dimethyl-2-oxo-3a,4,5,8,9,9a,10a,10b-octahydro-2*H*-spiro[oxireno[2',3':9,10]cyclodeca[1,2-*b*]furan-3,3'-pyrrolidine]-5'-carboxylate

The title product compound **34** was prepared in 0.10 mmol scale using **Condition C** and isolated by column chromatography (3:1 *n*-pentane: EA) giving an amorphous solid (27.0 mg, 0.06 mmol, 59% yield).

^{1}H NMR (500 MHz, CDCl$_3$) δ 7.39-7.32 (m, 4H), 4.86 – 4.81 (m, 2H), 4.15 (t, *J* = 8.7 Hz, 1H), 3.79 (s, 3H), 3.71 (t, *J* = 9.4 Hz, 1H), 2.46 – 2.40 (m, 2H), 2.29 – 2.19 (m, 2H), 2.10 – 2.03 (m, 2H), 2.00 – 1.92 (m, 2H), 1.67 (dd, *J* = 9.7, 7.8 Hz, 1H), 1.57 (s, 3H), 1.22 – 1.19 (m, 4H), 1.10 (td, *J* = 12.9, 6.0 Hz, 1H).

^{13}C NMR (126 MHz, CDCl$_3$) δ 178.7, 172.7, 137.1, 134.6, 134.0, 129.0, 128.9, 124.7, 81.7, 66.7, 66.6, 61.8, 57.6, 56.2, 52.5, 48.3, 40.4, 36.9, 33.1, 26.0, 23.9, 17.1.

HRMS(ESI): $[M+H]^+$ calcd. $C_{25}H_{31}NO_5Cl$ m/z 460.1885, found 460.1880.

IR (film, cm^{-1}): 2928, 2858, 1760, 1739, 1491, 1438, 1386, 1333, 1277, 1203, 1089, 1072, 1013, 976, 941, 910, 872, 824.

$[\alpha]_D^{20}$ = +28.2 (c = 0.11, $CHCl_3$)

Key ^{1}H & ^{13}C NMR signals — Key NOE signals

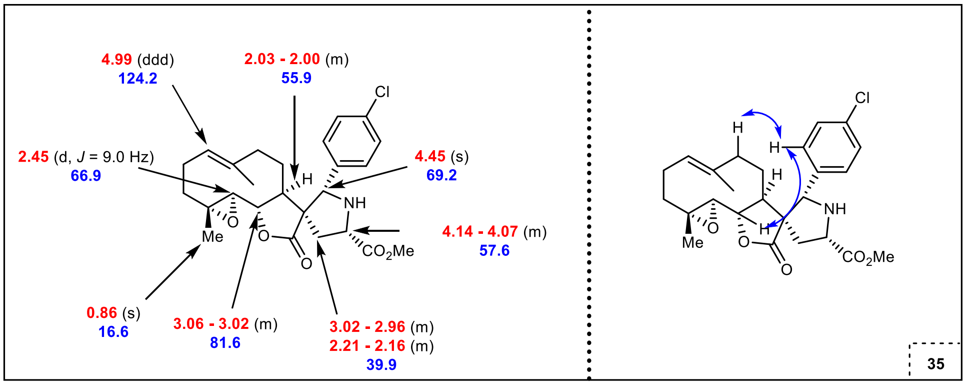

Methyl (2'*S*,3*S*,3a*S*,5'*S*,9a*R*,10a*R*,10b*S*,*E*)-2'-(4-chlorophenyl)-6,9a-dimethyl-2-oxo-3a,4,5,8,9,9a,10a,10b-octahydro-2*H*-spiro[oxireno[2',3':9,10]cyclodeca[1,2-*b*]furan-3,3'-pyrrolidine]-5'-carboxylate

The title product compound **35** was prepared in 0.10 mmol scale using **Condition D** and isolated by column chromatography (3:1 *n*-pentane: EA) giving an amorphous solid (15.1 mg, 0.03 mmol, 33% yield).

^{1}H NMR (500 MHz, CDCl$_3$) δ 7.45 (d, *J* = 8.2 Hz, 2H), 7.30 (d, *J* = 8.3 Hz, 2H), 4.99 (ddd, *J* = 12.0, 3.8, 1.8 Hz, 1H), 4.45 (s, 1H), 4.14 – 4.07 (m, 1H), 3.81 (s, 3H), 3.07 – 2.96 (m, 2H), 2.66 (dd, *J* = 15.5, 7.3 Hz, 1H), 2.45 (d, *J* = 9.0 Hz, 1H), 2.28 – 2.16 (m, 3H), 2.11 – 1.99 (m, 3H), 1.73 (t, *J* = 12.4 Hz, 1H), 1.45 (s, 3H), 1.14 – 1.01 (m, 2H), 0.86 (s, 3H).

^{13}C NMR (126 MHz, CDCl$_3$) δ 179.4, 173.1, 135.7, 134.9, 134.3, 129.2, 128.4, 124.2, 81.6, 69.2, 66.9, 61.5, 57.6, 57.3, 55.9, 52.5, 42.4, 39.9, 36.9, 23.8, 23.0, 17.0, 16.6.

HRMS(ESI): [M+H]$^{+}$ calcd. $C_{25}H_{31}NO_5Cl$ m/z 460.1885, found 460.1882.

IR (film, cm^{-1}): 2923, 2854, 1736, 1461, 1411, 1148, 1056, 967, 884, 811.

[α]$_D^{20}$ = +17.8 (c = 0.14, CHCl$_3$)

Key ^{1}H & ^{13}C NMR signals — Key NOE signals

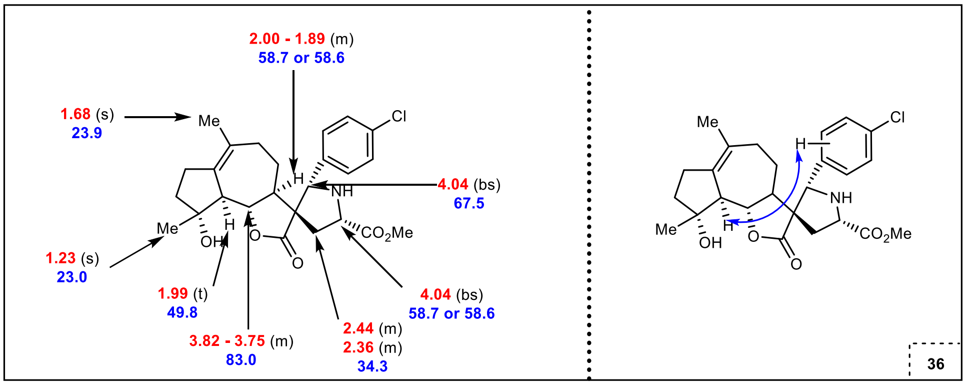

Methyl (2'*S*,3*R*,3a*S*,5'*S*,9*R*,9a*S*,9b*S*)-2'-(4-chlorophenyl)-9-hydroxy-6,9-dimethyl-2-oxo-3a,4,5,7,8,9,9a,9b-octahydro-2*H*-spiro[azuleno[4,5-*b*]furan-3,3'-pyrrolidine]-5'-carboxylate

The title product compound **36** was prepared in 0.10 mmol scale using **Condition A** and isolated by column chromatography (20:1 DCM: MeOH) giving an amorphous solid (44.5 mg, 0.10 mmol, 97% yield).

^{1}H NMR (500 MHz, CDCl$_3$) δ 7.36 – 7.28 (m, 4H), 4.08-3.99 (m, 2H), 3.82 – 3.75 (m, 4H), 2.51 – 2.24 (m, 5H), 2.21 – 2.09 (m, 2H), 1.99 (t, J = 11.0 Hz, 1H), 1.82 (d, J = 12.6 Hz, 1H), 1.74 – 1.64 (m, 5H), 1.42 – 1.32 (m, 1H), 1.23 (s, 3H).

^{13}C NMR (126 MHz, CDCl$_3$) δ 176.8, 172.7, 134.5, 133.9, 132.2, 131.3, 129.9, 128.8, 83.0, 80.3, 67.5, 58.7, 58.6, 55.5, 52.7, 49.8, 38.5, 35.3, 34.3, 30.1, 23.9, 23.8, 23.0.

HRMS(ESI): [M+H]$^+$ calcd. $C_{25}H_{31}NO_5Cl$ m/z 460.1885, found 460.1880.

IR (film, cm^{-1}): 2923, 2851, 1765, 1644, 1492, 1438, 1377, 1312, 1215, 1177, 1138, 1090, 1014, 990, 911, 873, 832.

[α]$_D^{20}$ = +21.5 (c = 0.14, CHCl$_3$)

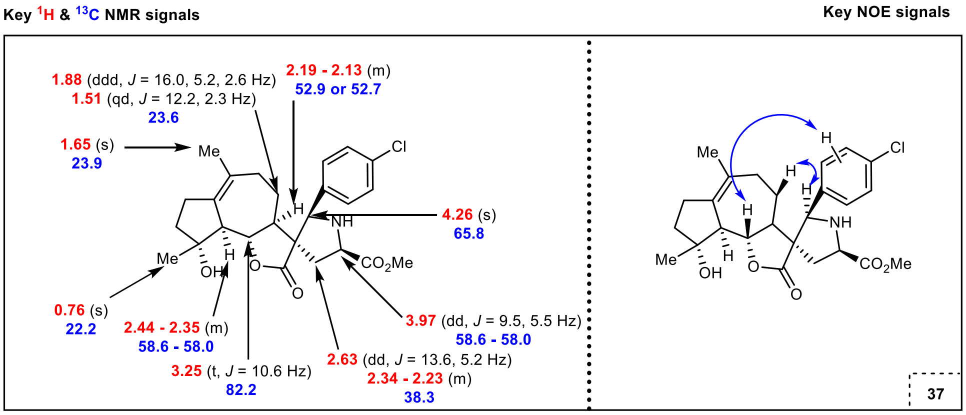

Methyl (2'*R*,3*S*,3a*S*,5'*R*,9*R*,9a*S*,9b*S*)-2'-(4-chlorophenyl)-9-hydroxy-6,9-dimethyl-2-oxo-3a,4,5,7,8,9,9a,9b-octahydro-2*H*-spiro[azuleno[4,5-*b*]furan-3,3'-pyrrolidine]-5'-carboxylate

The title product compound **37** was prepared in 0.10 mmol scale using **Condition B** and isolated by column chromatography (1:1 *n*-pentane: EA) giving an amorphous solid (42.7 mg, 0.09 mmol, 93% yield).

^{1}H NMR (500 MHz, CDCl$_3$) δ 7.40 – 7.29 (m, 4H), 4.26 (s, 1H), 3.97 (dd, J = 9.5, 5.5 Hz, 1H), 3.83 (s, 3H), 3.25 (t, J = 10.6 Hz, 1H), 2.63 (dd, J = 13.6, 5.2 Hz, 1H), 2.44 – 2.35 (m, 1H), 2.34 – 2.23 (m, 3H), 2.19 – 2.13 (m, 1H), 2.12 – 1.98 (m, 2H), 1.88 (ddd, J = 16.0, 5.2, 2.6 Hz, 1H), 1.71 – 1.59 (m, 5H), 1.51 (q, J = 12.3 Hz, 1H), 0.76 (s, 3H).

^{13}C NMR (126 MHz, CDCl$_3$) δ 178.0, 173.4, 136.1, 134.7, 132.2, 131.3, 129.5, 129.2, 82.2, 80.3, 65.8, 58.6, 58.4, 58.0, 52.9, 52.7, 38.3, 38.3, 35.6, 30.0, 23.9, 23.6, 22.2.

HRMS(ESI): [M+H]$^+$ calcd. $C_{25}H_{31}NO_5Cl$ m/z 460.1885, found 460.1880.

IR (film, cm^{-1}): 2934, 2854, 2247, 1760, 1491, 1436, 1376, 1316, 1202, 1181, 1136, 1111, 1091, 1074, 1014, 988, 909, 877, 825.

[α]$_D^{20}$ = +46.9 (c = 0.13, CHCl$_3$)

Key ^{1}H & ^{13}C NMR signals — Key NOE signals

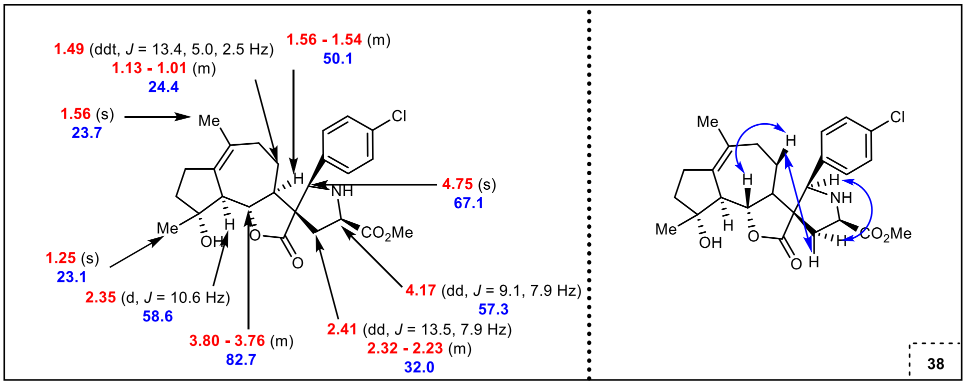

Methyl (2'*R*,3*R*,3a*S*,5'*R*,9*R*,9a*S*,9b*S*)-2'-(4-chlorophenyl)-9-hydroxy-6,9-dimethyl-2-oxo-3a,4,5,7,8,9,9a,9b-octahydro-2*H*-spiro[azuleno[4,5-*b*]furan-3,3'-pyrrolidine]-5'-carboxylate

The title product compound **38** was prepared in 0.10 mmol scale using **Condition C** and isolated by column chromatography (2:1 *n*-pentane: EA) giving an amorphous solid (44.0 mg, 0.10 mmol, 96% yield).

^{1}H NMR (500 MHz, $CDCl_3$) δ 7.38 – 7.29 (m, 4H), 4.75 (s, 1H), 4.17 (dd, *J* = 9.1, 7.9 Hz, 1H), 3.80 – 3.76 (m, 4H), 2.41 (dd, *J* = 13.5, 7.9 Hz, 1H), 2.35 (d, *J* = 10.6 Hz, 1H), 2.32 – 2.23 (m, 2H), 2.13 – 2.03 (m, 1H), 1.93 (ddd, *J* = 15.9, 5.4, 2.4 Hz, 1H), 1.72 – 1.54 (m, 7H), 1.52-1.46 (m, 1H), 1.25 (s, 3H), 1.13 – 1.01 (m, 1H).

^{13}C NMR (126 MHz, $CDCl_3$) δ 179.1, 173.2, 137.4, 133.9, 131.9, 131.4, 128.8, 128.7, 82.7, 80.4, 67.1, 58.6, 57.3, 55.0, 52.4, 50.1, 38.5, 34.8, 32.0, 29.9, 24.4, 23.7, 23.1.

HRMS(ESI): $[M+H]^+$ calcd. $C_{25}H_{31}NO_5Cl$ m/z 460.1885, found 460.1882.

IR (film, cm^{-1}): 2924, 2349, 2117, 1744, 1490, 1437, 1376, 1303, 1212, 1172, 1137, 1088, 1013, 989, 910, 877, 835.

$[\alpha]_D^{20}$ = +25.6 (c = 0.20, $CHCl_3$)

Key ^{1}H & ^{13}C NMR signals — Key NOE signals

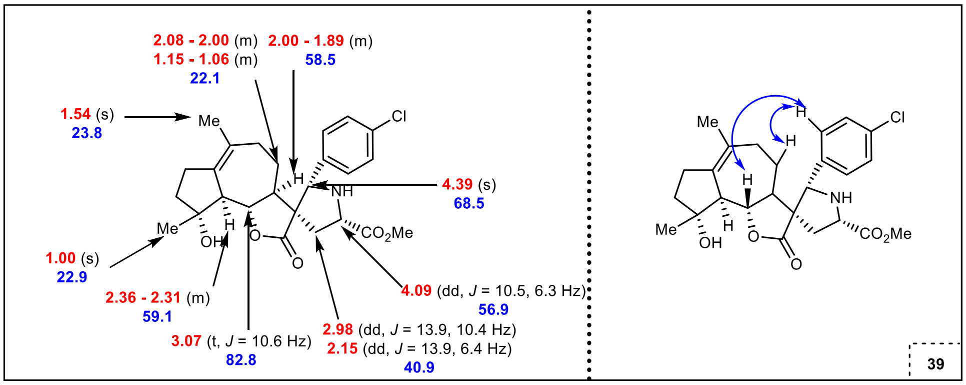

Methyl (2'*S*,3*S*,3a*S*,5'*S*,9*R*,9a*S*,9b*S*)-2'-(4-chlorophenyl)-9-hydroxy-6,9-dimethyl-2-oxo-3a,4,5,7,8,9,9a,9b-octahydro-2*H*-spiro[azuleno[4,5-*b*]furan-3,3'-pyrrolidine]-5'-carboxylate

The title product compound **39** was prepared in 0.10 mmol scale using **Condition D** and isolated by column chromatography (1:1 *n*-pentane: EA) giving an amorphous solid (42.9 mg, 0.09 mmol, 93% yield).

^{1}H NMR (500 MHz, CDCl$_3$) δ 7.38 (d, *J* = 8.2 Hz, 2H), 7.29 (d, *J* = 8.5 Hz, 2H), 4.39 (s, 1H), 4.09 (dd, *J* = 10.5, 6.3 Hz, 1H), 3.80 (s, 3H), 3.07 (t, *J* = 10.6 Hz, 1H), 2.98 (dd, *J* = 13.9, 10.4 Hz, 1H), 2.36 – 2.31 (m, 1H), 2.24 (dd, *J* = 16.5, 7.8 Hz, 1H), 2.15 (dd, *J* = 13.9, 6.4 Hz, 1H), 2.08 – 1.89 (m, 4H), 1.82 (t, *J* = 13.2 Hz, 1H), 1.72 – 1.60 (m, 2H), 1.54 (s, 3H), 1.15 – 1.06 (m, 1H), 1.00 (s, 3H).

^{13}C NMR (126 MHz, CDCl$_3$) δ 179.5, 173.4, 135.8, 134.0, 131.9, 131.3, 128.6, 128.5, 82.7, 80.4, 68.5, 59.1, 58.5, 56.9, 56.2, 52.5, 40.9, 38.5, 36.7, 29.8, 23.8, 22.9, 22.1.

HRMS(ESI): $[M+H]^+$ calcd. $C_{25}H_{31}NO_5Cl$ m/z 460.1885, found 460.1880.

IR (film, cm^{-1}): 2950, 1742, 1492, 1437, 1376, 1318, 1303, 1210, 1137, 1113, 1090, 1074, 1012, 987, 908, 883, 829.

$[\alpha]_D^{20}$ = +40.6 (c = 0.17, $CHCl_3$)

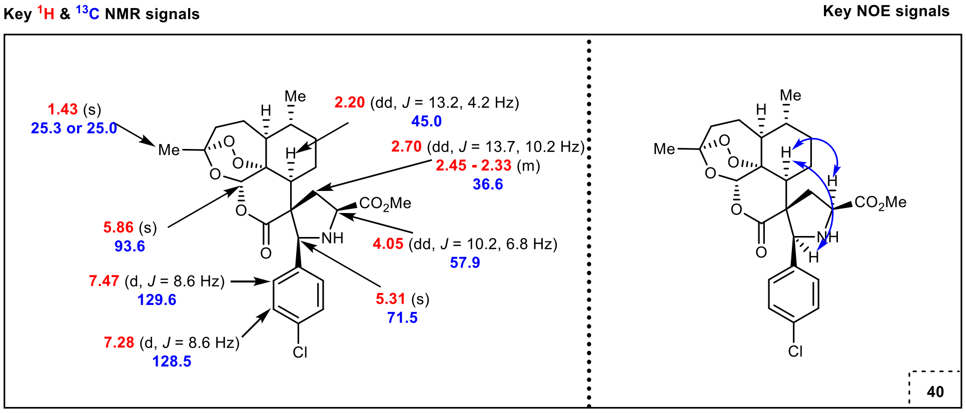

Methyl (2*S*,3*R*,3'*R*,5*S*,5a'*S*,6'*R*,8a'*S*,12'*S*,12a'*R*)-2-(4-chlorophenyl)-3',6'-dimethyl-10'-oxooctahydro-10'*H*,12'*H*-spiro[pyrrolidine-3,9'-[3,12]epoxy[1,2]dioxepino[4,3-*i*]isochromene]-5-carboxylate

The title product compound **40** was prepared from substrate **17** (14.0 mg, 0.05 mmol) using **Condition A** and isolated by column chromatography (1:1 *n*-pentane: EA) giving an amorphous solid (13.3 mg, 0.03 mmol, 54% yield).

^{1}H NMR (500 MHz, CDCl$_3$) δ 7.47 (d, *J* = 8.6 Hz, 2H), 7.28 (d, *J* = 8.5 Hz, 2H), 5.86 (s, 1H), 5.31 (s, 1H), 4.05 (dd, *J* = 10.2, 6.8 Hz, 1H), 3.83 (s, 3H), 2.70 (dd, *J* = 13.7, 10.2 Hz, 1H), 2.45 – 2.33 (m, 2H), 2.20 (dd, *J* = 13.2, 4.2 Hz, 1H), 2.09 – 1.98 (m, 3H), 1.85 – 1.78 (m, 1H), 1.56 – 1.48 (m, 2H), 1.43 (s, 3H), 1.41 – 1.36 (m, 1H), 1.27 – 1.22 (m, 1H), 1.21 – 1.14 (m, 1H), 1.01 (d, *J* = 6.3 Hz, 3H).

^{13}C NMR (126 MHz, CDCl$_3$) δ 173.2, 169.8, 138.7, 133.7, 129.6, 128.5, 105.5, 93.6, 80.6, 71.5, 57.9, 56.3, 53.0, 50.9, 45.0, 37.6, 36.6, 35.9, 34.0, 26.1, 25.3, 25.0, 20.0.

HRMS(ESI): $[M+H]^+$ calcd. $C_{25}H_{31}NO_7Cl$ m/z 492.1784, found 492.1779.

IR (film, cm^{-1}): 2926, 2112, 1733, 1633, 1494, 1437, 1377, 1205, 1154, 1138, 1110, 1091, 1052, 1035, 1011, 991, 971, 935, 880, 835.

Key ^{1}H & ^{13}C NMR signals

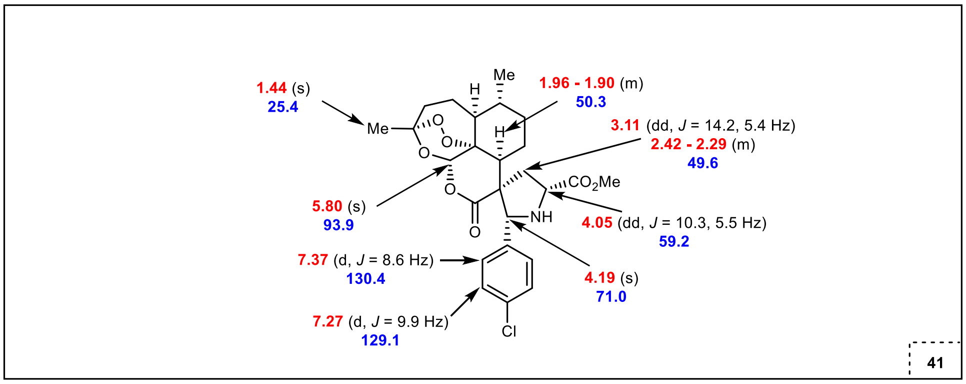

41

Methyl (2*R*,3*S*,3'*R*,5*R*,5a'*S*,6'*R*,8a'*S*,12'*S*,12a'*R*)-2-(4-chlorophenyl)-3',6'-dimethyl-10'-oxooctahydro-10'*H*,12'*H*-spiro[pyrrolidine-3,9'-[3,12]epoxy[1,2]dioxepino[4,3-*i*]isochromene]-5-carboxylate

The title product compound **41** was prepared from substrate **17** (14.0 mg, 0.05 mmol) using **Condition B** and isolated by column chromatography (20:1 DCM: MeOH) giving an amorphous solid (19.0 mg, 0.04 mmol, 77% yield).

^{1}H NMR (500 MHz, $CDCl_3$) δ 7.37 (d, *J* = 8.6 Hz, 2H), 7.27 (d, *J* = 9.9 Hz, 2H), 5.80 (s, 1H), 4.19 (s, 1H), 4.05 (dd, *J* = 10.3, 5.5 Hz, 1H), 3.79 (s, 3H), 3.11 (dd, *J* = 14.2, 5.4 Hz, 1H), 2.42 – 2.29 (m, 2H), 2.06 – 2.00 (m, 1H), 1.96 – 1.90 (m, 2H), 1.86 – 1.81 (m, 1H), 1.67 – 1.63 (m, 1H), 1.45 – 1.37 (m, 5H), 1.25 (t, *J* = 7.2 Hz, 1H), 1.00 – 0.91 (m, 5H).

^{13}C NMR (126 MHz, $CDCl_3$) δ 173.5, 172.7, 136.2, 134.3, 130.4, 129.1, 105.5, 93.9, 80.8, 71.0, 59.2, 53.2, 52.6, 50.3, 50.3, 49.6, 37.4, 36.0, 34.3, 27.3, 25.4, 25.0, 19.9.

HRMS(ESI): $[M+H]^+$ calcd. $C_{25}H_{31}NO_7Cl$ m/z 492.1784, found 492.1779.

IR (film, cm^{-1}): 2952, 1737, 1492, 1435, 1379, 1322, 1200, 1153, 1110, 1032, 1013, 992, 958, 908, 879, 832.

$[\alpha]_D^{20}$ = +52.0 (c = 0.13, $CHCl_3$)

Key ^{1}H & ^{13}C NMR signals

Key NOE signals

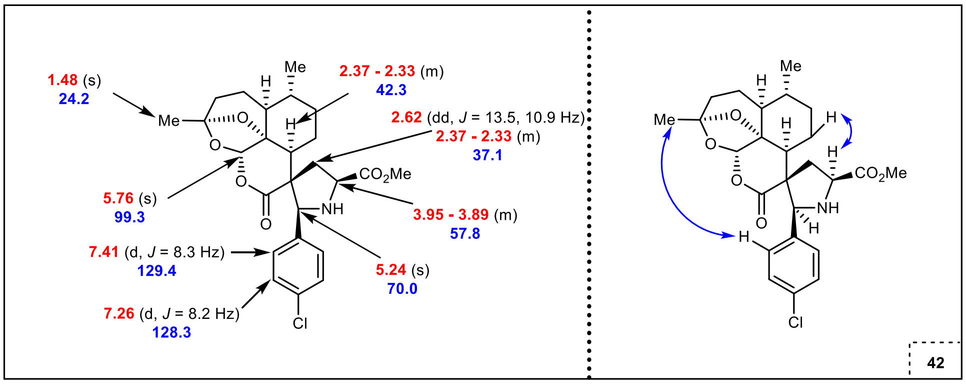

42

Methyl (2*S*,3*R*,3a'*S*,3a[1]'*R*,5*S*,6'*R*,6a'*S*,9'*S*,10a'*S*)-2-(4-chlorophenyl)-6',9'-dimethyl-2'-oxooctahydro-2'*H*,10a'*H*-spiro[pyrrolidine-3,3'-[3a[1],9]epoxyoxepino[4,3,2-*iJ*]isochromene]-5-carboxylate

The title product compound **42** was prepared from substrate **18** (13.2 mg, 0.05 mmol) using **Condition A** and isolated by column chromatography (1:1 *n*-pentane: EA) giving an amorphous solid (12.8 mg, 0.03 mmol, 54% yield).

^{1}H NMR (500 MHz, $CDCl_3$) δ 7.41 (d, *J* = 8.3 Hz, 2H), 7.26 (d, *J* = 8.2 Hz, 2H), 5.76 (s, 1H), 5.24 (s, 1H), 3.95 – 3.89 (m, 1H), 3.81 (s, 3H), 2.62 (dd, *J* = 13.5, 10.9 Hz, 1H), 2.37 – 2.33 (m, 2H), 2.09 – 2.01 (m, 1H), 1.94 (dt, *J* = 13.6, 5.1 Hz, 1H), 1.86 (dt, *J* = 9.9, 3.5 Hz, 1H), 1.77 – 1.71 (m, 1H), 1.63 (ddd, *J* = 13.6, 11.5, 5.7 Hz, 1H), 1.48 (s, 3H), 1.43 – 1.36 (m, 1H), 1.33 – 1.26 (m, 3H), 1.21 – 1.14 (m, 2H).

^{13}C NMR (126 MHz, $CDCl_3$) δ 174.1, 169.2, 140.5, 133.3, 129.4, 128.3, 110.3, 99.3, 83.1, 70.0, 57.8, 56.0, 52.7, 45.4, 42.3, 37.1, 35.7, 34.1, 34.0, 26.5, 24.2, 22.4, 18.9.

HRMS(ESI): $[M+H]^+$ calcd. $C_{25}H_{31}NO_6Cl$ m/z 476.1834, found 476.1829.

IR (film, cm^{-1}): 2924, 1733, 1493, 1437, 1387, 1281, 1209, 1189, 1153, 1124, 1092, 1019, 1004, 974, 939, 883, 856.

$[\alpha]_D^{20}$ = -123.7 (c = 0.14, $CHCl_3$)

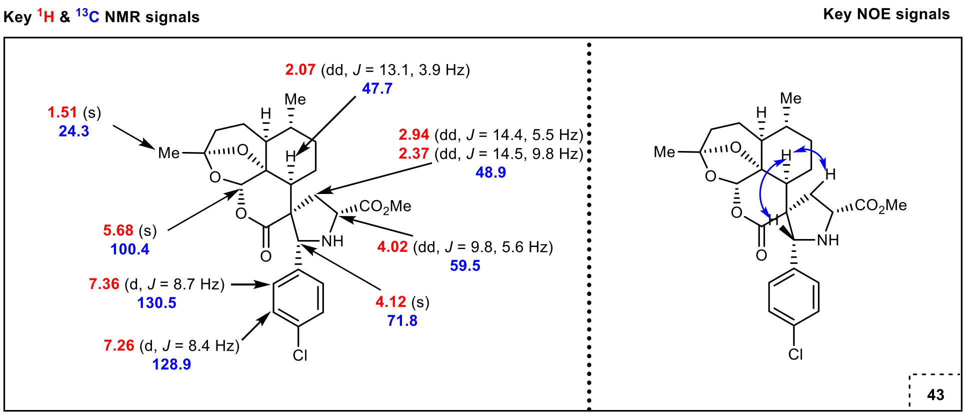

Methyl (2*R*,3*S*,3a'*S*,3a1'*R*,5*R*,6'*R*,6a'*S*,9'*S*,10a'*S*)-2-(4-chlorophenyl)-6',9'-dimethyl-2'-oxooctahydro-2'*H*,10a'*H*-spiro[pyrrolidine-3,3'-[3a1,9]epoxyoxepino[4,3,2-*iJ*]isochromene]-5-carboxylate

The title product compound **43** was prepared from substrate **18** (13.2 mg, 0.05 mmol) using **Condition B** and isolated by column chromatography (1:2 *n*-pentane: EA) giving an amorphous solid (16.6 mg, 0.03 mmol, 70% yield).

^{1}H NMR (500 MHz, $CDCl_3$) δ 7.36 (d, *J* = 8.7 Hz, 2H), 7.26 (d, *J* = 8.4 Hz, 2H), 5.68 (s, 1H), 4.12 (s, 1H), 4.02 (dd, *J* = 9.8, 5.6 Hz, 1H), 3.78 (s, 3H), 2.94 (dd, *J* = 14.4, 5.5 Hz, 1H), 2.37 (dd, *J* = 14.5, 9.8 Hz, 1H), 2.07 (dd, *J* = 13.1, 3.9 Hz, 1H), 1.91 – 1.83 (m, 1H), 1.83 – 1.76 (m, 1H), 1.76 – 1.64 (m, 2H), 1.64 – 1.54 (m, 1H), 1.51 (s, 3H), 1.26 – 1.14 (m, 6H), 1.06 – 0.92 (m, 2H), 0.88 (d, *J*=6.2 Hz, 3H).

^{13}C NMR (126 MHz, $CDCl_3$) δ 174.6, 172.9, 136.6, 134.2, 130.5, 128.9, 110.3, 100.4, 82.7, 71.8, 59.5, 53.0, 52.6, 48.9, 47.7, 44.7, 35.5, 34.3, 33.7, 27.9, 24.3, 22.4, 18.7.

HRMS(ESI): $[M+H]^+$ calcd. $C_{25}H_{31}NO_6Cl$ m/z 476.1834, found 476.1829.

IR (film, cm^{-1}): 2924, 2853, 1738, 1492, 1435, 1388, 1323, 1267, 1209, 1180, 1146, 1130, 1095, 1068, 1011, 983, 944, 886, 855, 822.

$[\alpha]_D^{20}$ = -54.5 (c = 0.11, $CHCl_3$)

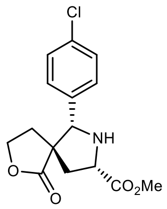

44

Methyl (5*R*,6*S*,8*S*)-6-(4-chlorophenyl)-1-oxo-2-oxa-7-azaspiro[4.4]nonane-8-carboxylate

The title product compound **44** was prepared in 0.10 mmol scale using **Condition A** and isolated by column chromatography (20:1 DCM: MeOH) giving an amorphous solid (27.5 mg, 0.09 mmol, 89% yield).

^{1}H NMR (400 MHz, $CDCl_3$) δ 7.40 – 7.28 (m, 4H), 4.19 (s, 1H), 4.09 (tt, *J* = 8.0, 4.0 Hz, 2H), 3.83 (s, 3H), 3.61 (td, *J* = 9.5, 7.0 Hz, 1H), 2.70 (dd, *J* = 13.4, 5.1 Hz, 1H), 2.46 (dt, *J* = 13.3, 9.4 Hz, 1H), 2.33 (ddd, *J* = 15.6, 8.3, 3.9 Hz, 2H).

^{13}C NMR (101 MHz, $CDCl_3$) δ 178.4, 173.2, 135.3, 135.0, 129.3, 128.8, 72.4, 65.4, 59.0, 53.7, 52.7, 40.3, 33.2.

$[\alpha]_D^{20}$ = +16.1 (c = 0.83, CH_2Cl_2)

HPLC conditions: CHIRAPAK IC column, *iso*-propanol / *iso*-hexane = 40/60, flow rate = 0.5 mL min^{-1}, major enantiomer: t_R = 24.9 min; minor enantiomer: t_R = 41.5 min. *ee* = 96%.

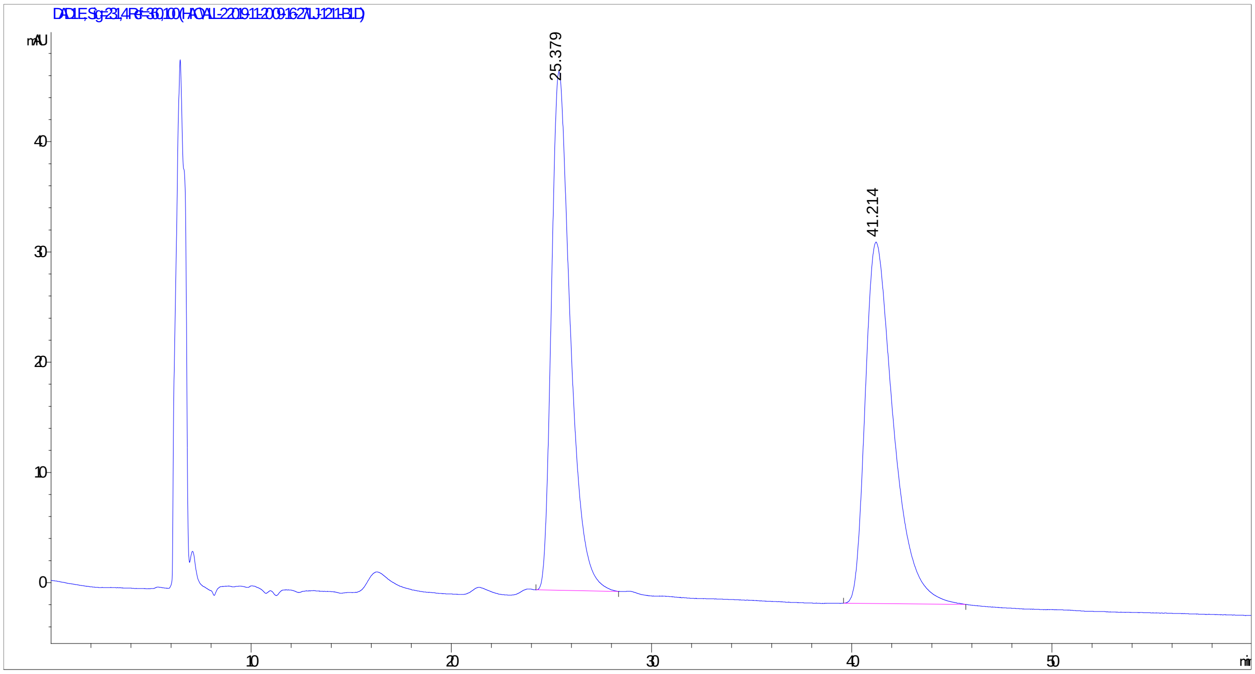

#	Time	Area	Height	Width	Area%	Symmetry
1	25.379	3104.8	47.1	0.9699	49.042	0.614
2	41.214	3226.1	32.8	1.3599	50.958	0.569

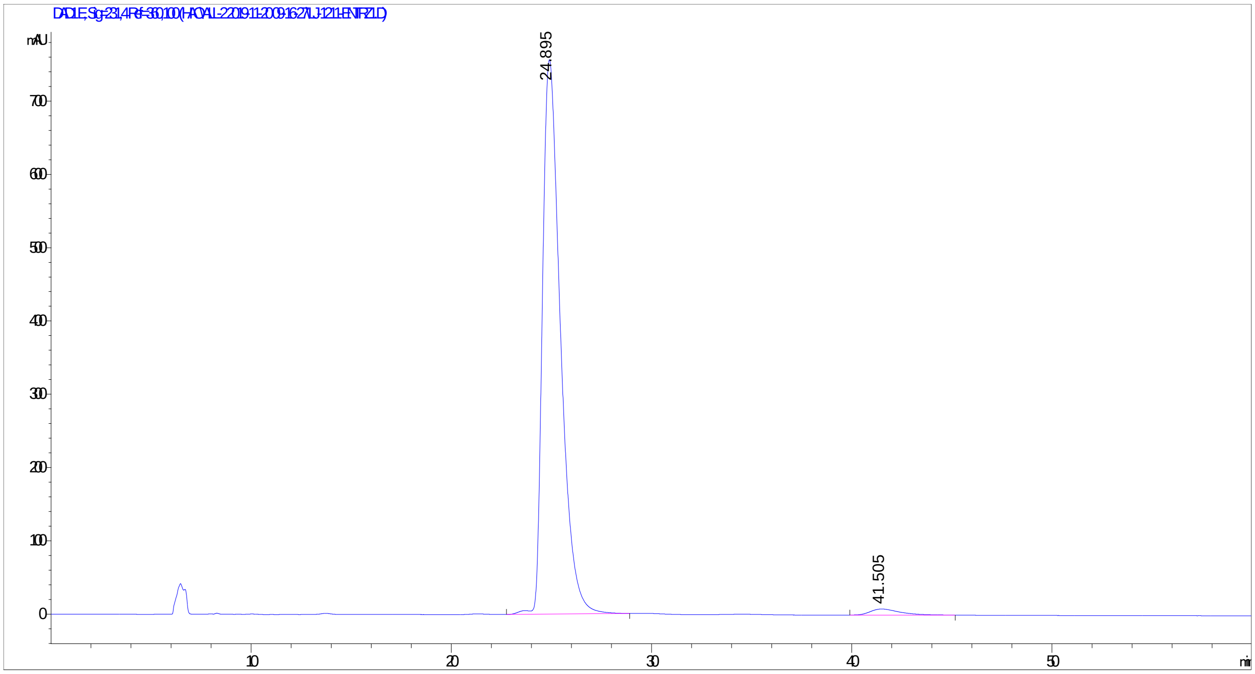

#	Time	Area	Height	Width	Area%	Symmetry
1	24.895	47201.6	756.6	0.9518	98.215	0.579
2	41.505	857.8	8.3	1.2486	1.785	0.627

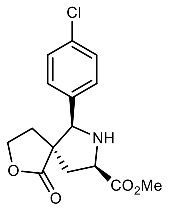

45

Methyl (5*S*,6*R*,8*R*)-6-(4-chlorophenyl)-1-oxo-2-oxa-7-azaspiro[4.4]nonane-8-carboxylate

The title product compound **45** was prepared in 0.10 mmol scale using **Condition B** and isolated by column chromatography (20:1 DCM: MeOH) giving an amorphous solid (27.9 mg, 0.09 mmol, 90% yield).

^{1}H NMR (400 MHz, $CDCl_3$) δ 7.40 – 7.28 (m, 4H), 4.19 (s, 1H), 4.09 (tt, *J* = 8.0, 4.0 Hz, 2H), 3.83 (s, 3H), 3.61 (td, *J* = 9.5, 7.0 Hz, 1H), 2.70 (dd, *J* = 13.4, 5.1 Hz, 1H), 2.46 (dt, *J* = 13.3, 9.4 Hz, 1H), 2.33 (ddd, *J* = 15.6, 8.3, 3.9 Hz, 2H).

^{13}C NMR (101 MHz, $CDCl_3$) δ 178.4, 173.2, 135.3, 135.0, 129.3, 128.8, 72.4, 65.4, 59.0, 53.7, 52.7, 40.3, 33.2.

$[\alpha]_D^{20}$ = -13.5 (c = 0.73, CH_2Cl_2)

HPLC conditions: CHIRAPAK IC column, *iso*-propanol / *iso*-hexane = 40/60, flow rate = 0.5 mL min^{-1}, major enantiomer: t_R = 40.9 min; minor enantiomer: t_R = 25.6 min. *ee* = 95%.

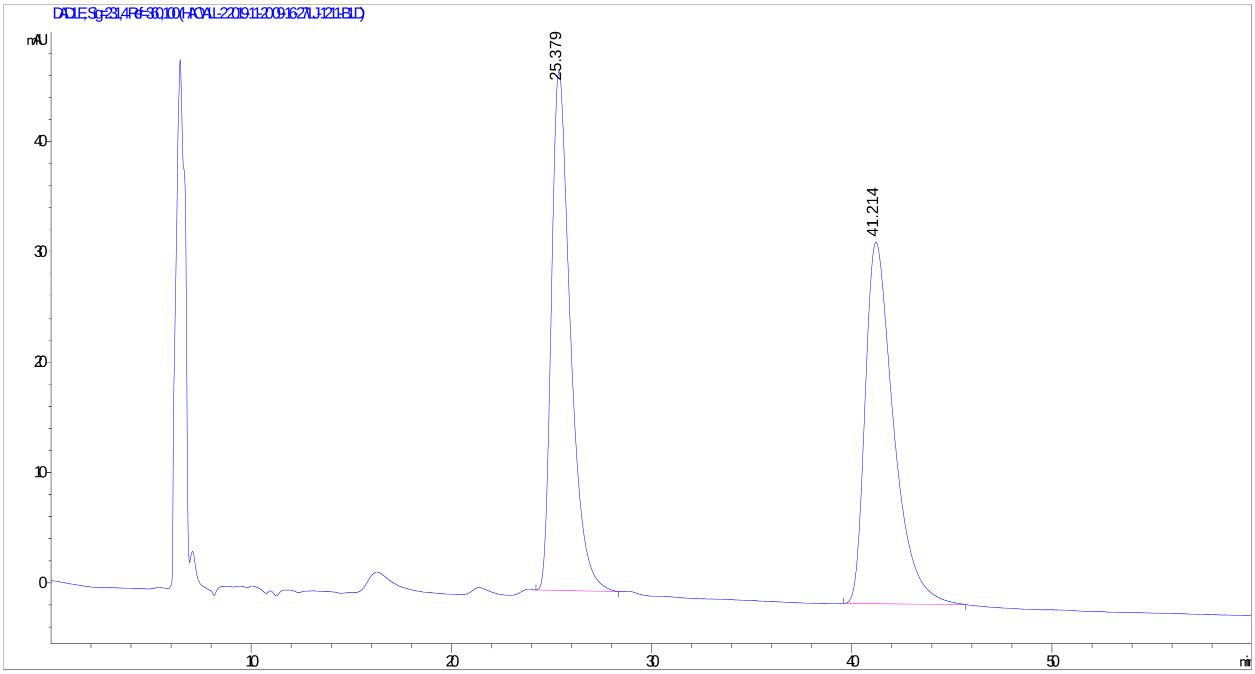

#	Time	Area	Height	Width	Area%	Symmetry
1	25.379	3104.8	47.1	0.9699	49.042	0.614
2	41.214	3226.1	32.8	1.3599	50.958	0.569

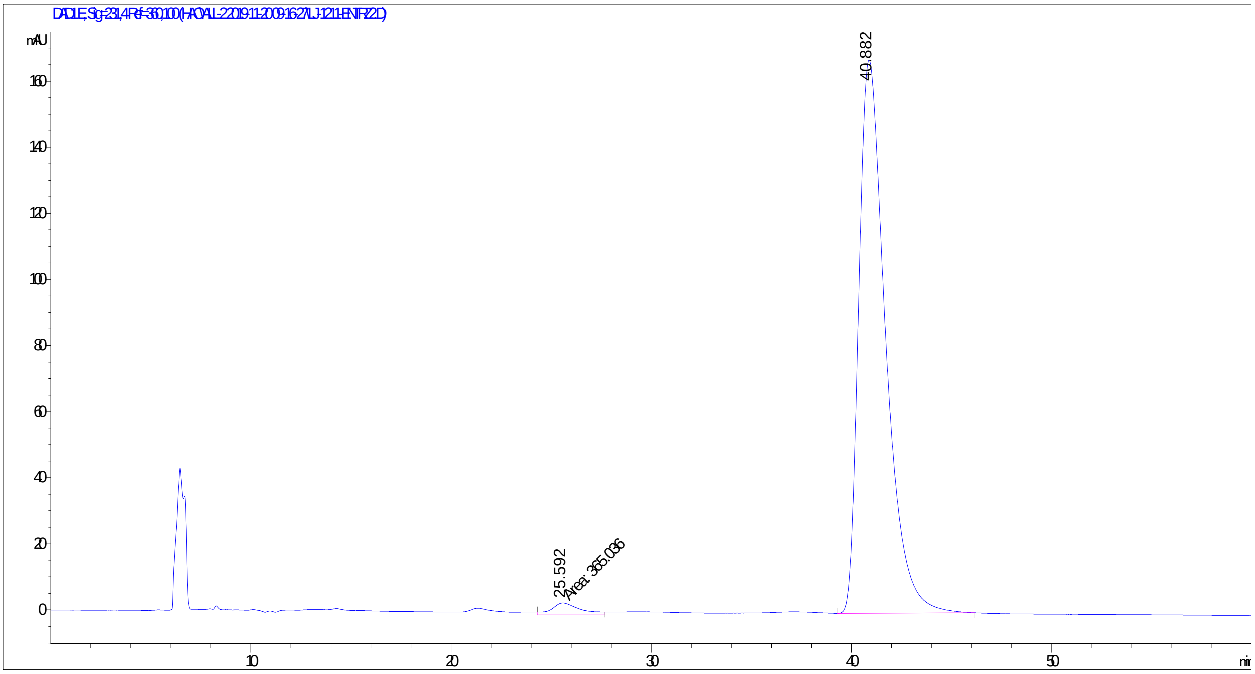

#	Time	Area	Height	Width	Area%	Symmetry
1	25.592	365	3.6	1.6901	2.291	0.656
2	40.882	15568.8	167.5	1.4044	97.709	0.574

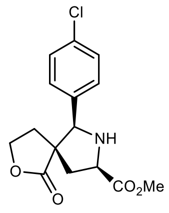

46

Methyl (5*R*,6*R*,8*R*)-6-(4-chlorophenyl)-1-oxo-2-oxa-7-azaspiro[4.4]nonane-8-carboxylate

The title product compound **46** was prepared in 0.10 mmol scale using **Condition C** and isolated by column chromatography (1:2 *n*-pentane: EA) giving an amorphous solid (25.1 mg, 0.08 mmol, 81% yield).

^{1}H NMR (400 MHz, $CDCl_3$) δ 7.42 – 7.29 (m, 4H), 4.61 (s, 1H), 4.15 (dd, *J* = 10.0, 4.7 Hz, 1H), 4.06 – 3.95 (m, 1H), 3.79 (s, 3H), 3.37 (ddd, *J* = 8.8, 7.9, 6.5 Hz, 1H), 2.76 (dd, *J* = 13.3, 10.0 Hz, 1H), 2.26 (dd, *J* = 13.3, 4.7 Hz, 1H), 2.06 (ddd, *J* = 13.7, 7.9, 5.9 Hz, 1H), 1.90 (ddd, *J* = 13.4, 8.1, 6.5 Hz, 1H).

^{13}C NMR (101 MHz, $CDCl_3$) δ 179.8, 174.1, 136.3, 134.3, 128.9, 128.5, 68.0, 66.0, 57.2, 52.7, 52.5, 40.6, 31.0.

$[\alpha]_D^{20}$ = +20.6 (c = 0.16, $CHCl_3$)

HPLC conditions: CHIRAPAK IC column, *iso*-propanol / *iso*-hexane = 40/60, flow rate = 0.5 mL min^{-1}, major enantiomer: t_R = 24.4 min; minor enantiomer: t_R = 28.6 min. *ee* = 97%.

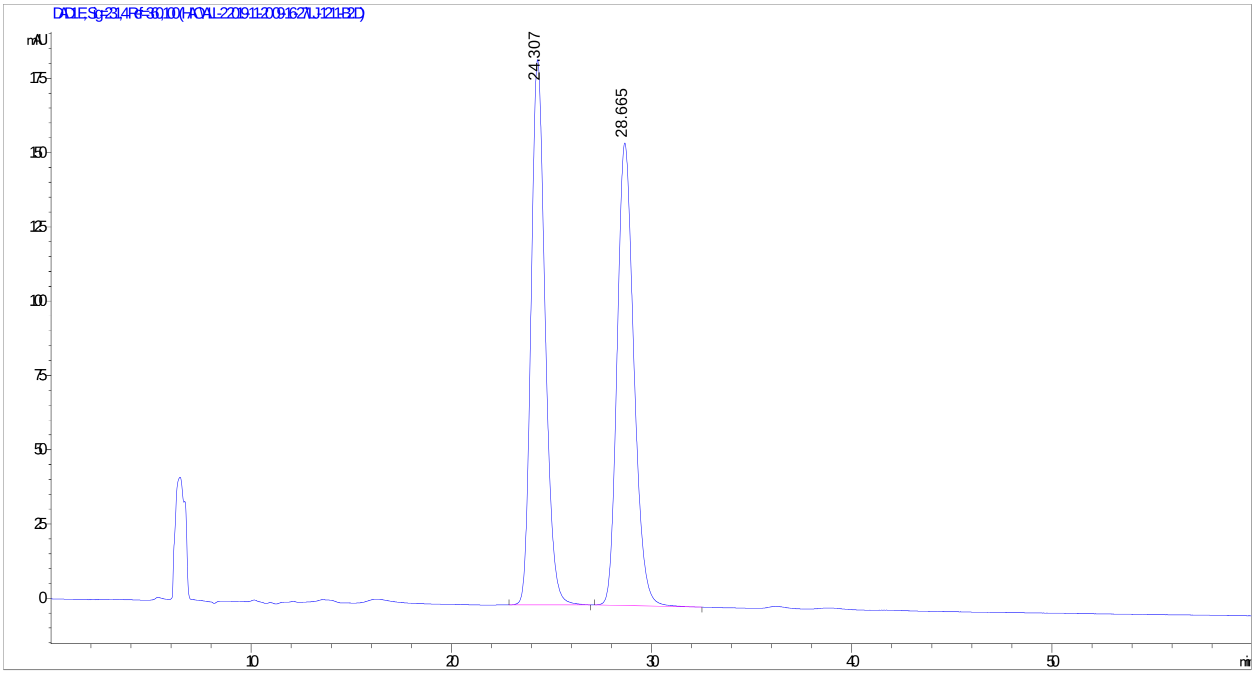

#	Time	Area	Height	Width	Area%	Symmetry
1	24.307	8995.1	183.5	0.7618	49.855	0.799
2	28.665	9047.3	155.7	0.894	50.145	0.784

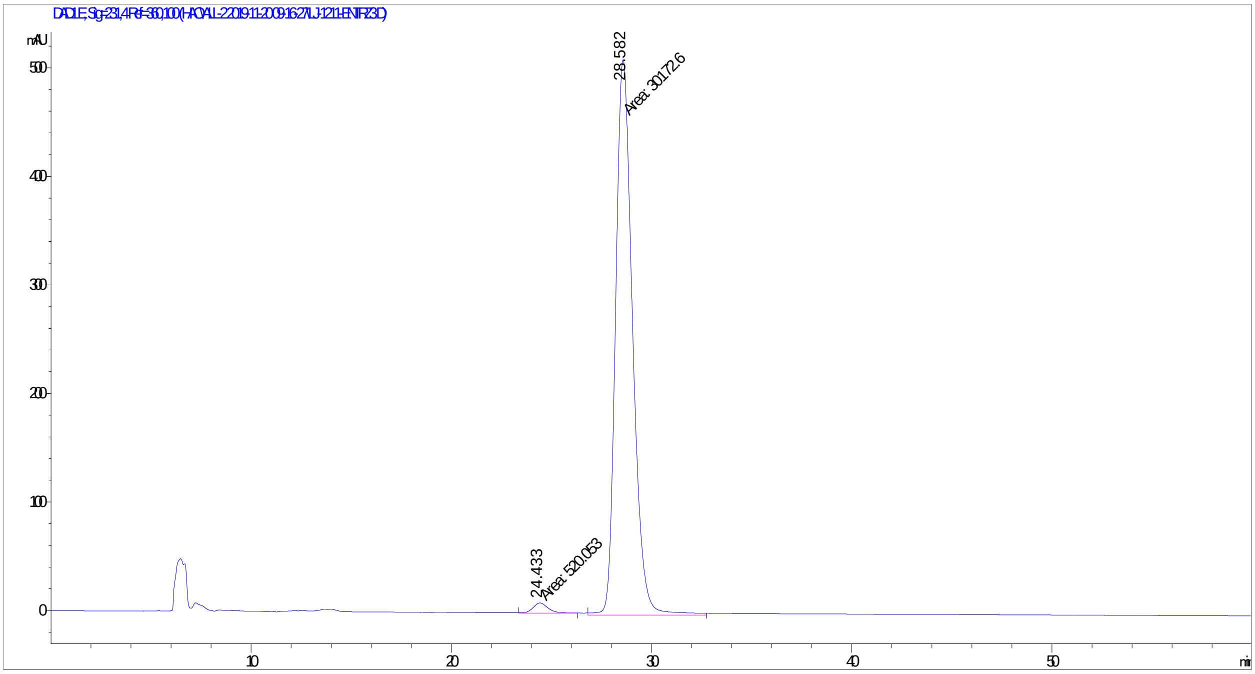

#	Time	Area	Height	Width	Area%	Symmetry
1	24.433	520.1	9.5	0.9118	1.694	0.826
2	28.582	30172.6	511.7	0.9828	98.306	0.752

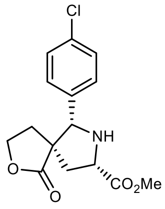

47

Methyl (5*S*,6*S*,8*S*)-6-(4-chlorophenyl)-1-oxo-2-oxa-7-azaspiro[4.4]nonane-8-carboxylate

The title product compound **47** was prepared in 0.10 mmol scale using **Condition D** and isolated by column chromatography (1:2 *n*-pentane: EA) giving an amorphous solid (24.2 mg, 0.08 mmol, 78% yield).

^{1}H NMR (400 MHz, $CDCl_3$) δ 7.42 – 7.29 (m, 4H), 4.61 (s, 1H), 4.15 (dd, *J* = 10.0, 4.7 Hz, 1H), 4.06 – 3.95 (m, 1H), 3.79 (s, 3H), 3.37 (ddd, *J* = 8.8, 7.9, 6.5 Hz, 1H), 2.76 (dd, *J* = 13.3, 10.0 Hz, 1H), 2.26 (dd, *J* = 13.3, 4.7 Hz, 1H), 2.06 (ddd, *J* = 13.7, 7.9, 5.9 Hz, 1H), 1.90 (ddd, *J* = 13.4, 8.1, 6.5 Hz, 1H).

^{13}C NMR (101 MHz, $CDCl_3$) δ 179.8, 174.1, 136.3, 134.3, 128.9, 128.5, 68.0, 66.0, 57.2, 52.7, 52.5, 40.6, 31.0.

$[\alpha]_D^{20}$ = -33.2 (c = 0.19, $CHCl_3$)

HPLC conditions: CHIRAPAK IC column, *iso*-propanol / *iso*-hexane = 40/60, flow rate = 0.5 mL min^{-1}, major enantiomer: t_R = 24.3 min; minor enantiomer: t_R = 28.8 min. *ee* = 98%.

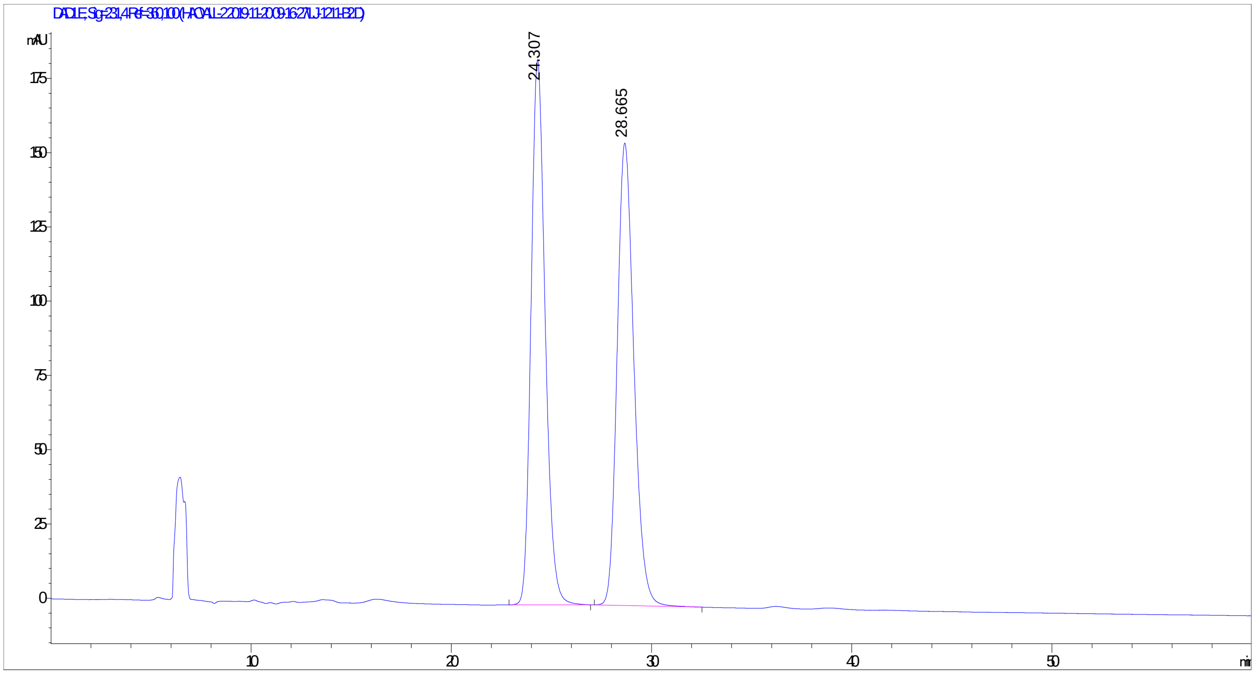

#	Time	Area	Height	Width	Area%	Symmetry
1	24.307	8995.1	183.5	0.7618	49.855	0.799
2	28.665	9047.3	155.7	0.894	50.145	0.784

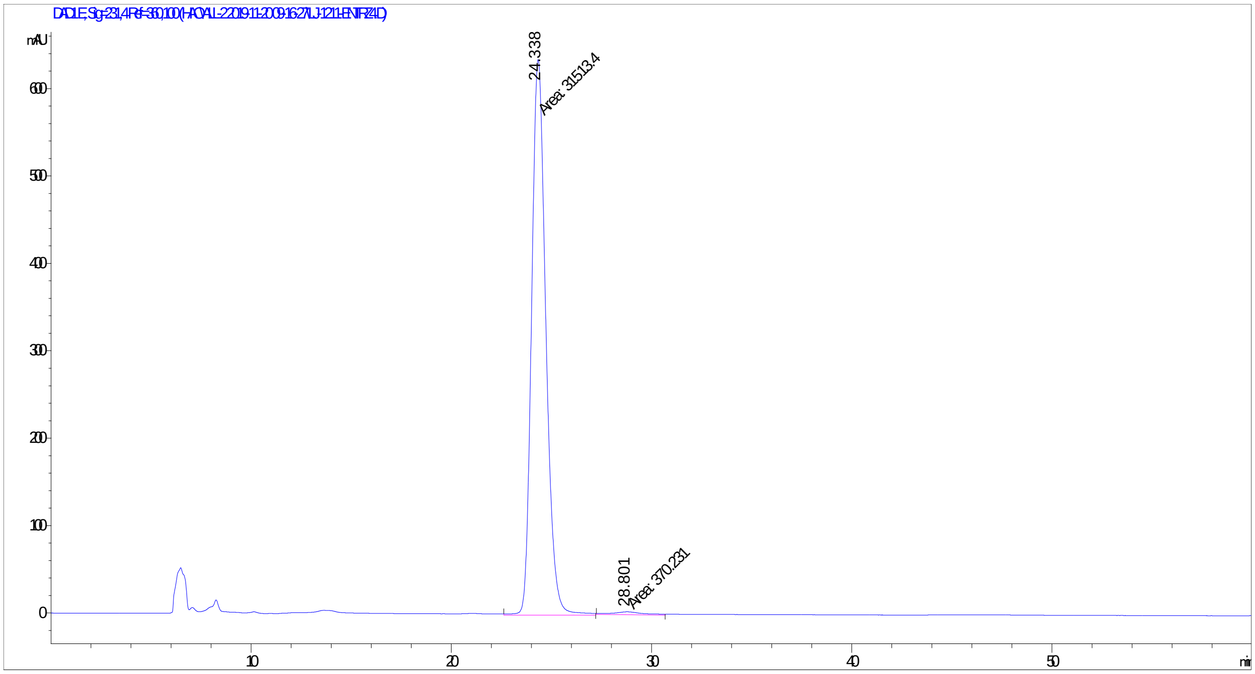

#	Time	Area	Height	Width	Area%	Symmetry
1	24.338	31513.4	635.6	0.8264	98.839	0.802
2	28.801	370.2	3.4	1.8404	1.161	0.867

5.4 Synthesis of 9b-Ts.

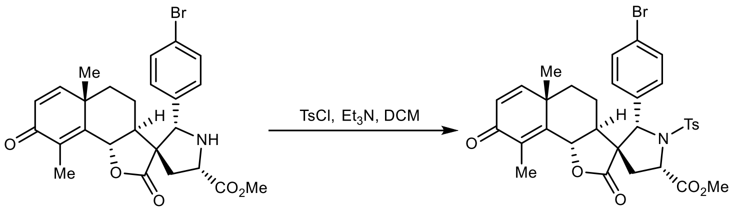

9b   9b-Ts

Methyl (2'*S*,3*R*,3a*S*,5a*S*,5'*S*,9b*S*)-2'-(4-bromophenyl)-5a,9-dimethyl-2,8-dioxo-1'-tosyl-3a,4,5,5a,8,9b-hexahydro-2*H*-spiro[naphtho[1,2-*b*]furan-3,3'-pyrrolidine]-5'-carboxylate

To a solution of **9b** (25.0 mg, 0.05 mmol) in DCM (1.0 mL) was added with $NaHCO_3$ (21.0 mg, 0.25 mmol) and TsCl (19.0 mg, 0.1 mmol). The reaction was stirred until full consumption of the starting material monitored by TLC. Then saturated $NaHCO_3$ solution (10 mL) was added to quench the reaction. The mixture was extracted with DCM (10 mL) for three times. And the resulting organic layers were combined, washed with brine and dried over Na_2SO_4. After evaporation, the residue was purified by flash column chromatography (*n*-pentane: ethyl acetate 3:1) giving a solid **9b-Ts** (25.0 mg, 0.04 mmol, 76% yield).

^{1}H NMR (600 MHz, $CDCl_3$) δ 7.47 (d, *J* = 7.9 Hz, 2H), 7.15 (d, *J* = 8.0 Hz, 2H), 7.09 (d, *J* = 7.9 Hz, 2H), 7.04 – 6.95 (m, 2H), 6.67 (d, *J* = 9.9 Hz, 1H), 6.24 (d, *J* = 9.9 Hz, 1H), 5.10 (dd, *J* = 8.9, 2.7 Hz, 1H), 4.82 – 4.72 (m, 2H), 3.82 (s, 3H), 2.71 (dd, *J* = 13.1, 2.7 Hz, 1H), 2.44 (dd, *J* = 13.1, 8.9 Hz, 1H), 2.38 (s, 3H), 2.07 (td, *J* = 12.2, 3.0 Hz, 1H), 1.97 (d, *J* = 14.1 Hz, 2H), 1.86 (d, *J* = 1.2 Hz, 3H), 1.77 (dd, *J* = 12.9, 3.7 Hz, 1H), 1.56 – 1.47 (m, 1H), 1.29 (s, 3H).

^{13}C NMR (151 MHz, $CDCl_3$) δ 186.0, 173.1, 170.9, 154.4, 149.8, 144.3, 135.9, 133.0, 131.1, 131.0, 129.7, 129.1, 128.4, 126.3, 122.6, 79.6, 68.8, 60.1, 56.8, 52.9, 51.0, 41.1, 37.4, 31.4, 25.3, 21.7, 19.2, 10.9.

6. References

[1] M.-A. Bray, S. Singh, H. Han, C. T. Davis, B. Borgeson, C. Hartland, M. Kost-Alimova, S. M. Gustafsdottir, C. C. Gibson, A. E. Carpenter, *Nat. Protoc.* **2016**, *11*, 1757-1774.

[2] M. H. Woehrmann, W. M. Bray, J. K. Durbin, S. C. Nisam, A. K. Michael, E. Glassey, J. M. Stuart, R. S. Lokey, *Mol. Biosyst.* **2013**, *9*, 2604-2617.

[3] O. V. Dolomanov, L. J. Bourhis, R. J. Gildea, J. A. K. Howard, H. Puschmann, *J. Appl. Crystallogr.* **2009**, *42*, 339-341.

[4] G. Sheldrick, *Acta Crystallogr. Sect. A* **2015**, *71*, 3-8.

[5] G. Sheldrick, *Acta Crystallogr. Sect. A* **2008**, *64*, 112-122.

[6] H. Chen, G. Wu, S. Gao, R. Guo, Z. Zhao, H. Yuan, S. Liu, J. Wu, X. Lu, X. Yuan, Z. Yu, X. Zu, N. Xie, N. Yang, Z. Hu, Q. Sun, W. Zhang, *J. Med. Chem.* **2017**, *60*, 6828-6852.

[7] Y. Mu, X. Tan, Y. Zhang, X. Jing, Z. Shi, *Org. Chem. Front.* **2016**, *3*, 380-384.

[8] J.-D. Zhai, D. Li, J. Long, H.-L. Zhang, J.-P. Lin, C.-J. Qiu, Q. Zhang, Y. Chen, *J. Org. Chem.* **2012**, *77*, 7103-7107.

[9] G. Liu, S. Song, S. Shu, Z. Miao, A. Zhang, C. Ding, *Eur. J. Med. Chem.* **2015**, *103*, 17-28.

7. NMR spectra

7.1 NMR spectra of santonin-pyrrolidines

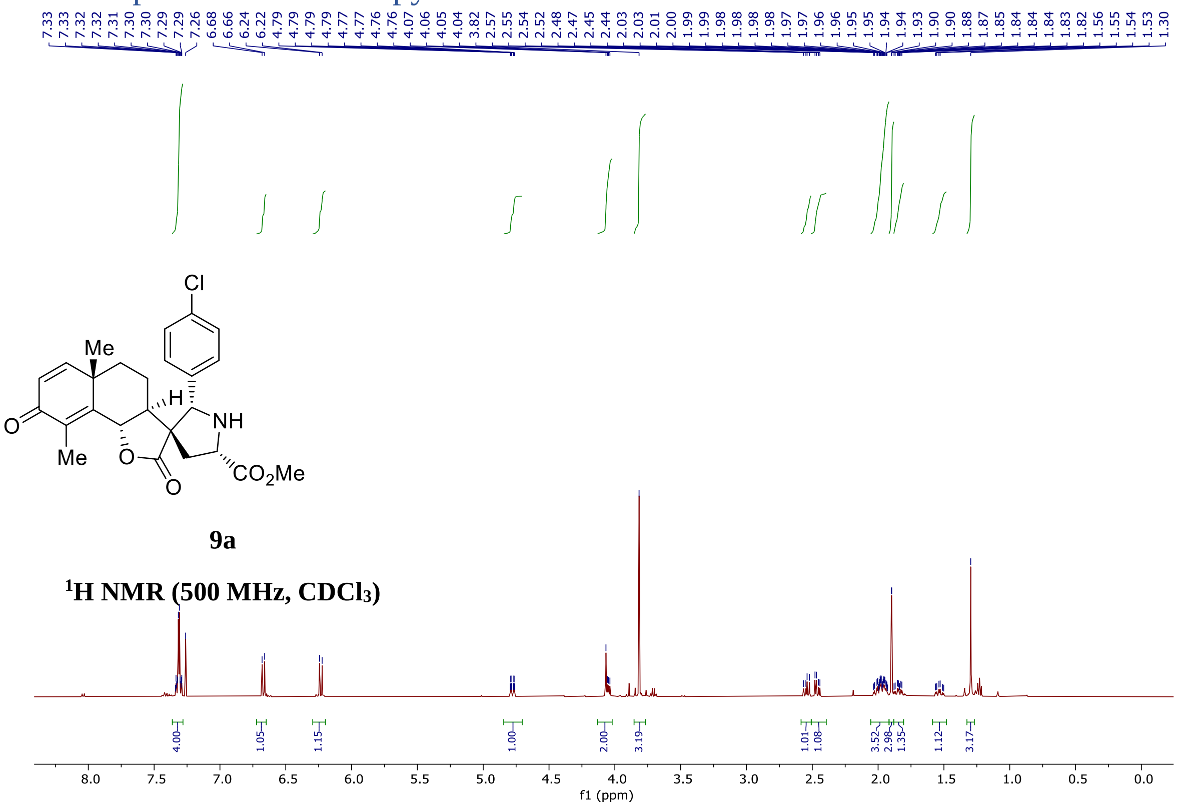

9a

^{1}H NMR (500 MHz, CDCl$_3$)

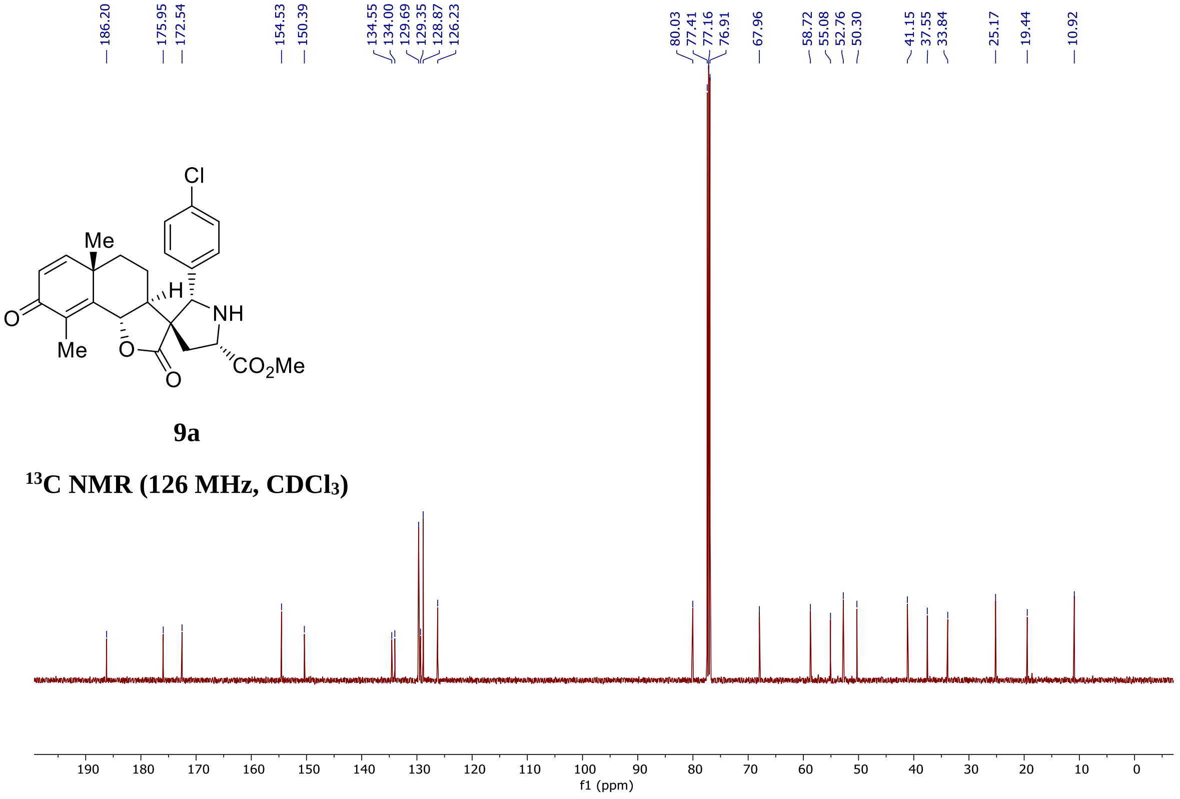

9a

^{13}C NMR (126 MHz, CDCl$_3$)

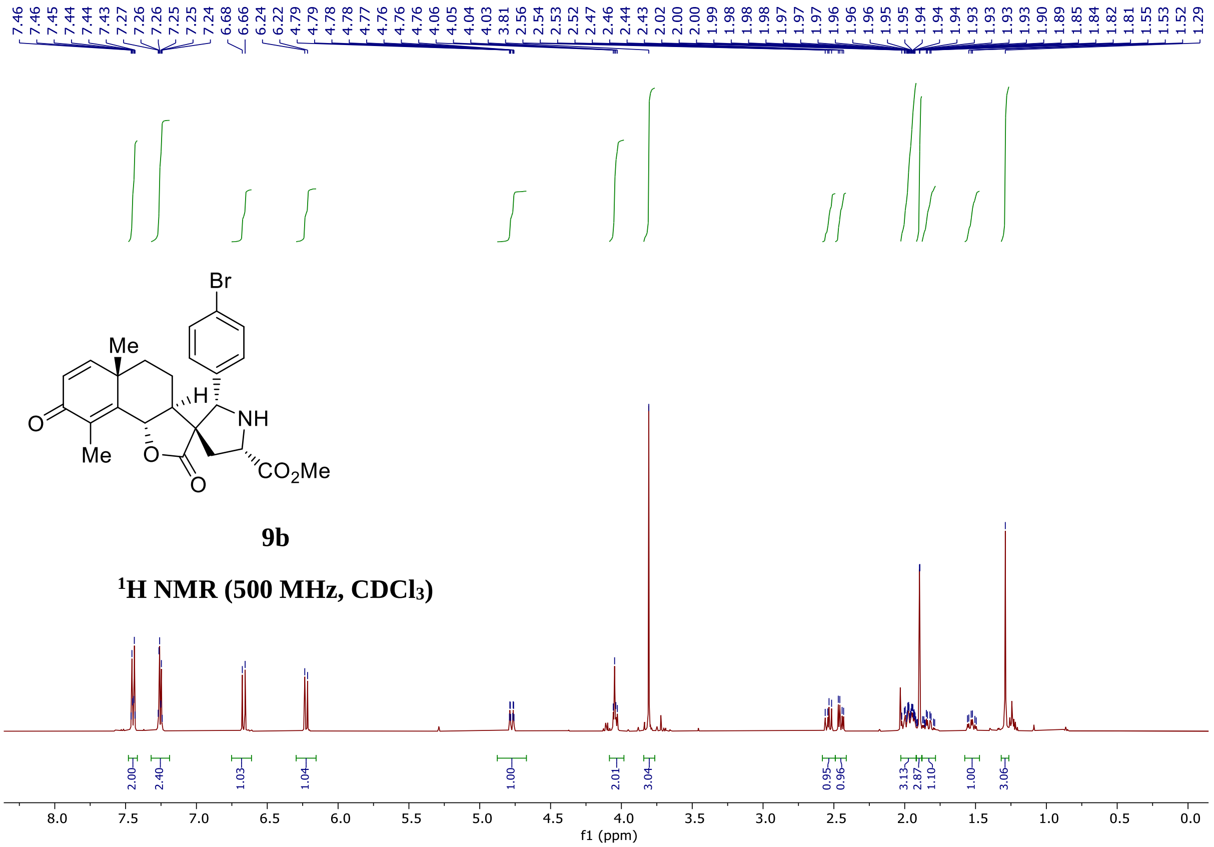
7.46 7.46 7.45 7.44 7.44 7.43 7.27 7.26 7.26 7.25 7.25 7.24 6.68 6.66 6.24 6.22 4.79 4.79 4.78 4.78 4.77 4.76 4.76 4.76 4.06 4.05 4.04 4.03 3.81 2.56 2.54 2.53 2.52 2.47 2.46 2.44 2.43 2.02 2.00 2.00 1.99 1.98 1.98 1.98 1.97 1.97 1.97 1.96 1.96 1.96 1.95 1.95 1.94 1.94 1.94 1.93 1.93 1.93 1.93 1.90 1.89 1.85 1.84 1.82 1.81 1.55 1.53 1.52 1.29
Br
Me
H
NH
O
Me
O
O
CO2Me
9b
1H NMR (500 MHz, CDCl3)
2.00 2.40 1.03 1.04 1.00 2.01 3.04 0.95 0.96 3.13 2.87 1.10 1.00 3.06
8.0 7.5 7.0 6.5 6.0 5.5 5.0 4.5 4.0 3.5 3.0 2.5 2.0 1.5 1.0 0.5 0.0
f1 (ppm)

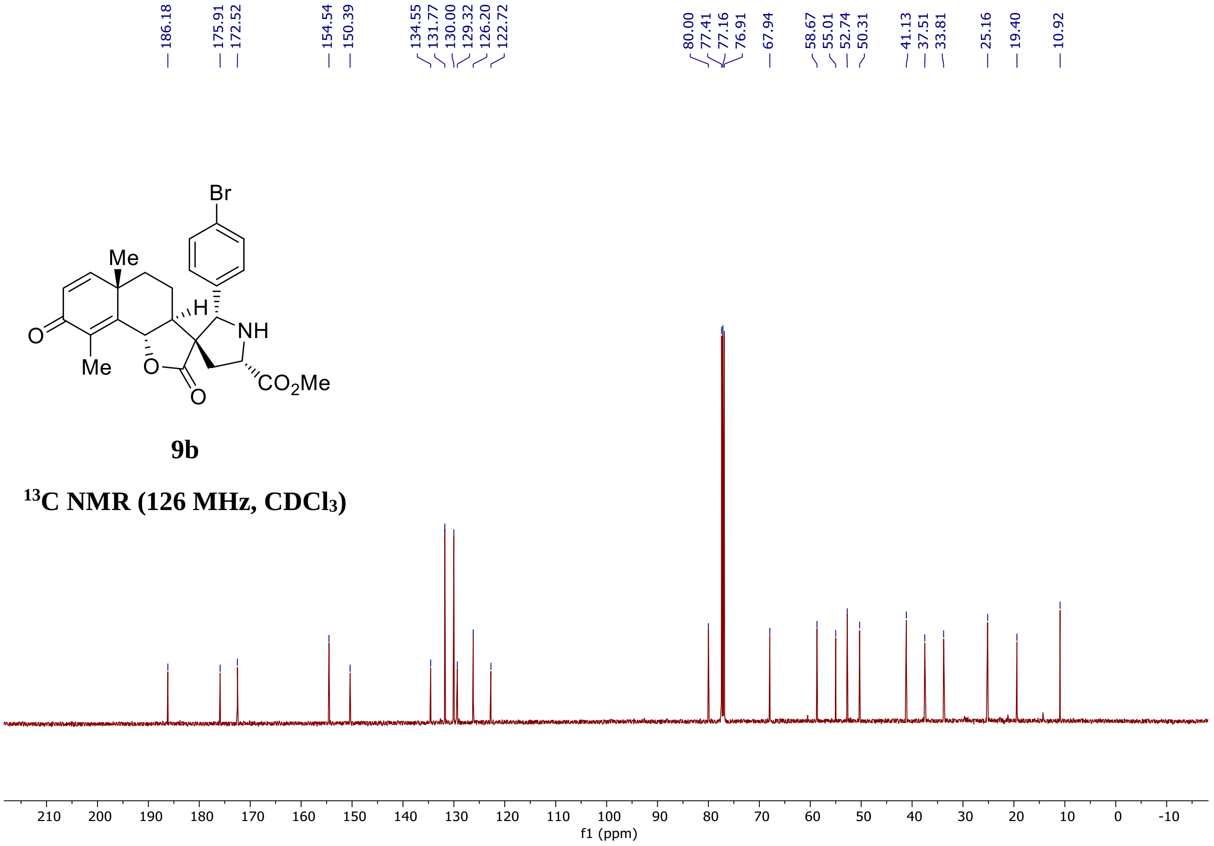
186.18 175.91 172.52 154.54 150.39 134.55 131.77 130.00 129.32 126.20 122.72 80.00 77.41 77.16 76.91 67.94 58.67 55.01 52.74 50.31 41.13 37.51 33.81 25.16 19.40 10.92
Br
Me
H
NH
O
Me
O
O
CO2Me
9b
13C NMR (126 MHz, CDCl3)
210 200 190 180 170 160 150 140 130 120 110 100 90 80 70 60 50 40 30 20 10 0 -10
f1 (ppm)

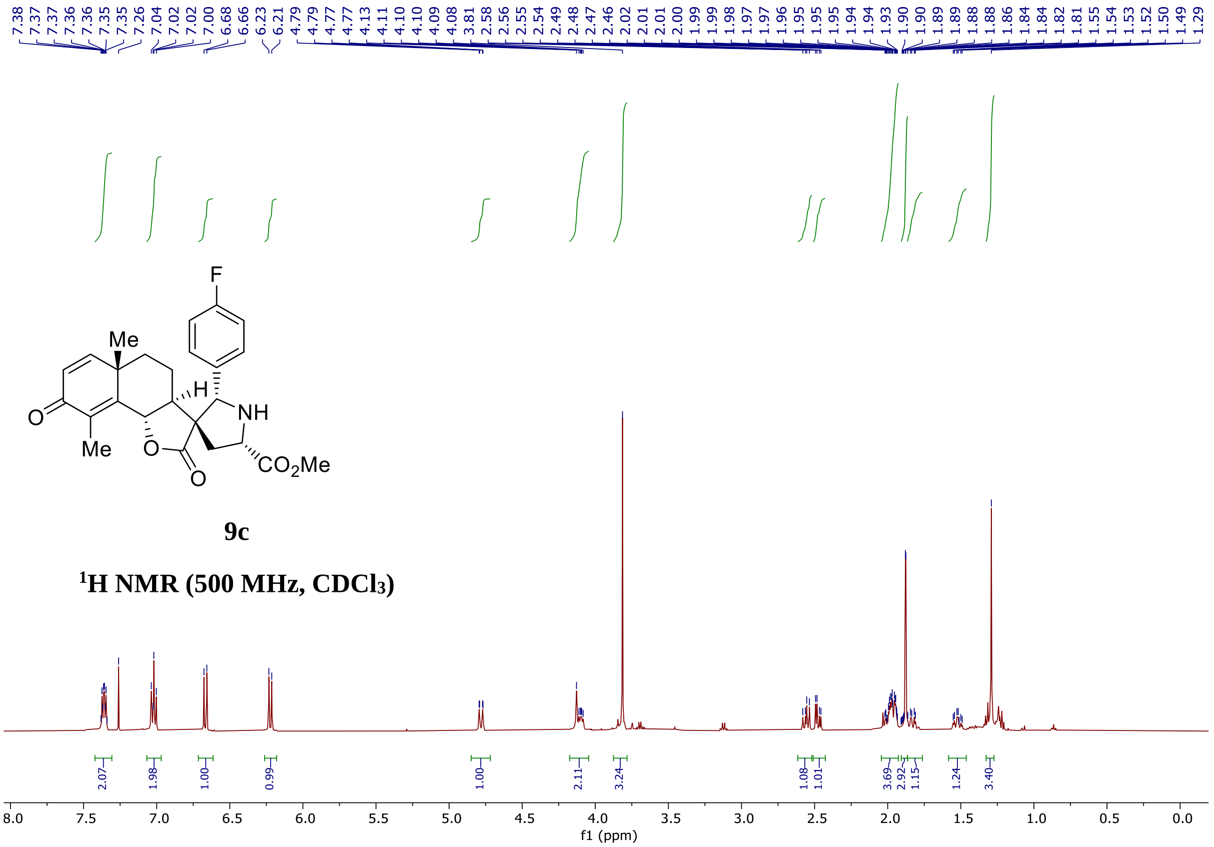

9c

^{1}H NMR (500 MHz, CDCl$_3$)

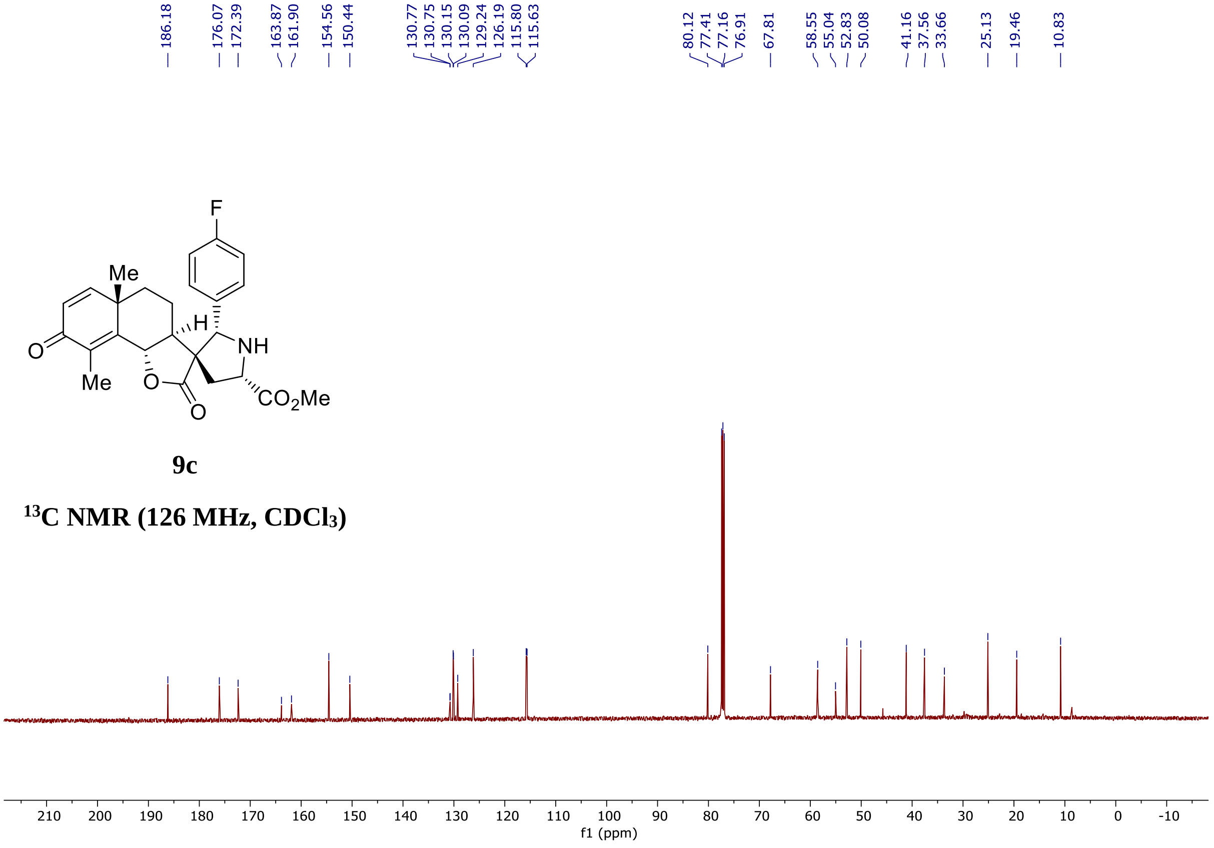

9c

^{13}C NMR (126 MHz, CDCl$_3$)

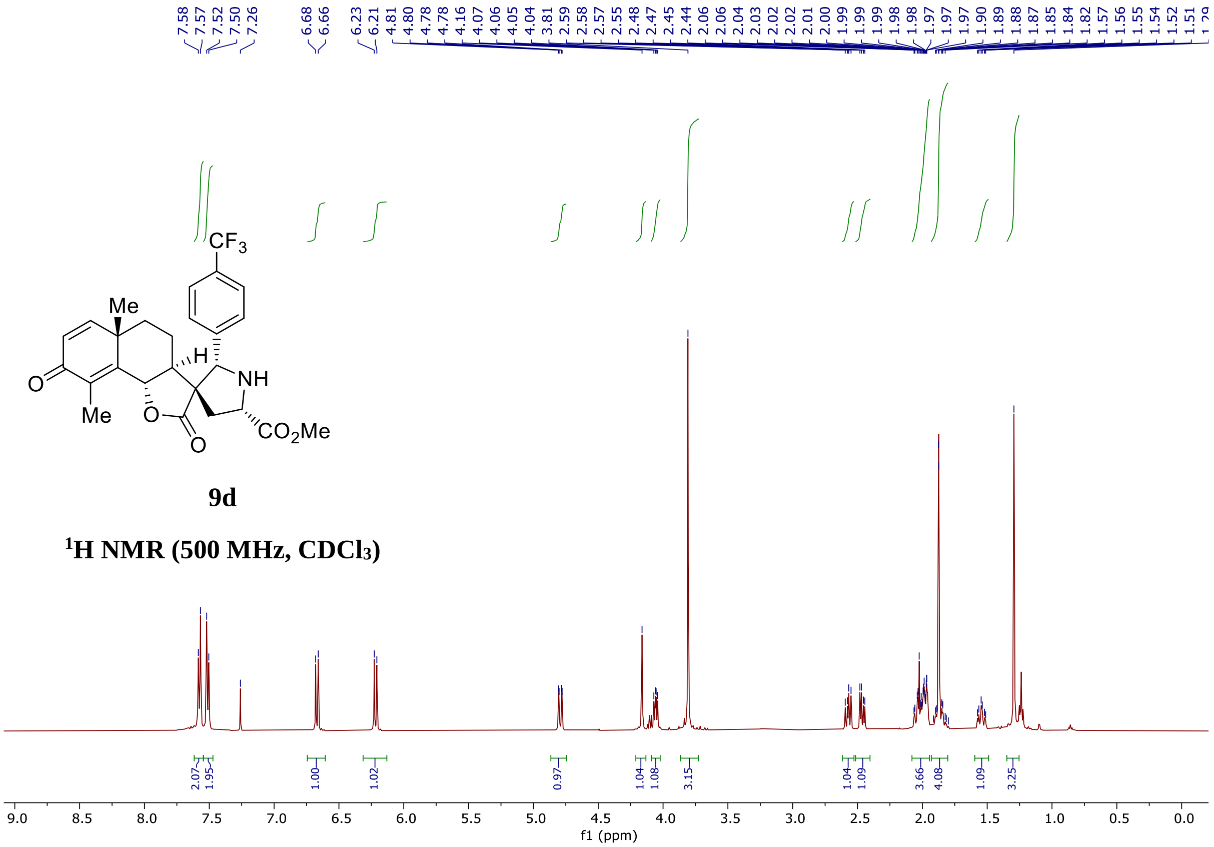
7.58
7.57
7.52
7.50
7.26
6.68
6.66
6.23
6.21
4.81
4.80
4.78
4.78
4.16
4.07
4.06
4.05
4.04
3.81
2.59
2.58
2.57
2.55
2.48
2.47
2.45
2.44
2.06
2.06
2.04
2.03
2.02
2.02
2.01
2.00
1.99
1.99
1.99
1.98
1.98
1.97
1.97
1.97
1.90
1.89
1.88
1.87
1.85
1.84
1.82
1.57
1.56
1.55
1.54
1.52
1.51
CF3
Me
H
NH
O
Me
O
O
CO2Me
9d
1H NMR (500 MHz, CDCl3)
2.07
1.95
1.00
1.02
0.97
1.04
1.08
3.15
1.04
1.09
3.66
4.08
1.09
3.25
f1 (ppm)

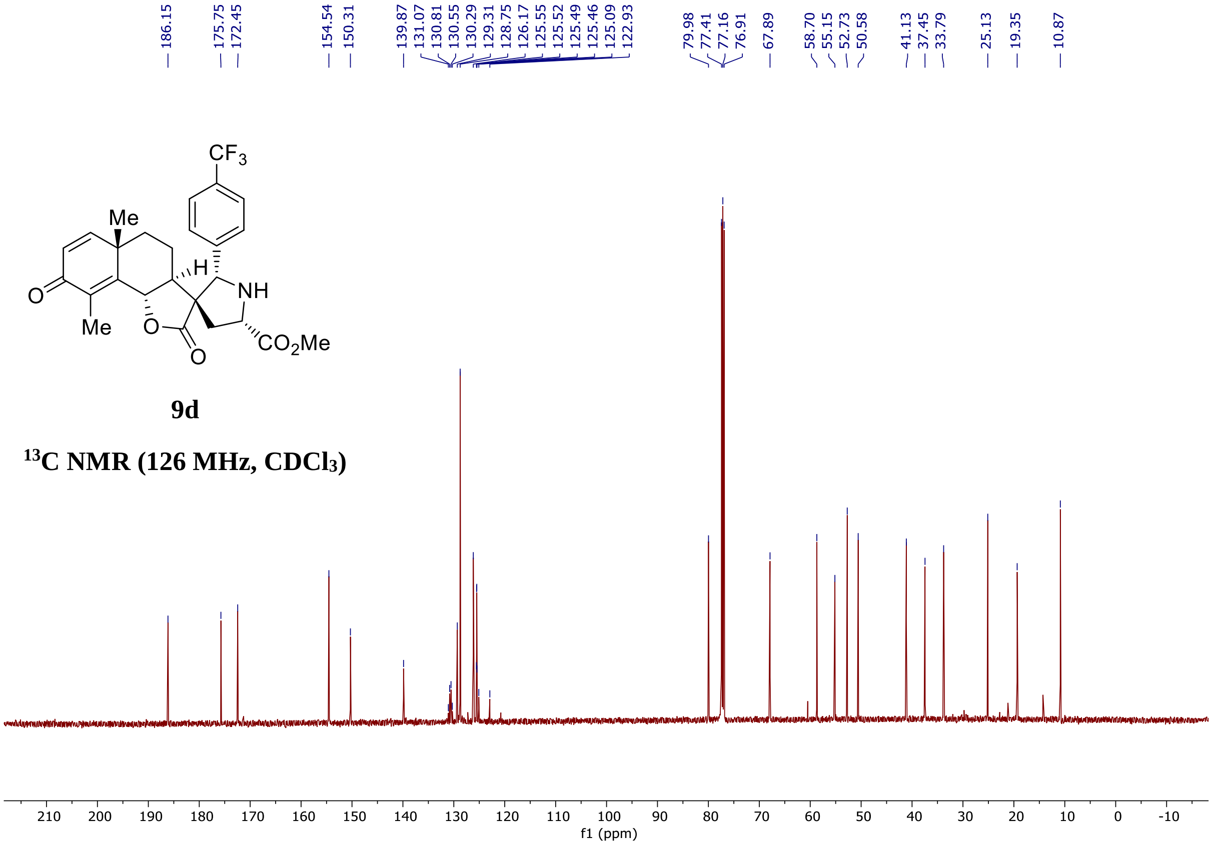
186.15
175.75
172.45
154.54
150.31
139.87
131.07
130.81
130.55
130.29
129.31
128.75
126.17
125.55
125.52
125.49
125.46
125.09
122.93
79.98
77.41
77.16
76.91
67.89
58.70
55.15
52.73
50.58
41.13
37.45
33.79
25.13
19.35
10.87
CF3
Me
H
NH
O
Me
O
O
CO2Me
9d
13C NMR (126 MHz, CDCl3)
f1 (ppm)

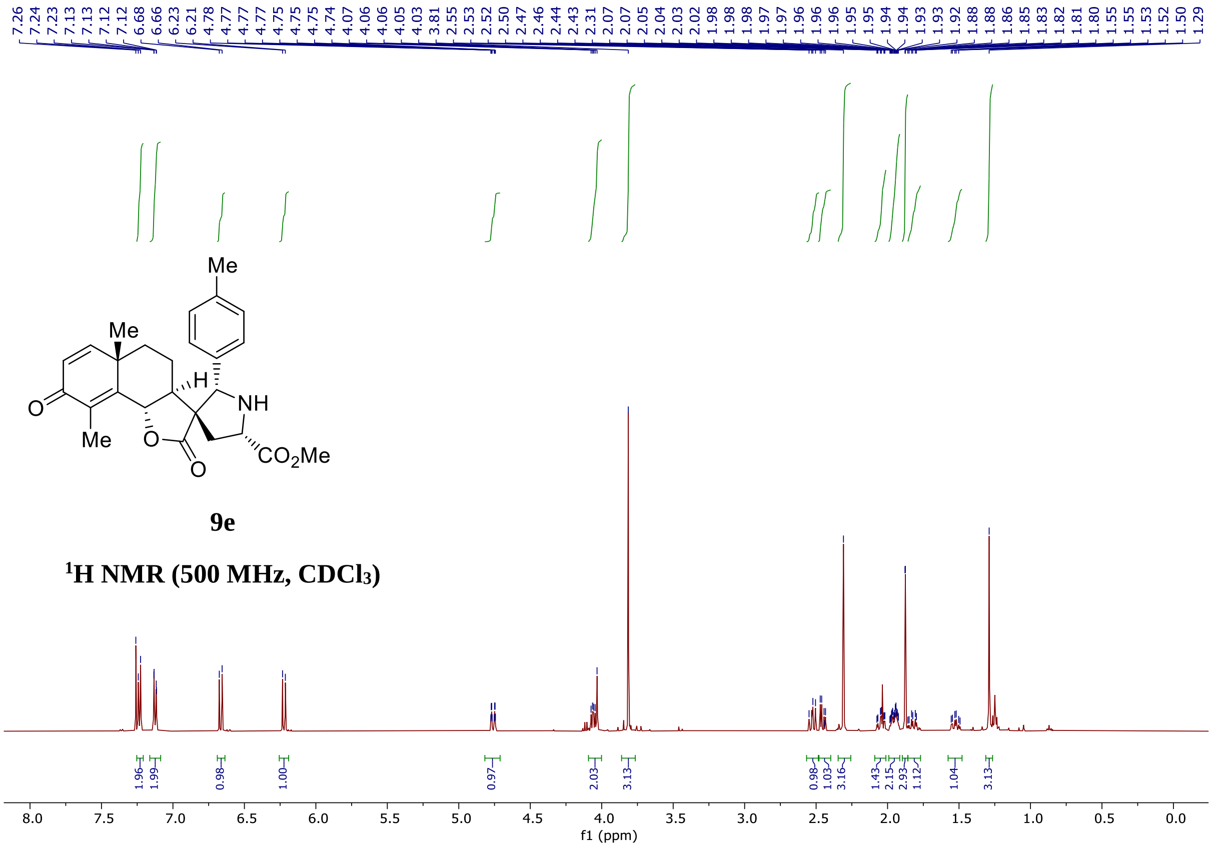

9e

^{1}H NMR (500 MHz, CDCl$_3$)

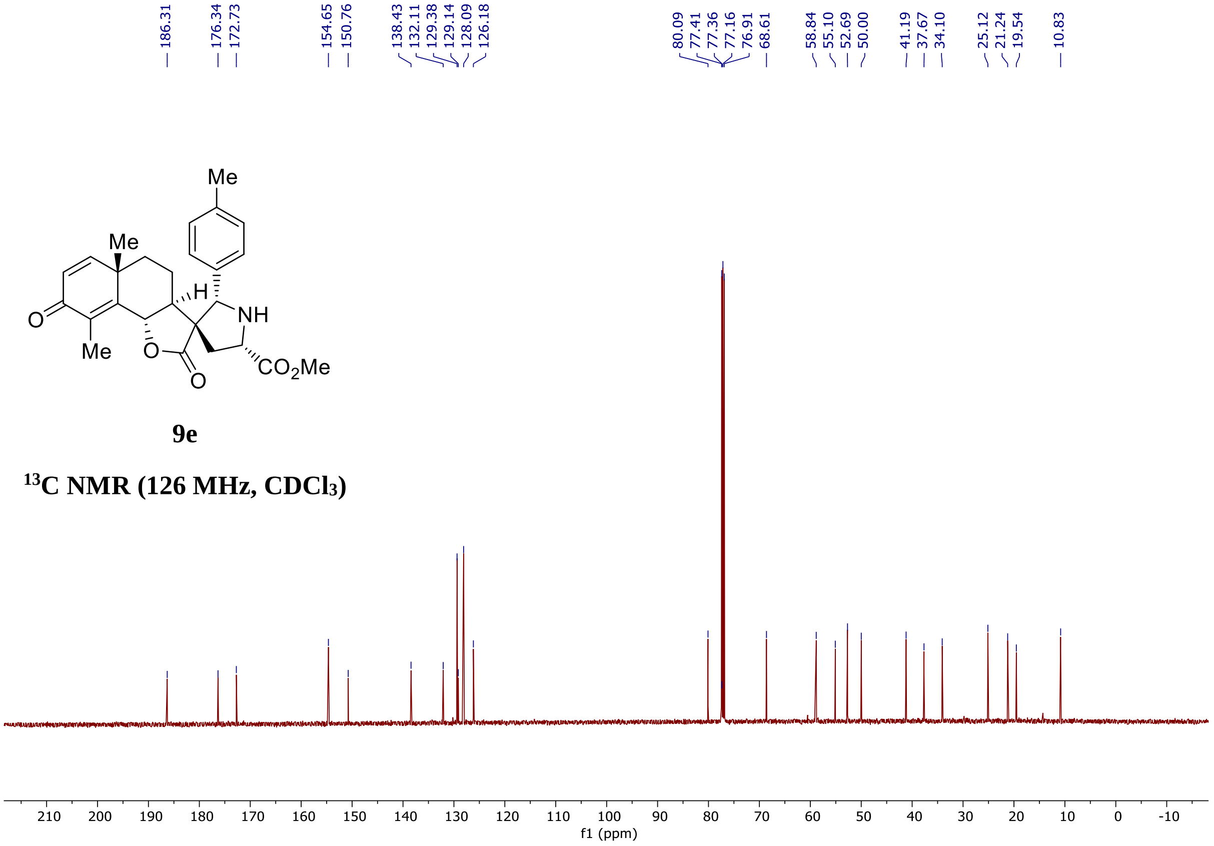

9e

^{13}C NMR (126 MHz, CDCl$_3$)

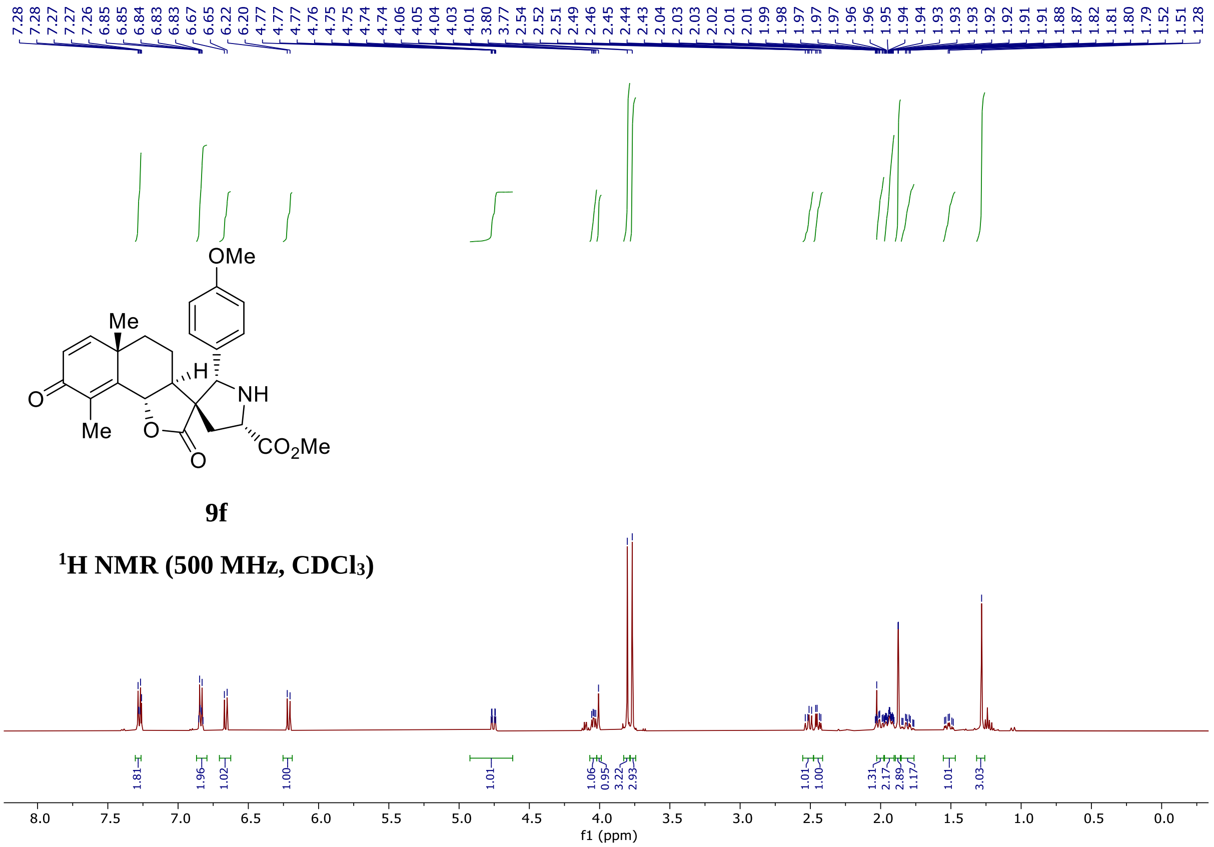

9f

^{1}H NMR (500 MHz, CDCl$_3$)

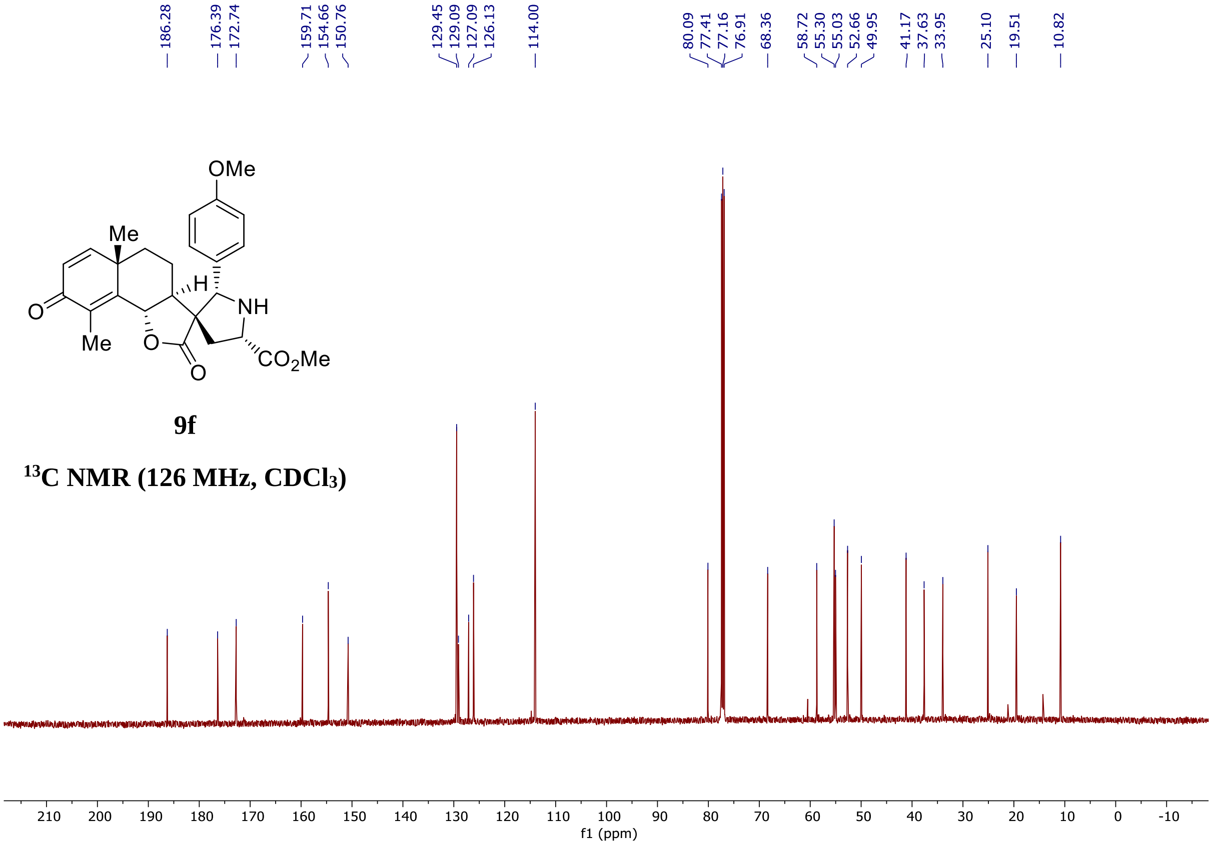

9f

^{13}C NMR (126 MHz, CDCl$_3$)

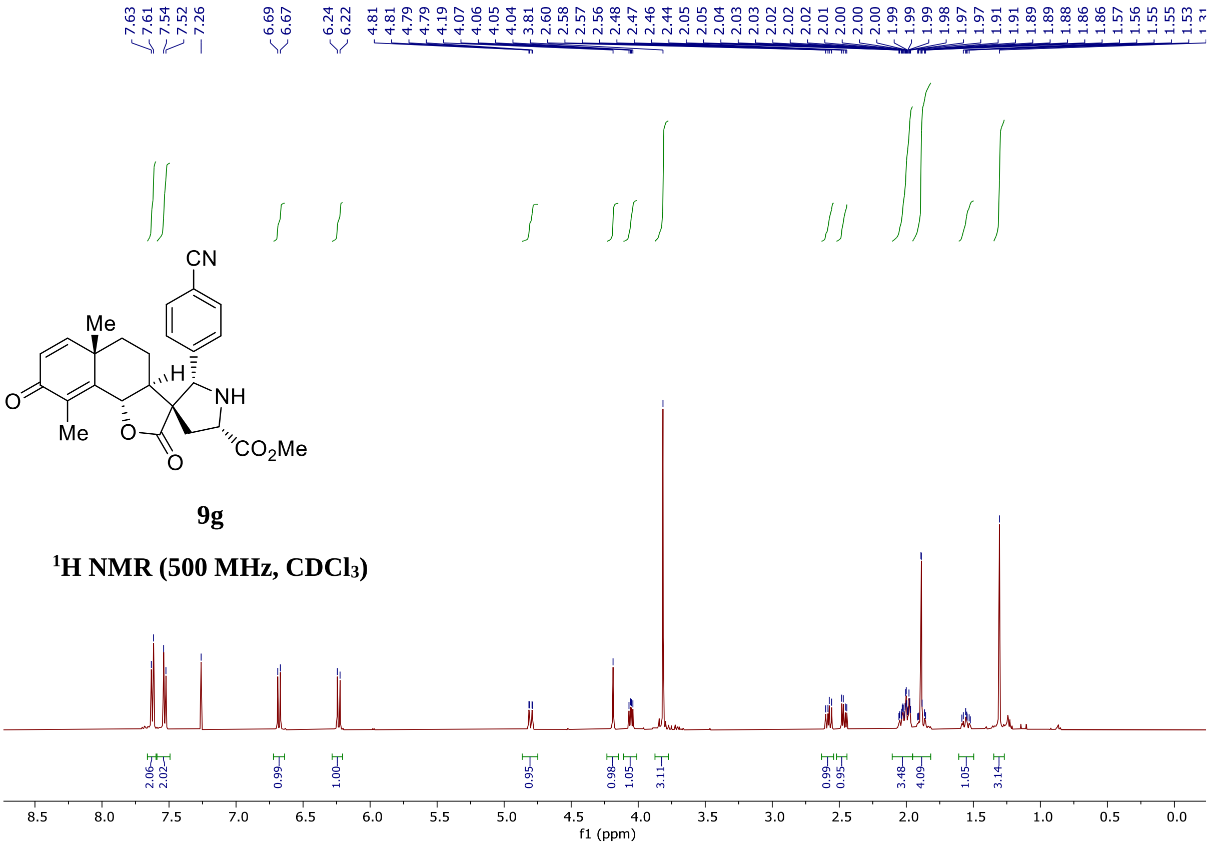

9g

^{1}H NMR (500 MHz, CDCl3)

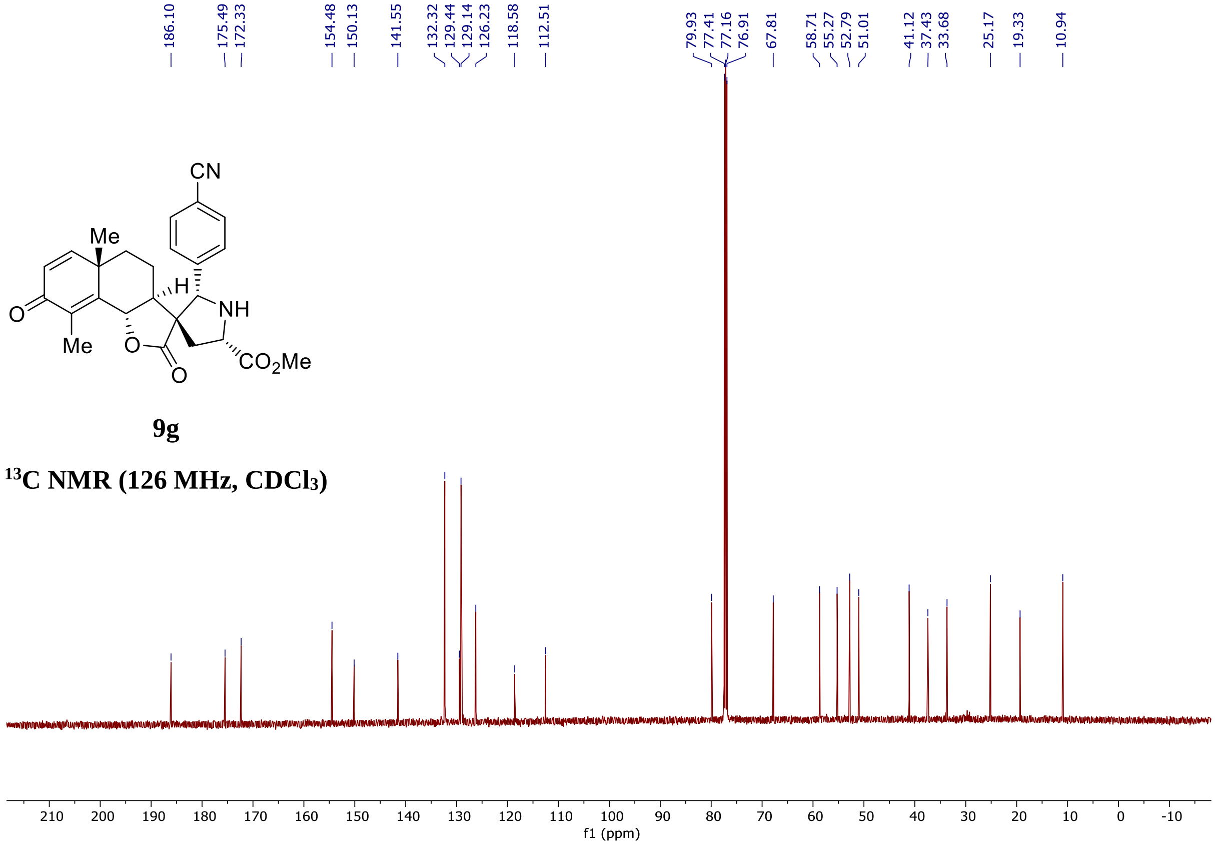

9g

^{13}C NMR (126 MHz, CDCl3)

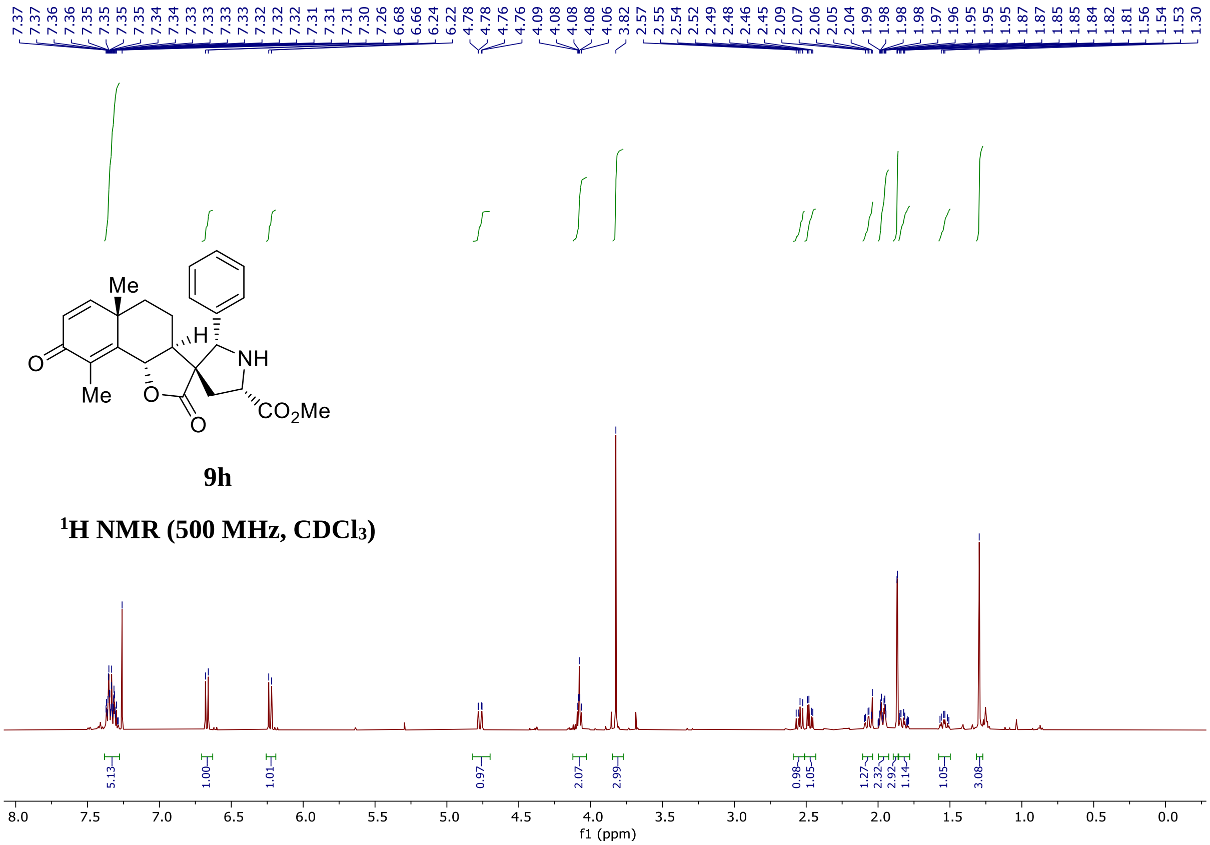

9h

^{1}H NMR (500 MHz, $CDCl_3$)

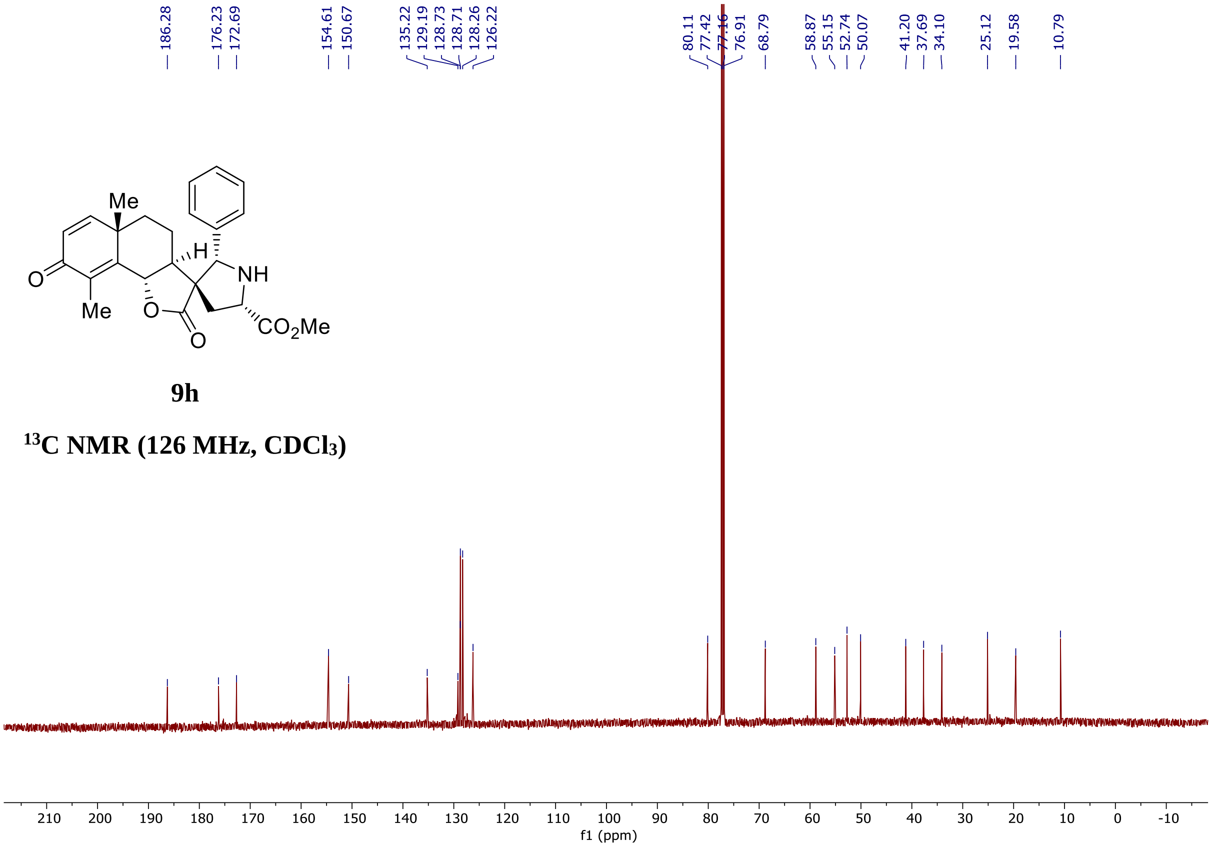

9h

^{13}C NMR (126 MHz, $CDCl_3$)

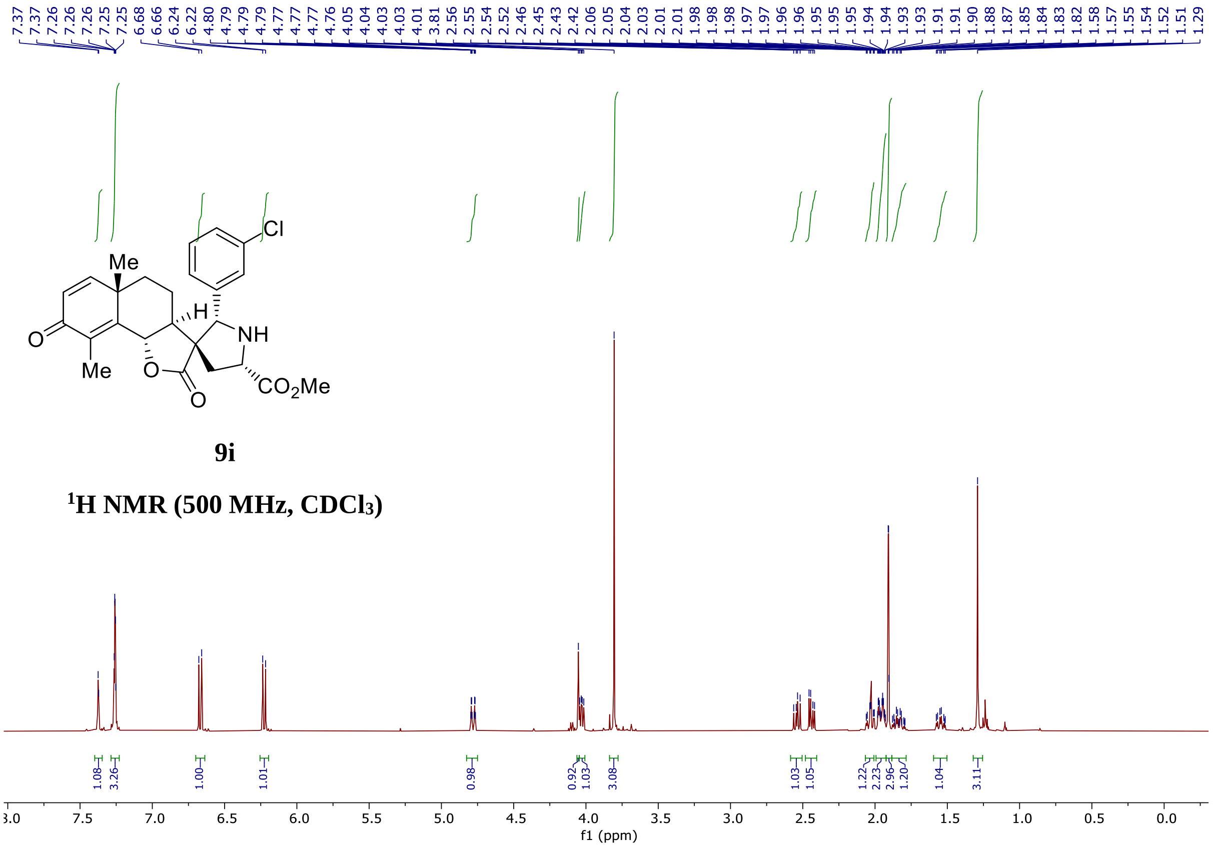

9i

^{1}H NMR (500 MHz, $CDCl_3$)

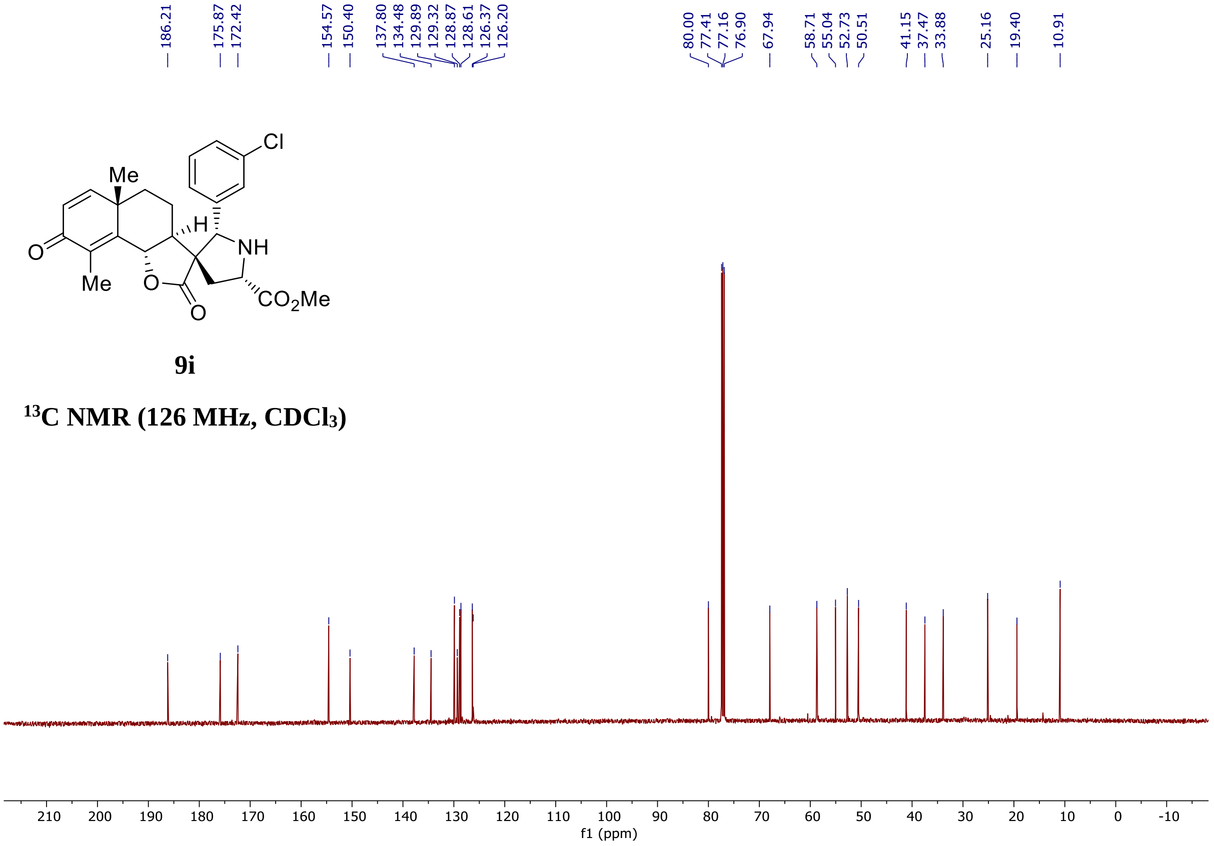

9i

^{13}C NMR (126 MHz, $CDCl_3$)

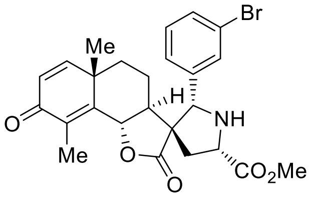

9j

^{1}H NMR (500 MHz, CDCl3)

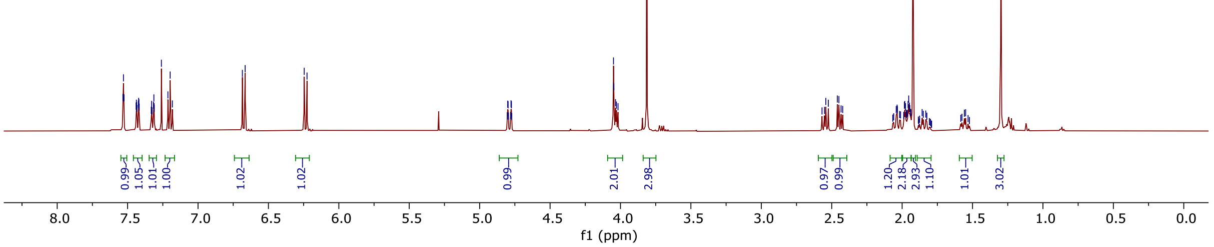

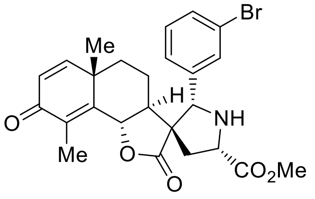

9j

^{13}C NMR (126 MHz, CDCl3)

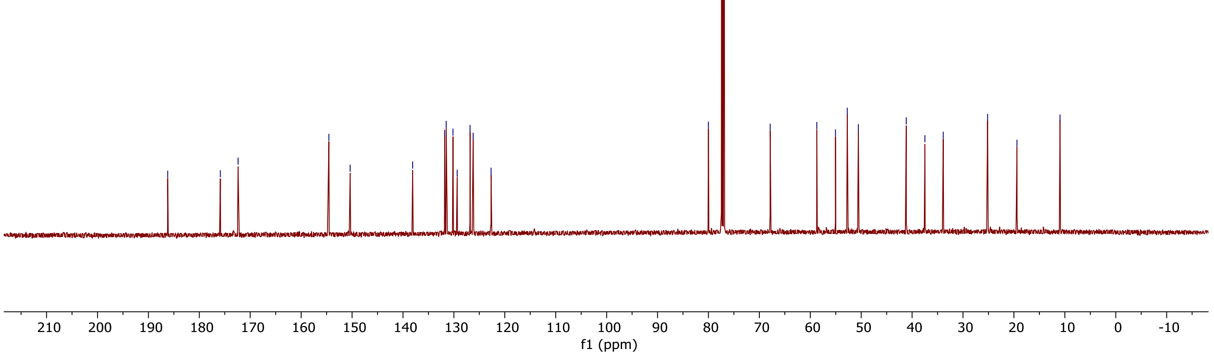

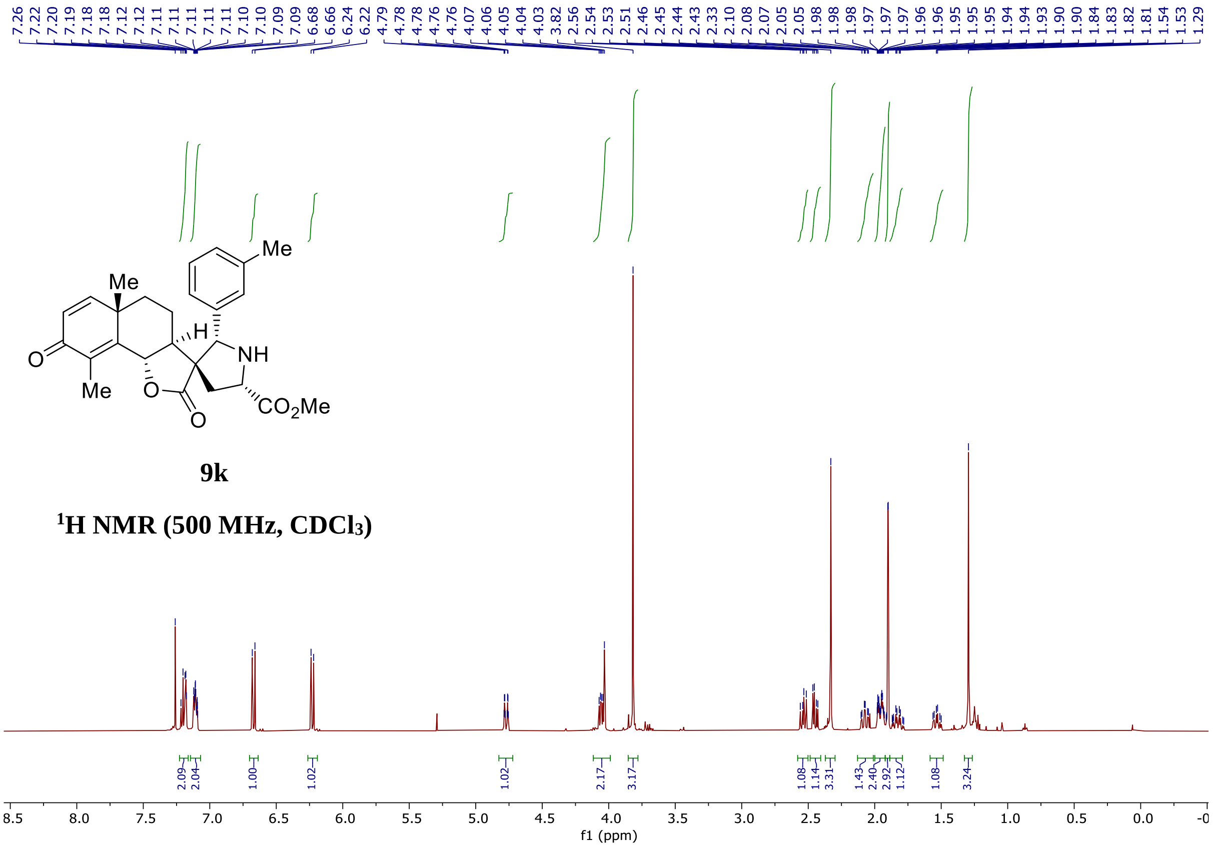
7.26 7.22 7.20 7.19 7.18 7.18 7.12 7.12 7.11 7.11 7.11 7.11 7.10 7.09 7.09 6.68 6.66 6.24 6.22 4.79 4.78 4.78 4.76 4.07 4.06 4.05 4.04 4.03 3.82 2.56 2.54 2.53 2.51 2.46 2.45 2.44 2.43 2.33 2.10 2.08 2.07 2.05 2.05 1.98 1.98 1.98 1.97 1.97 1.97 1.96 1.96 1.95 1.95 1.95 1.94 1.94 1.93 1.90 1.90 1.84 1.83 1.82 1.81 1.54 1.53 1.29
Me
Me
H
NH
O
Me
O
O
CO2Me
9k
1H NMR (500 MHz, CDCl3)
2.09 2.04 1.00 1.02 1.02 2.17 3.17 1.08 1.14 3.31 1.43 2.40 2.92 1.12 1.08 3.24
8.5 8.0 7.5 7.0 6.5 6.0 5.5 5.0 4.5 4.0 3.5 3.0 2.5 2.0 1.5 1.0 0.5 0.0
f1 (ppm)

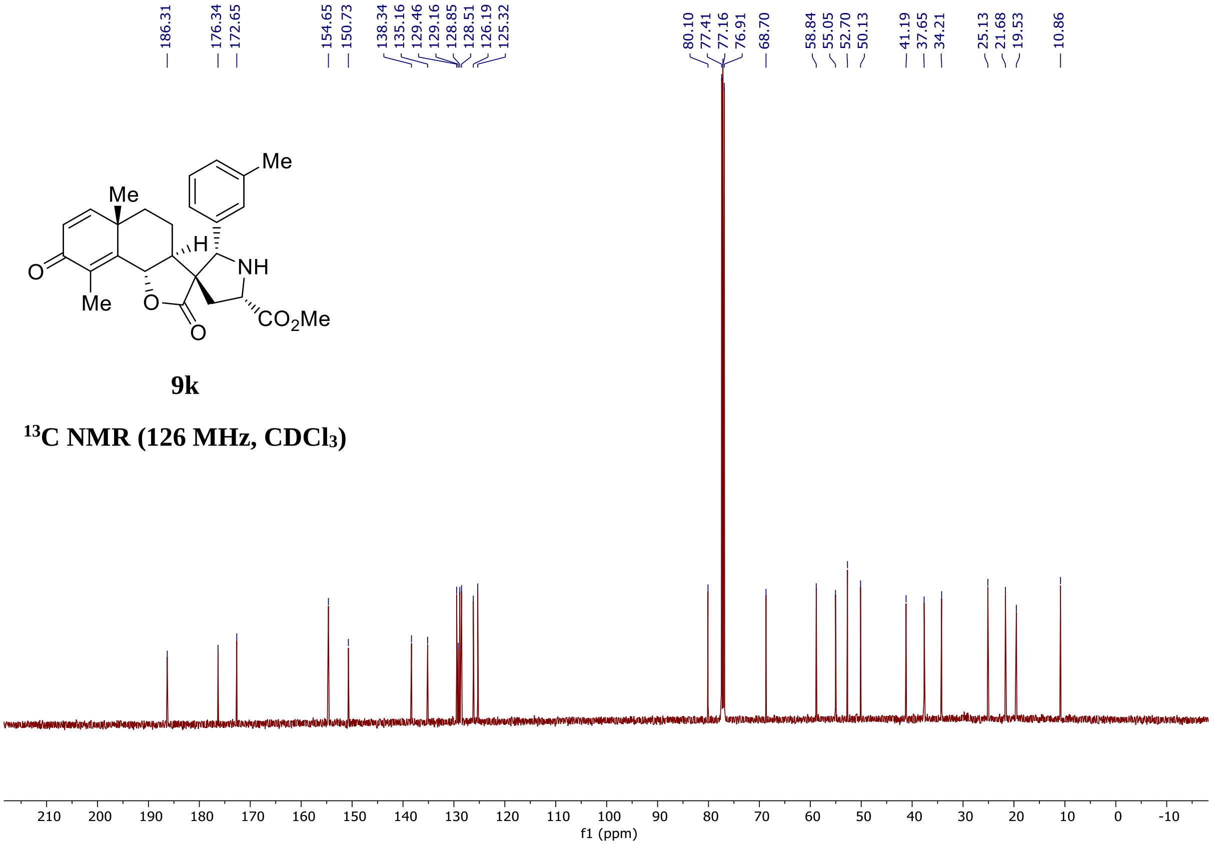
186.31 176.34 172.65 154.65 150.73 138.34 135.16 129.46 129.16 128.85 128.51 126.19 125.32 80.10 77.41 77.16 76.91 68.70 58.84 55.05 52.70 50.13 41.19 37.65 34.21 25.13 21.68 19.53 10.86
Me
Me
H
NH
O
Me
O
O
CO2Me
9k
13C NMR (126 MHz, CDCl3)
210 200 190 180 170 160 150 140 130 120 110 100 90 80 70 60 50 40 30 20 10 0 -10
f1 (ppm)

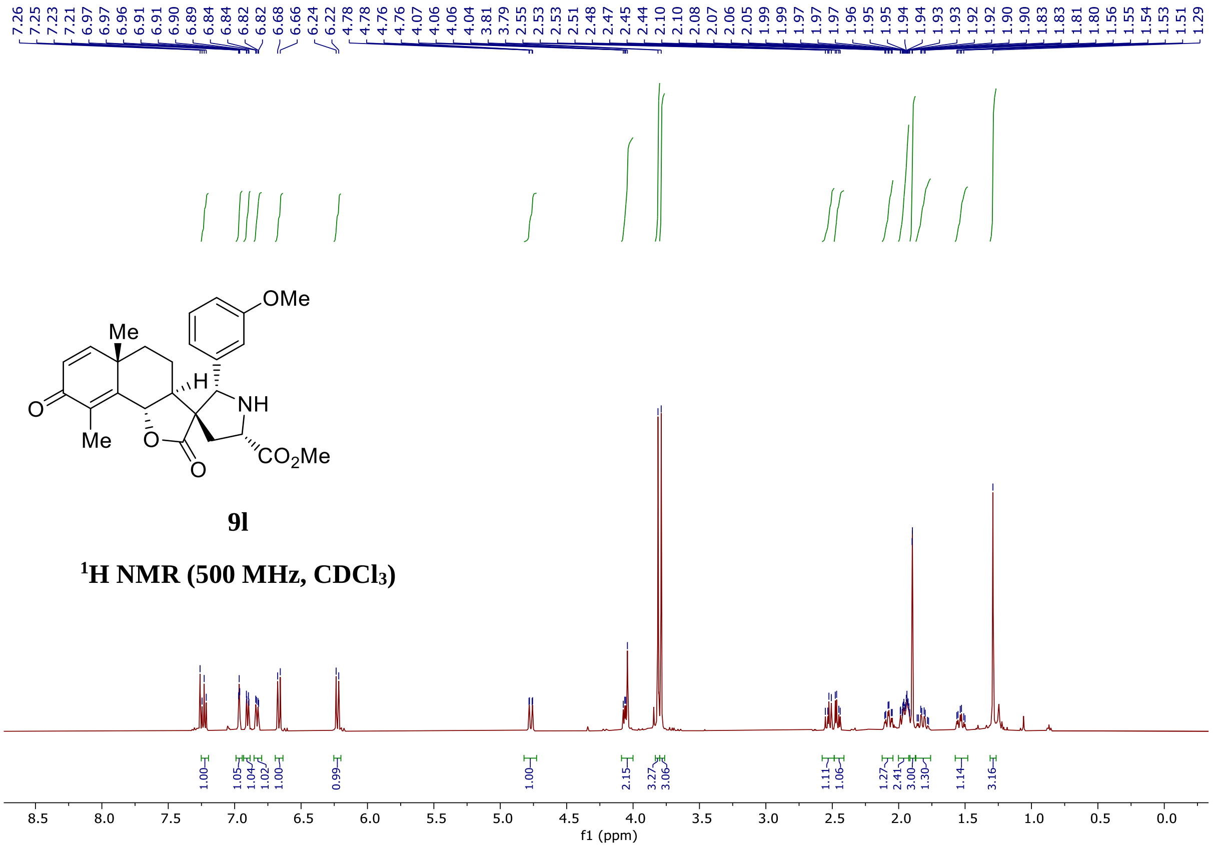
7.26 7.25 7.23 7.21 6.97 6.97 6.96 6.91 6.91 6.90 6.89 6.84 6.84 6.82 6.82 6.68 6.66 6.24 6.22 4.78 4.76 4.07 4.06 4.06 4.04 3.81 3.79 2.55 2.53 2.53 2.51 2.48 2.47 2.45 2.44 2.10 2.10 2.08 2.07 2.06 2.05 1.99 1.99 1.97 1.97 1.97 1.96 1.95 1.95 1.94 1.94 1.93 1.93 1.92 1.92 1.90 1.90 1.83 1.83 1.81 1.80 1.56 1.55 1.54 1.53 1.51 1.29
OMe
Me
H
NH
O
Me
O
O
CO2Me
9l
1H NMR (500 MHz, CDCl3)
1.00 1.05 1.04 1.02 1.00 0.99 1.00 2.15 3.27 3.06 1.11 1.06 1.27 2.41 3.00 1.30 1.14 3.16
8.5 8.0 7.5 7.0 6.5 6.0 5.5 5.0 4.5 4.0 3.5 3.0 2.5 2.0 1.5 1.0 0.5 0.0
f1 (ppm)

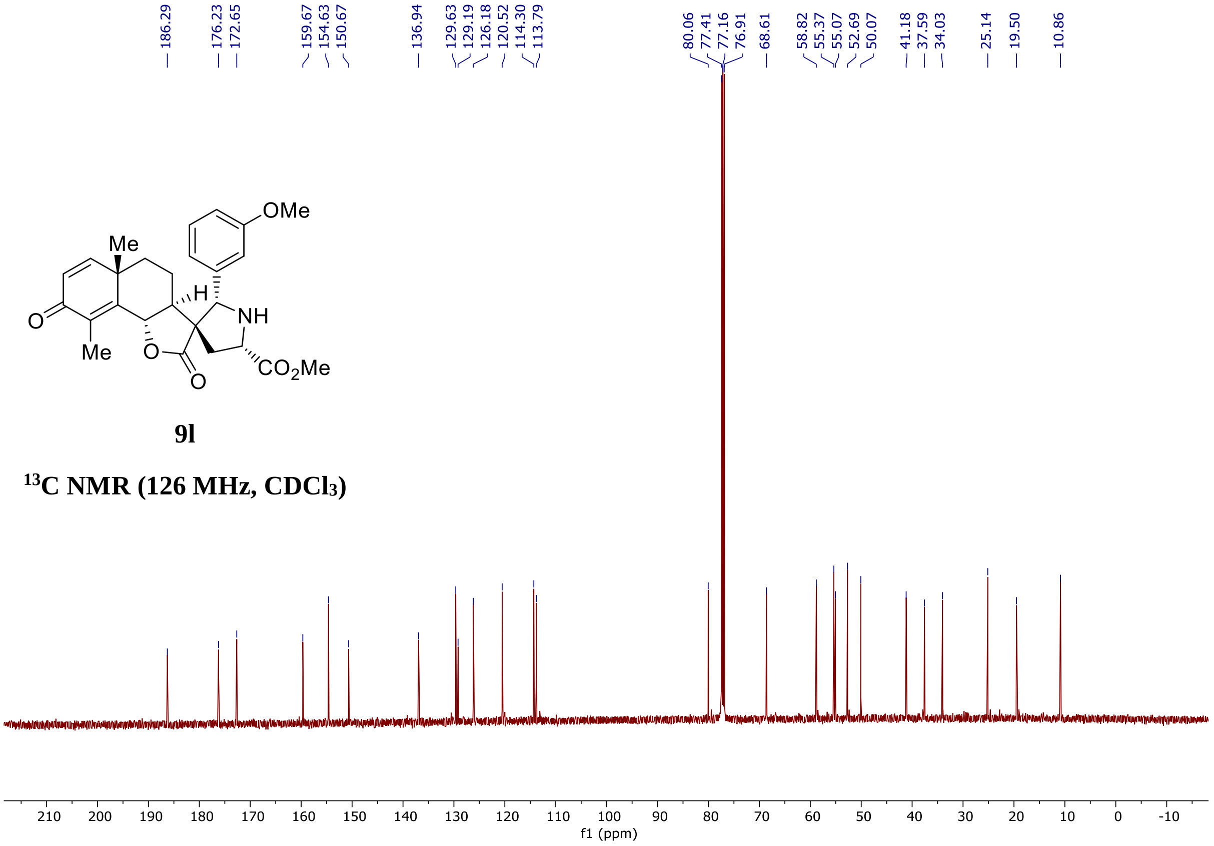
186.29 176.23 172.65 159.67 154.63 150.67 136.94 129.63 129.19 126.18 120.52 114.30 113.79 80.06 77.41 77.16 76.91 68.61 58.82 55.37 55.07 52.69 50.07 41.18 37.59 34.03 25.14 19.50 10.86
OMe
Me
H
NH
O
Me
O
O
CO2Me
9l
13C NMR (126 MHz, CDCl3)
210 200 190 180 170 160 150 140 130 120 110 100 90 80 70 60 50 40 30 20 10 0 -10
f1 (ppm)

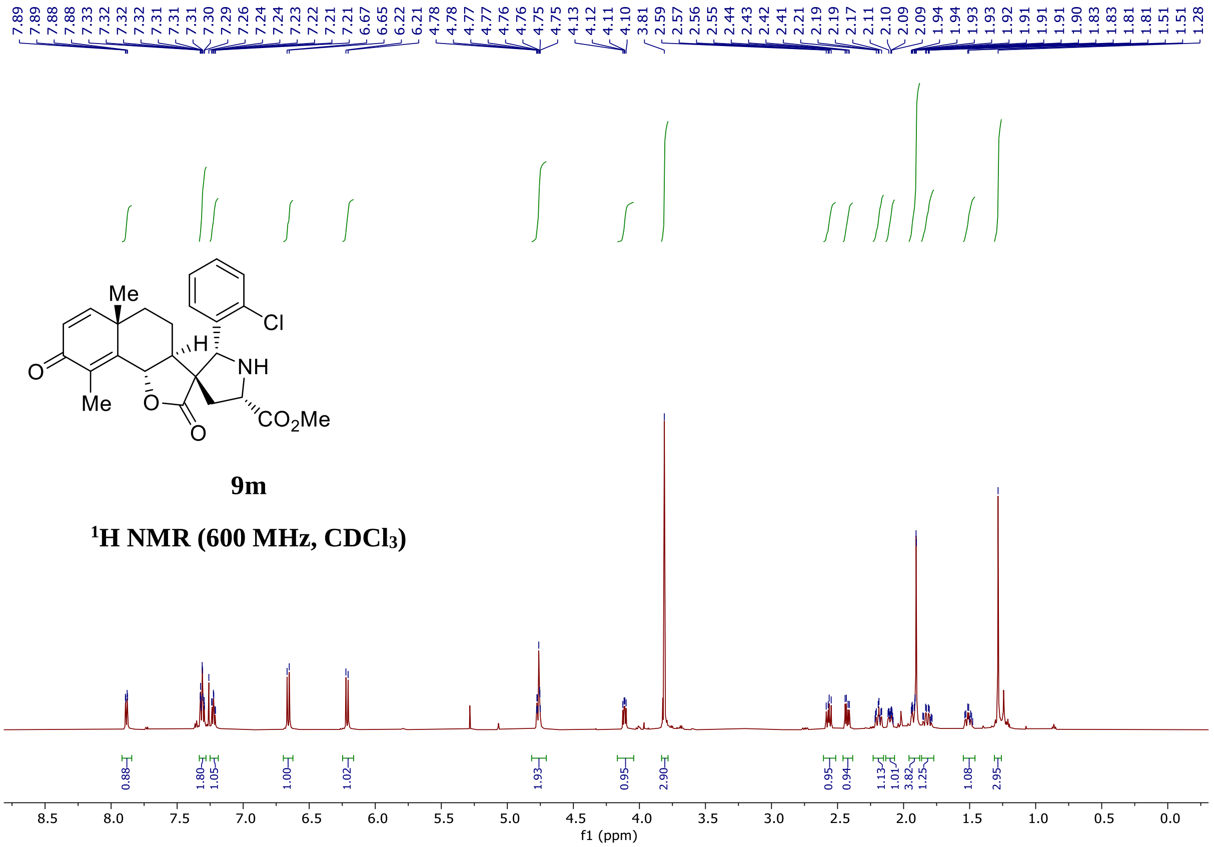

9m

^{1}H NMR (600 MHz, $CDCl_3$)

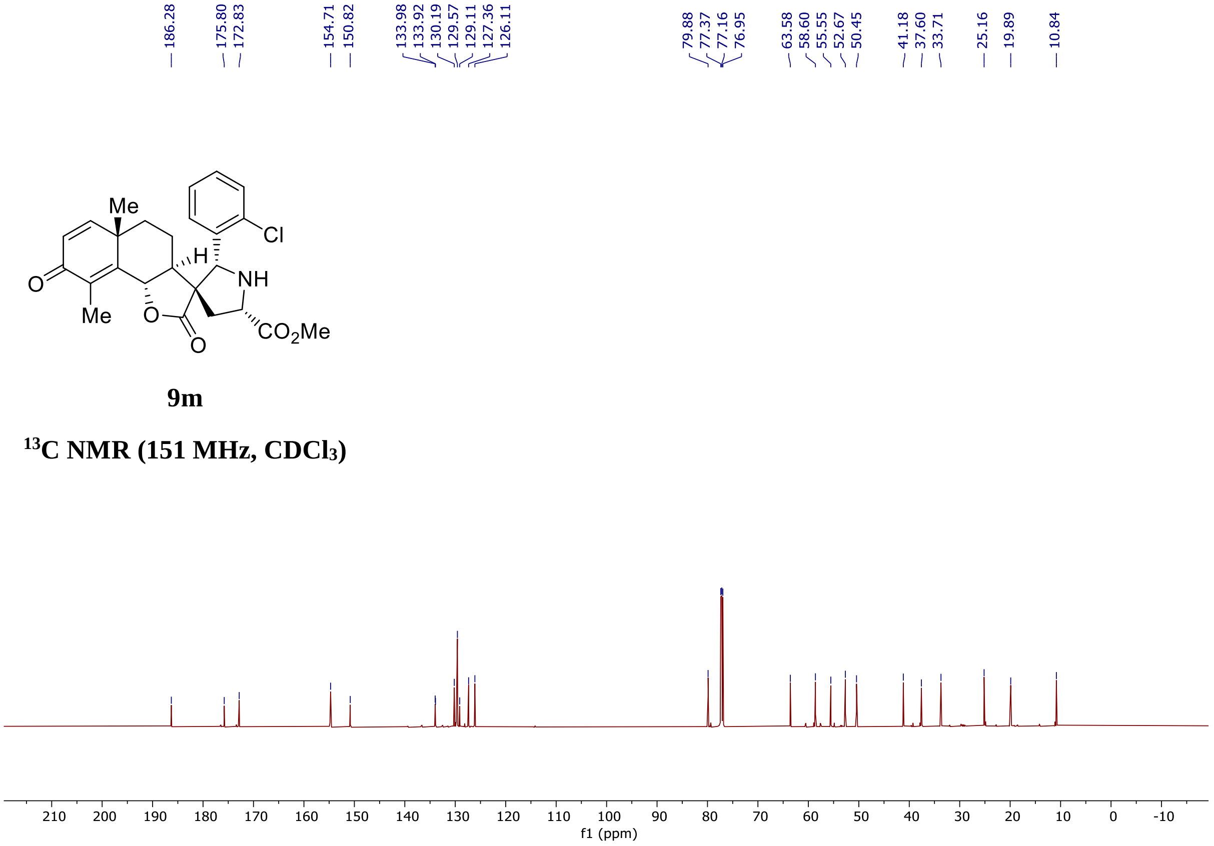

9m

^{13}C NMR (151 MHz, $CDCl_3$)

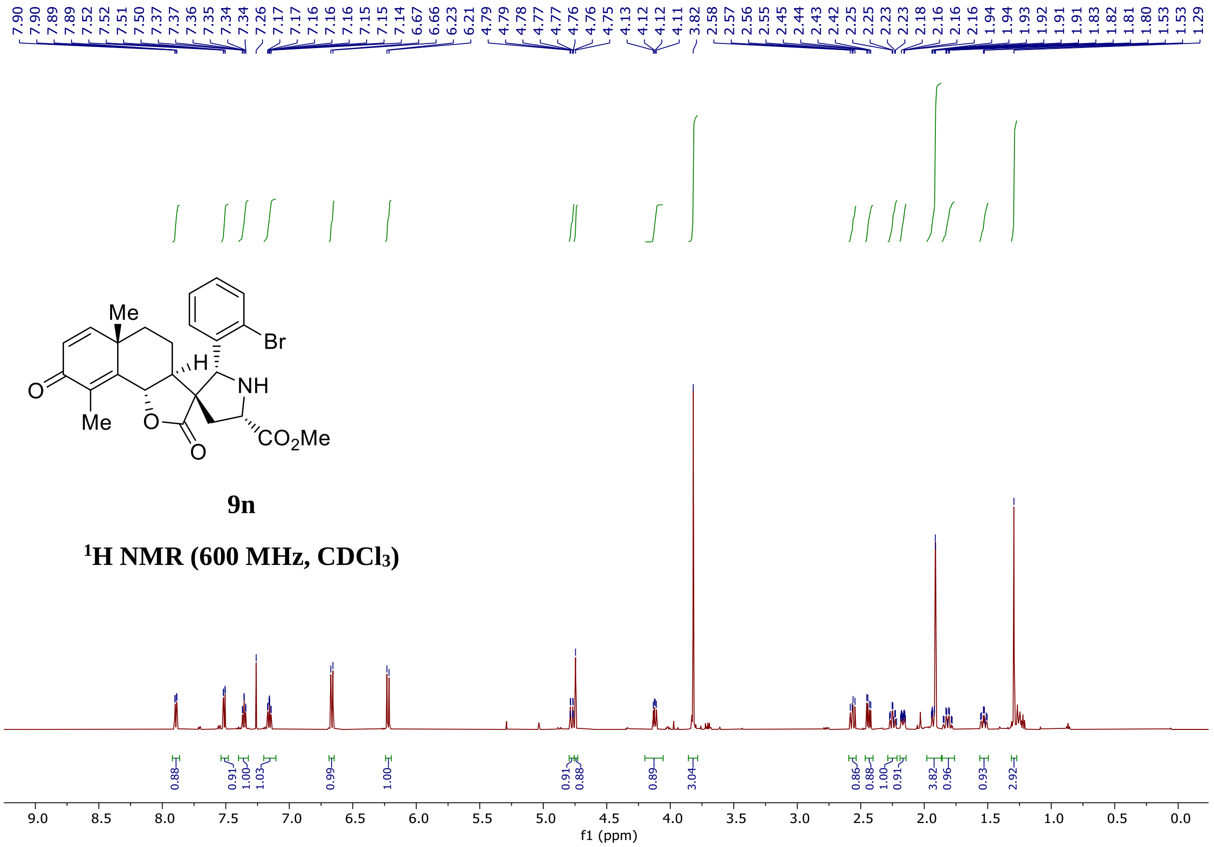

9n

^{1}H NMR (600 MHz, CDCl3)

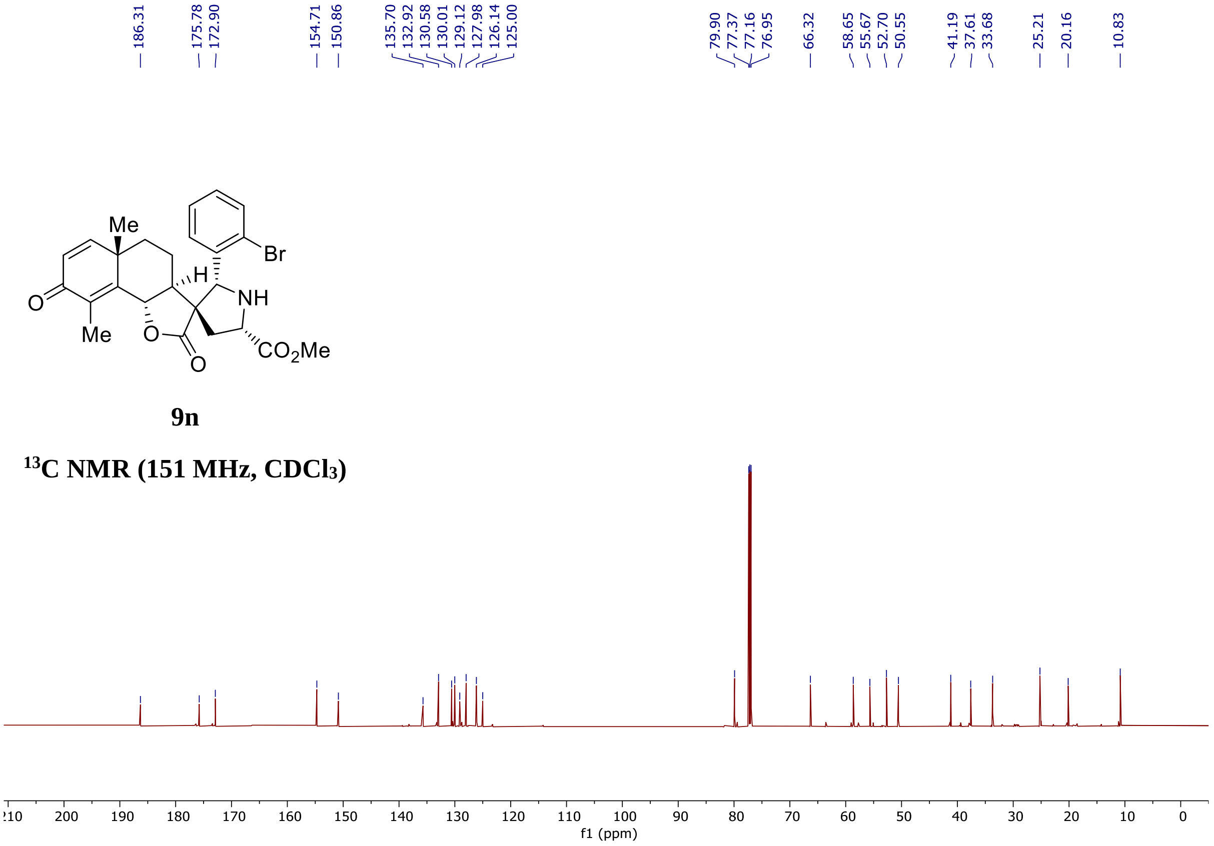

9n

^{13}C NMR (151 MHz, CDCl3)

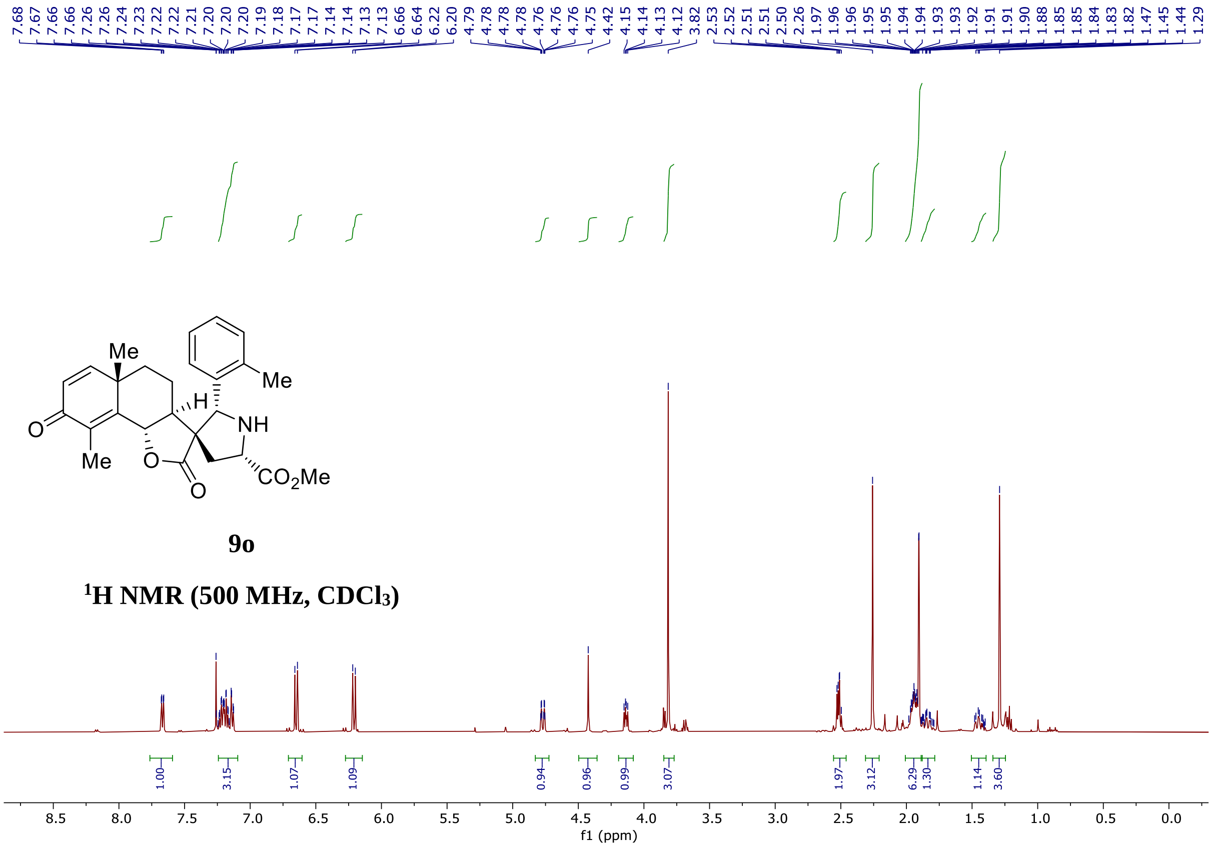

9o

^{1}H NMR (500 MHz, $CDCl_3$)

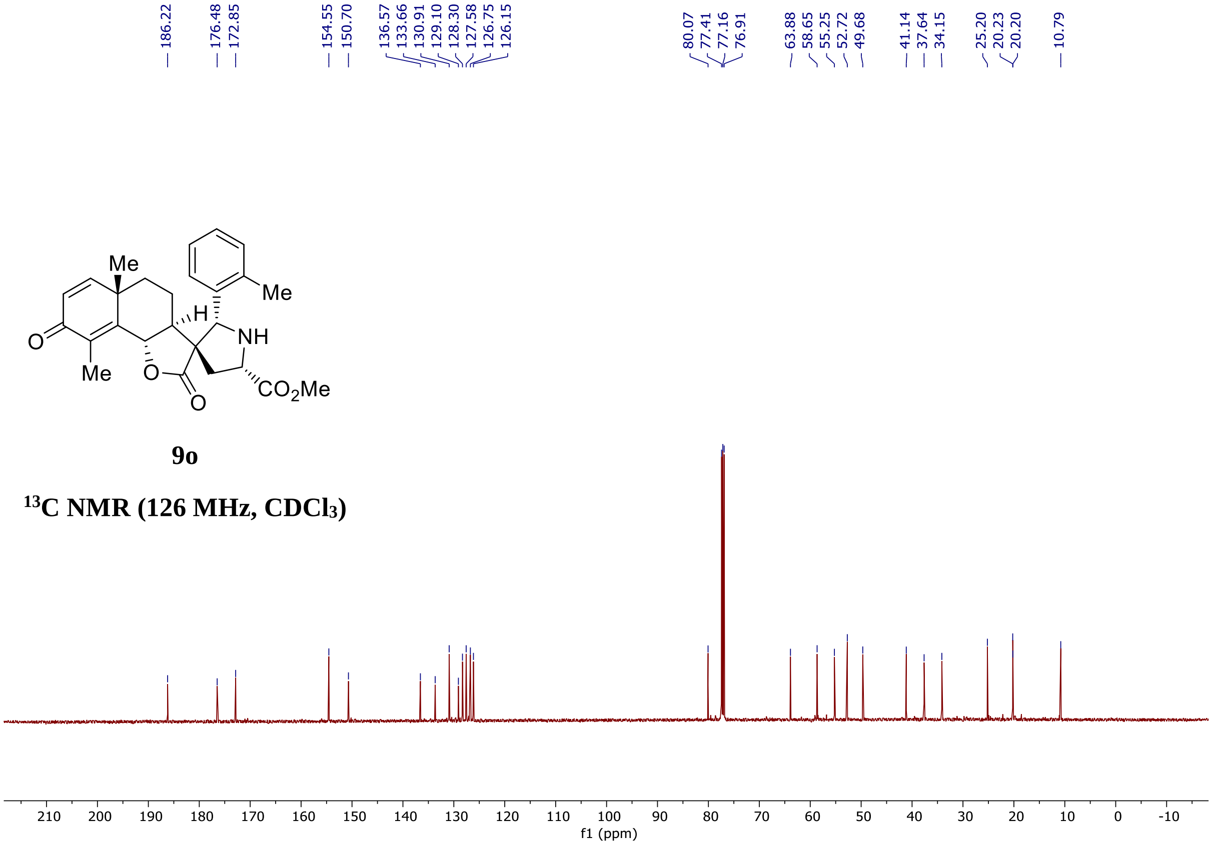

9o

^{13}C NMR (126 MHz, $CDCl_3$)

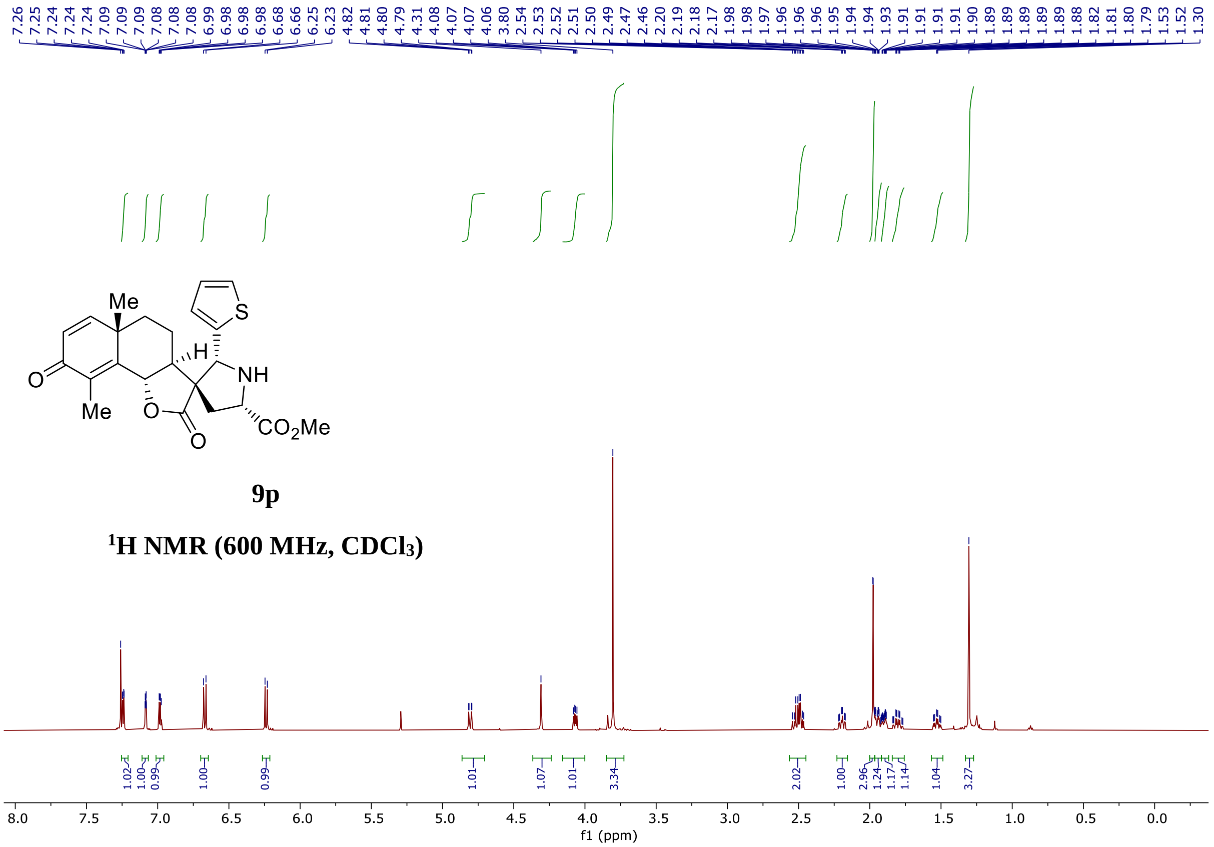

9p

^{1}H NMR (600 MHz, CDCl$_3$)

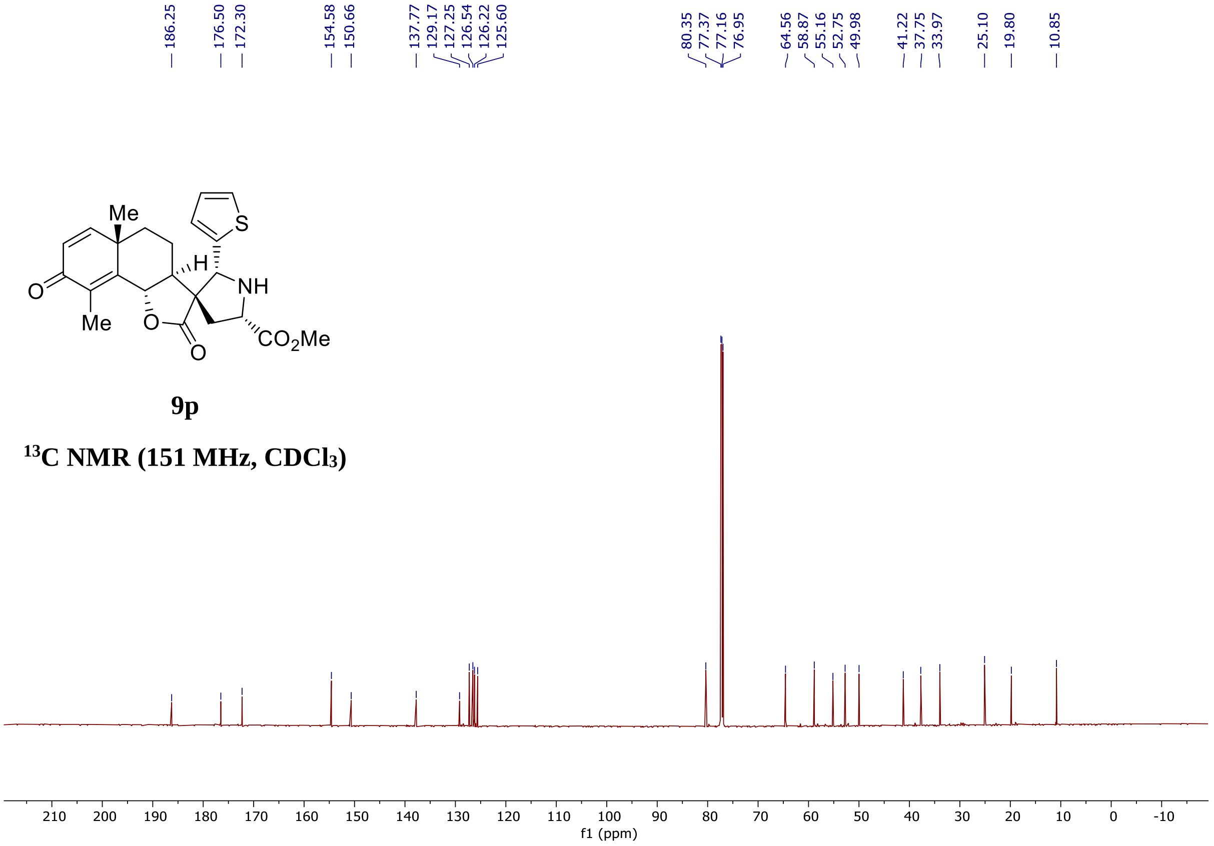

9p

^{13}C NMR (151 MHz, CDCl$_3$)

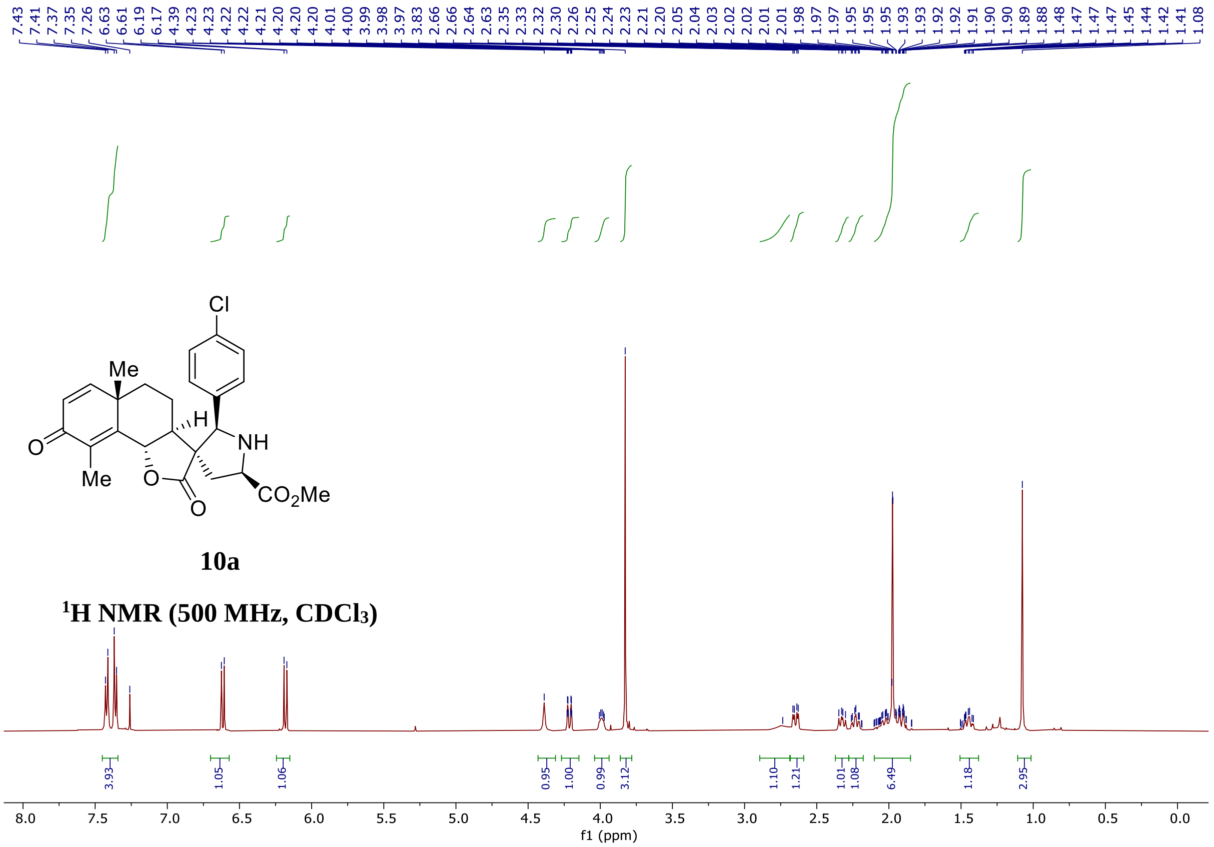

10a

^{1}H NMR (500 MHz, CDCl3)

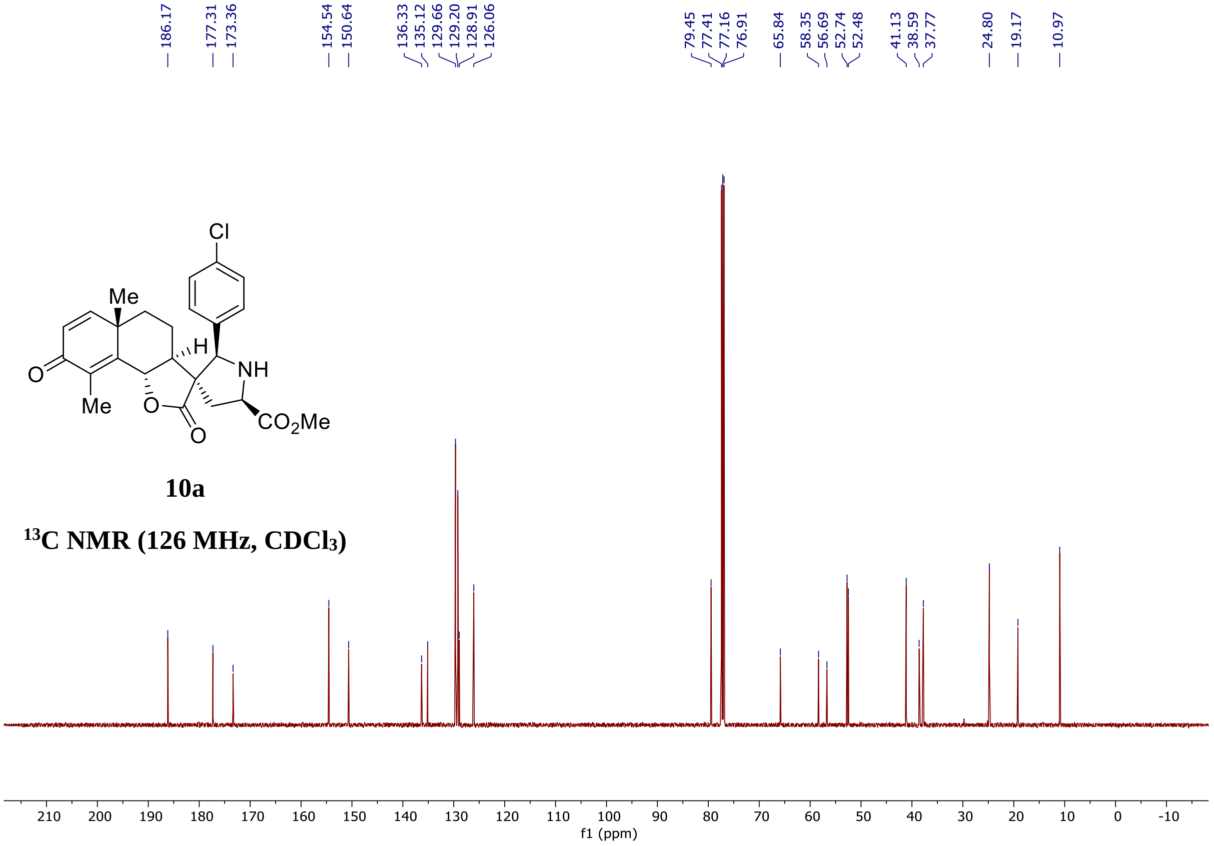

10a

^{13}C NMR (126 MHz, CDCl3)

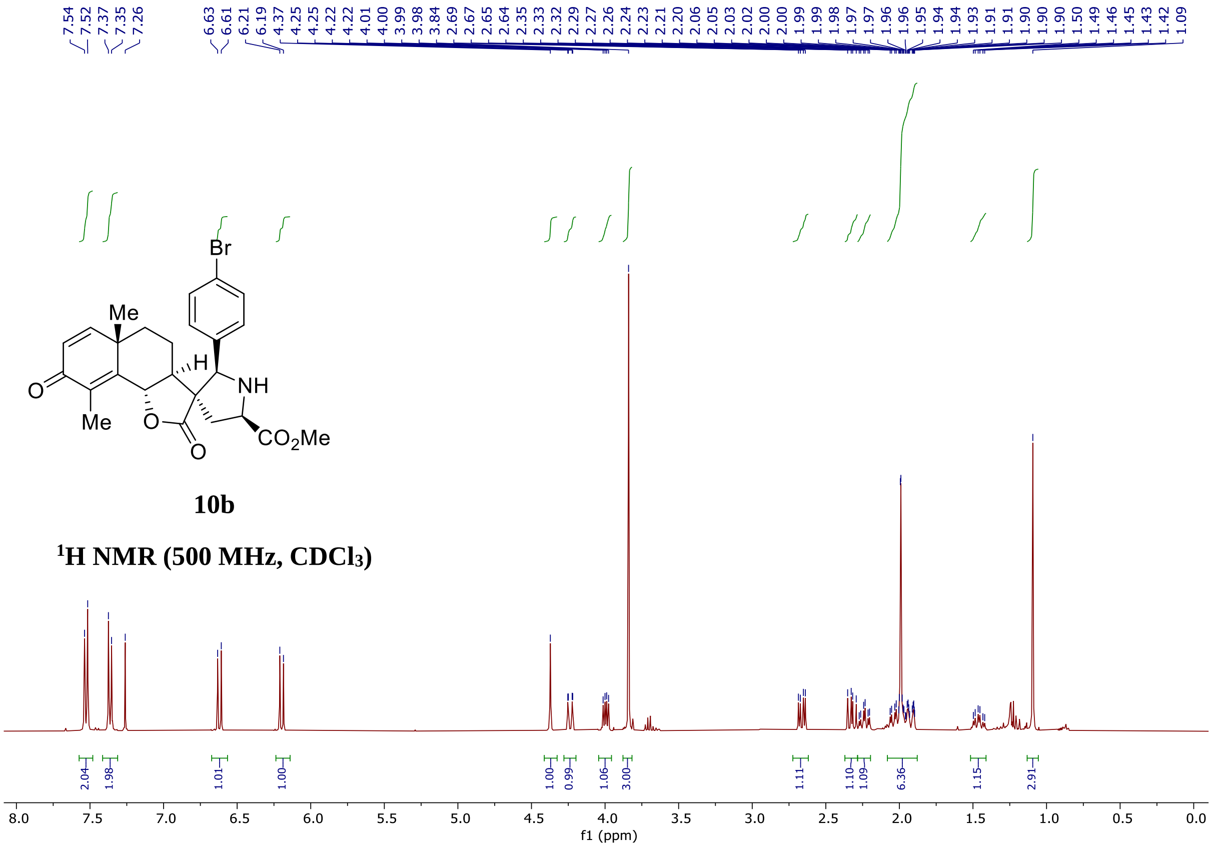

10b

^{1}H NMR (500 MHz, CDCl$_3$)

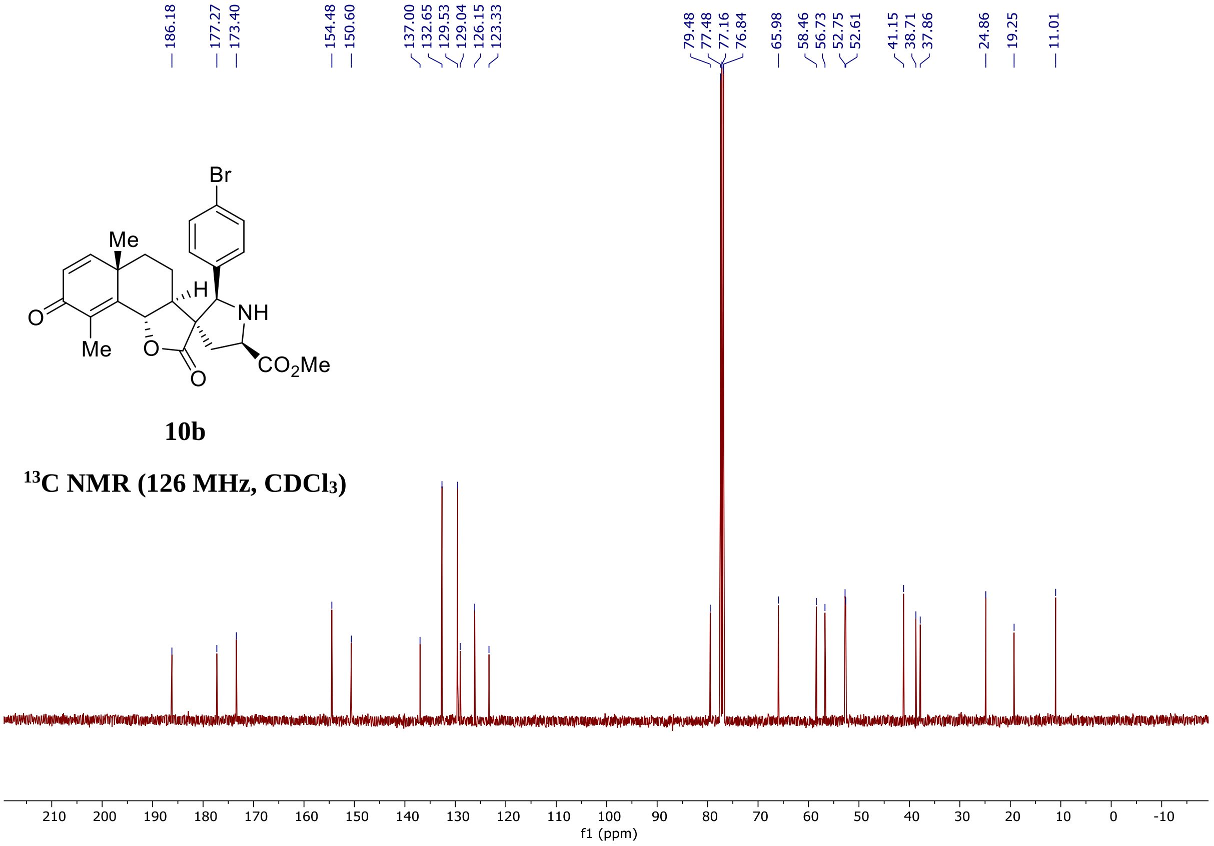

10b

^{13}C NMR (126 MHz, CDCl$_3$)

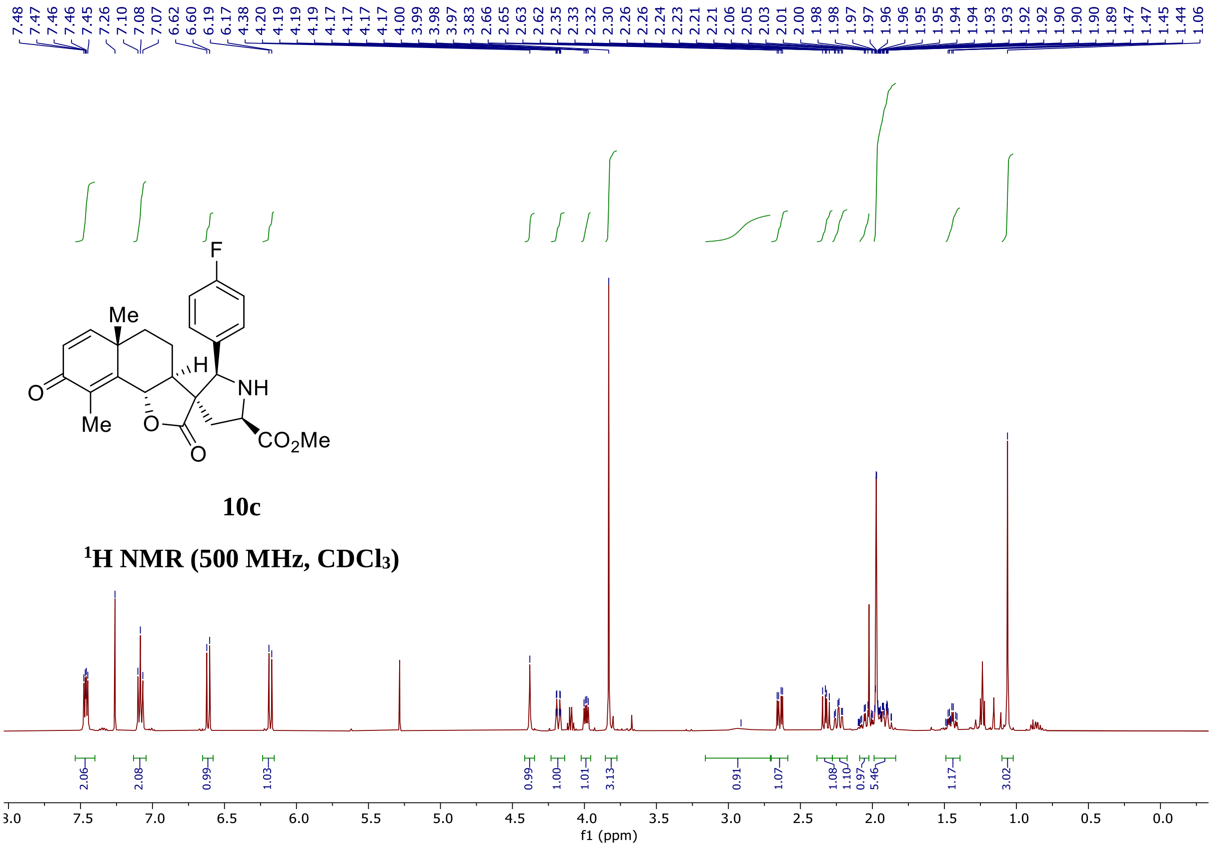

10c

^{1}H NMR (500 MHz, $CDCl_3$)

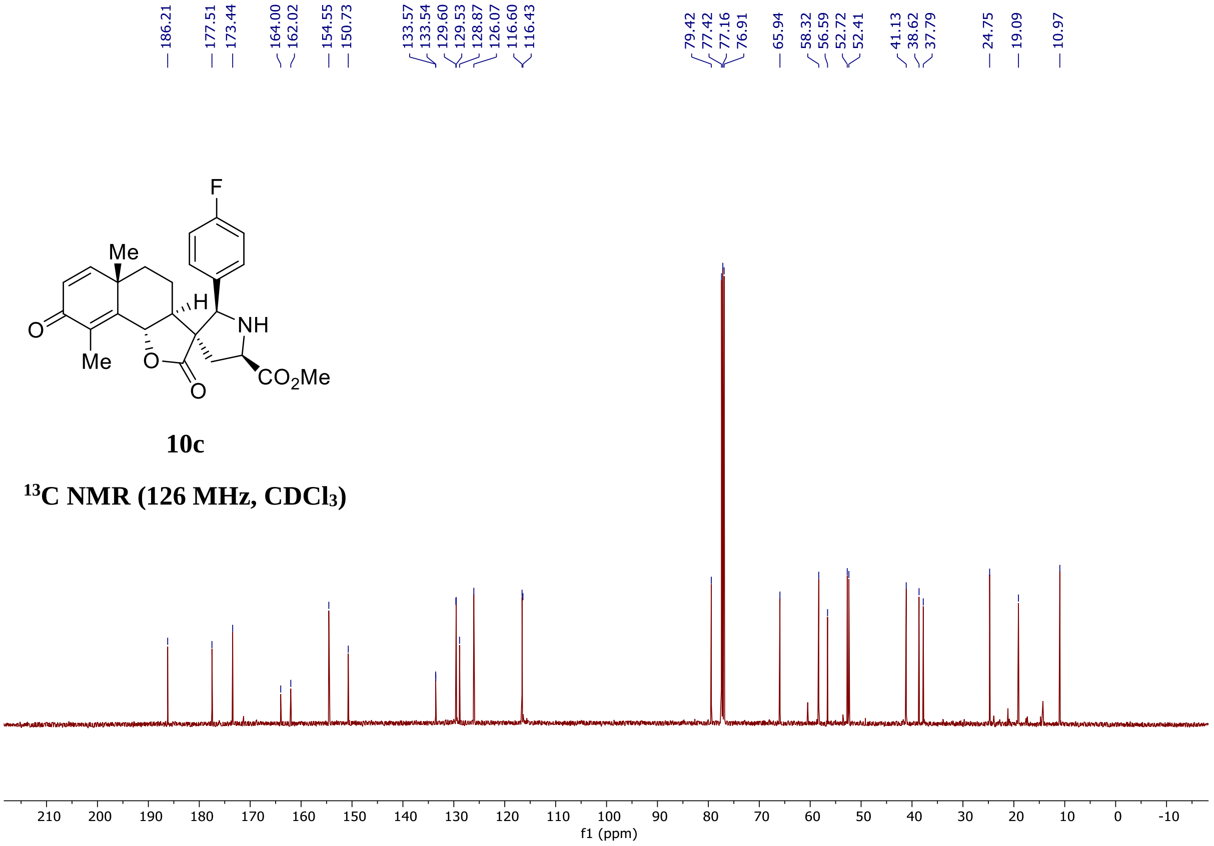

10c

^{13}C NMR (126 MHz, $CDCl_3$)

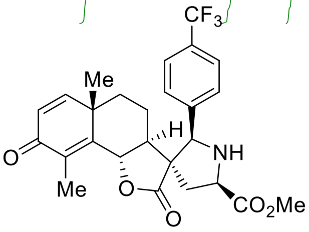

10d

^{1}H NMR (500 MHz, CDCl₃)

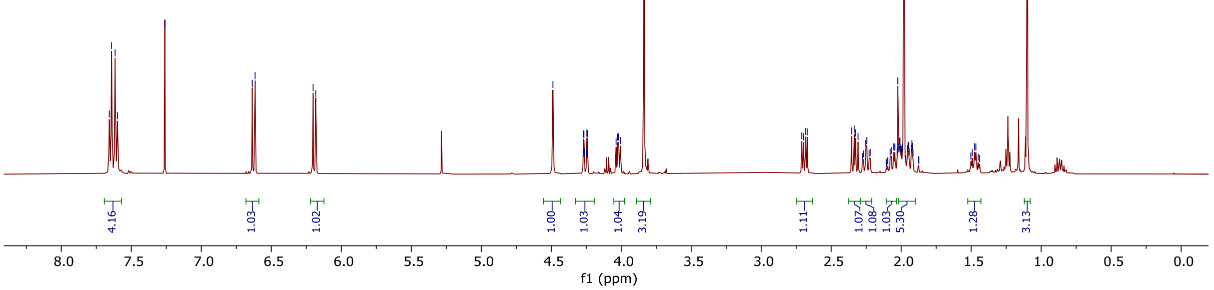

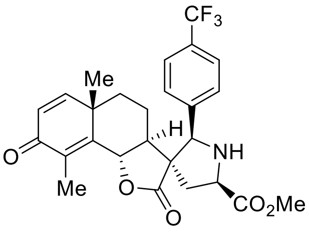

10d

^{13}C NMR (126 MHz, CDCl₃)

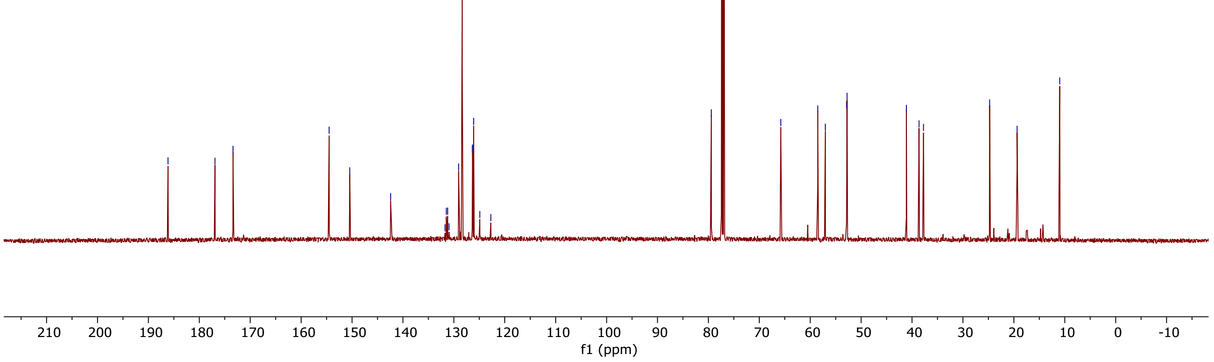

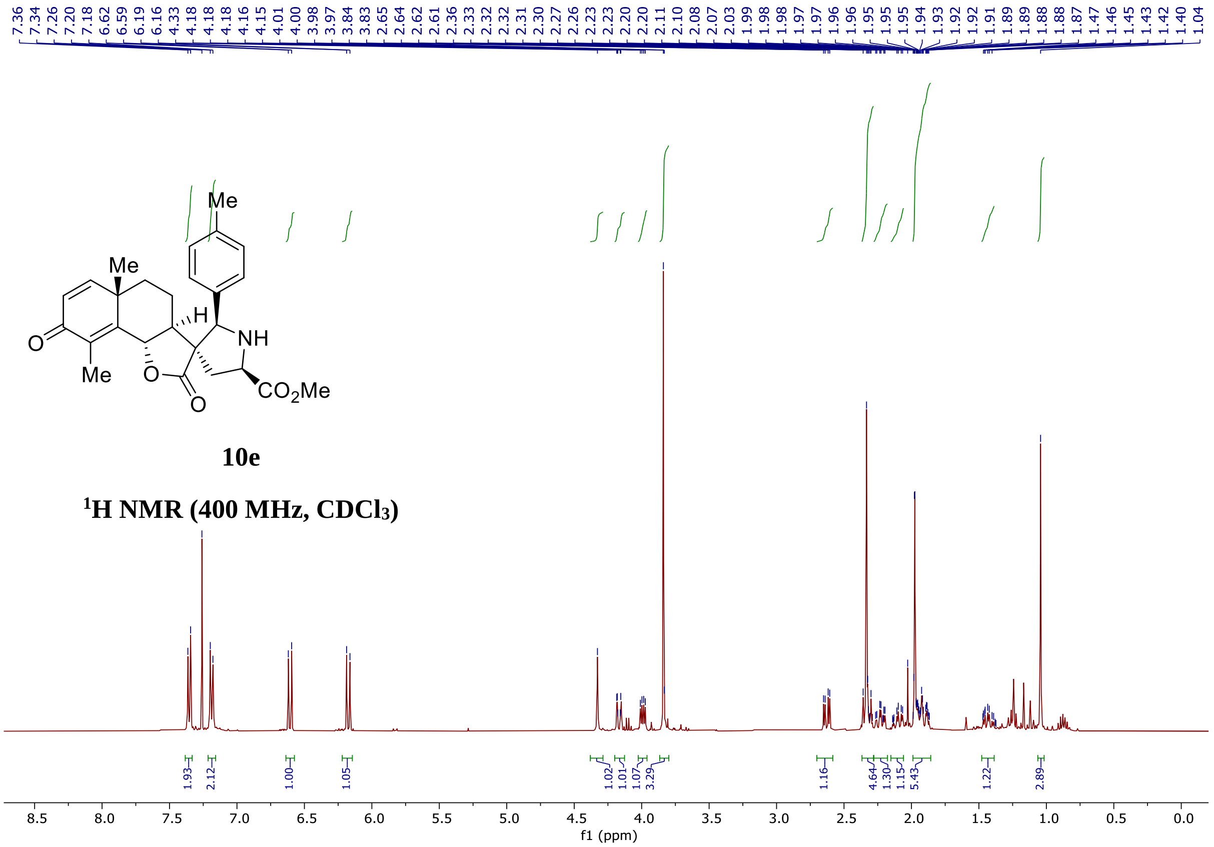
7.36 7.34 7.26 7.20 7.18 6.62 6.59 6.19 6.16 4.33 4.18 4.18 4.18 4.16 4.15 4.01 4.00 3.98 3.97 3.84 3.83 2.65 2.64 2.62 2.61 2.36 2.33 2.32 2.32 2.31 2.30 2.27 2.26 2.23 2.23 2.20 2.20 2.11 2.10 2.08 2.07 2.03 1.99 1.98 1.98 1.97 1.97 1.96 1.95 1.95 1.95 1.95 1.94 1.93 1.92 1.92 1.91 1.89 1.88 1.88 1.87 1.47 1.46 1.45 1.43 1.42 1.40 1.04
Me
Me
H
NH
O
Me
O
O
CO_2Me
10e
^{1}H NMR (400 MHz, $CDCl_3$)
1.93 2.12 1.00 1.05 1.02 1.01 1.07 3.29 1.16 4.64 1.30 1.15 5.43 1.22 2.89
8.5 8.0 7.5 7.0 6.5 6.0 5.5 5.0 4.5 4.0 3.5 3.0 2.5 2.0 1.5 1.0 0.5 0.0
f1 (ppm)

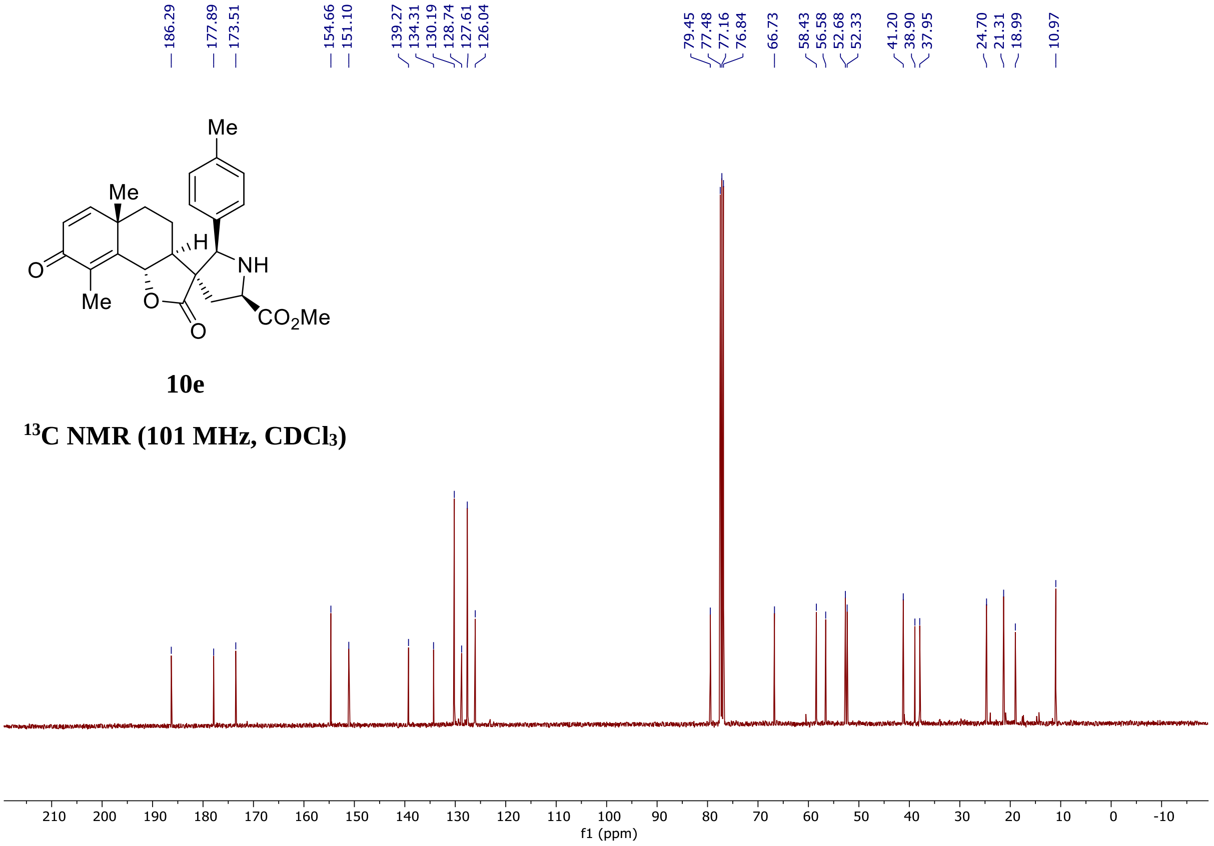
186.29 177.89 173.51 154.66 151.10 139.27 134.31 130.19 128.74 127.61 126.04 79.45 77.48 77.16 76.84 66.73 58.43 56.58 52.68 52.33 41.20 38.90 37.95 24.70 21.31 18.99 10.97
Me
Me
H
NH
O
Me
O
O
CO_2Me
10e
^{13}C NMR (101 MHz, $CDCl_3$)
210 200 190 180 170 160 150 140 130 120 110 100 90 80 70 60 50 40 30 20 10 0 -10
f1 (ppm)

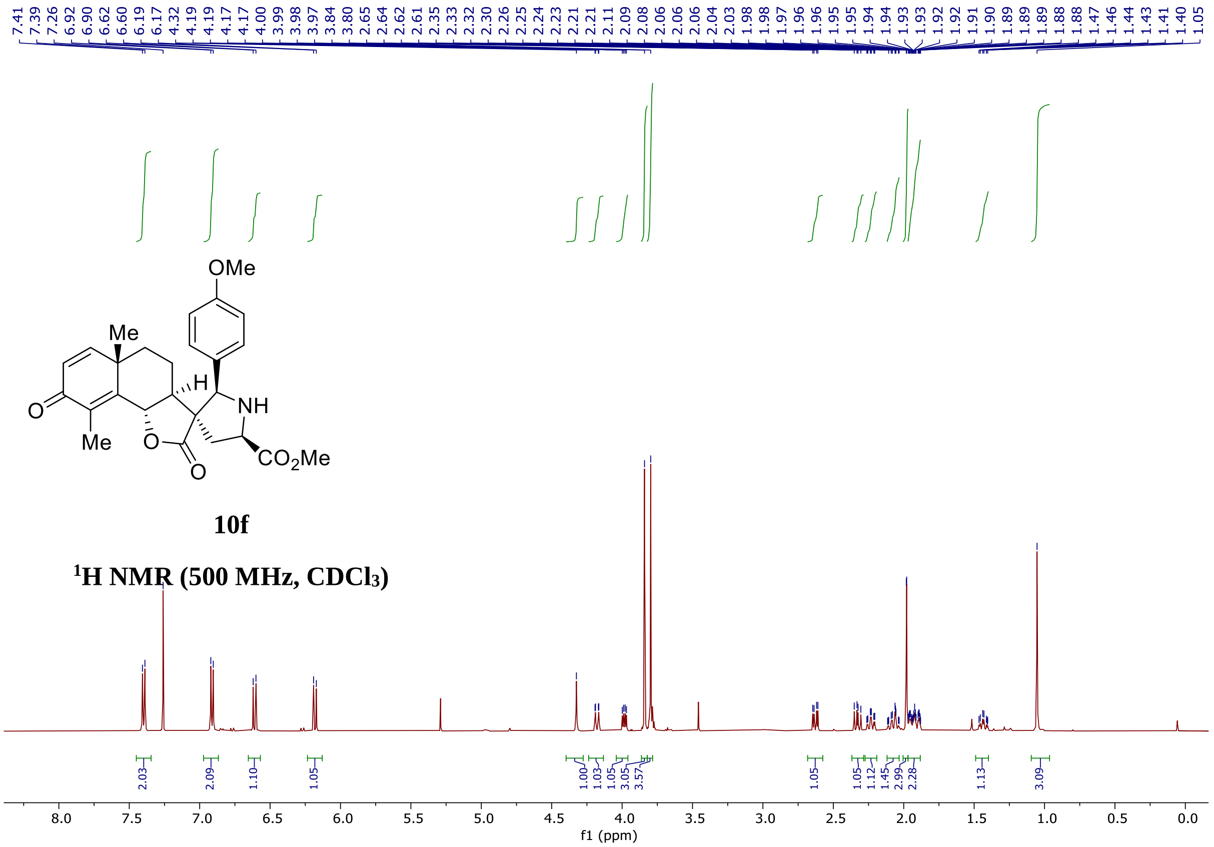

10f

^{1}H NMR (500 MHz, CDCl$_3$)

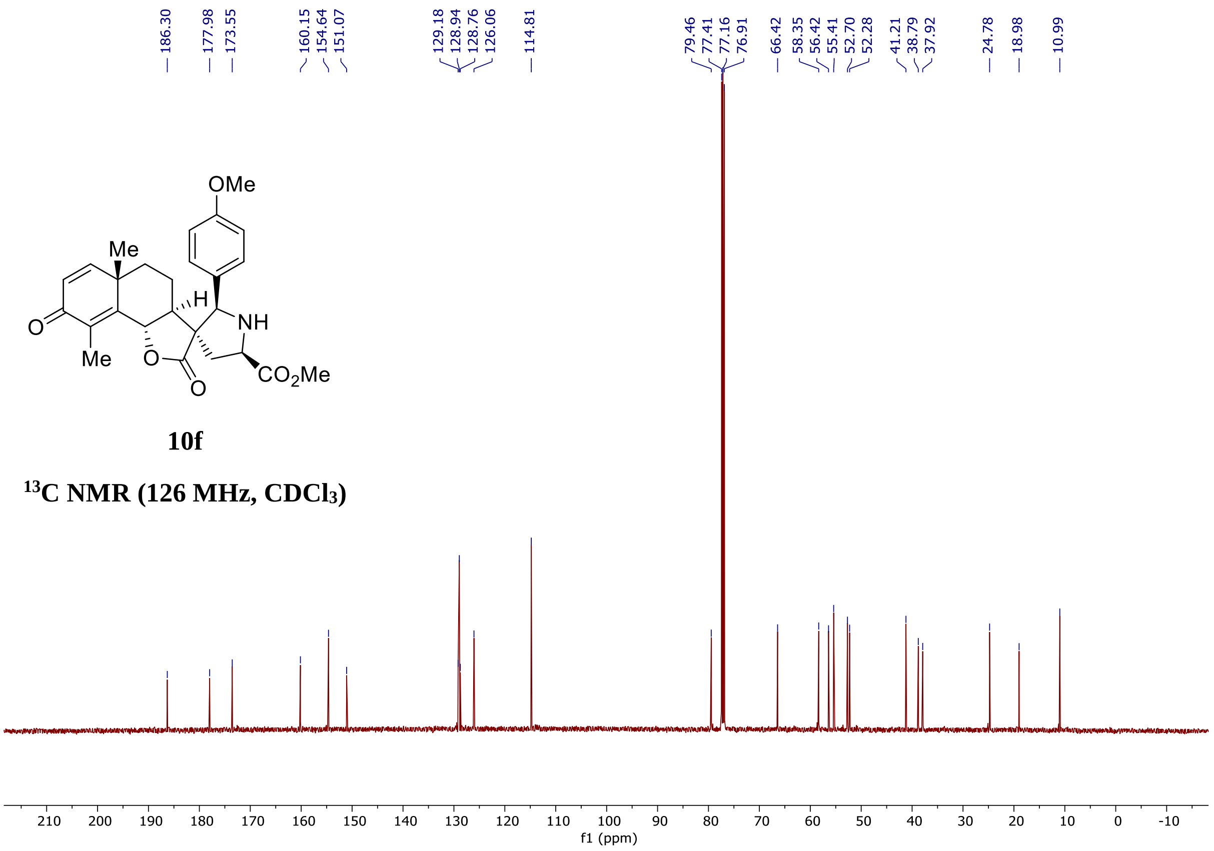

10f

^{13}C NMR (126 MHz, CDCl$_3$)

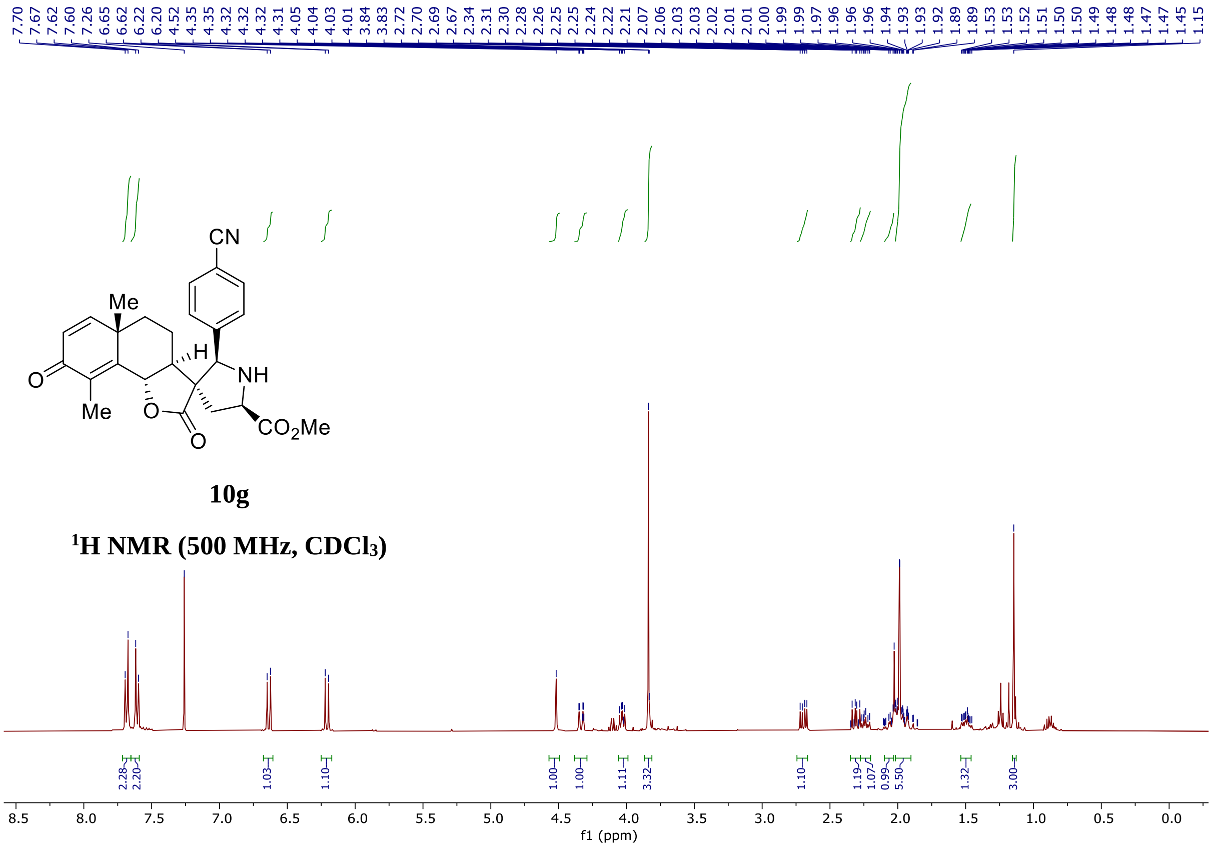

10g

^{1}H NMR (500 MHz, CDCl$_3$)

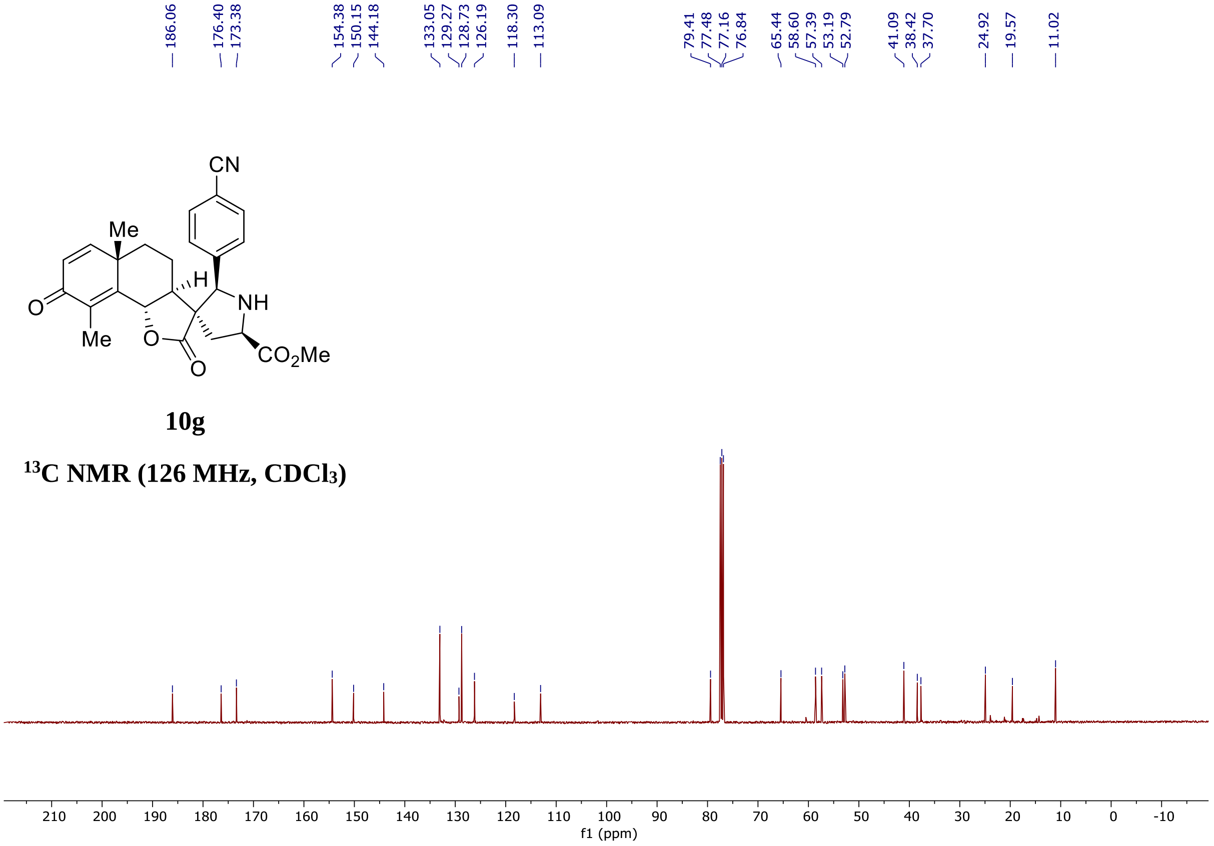

10g

^{13}C NMR (126 MHz, CDCl$_3$)

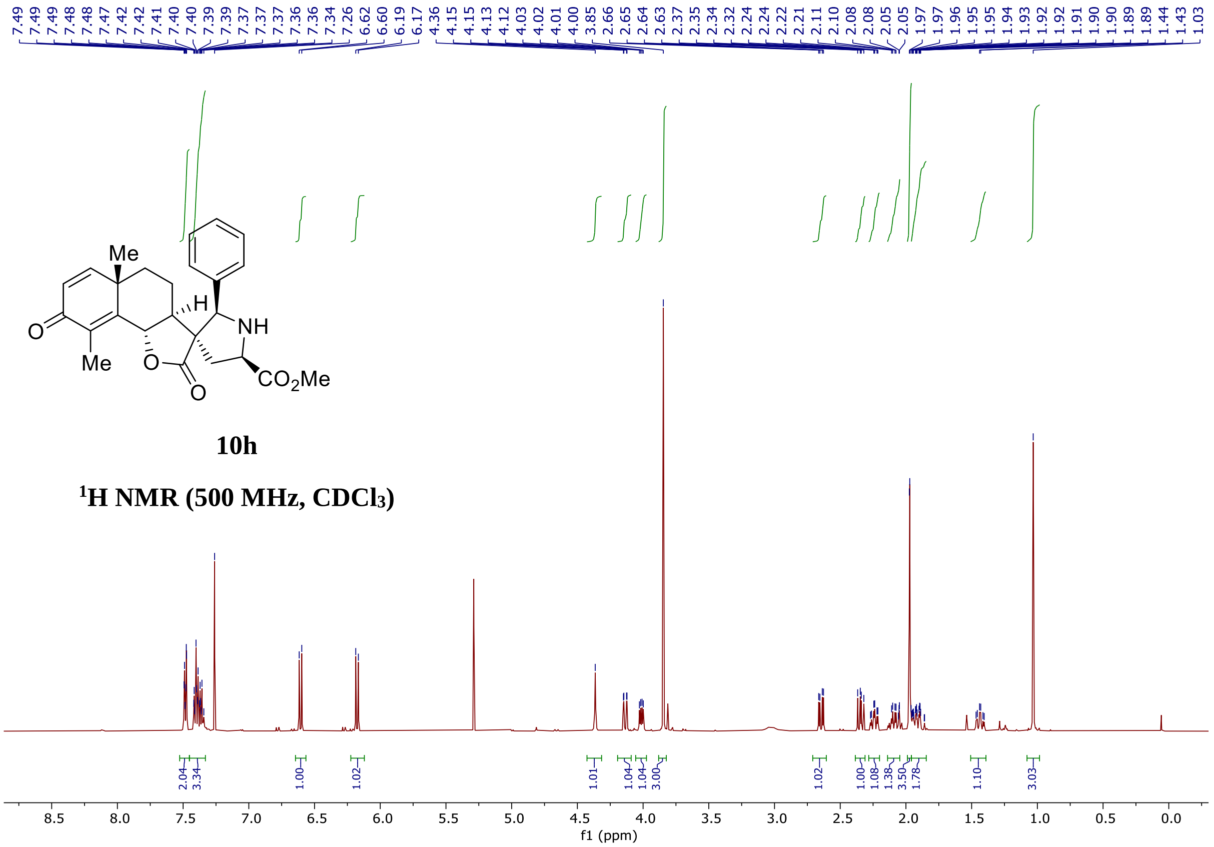

10h

^{1}H NMR (500 MHz, CDCl$_3$)

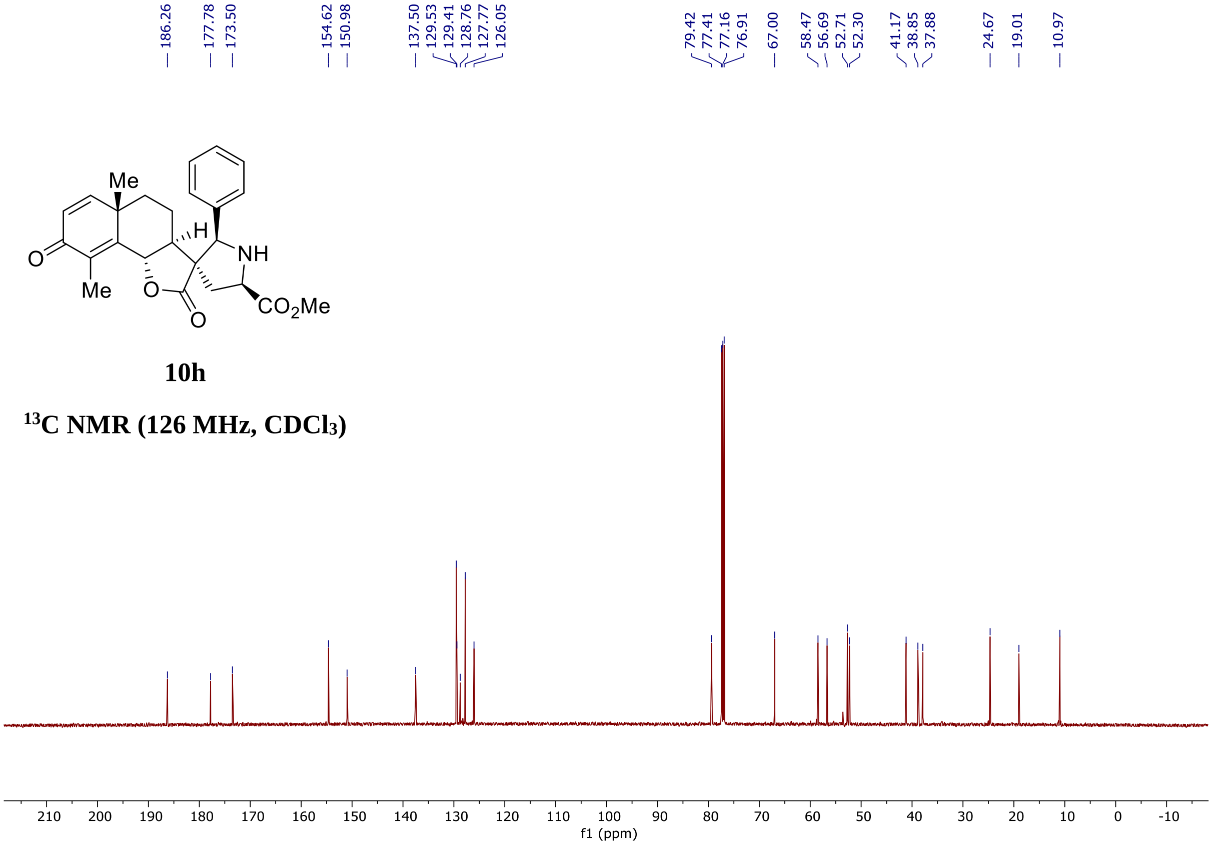

10h

^{13}C NMR (126 MHz, CDCl$_3$)

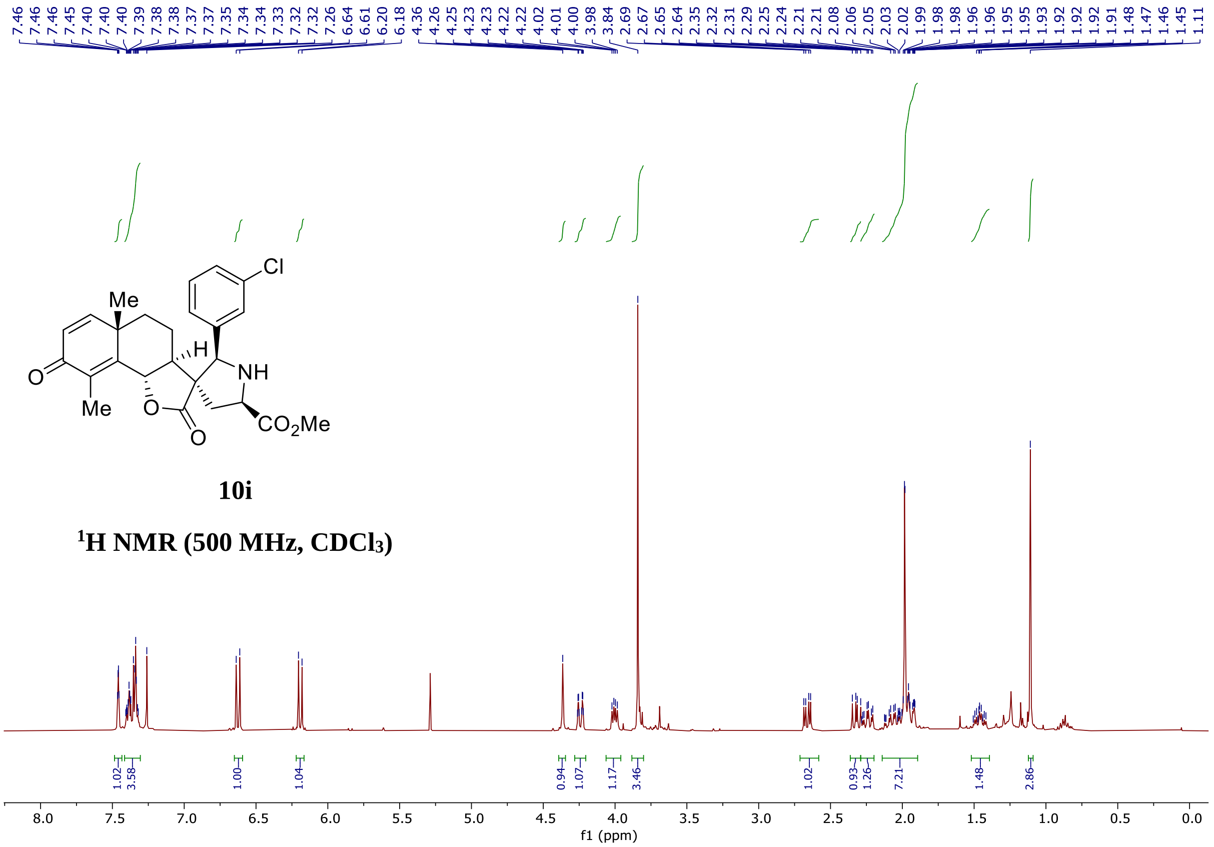
7.46 7.46 7.45 7.40 7.40 7.40 7.39 7.38 7.38 7.37 7.37 7.35 7.34 7.34 7.33 7.32 7.32 7.26 6.64 6.61 6.20 6.18 4.36 4.26 4.25 4.23 4.23 4.22 4.22 4.02 4.01 4.00 3.98 3.84 2.69 2.67 2.65 2.64 2.35 2.32 2.31 2.29 2.25 2.24 2.21 2.21 2.08 2.06 2.05 2.03 2.02 1.99 1.98 1.98 1.96 1.96 1.95 1.95 1.93 1.92 1.92 1.92 1.91 1.48 1.47 1.46 1.45 1.11
Cl
Me
H
NH
O
Me
O
O
CO_2Me
10i
^{1}H NMR (500 MHz, $CDCl_3$)
1.02 3.58 1.00 1.04 0.94 1.07 1.17 3.46 1.02 0.93 1.26 7.21 1.48 2.86
8.0 7.5 7.0 6.5 6.0 5.5 5.0 4.5 4.0 3.5 3.0 2.5 2.0 1.5 1.0 0.5 0.0
f1 (ppm)

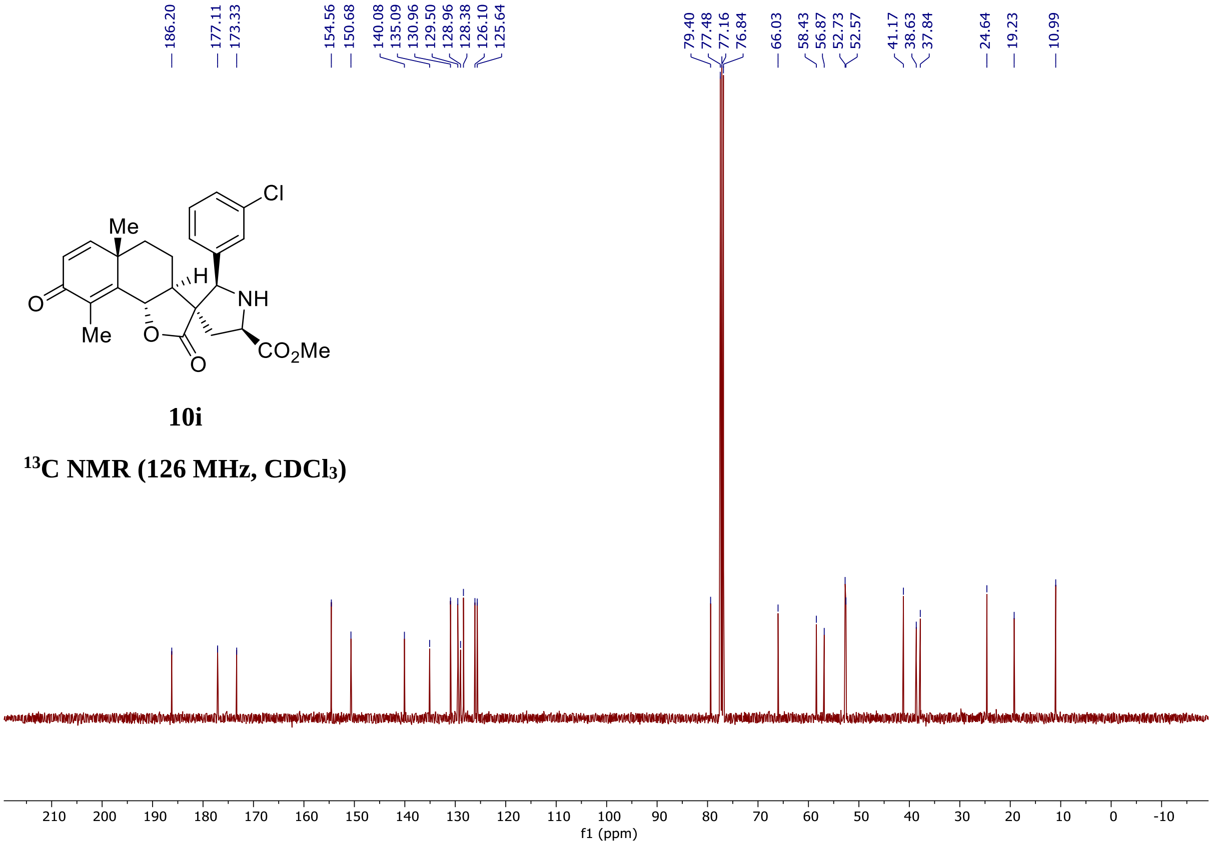
186.20
177.11 173.33
154.56 150.68
140.08 135.09 130.96 129.50 128.96 128.38 126.10 125.64
79.40 77.48 77.16 76.84
66.03
58.43 56.87 52.73 52.57
41.17 38.63 37.84
24.64 19.23 10.99
Cl
Me
H
NH
O
Me
O
O
CO_2Me
10i
^{13}C NMR (126 MHz, $CDCl_3$)
210 200 190 180 170 160 150 140 130 120 110 100 90 80 70 60 50 40 30 20 10 0 -10
f1 (ppm)

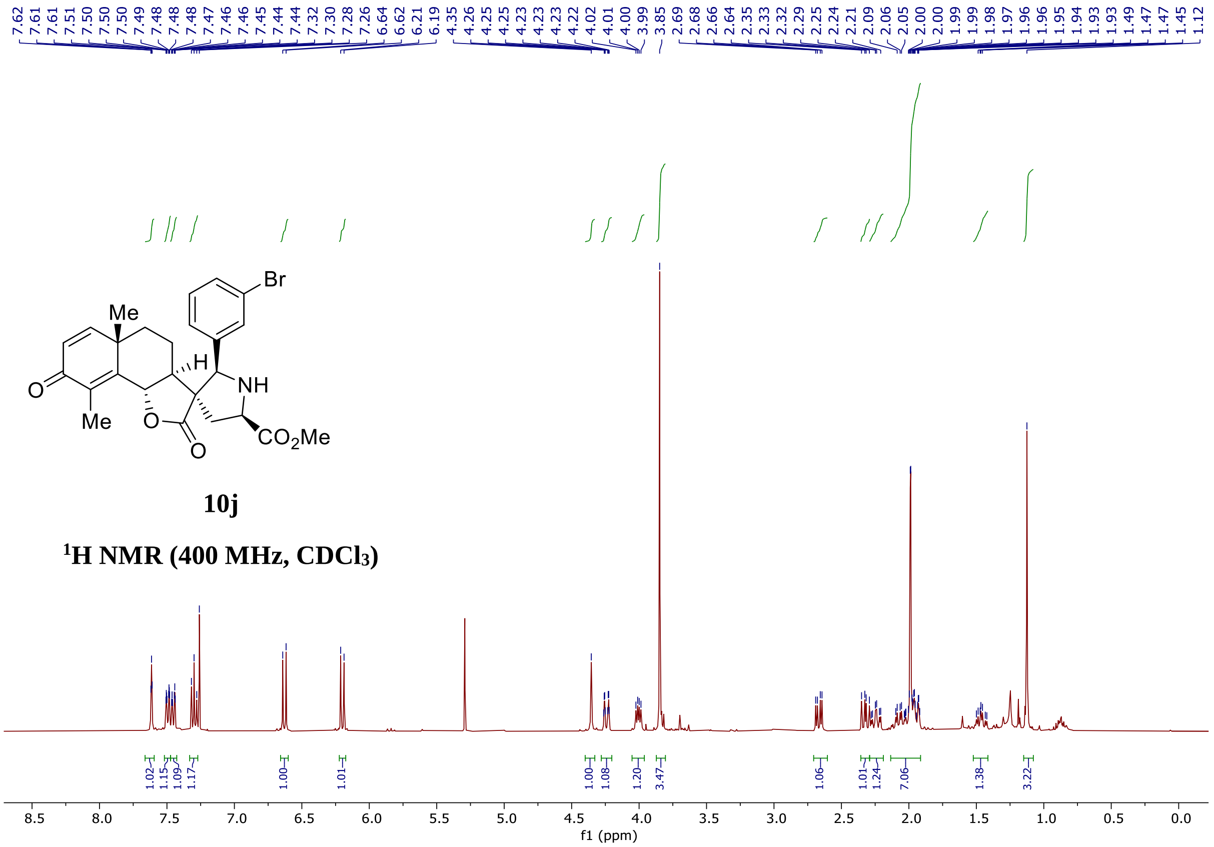
7.62 7.61 7.51 7.50 7.50 7.49 7.48 7.48 7.48 7.47 7.46 7.45 7.44 7.44 7.32 7.30 7.28 7.26 6.64 6.62 6.21 6.19 4.35 4.26 4.25 4.25 4.23 4.23 4.23 4.22 4.02 4.01 4.00 3.99 3.85 2.69 2.68 2.66 2.64 2.35 2.33 2.32 2.29 2.25 2.24 2.21 2.09 2.06 2.05 2.00 1.99 1.99 1.98 1.97 1.96 1.96 1.95 1.94 1.93 1.93 1.49 1.47 1.47 1.45 1.12
Br
Me
H
NH
O
Me
O
O
CO2Me
10j
1H NMR (400 MHz, CDCl3)
1.02 1.15 1.09 1.17 1.00 1.01 1.00 1.08 1.20 3.47 1.06 1.01 1.24 7.06 1.38 3.22
8.5 8.0 7.5 7.0 6.5 6.0 5.5 5.0 4.5 4.0 3.5 3.0 2.5 2.0 1.5 1.0 0.5 0.0
f1 (ppm)

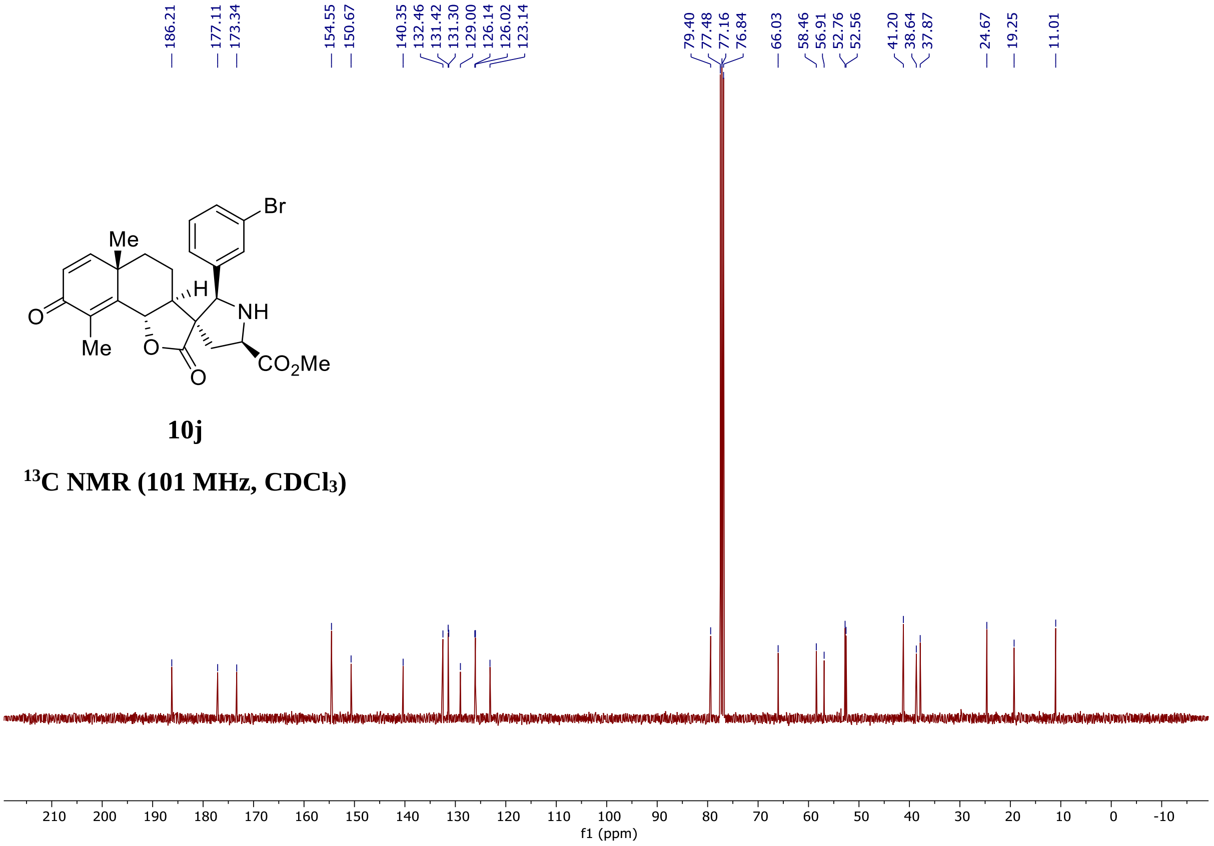
186.21 177.11 173.34 154.55 150.67 140.35 132.46 131.42 131.30 129.00 126.14 126.02 123.14 79.40 77.48 77.16 76.84 66.03 58.46 56.91 52.76 52.56 41.20 38.64 37.87 24.67 19.25 11.01
Br
Me
H
NH
O
Me
O
O
CO2Me
10j
13C NMR (101 MHz, CDCl3)
210 200 190 180 170 160 150 140 130 120 110 100 90 80 70 60 50 40 30 20 10 0 -10
f1 (ppm)

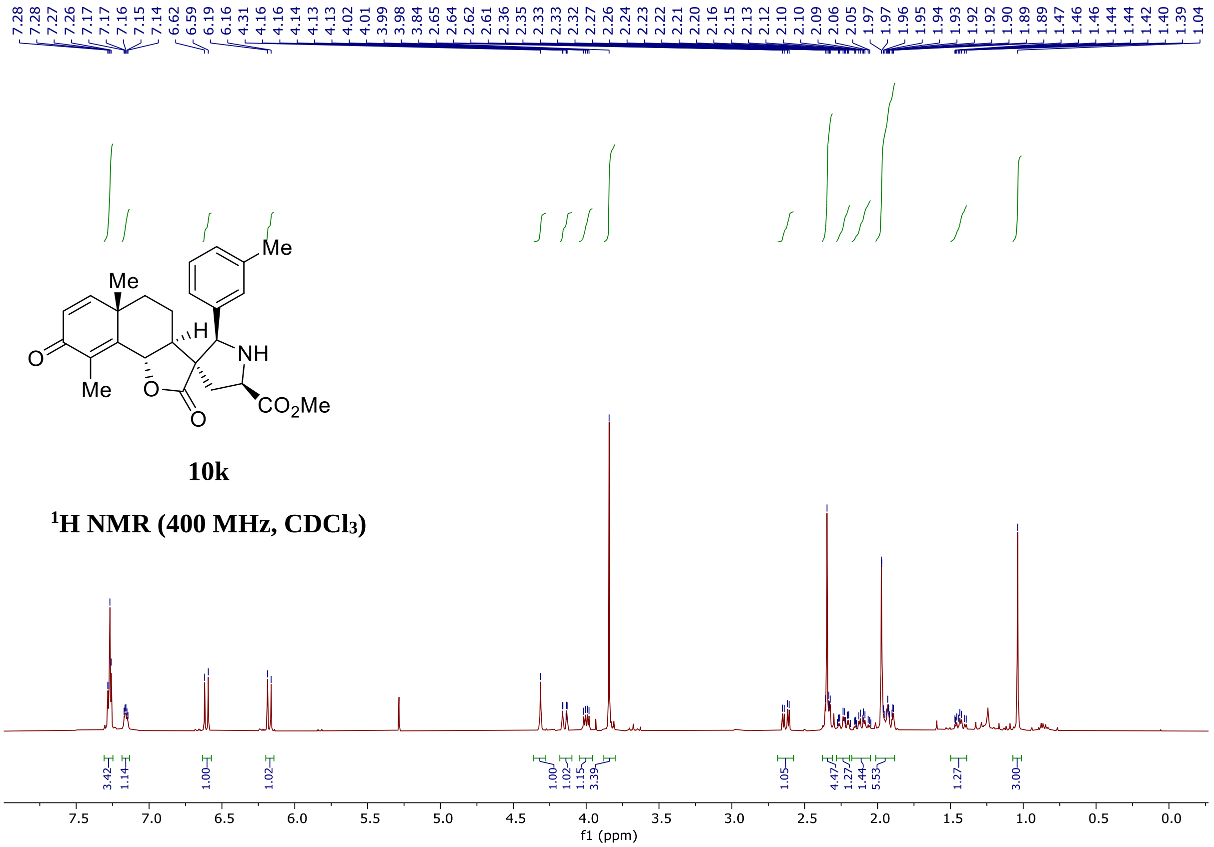

10k

^{1}H NMR (400 MHz, CDCl3)

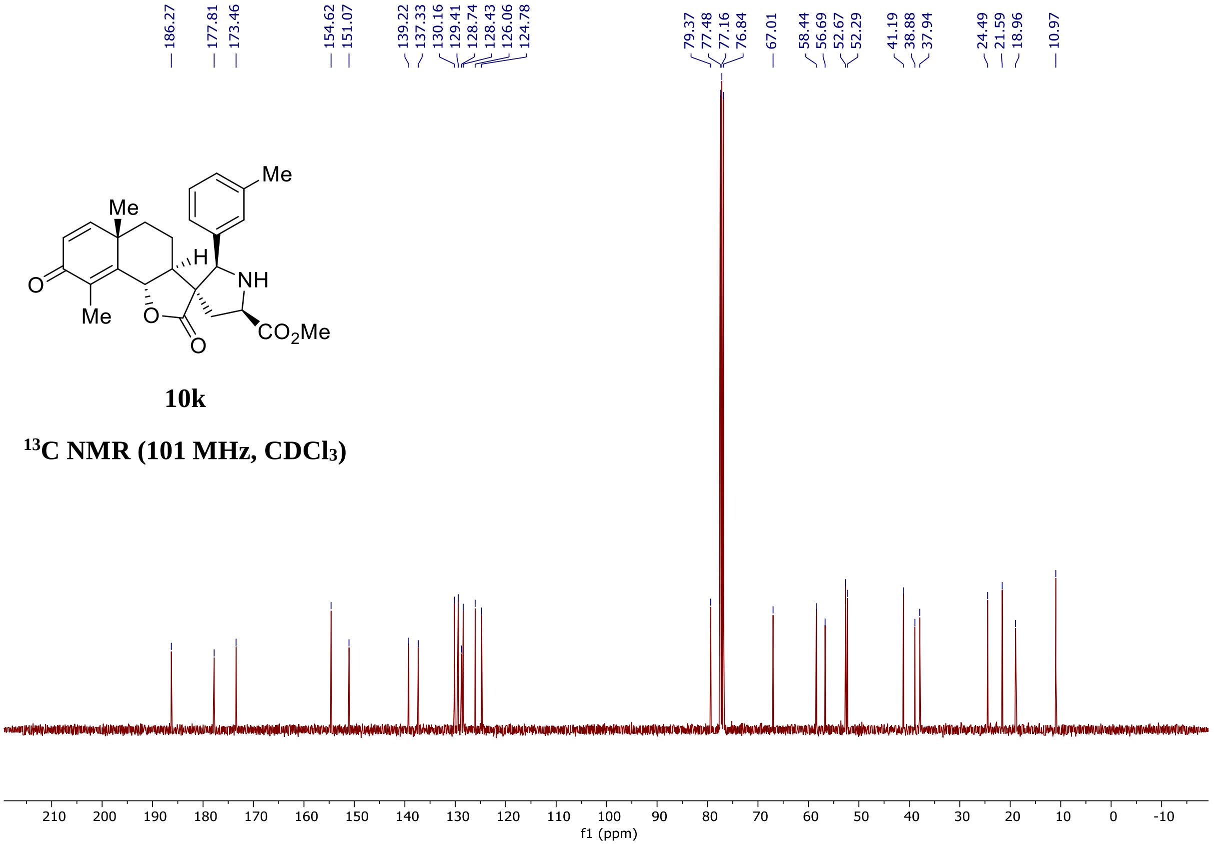

10k

^{13}C NMR (101 MHz, CDCl3)

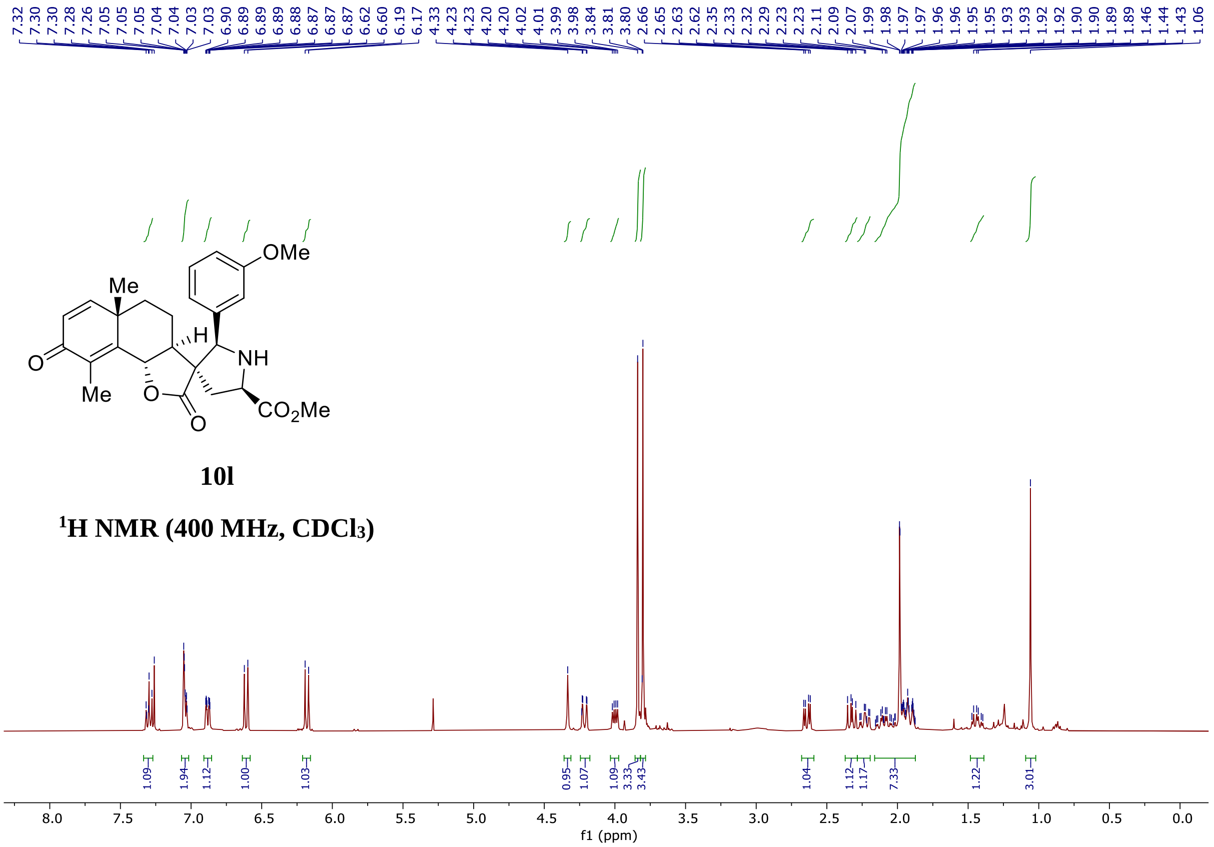

10l

^{1}H NMR (400 MHz, CDCl$_3$)

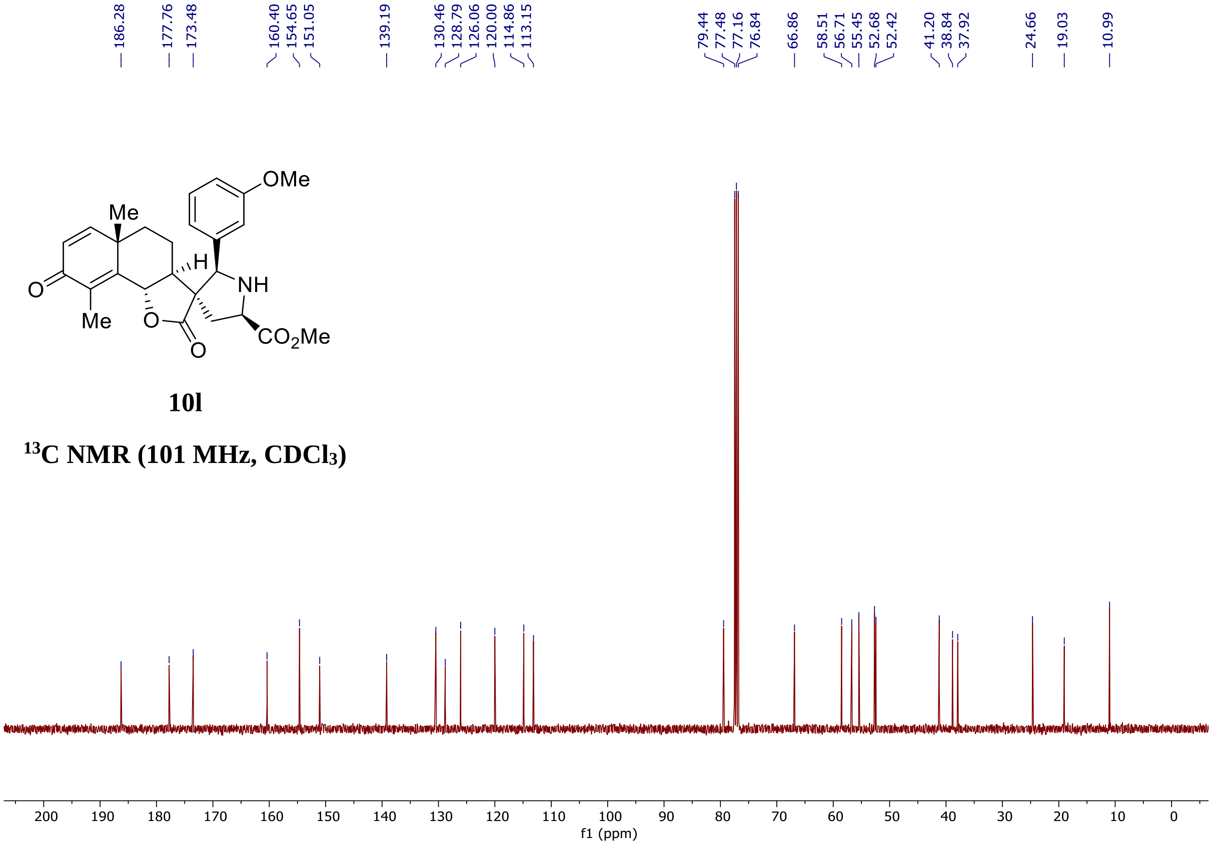

10l

^{13}C NMR (101 MHz, CDCl$_3$)

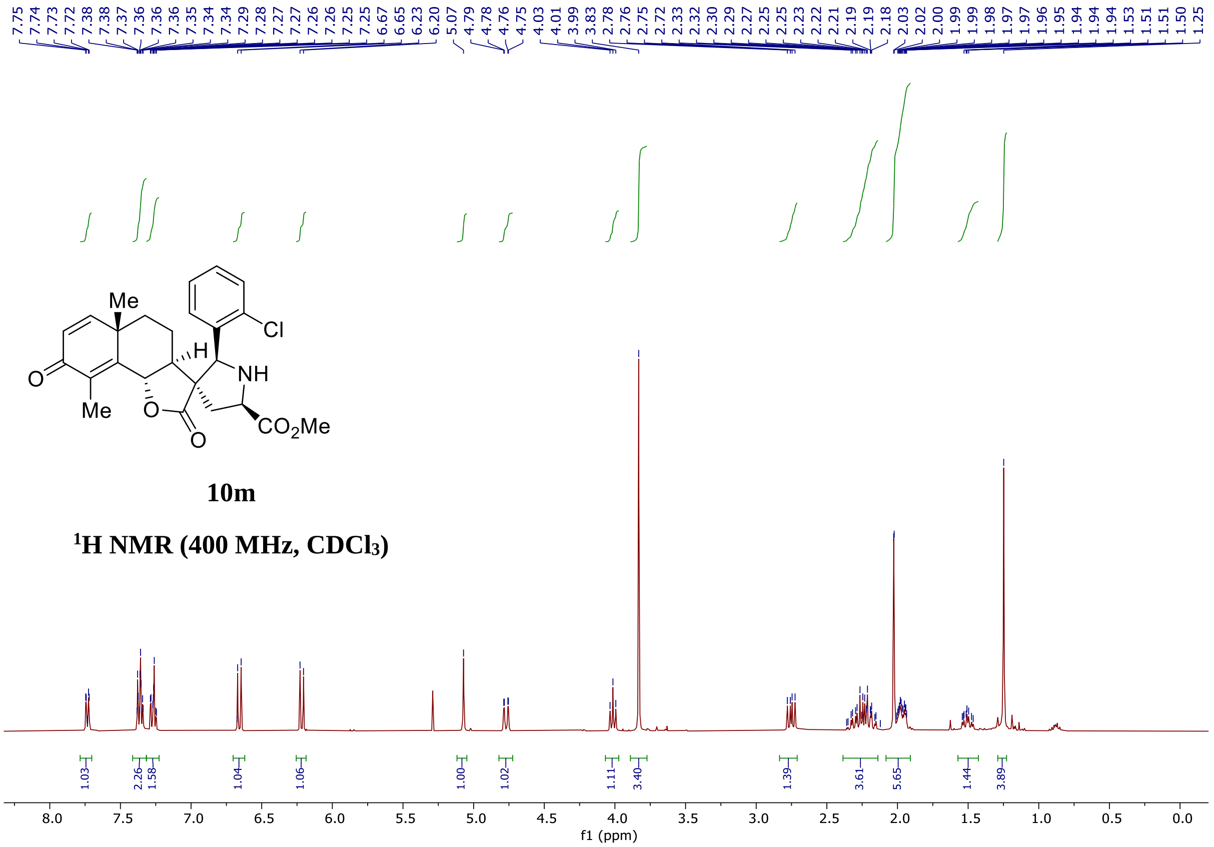

10m

^{1}H NMR (400 MHz, $CDCl_3$)

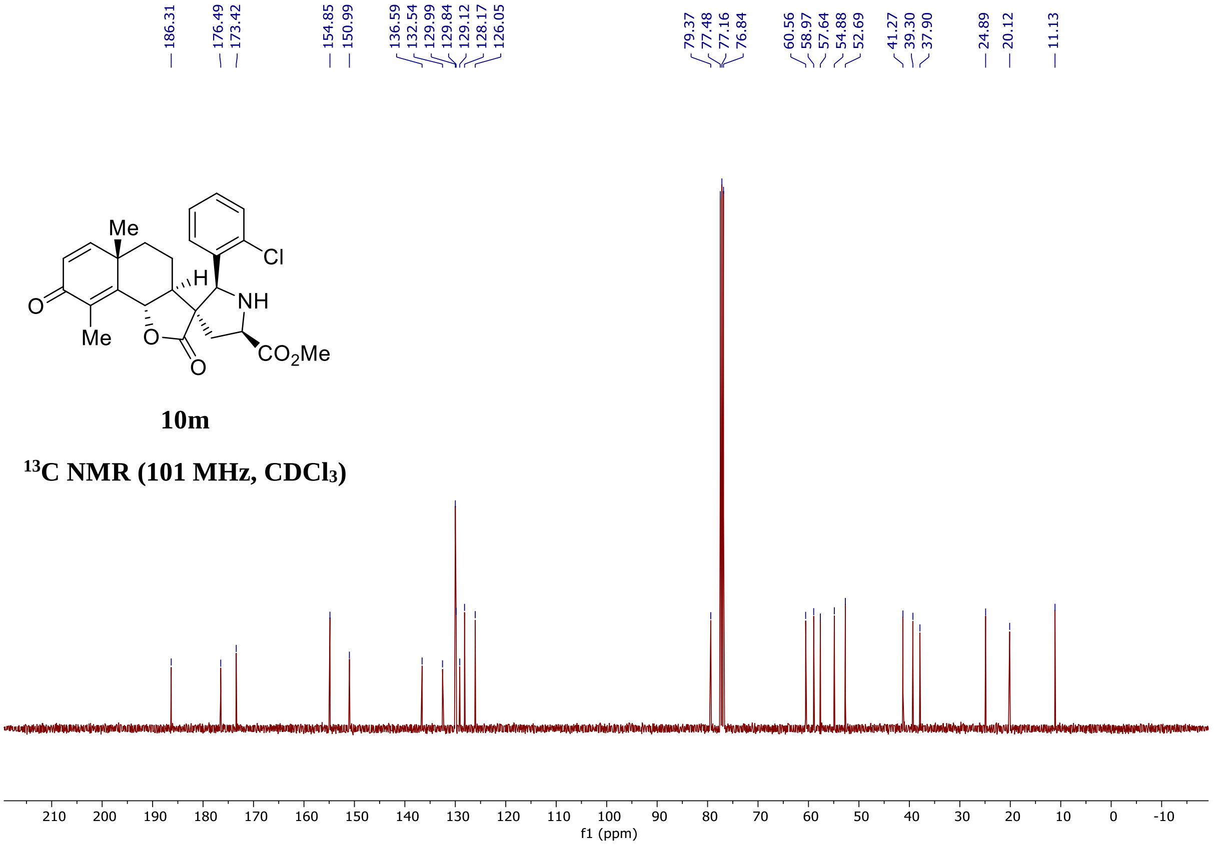

10m

^{13}C NMR (101 MHz, $CDCl_3$)

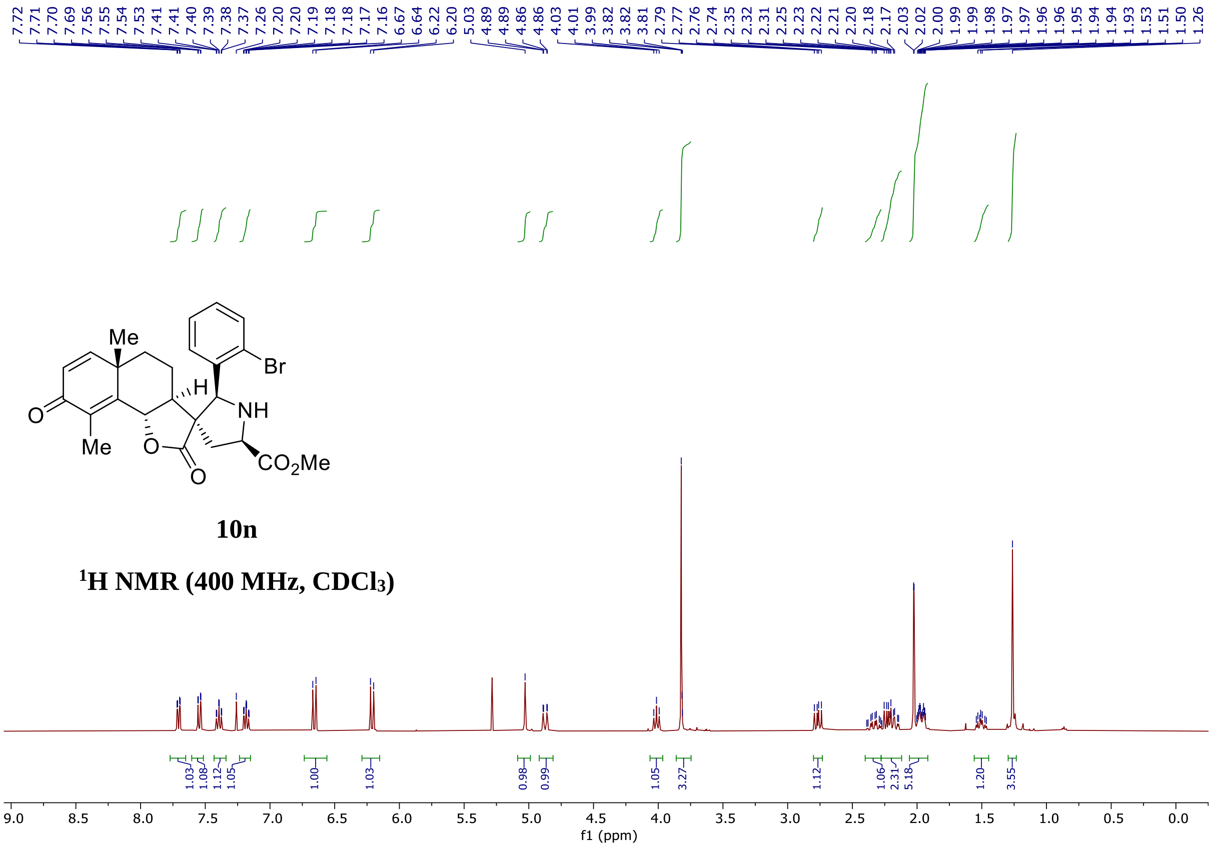

10n

^{1}H NMR (400 MHz, $CDCl_3$)

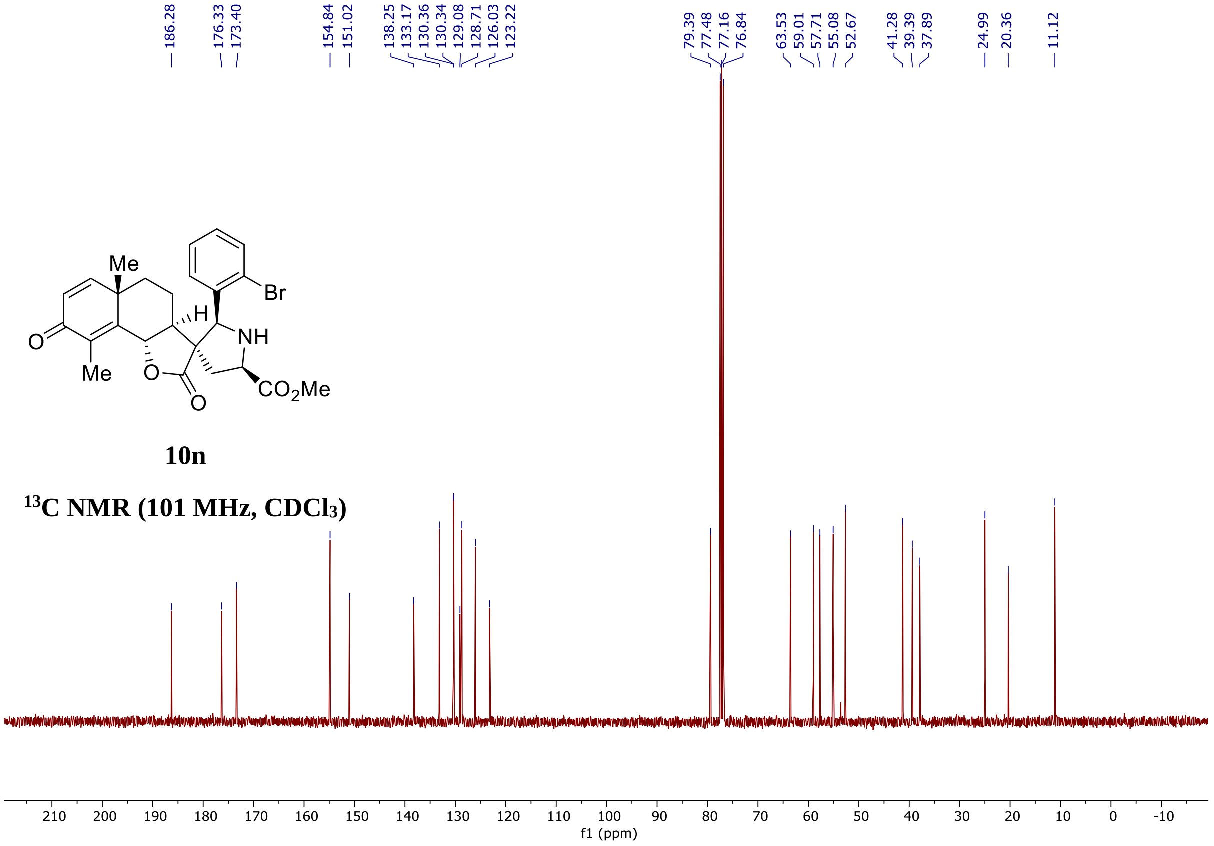

10n

^{13}C NMR (101 MHz, $CDCl_3$)

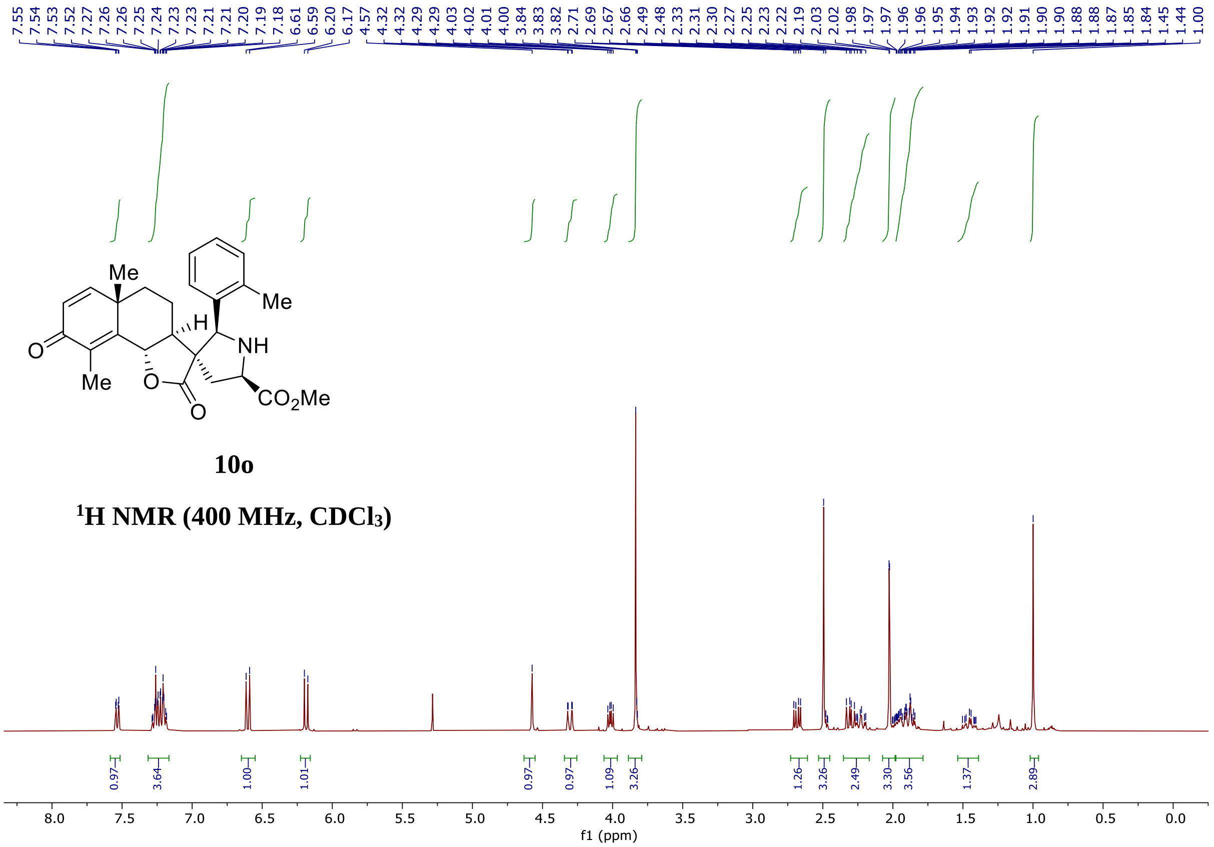

10o

^{1}H NMR (400 MHz, CDCl3)

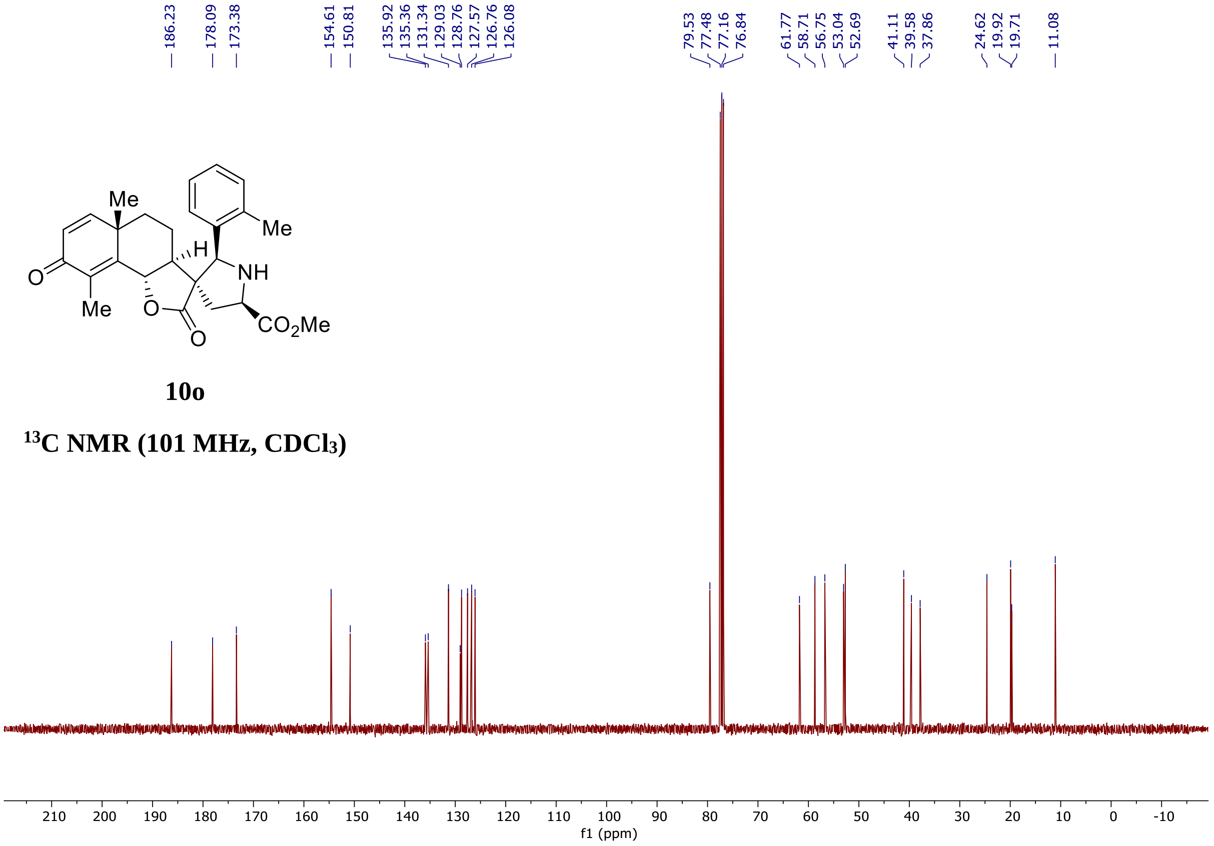

10o

^{13}C NMR (101 MHz, CDCl3)

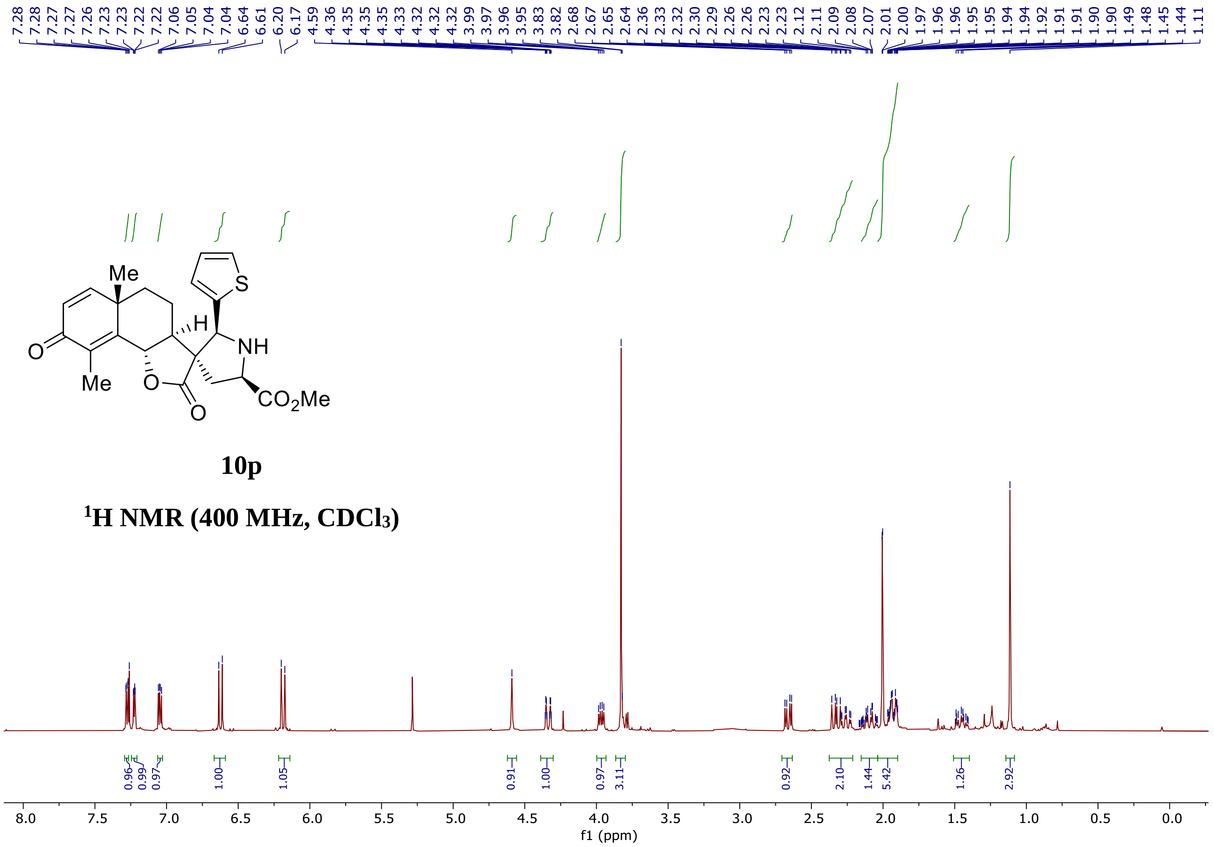

10p

^{1}H NMR (400 MHz, $CDCl_3$)

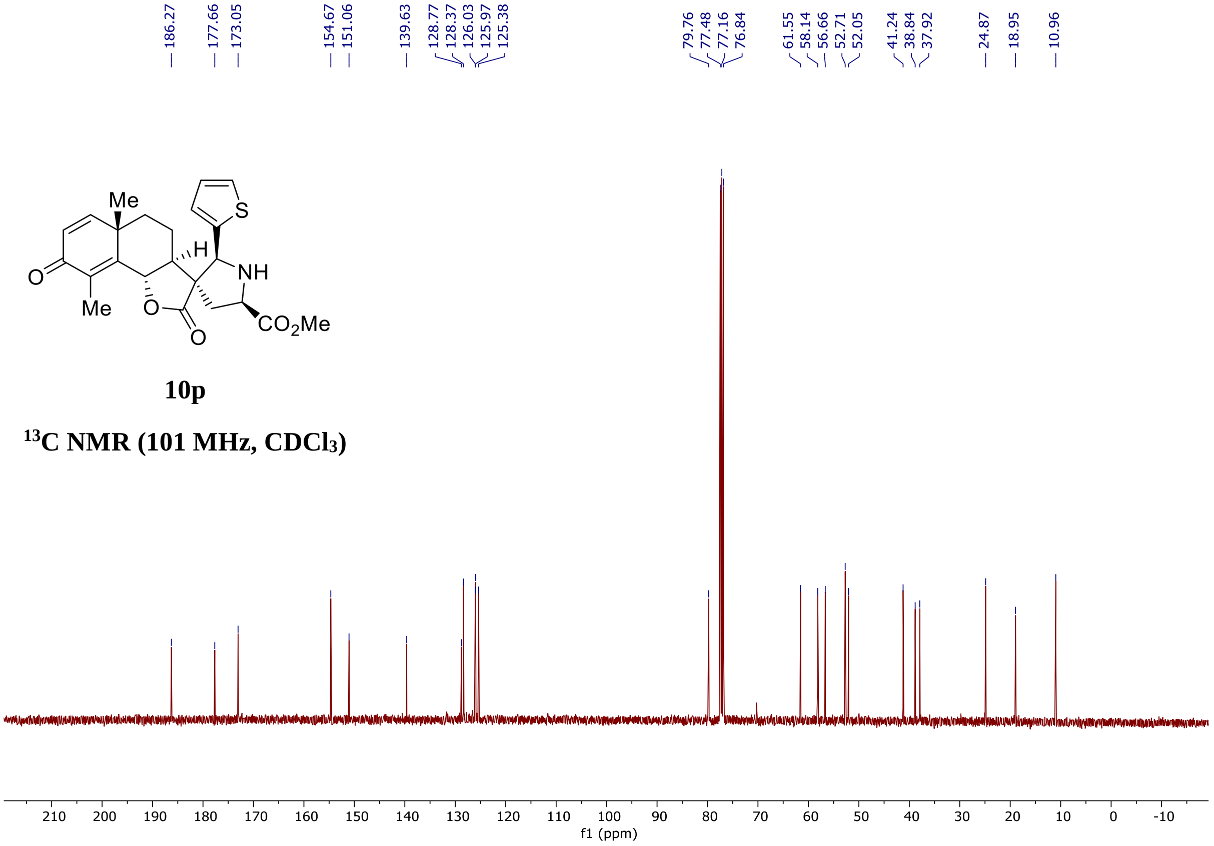

10p

^{13}C NMR (101 MHz, $CDCl_3$)

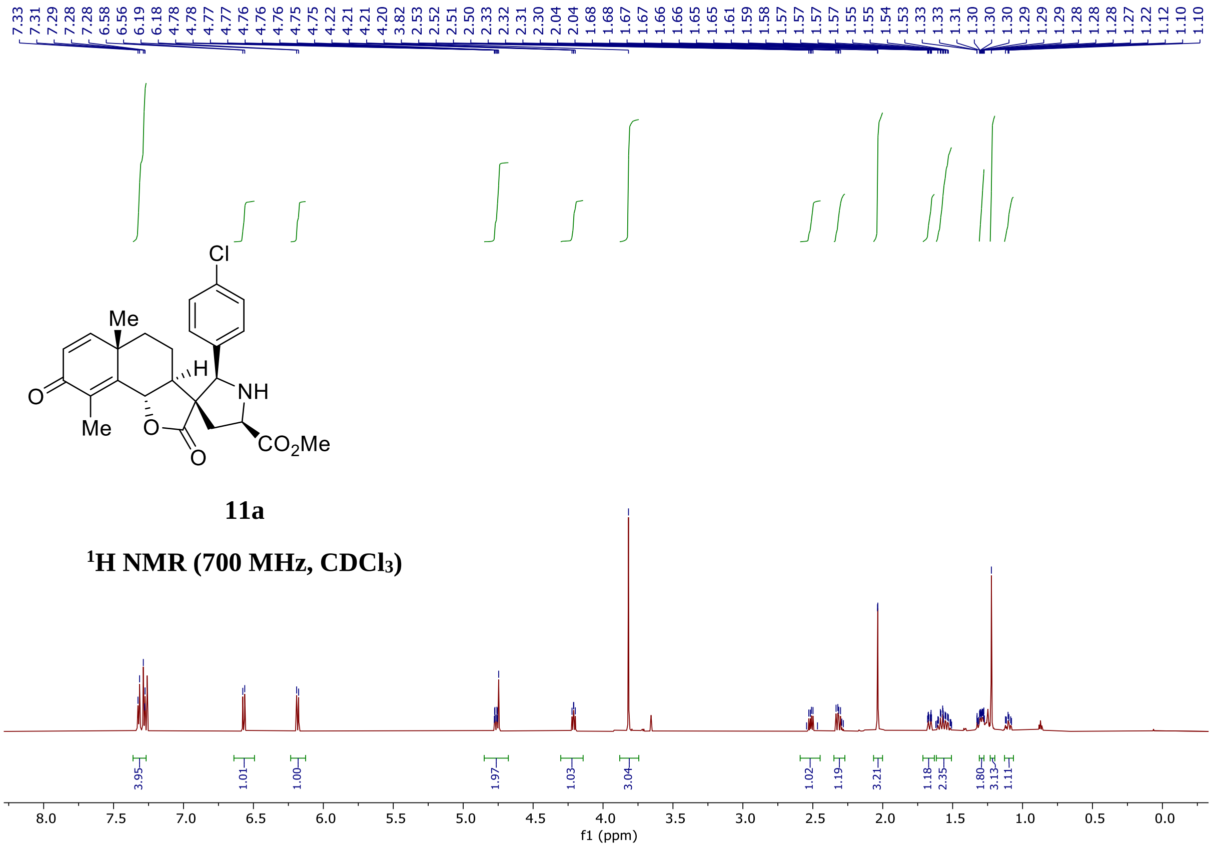

11a

^{1}H NMR (700 MHz, CDCl$_3$)

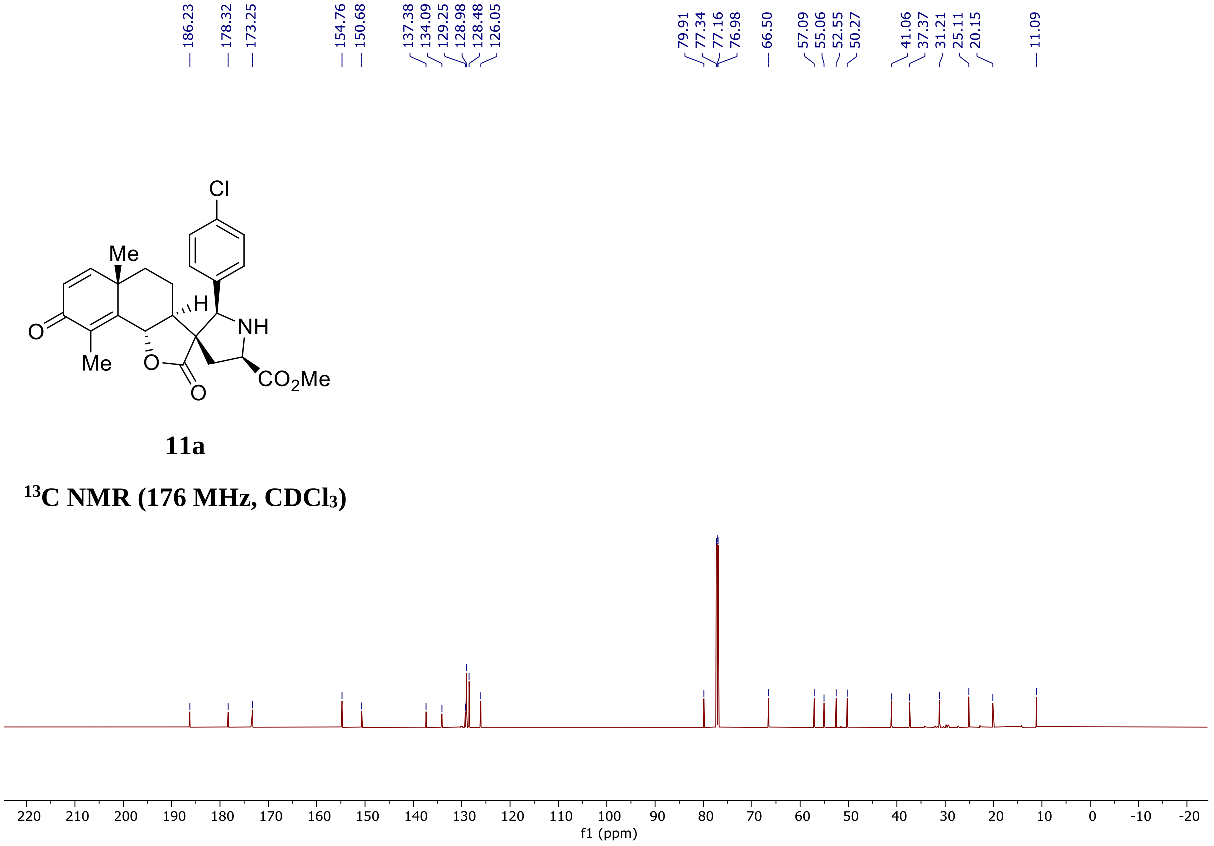

11a

^{13}C NMR (176 MHz, CDCl$_3$)

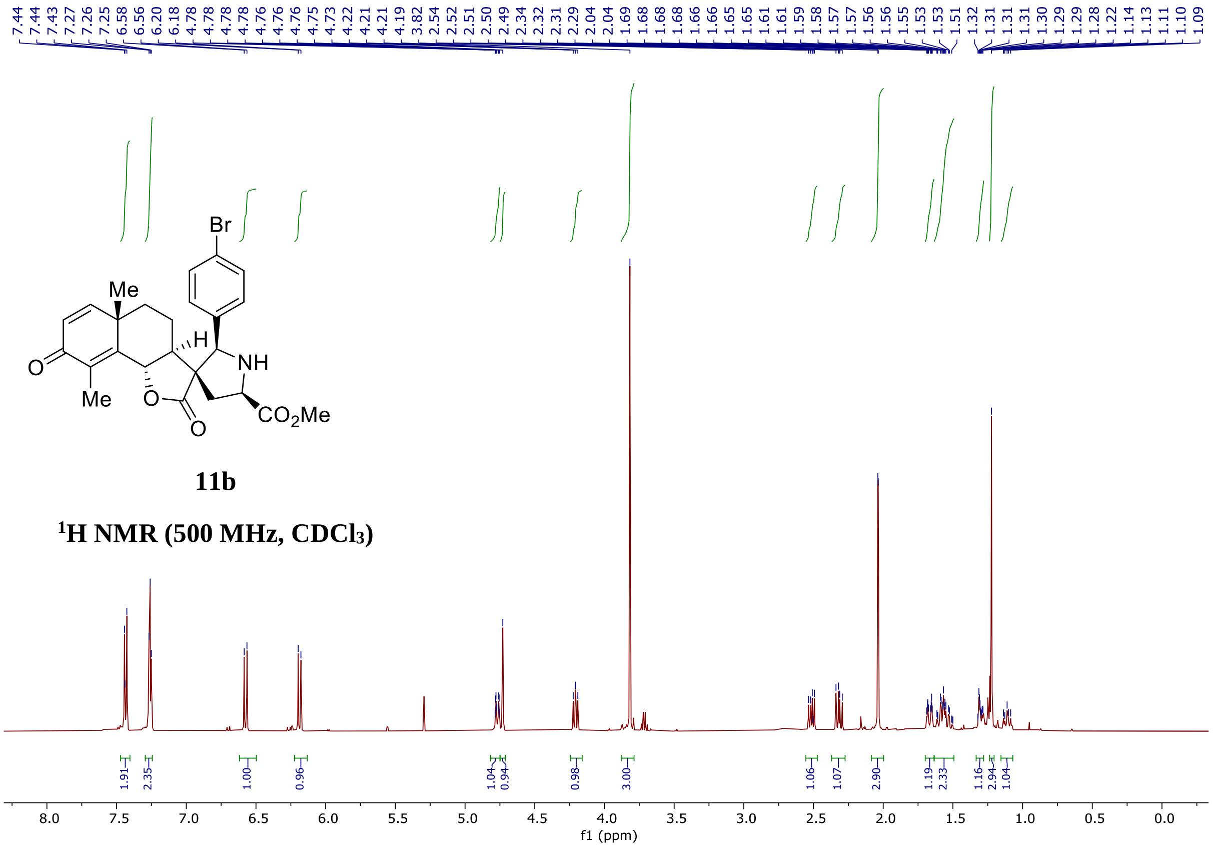

11b

^{1}H NMR (500 MHz, CDCl$_3$)

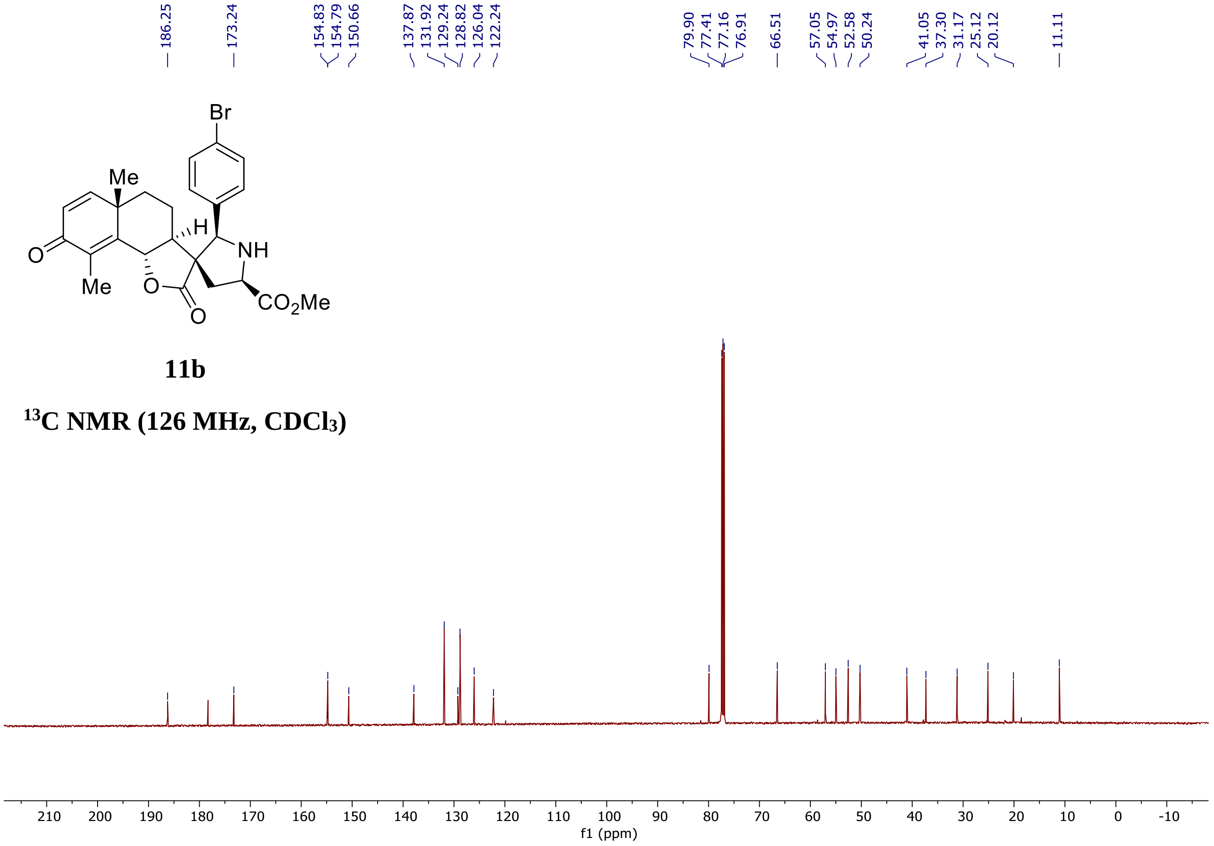

11b

^{13}C NMR (126 MHz, CDCl$_3$)

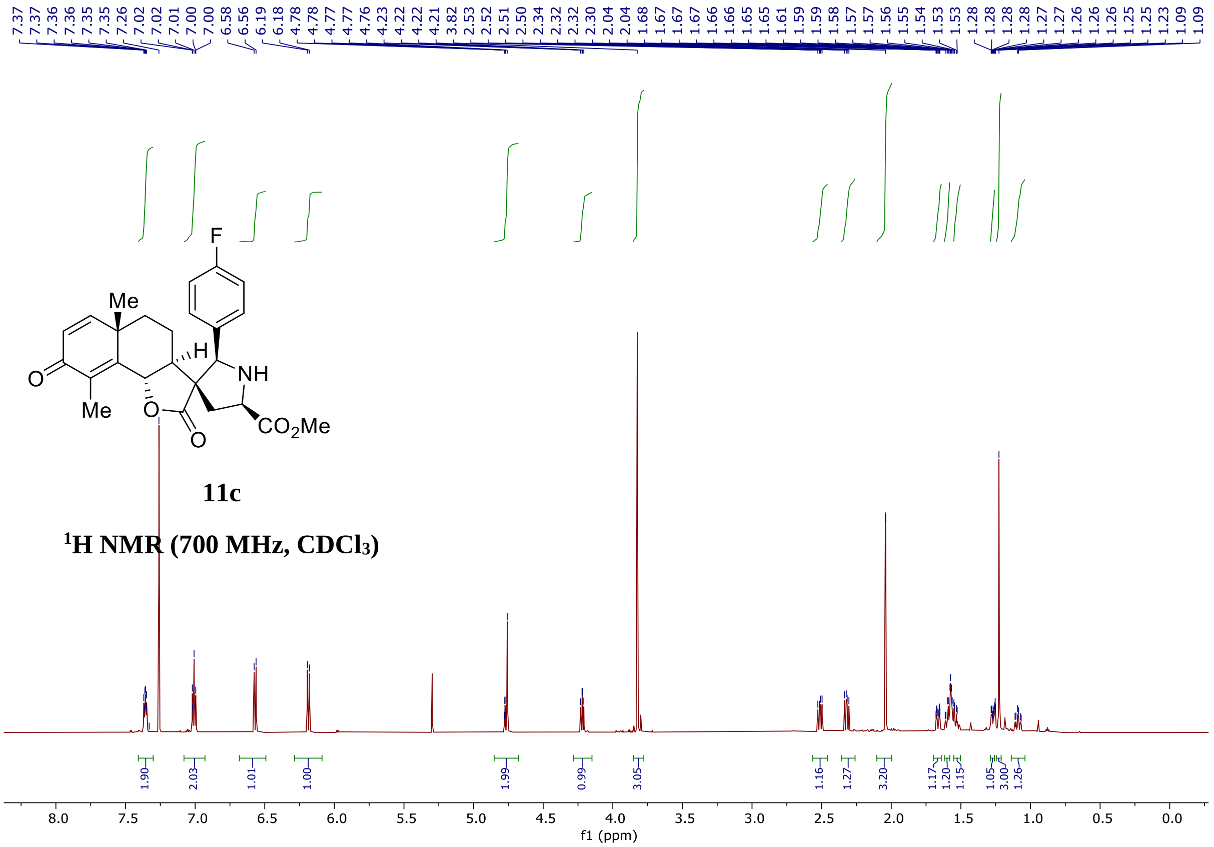

11c

^{1}H NMR (700 MHz, $CDCl_3$)

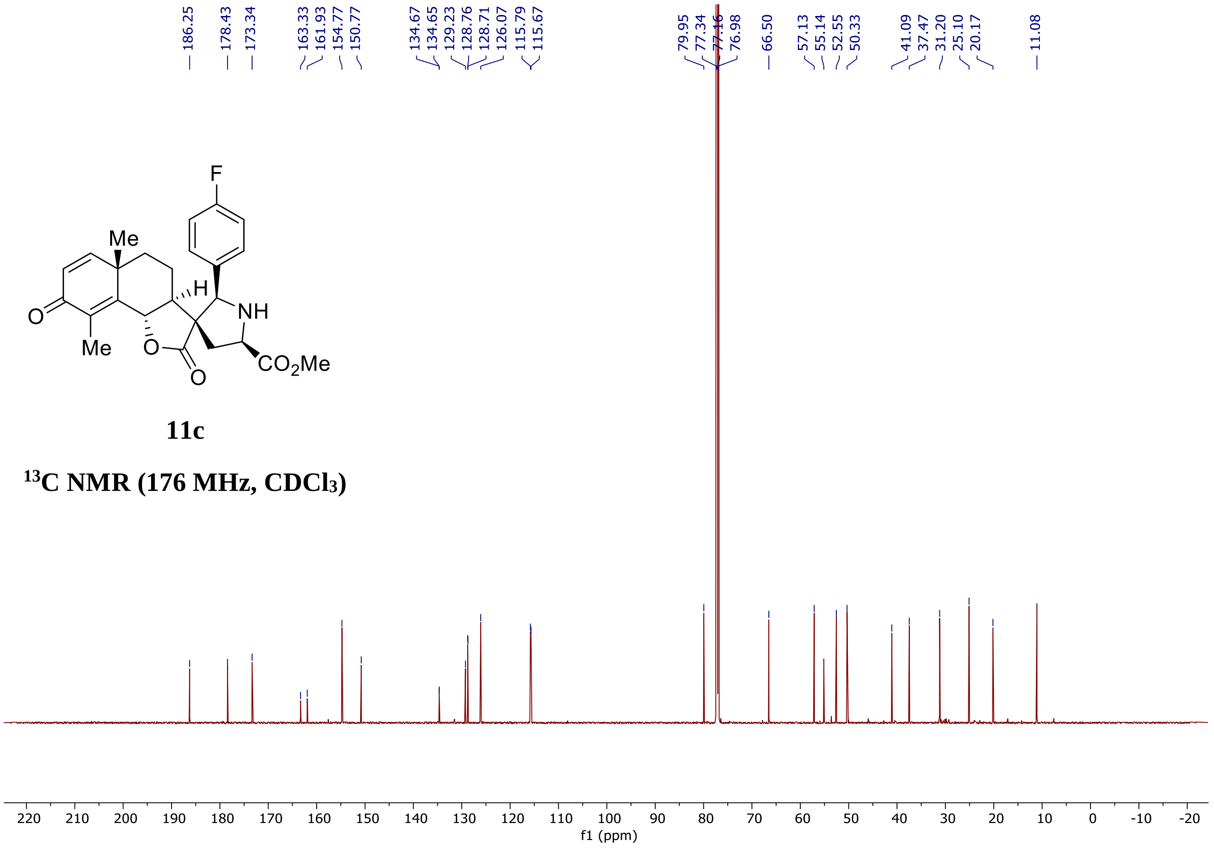

11c

^{13}C NMR (176 MHz, $CDCl_3$)

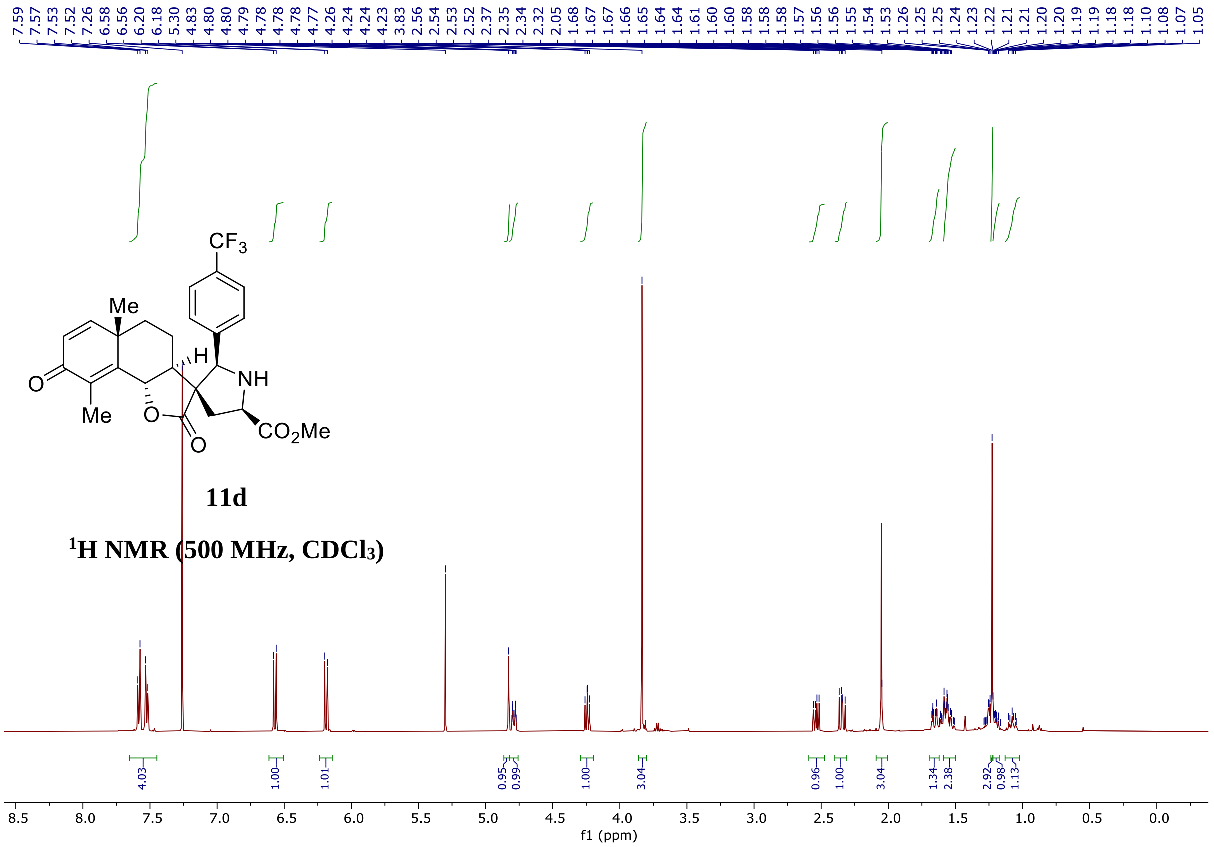

11d

^{1}H NMR (500 MHz, CDCl$_3$)

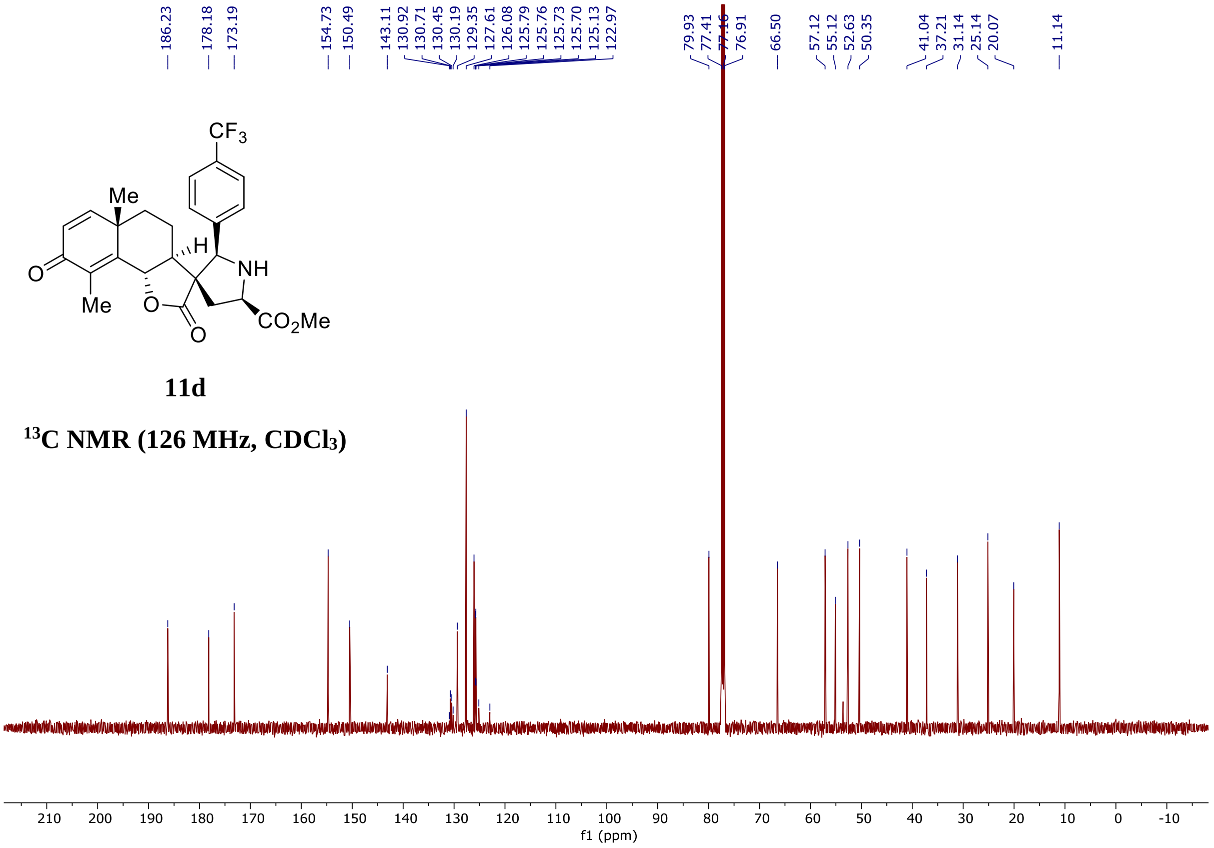

11d

^{13}C NMR (126 MHz, CDCl$_3$)

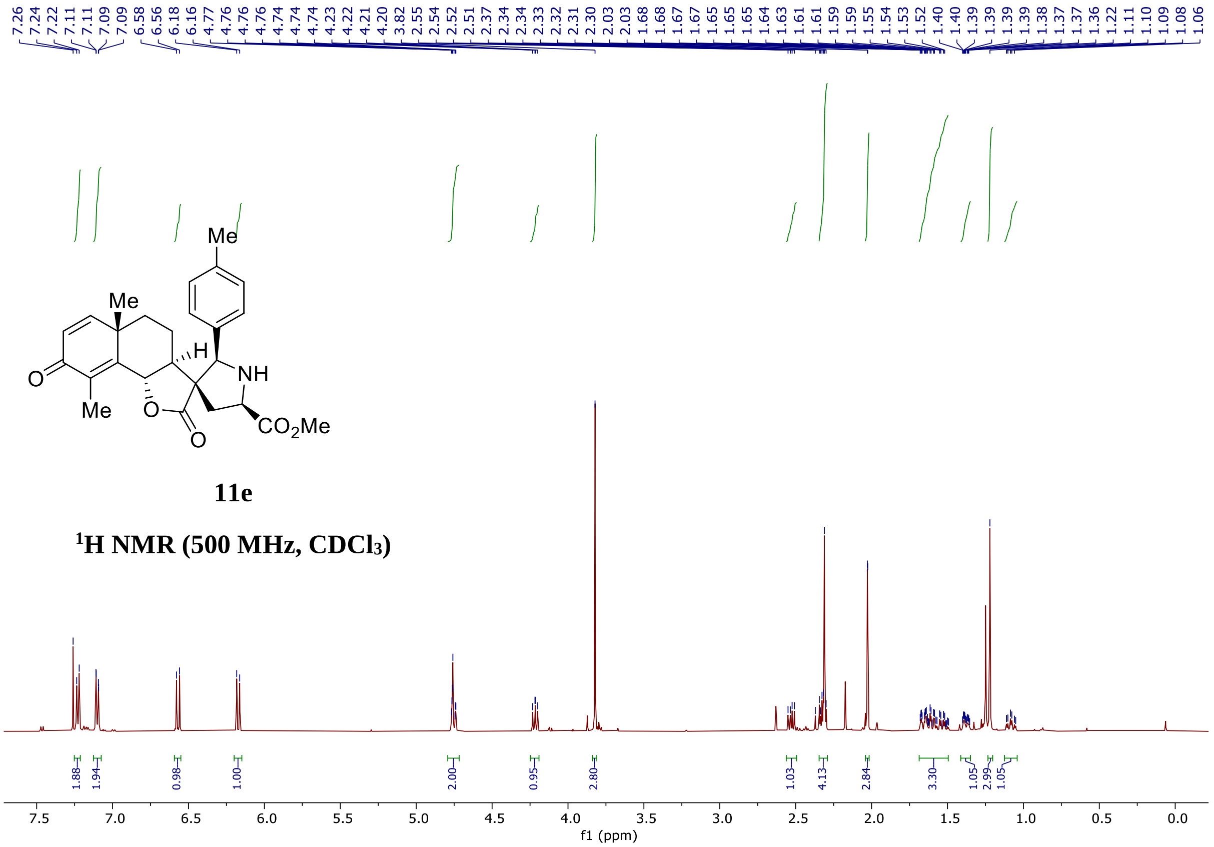

11e

^{1}H NMR (500 MHz, CDCl$_3$)

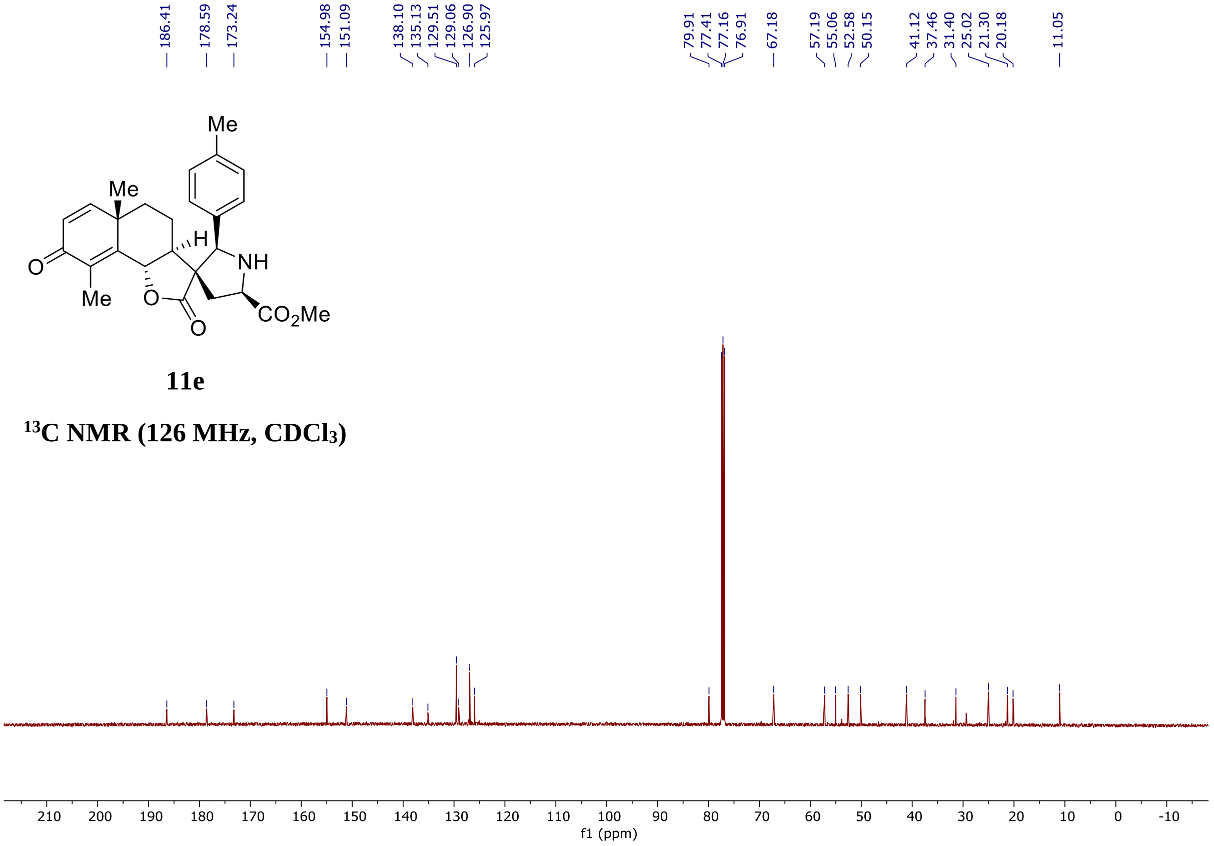

11e

^{13}C NMR (126 MHz, CDCl$_3$)

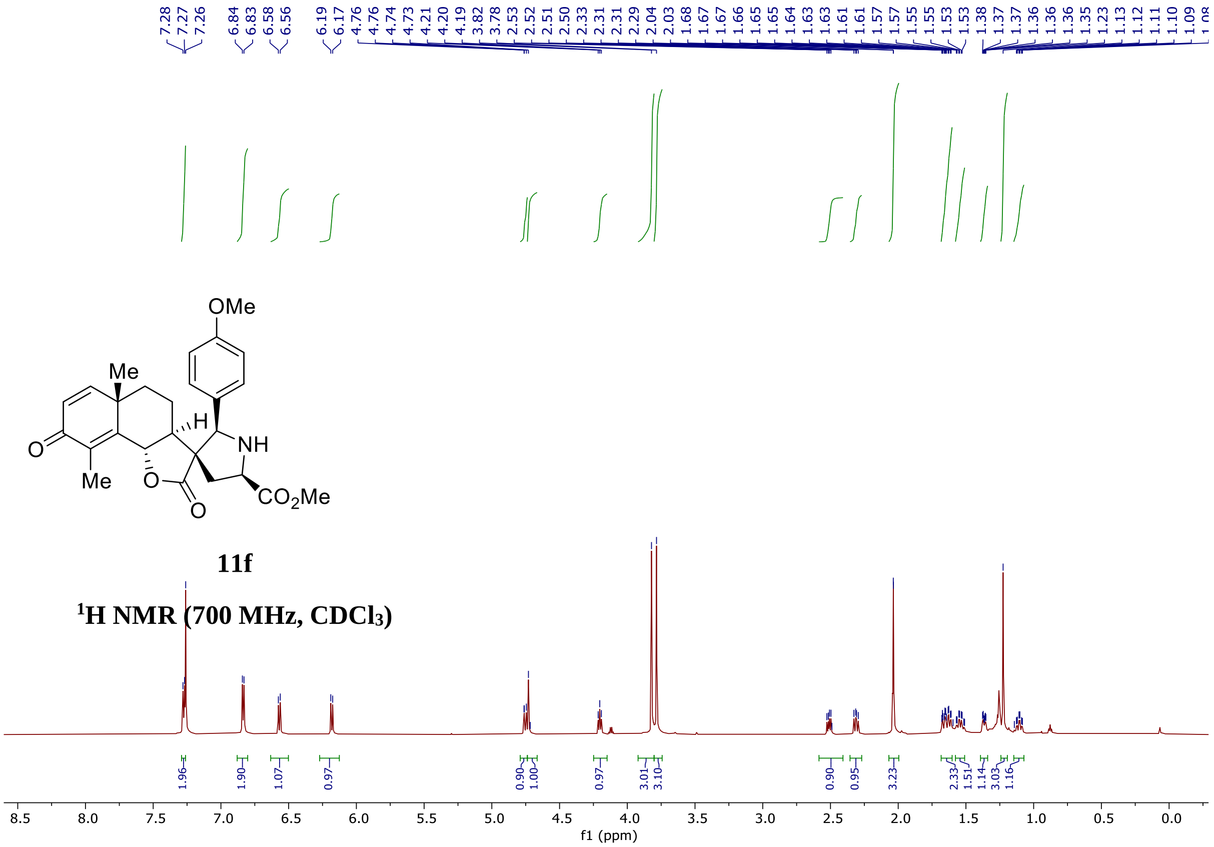

11f

^{1}H NMR (700 MHz, CDCl3)

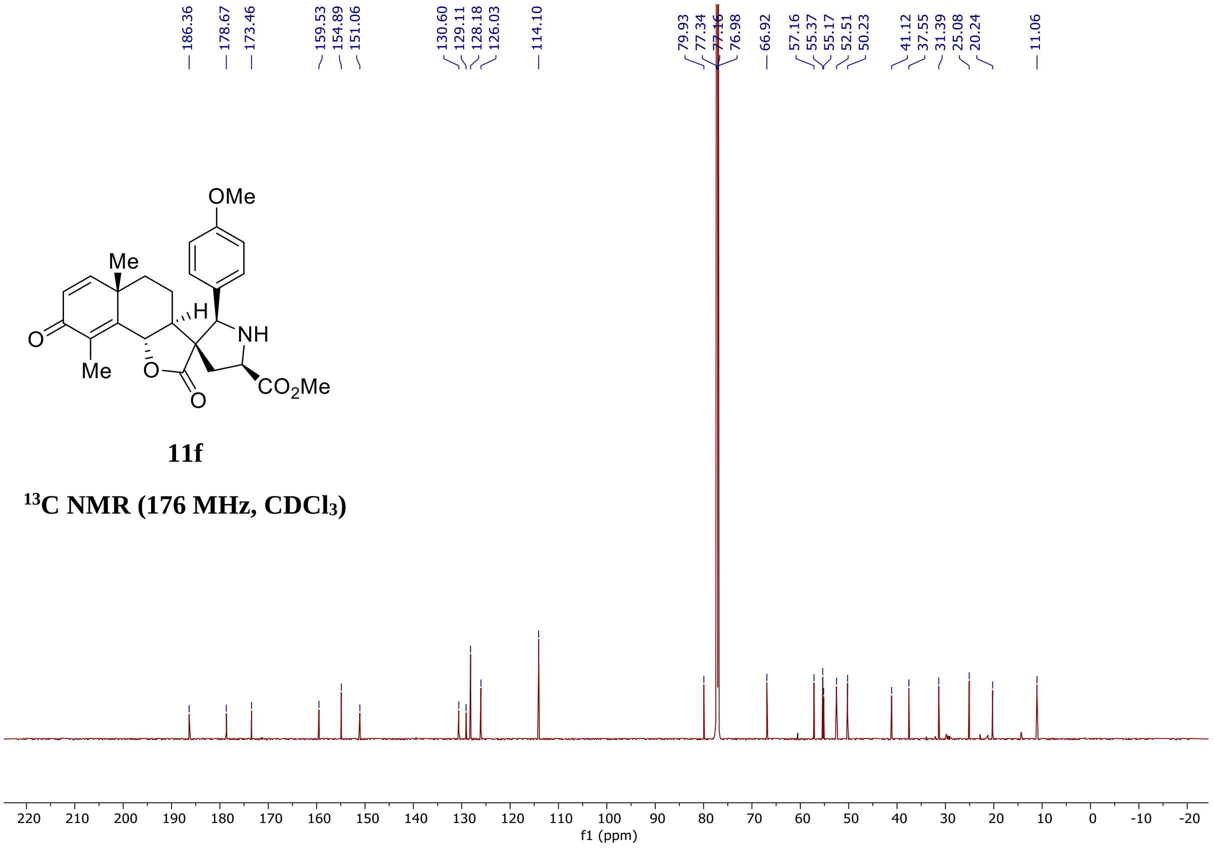

11f

^{13}C NMR (176 MHz, CDCl3)

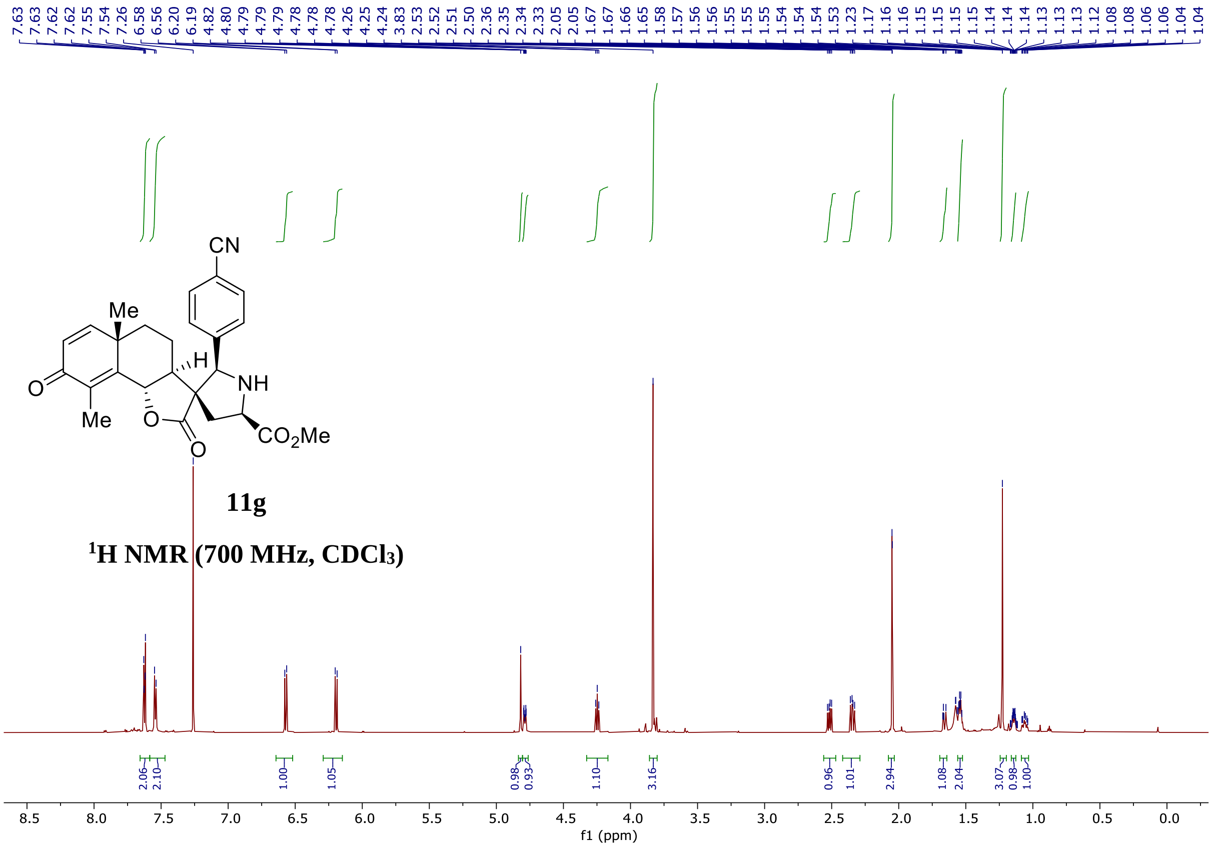
7.63 7.63 7.62 7.62 7.55 7.54 7.26 6.58 6.56 6.20 6.19 4.82 4.80 4.79 4.79 4.79 4.78 4.78 4.78 4.26 4.25 4.24 3.83 2.53 2.52 2.51 2.50 2.36 2.35 2.34 2.33 2.05 2.05 1.67 1.67 1.66 1.65 1.58 1.57 1.56 1.56 1.55 1.55 1.55 1.54 1.54 1.54 1.53 1.23 1.17 1.16 1.16 1.15 1.15 1.15 1.15 1.14 1.14 1.14 1.13 1.13 1.13 1.12 1.08 1.08 1.06 1.06 1.04 1.04
CN
Me
H
NH
O
Me
O
O
CO2Me
11g
1H NMR (700 MHz, CDCl3)
2.06 2.10 1.00 1.05 0.98 0.93 1.10 3.16 0.96 1.01 2.94 1.08 2.04 3.07 0.98 1.00
8.5 8.0 7.5 7.0 6.5 6.0 5.5 5.0 4.5 4.0 3.5 3.0 2.5 2.0 1.5 1.0 0.5 0.0
f1 (ppm)

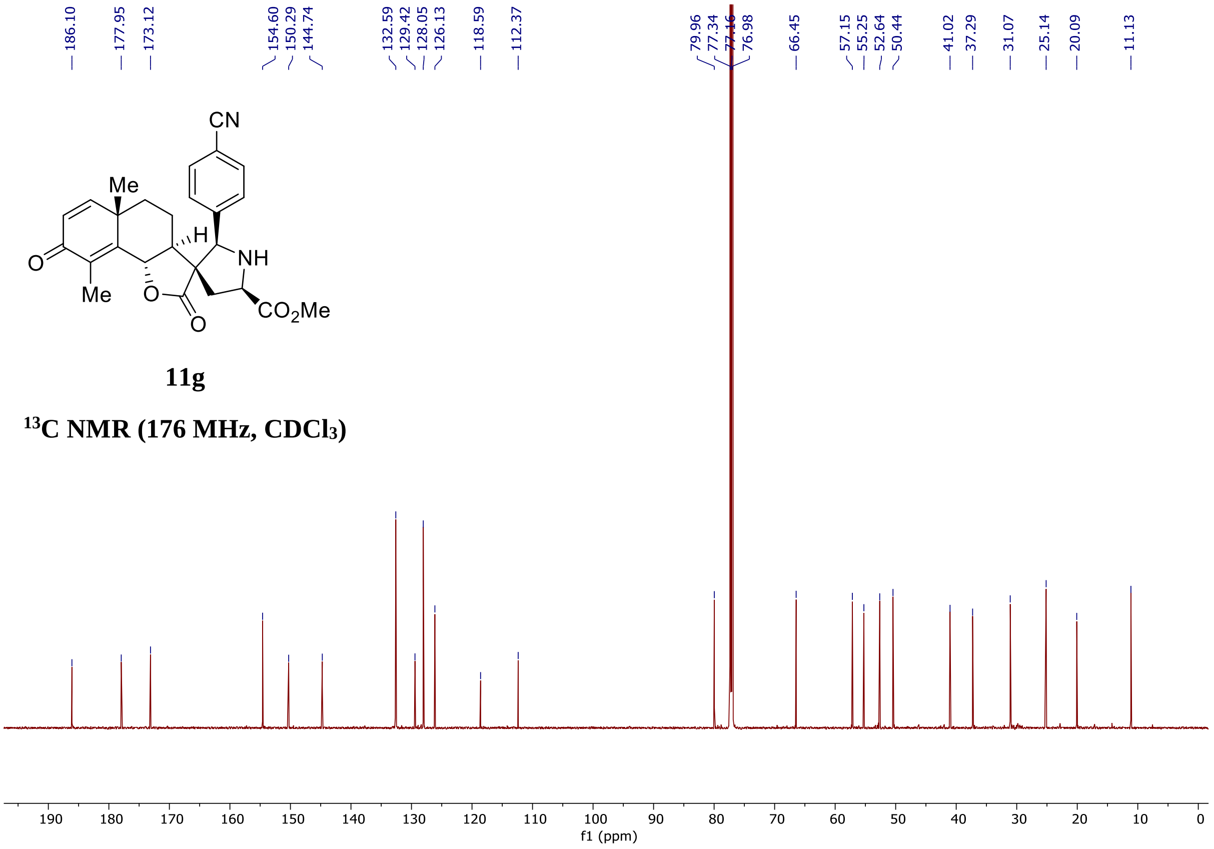
186.10 177.95 173.12 154.60 150.29 144.74 132.59 129.42 128.05 126.13 118.59 112.37 79.96 77.34 77.16 76.98 66.45 57.15 55.25 52.64 50.44 41.02 37.29 31.07 25.14 20.09 11.13
CN
Me
H
NH
O
Me
O
O
CO2Me
11g
13C NMR (176 MHz, CDCl3)
190 180 170 160 150 140 130 120 110 100 90 80 70 60 50 40 30 20 10 0
f1 (ppm)

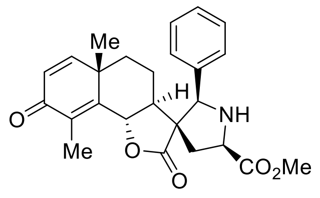

11h

^{1}H NMR (700 MHz, CDCl$_3$)

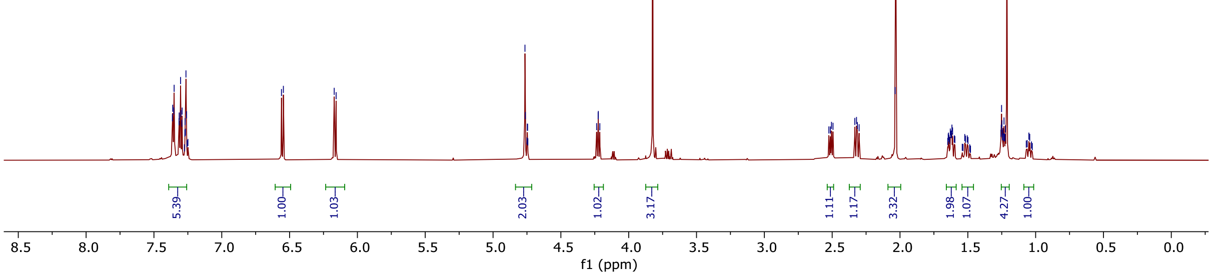

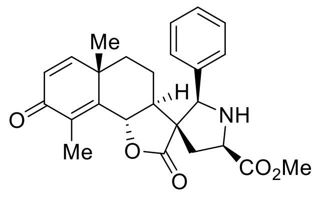

11h

^{13}C NMR (176 MHz, CDCl$_3$)

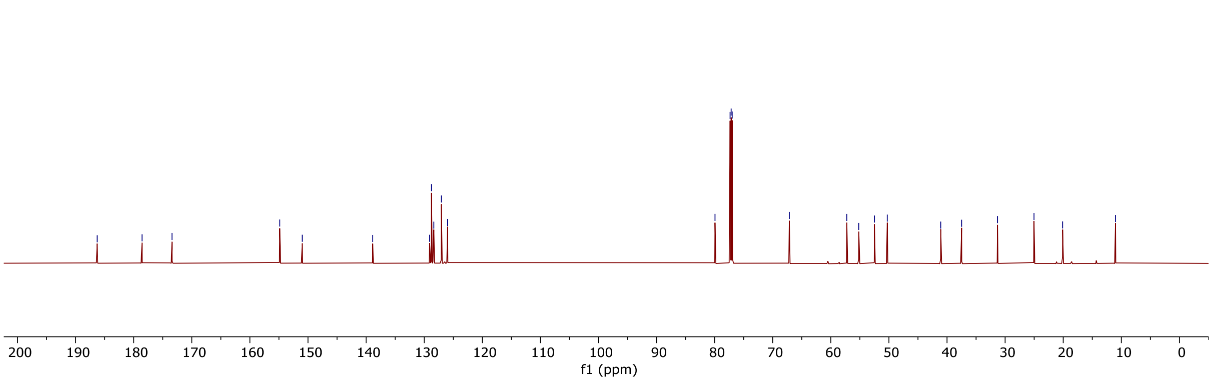

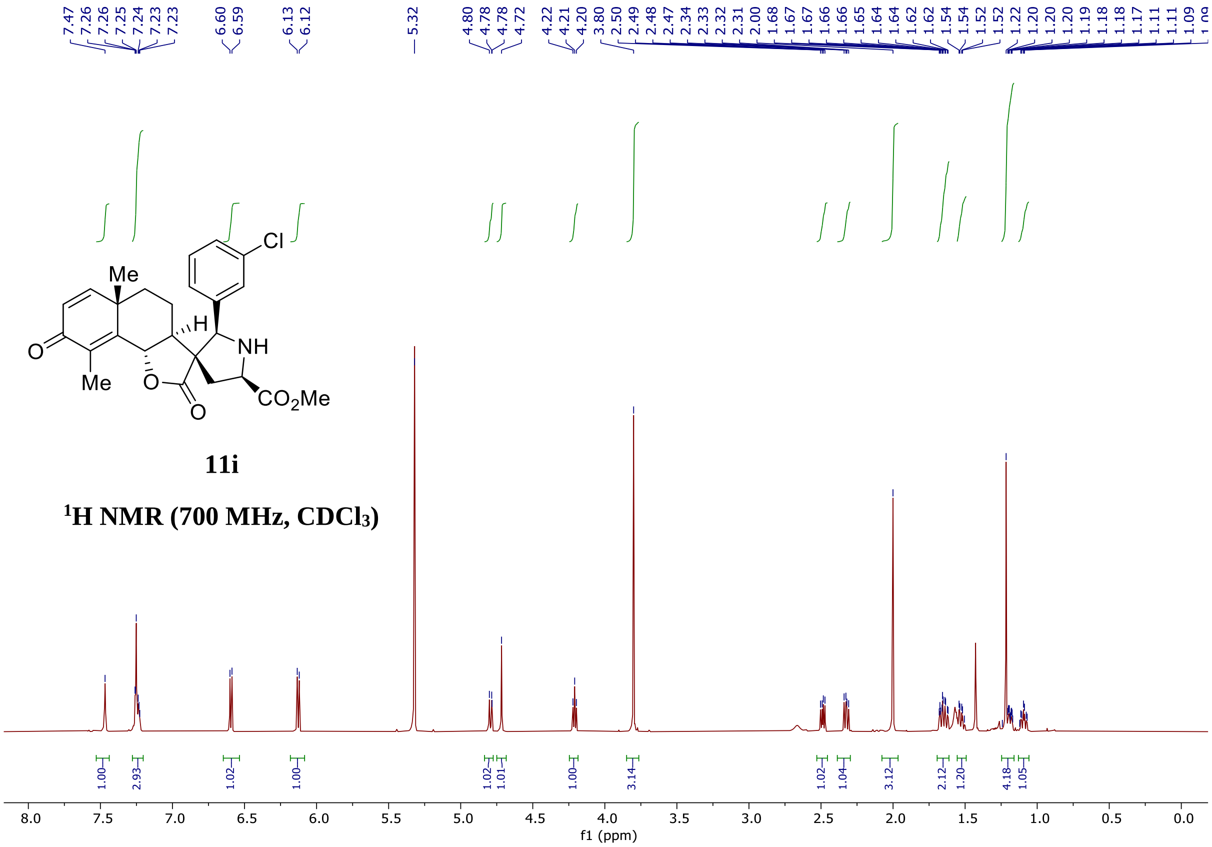

11i

^{1}H NMR (700 MHz, $CDCl_3$)

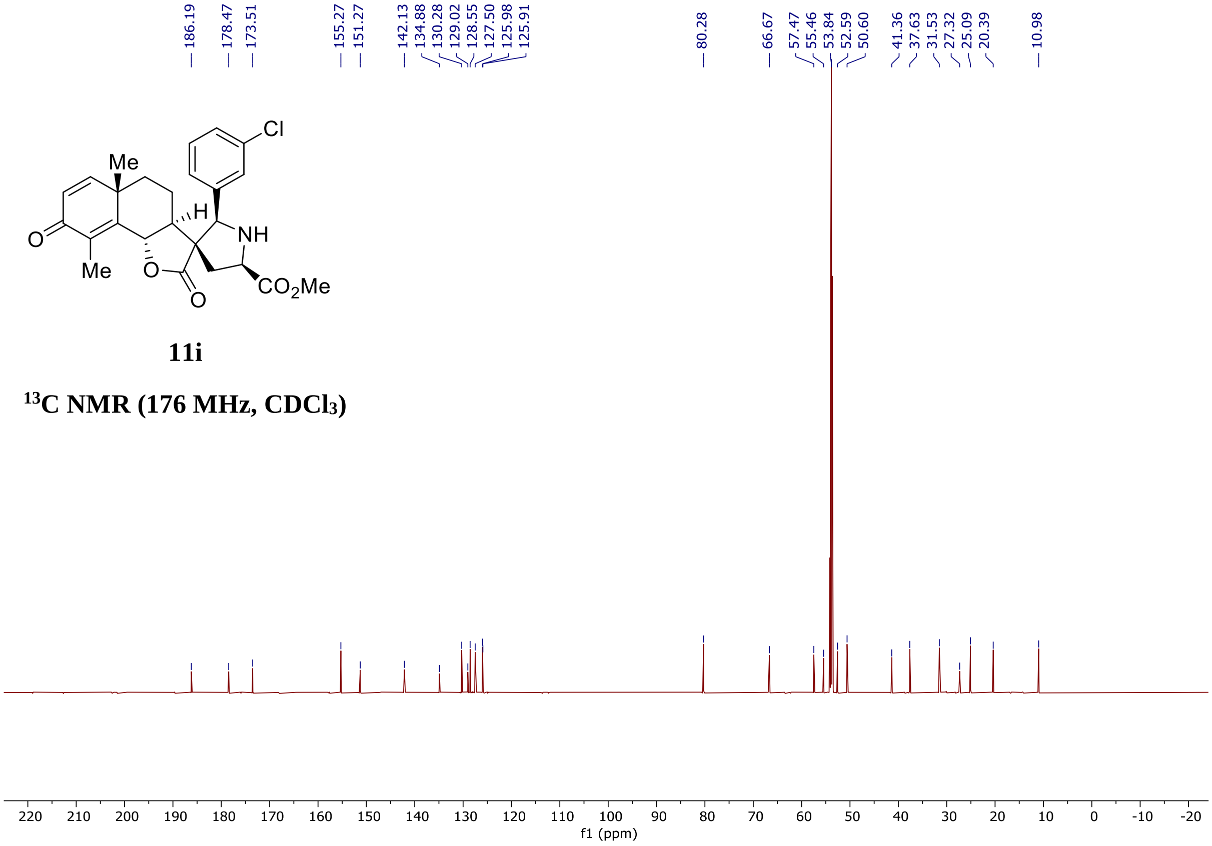

11i

^{13}C NMR (176 MHz, $CDCl_3$)

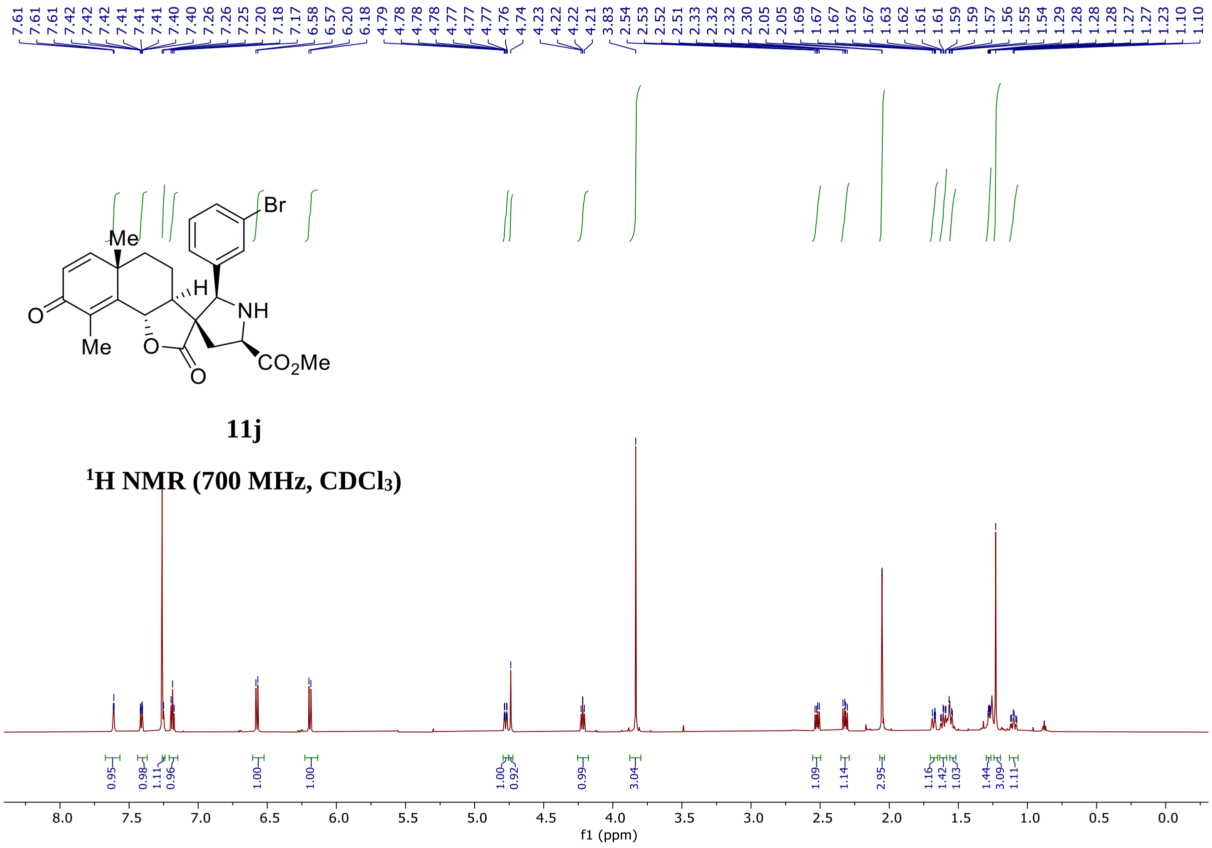
7.61 7.61 7.42 7.42 7.41 7.41 7.41 7.40 7.40 7.26 7.25 7.20 7.18 7.17 6.58 6.57 6.20 6.18 4.79 4.78 4.78 4.77 4.77 4.76 4.74 4.23 4.22 4.22 4.21 3.83 2.54 2.53 2.52 2.51 2.33 2.32 2.32 2.30 2.05 2.05 1.69 1.67 1.67 1.67 1.63 1.62 1.61 1.61 1.59 1.59 1.57 1.56 1.55 1.54 1.29 1.28 1.28 1.28 1.27 1.27 1.23 1.10 1.10
Br
Me
H
NH
O
Me
O
O
CO2Me
11j
1H NMR (700 MHz, CDCl3)
0.95 0.98 1.11 0.96 1.00 1.00 1.00 0.92 0.99 3.04 1.09 1.14 2.95 1.16 1.42 1.03 1.44 3.09 1.11
8.0 7.5 7.0 6.5 6.0 5.5 5.0 4.5 4.0 3.5 3.0 2.5 2.0 1.5 1.0 0.5 0.0
f1 (ppm)

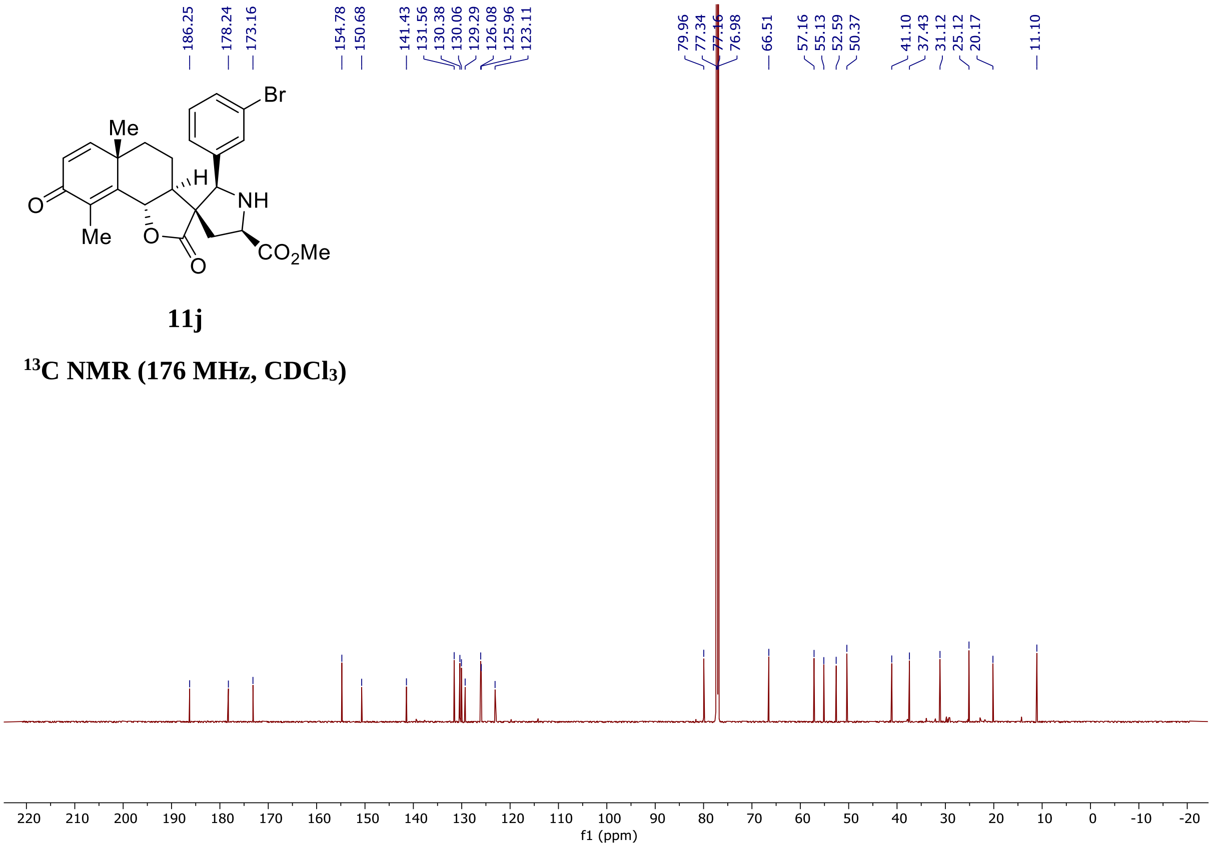
186.25 178.24 173.16 154.78 150.68 141.43 131.56 130.38 130.06 129.29 126.08 125.96 123.11 79.96 77.34 77.16 76.98 66.51 57.16 55.13 52.59 50.37 41.10 37.43 31.12 25.12 20.17 11.10
Br
Me
H
NH
O
Me
O
O
CO2Me
11j
13C NMR (176 MHz, CDCl3)
220 210 200 190 180 170 160 150 140 130 120 110 100 90 80 70 60 50 40 30 20 10 0 -10 -20
f1 (ppm)

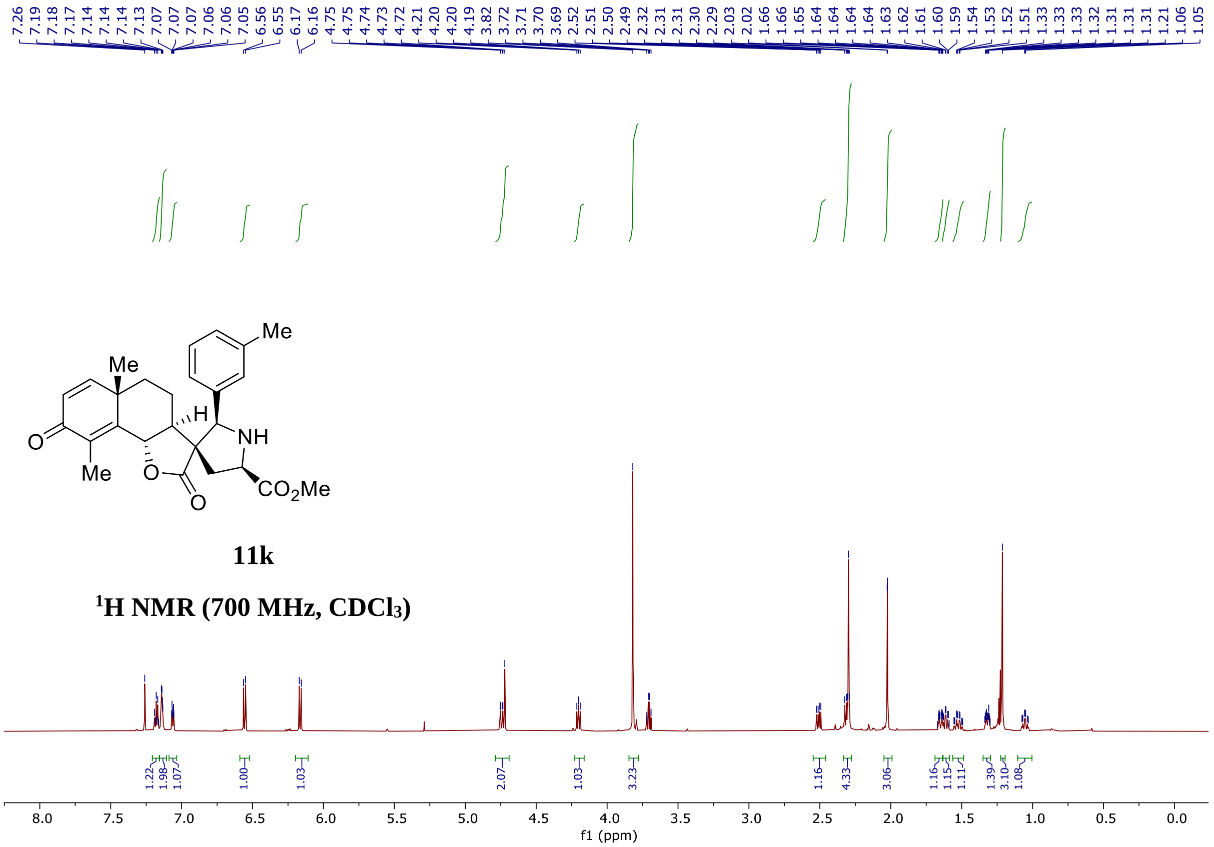

11k

^{1}H NMR (700 MHz, $CDCl_3$)

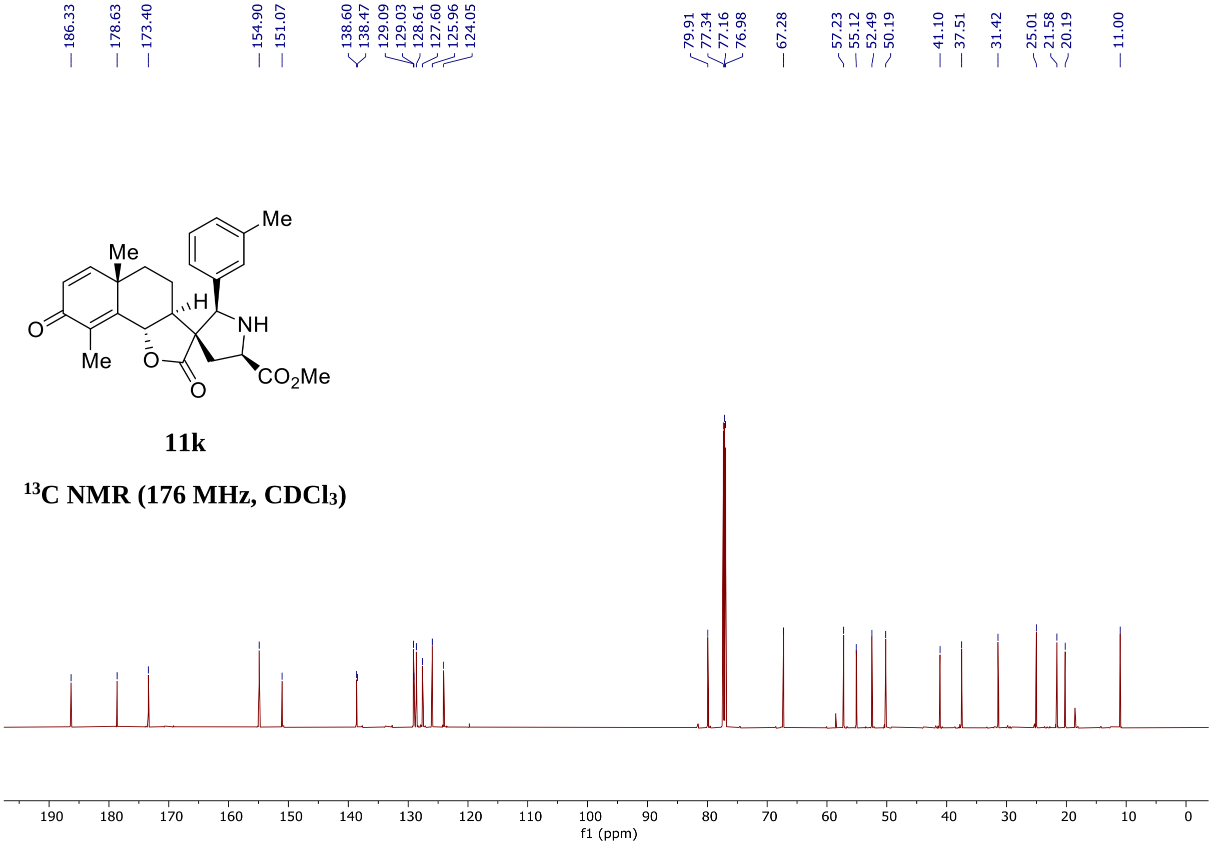

11k

^{13}C NMR (176 MHz, $CDCl_3$)

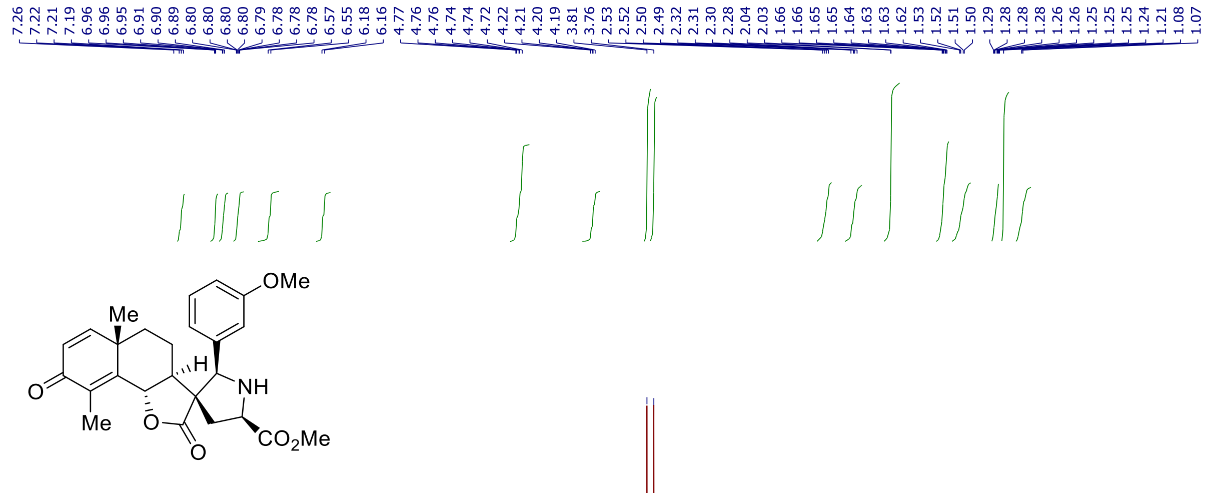

11l

^{1}H NMR (500 MHz, $CDCl_3$)

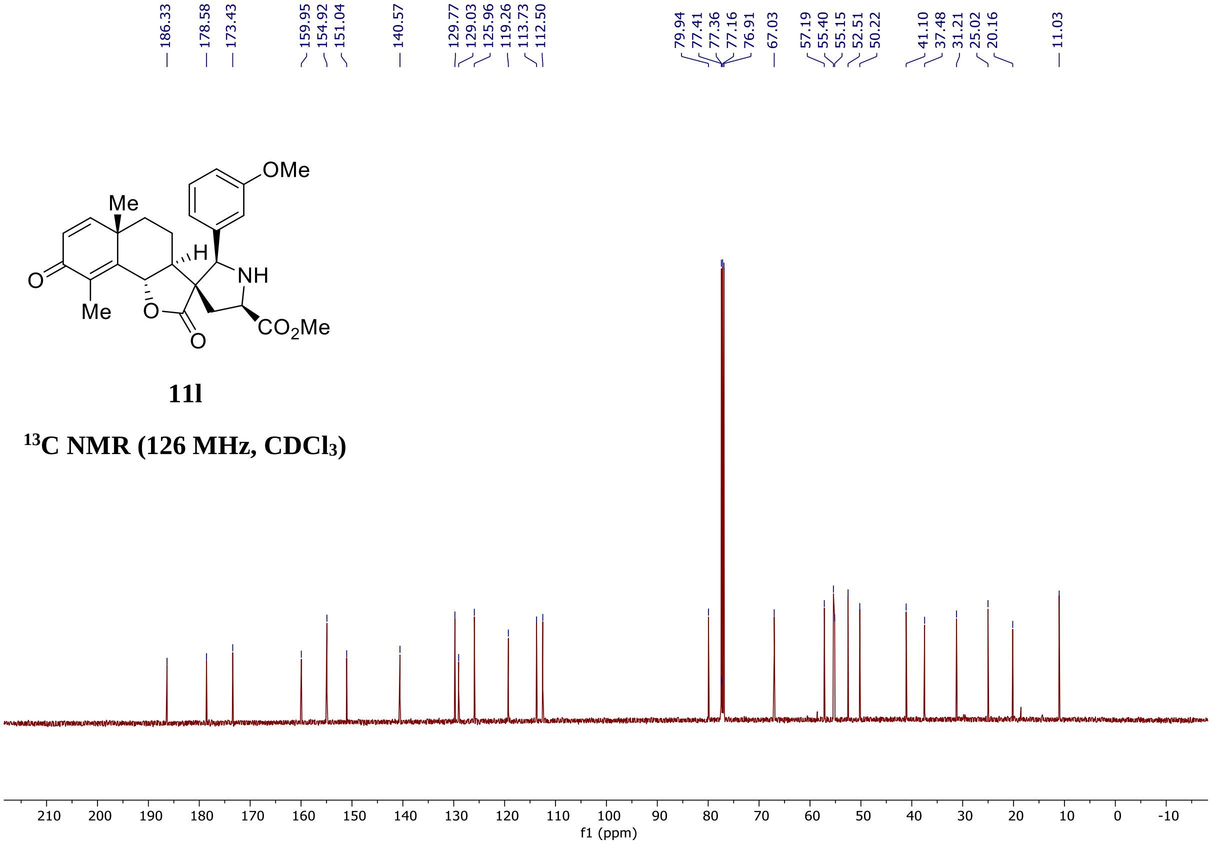

11l

^{13}C NMR (126 MHz, $CDCl_3$)

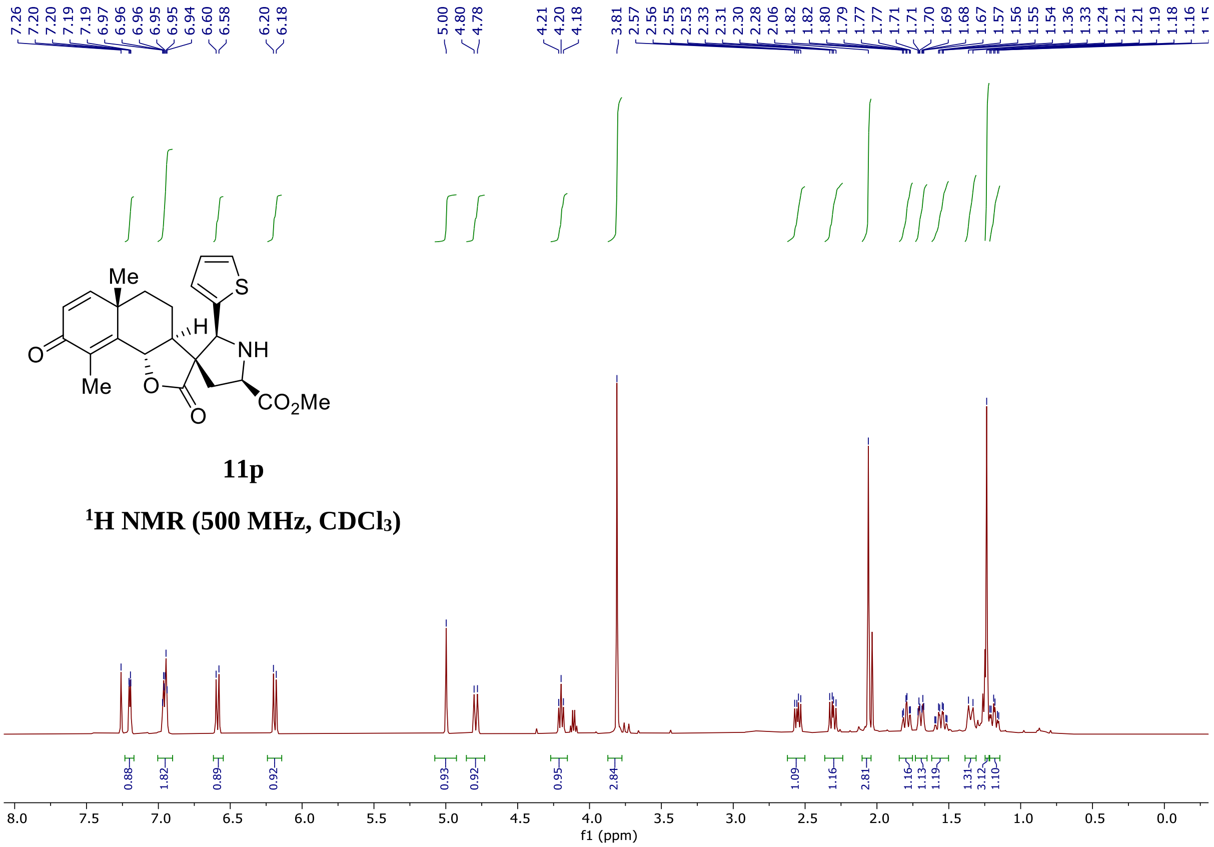

11p

^{1}H NMR (500 MHz, $CDCl_3$)

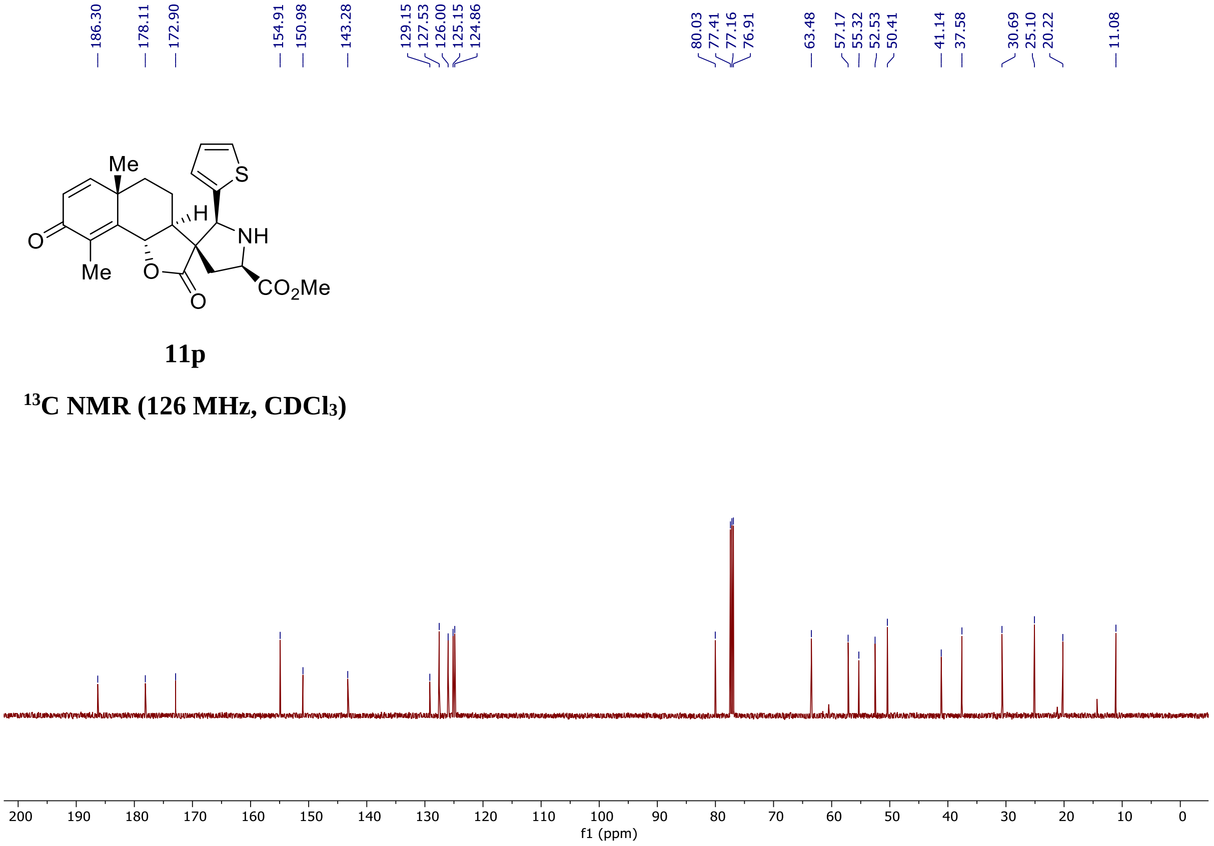

11p

^{13}C NMR (126 MHz, $CDCl_3$)

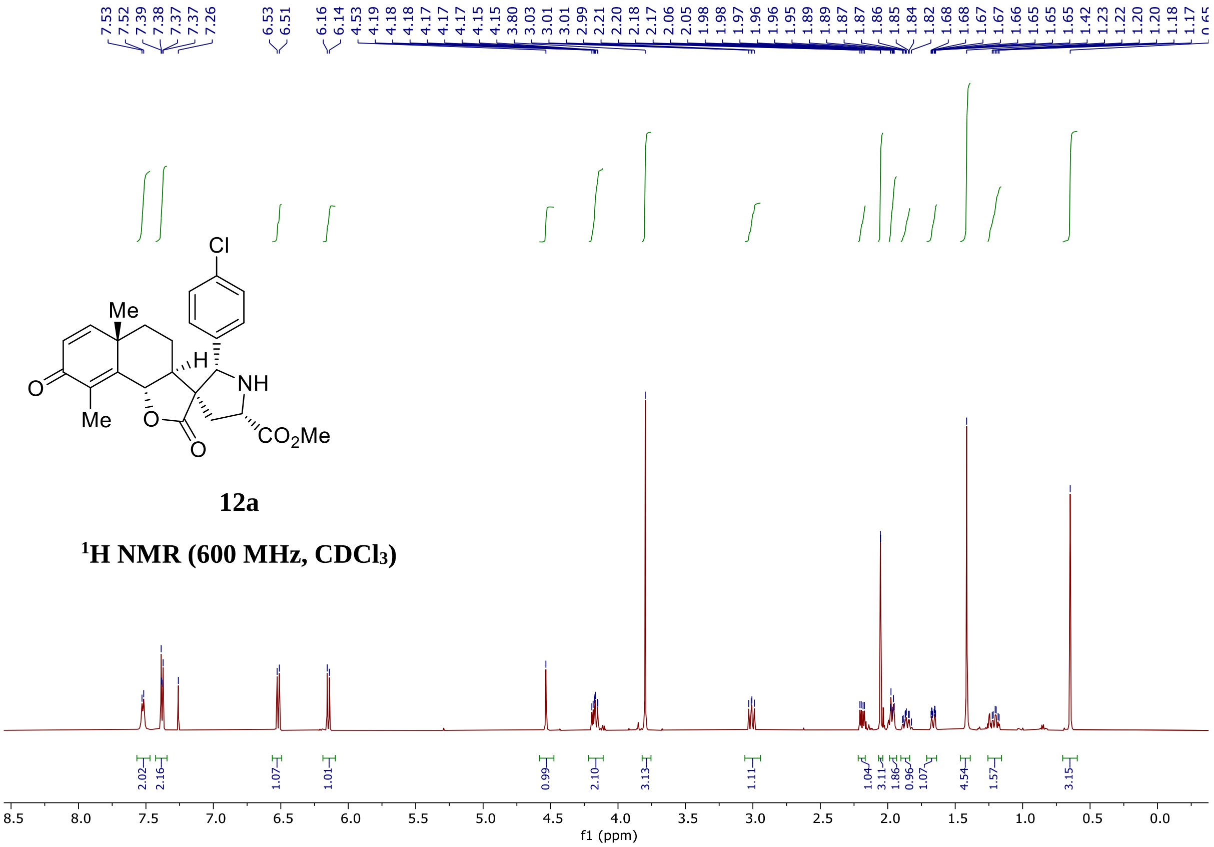

12a

^{1}H NMR (600 MHz, CDCl3)

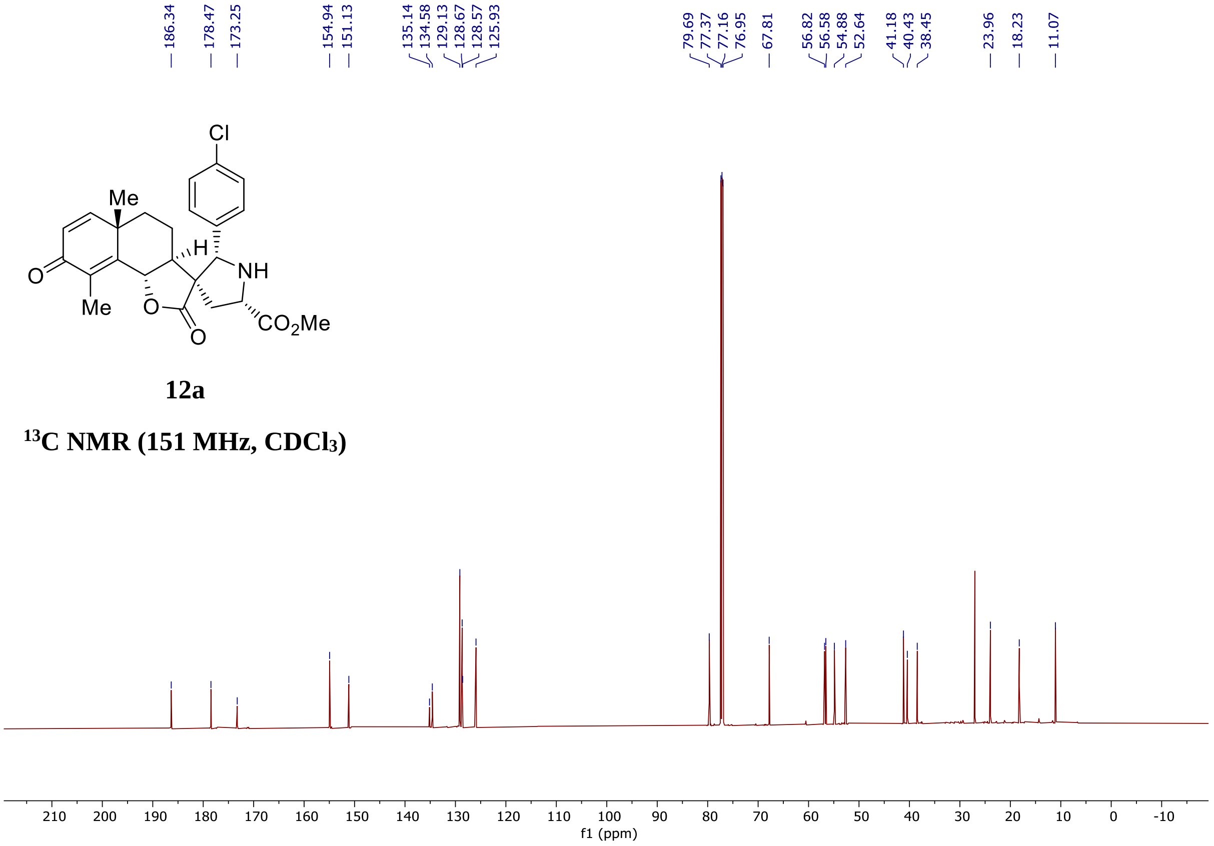

12a

^{13}C NMR (151 MHz, CDCl3)

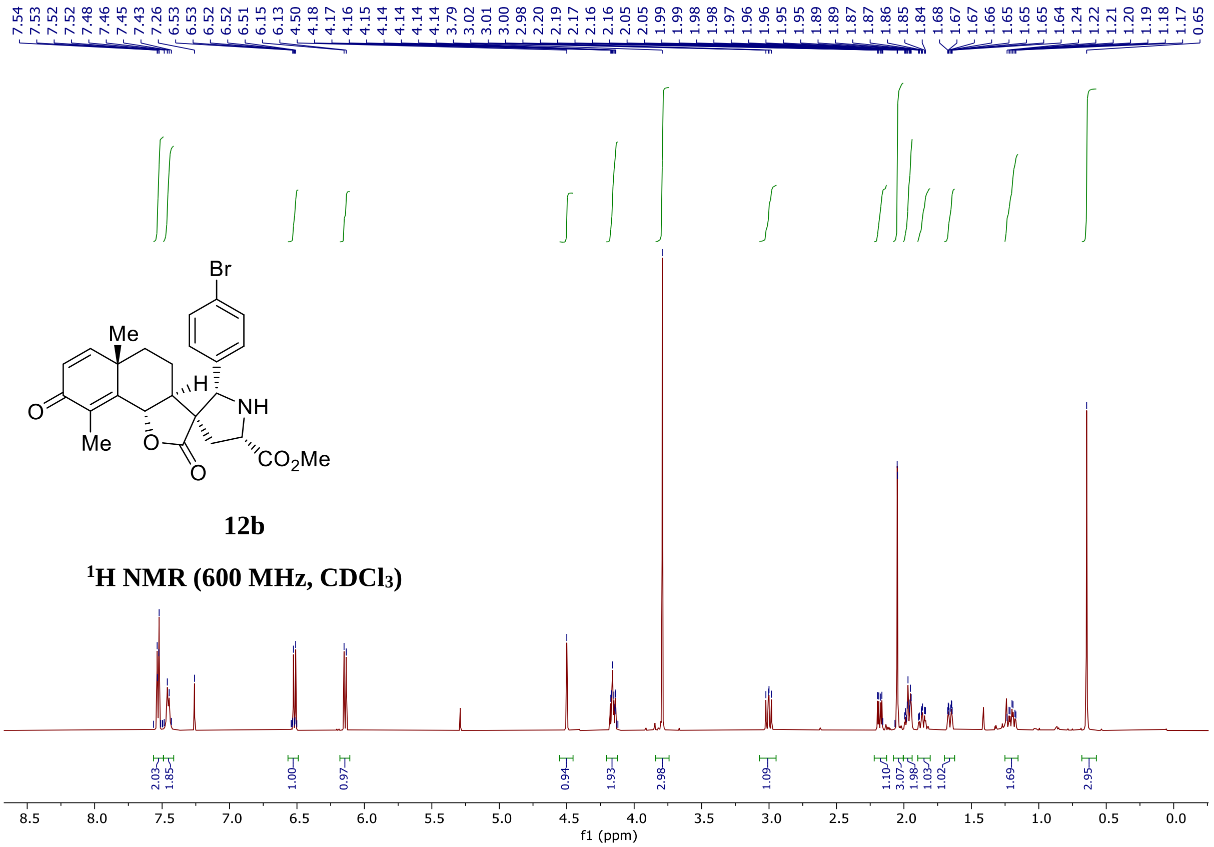

12b

^{1}H NMR (600 MHz, $CDCl_3$)

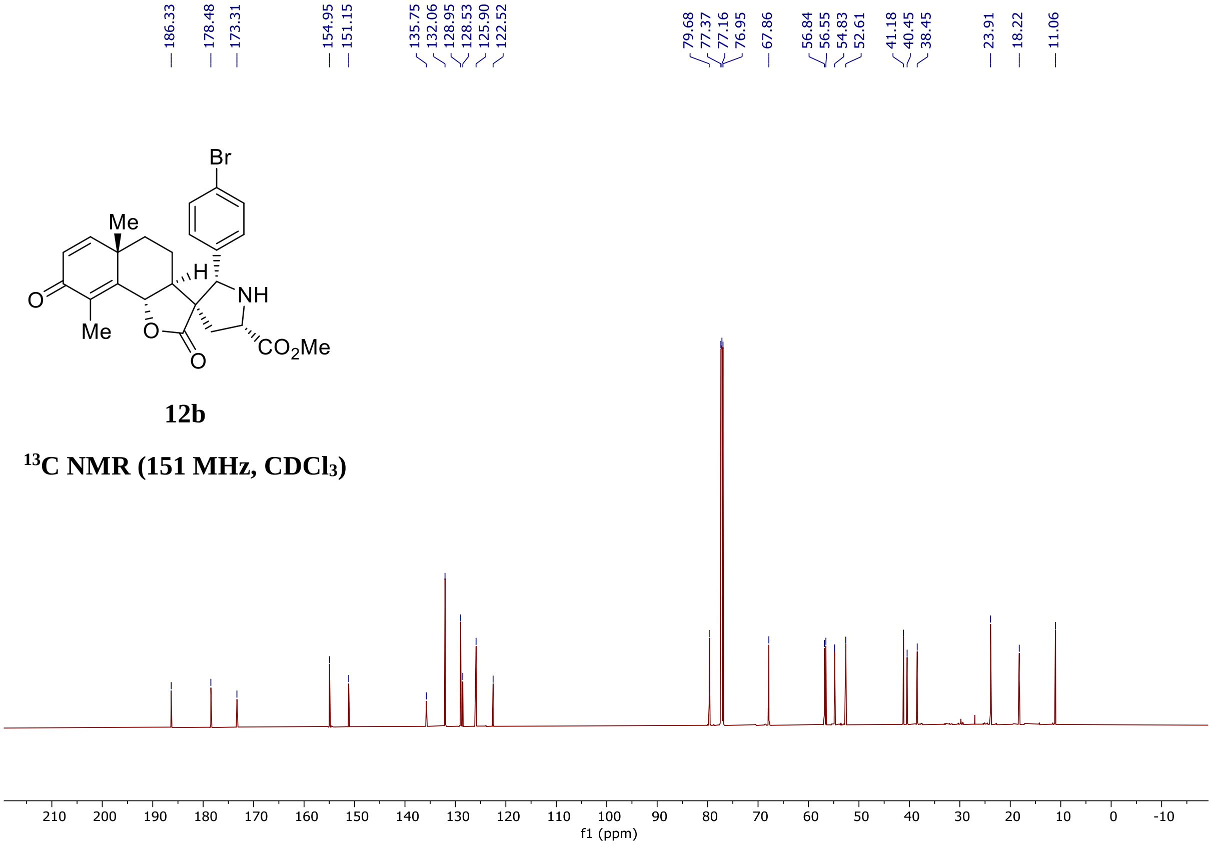

12b

^{13}C NMR (151 MHz, $CDCl_3$)

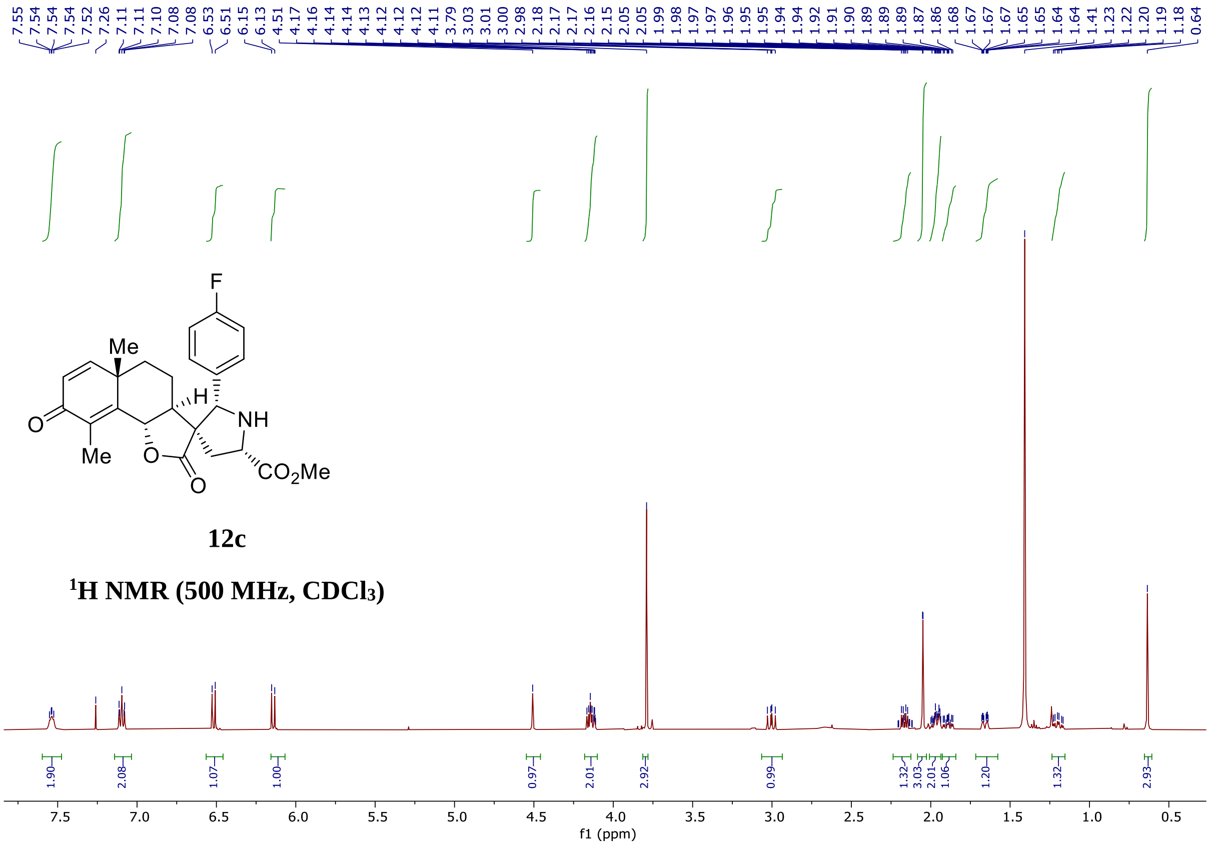

12c

^{1}H NMR (500 MHz, CDCl3)

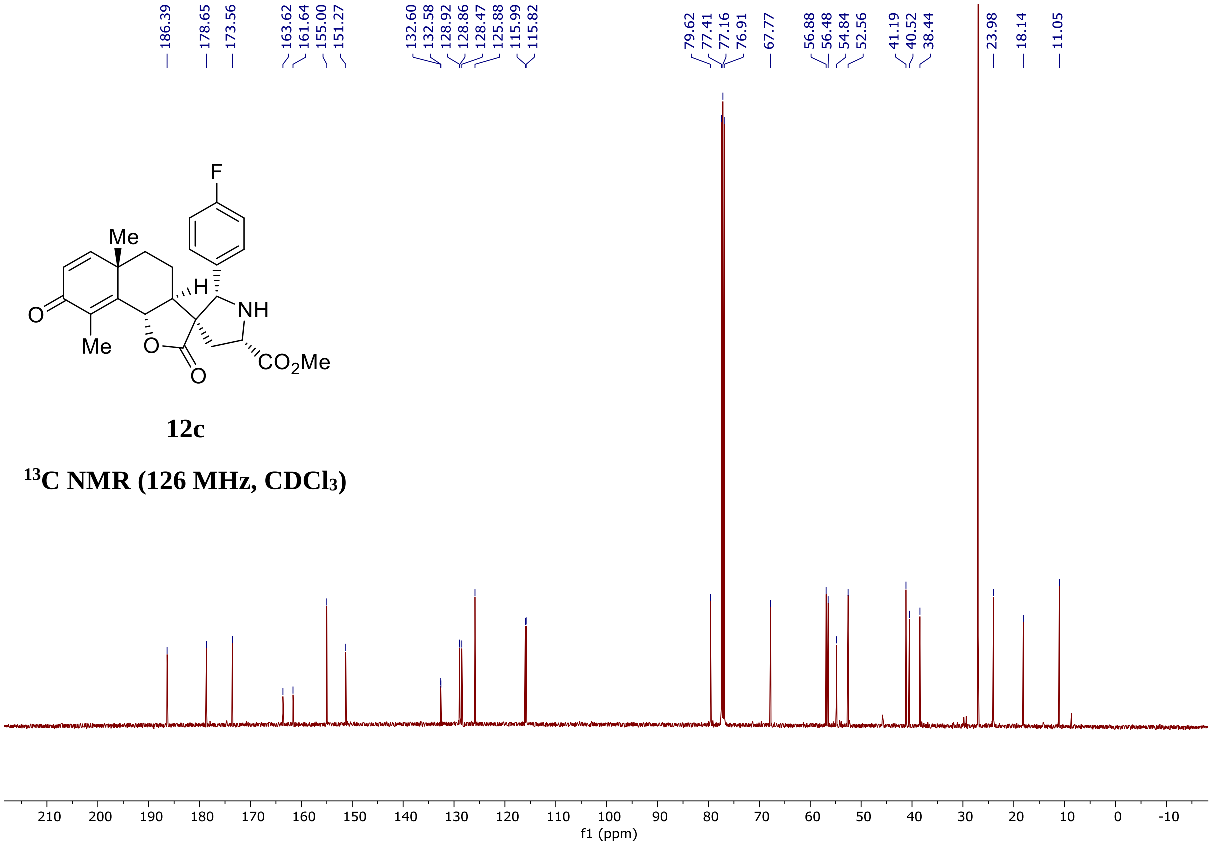

12c

^{13}C NMR (126 MHz, CDCl3)

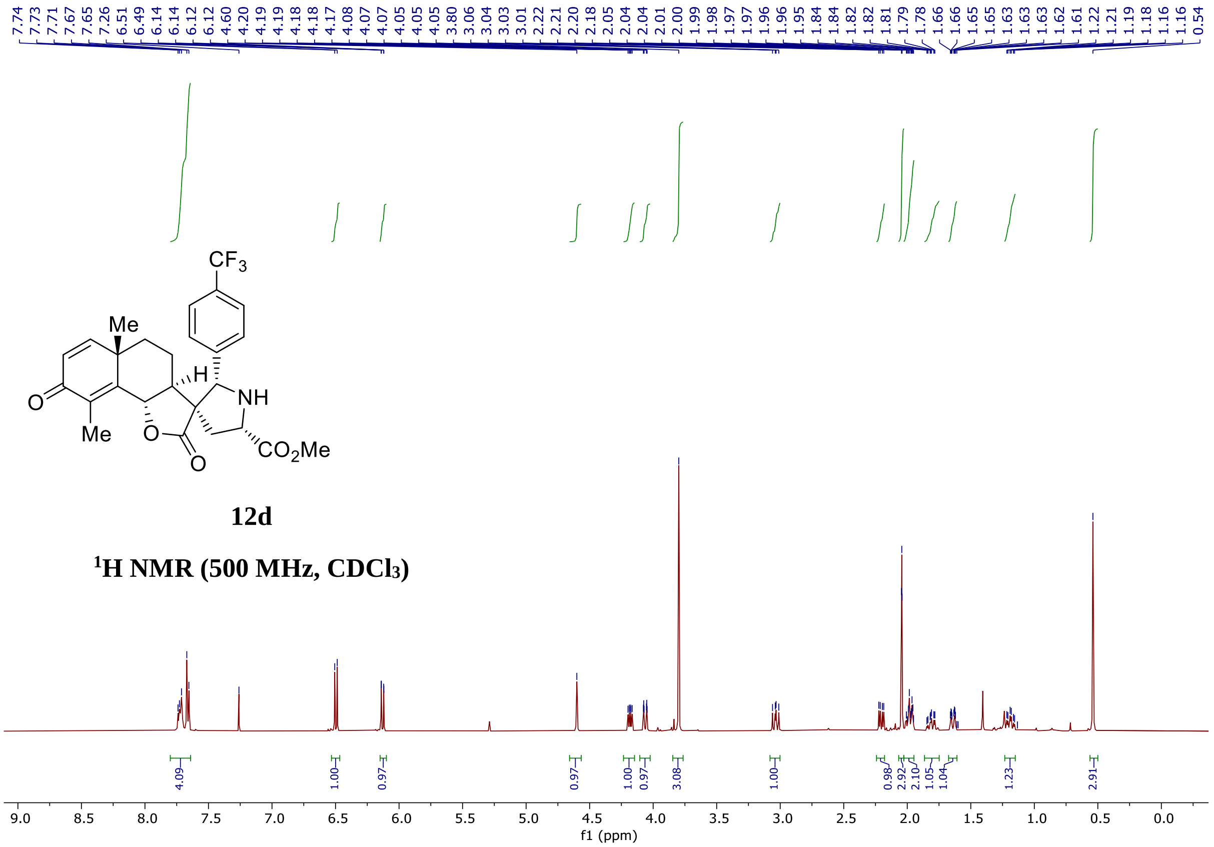

12d

^{1}H NMR (500 MHz, $CDCl_3$)

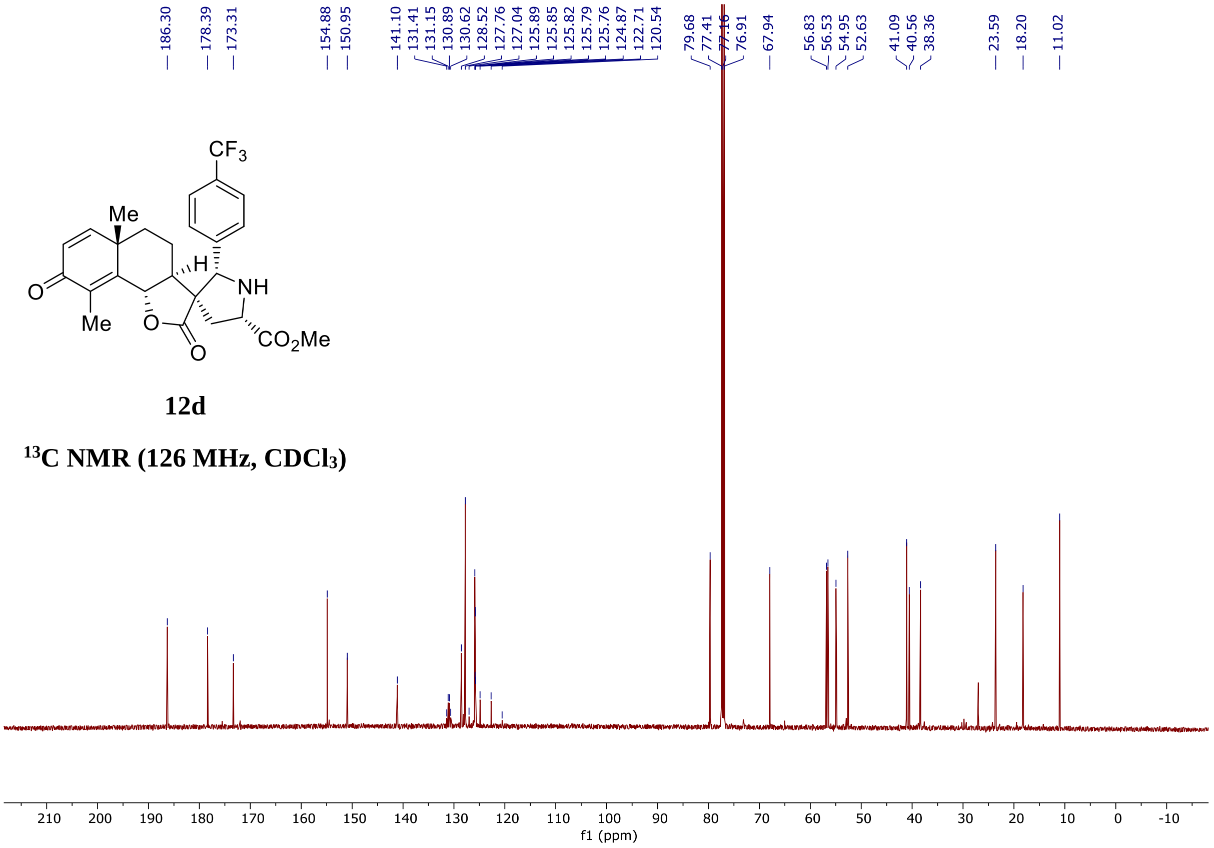

12d

^{13}C NMR (126 MHz, $CDCl_3$)

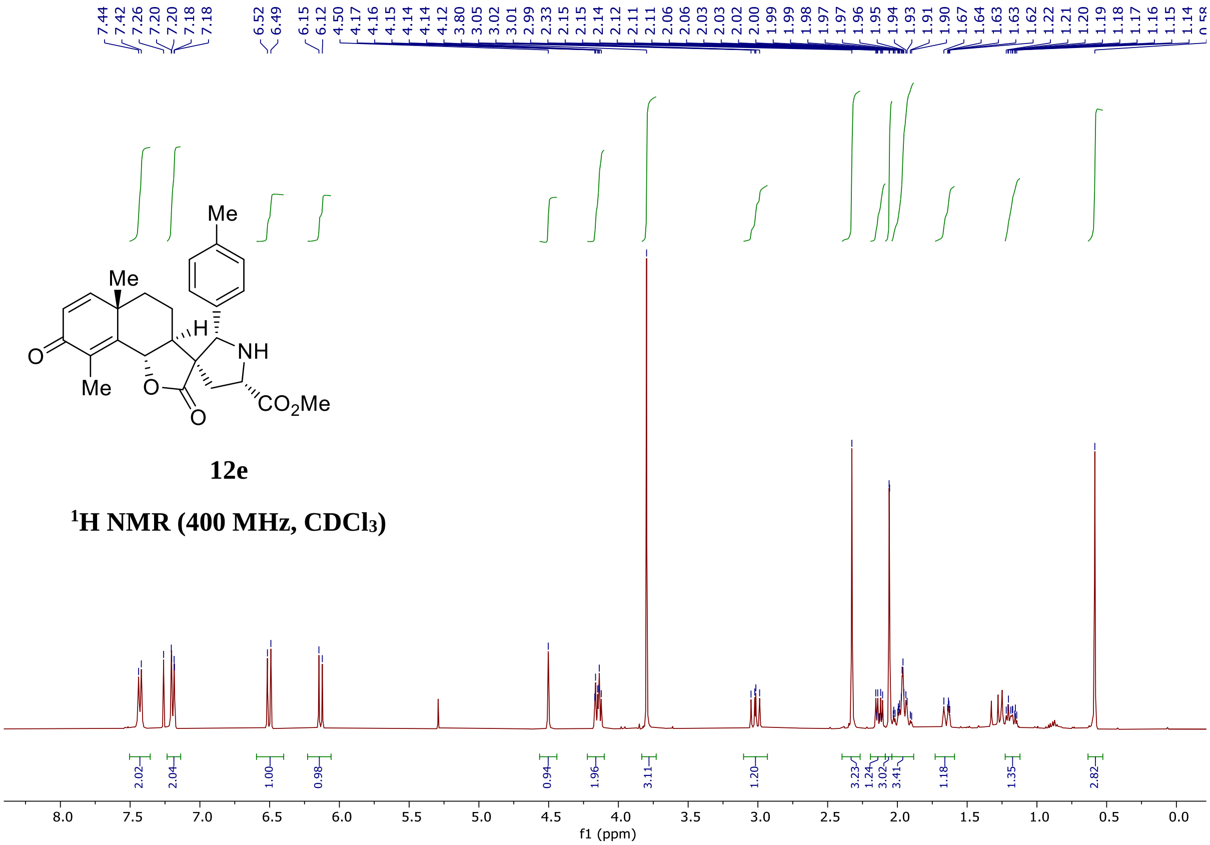

12e

^{1}H NMR (400 MHz, $CDCl_3$)

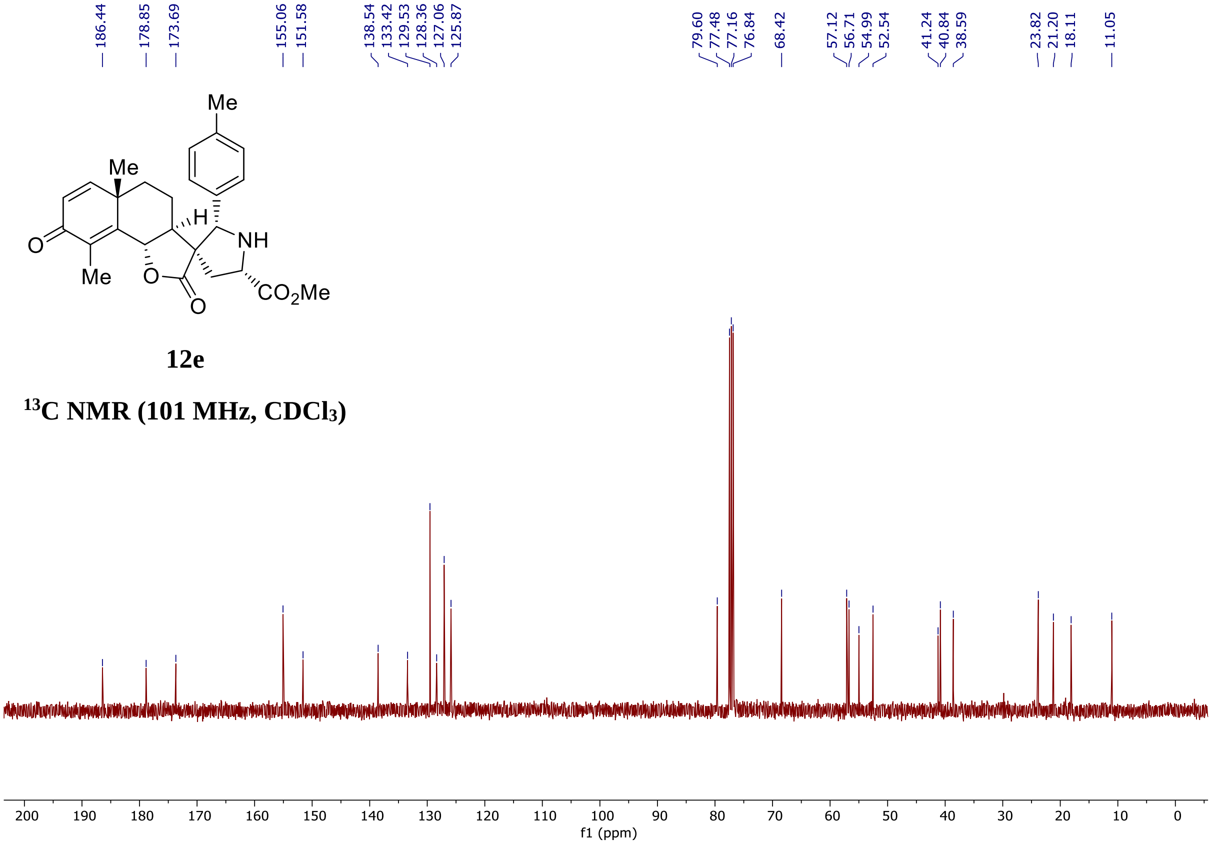

12e

^{13}C NMR (101 MHz, $CDCl_3$)

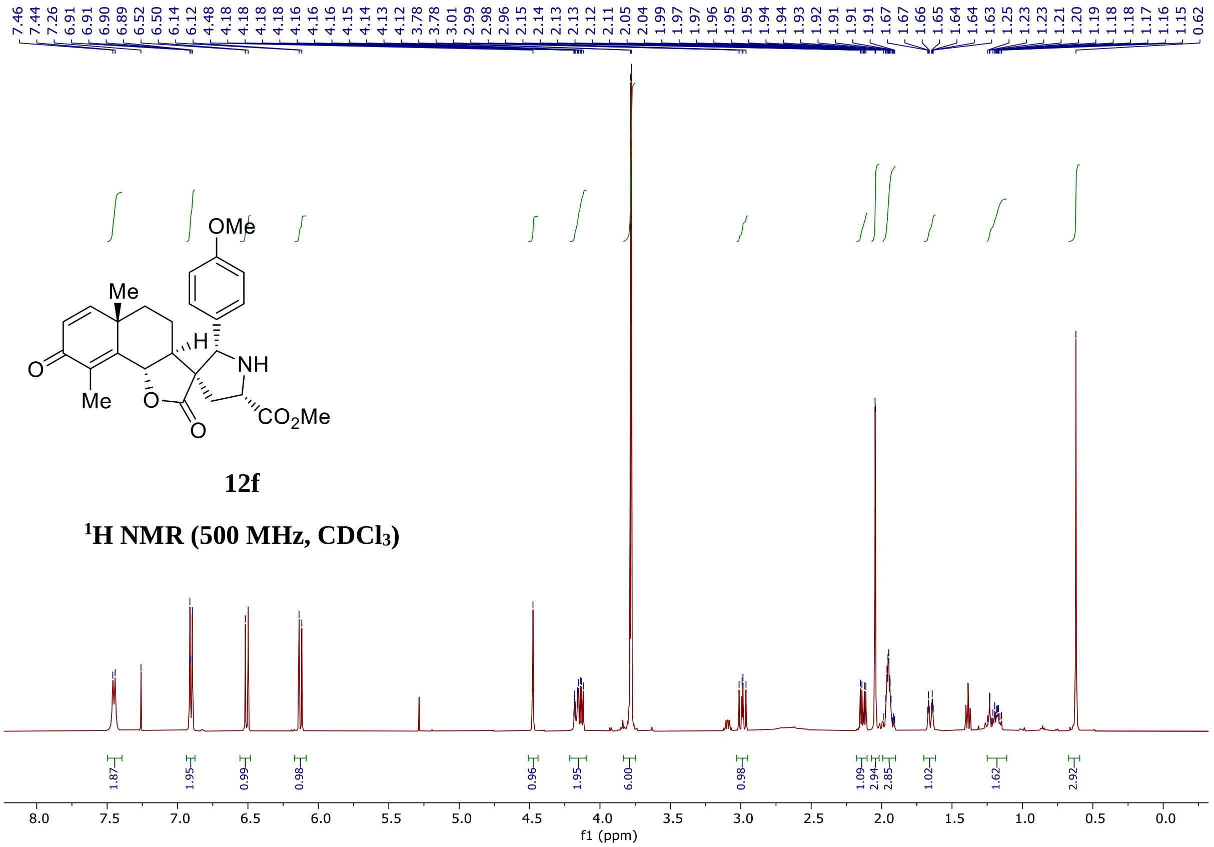

12f

^{1}H NMR (500 MHz, $CDCl_3$)

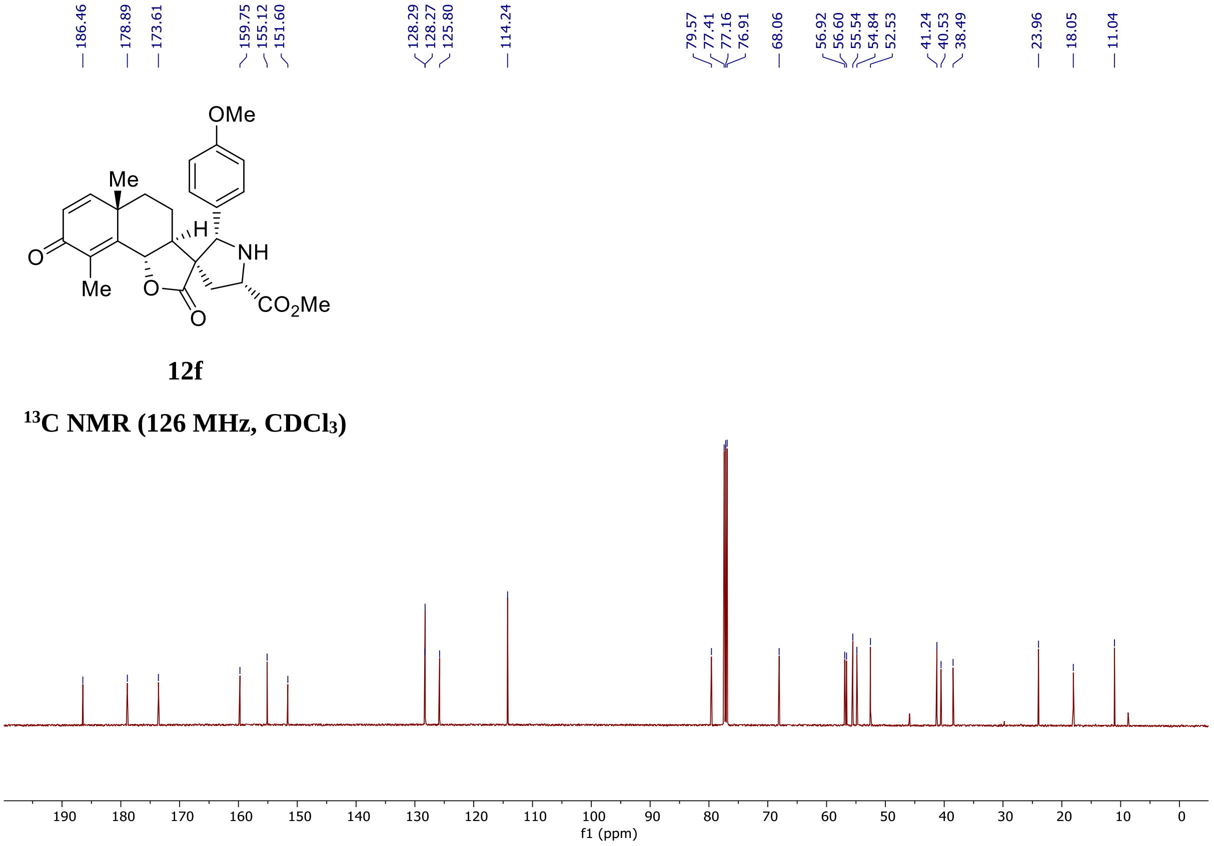

12f

^{13}C NMR (126 MHz, $CDCl_3$)

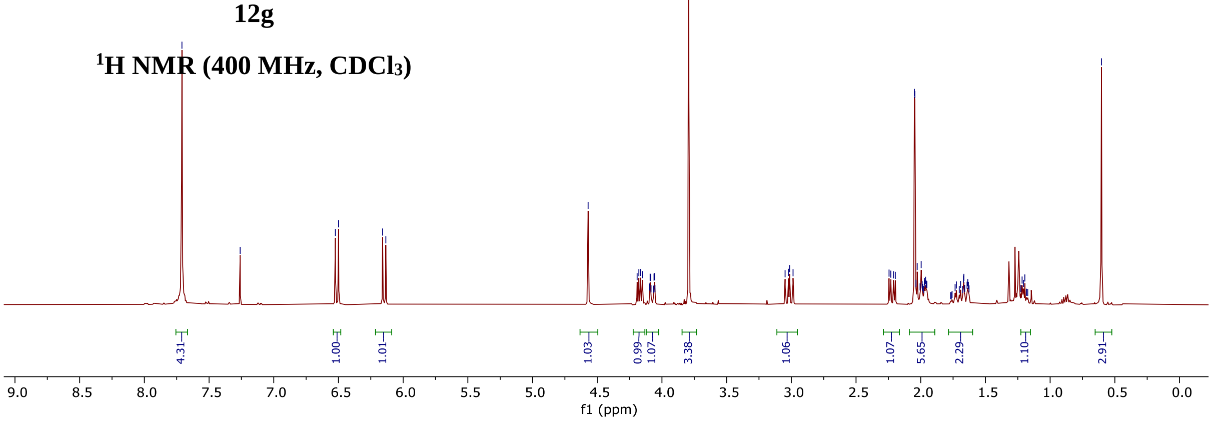

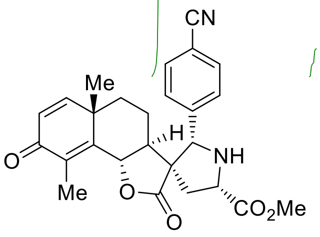

12g

^{1}H NMR (400 MHz, CDCl₃)

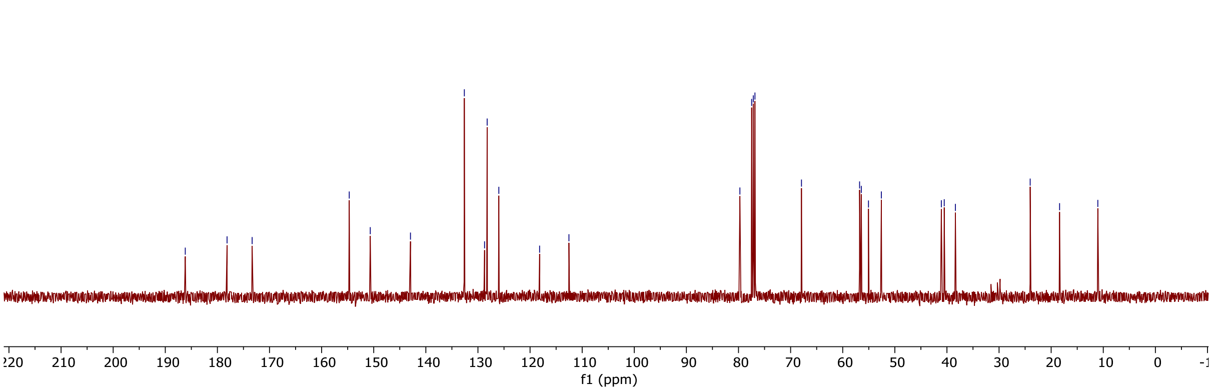

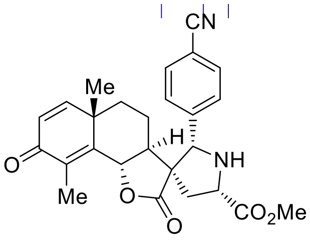

12g

^{13}C NMR (101 MHz, CDCl₃)

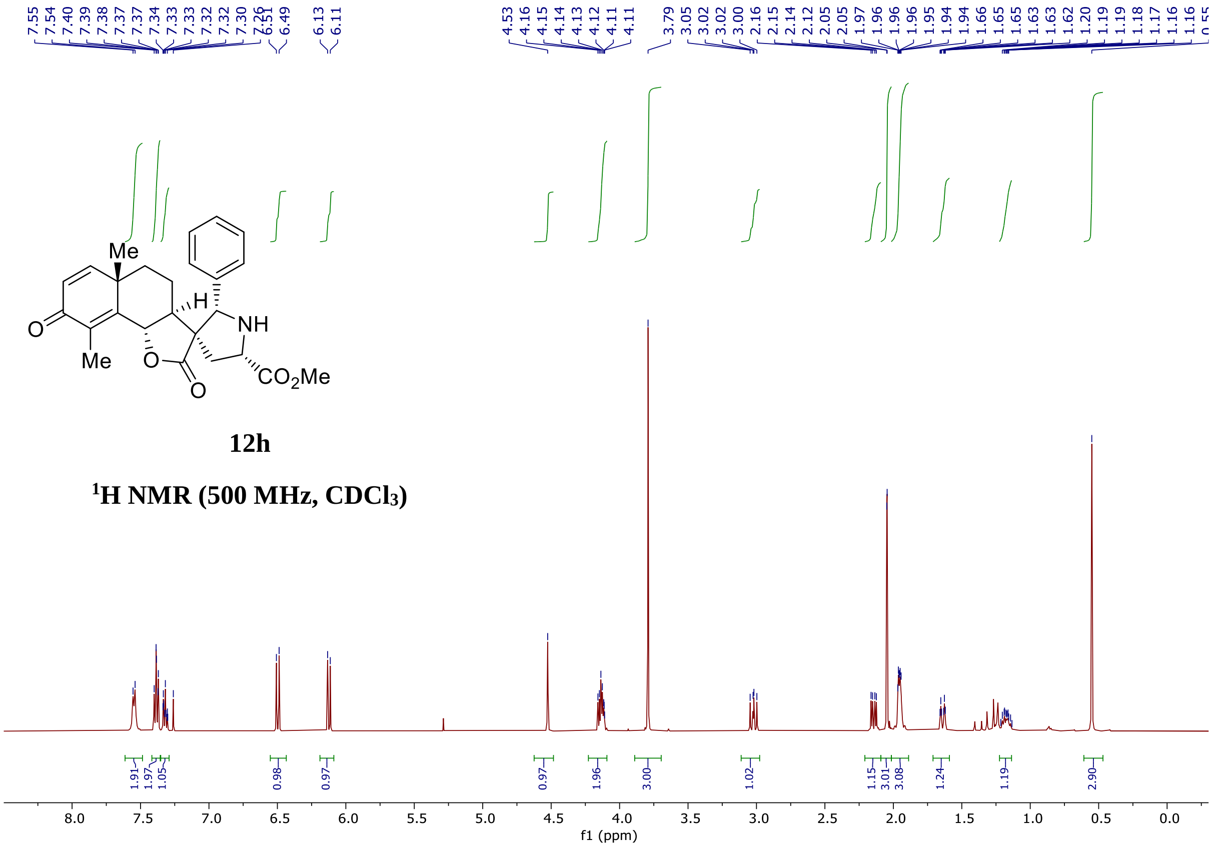

12h

^{1}H NMR (500 MHz, CDCl$_3$)

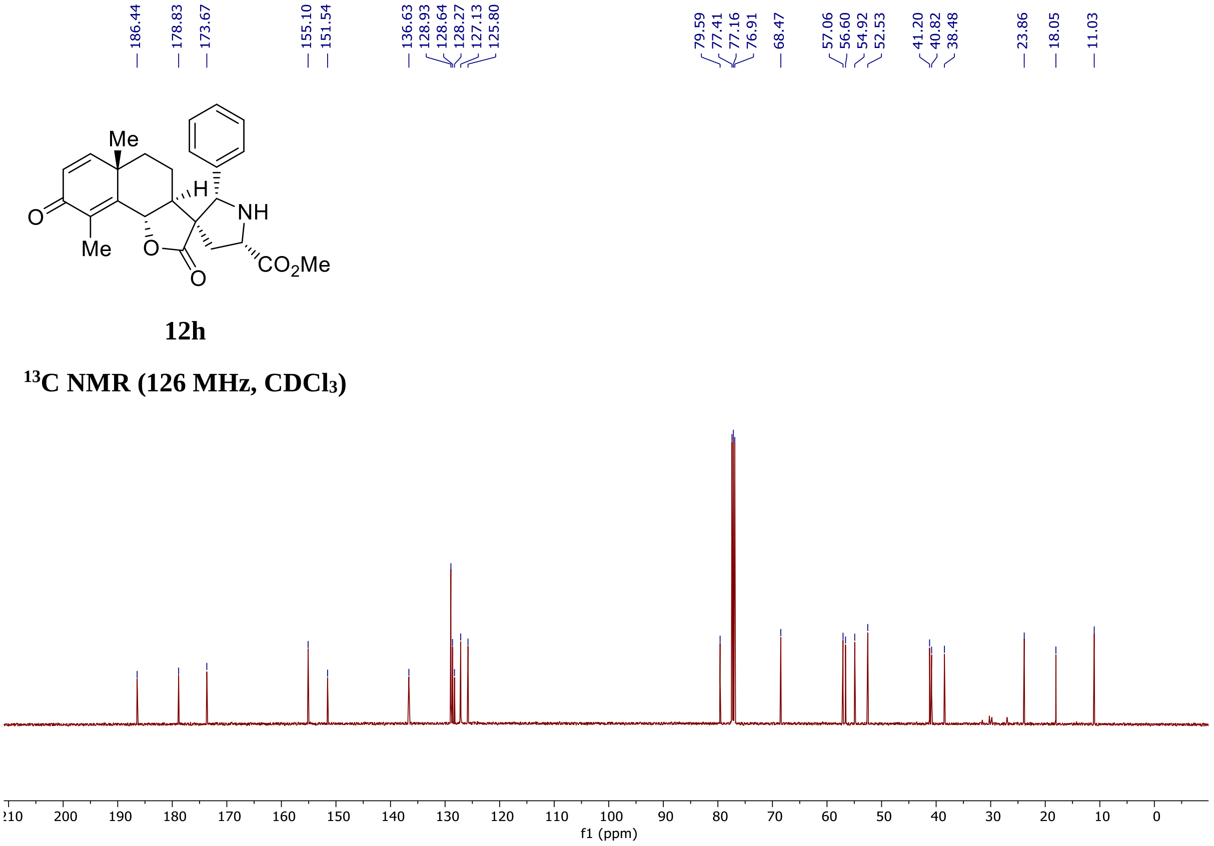

12h

^{13}C NMR (126 MHz, CDCl$_3$)

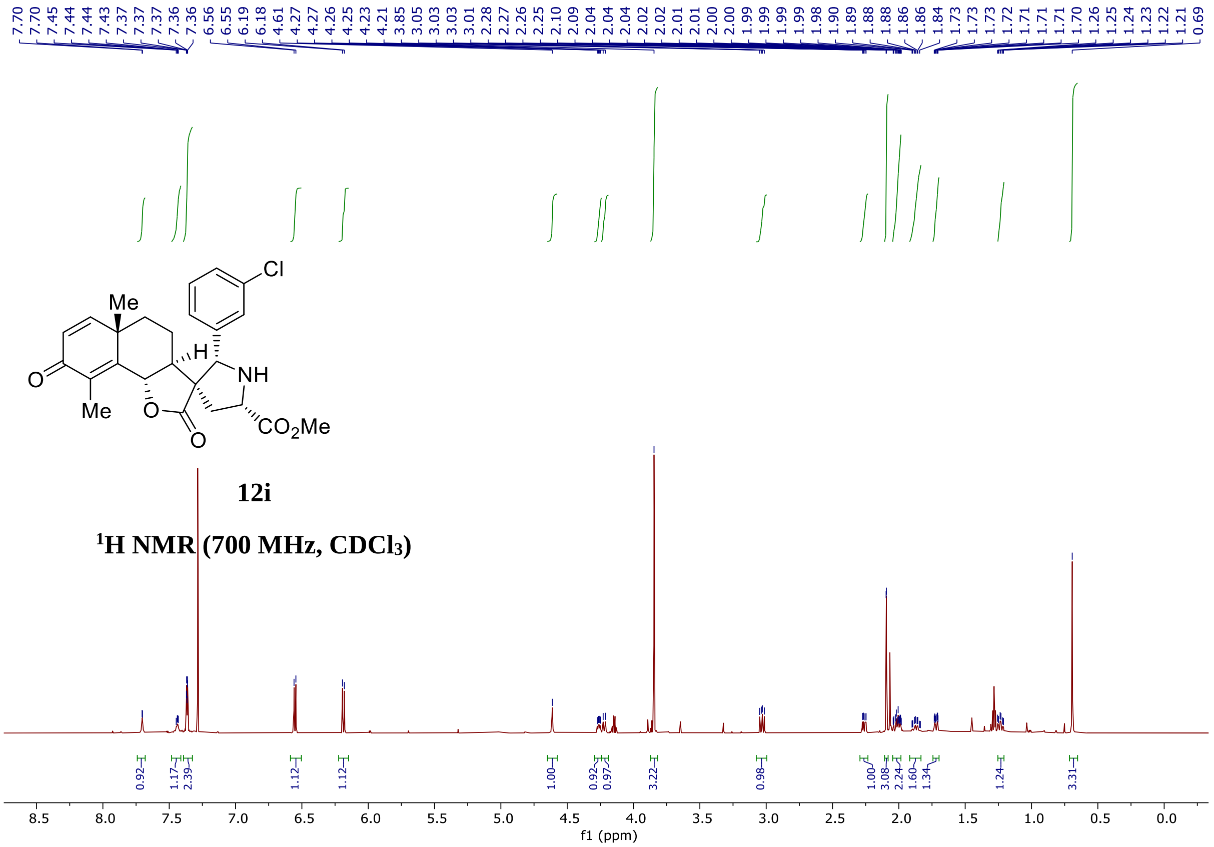

12i

^{1}H NMR (700 MHz, $CDCl_3$)

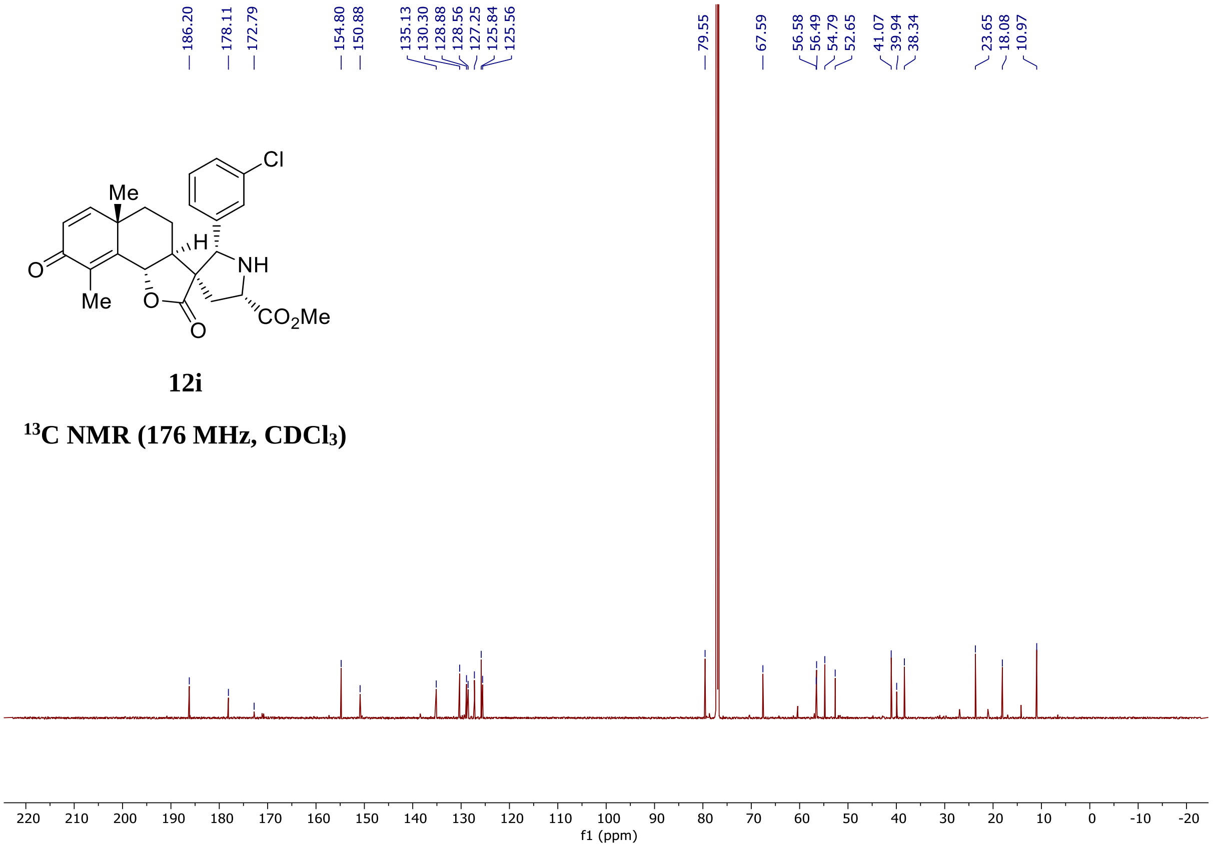

12i

^{13}C NMR (176 MHz, $CDCl_3$)

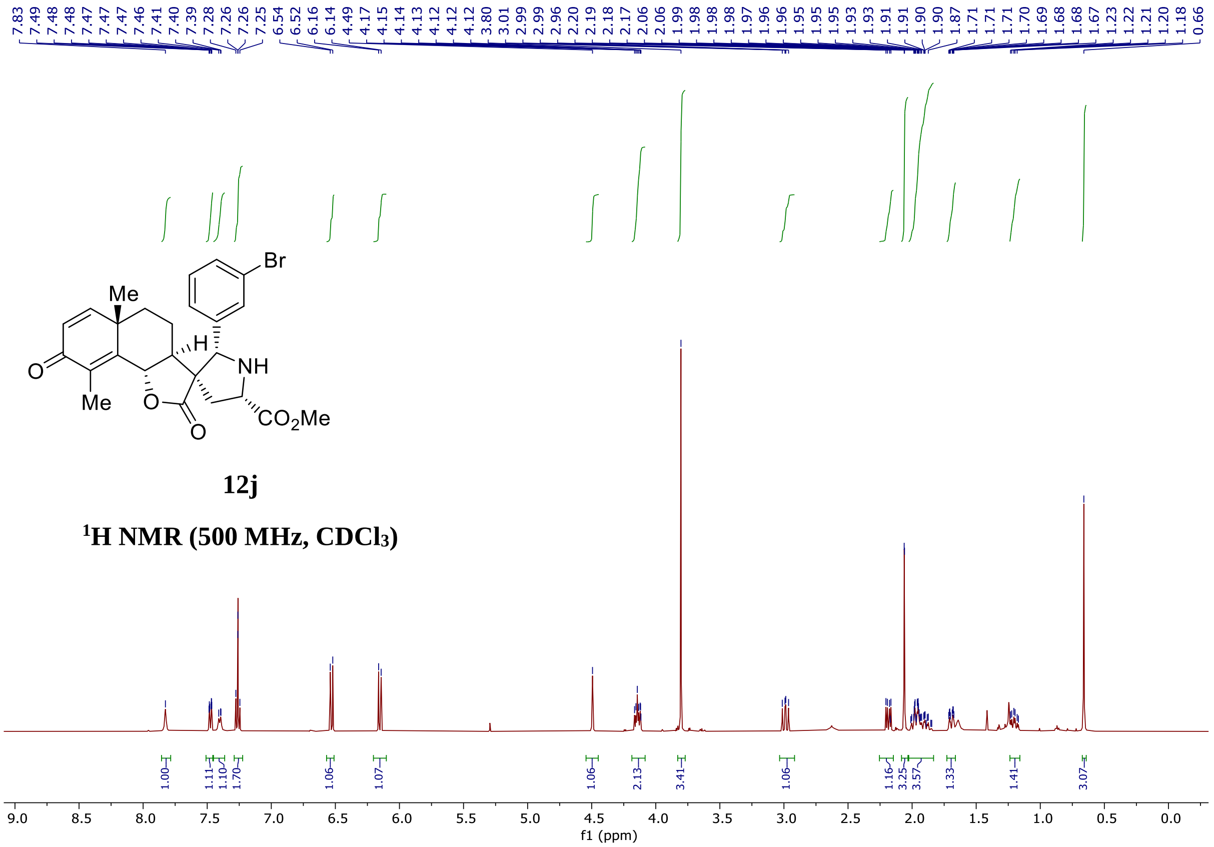

12j

^{1}H NMR (500 MHz, CDCl$_3$)

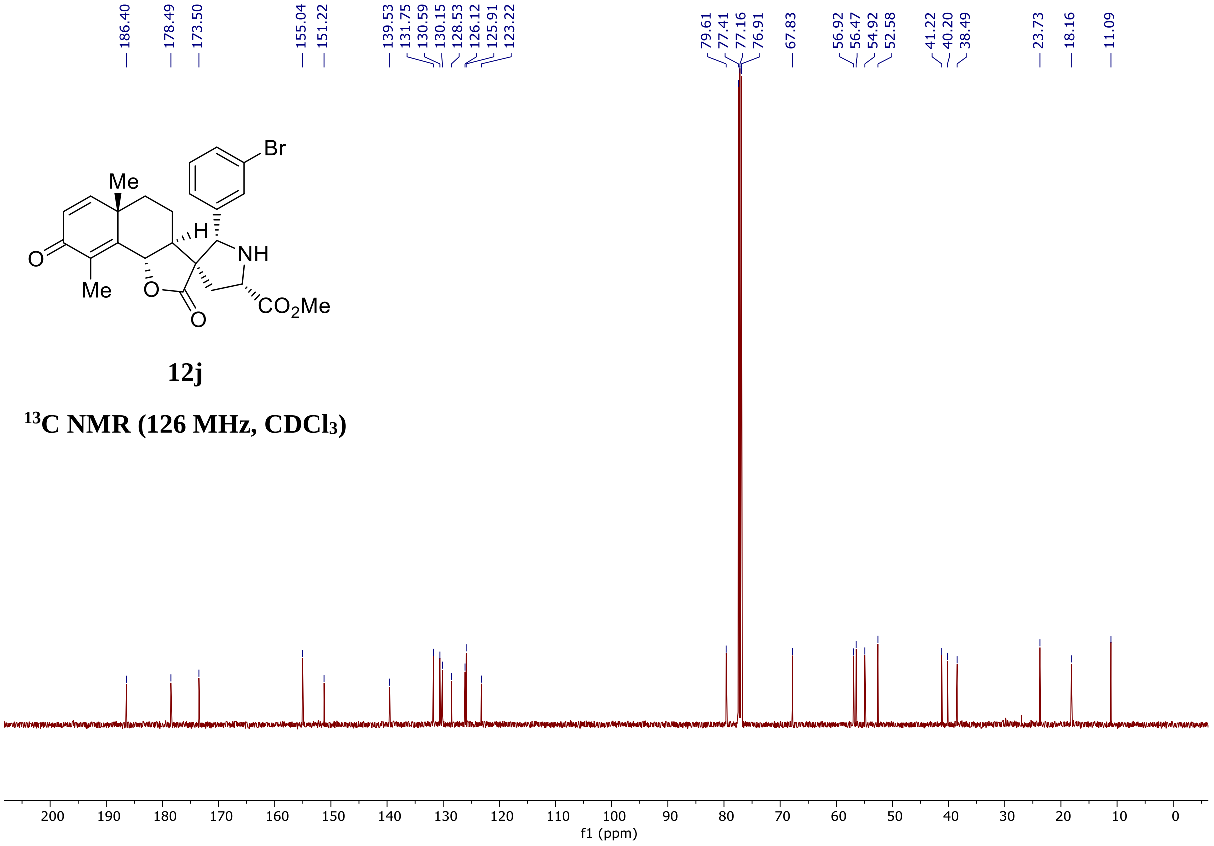

12j

^{13}C NMR (126 MHz, CDCl$_3$)

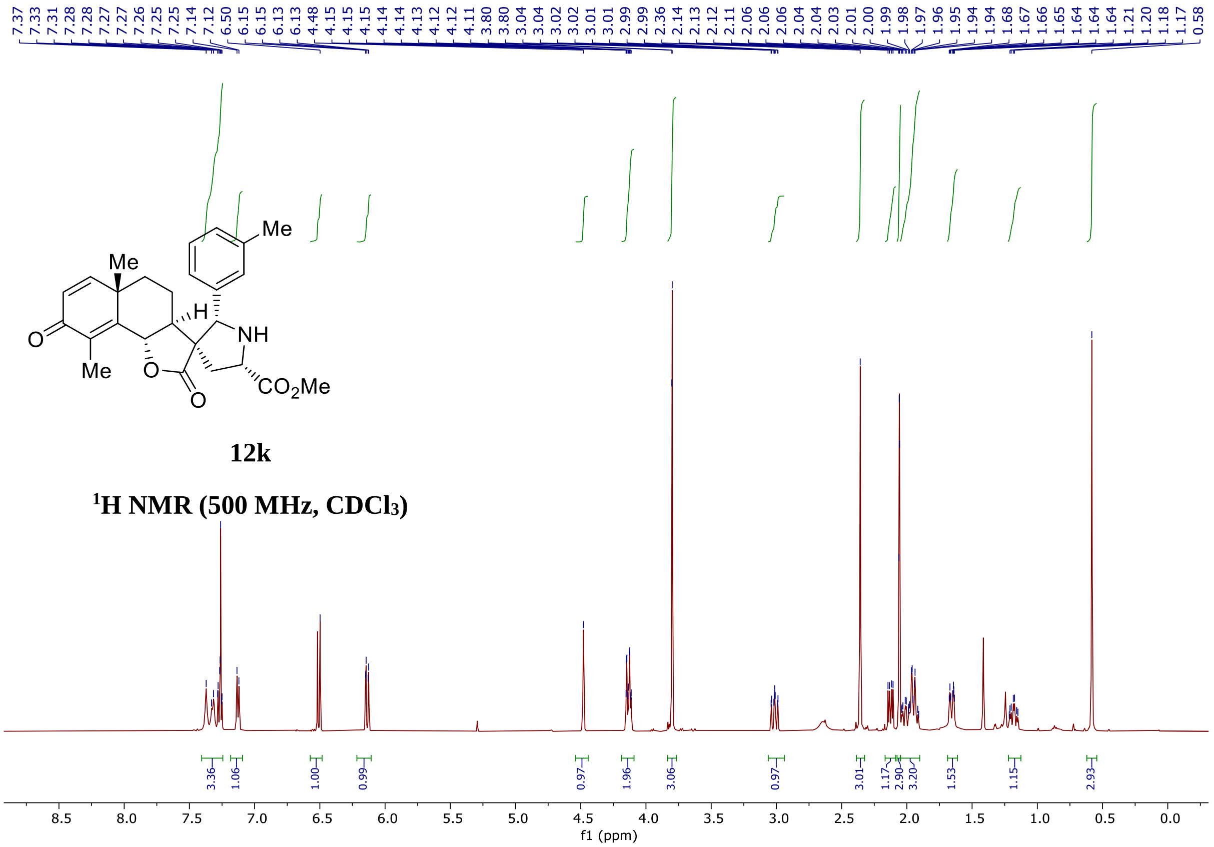
7.37 7.33 7.31 7.28 7.28 7.27 7.27 7.26 7.25 7.25 7.14 7.12 6.50 6.15 6.13 6.13 4.48 4.15 4.15 4.15 4.14 4.14 4.13 4.12 4.12 4.11 3.80 3.80 3.04 3.04 3.02 3.02 3.01 3.01 2.99 2.99 2.36 2.14 2.13 2.12 2.11 2.06 2.06 2.04 2.04 2.03 2.01 2.00 1.99 1.98 1.97 1.96 1.95 1.94 1.94 1.68 1.67 1.66 1.65 1.64 1.64 1.64 1.21 1.20 1.18 1.17 0.58
Me
Me
H
NH
O
Me
O
O
CO2Me
12k
1H NMR (500 MHz, CDCl3)
3.36 1.06 1.00 0.99 0.97 1.96 3.06 0.97 3.01 1.17 2.90 3.20 1.53 1.15 2.93
f1 (ppm)

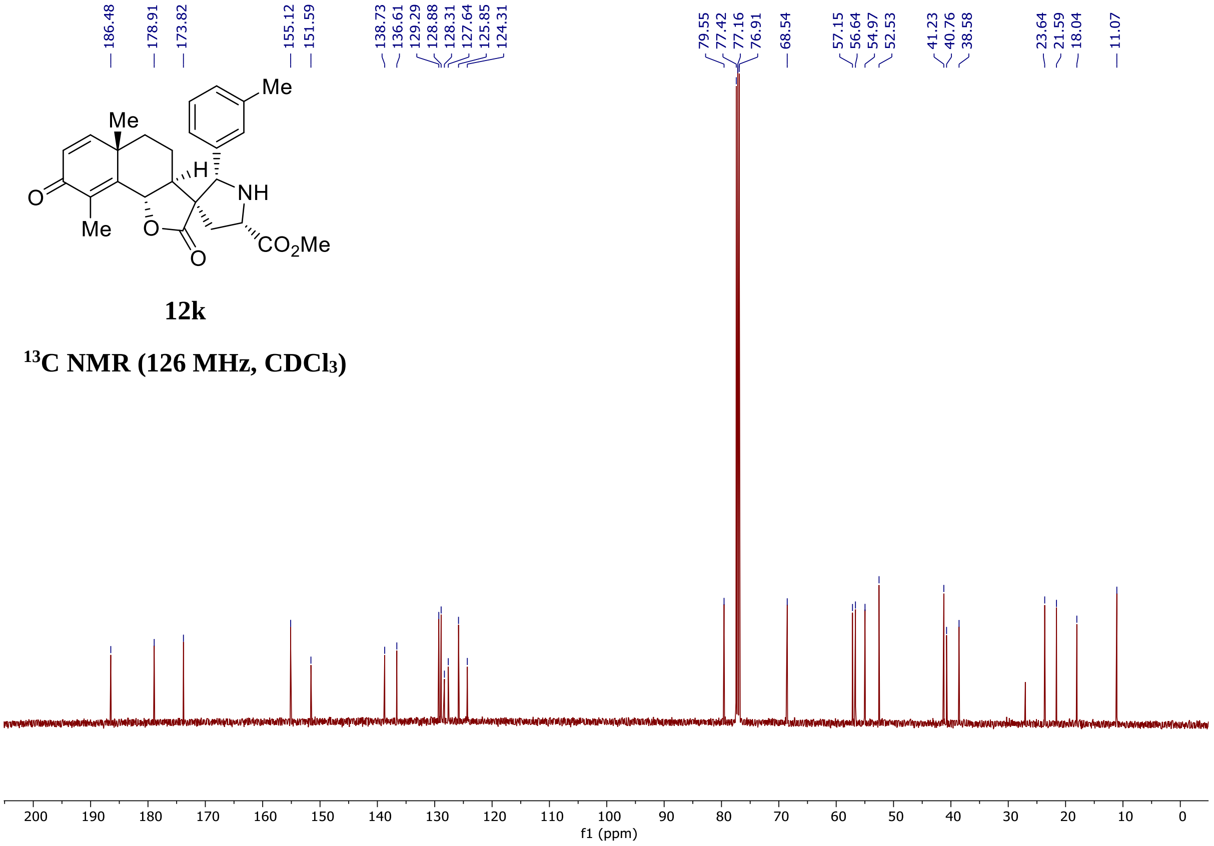
186.48 178.91 173.82 155.12 151.59 138.73 136.61 129.29 128.88 128.31 127.64 125.85 124.31 79.55 77.42 77.16 76.91 68.54 57.15 56.64 54.97 52.53 41.23 40.76 38.58 23.64 21.59 18.04 11.07
Me
Me
H
NH
O
Me
O
O
CO2Me
12k
13C NMR (126 MHz, CDCl3)
f1 (ppm)

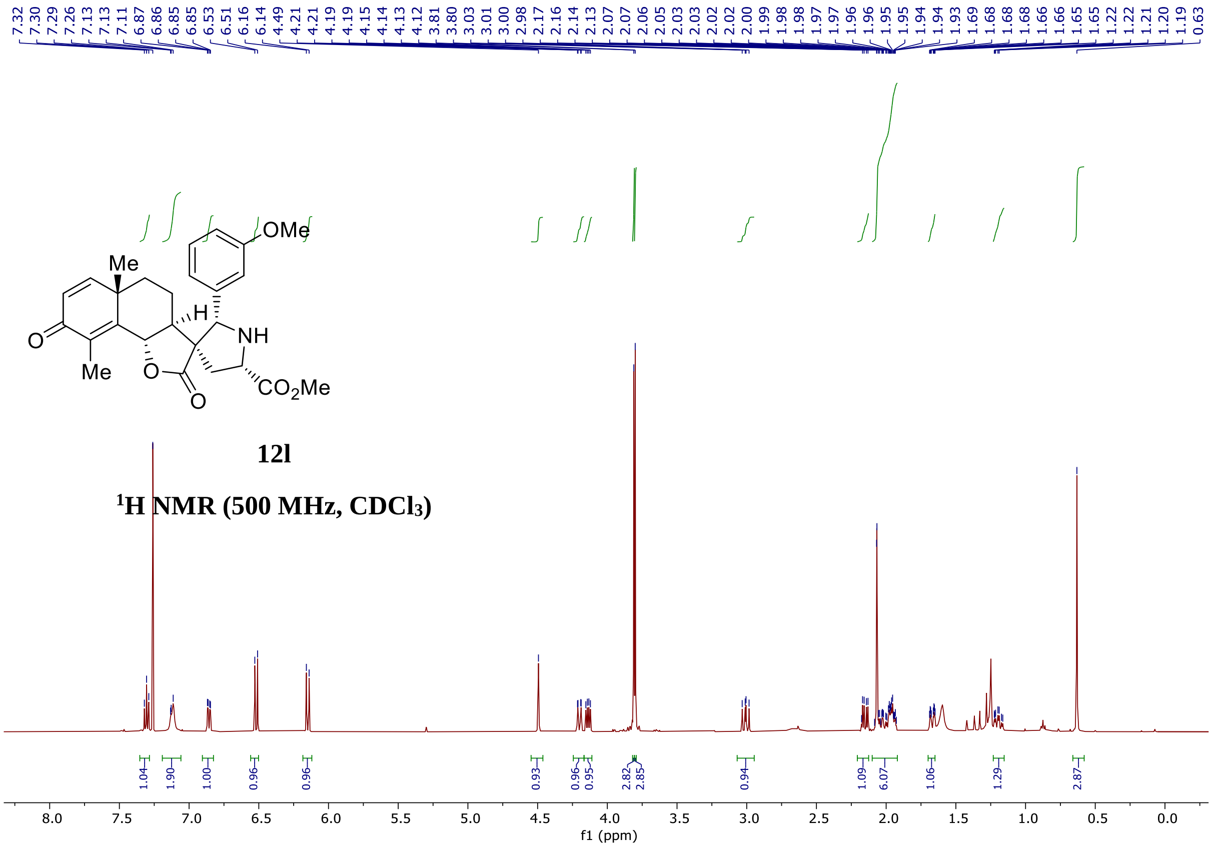

12l

^{1}H NMR (500 MHz, CDCl3)

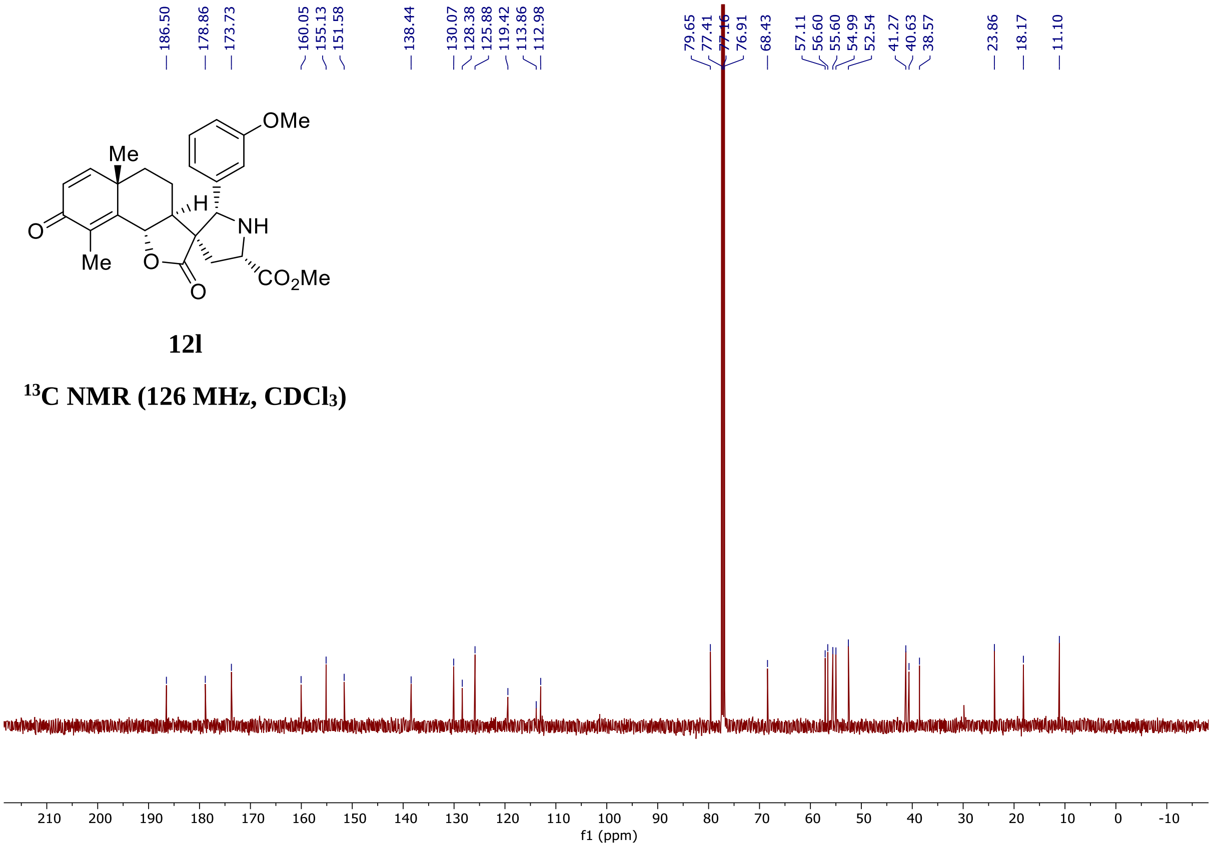

12l

^{13}C NMR (126 MHz, CDCl3)

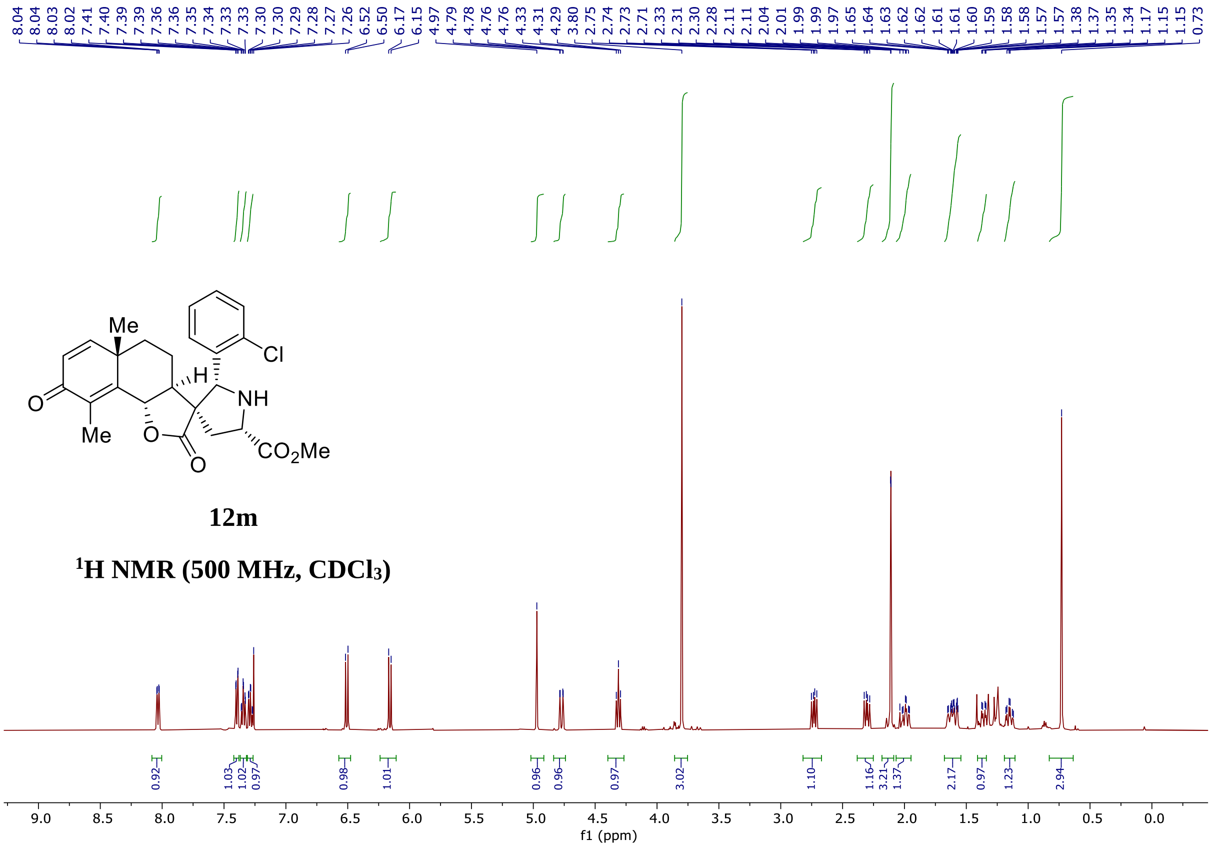
8.04 8.04 8.03 8.02 7.41 7.40 7.39 7.39 7.36 7.36 7.35 7.34 7.33 7.33 7.30 7.30 7.29 7.28 7.27 7.26 6.52 6.50 6.17 6.15 4.97 4.79 4.78 4.76 4.76 4.33 4.31 4.29 3.80 2.75 2.74 2.73 2.71 2.33 2.31 2.30 2.28 2.11 2.11 2.04 2.01 1.99 1.99 1.97 1.65 1.64 1.63 1.62 1.62 1.61 1.61 1.60 1.59 1.58 1.57 1.57 1.38 1.37 1.35 1.34 1.17 1.15 1.15 0.73
Me
H
Cl
NH
O
Me
O
O
CO_2Me
12m
^{1}H NMR (500 MHz, CDCl3)
0.92 1.03 1.02 0.97 0.98 1.01 0.96 0.96 0.97 3.02 1.10 1.16 3.21 1.37 2.17 0.97 1.23 2.94
9.0 8.5 8.0 7.5 7.0 6.5 6.0 5.5 5.0 4.5 4.0 3.5 3.0 2.5 2.0 1.5 1.0 0.5 0.0
f1 (ppm)

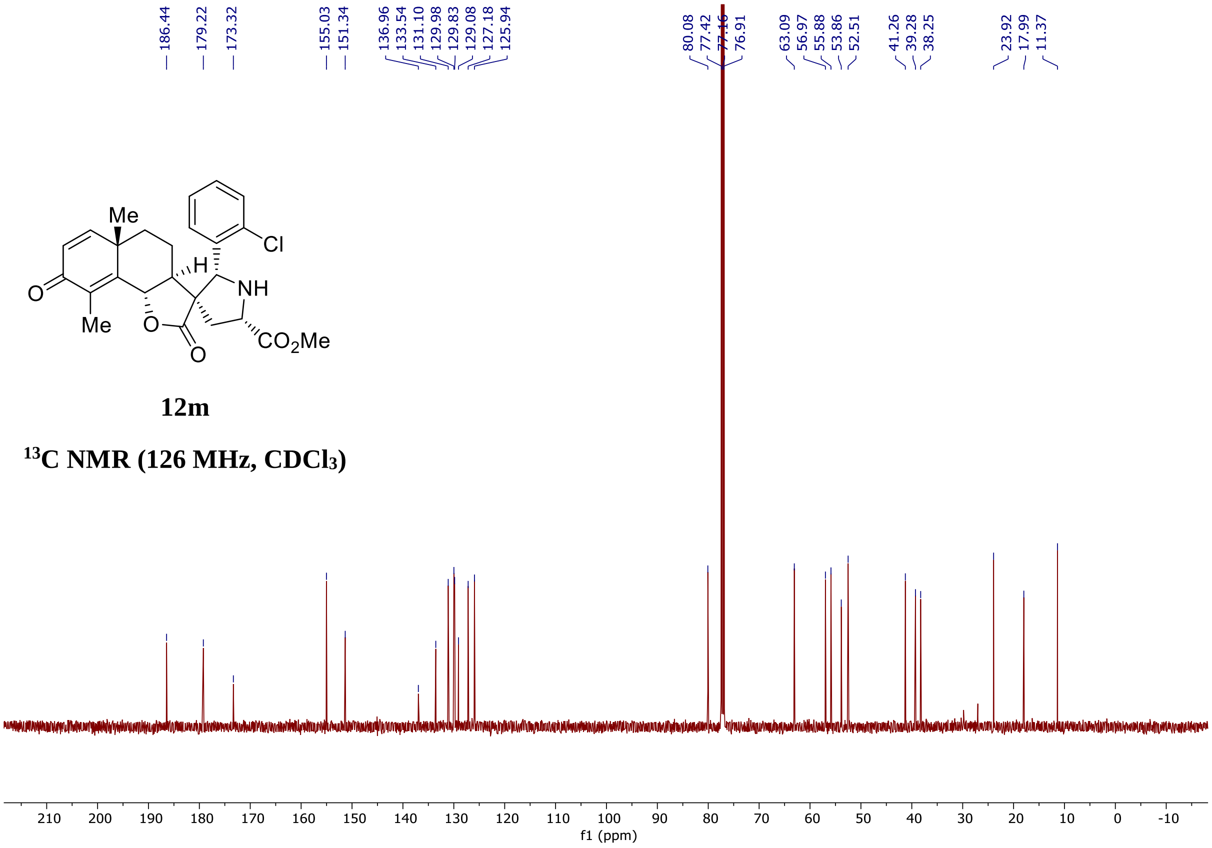
186.44 179.22 173.32 155.03 151.34 136.96 133.54 131.10 129.98 129.83 129.08 127.18 125.94 80.08 77.42 77.16 76.91 63.09 56.97 55.88 53.86 52.51 41.26 39.28 38.25 23.92 17.99 11.37
Me
H
Cl
NH
O
Me
O
O
CO_2Me
12m
^{13}C NMR (126 MHz, CDCl3)
210 200 190 180 170 160 150 140 130 120 110 100 90 80 70 60 50 40 30 20 10 0 -10
f1 (ppm)

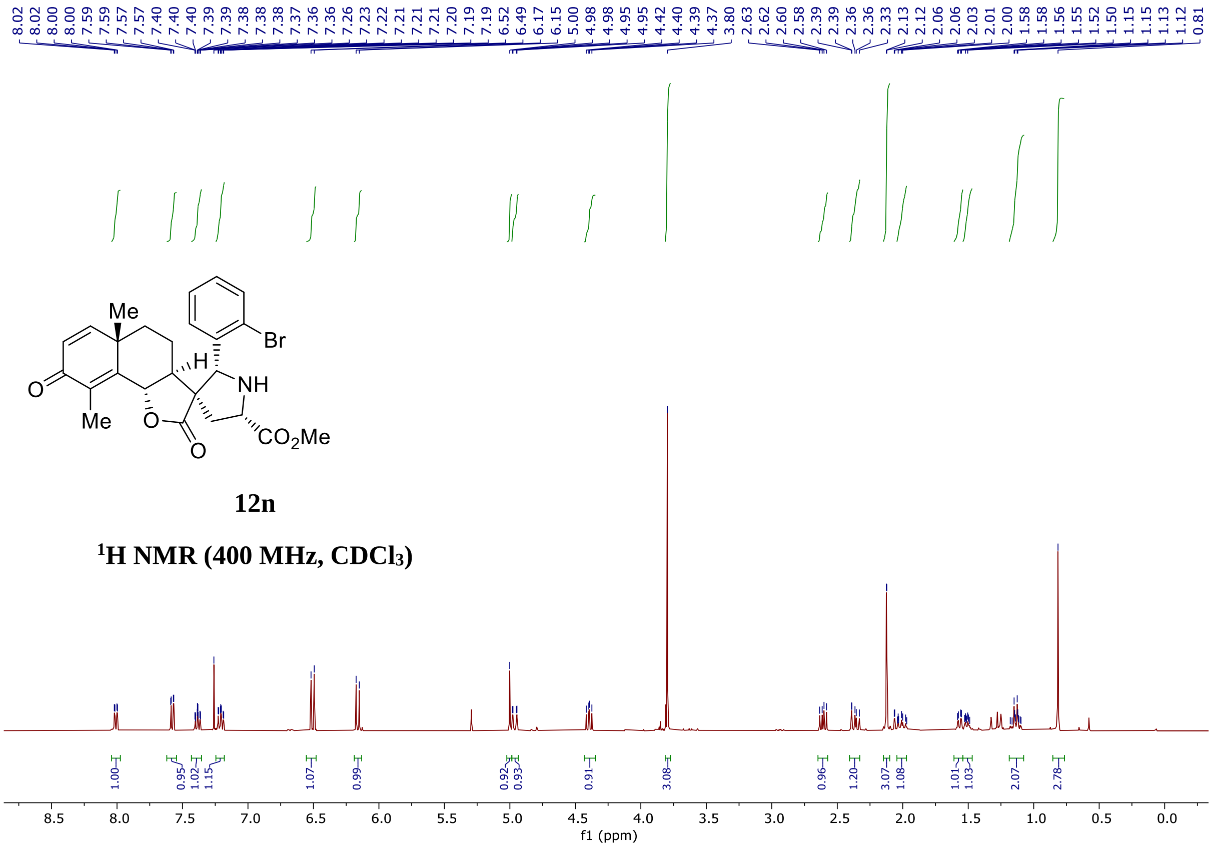

12n

^{1}H NMR (400 MHz, CDCl3)

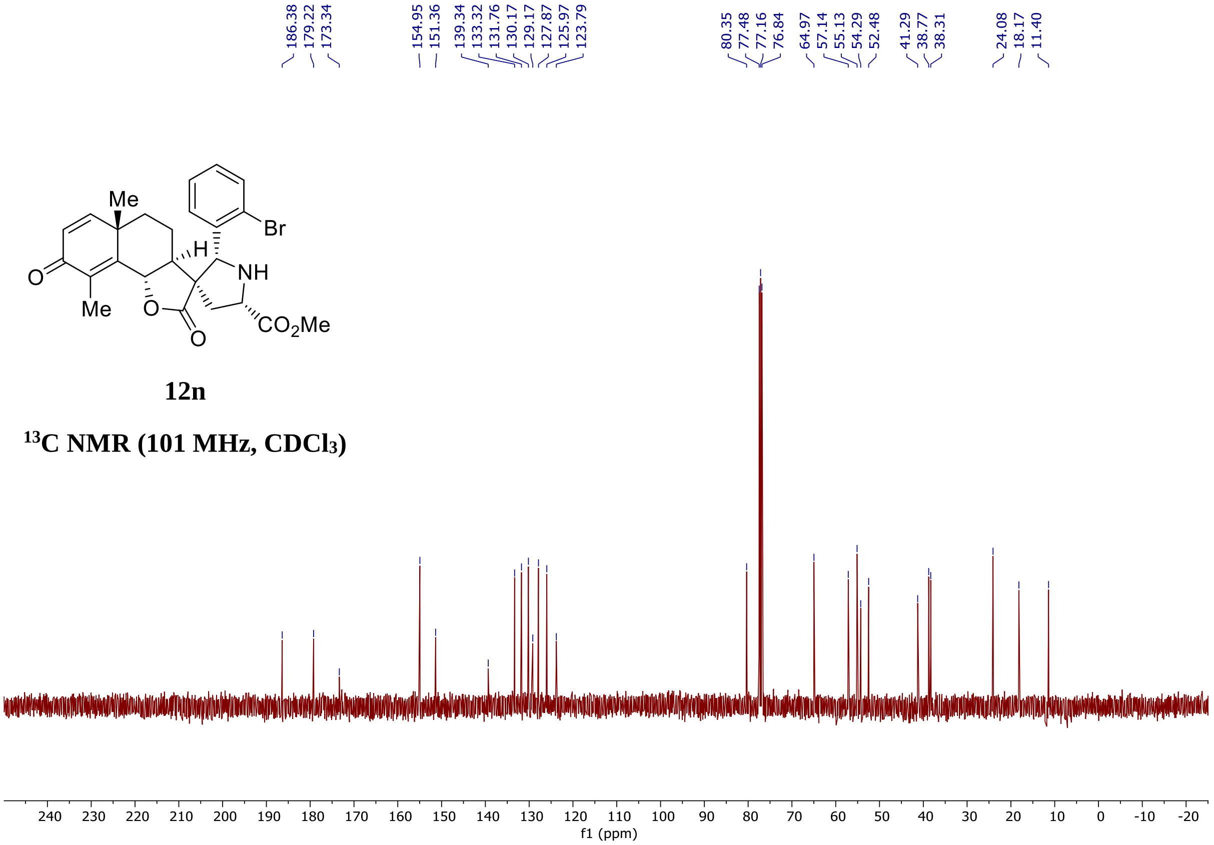

12n

^{13}C NMR (101 MHz, CDCl3)

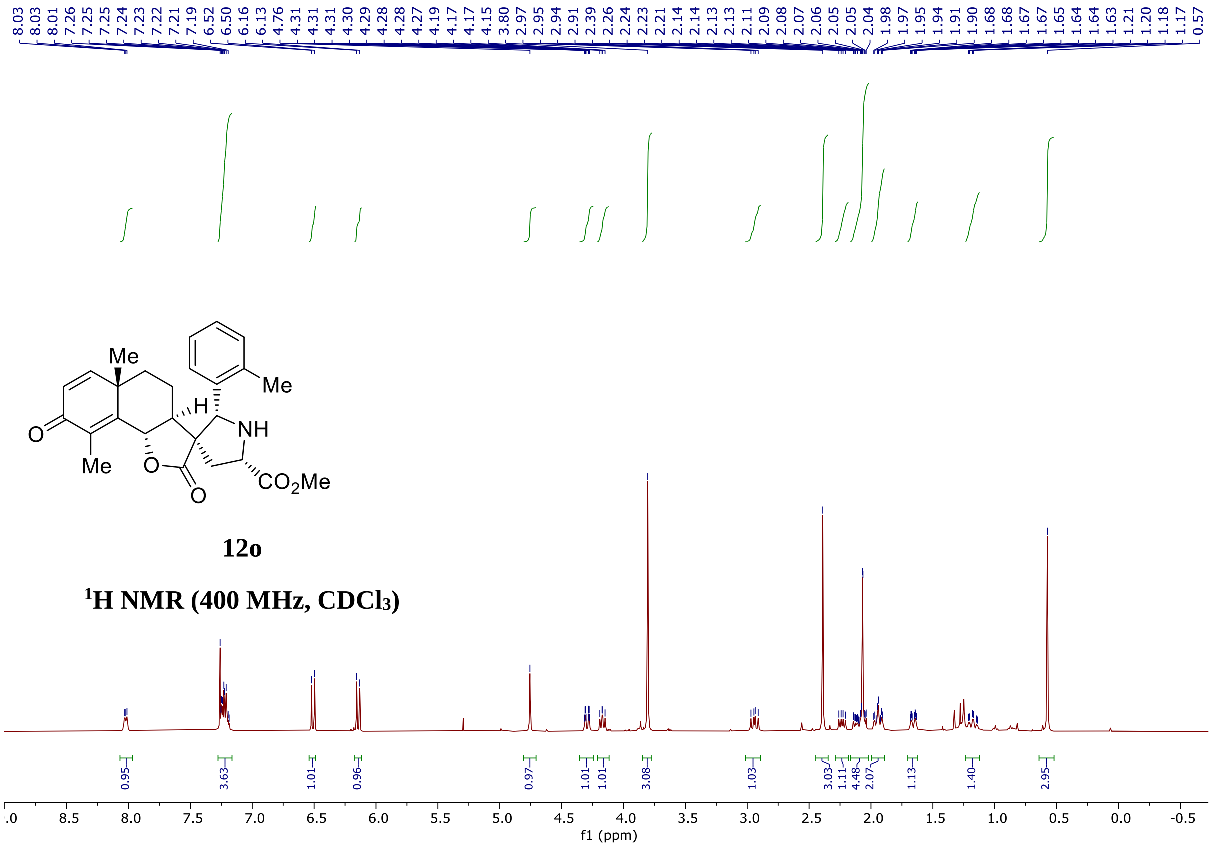

8.03 8.03 8.01 7.26 7.25 7.25 7.24 7.23 7.22 7.21 7.19 6.52 6.50 6.16 6.13 4.76 4.31 4.31 4.30 4.29 4.28 4.28 4.27 4.19 4.17 4.17 4.15 3.80 2.97 2.95 2.94 2.91 2.39 2.26 2.24 2.23 2.21 2.14 2.13 2.13 2.11 2.09 2.08 2.07 2.06 2.05 2.05 2.04 1.98 1.97 1.95 1.94 1.91 1.90 1.68 1.68 1.67 1.67 1.65 1.64 1.64 1.63 1.21 1.20 1.18 1.17 0.57
0.95 3.63 1.01 0.96 0.97 1.01 1.01 3.08 1.03 3.03 1.11 4.48 2.07 1.13 1.40 2.95
f1 (ppm)
12o
1H NMR (400 MHz, CDCl3)

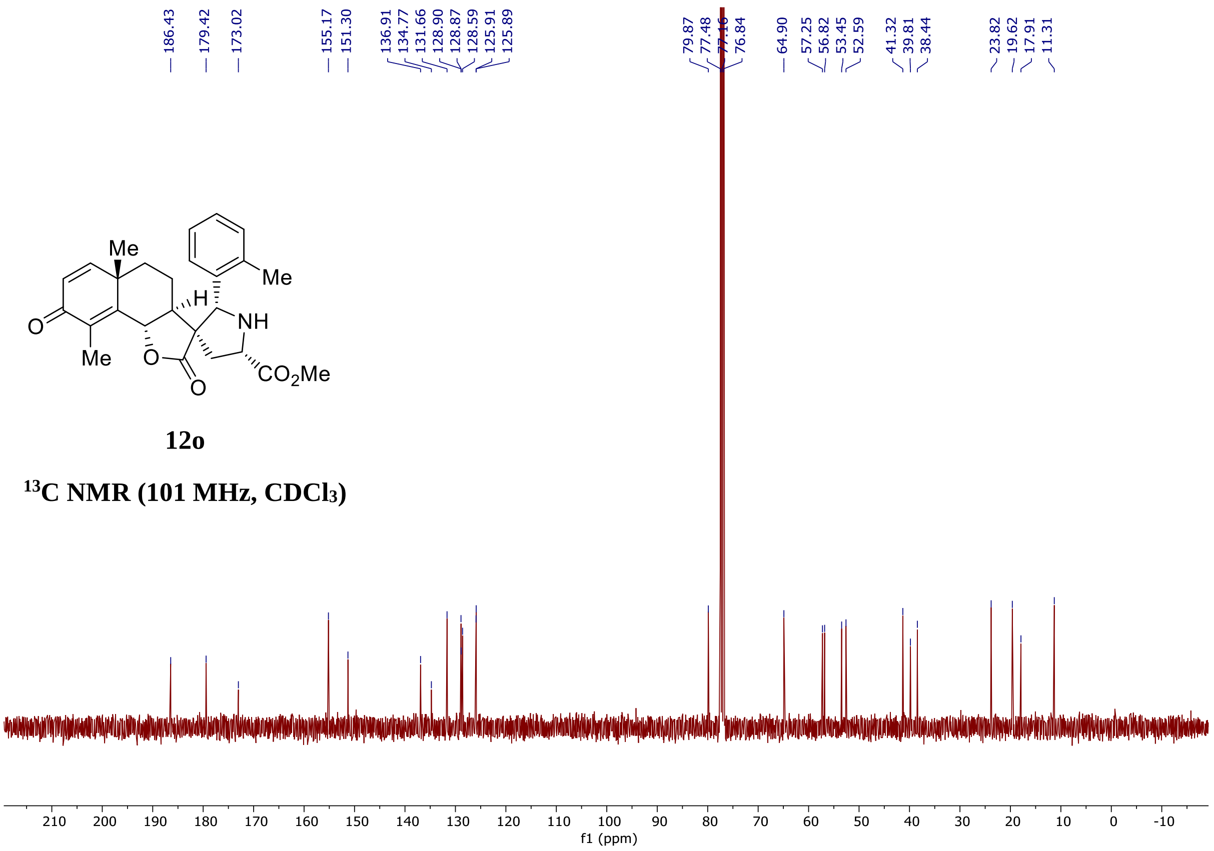

186.43 179.42 173.02 155.17 151.30 136.91 134.77 131.66 128.90 128.87 128.59 125.91 125.89 79.87 77.48 77.16 76.84 64.90 57.25 56.82 53.45 52.59 41.32 39.81 38.44 23.82 19.62 17.91 11.31
f1 (ppm)
12o
13C NMR (101 MHz, CDCl3)

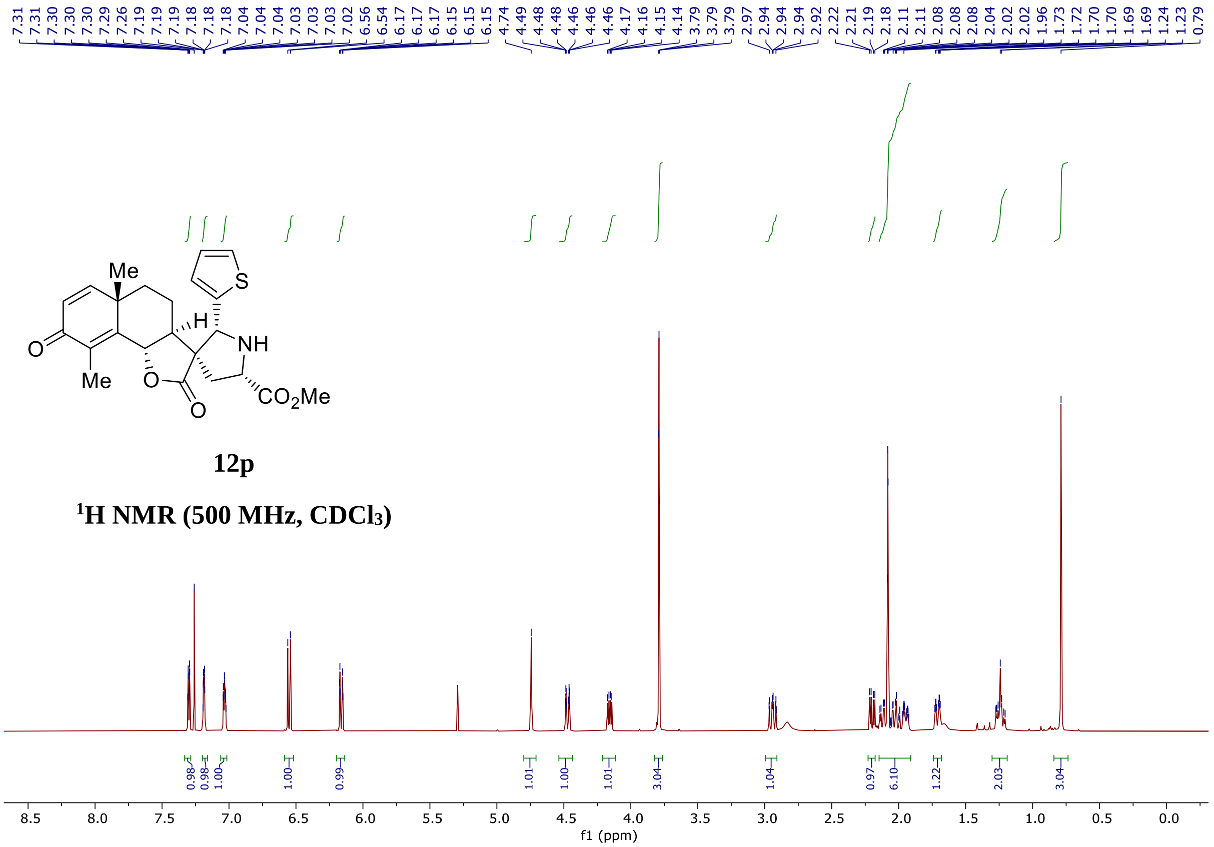

12p

^{1}H NMR (500 MHz, $CDCl_3$)

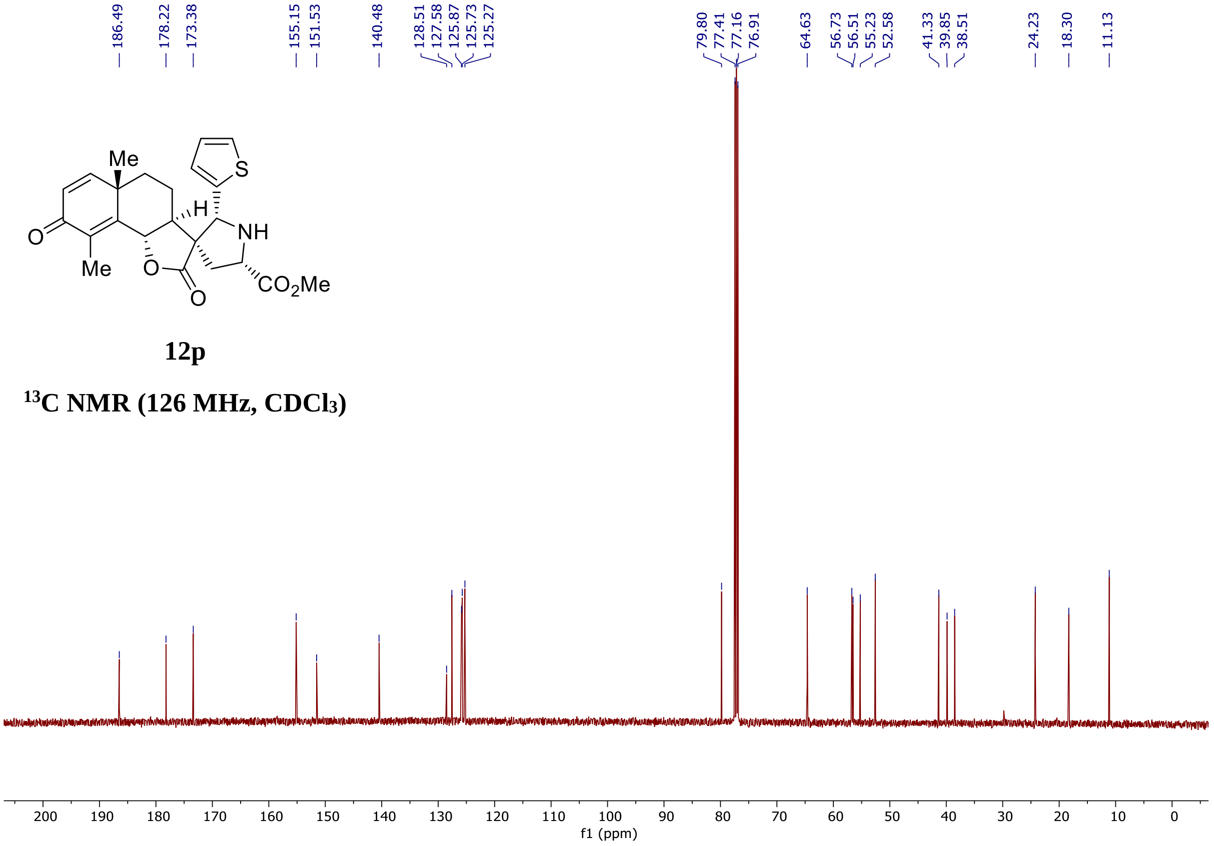

12p

^{13}C NMR (126 MHz, $CDCl_3$)

7.2 NMR spectra of SLs derivatives

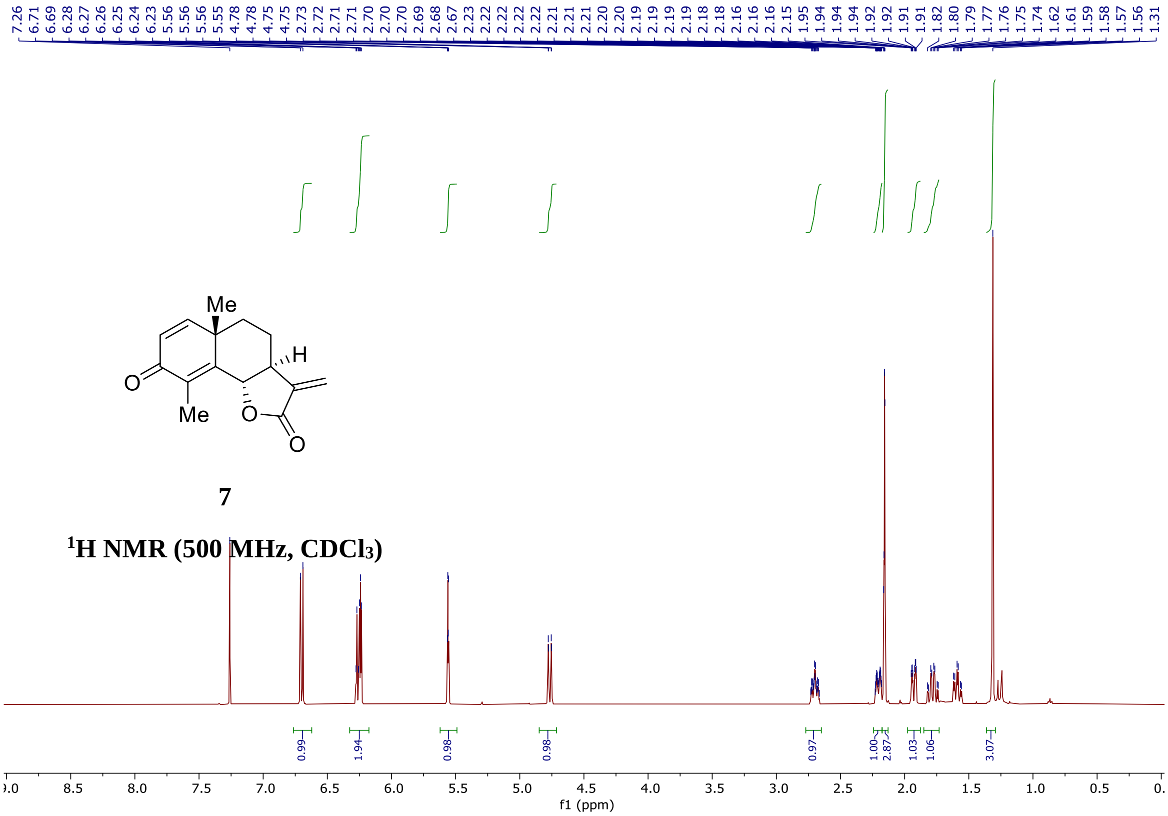

7

^{1}H NMR (500 MHz, CDCl$_3$)

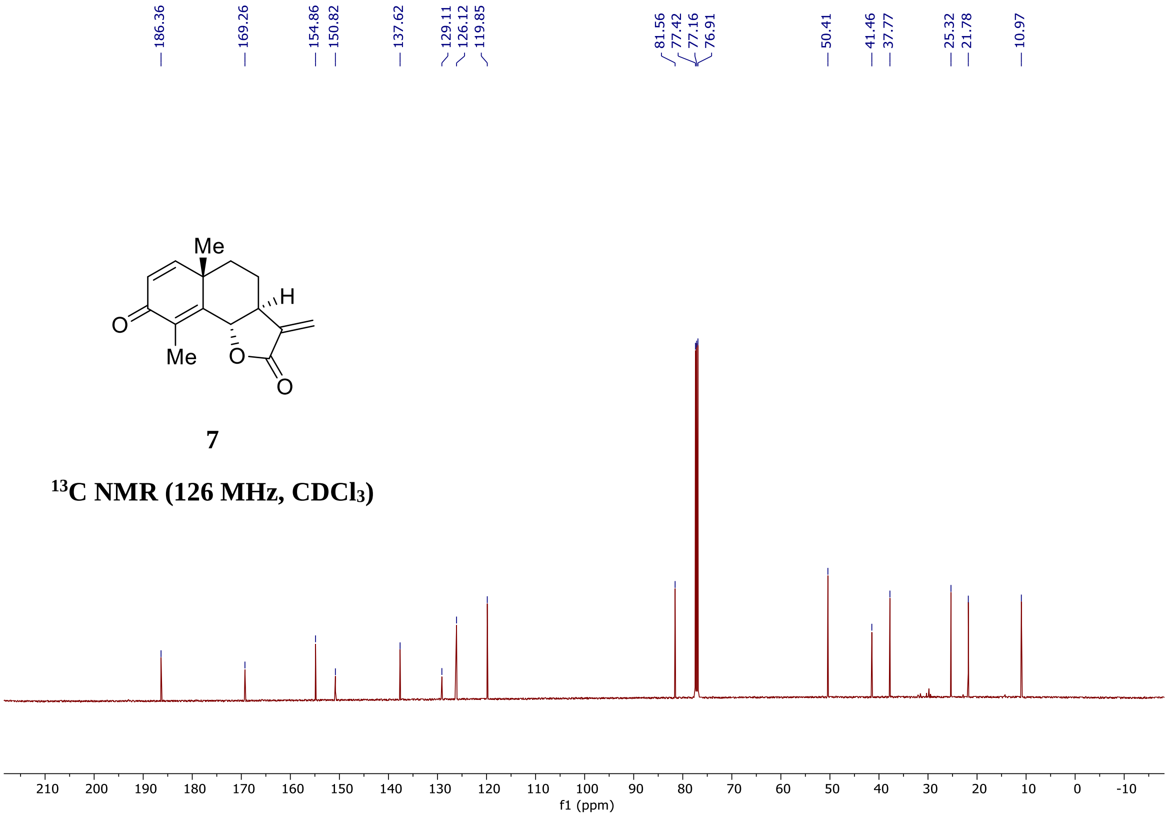

7

^{13}C NMR (126 MHz, CDCl$_3$)

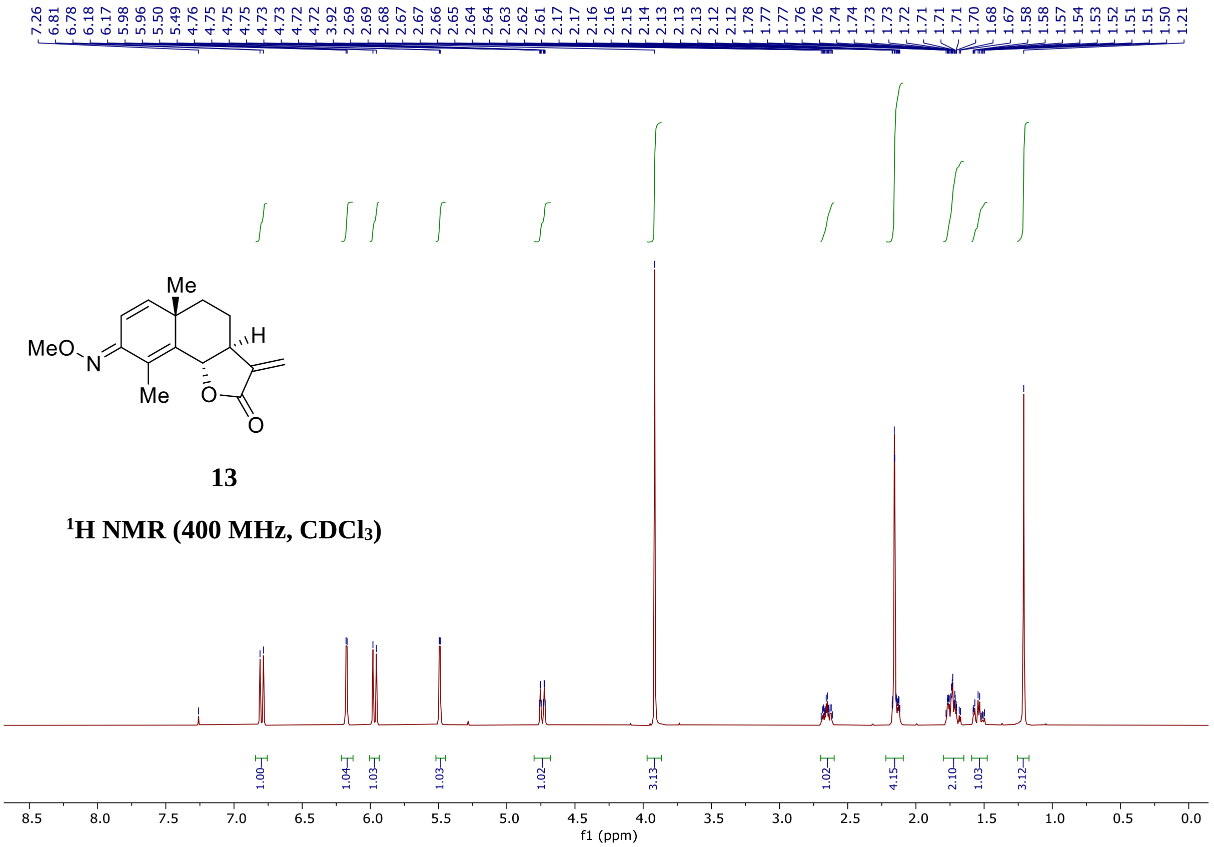

13

^{1}H NMR (400 MHz, $CDCl_3$)

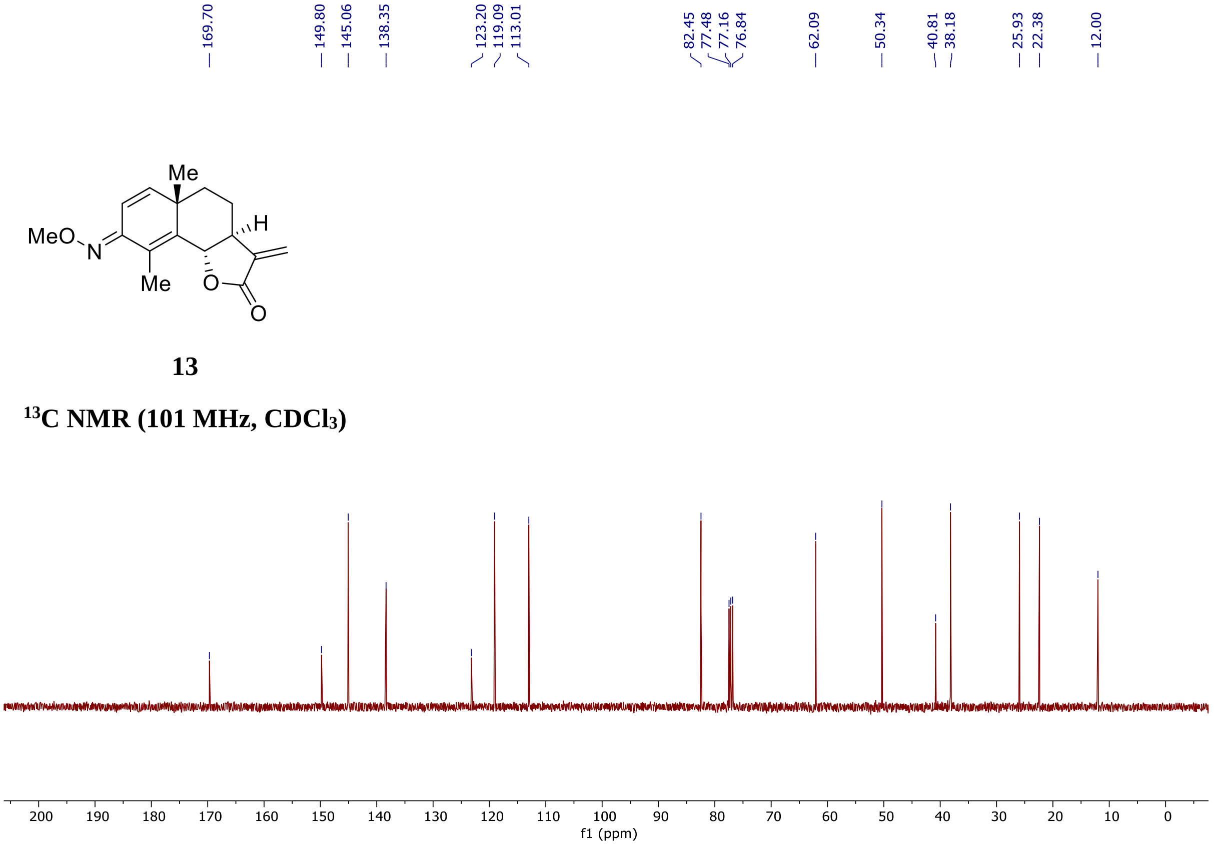

13

^{13}C NMR (101 MHz, $CDCl_3$)

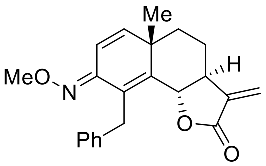

14

^{1}H NMR (400 MHz, CDCl$_3$)

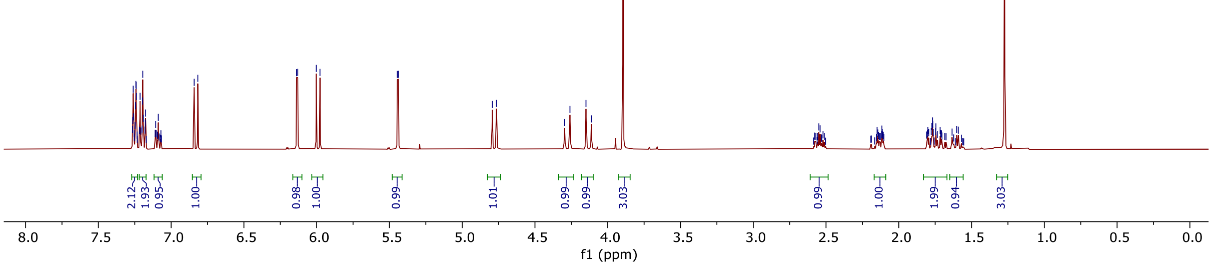

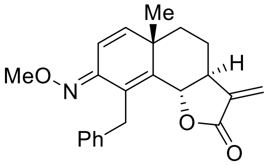

14

^{13}C NMR (101 MHz, CDCl$_3$)

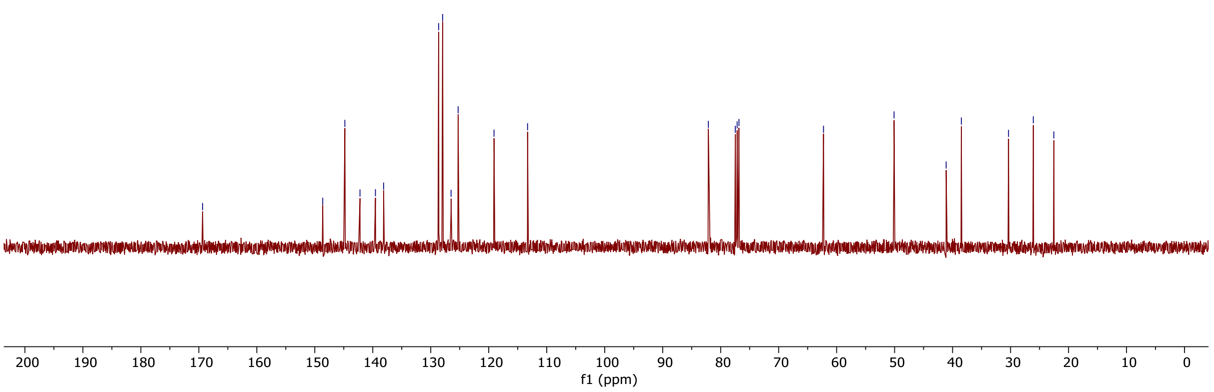

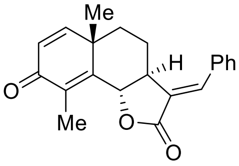

15

^{1}H NMR (400 MHz, CDCl₃)

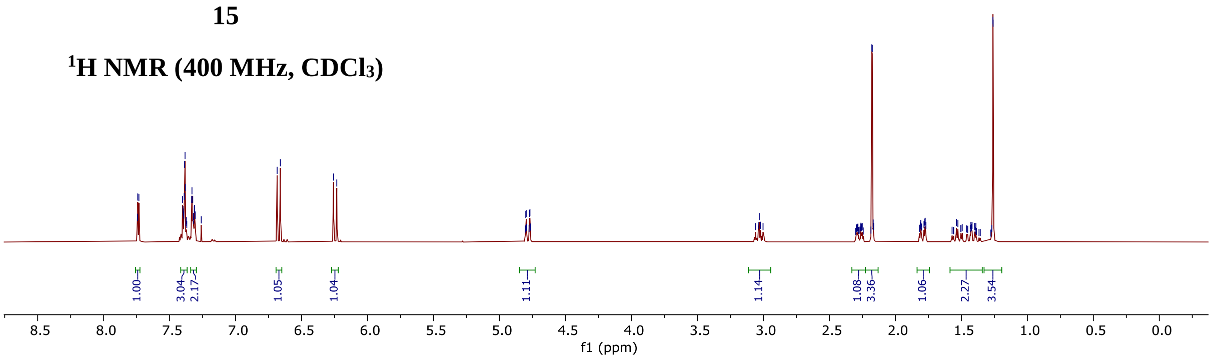

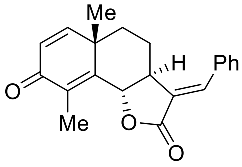

15

^{13}C NMR (101 MHz, CDCl₃)

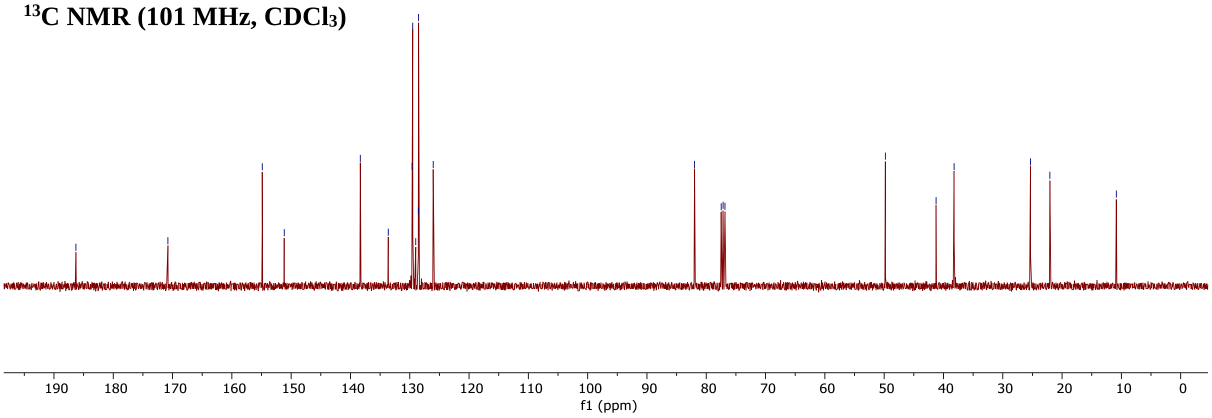

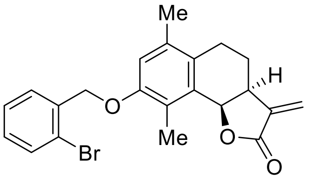

16

^{1}H NMR (400 MHz, CDCl$_3$)

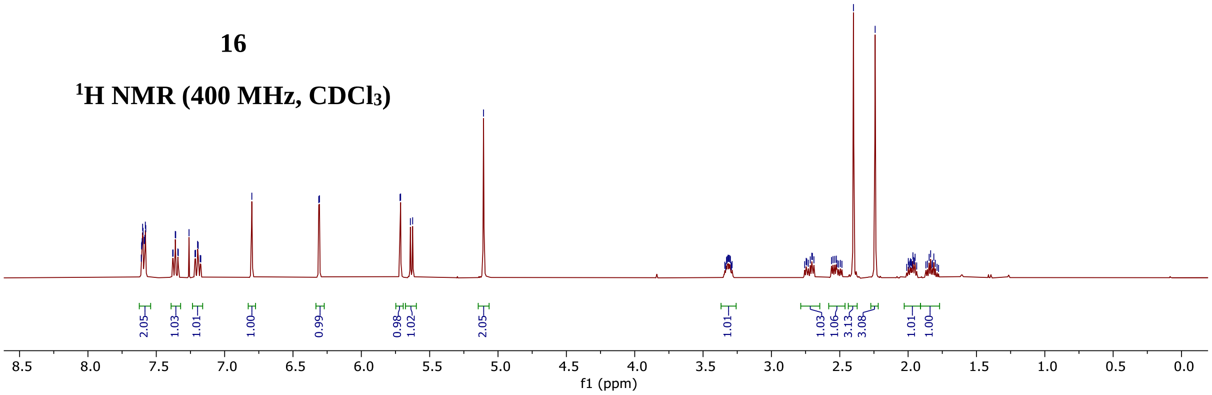

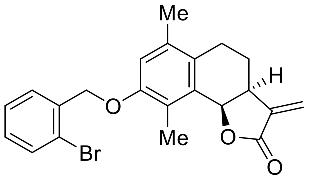

16

^{13}C NMR (101 MHz, CDCl$_3$)

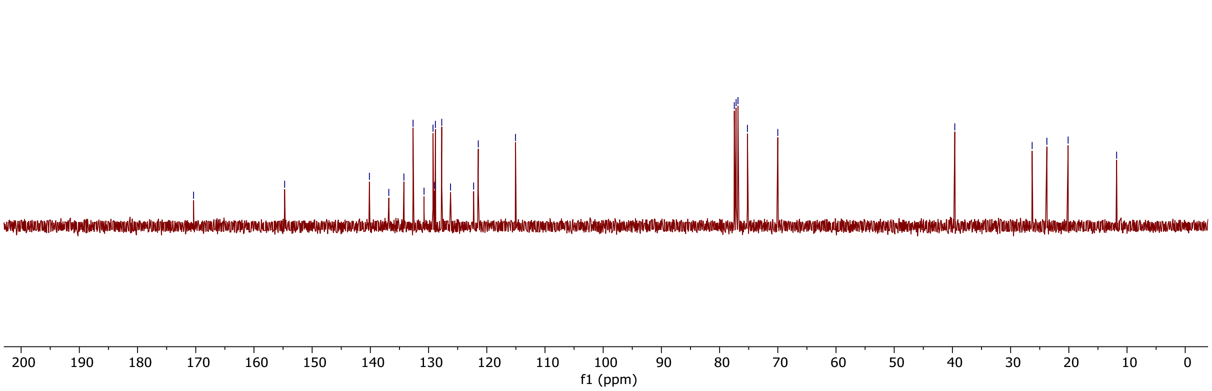

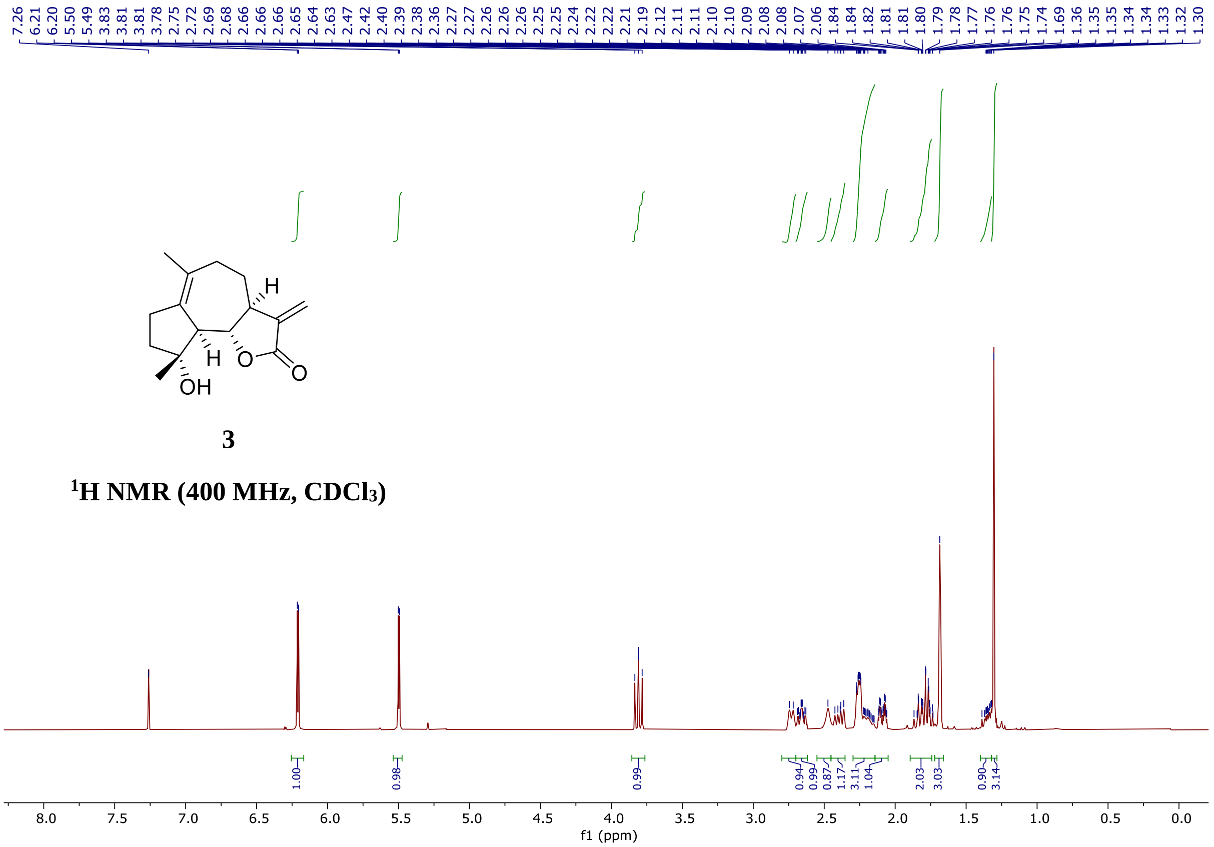

3

^{1}H NMR (400 MHz, CDCl$_3$)

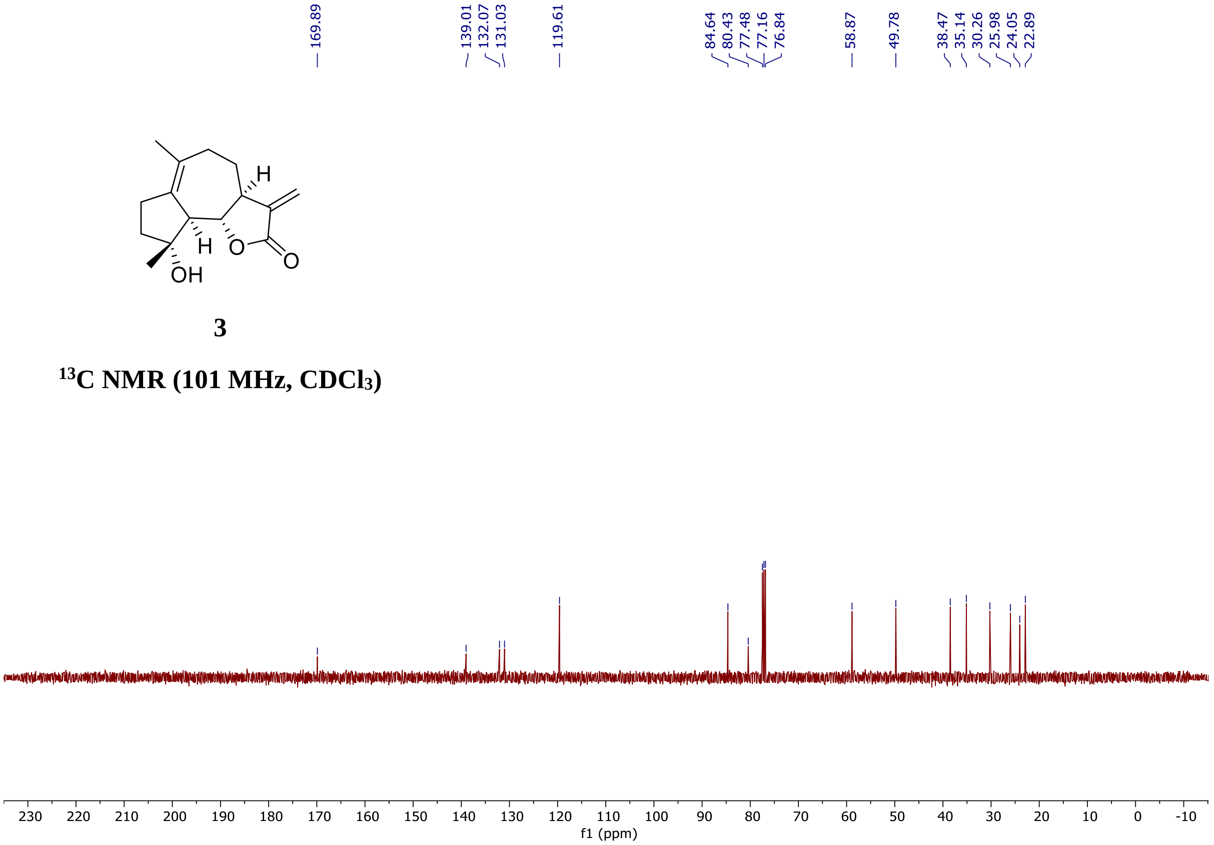

3

^{13}C NMR (101 MHz, CDCl$_3$)

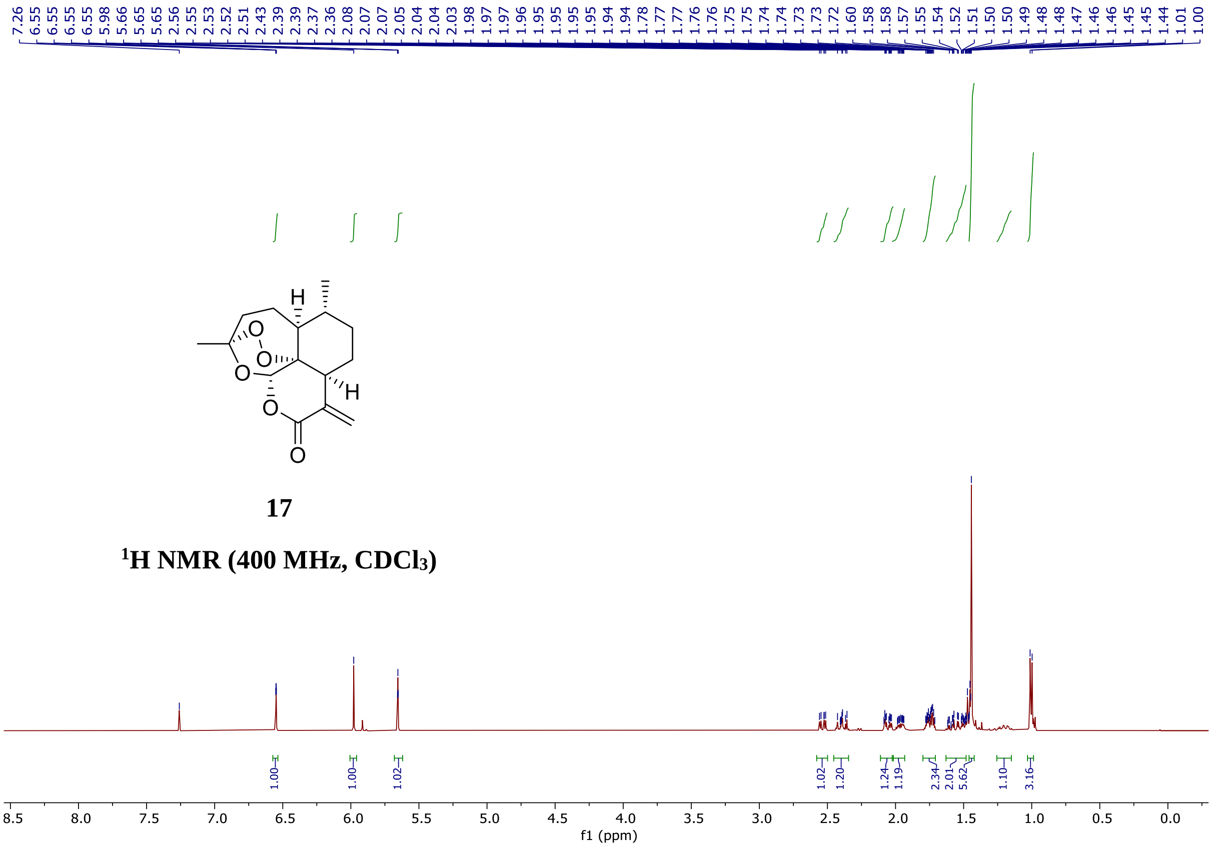

17

^{1}H NMR (400 MHz, CDCl$_3$)

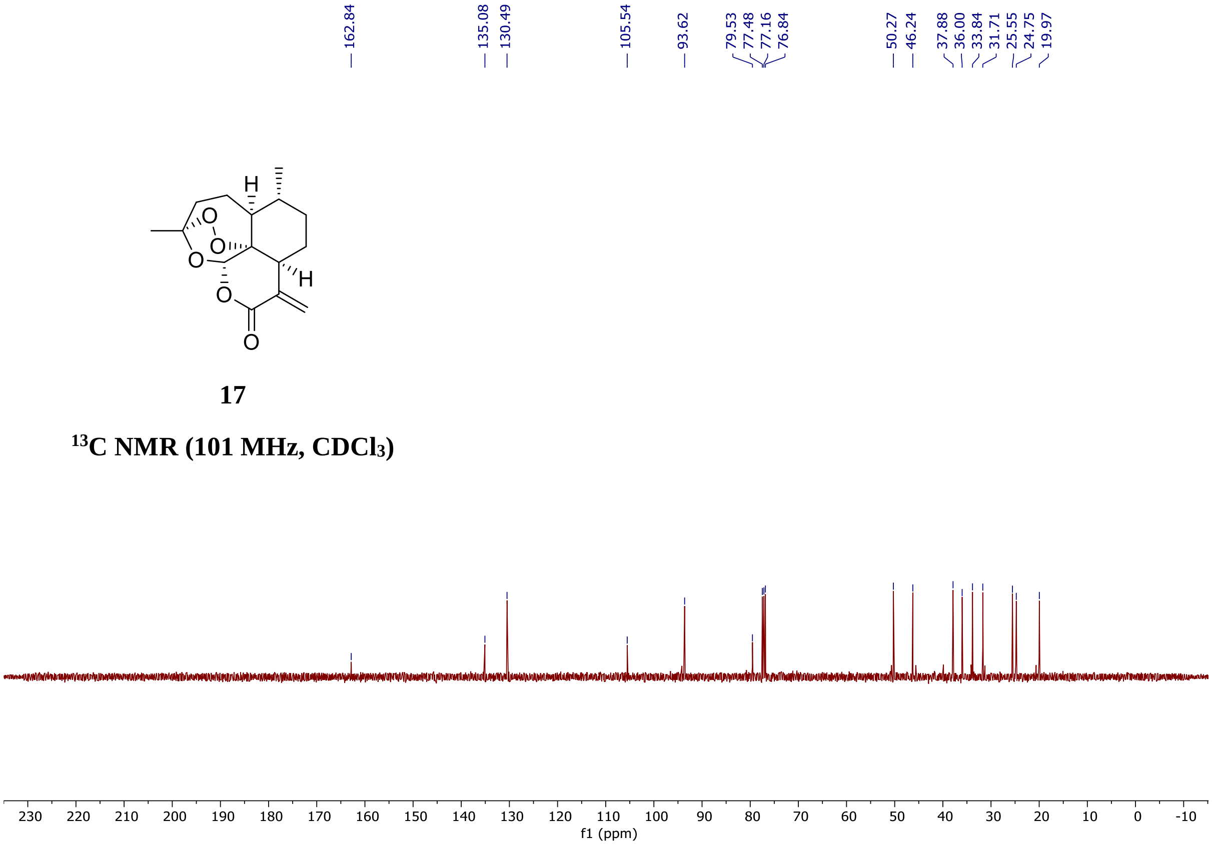

17

^{13}C NMR (101 MHz, CDCl$_3$)

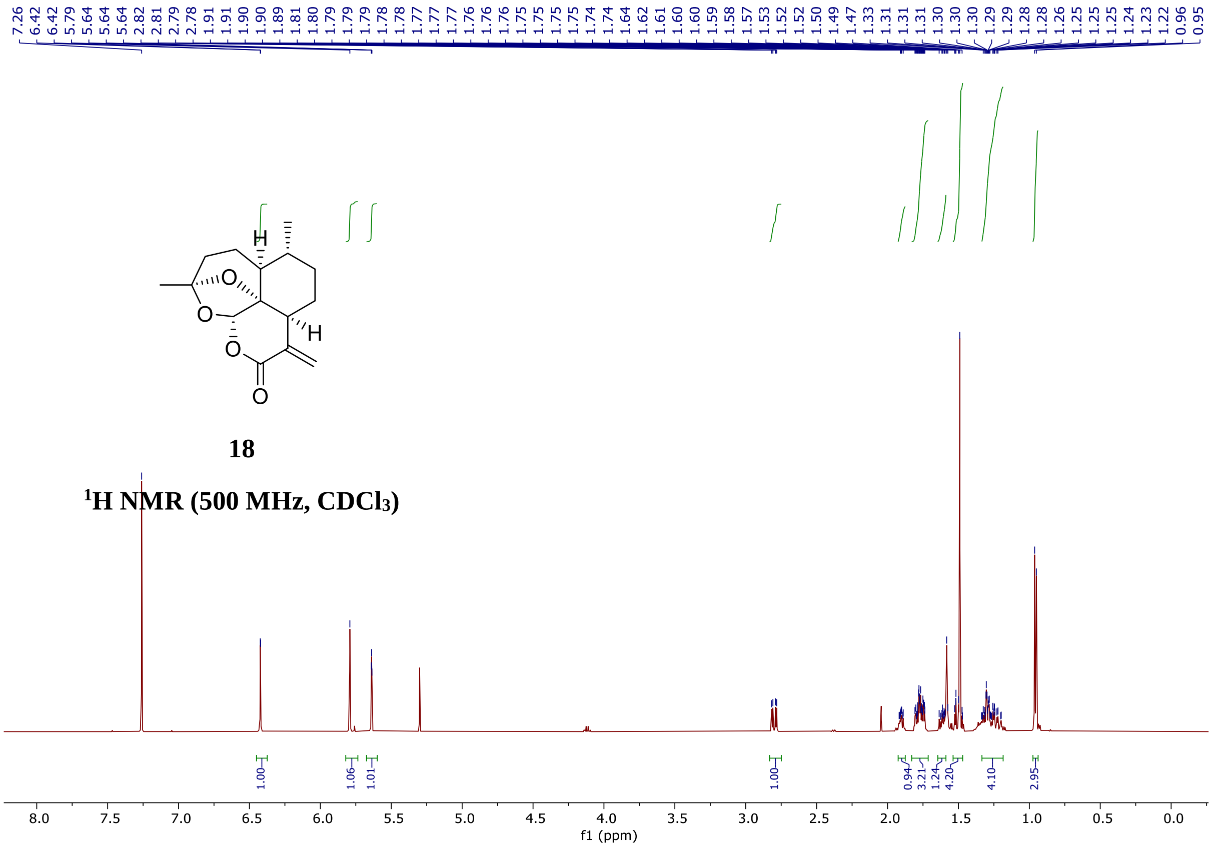
7.26 6.42 6.42 5.79 5.64 5.64 5.64 2.82 2.81 2.79 2.78 1.91 1.91 1.90 1.89 1.81 1.80 1.79 1.79 1.79 1.78 1.78 1.77 1.77 1.76 1.76 1.75 1.75 1.75 1.75 1.74 1.64 1.62 1.61 1.60 1.60 1.59 1.58 1.57 1.53 1.52 1.52 1.50 1.49 1.47 1.33 1.31 1.31 1.31 1.30 1.30 1.30 1.29 1.29 1.28 1.28 1.26 1.25 1.25 1.25 1.24 1.23 1.22 0.96 0.95
H
H
O
O
O
O
18
^{1}H NMR (500 MHz, CDCl$_3$)
1.00
1.06
1.01
1.00
0.94
3.21
1.24
4.20
4.10
2.95
8.0 7.5 7.0 6.5 6.0 5.5 5.0 4.5 4.0 3.5 3.0 2.5 2.0 1.5 1.0 0.5 0.0
f1 (ppm)

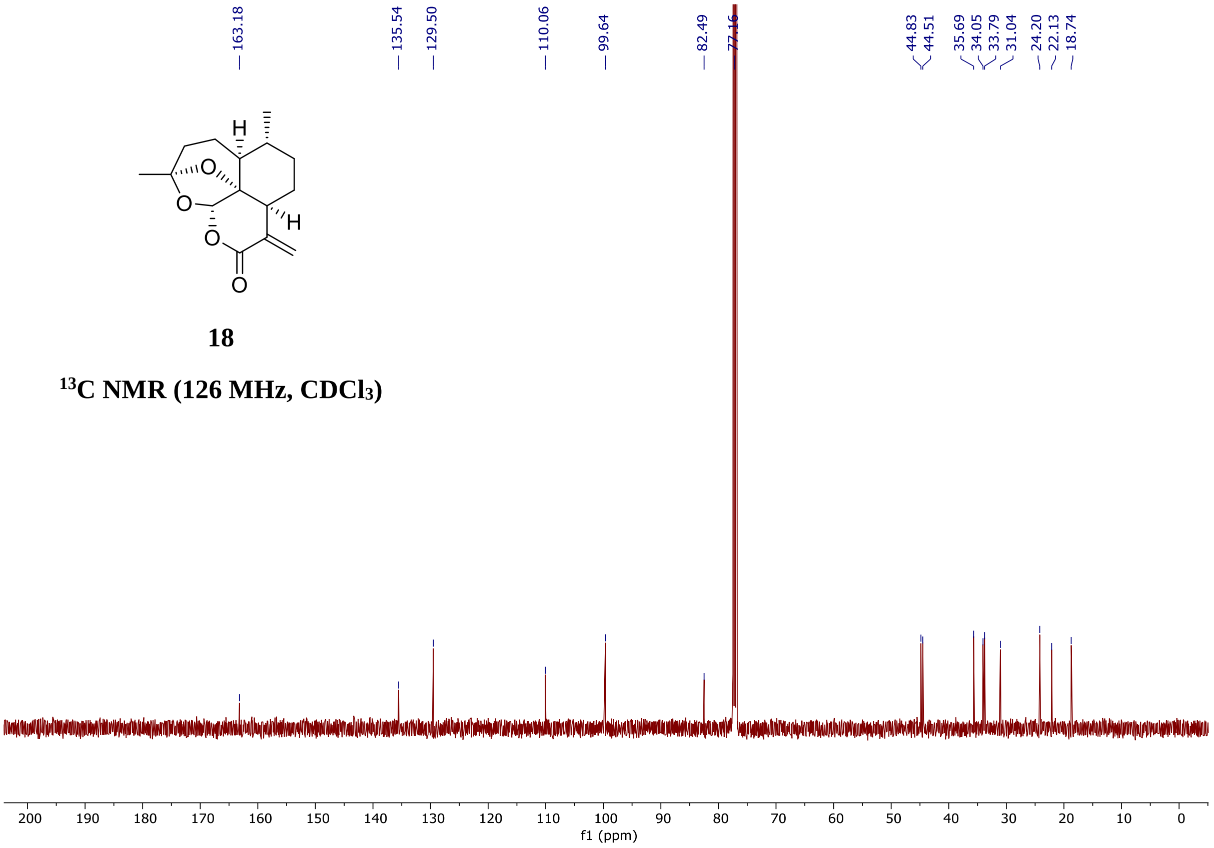
163.18
135.54
129.50
110.06
99.64
82.49
77.16
44.83
44.51
35.69
34.05
33.79
31.04
24.20
22.13
18.74
H
H
O
O
O
O
18
^{13}C NMR (126 MHz, CDCl$_3$)
200 190 180 170 160 150 140 130 120 110 100 90 80 70 60 50 40 30 20 10 0
f1 (ppm)

7.3 NMR spectra of diverse sesquiterpenoid alkaloids (**20-47**)

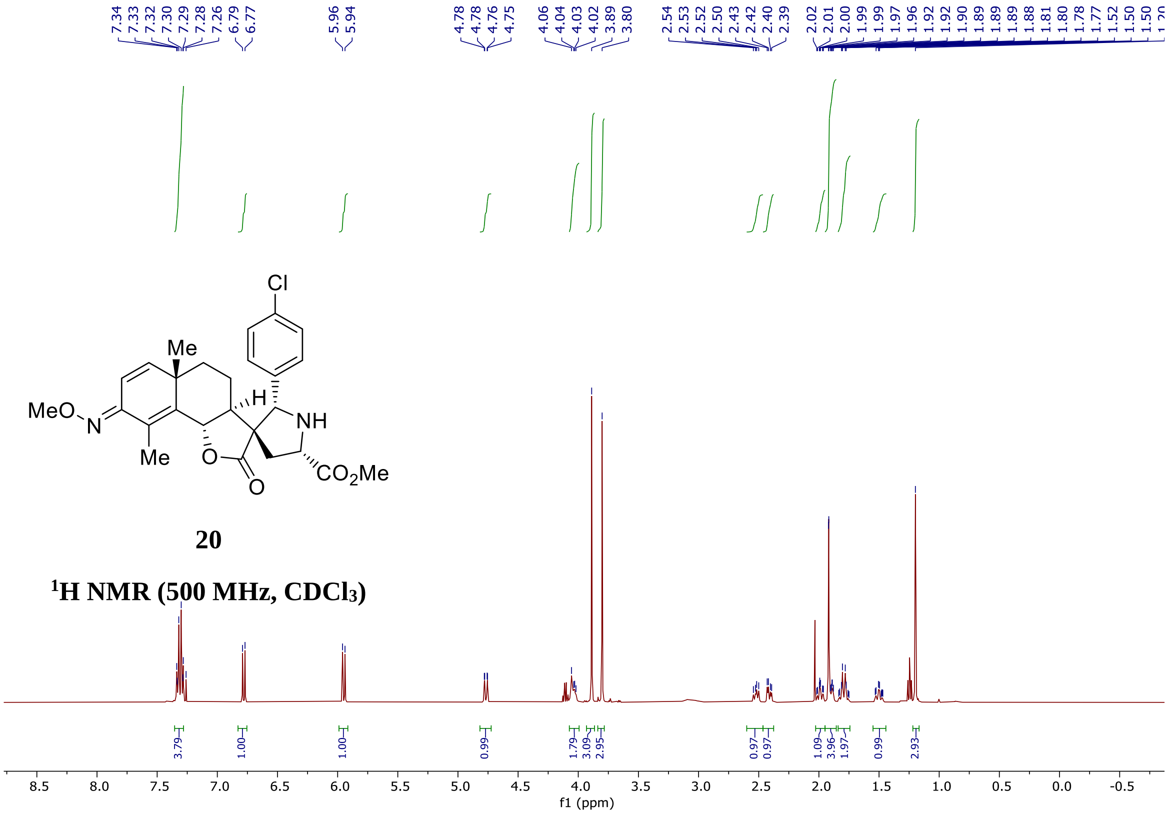

20

^{1}H NMR (500 MHz, CDCl₃)

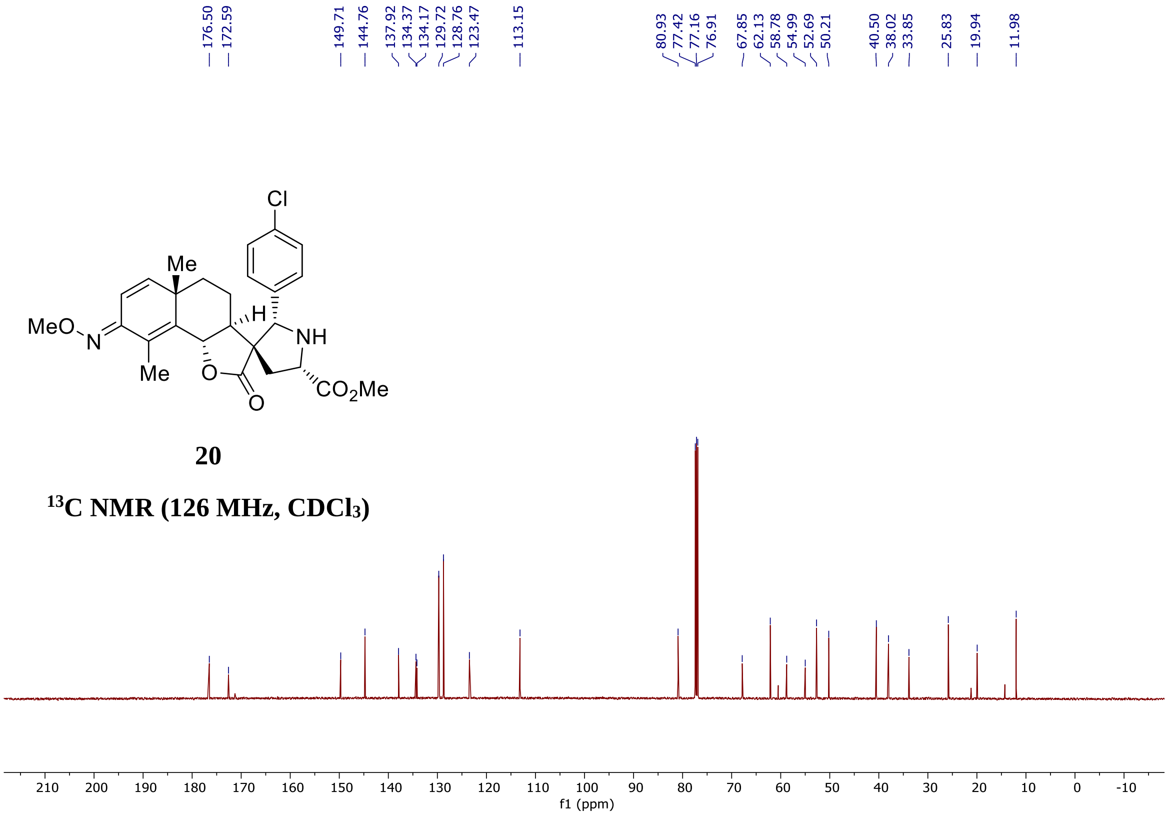

20

^{13}C NMR (126 MHz, CDCl₃)

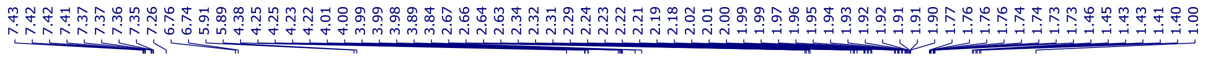

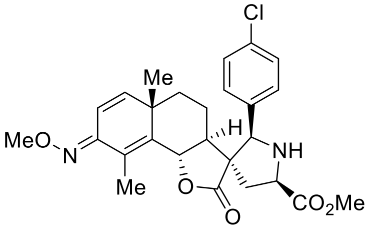

21

^{1}H NMR (500 MHz, $CDCl_3$)

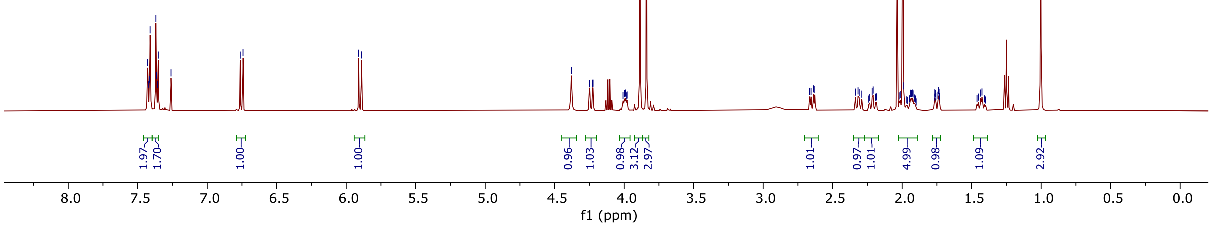

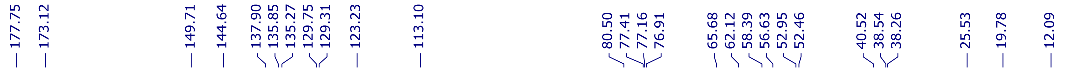

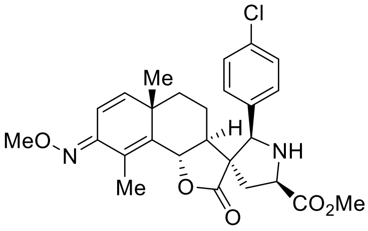

21

^{13}C NMR (126 MHz, $CDCl_3$)

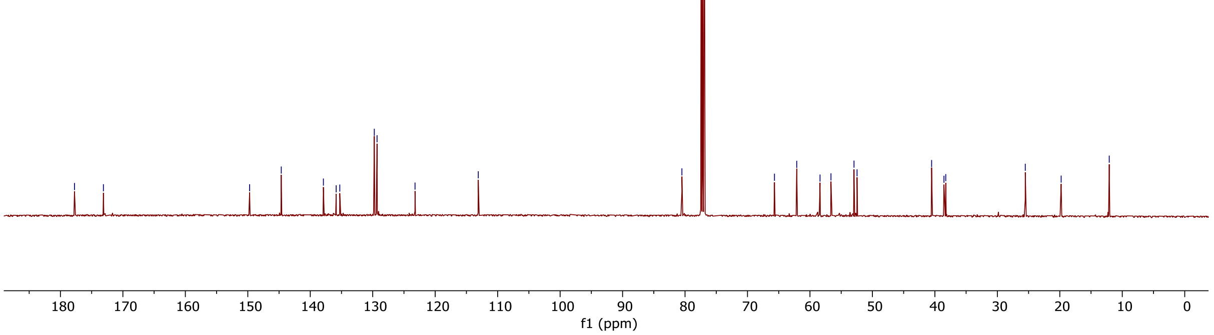

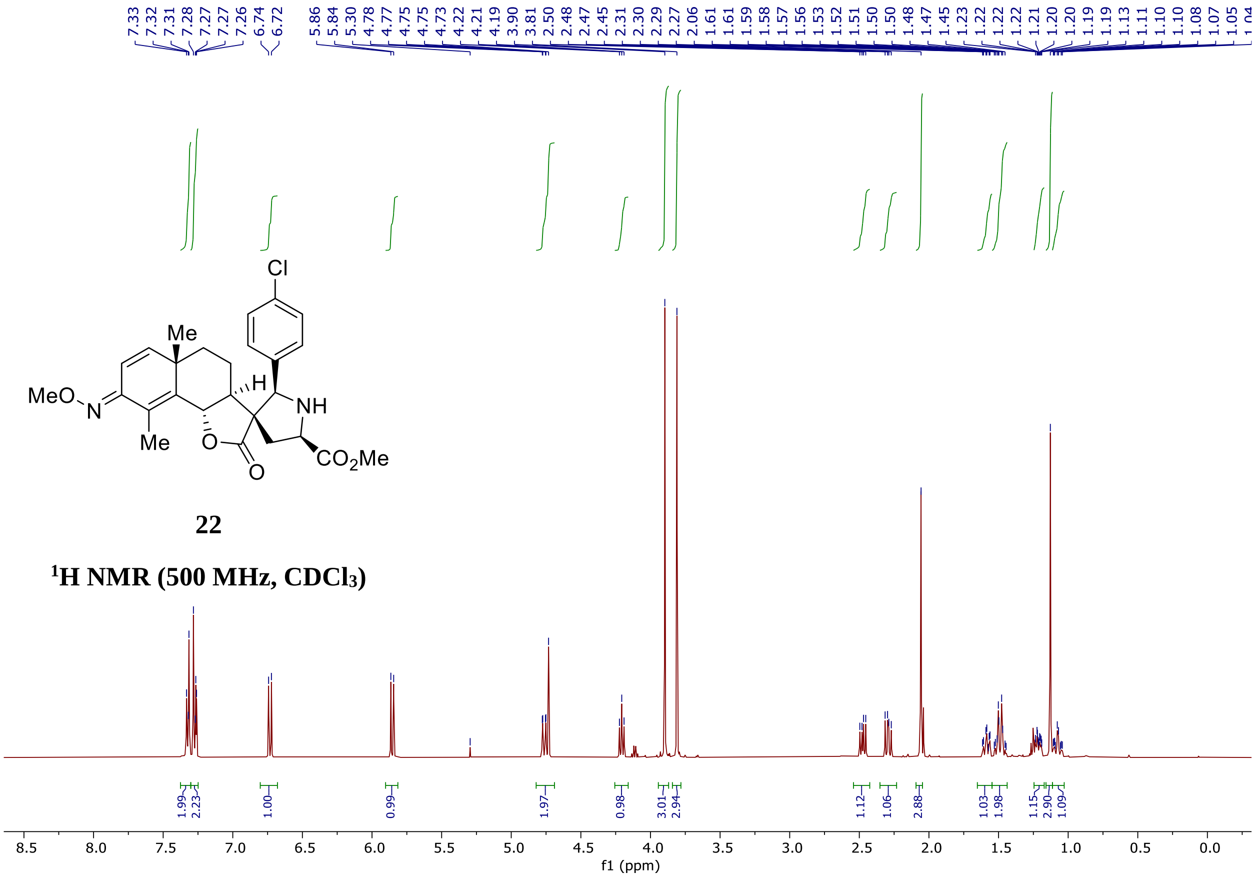

22

^{1}H NMR (500 MHz, CDCl$_3$)

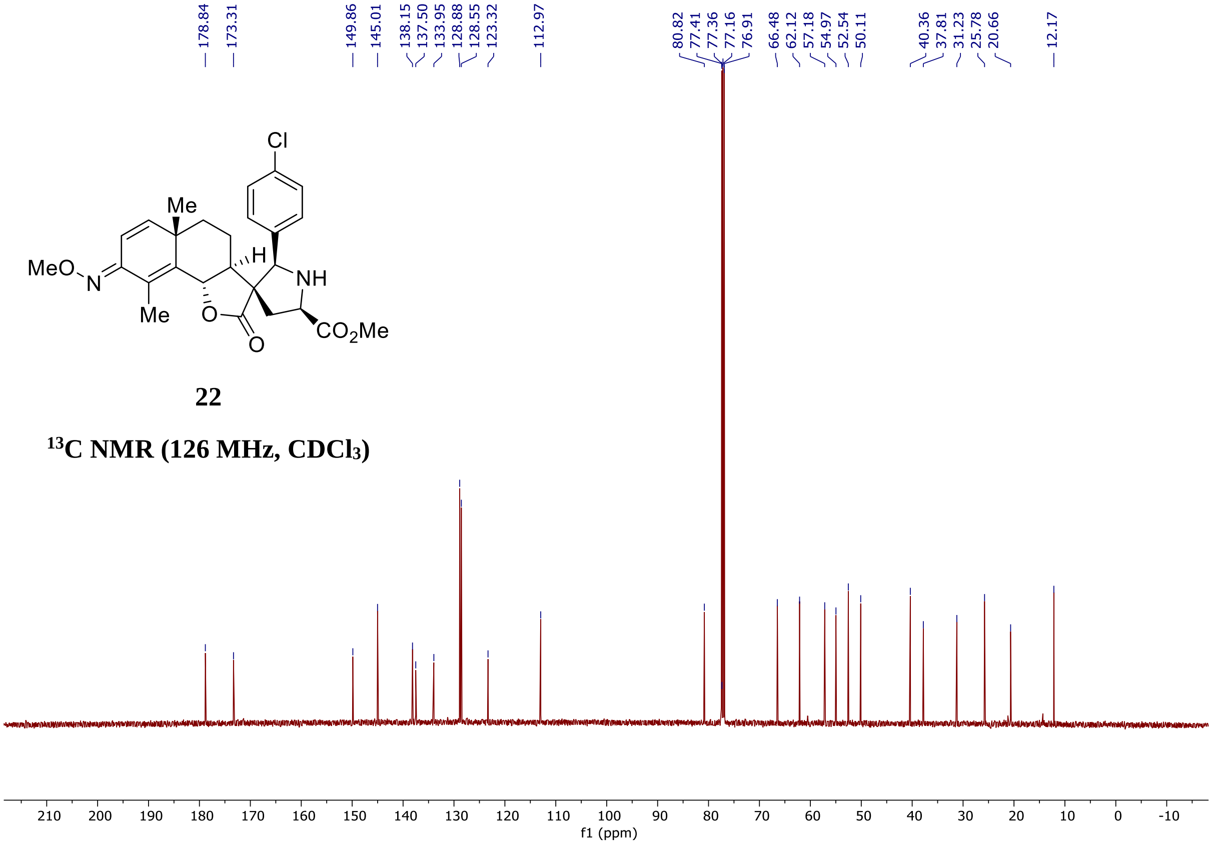

22

^{13}C NMR (126 MHz, CDCl$_3$)

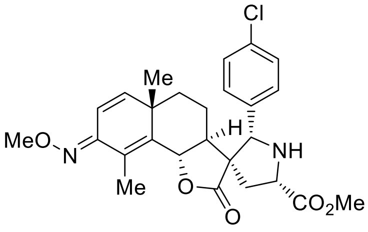

23

^{1}H NMR (500 MHz, CDCl$_3$)

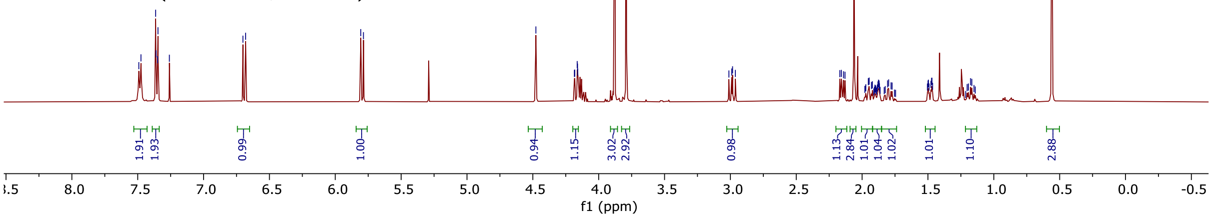

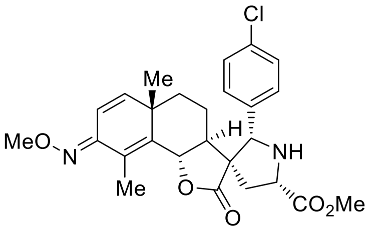

23

^{13}C NMR (126 MHz, CDCl$_3$)

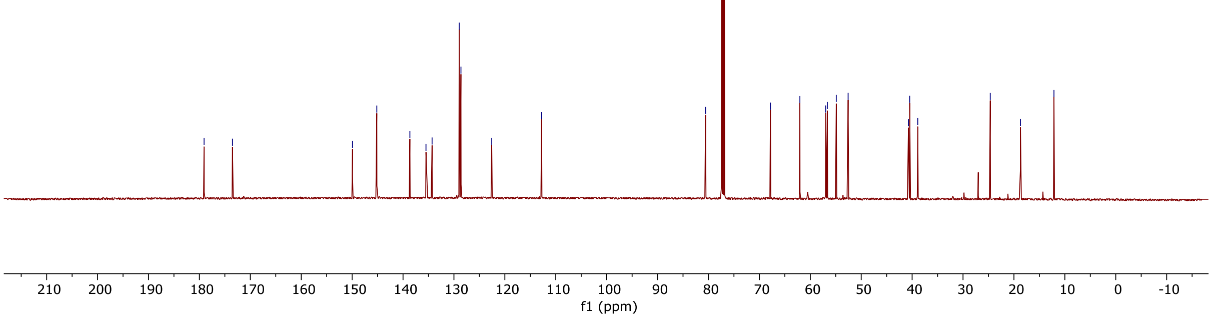

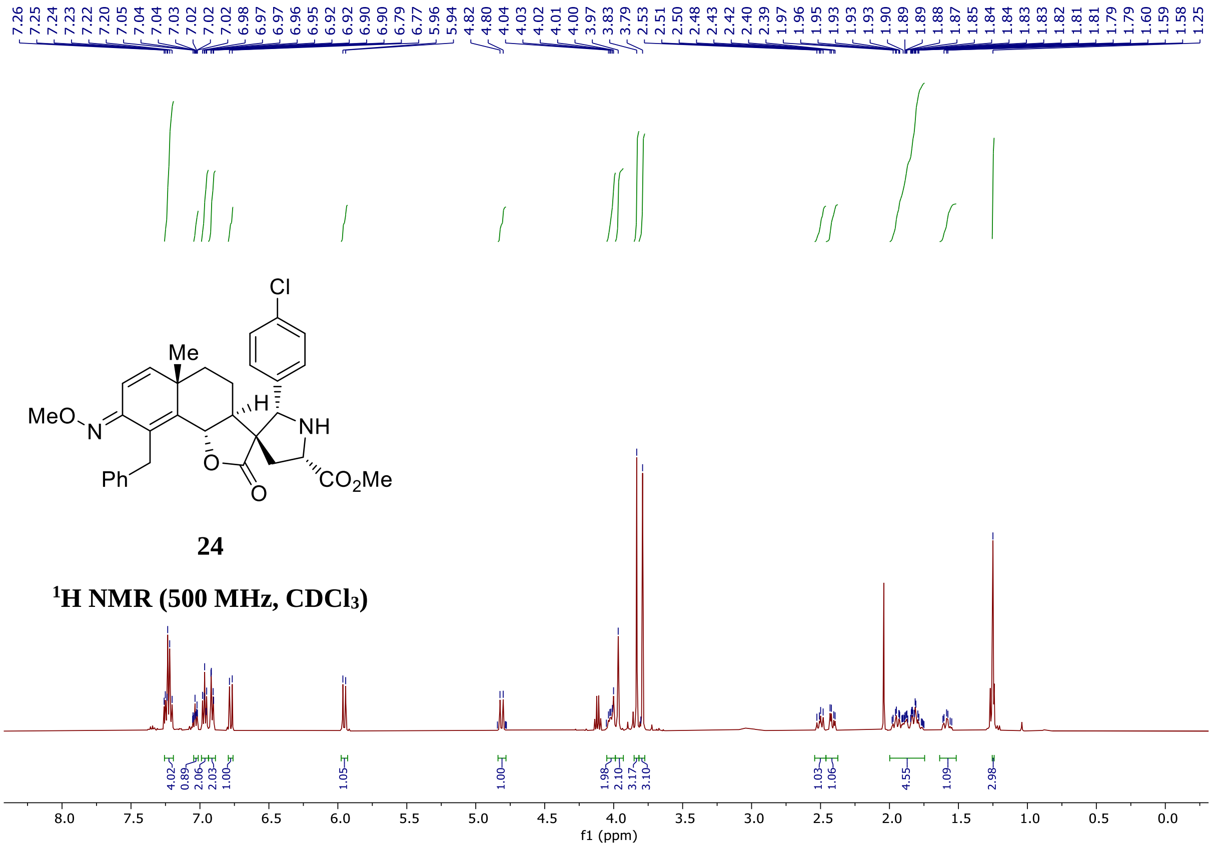

24

^{1}H NMR (500 MHz, $CDCl_3$)

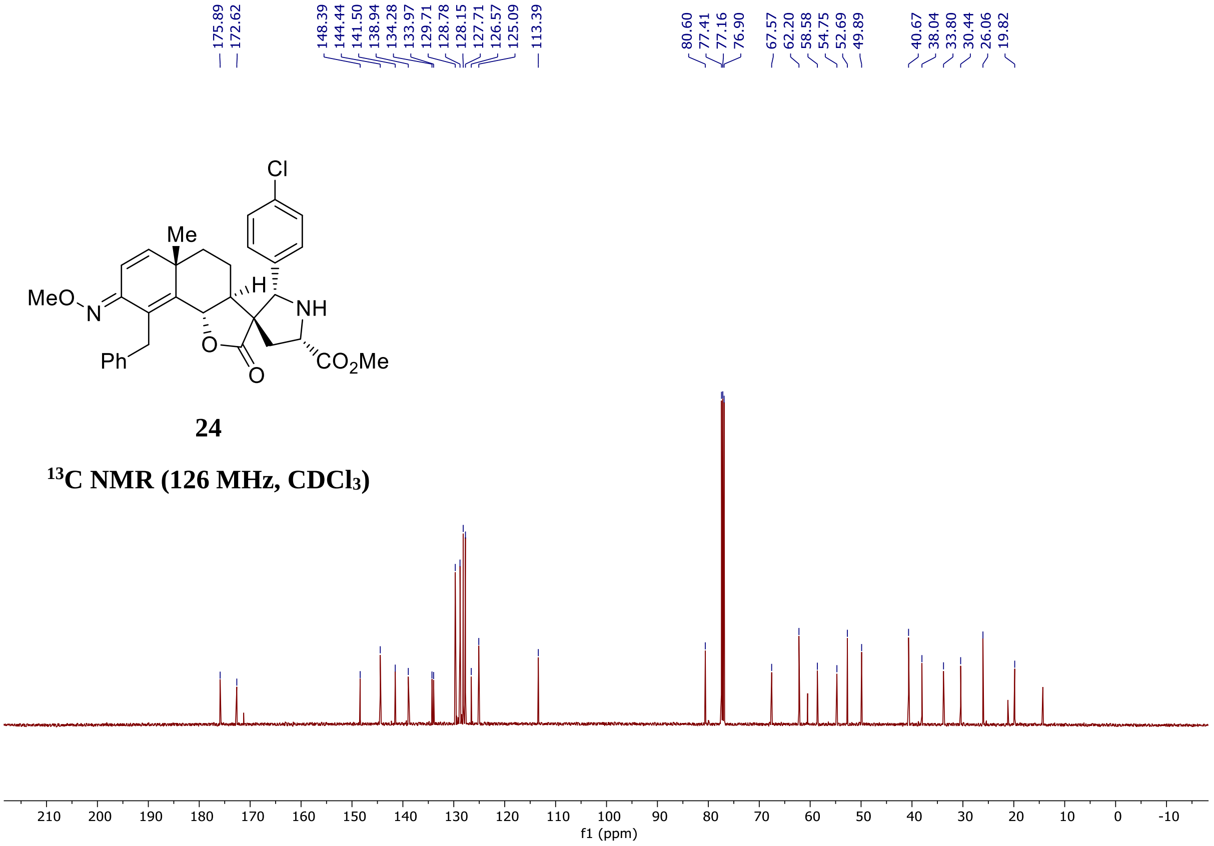

24

^{13}C NMR (126 MHz, $CDCl_3$)

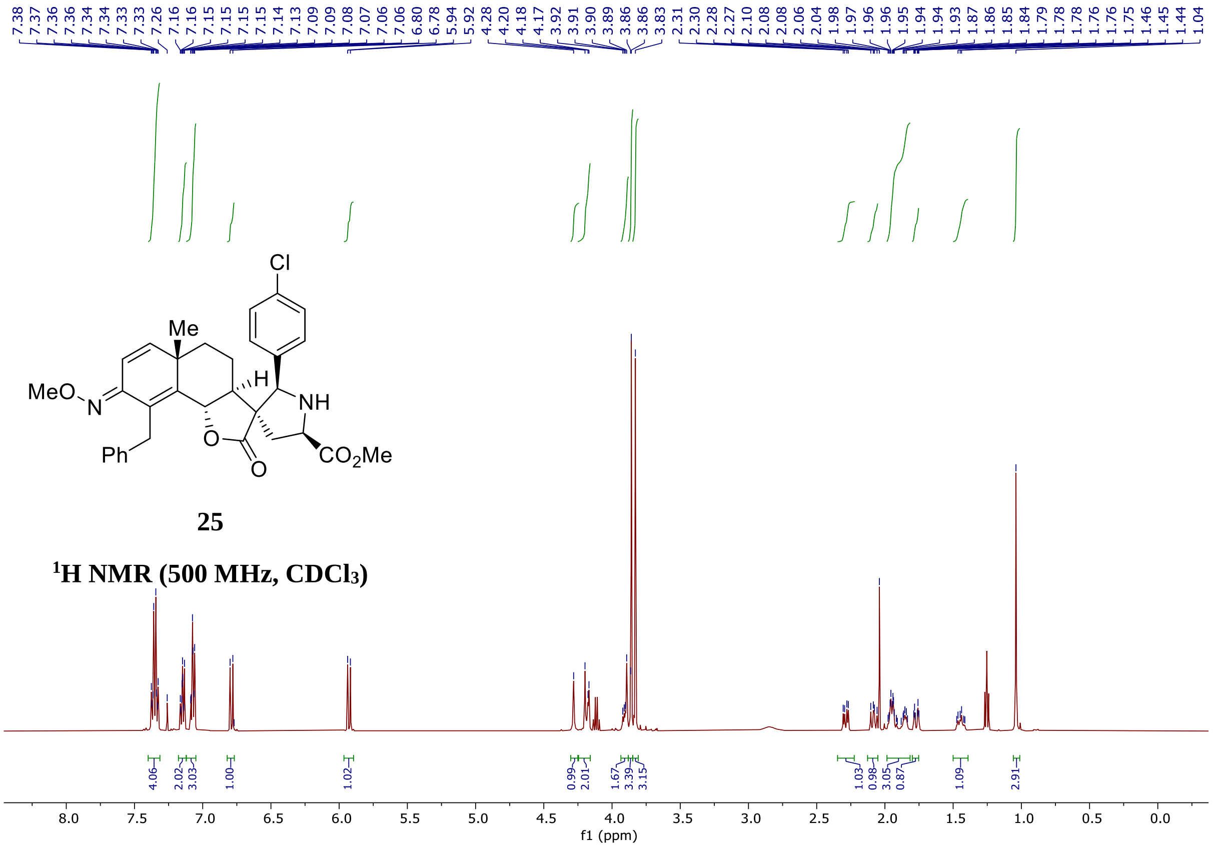

25

^{1}H NMR (500 MHz, CDCl3)

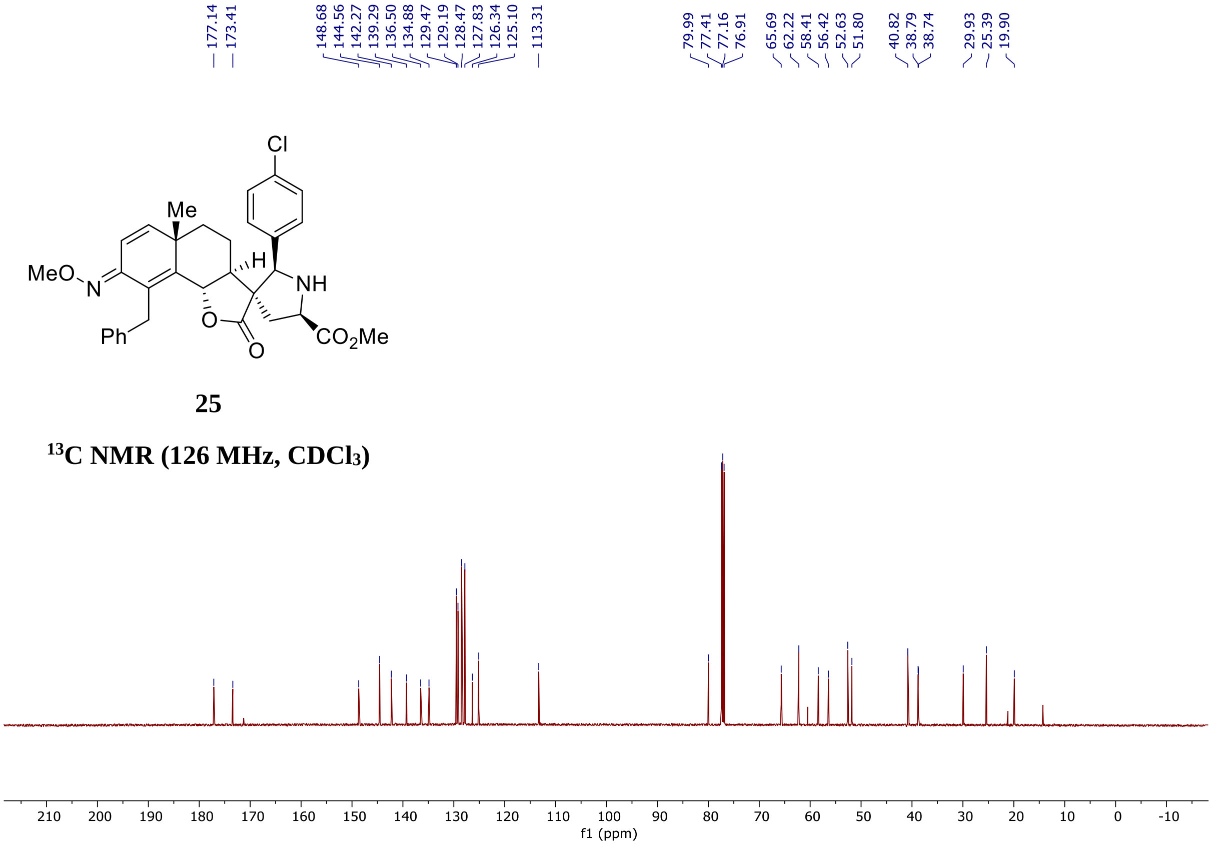

25

^{13}C NMR (126 MHz, CDCl3)

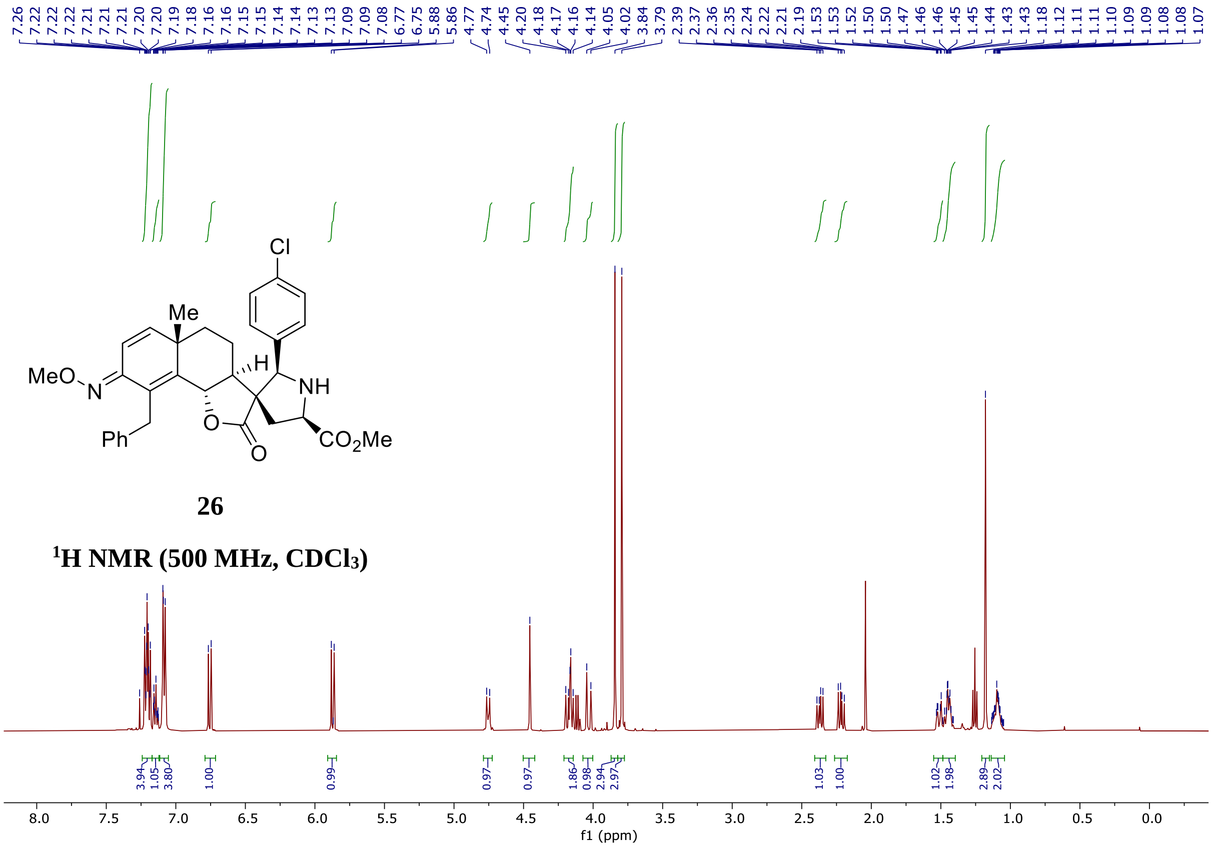
7.26 7.22 7.22 7.22 7.21 7.21 7.21 7.20 7.20 7.19 7.18 7.16 7.16 7.15 7.15 7.14 7.14 7.13 7.13 7.09 7.09 7.08 6.77 6.75 5.88 5.86 4.77 4.74 4.45 4.20 4.18 4.17 4.16 4.14 4.05 4.02 3.84 3.79 2.39 2.37 2.36 2.35 2.24 2.22 2.21 2.19 1.53 1.53 1.52 1.50 1.50 1.47 1.46 1.46 1.45 1.45 1.44 1.43 1.43 1.18 1.12 1.11 1.11 1.10 1.09 1.09 1.08 1.08 1.07
Cl
Me
MeO
N
H
NH
O
O
Ph
CO_2Me
26
^{1}H NMR (500 MHz, $CDCl_3$)
3.94 1.05 3.80 1.00 0.99 0.97 0.97 1.86 0.98 2.94 2.97 1.03 1.00 1.02 1.98 2.89 2.02
8.0 7.5 7.0 6.5 6.0 5.5 5.0 4.5 4.0 3.5 3.0 2.5 2.0 1.5 1.0 0.5 0.0
f1 (ppm)

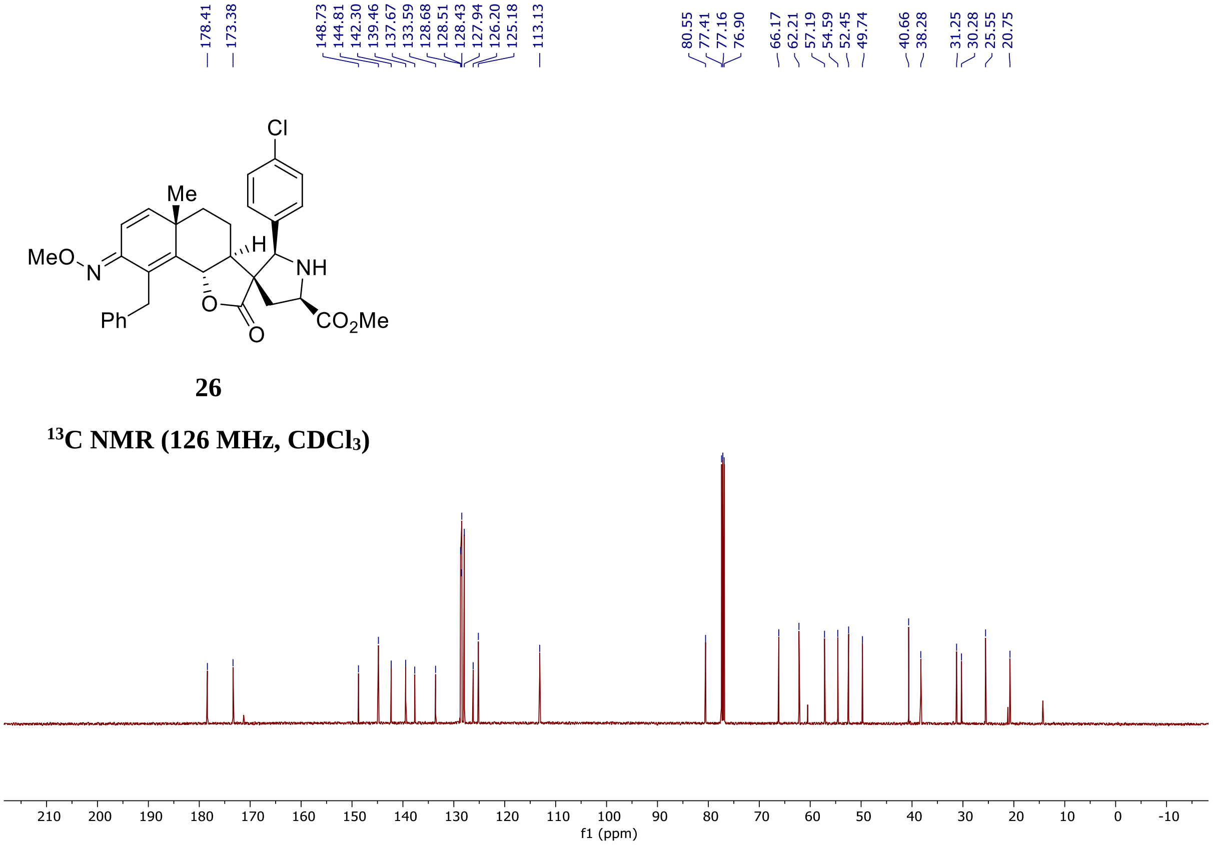
178.41 173.38 148.73 144.81 142.30 139.46 137.67 133.59 128.68 128.51 128.43 127.94 126.20 125.18 113.13 80.55 77.41 77.16 76.90 66.17 62.21 57.19 54.59 52.45 49.74 40.66 38.28 31.25 30.28 25.55 20.75
Cl
Me
MeO
N
H
NH
O
O
Ph
CO_2Me
26
^{13}C NMR (126 MHz, $CDCl_3$)
210 200 190 180 170 160 150 140 130 120 110 100 90 80 70 60 50 40 30 20 10 0 -10
f1 (ppm)

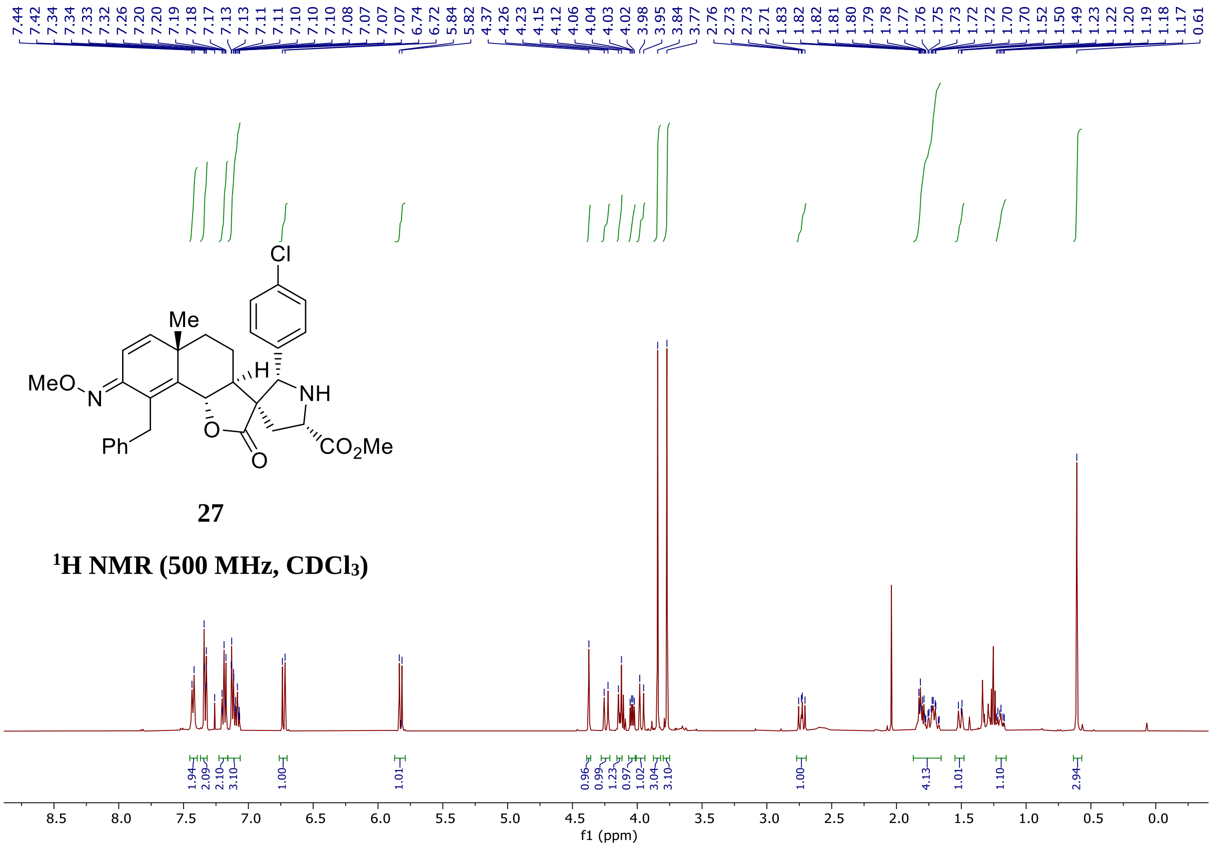

27

^{1}H NMR (500 MHz, $CDCl_3$)

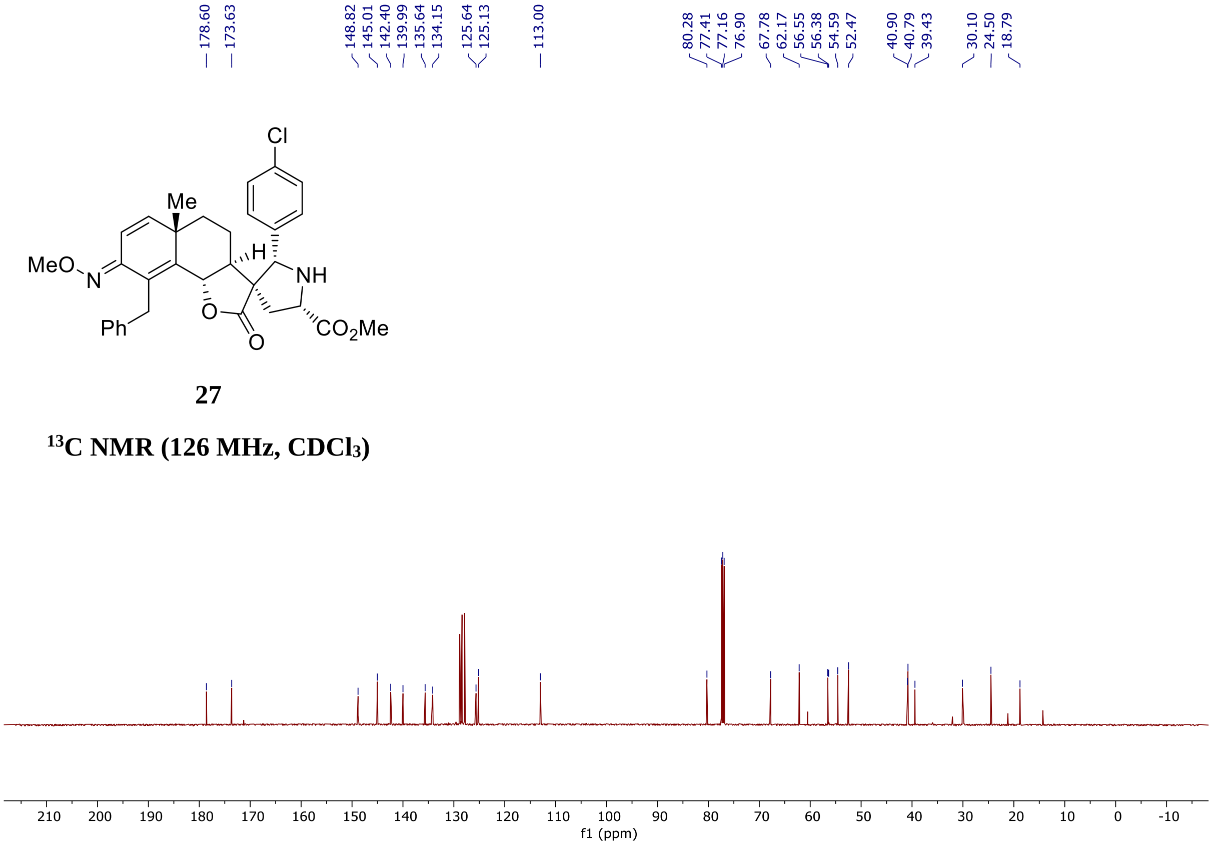

27

^{13}C NMR (126 MHz, $CDCl_3$)

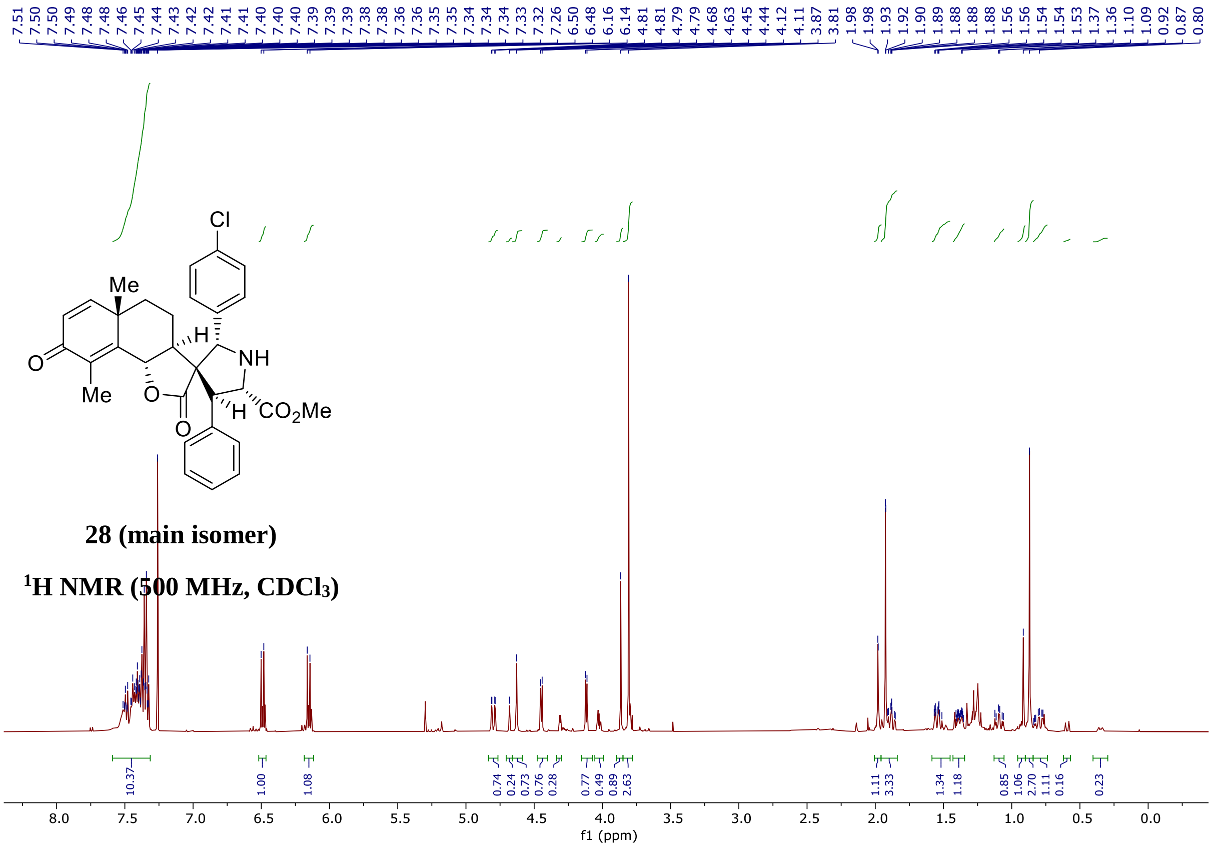

28 (main isomer)

^{1}H NMR (500 MHz, $CDCl_3$)

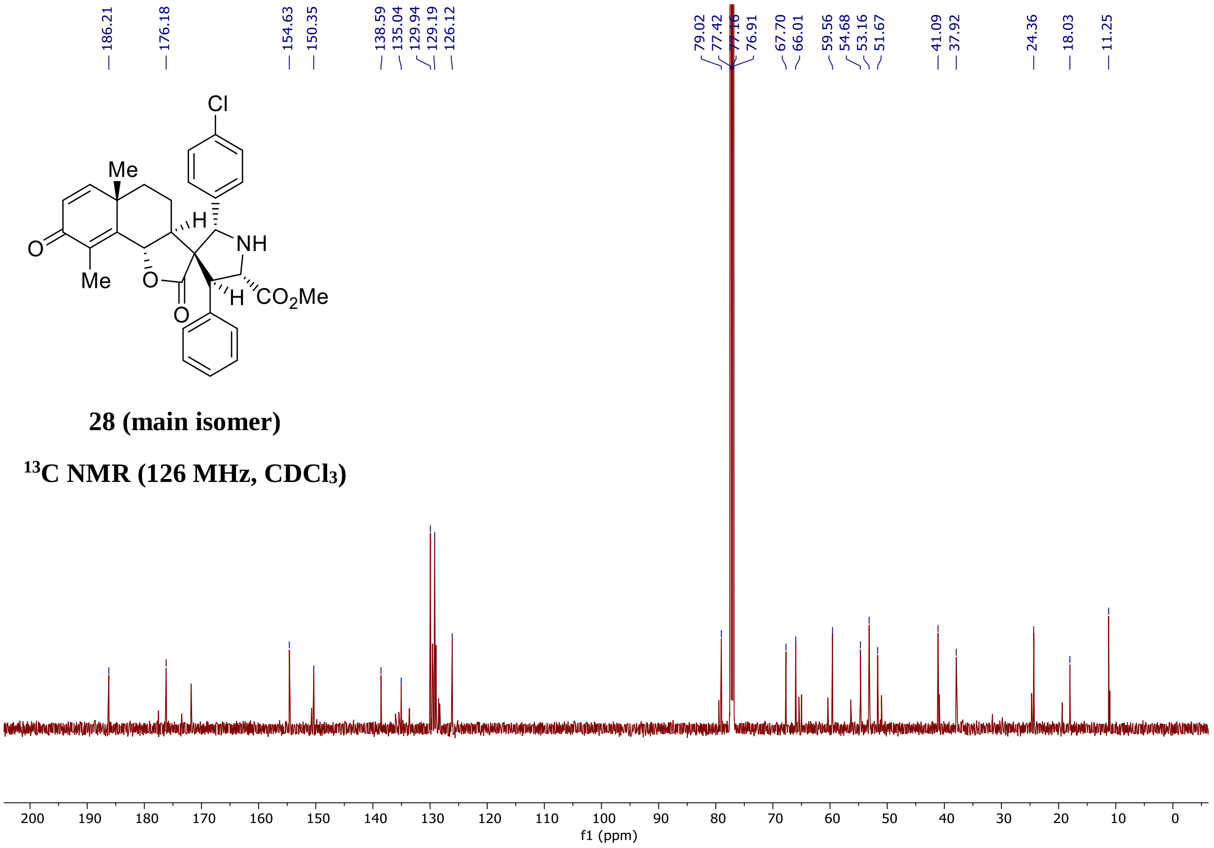

28 (main isomer)

^{13}C NMR (126 MHz, $CDCl_3$)

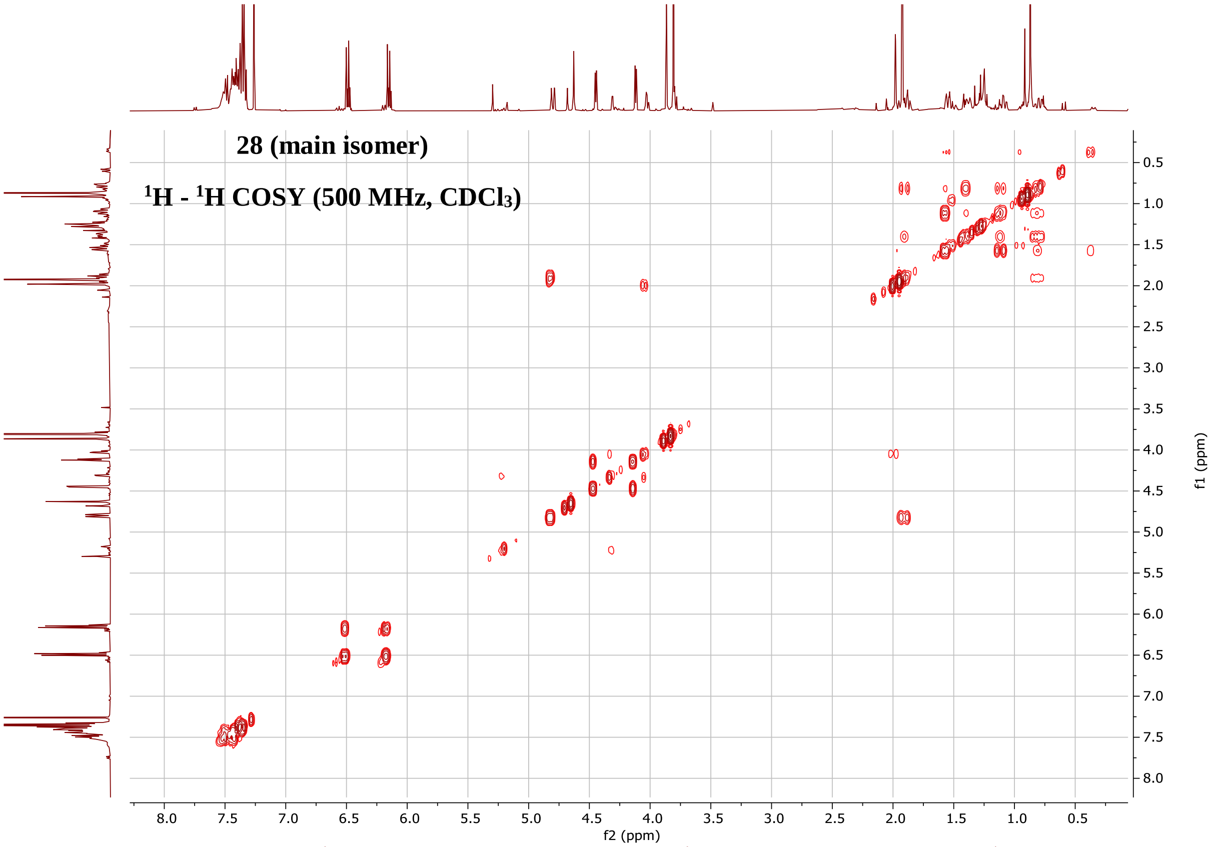
28 (main isomer)
1H - 1H COSY (500 MHz, CDCl3)
f1 (ppm)
f2 (ppm)

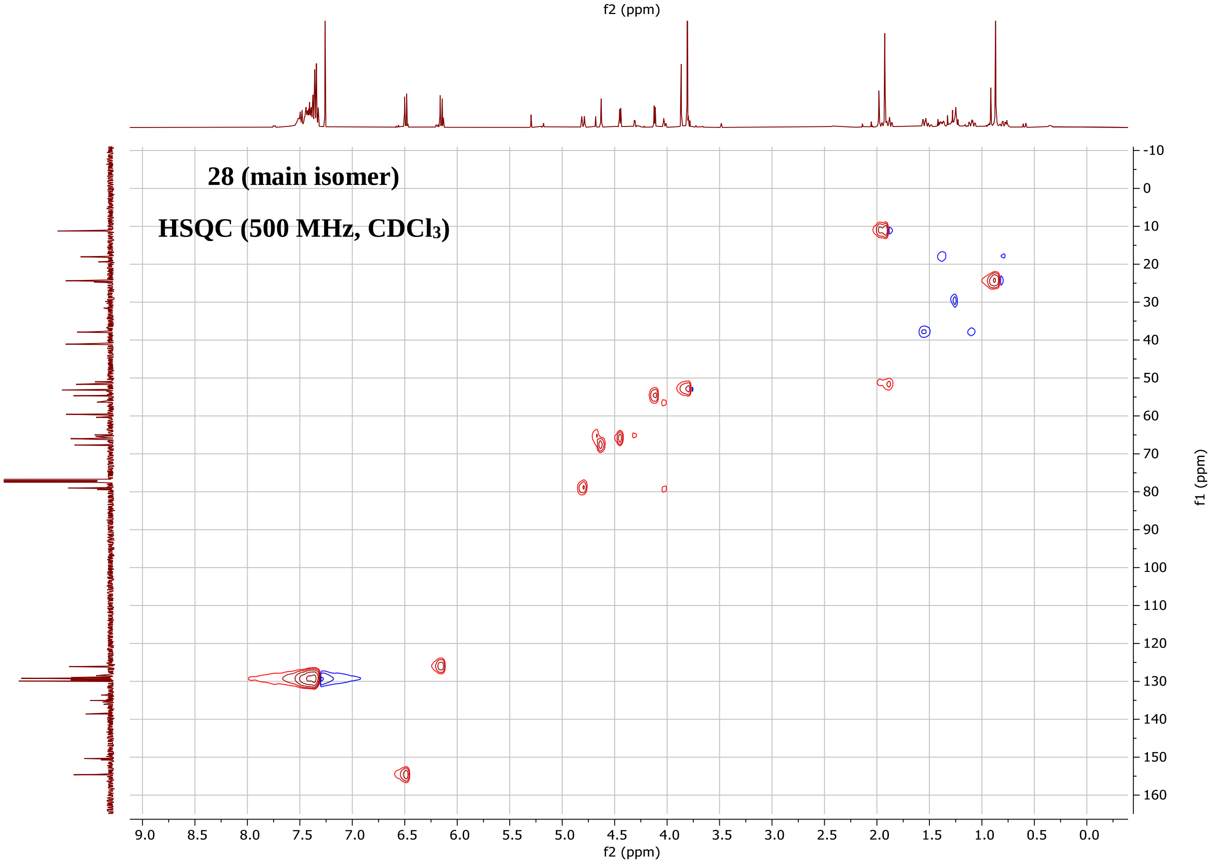
28 (main isomer)
HSQC (500 MHz, CDCl3)
f1 (ppm)
f2 (ppm)

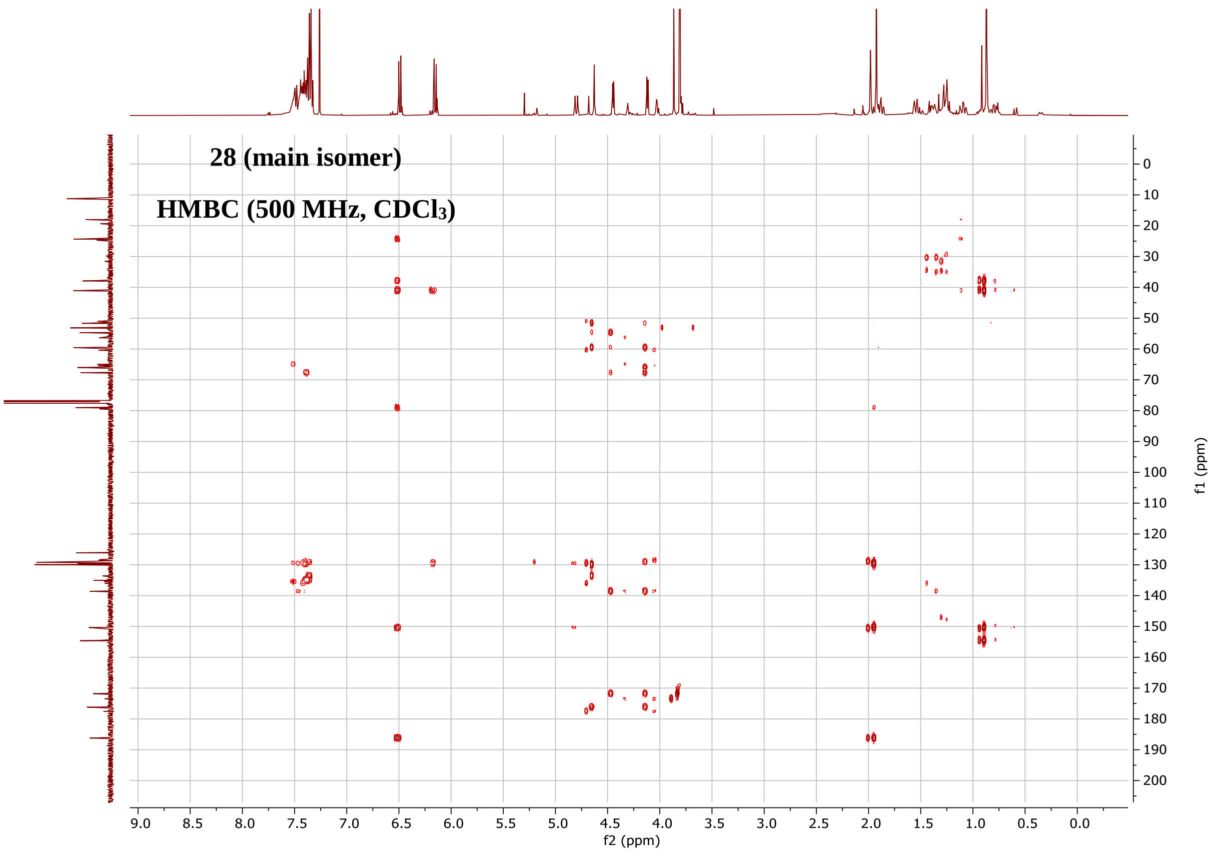
28 (main isomer)
HMBC (500 MHz, CDCl3)
f1 (ppm)
f2 (ppm)

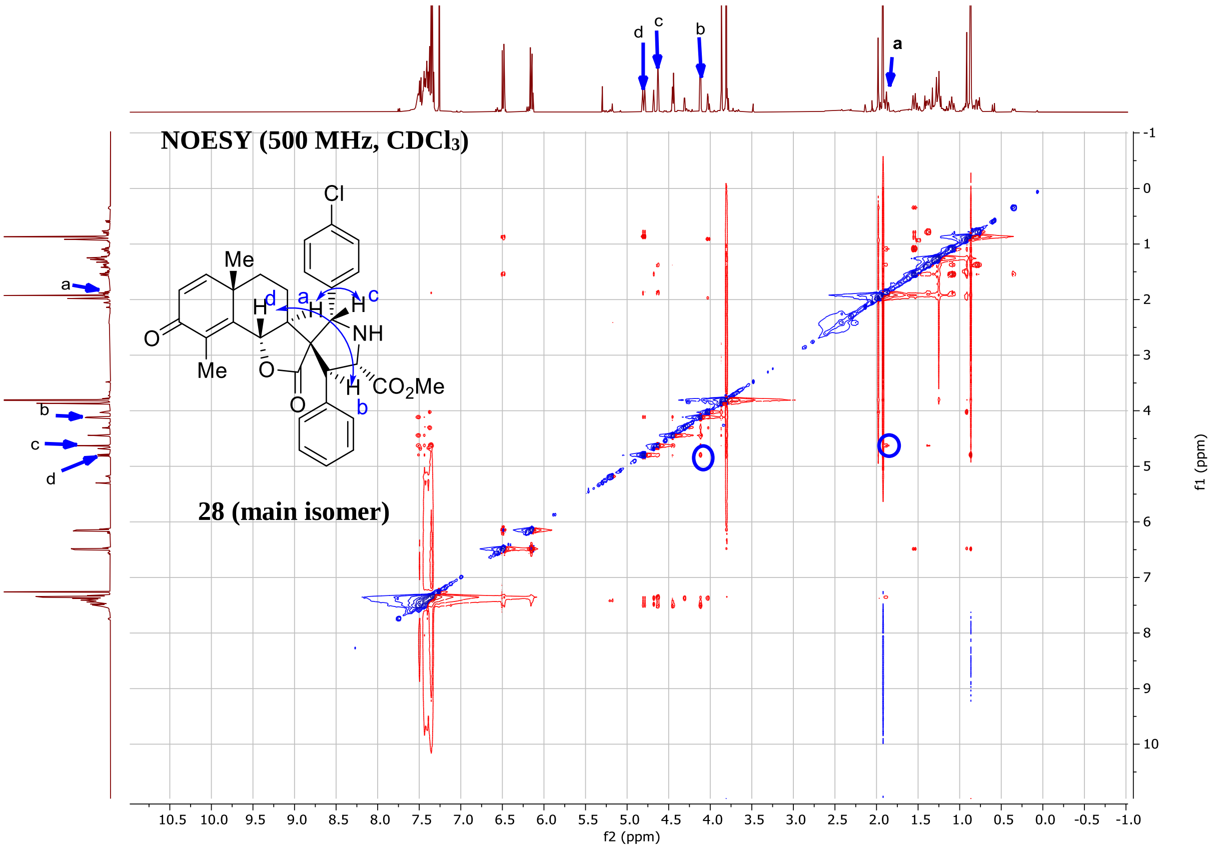
NOESY (500 MHz, CDCl3)
28 (main isomer)
a
b
c
d
Cl
Me
Me
O
NH
CO2Me
f1 (ppm)
f2 (ppm)

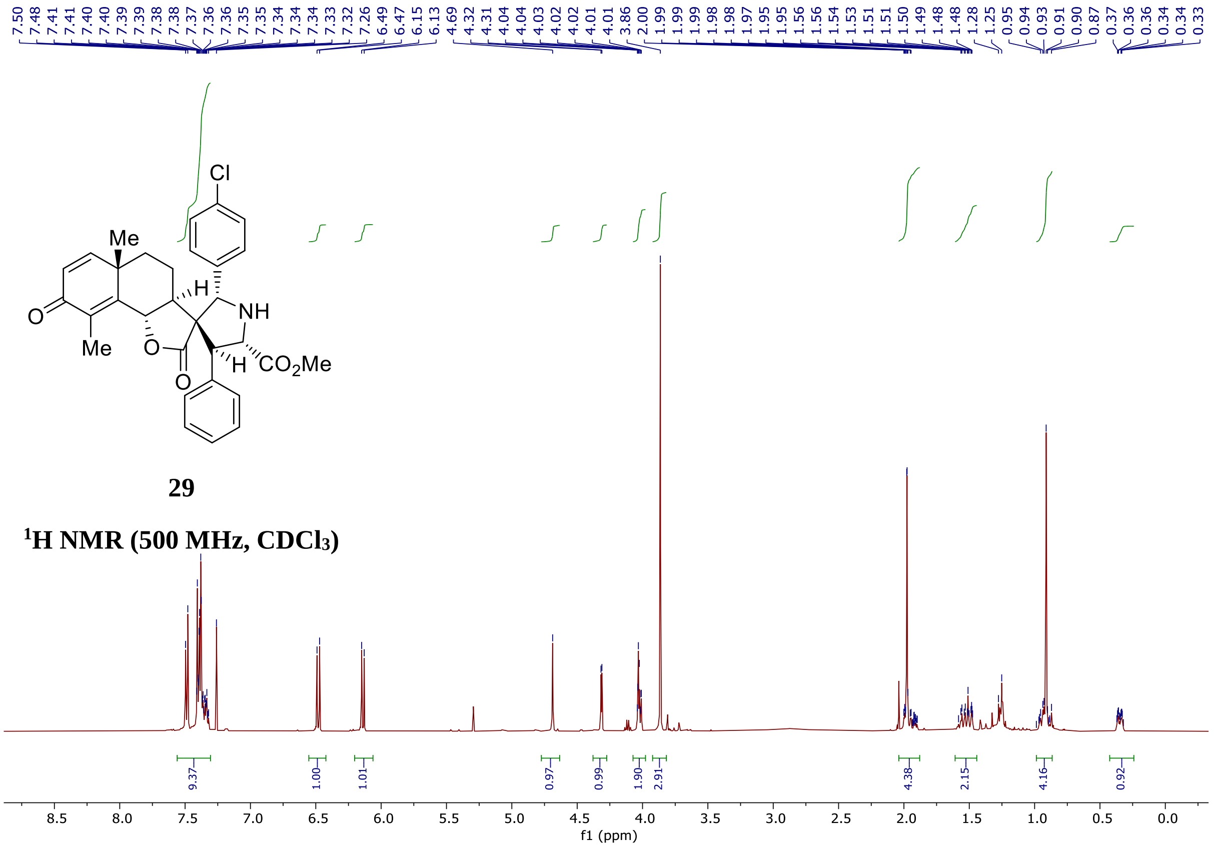

29

^{1}H NMR (500 MHz, $CDCl_3$)

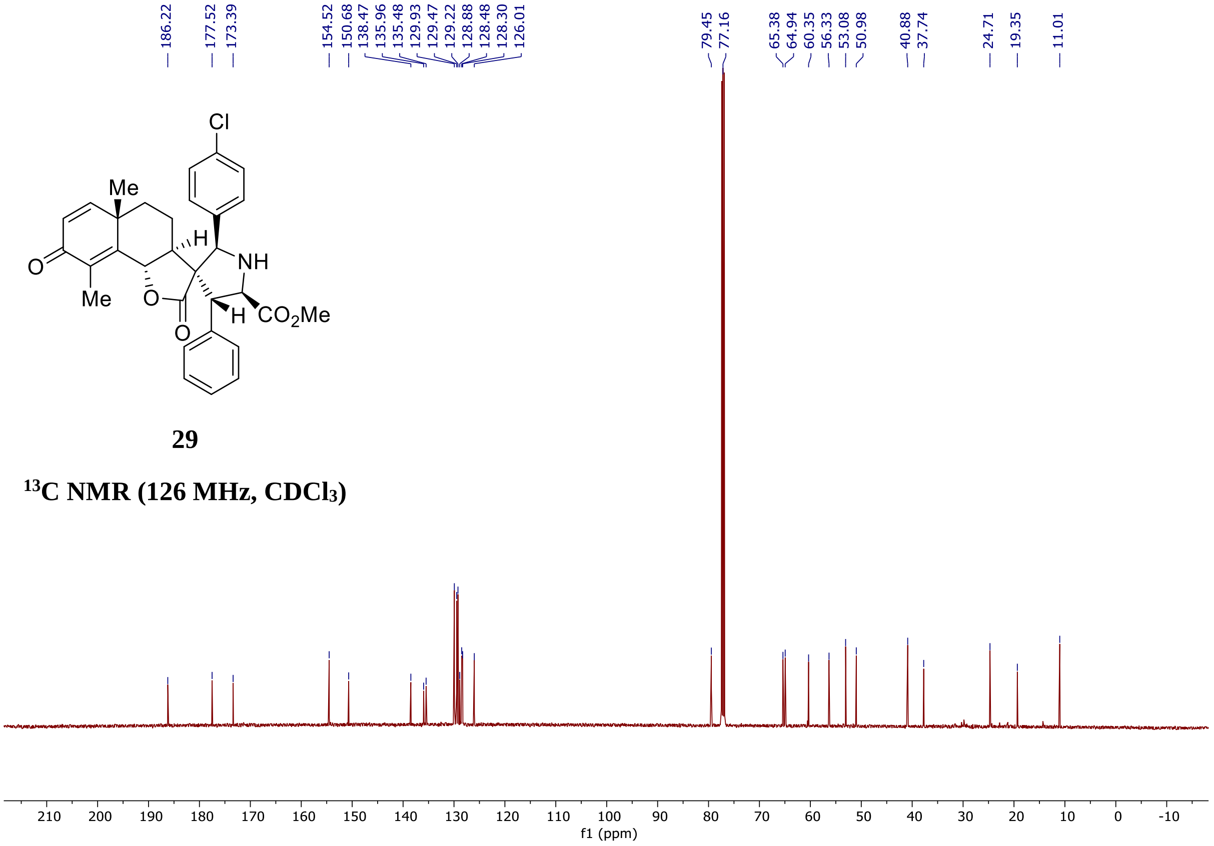

29

^{13}C NMR (126 MHz, $CDCl_3$)

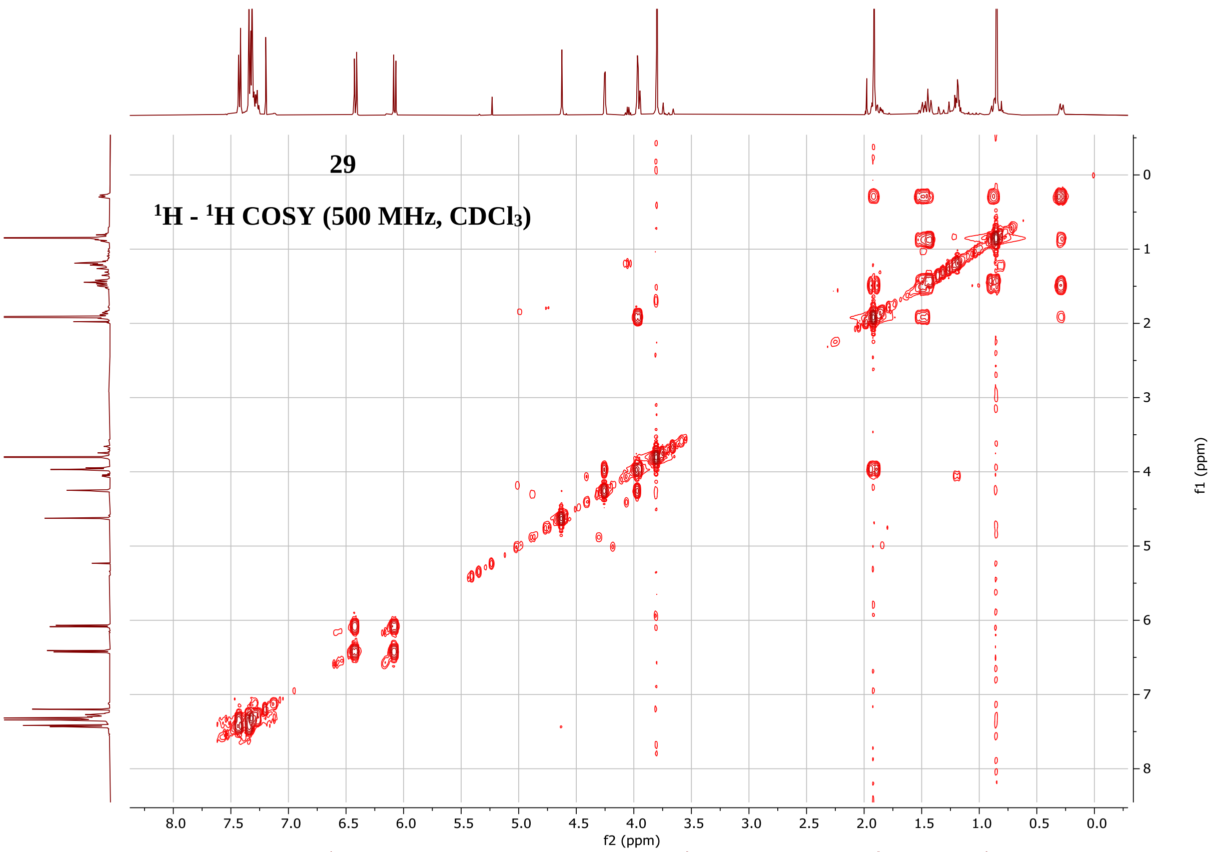
29
^{1}H - ^{1}H COSY (500 MHz, CDCl$_3$)
f2 (ppm)
f1 (ppm)

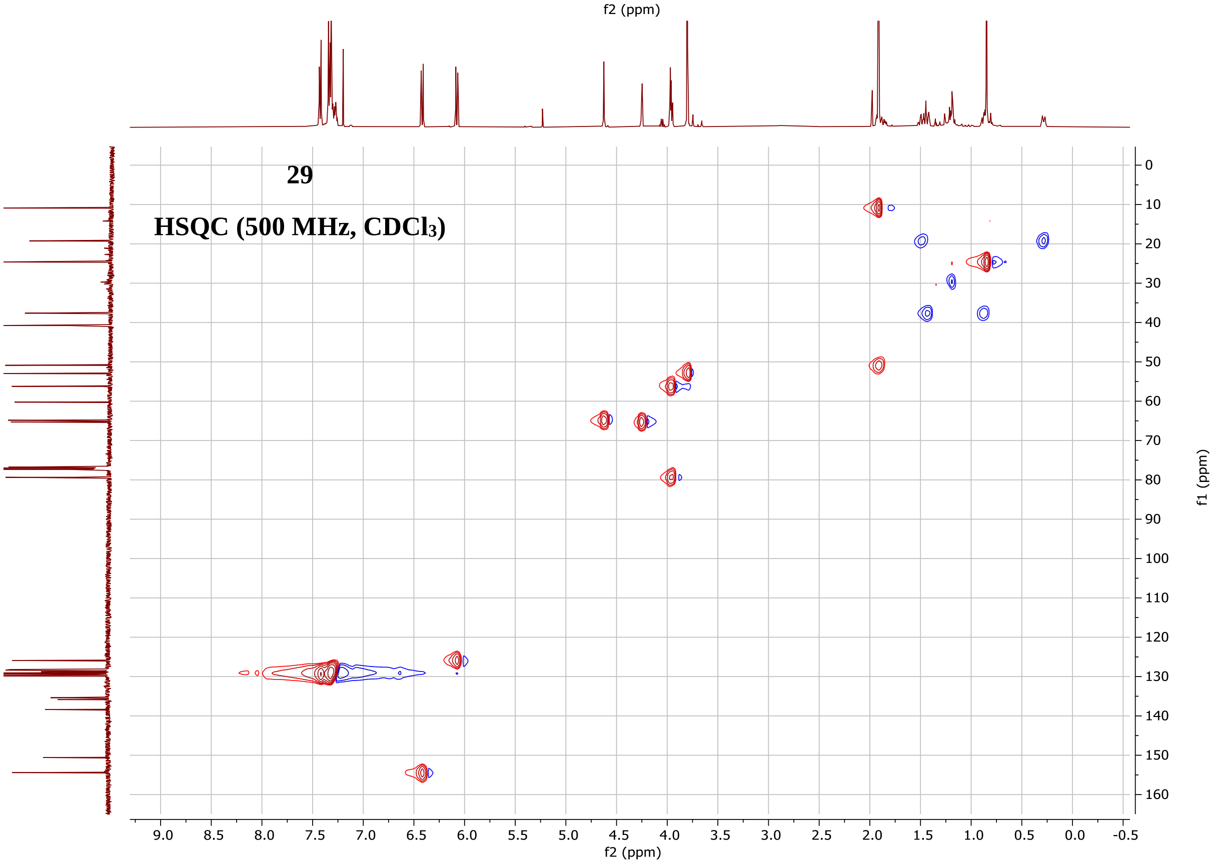
29
HSQC (500 MHz, CDCl$_3$)
f2 (ppm)
f1 (ppm)

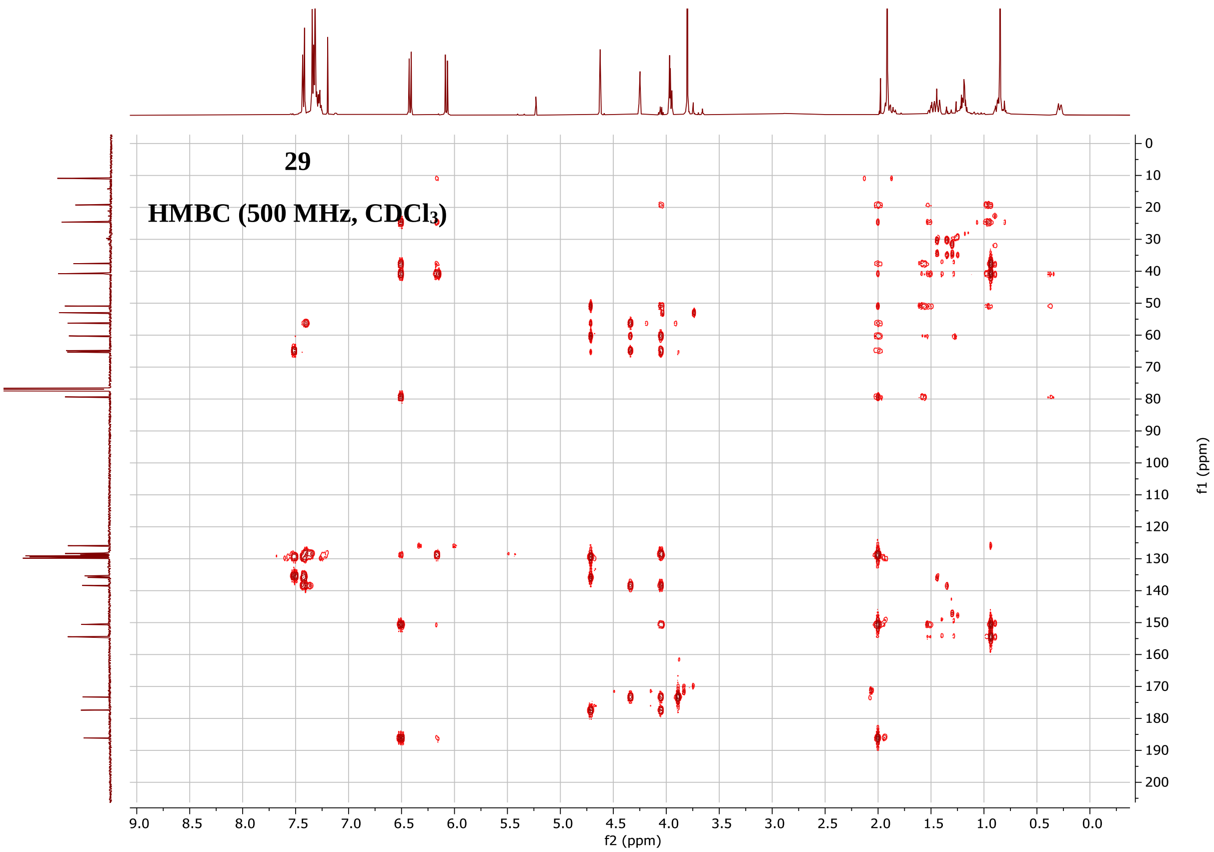
29
HMBC (500 MHz, CDCl3)
f2 (ppm)
f1 (ppm)

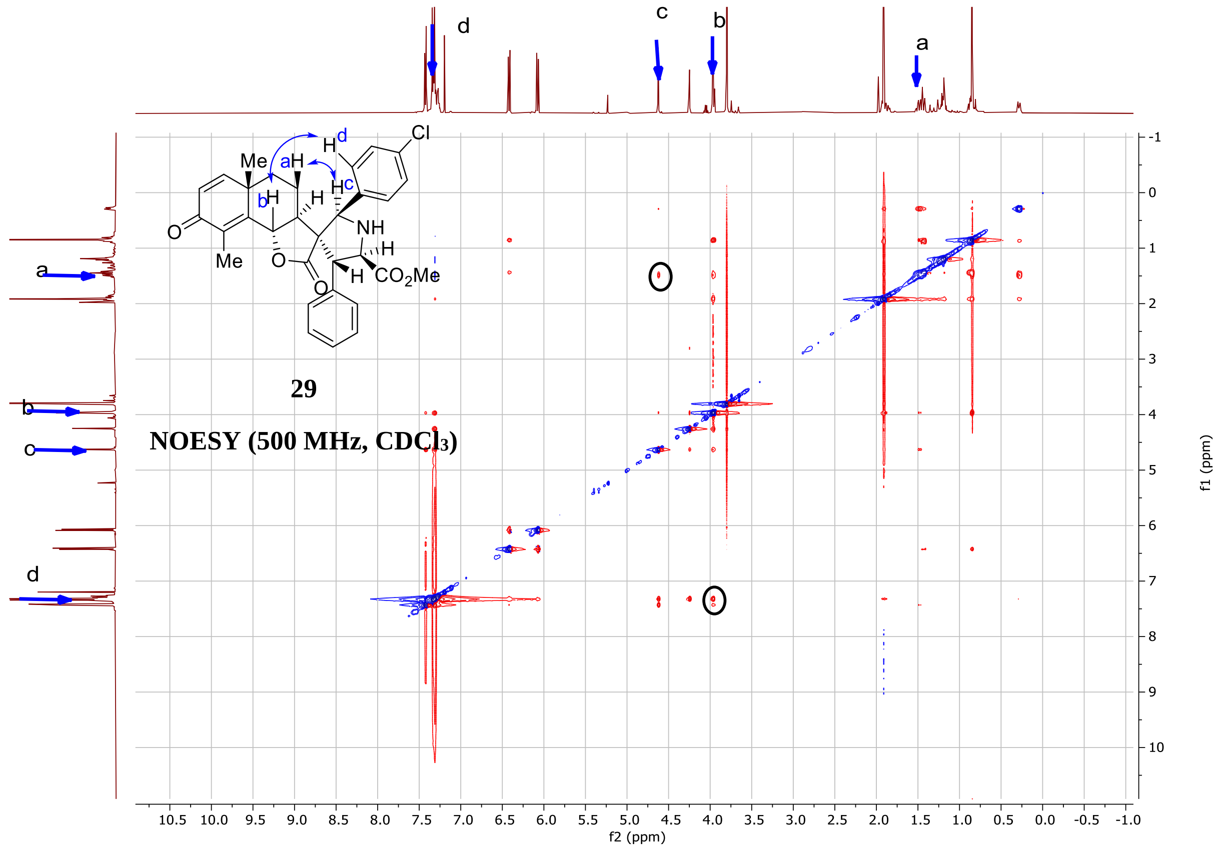
29
NOESY (500 MHz, CDCl3)
a
b
c
d
Me
Cl
NH
CO2Me
O
f2 (ppm)
f1 (ppm)

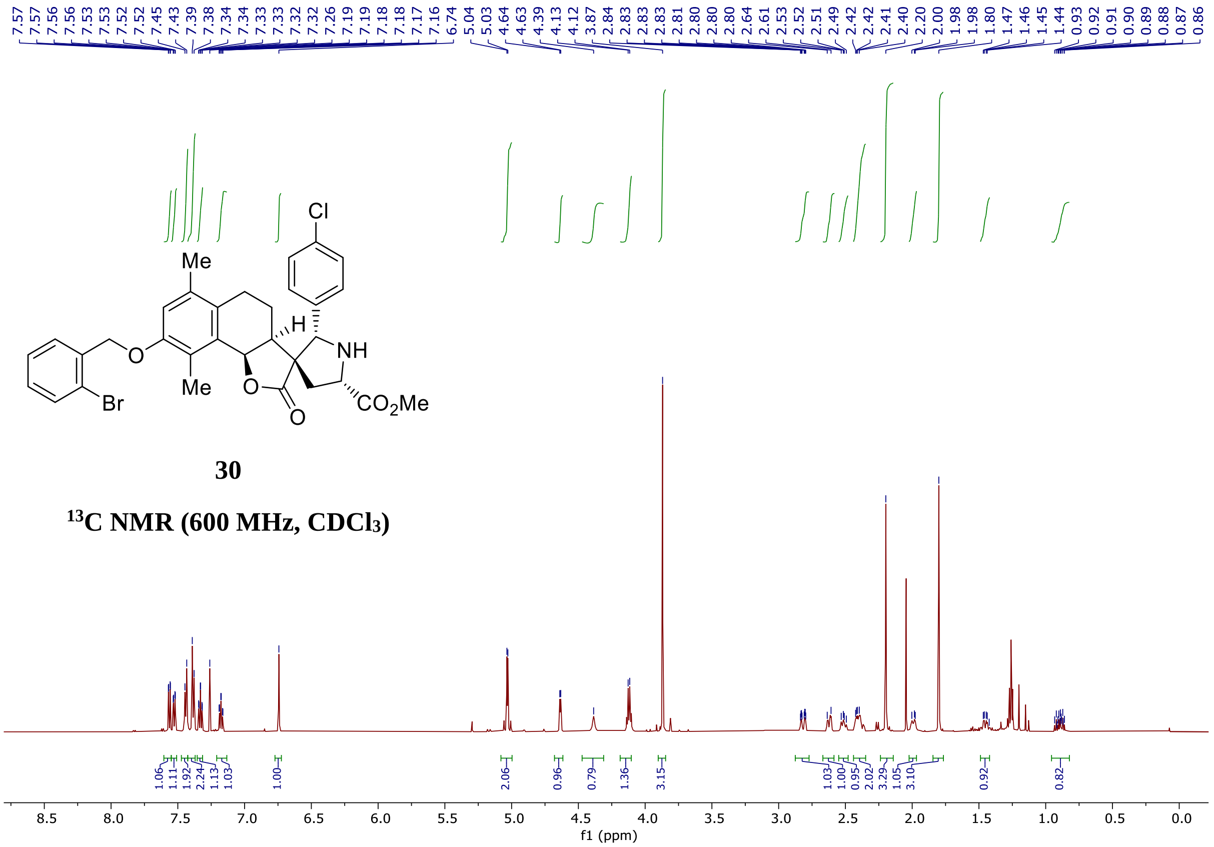
7.57 7.57 7.56 7.53 7.53 7.52 7.52 7.45 7.43 7.39 7.38 7.34 7.34 7.33 7.33 7.32 7.32 7.26 7.19 7.19 7.18 7.17 7.16 6.74 5.04 5.03 4.64 4.63 4.39 4.13 4.12 3.87 2.84 2.83 2.83 2.83 2.81 2.80 2.80 2.80 2.64 2.61 2.53 2.52 2.51 2.49 2.42 2.42 2.41 2.40 2.20 2.00 1.98 1.98 1.80 1.47 1.46 1.45 1.44 0.93 0.92 0.91 0.90 0.89 0.88 0.87 0.86
Cl
Me
H
NH
O
Me
O
Br
O
CO2Me
30
13C NMR (600 MHz, CDCl3)
1.06 1.11 1.92 2.24 1.13 1.03 1.00 2.06 0.96 0.79 1.36 3.15 1.03 1.00 0.95 2.02 3.29 1.05 3.10 0.92 0.82
8.5 8.0 7.5 7.0 6.5 6.0 5.5 5.0 4.5 4.0 3.5 3.0 2.5 2.0 1.5 1.0 0.5 0.0
f1 (ppm)

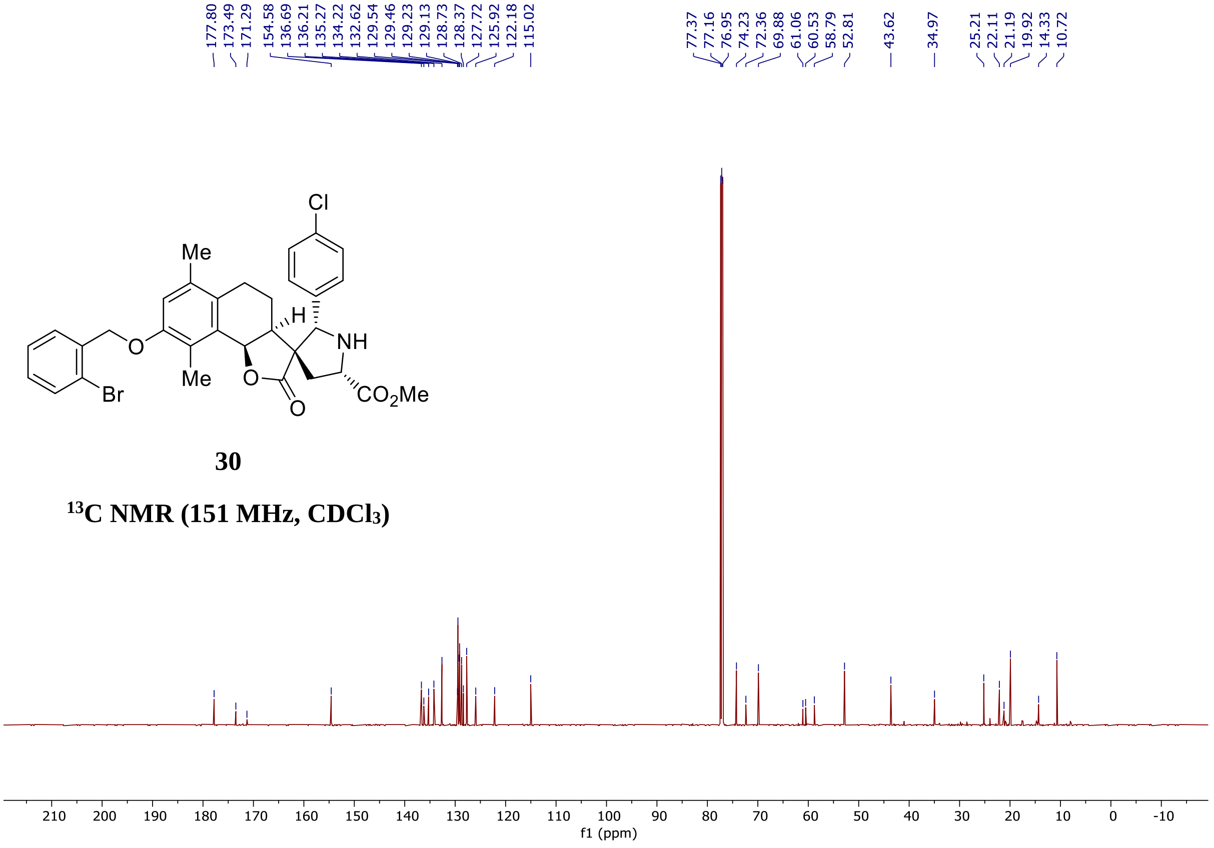
177.80 173.49 171.29 154.58 136.69 136.21 135.27 134.22 132.62 129.54 129.46 129.23 129.13 128.73 128.37 127.72 125.92 122.18 115.02 77.37 77.16 76.95 74.23 72.36 69.88 61.06 60.53 58.79 52.81 43.62 34.97 25.21 22.11 21.19 19.92 14.33 10.72
Cl
Me
H
NH
O
Me
O
Br
O
CO2Me
30
13C NMR (151 MHz, CDCl3)
210 200 190 180 170 160 150 140 130 120 110 100 90 80 70 60 50 40 30 20 10 0 -10
f1 (ppm)

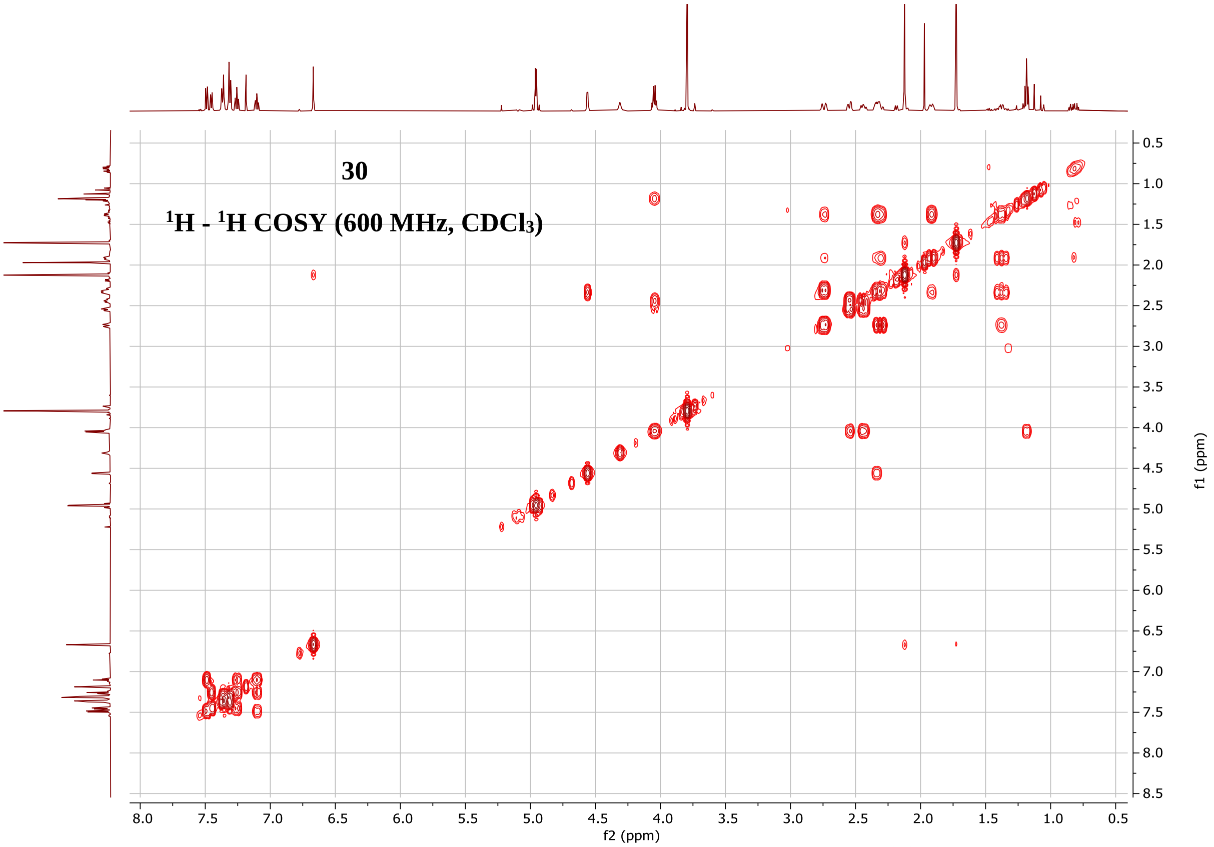
30
1H - 1H COSY (600 MHz, CDCl3)
f2 (ppm)
f1 (ppm)

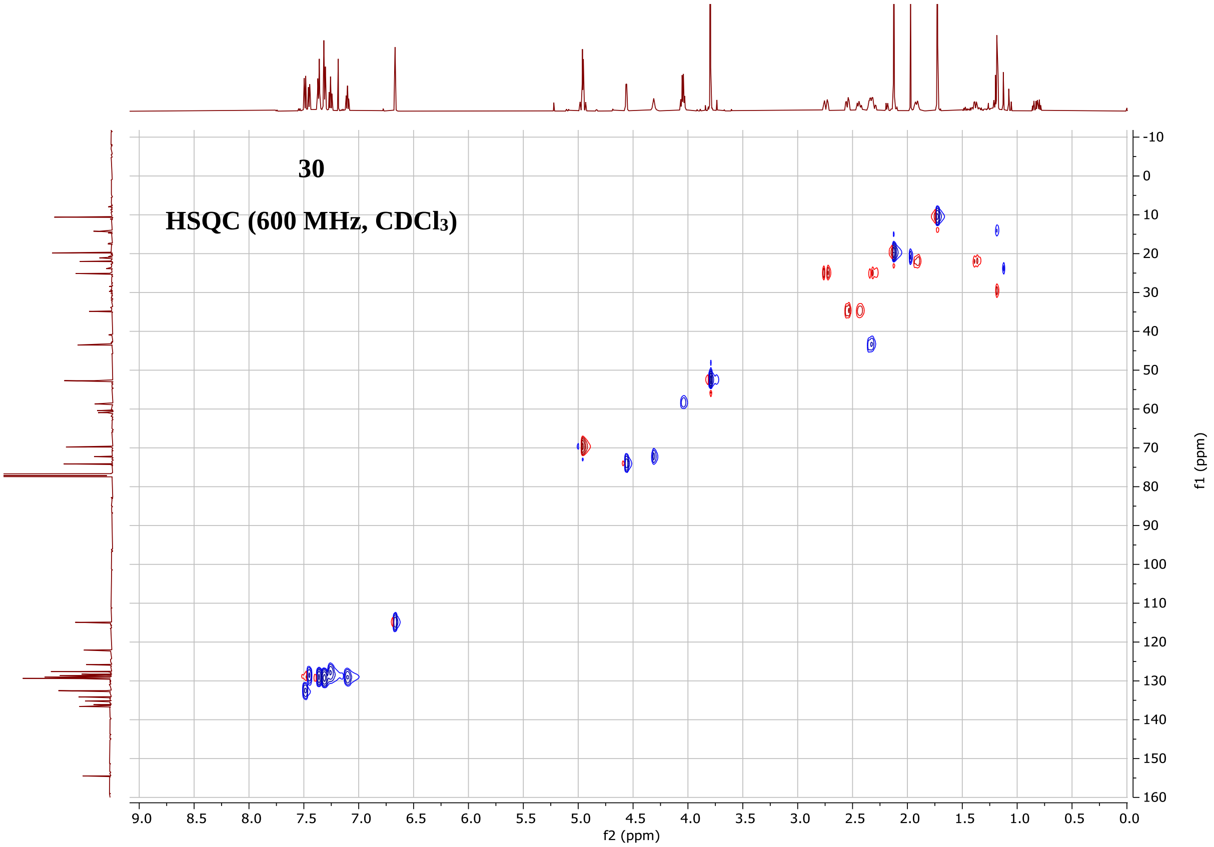
30
HSQC (600 MHz, CDCl3)
f2 (ppm)
f1 (ppm)

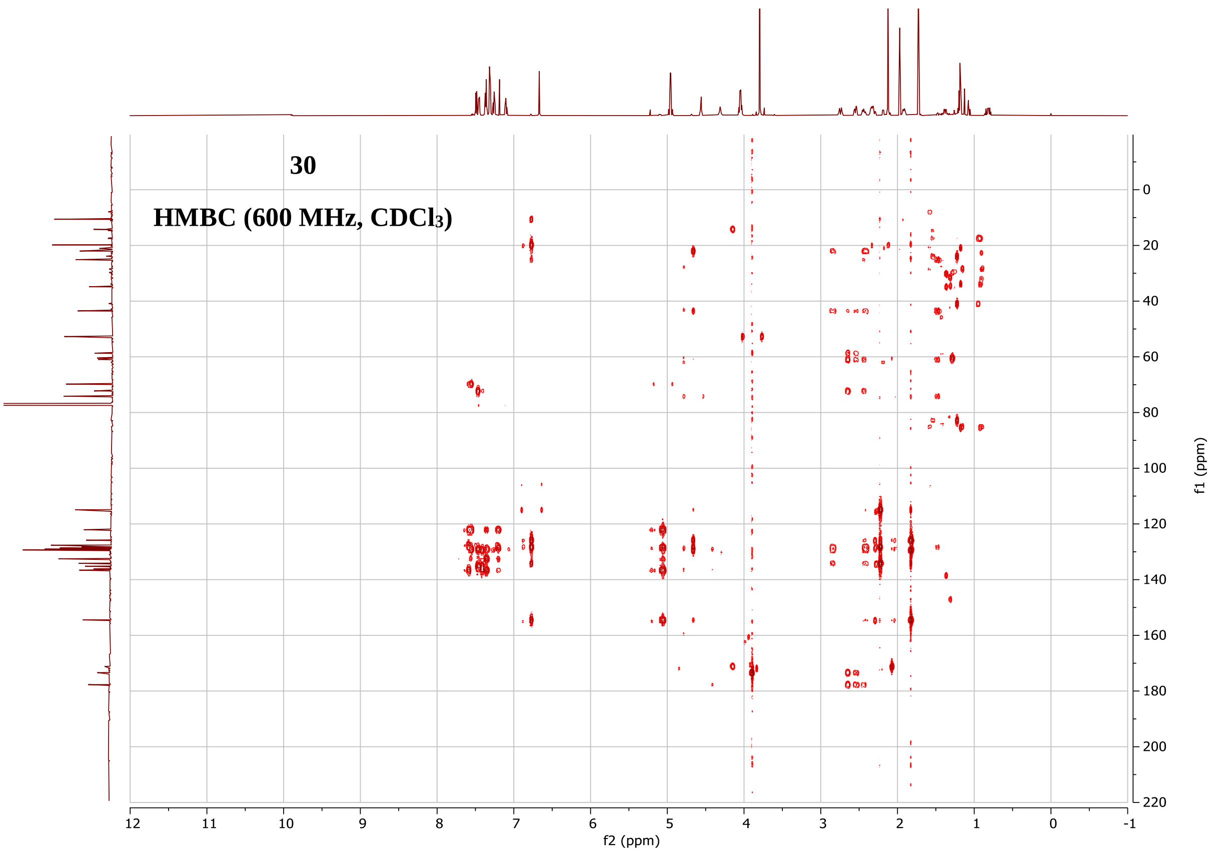

30
HMBC (600 MHz, CDCl3)
f2 (ppm)
f1 (ppm)

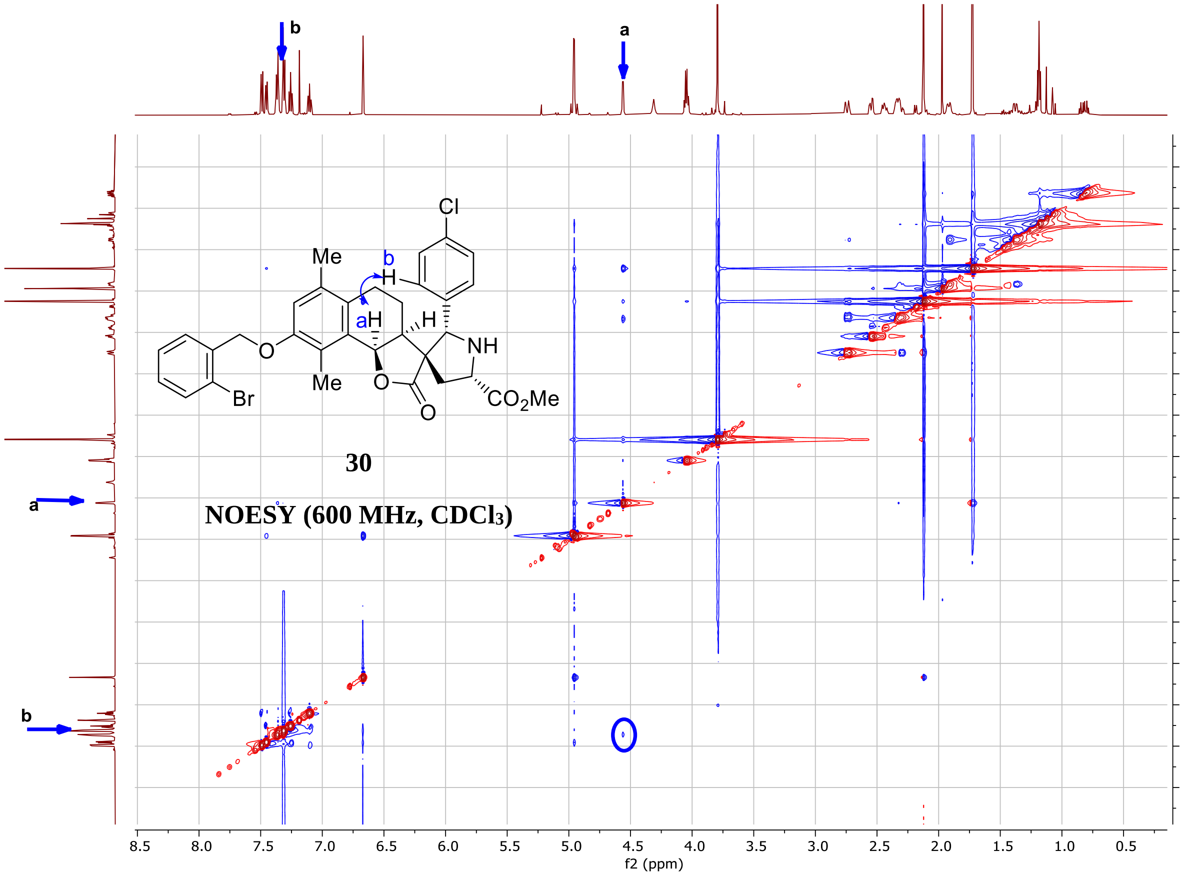

a
b
Me
Cl
H
aH
H
NH
O
Br
Me
O
O
CO2Me
30
NOESY (600 MHz, CDCl3)
f2 (ppm)
f1 (ppm)

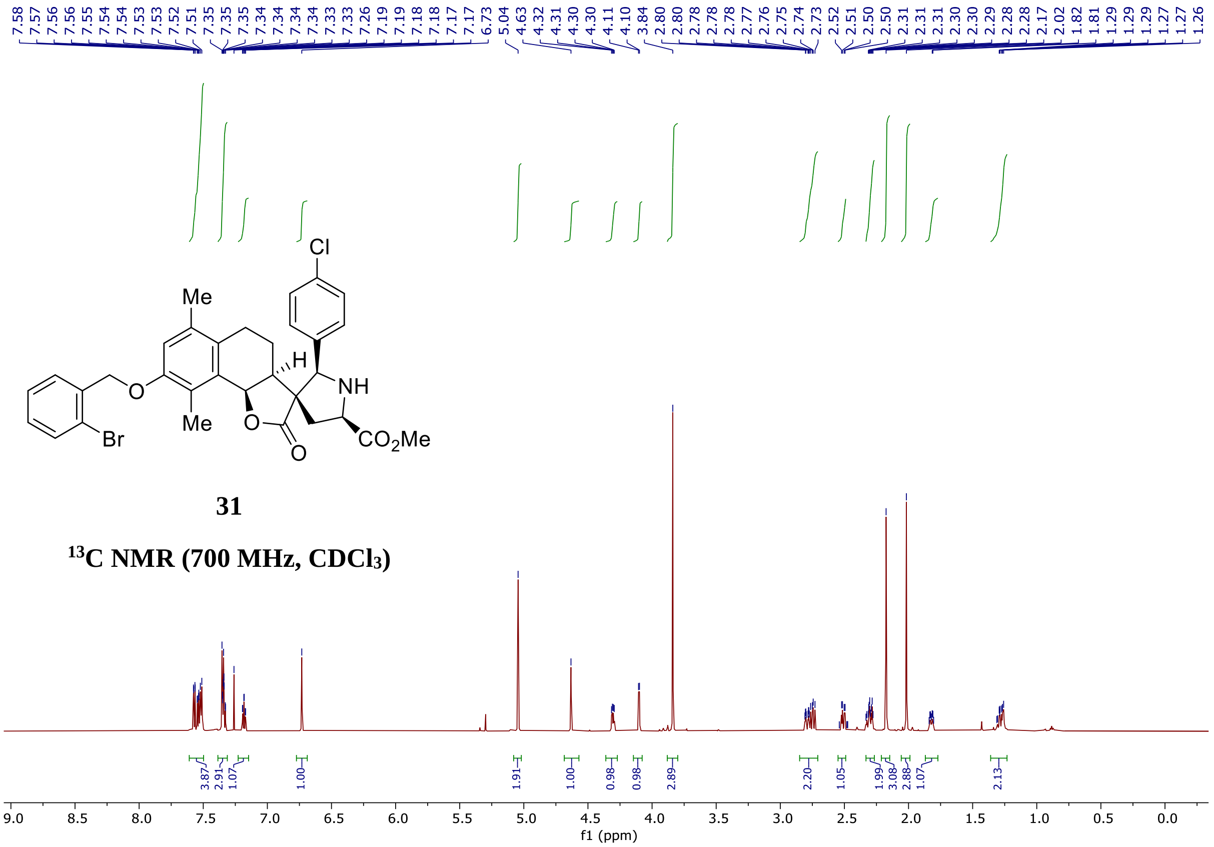
7.58 7.57 7.56 7.56 7.55 7.54 7.54 7.53 7.53 7.52 7.51 7.35 7.35 7.34 7.34 7.34 7.34 7.33 7.33 7.26 7.19 7.19 7.18 7.18 7.17 7.17 6.73 5.04 4.63 4.32 4.31 4.30 4.30 4.11 4.10 3.84 2.80 2.80 2.78 2.78 2.77 2.76 2.75 2.74 2.73 2.52 2.51 2.50 2.50 2.31 2.31 2.31 2.30 2.30 2.29 2.28 2.28 2.17 2.02 1.82 1.81 1.29 1.29 1.29 1.27 1.26
Cl
Me
H
NH
O
Me
O
Br
O
CO_2Me
31
13C NMR (700 MHz, CDCl3)
3.87 2.91 1.07 1.00 1.91 1.00 0.98 0.98 2.89 2.20 1.05 1.99 3.08 2.88 1.07 2.13
f1 (ppm)

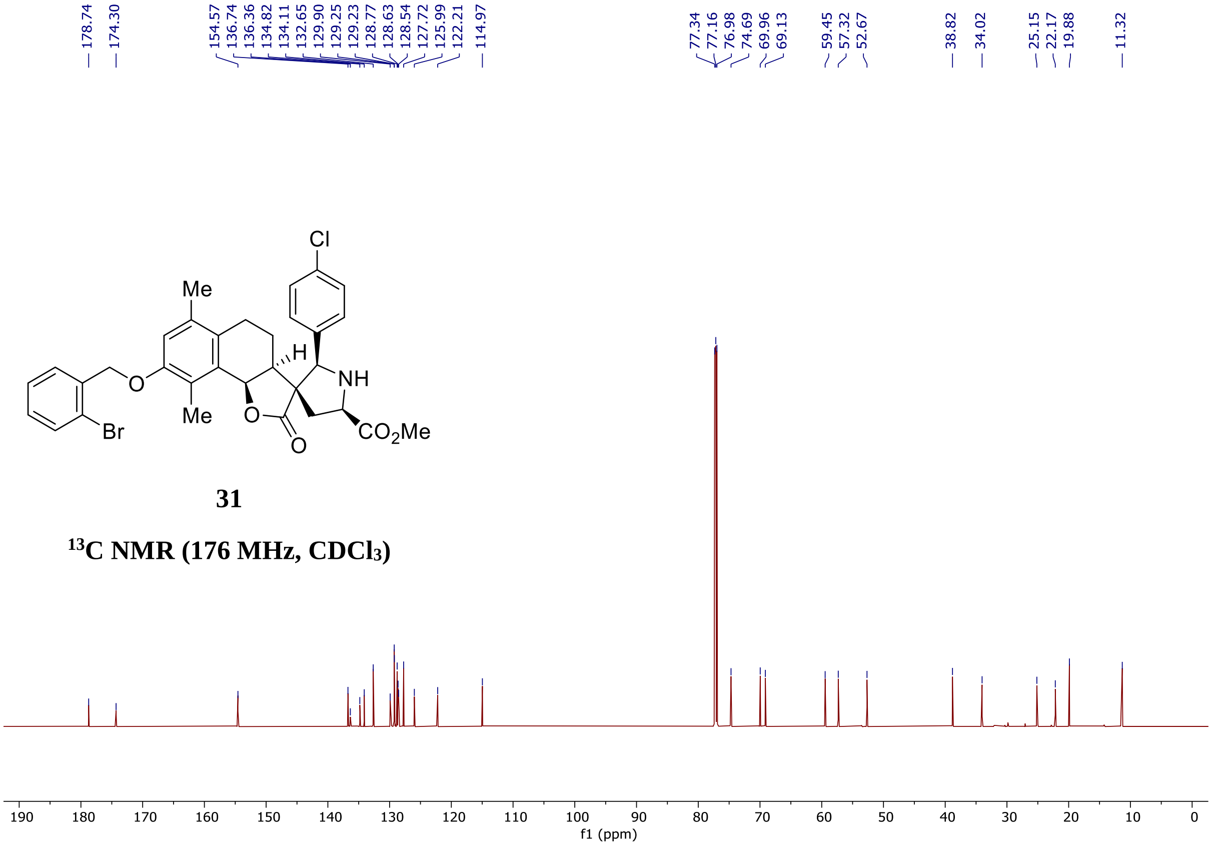
178.74 174.30 154.57 136.74 136.36 134.82 134.11 132.65 129.90 129.25 129.23 128.77 128.63 128.54 127.72 125.99 122.21 114.97 77.34 77.16 76.98 74.69 69.96 69.13 59.45 57.32 52.67 38.82 34.02 25.15 22.17 19.88 11.32
Cl
Me
H
NH
O
Me
O
Br
O
CO_2Me
31
13C NMR (176 MHz, CDCl3)
f1 (ppm)

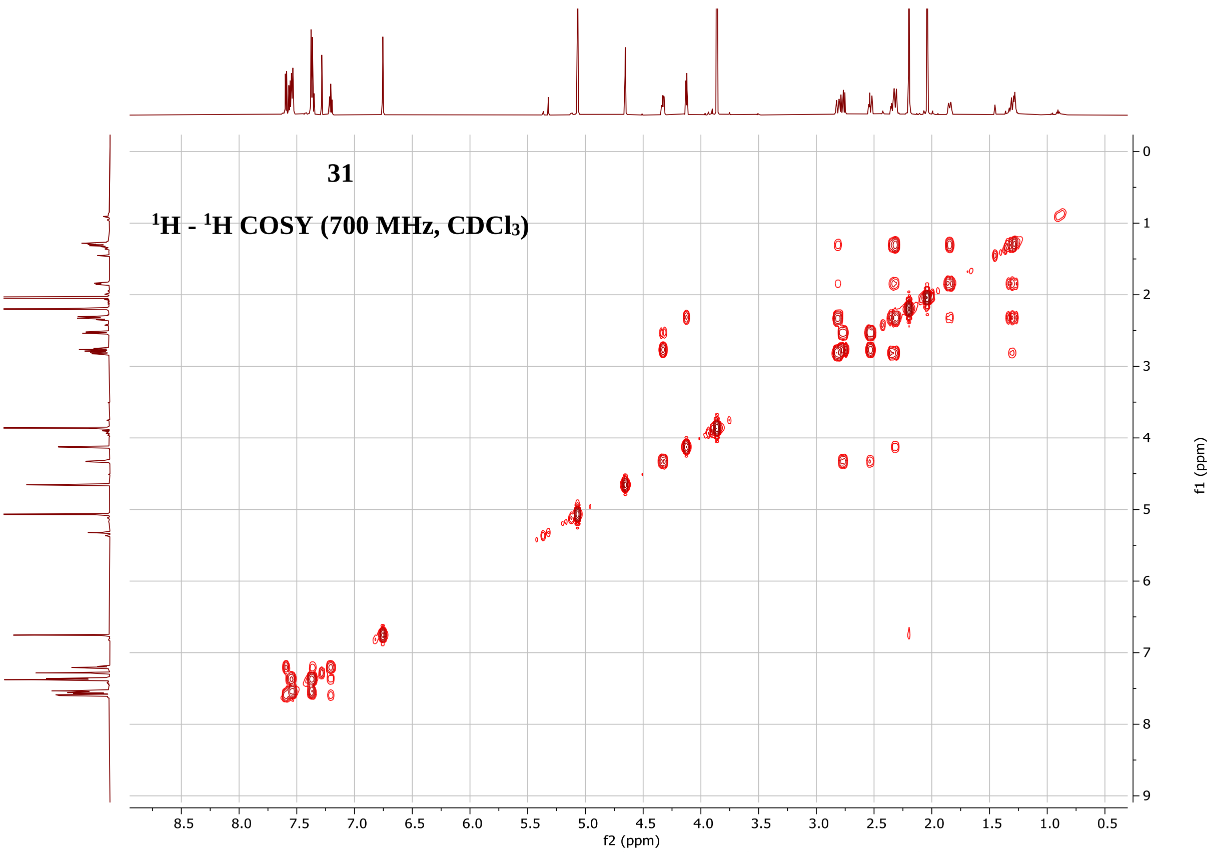
31
¹H - ¹H COSY (700 MHz, CDCl3)
f2 (ppm)
f1 (ppm)

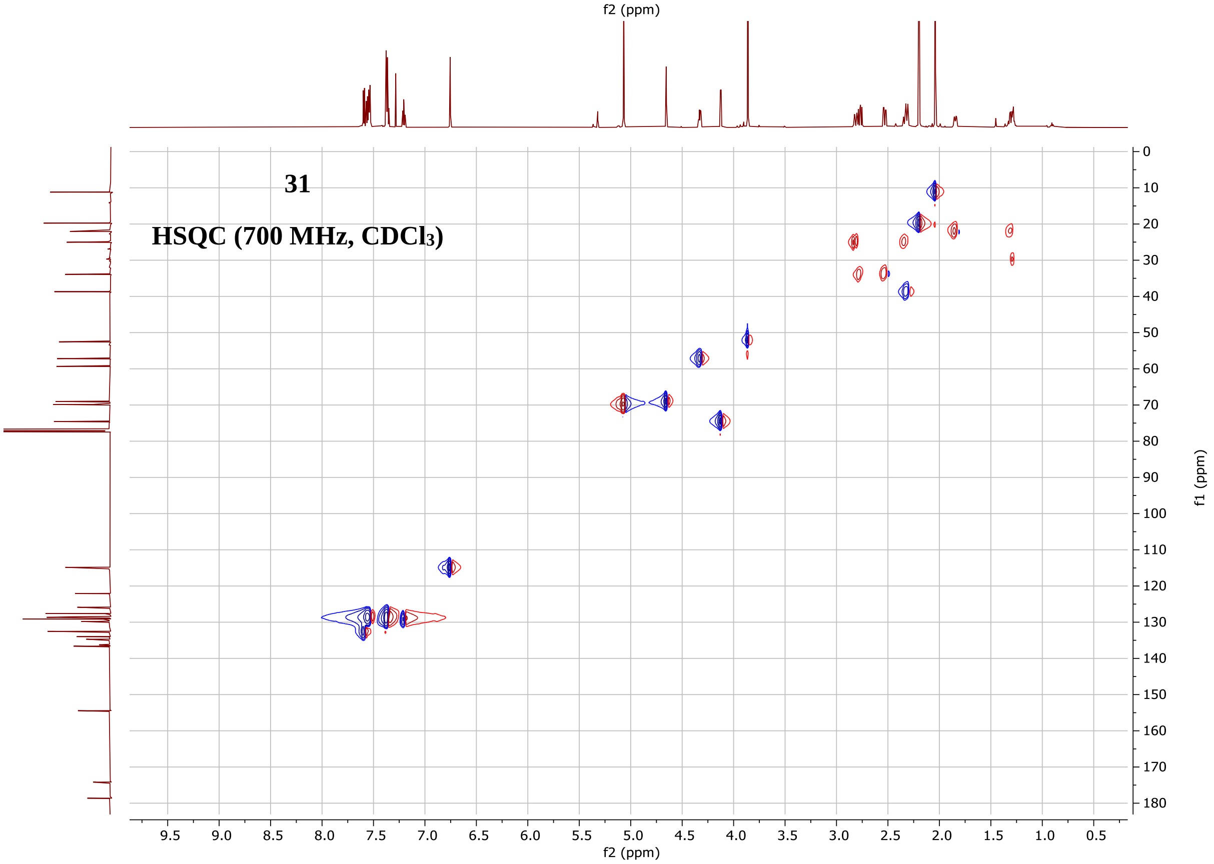
31
HSQC (700 MHz, CDCl3)
f2 (ppm)
f1 (ppm)

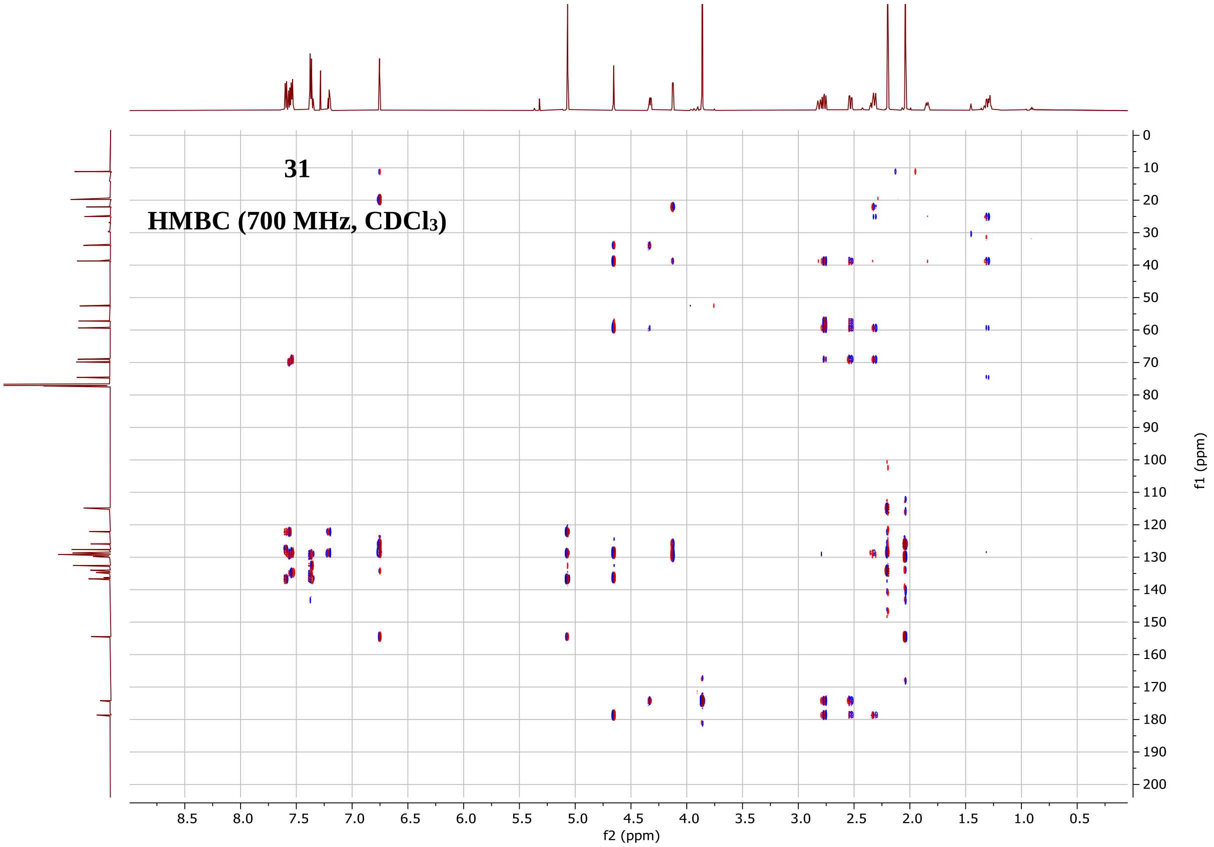
31
HMBC (700 MHz, CDCl3)
f2 (ppm)
f1 (ppm)

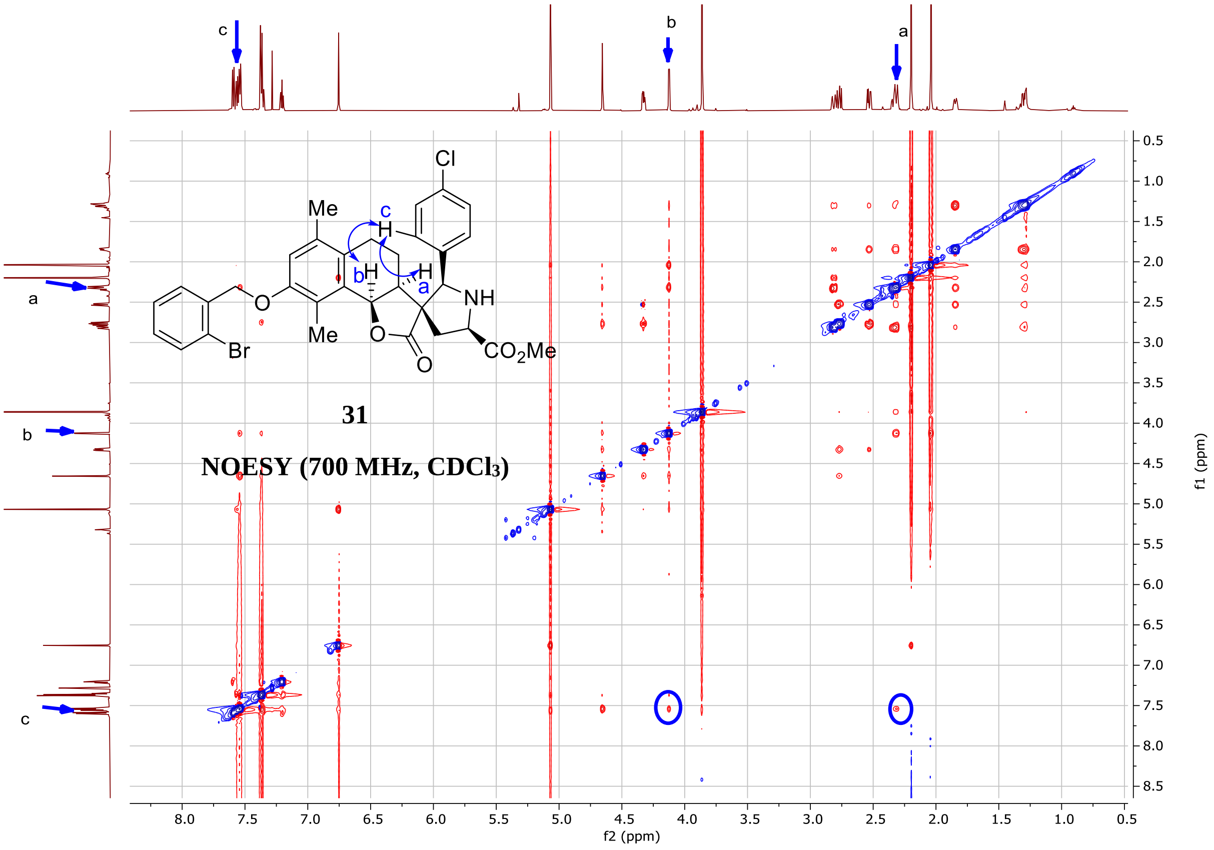
a
b
c
Cl
Me
Me
O
Br
NH
CO2Me
O
31
NOESY (700 MHz, CDCl3)
f2 (ppm)
f1 (ppm)

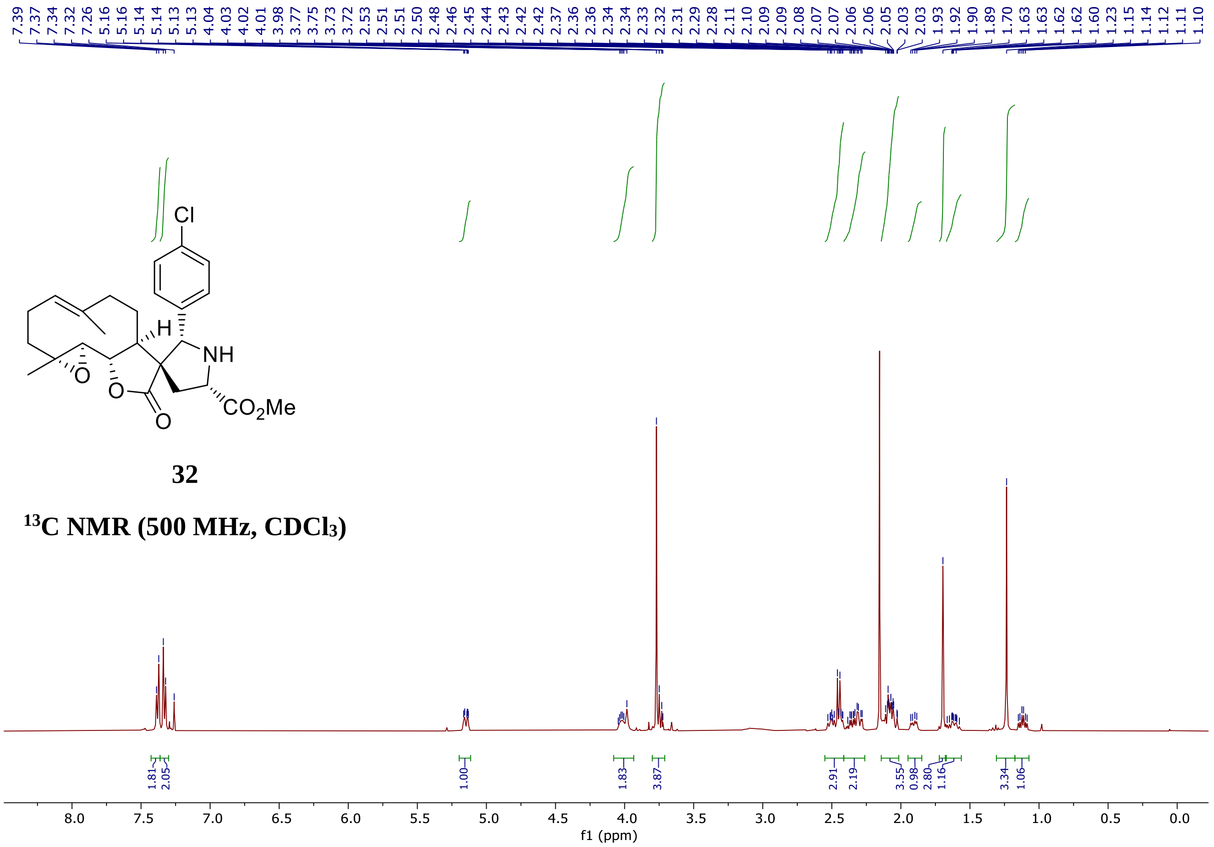

32

^{13}C NMR (500 MHz, CDCl$_3$)

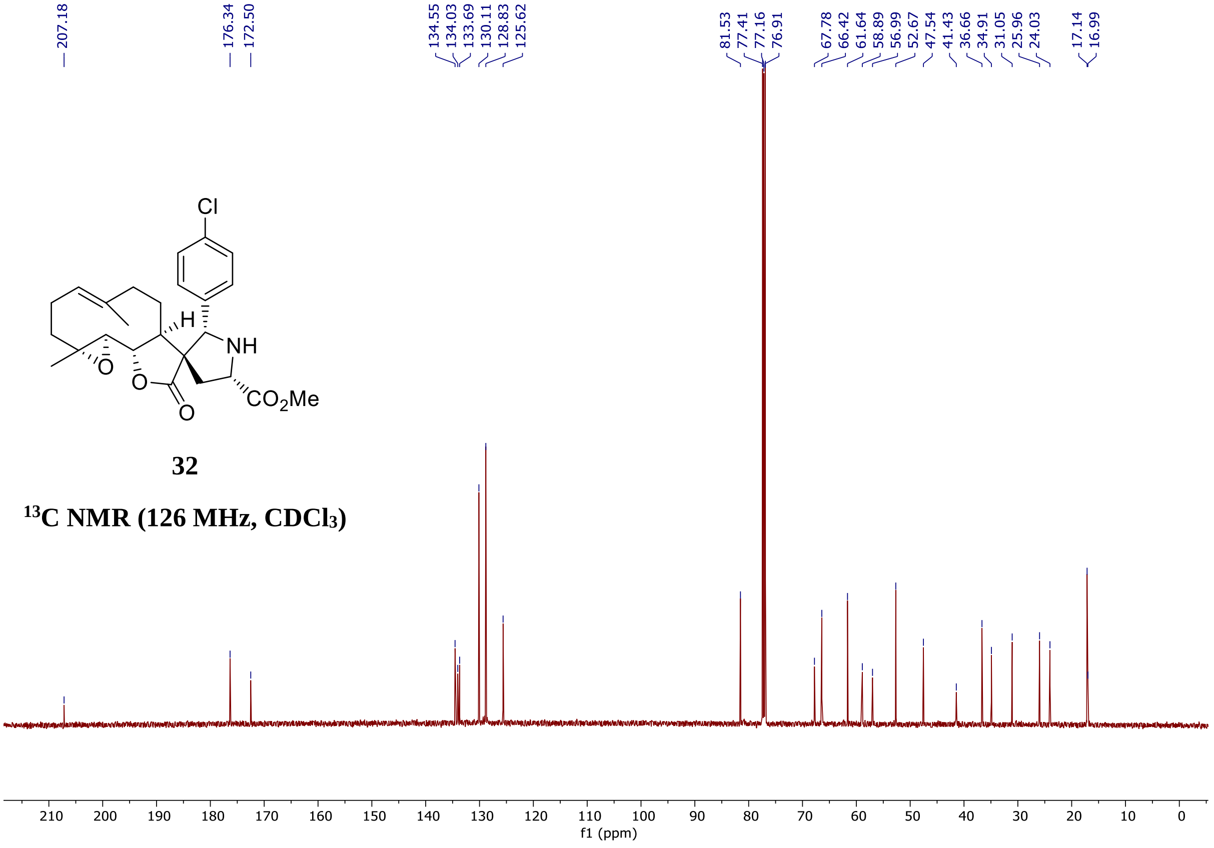

32

^{13}C NMR (126 MHz, CDCl$_3$)

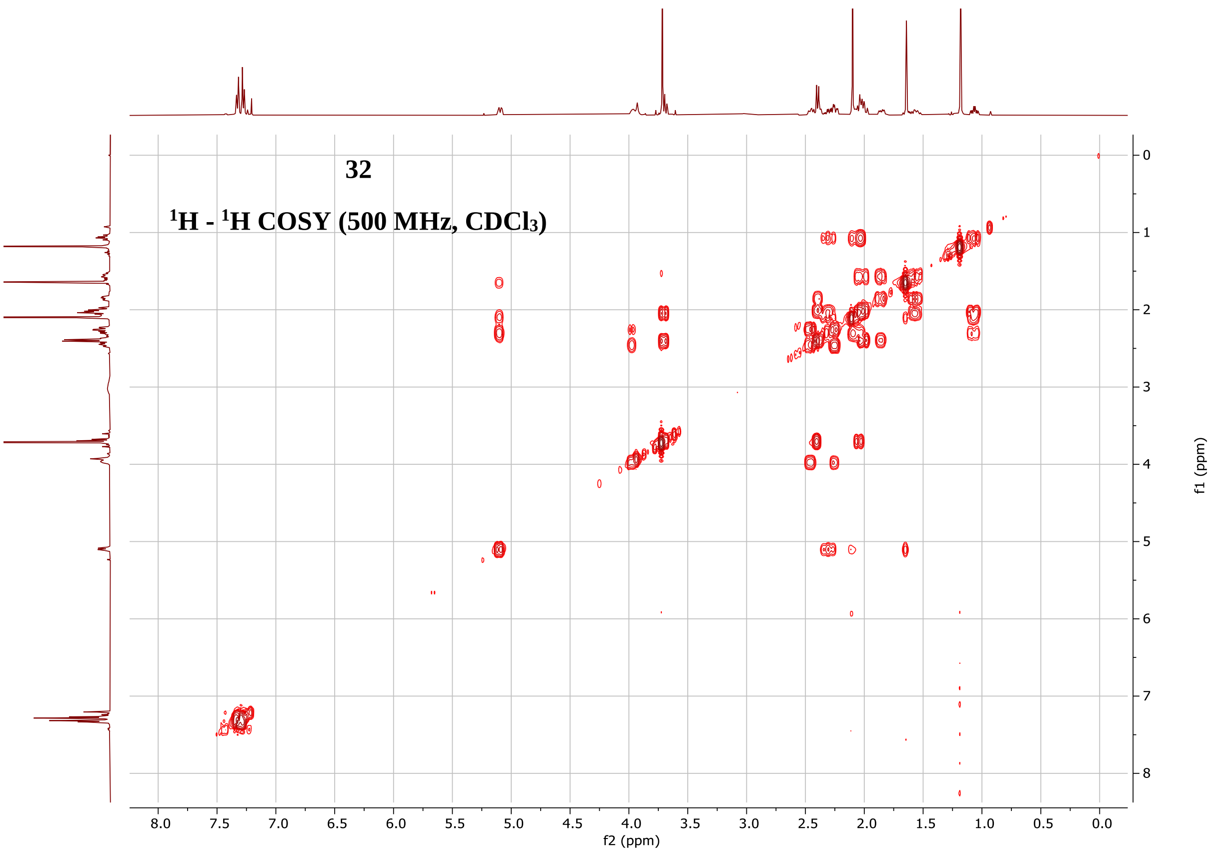

32
^{1}H - ^{1}H COSY (500 MHz, CDCl$_3$)
f2 (ppm)
f1 (ppm)

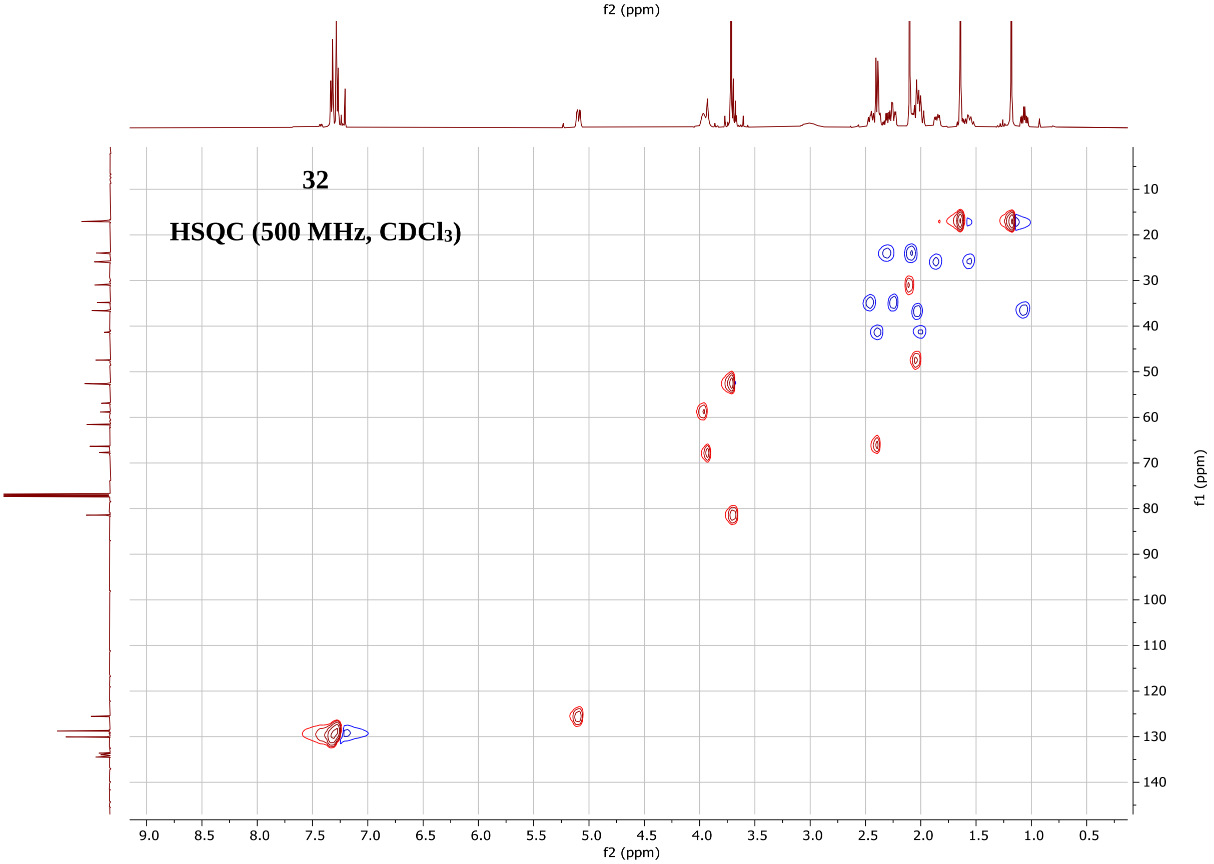

32
HSQC (500 MHz, CDCl$_3$)
f2 (ppm)
f1 (ppm)

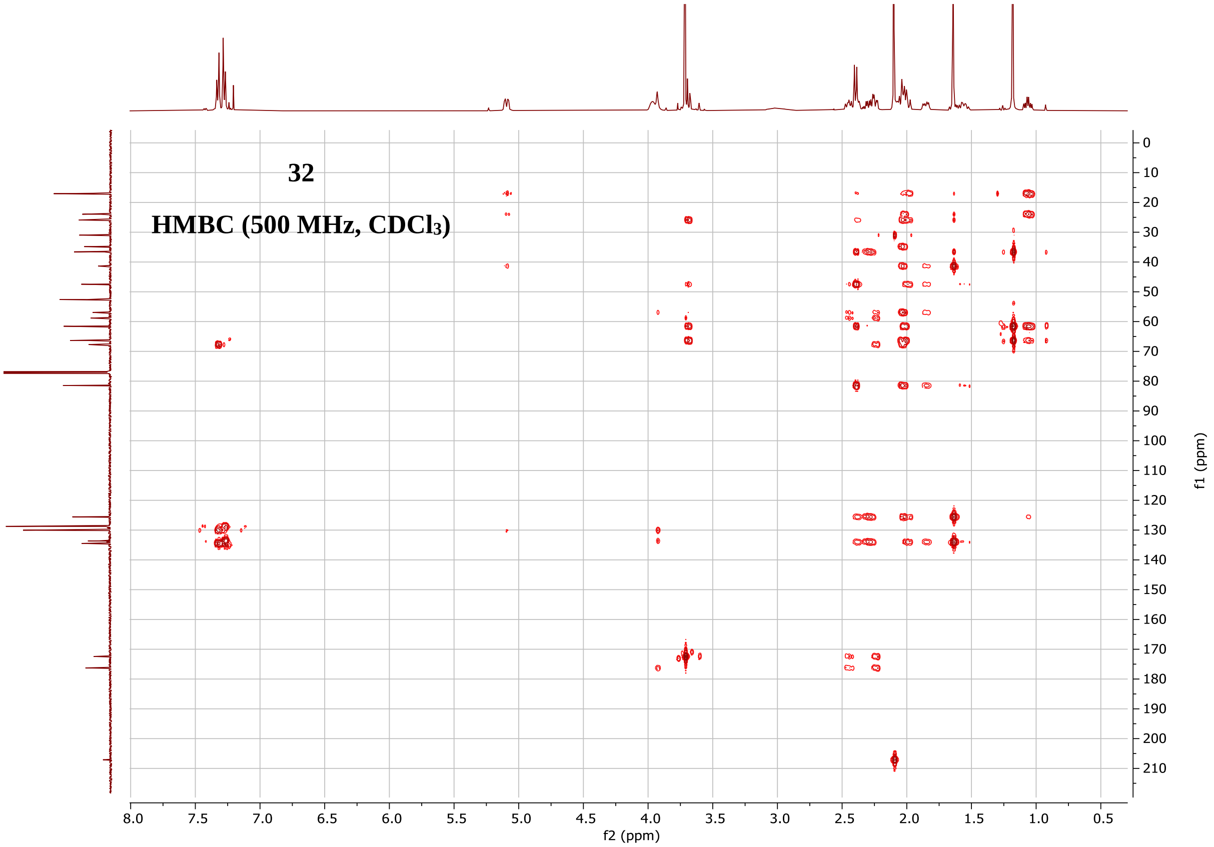
32
HMBC (500 MHz, CDCl3)
f2 (ppm)
f1 (ppm)

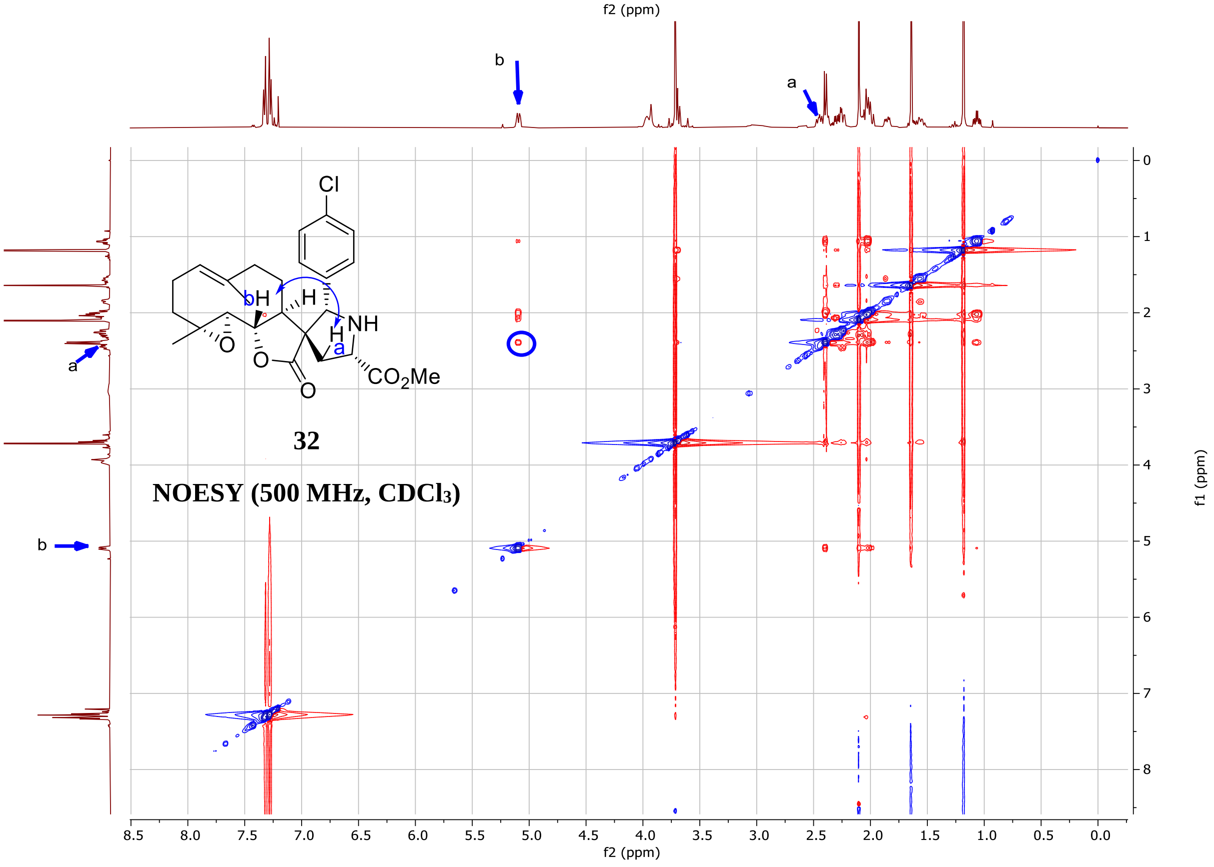
Cl
H
H
H
NH
a
b
O
O
O
CO2Me
32
NOESY (500 MHz, CDCl3)
a
b
f2 (ppm)
f1 (ppm)

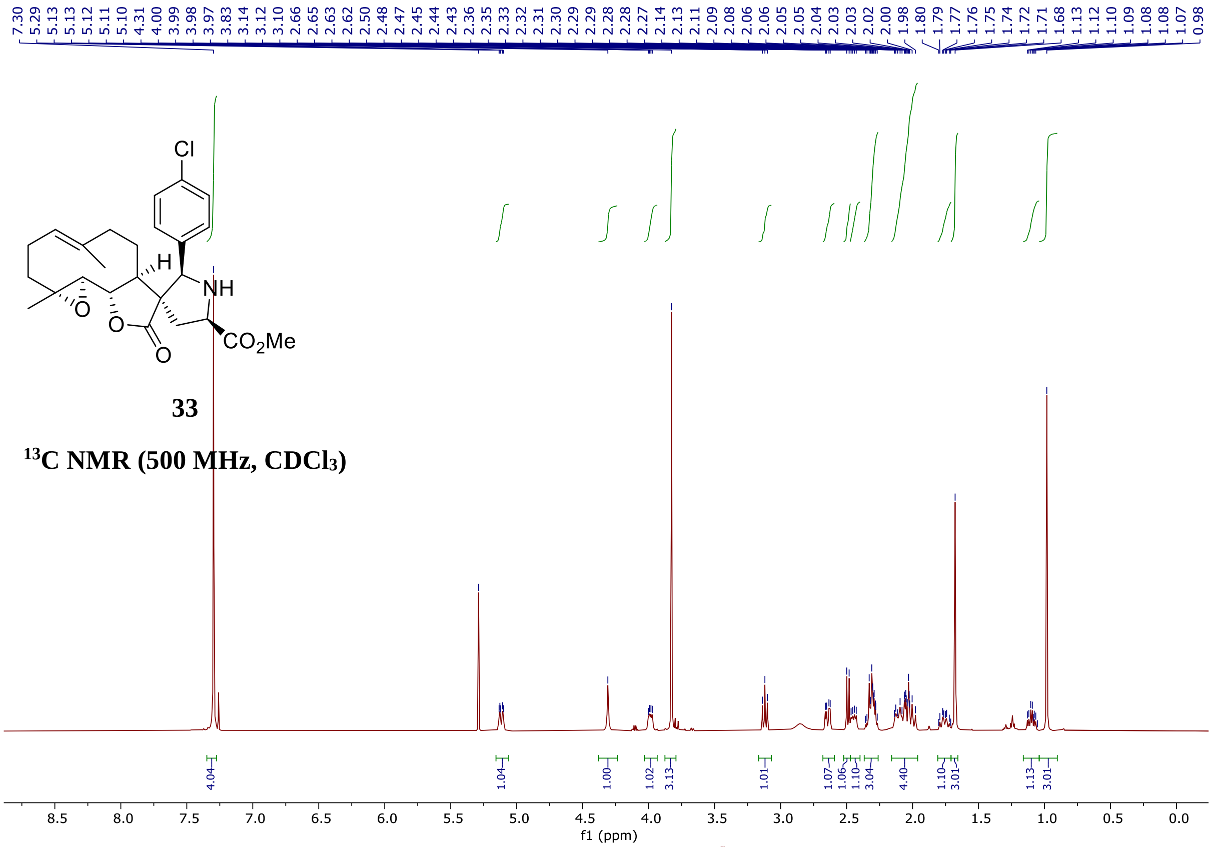

33

^{13}C NMR (500 MHz, CDCl$_3$)

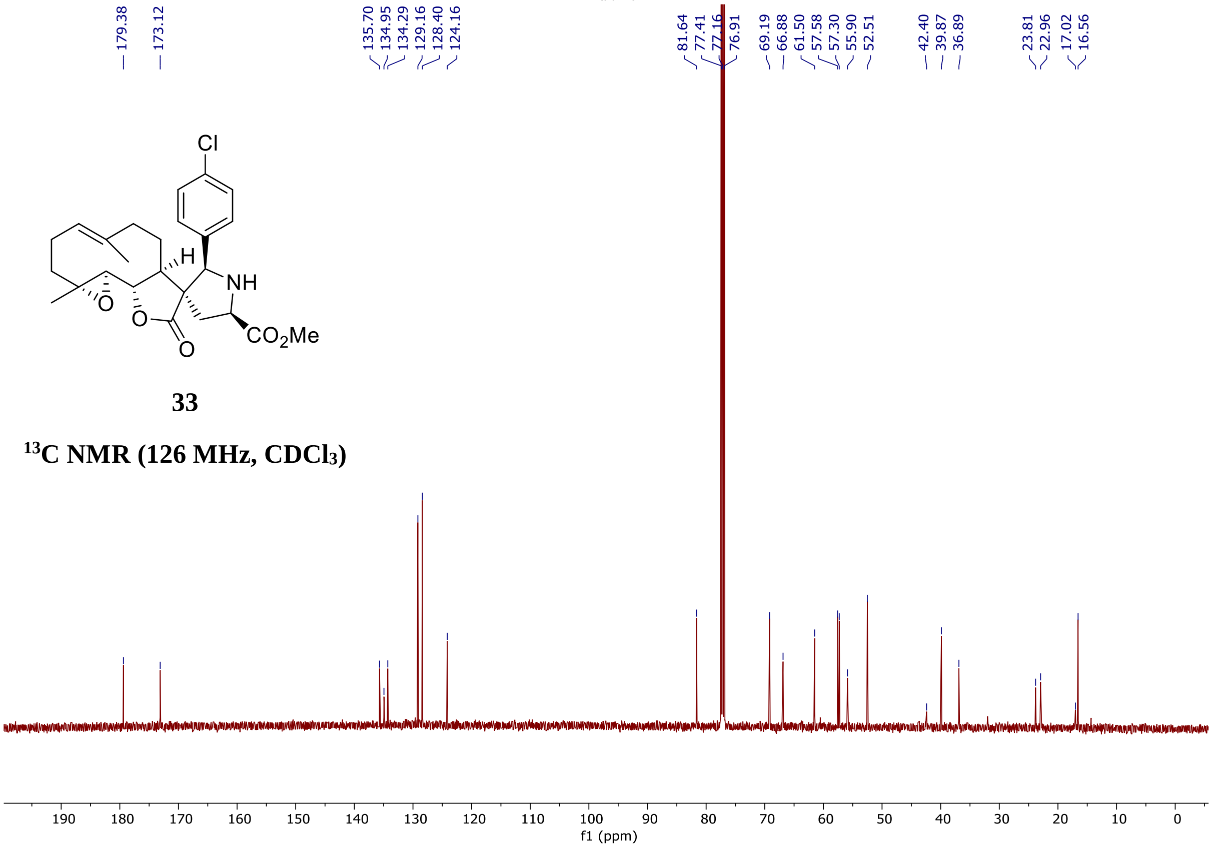

33

^{13}C NMR (126 MHz, CDCl$_3$)

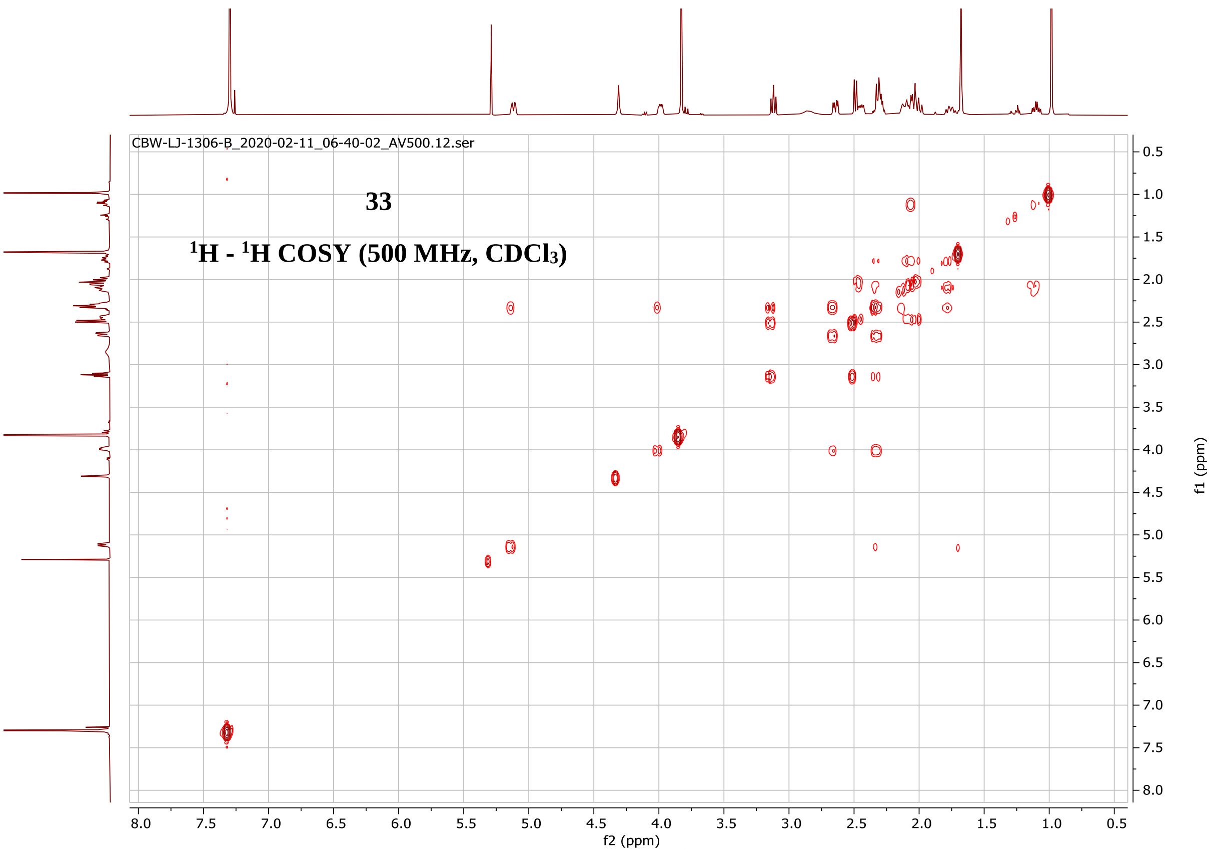
CBW-LJ-1306-B_2020-02-11_06-40-02_AV500.12.ser
33
1H - 1H COSY (500 MHz, CDCl3)
f2 (ppm)
f1 (ppm)

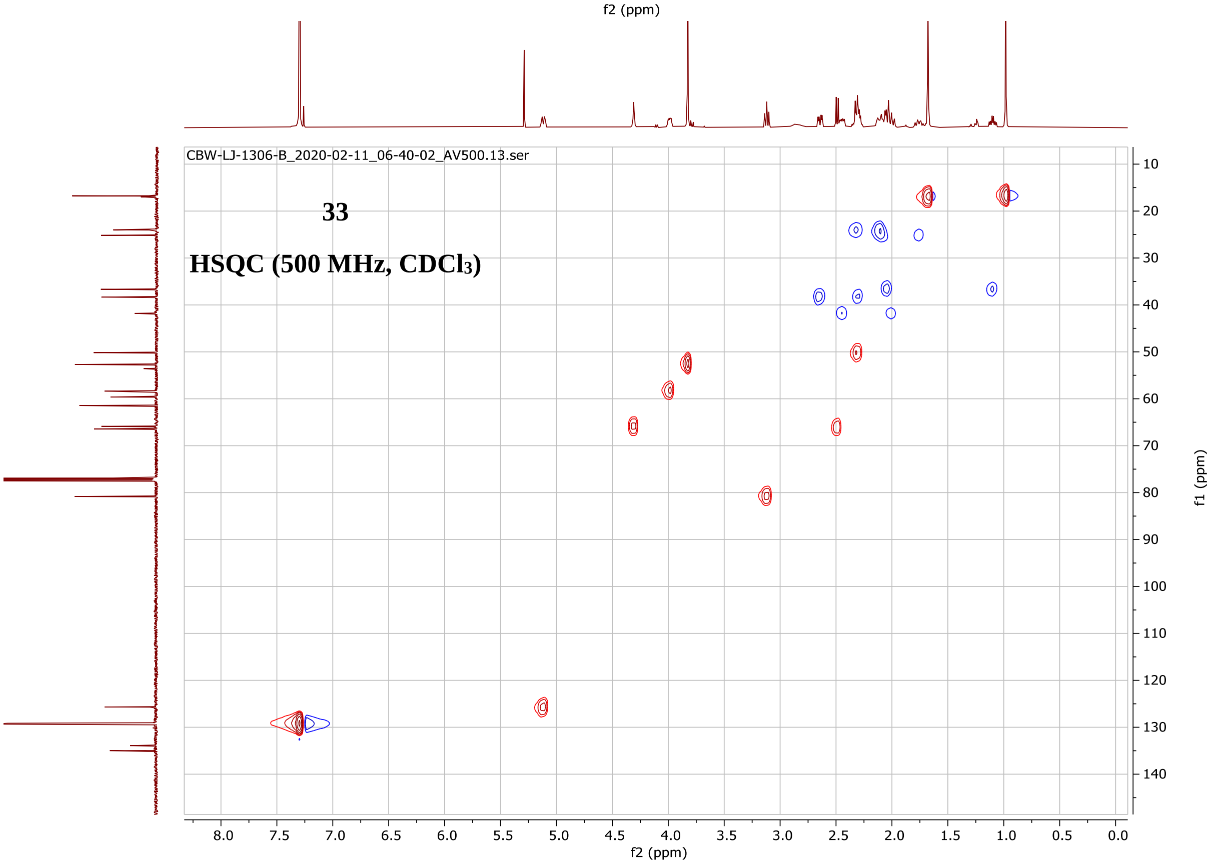
CBW-LJ-1306-B_2020-02-11_06-40-02_AV500.13.ser
33
HSQC (500 MHz, CDCl3)
f2 (ppm)
f1 (ppm)

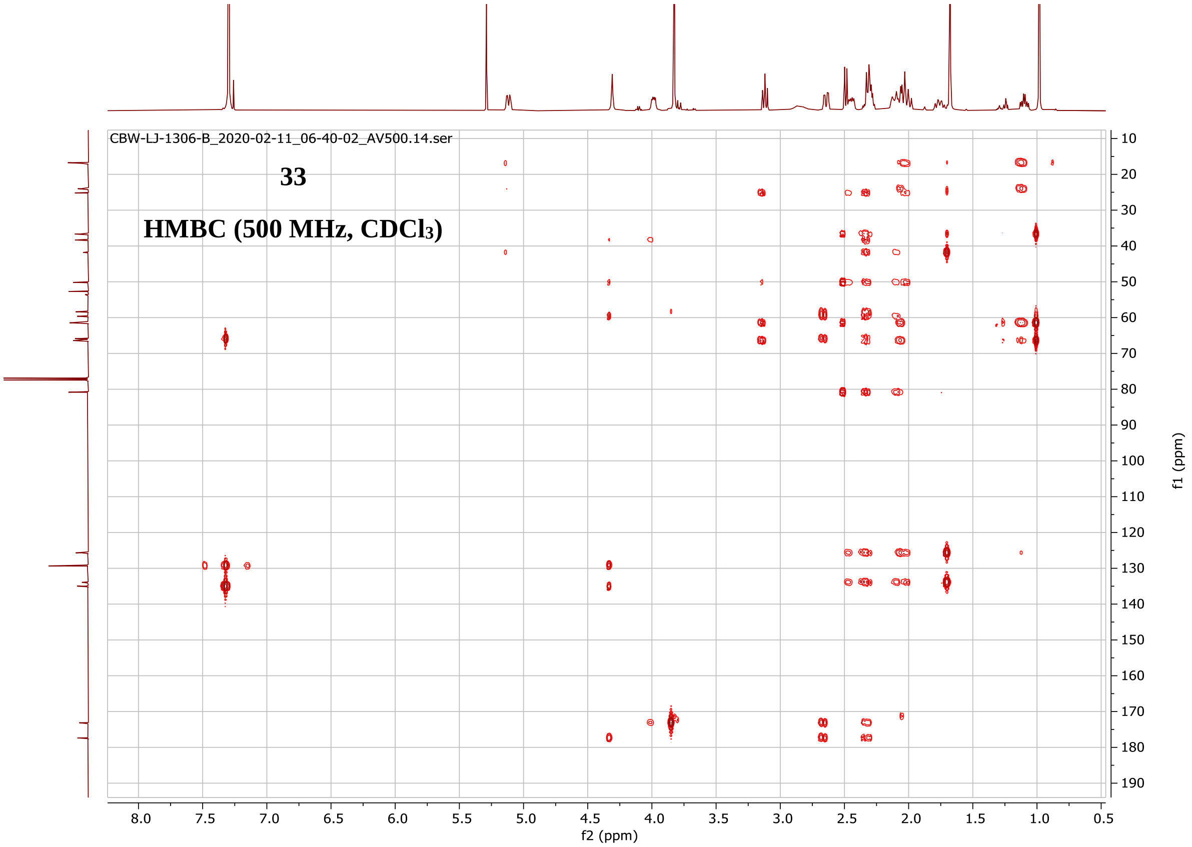
CBW-LJ-1306-B_2020-02-11_06-40-02_AV500.14.ser
33
HMBC (500 MHz, CDCl3)
f2 (ppm)
f1 (ppm)

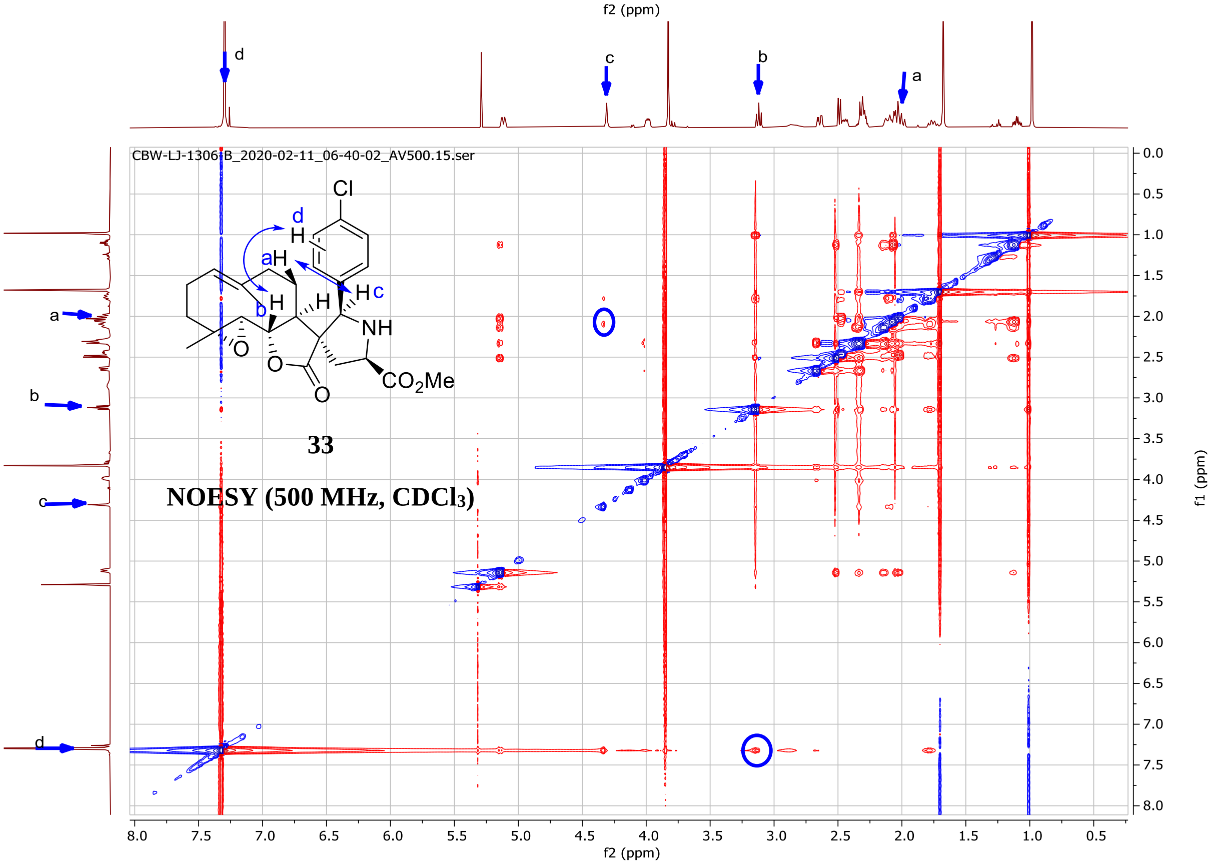
CBW-LJ-1306-B_2020-02-11_06-40-02_AV500.15.ser
Cl
d
H
aH
b
H
H
H c
NH
O
O
O
CO2Me
33
NOESY (500 MHz, CDCl3)
a
b
c
d
f2 (ppm)
f1 (ppm)

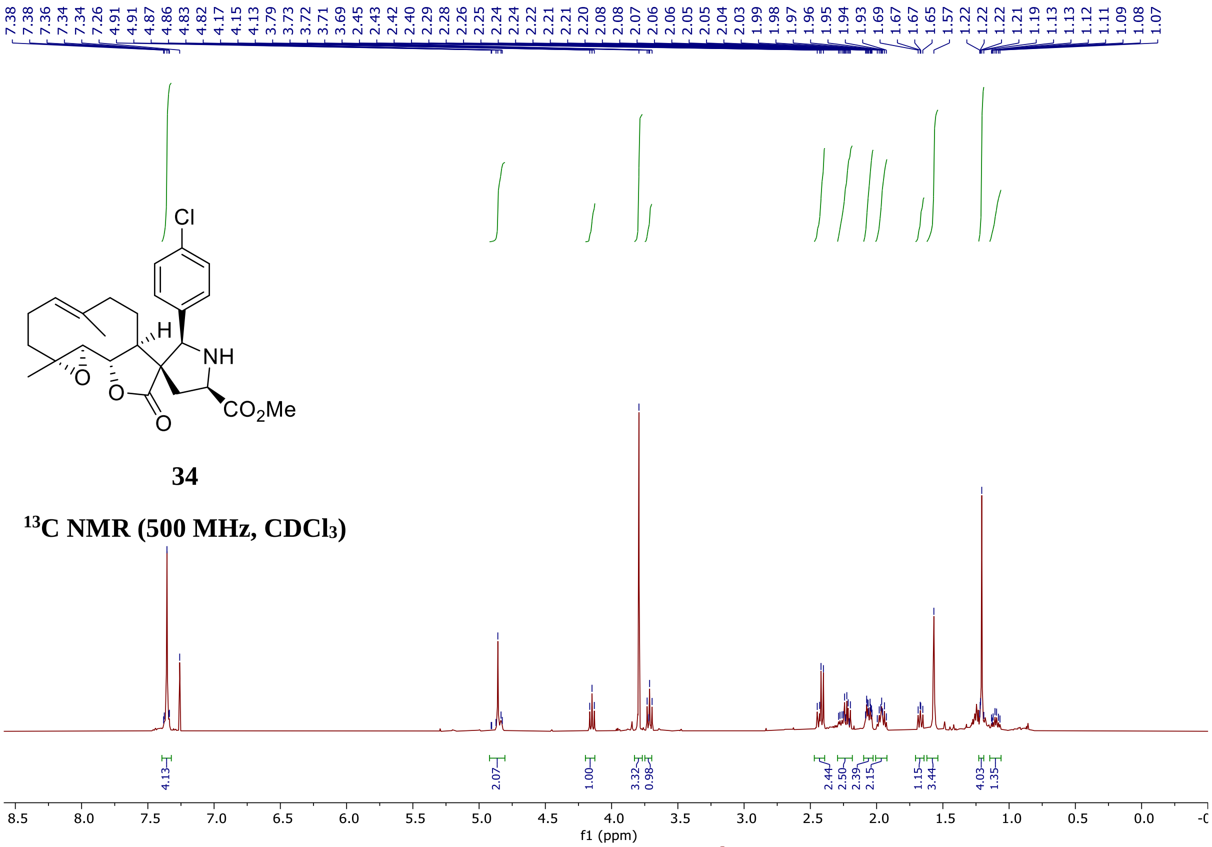
7.38 7.38 7.36 7.34 7.34 7.26 4.91 4.91 4.87 4.86 4.83 4.82 4.17 4.15 4.13 3.79 3.73 3.72 3.71 3.69 2.45 2.43 2.42 2.40 2.29 2.28 2.26 2.25 2.24 2.24 2.22 2.22 2.21 2.20 2.08 2.08 2.07 2.06 2.06 2.05 2.05 2.04 2.03 1.99 1.98 1.97 1.96 1.95 1.94 1.93 1.69 1.67 1.65 1.57 1.22 1.22 1.21 1.19 1.13 1.12 1.11 1.09 1.08 1.07
Cl
H
NH
O
O
O
CO2Me
34
13C NMR (500 MHz, CDCl3)
4.13
2.07
1.00
3.32
0.98
2.44
2.50
2.39
2.15
1.15
3.44
4.03
1.35
8.5 8.0 7.5 7.0 6.5 6.0 5.5 5.0 4.5 4.0 3.5 3.0 2.5 2.0 1.5 1.0 0.5 0.0
f1 (ppm)

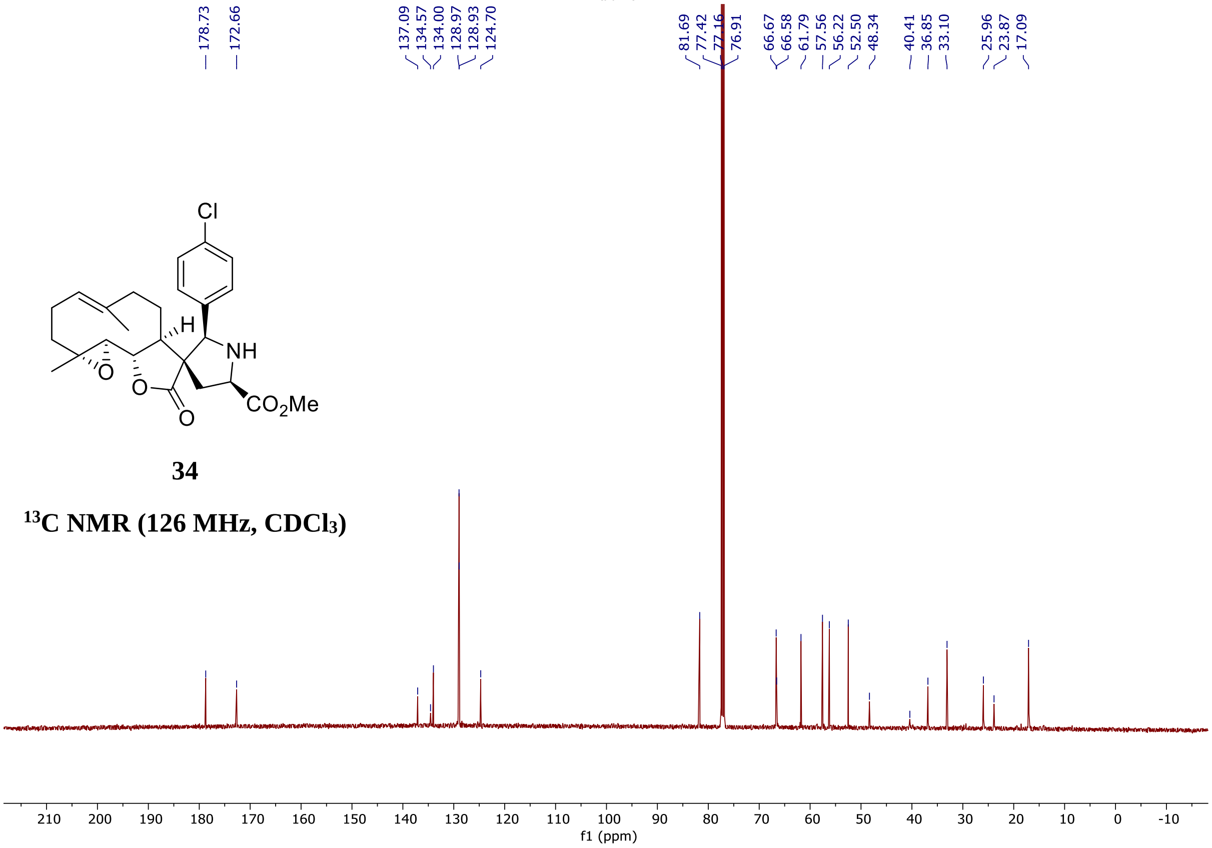
178.73
172.66
137.09 134.57 134.00 128.97 128.93 124.70
81.69 77.42 77.16 76.91
66.67 66.58 61.79 57.56 56.22 52.50 48.34
40.41 36.85 33.10
25.96 23.87 17.09
Cl
H
NH
O
O
O
CO2Me
34
13C NMR (126 MHz, CDCl3)
210 200 190 180 170 160 150 140 130 120 110 100 90 80 70 60 50 40 30 20 10 0 -10
f1 (ppm)

34

^{1}H - ^{1}H COSY (500 MHz, $CDCl_3$)

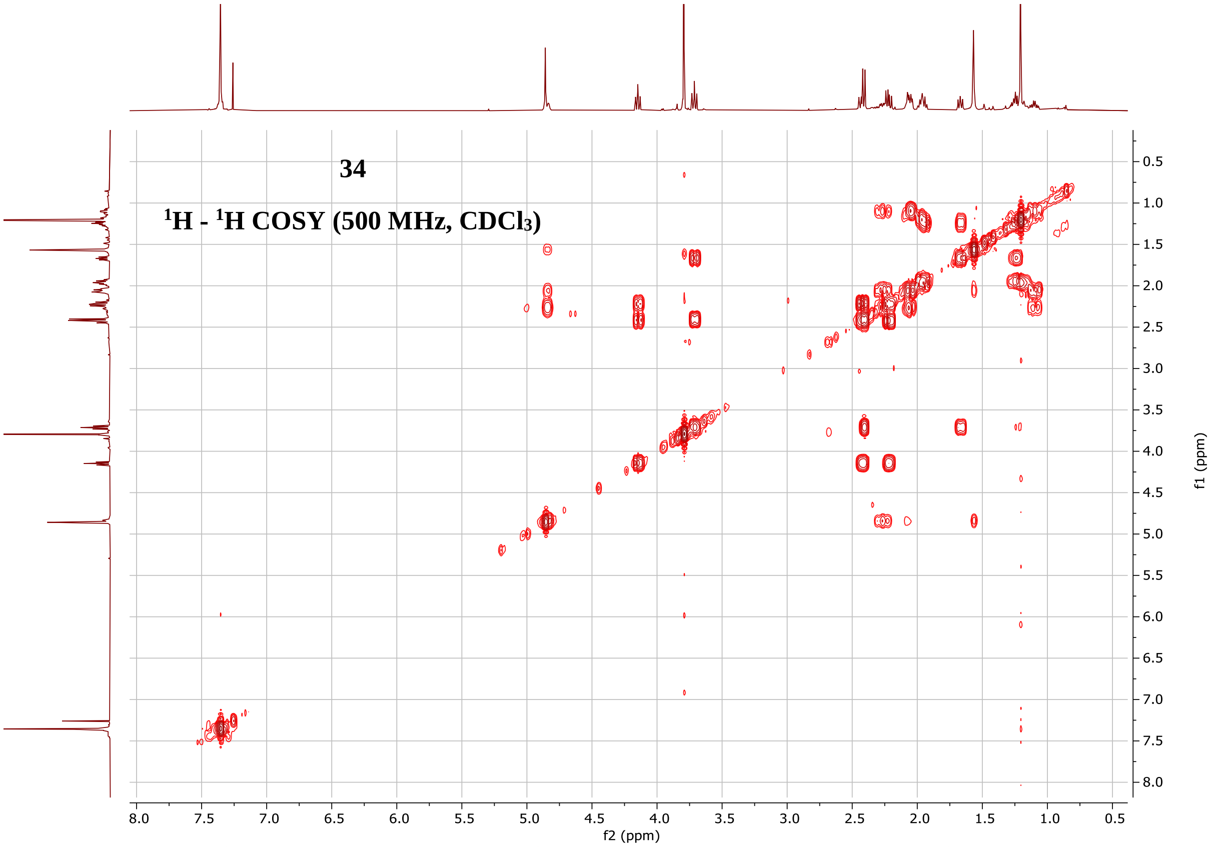

34

HSQC (500 MHz, $CDCl_3$)

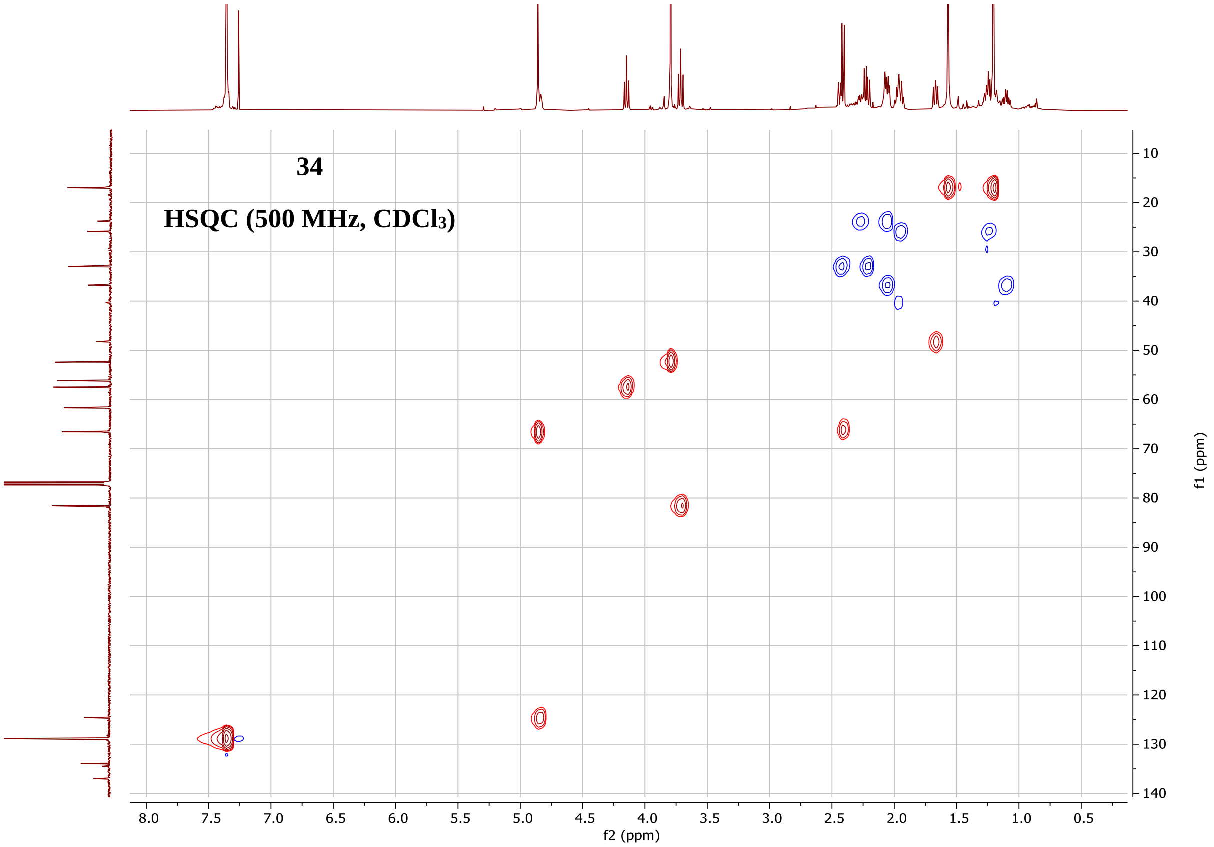

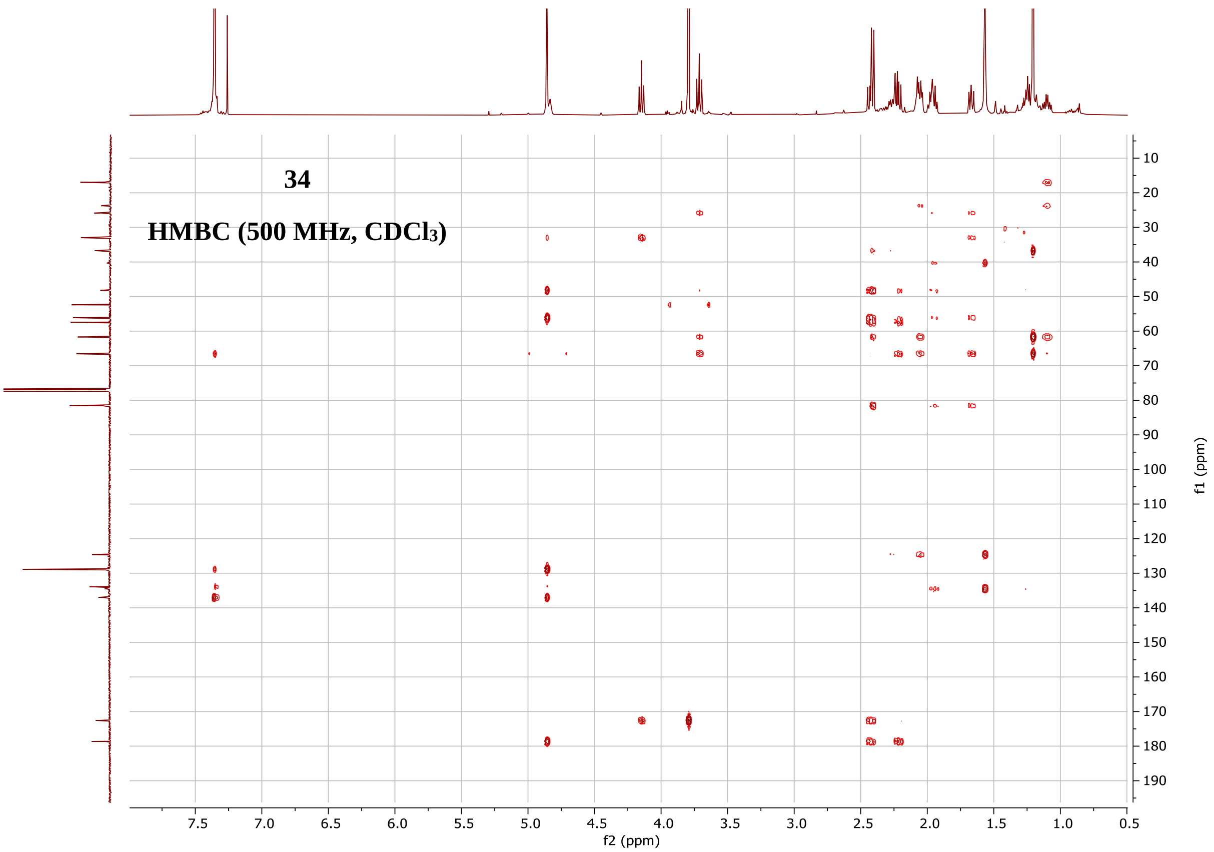
34
HMBC (500 MHz, CDCl3)
f2 (ppm)
f1 (ppm)

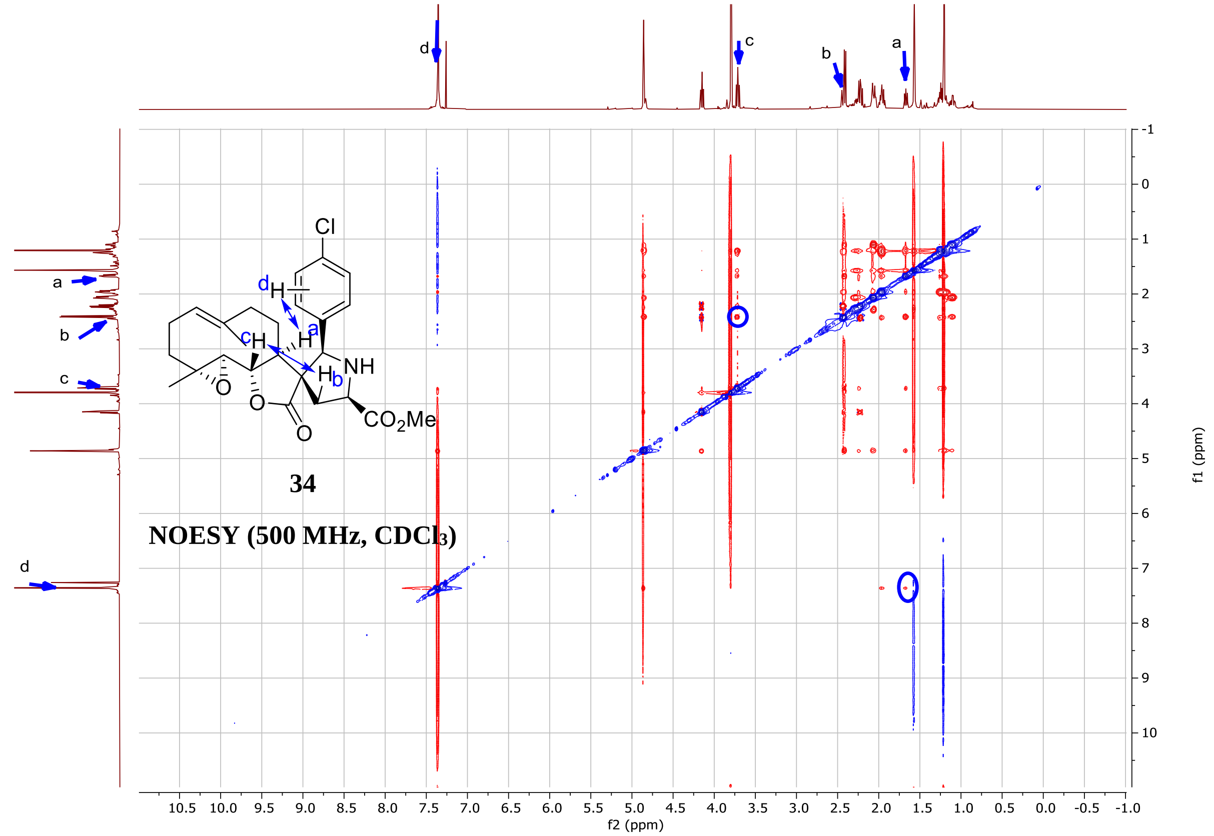
d
c
b
a
Cl
NH
CO2Me
O
34
NOESY (500 MHz, CDCl3)
f2 (ppm)
f1 (ppm)

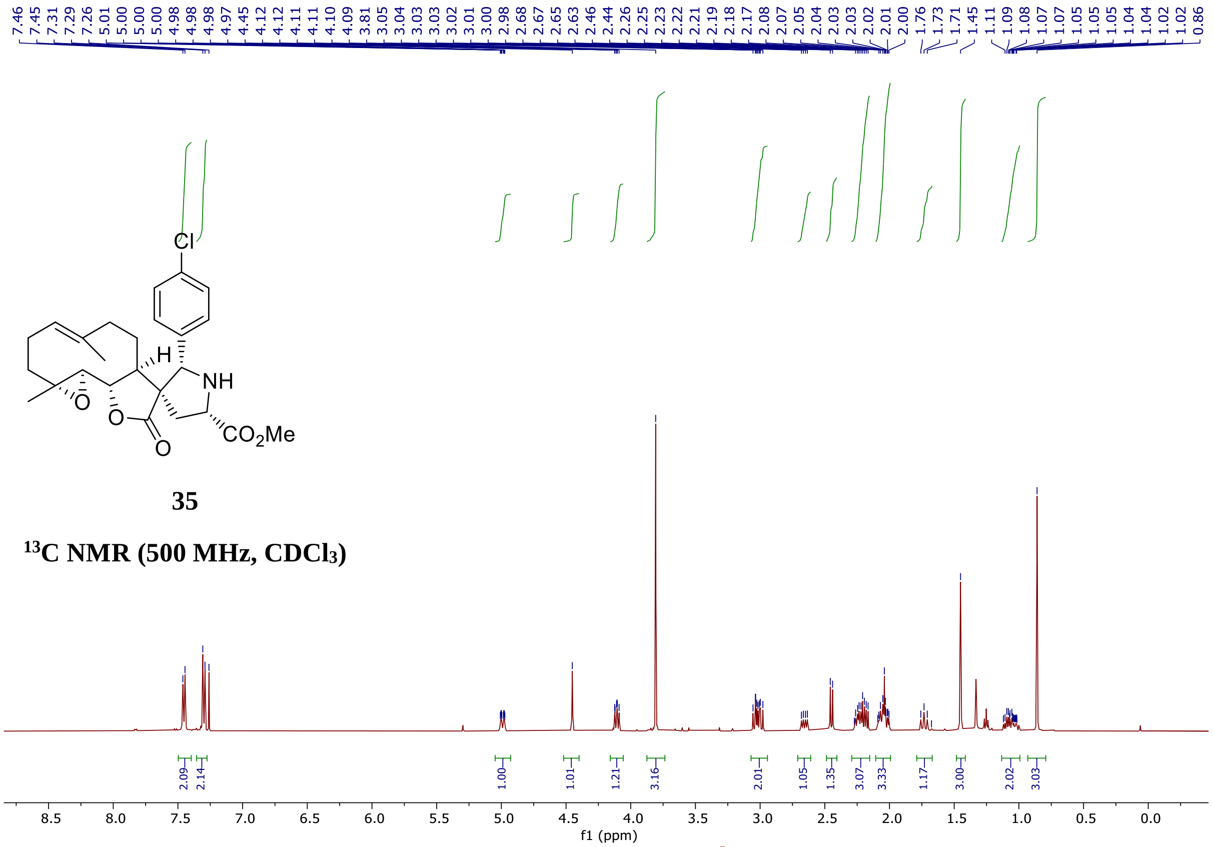
7.46 7.45 7.31 7.29 7.26 5.01 5.00 5.00 4.98 4.98 4.97 4.45 4.12 4.12 4.11 4.11 4.10 4.09 3.81 3.05 3.04 3.03 3.03 3.02 3.01 3.00 2.98 2.68 2.67 2.65 2.63 2.46 2.44 2.26 2.25 2.23 2.22 2.21 2.19 2.18 2.17 2.08 2.07 2.05 2.04 2.03 2.03 2.02 2.01 2.00 1.76 1.73 1.71 1.45 1.11 1.09 1.08 1.07 1.07 1.05 1.05 1.05 1.04 1.04 1.02 1.02 0.86
Cl
H
NH
O
O
O
CO_2Me
35
^{13}C NMR (500 MHz, $CDCl_3$)
2.09 2.14 1.00 1.01 1.21 3.16 2.01 1.05 1.35 3.07 3.33 1.17 3.00 2.02 3.03
8.5 8.0 7.5 7.0 6.5 6.0 5.5 5.0 4.5 4.0 3.5 3.0 2.5 2.0 1.5 1.0 0.5 0.0
f1 (ppm)

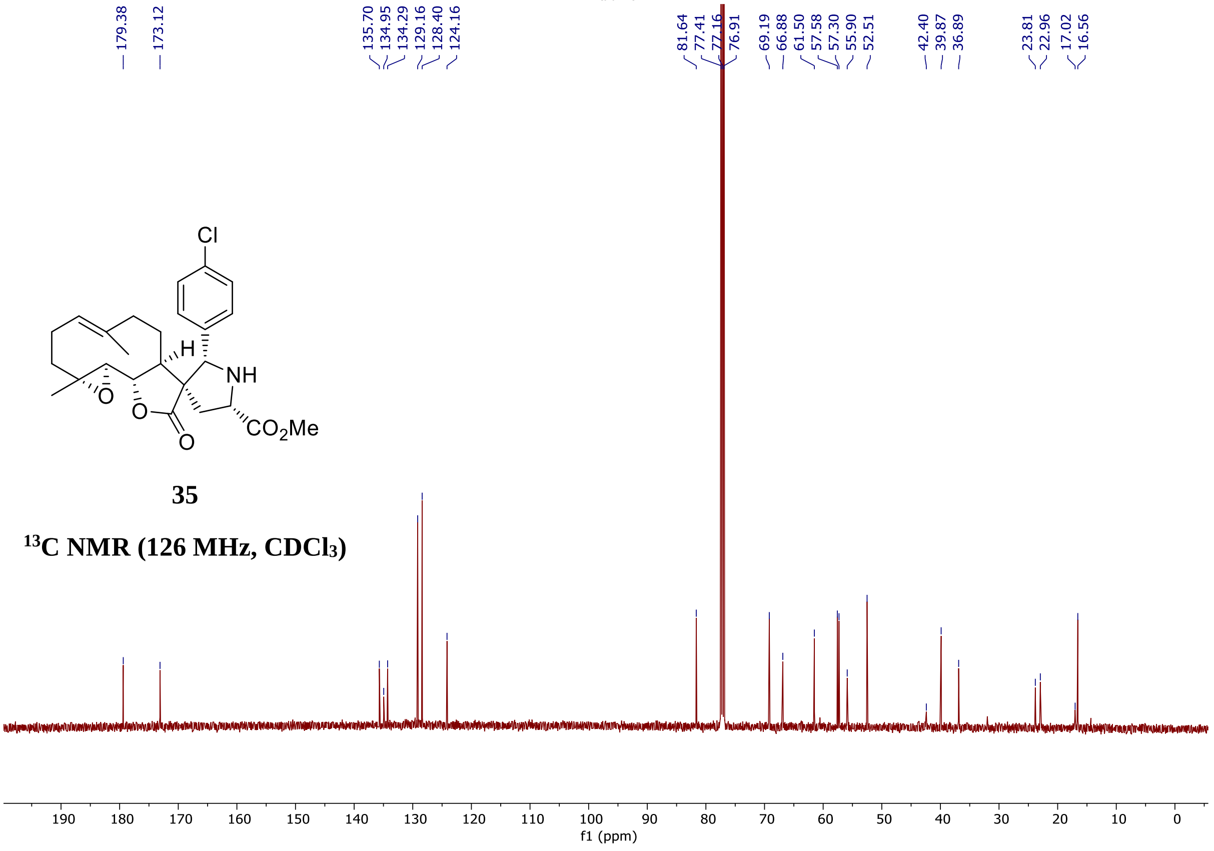
179.38 173.12 135.70 134.95 134.29 129.16 128.40 124.16 81.64 77.41 77.16 76.91 69.19 66.88 61.50 57.58 57.30 55.90 52.51 42.40 39.87 36.89 23.81 22.96 17.02 16.56
Cl
H
NH
O
O
O
CO_2Me
35
^{13}C NMR (126 MHz, $CDCl_3$)
190 180 170 160 150 140 130 120 110 100 90 80 70 60 50 40 30 20 10 0
f1 (ppm)

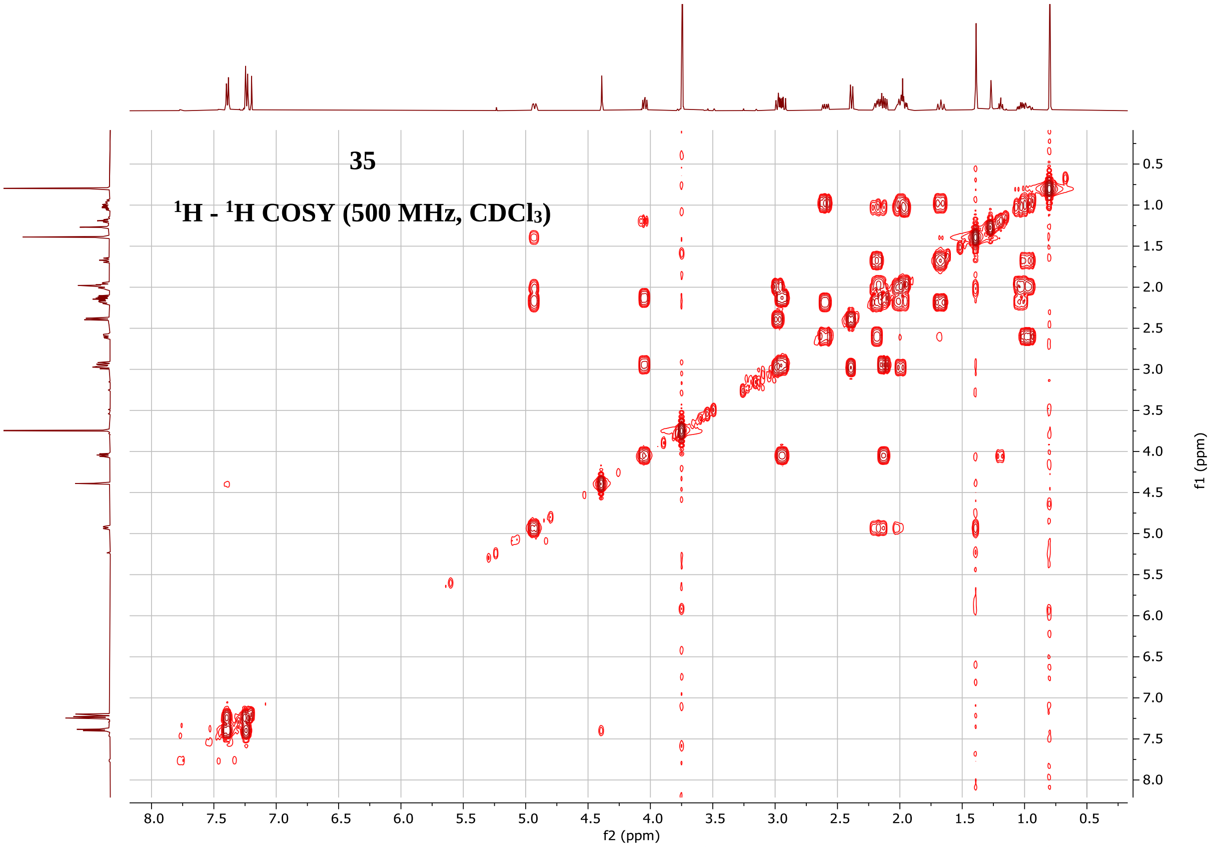
35
^{1}H - ^{1}H COSY (500 MHz, CDCl$_3$)
f2 (ppm)
f1 (ppm)

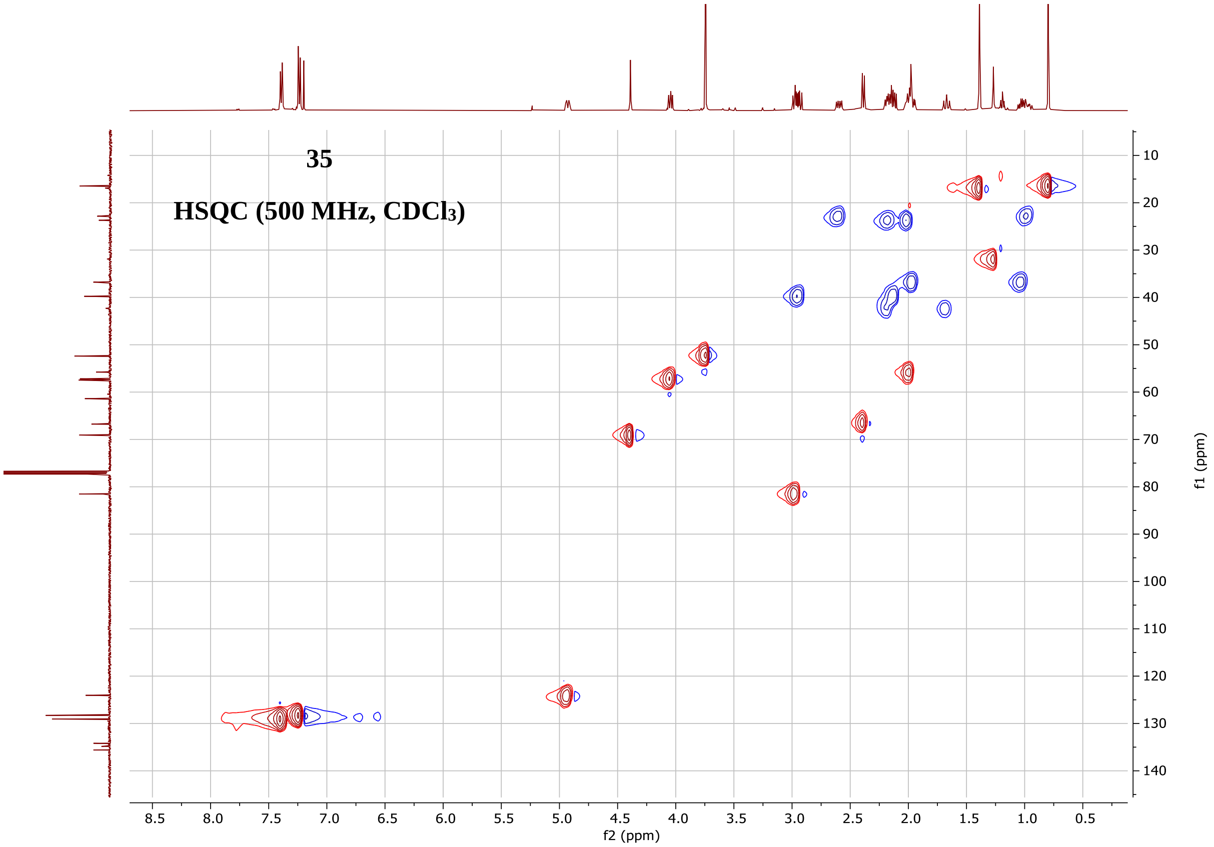
35
HSQC (500 MHz, CDCl$_3$)
f2 (ppm)
f1 (ppm)

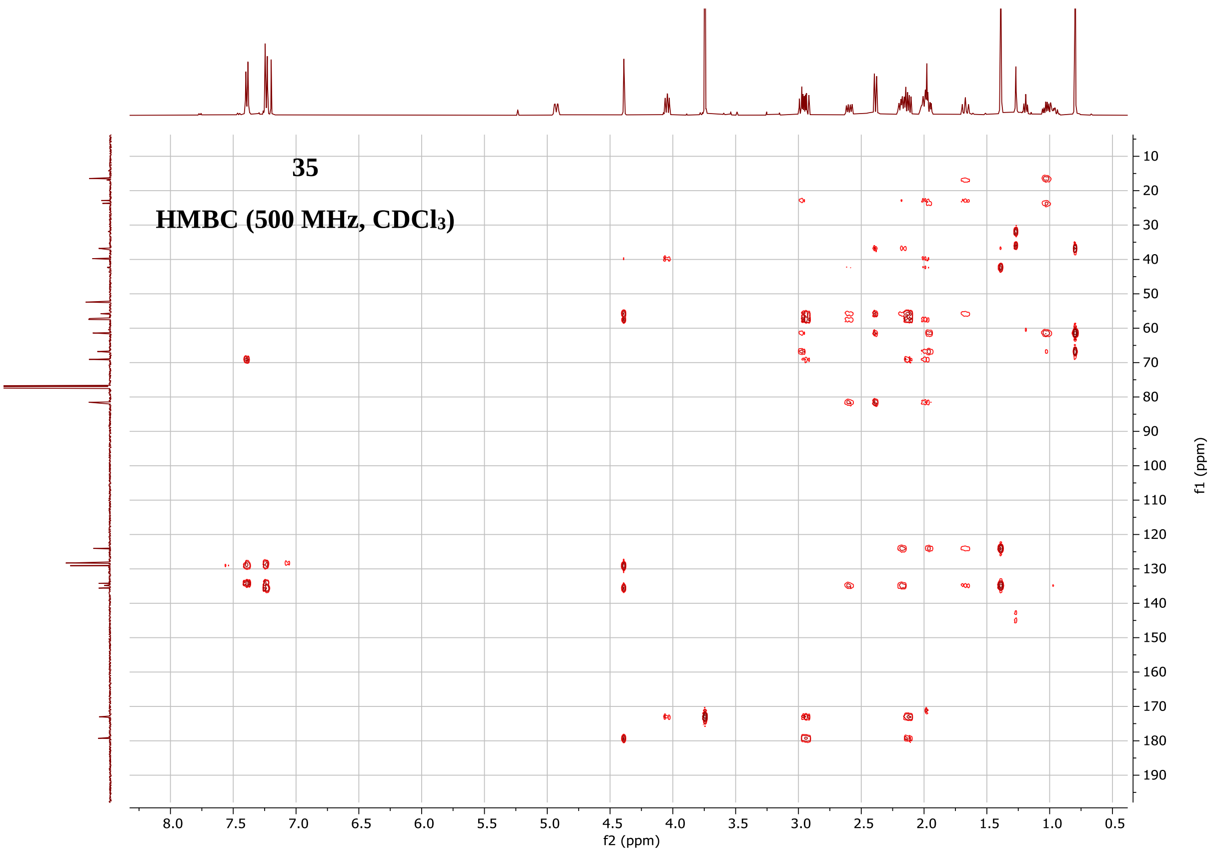
35
HMBC (500 MHz, CDCl3)
f2 (ppm)
f1 (ppm)

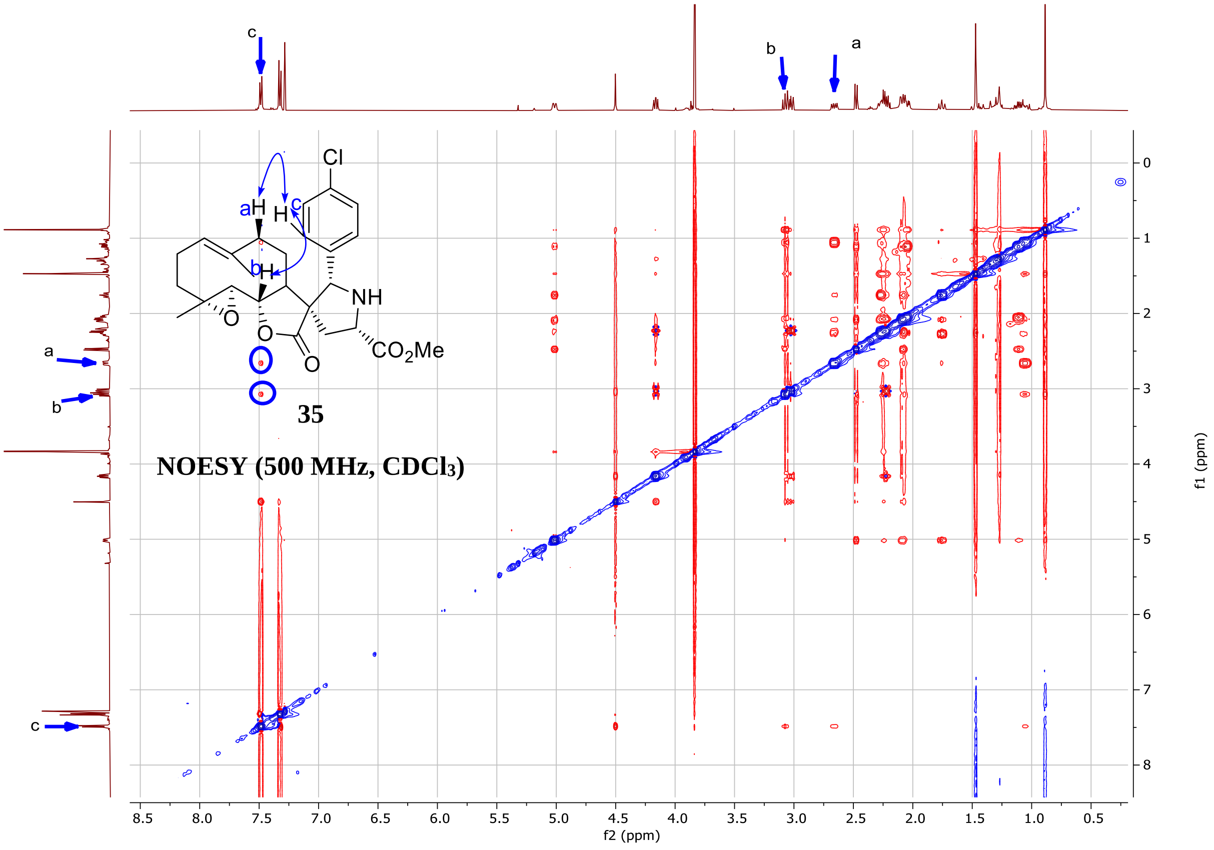
Cl
aH
H
c
bH
NH
O
O
O
CO2Me
35
a
b
c
NOESY (500 MHz, CDCl3)
f2 (ppm)
f1 (ppm)

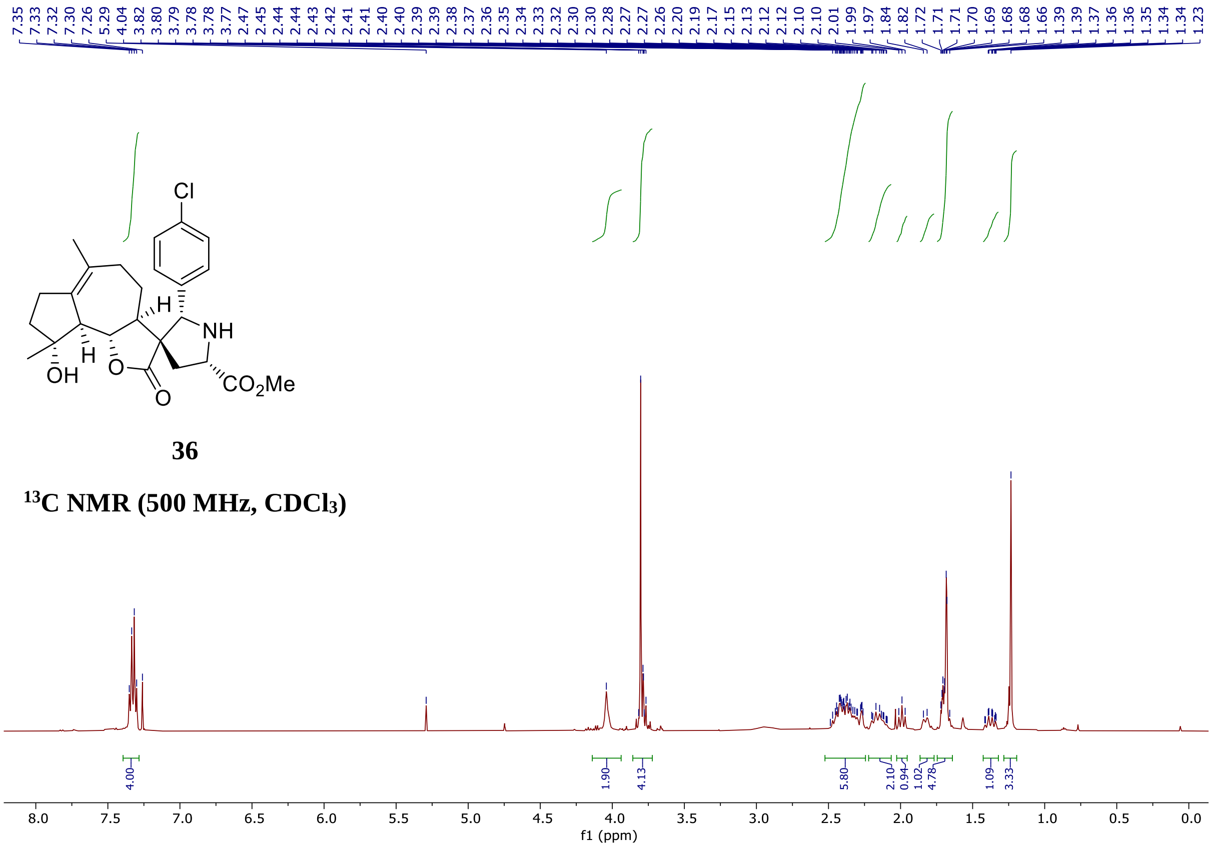
7.35 7.33 7.32 7.30 7.26 5.29 4.04 3.82 3.80 3.79 3.78 3.78 3.77 2.47 2.45 2.44 2.44 2.43 2.42 2.41 2.41 2.40 2.40 2.39 2.39 2.38 2.37 2.36 2.35 2.34 2.33 2.32 2.30 2.28 2.27 2.27 2.26 2.20 2.19 2.17 2.15 2.13 2.12 2.12 2.10 2.10 2.01 1.99 1.97 1.84 1.82 1.72 1.71 1.71 1.70 1.69 1.68 1.66 1.39 1.39 1.37 1.36 1.36 1.35 1.34 1.23
Cl
H
NH
H
OH
O
O
CO_2Me
36
^{13}C NMR (500 MHz, $CDCl_3$)
4.00
1.90
4.13
5.80
2.10
0.94
1.02
4.78
1.09
3.33
8.0 7.5 7.0 6.5 6.0 5.5 5.0 4.5 4.0 3.5 3.0 2.5 2.0 1.5 1.0 0.5 0.0
f1 (ppm)

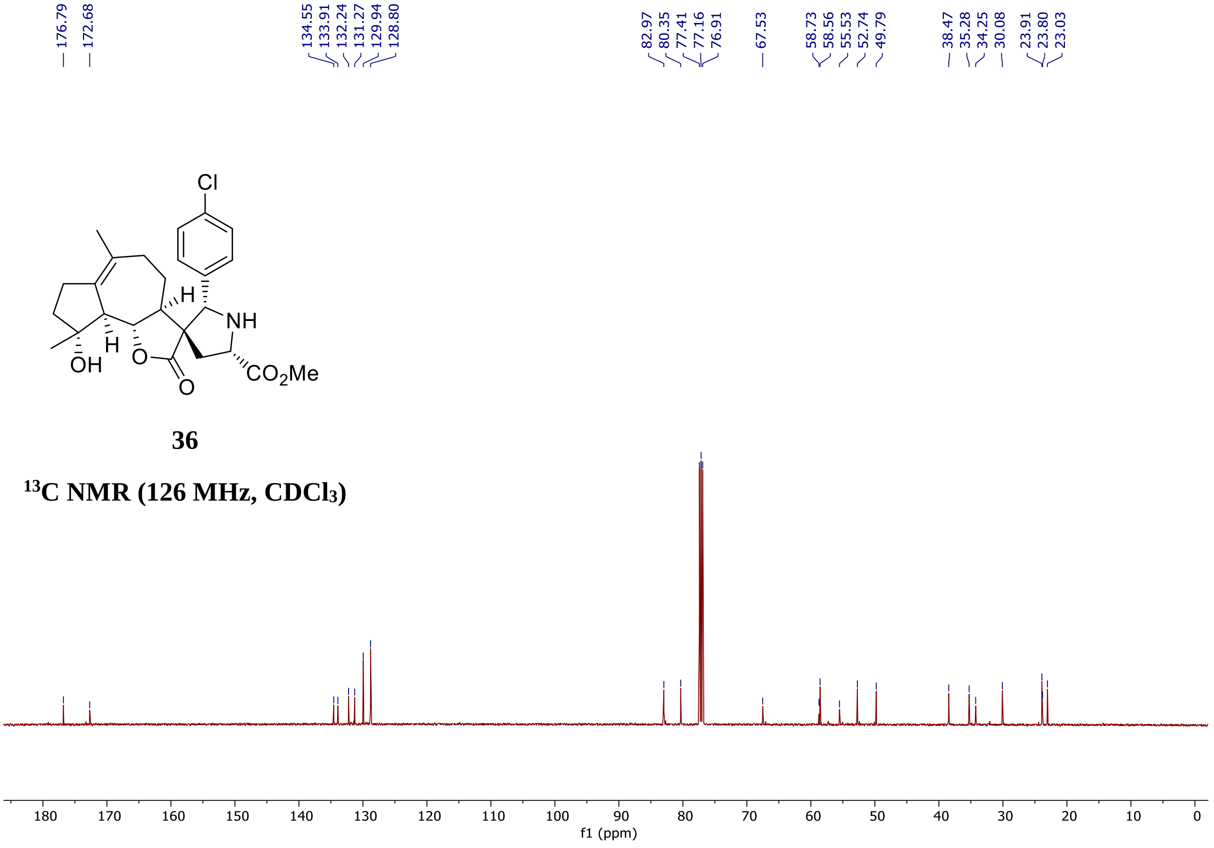
176.79 172.68 134.55 133.91 132.24 131.27 129.94 128.80 82.97 80.35 77.41 77.16 76.91 67.53 58.73 58.56 55.53 52.74 49.79 38.47 35.28 34.25 30.08 23.91 23.80 23.03
Cl
H
NH
H
OH
O
O
CO_2Me
36
^{13}C NMR (126 MHz, $CDCl_3$)
180 170 160 150 140 130 120 110 100 90 80 70 60 50 40 30 20 10 0
f1 (ppm)

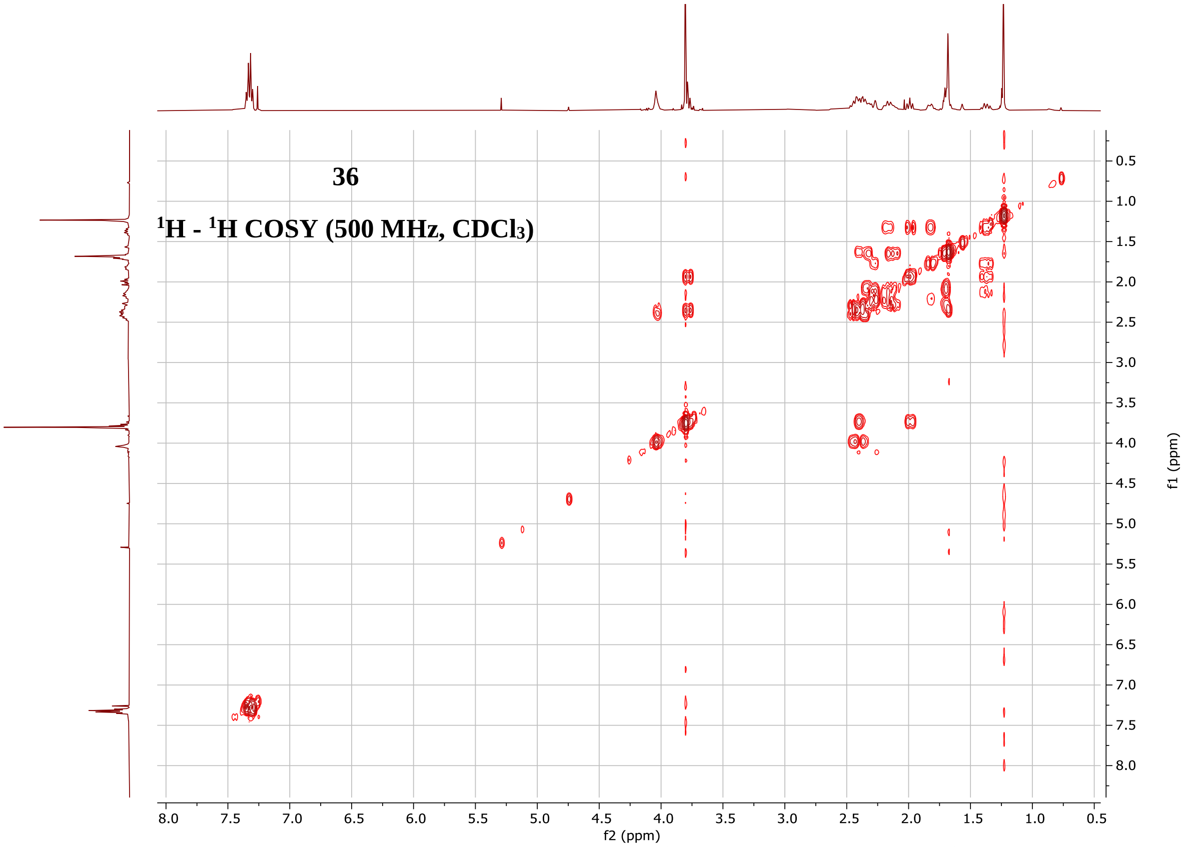
36
¹H - ¹H COSY (500 MHz, CDCl₃)
f2 (ppm)
f1 (ppm)

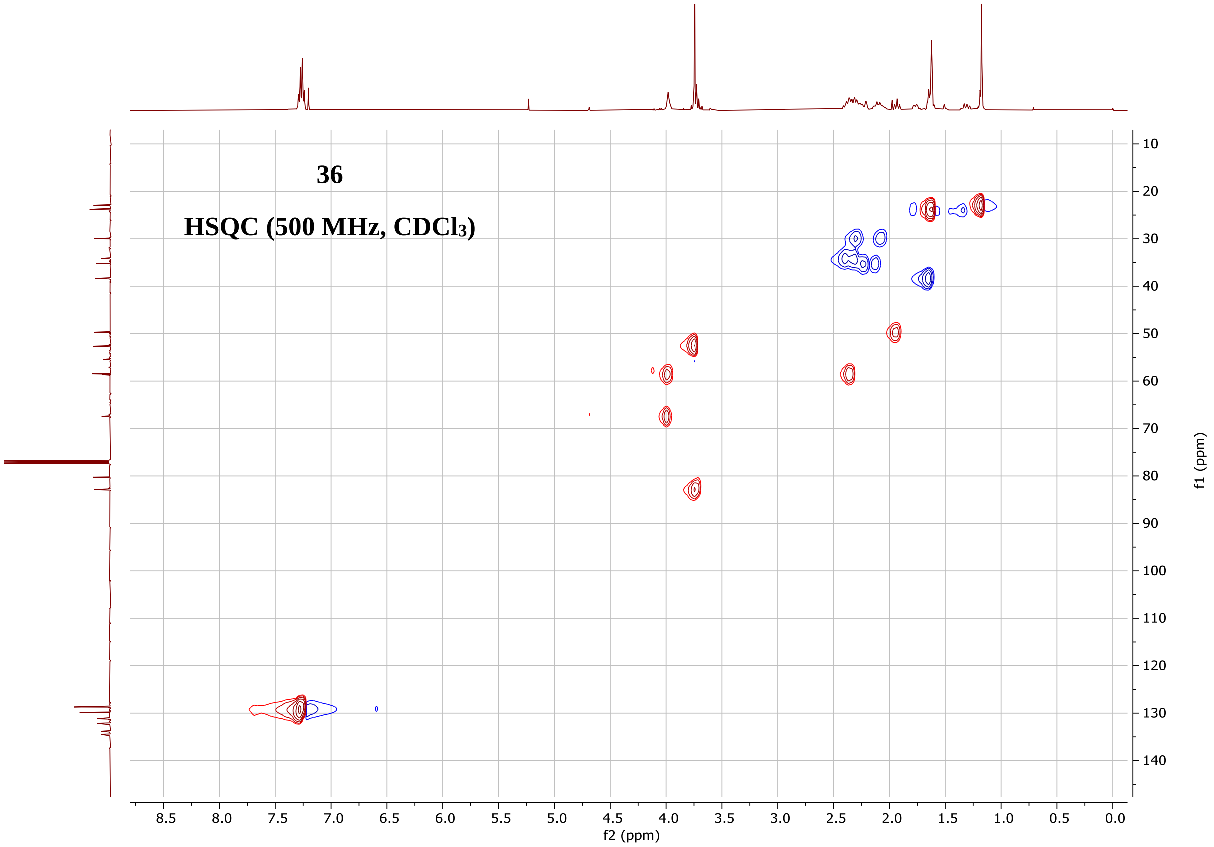
36
HSQC (500 MHz, CDCl₃)
f2 (ppm)
f1 (ppm)

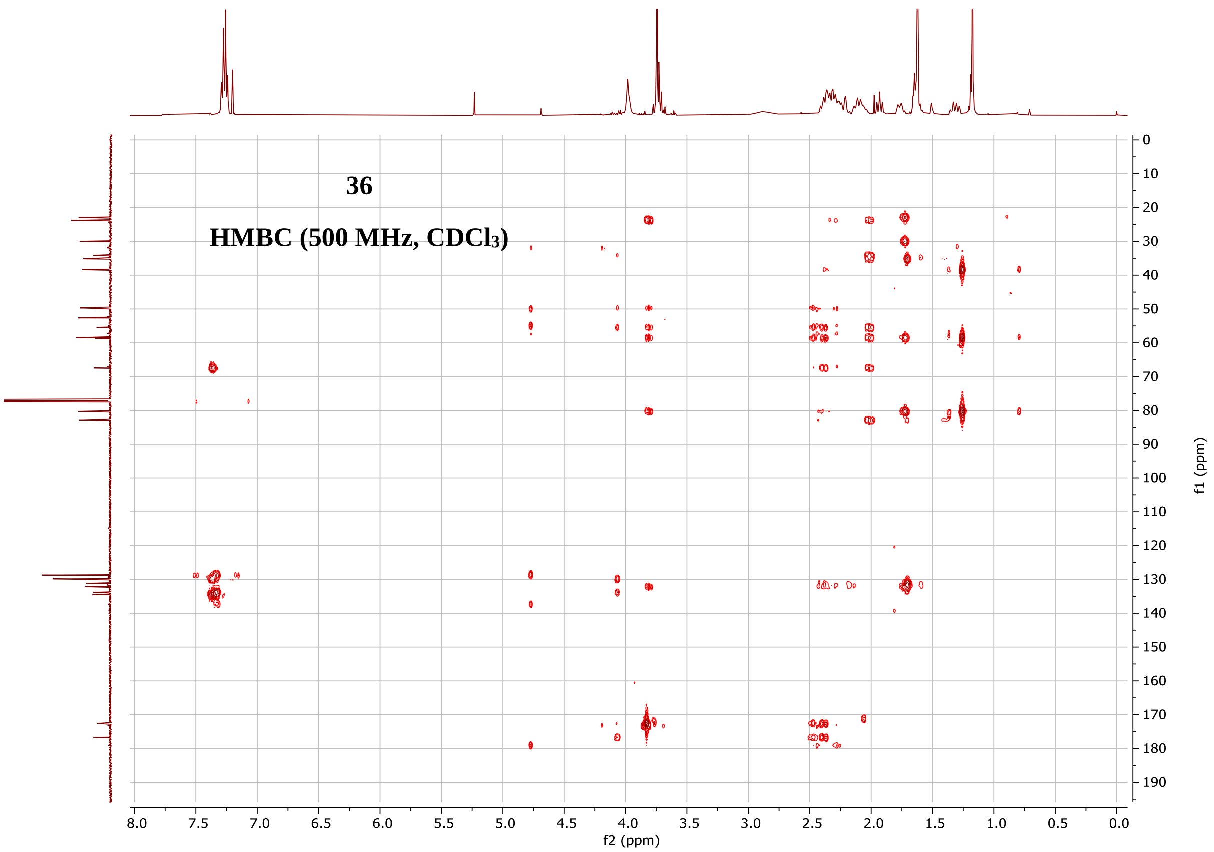
36
HMBC (500 MHz, CDCl3)
f1 (ppm)
f2 (ppm)

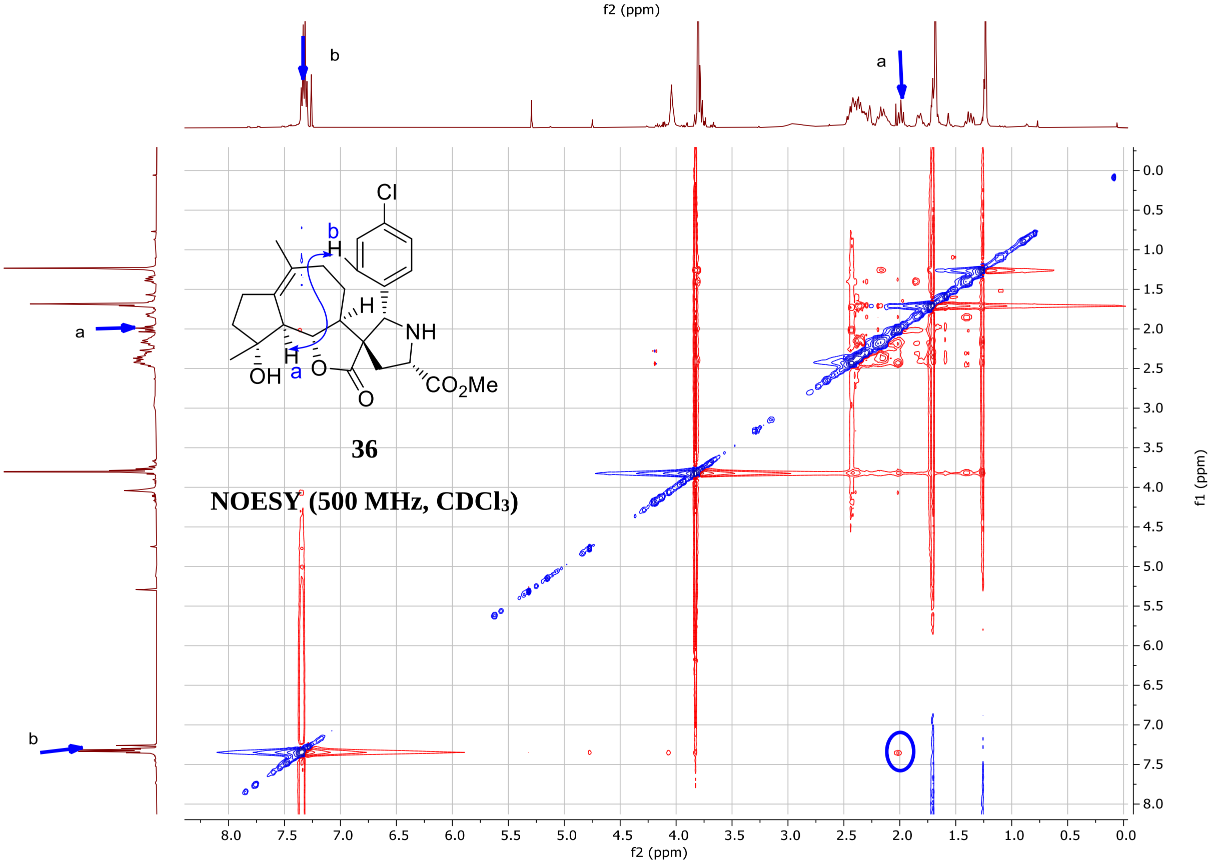
b
a
Cl
H
NH
OH
O
CO2Me
36
NOESY (500 MHz, CDCl3)
f1 (ppm)
f2 (ppm)

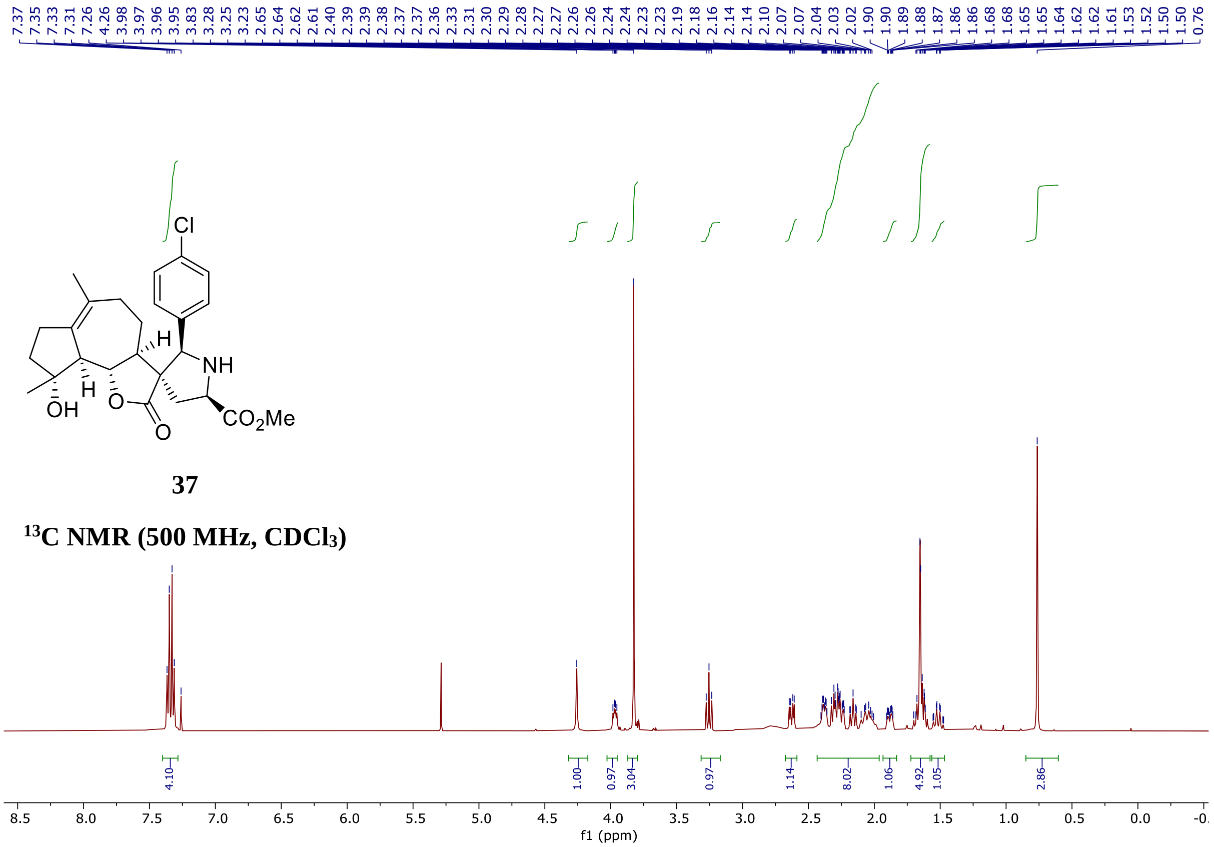
7.37 7.35 7.33 7.31 7.26 4.26 3.98 3.97 3.96 3.95 3.83 3.28 3.25 3.23 2.65 2.64 2.62 2.61 2.40 2.39 2.39 2.38 2.37 2.37 2.36 2.33 2.31 2.30 2.29 2.28 2.27 2.27 2.26 2.26 2.24 2.24 2.23 2.23 2.19 2.18 2.16 2.14 2.14 2.10 2.07 2.04 2.03 2.03 2.02 1.90 1.90 1.89 1.88 1.87 1.86 1.68 1.68 1.65 1.65 1.64 1.62 1.62 1.61 1.53 1.52 1.50 1.50 0.76
Cl
H
NH
H
OH
O
O
CO2Me
37
13C NMR (500 MHz, CDCl3)
4.10 1.00 0.97 3.04 0.97 1.14 8.02 1.06 4.92 1.05 2.86
8.5 8.0 7.5 7.0 6.5 6.0 5.5 5.0 4.5 4.0 3.5 3.0 2.5 2.0 1.5 1.0 0.5 0.0 -0.
f1 (ppm)

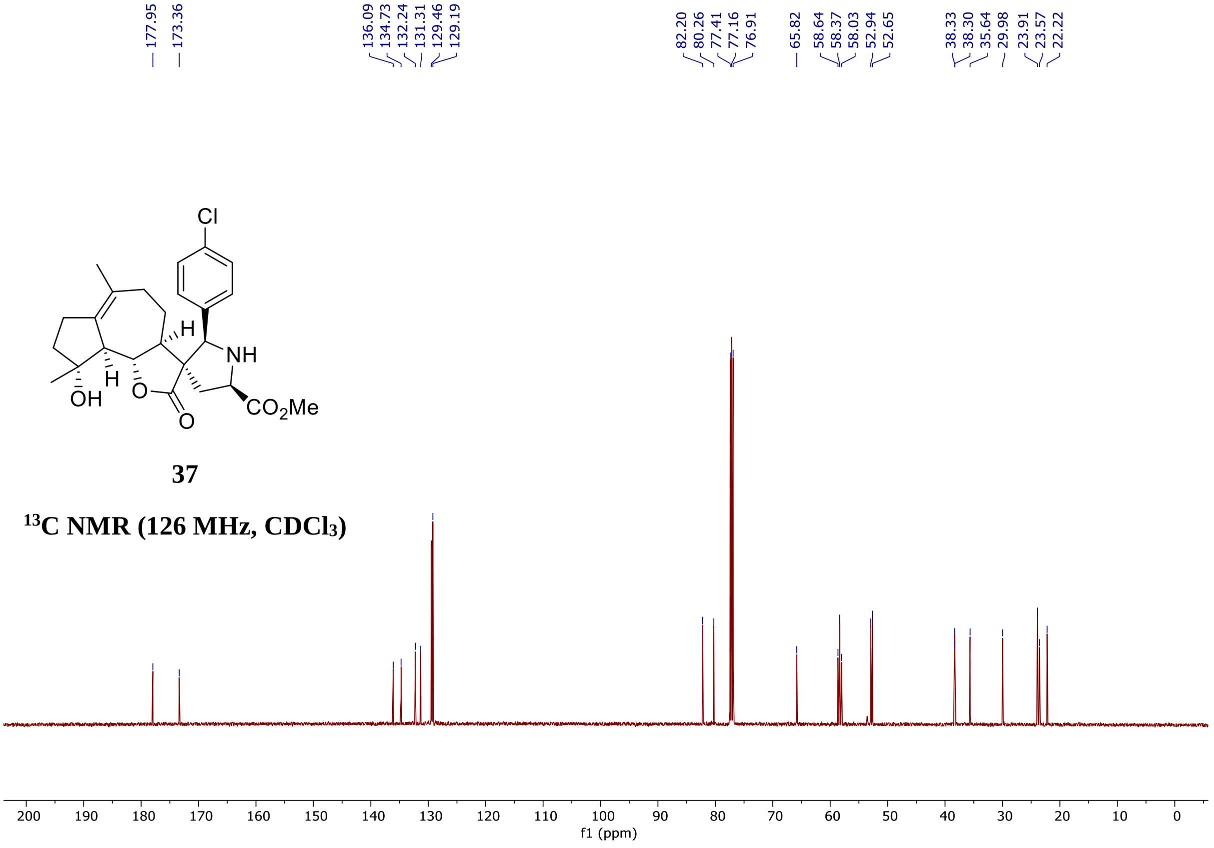
177.95 173.36 136.09 134.73 132.24 131.31 129.46 129.19 82.20 80.26 77.41 77.16 76.91 65.82 58.64 58.37 58.03 52.94 52.65 38.33 38.30 35.64 29.98 23.91 23.57 22.22
Cl
H
NH
H
OH
O
O
CO2Me
37
13C NMR (126 MHz, CDCl3)
200 190 180 170 160 150 140 130 120 110 100 90 80 70 60 50 40 30 20 10 0
f1 (ppm)

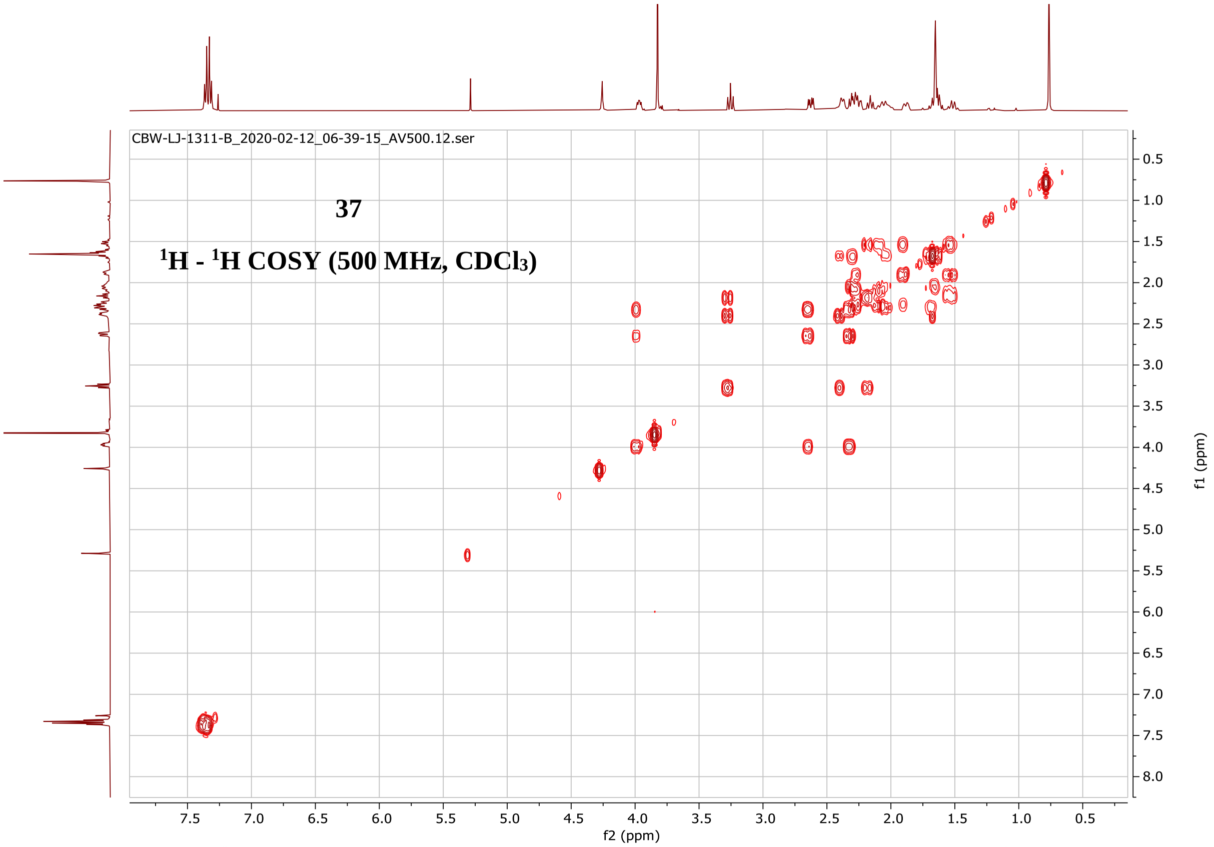
CBW-LJ-1311-B_2020-02-12_06-39-15_AV500.12.ser
37
^{1}H - ^{1}H COSY (500 MHz, CDCl$_3$)
f2 (ppm)
f1 (ppm)

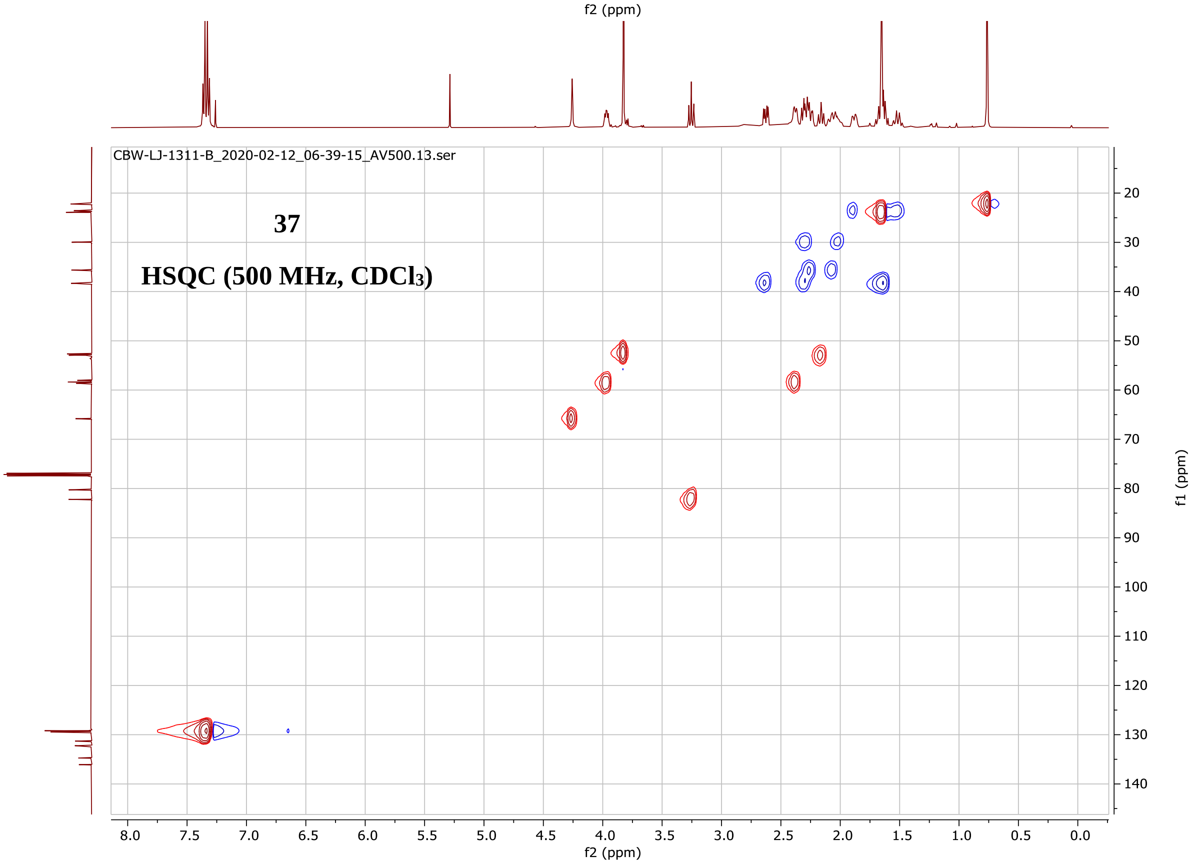
CBW-LJ-1311-B_2020-02-12_06-39-15_AV500.13.ser
37
HSQC (500 MHz, CDCl$_3$)
f2 (ppm)
f1 (ppm)

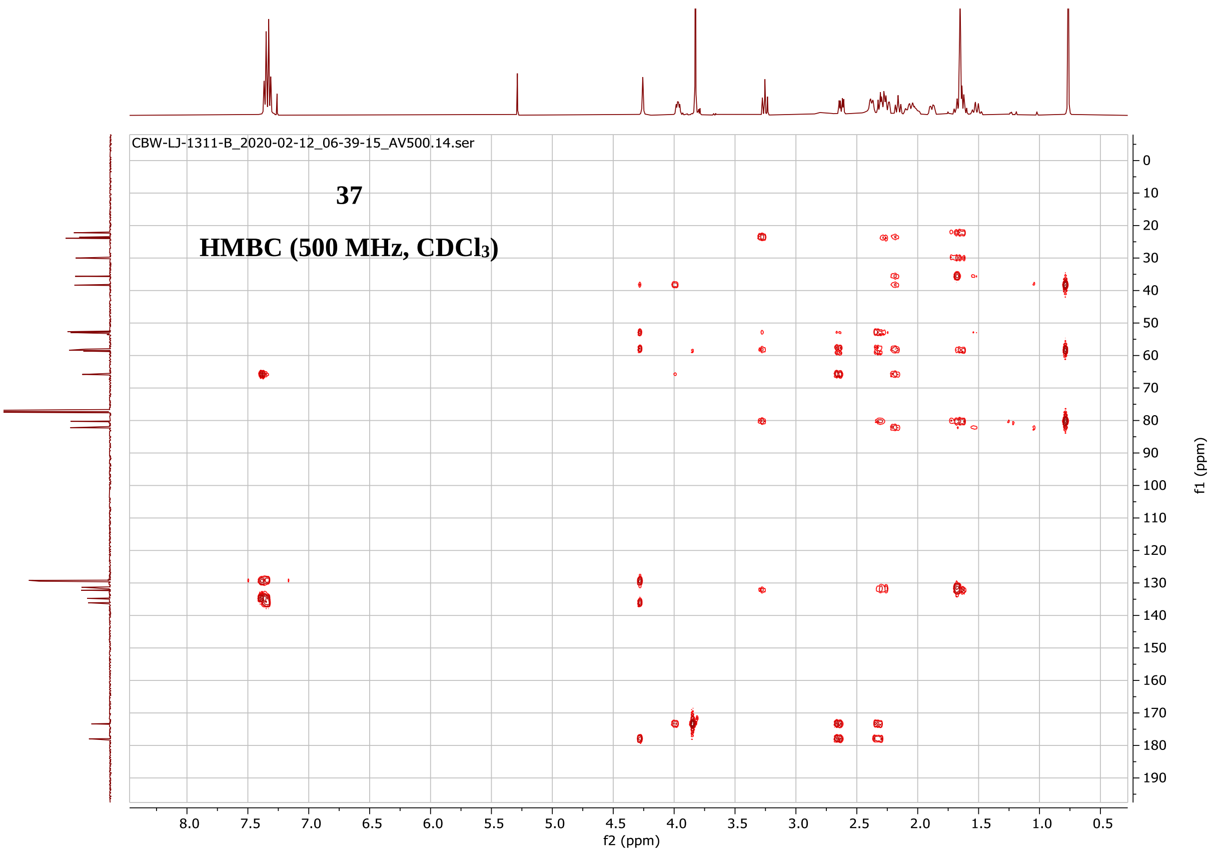
CBW-LJ-1311-B_2020-02-12_06-39-15_AV500.14.ser
37
HMBC (500 MHz, CDCl3)
f2 (ppm)
f1 (ppm)

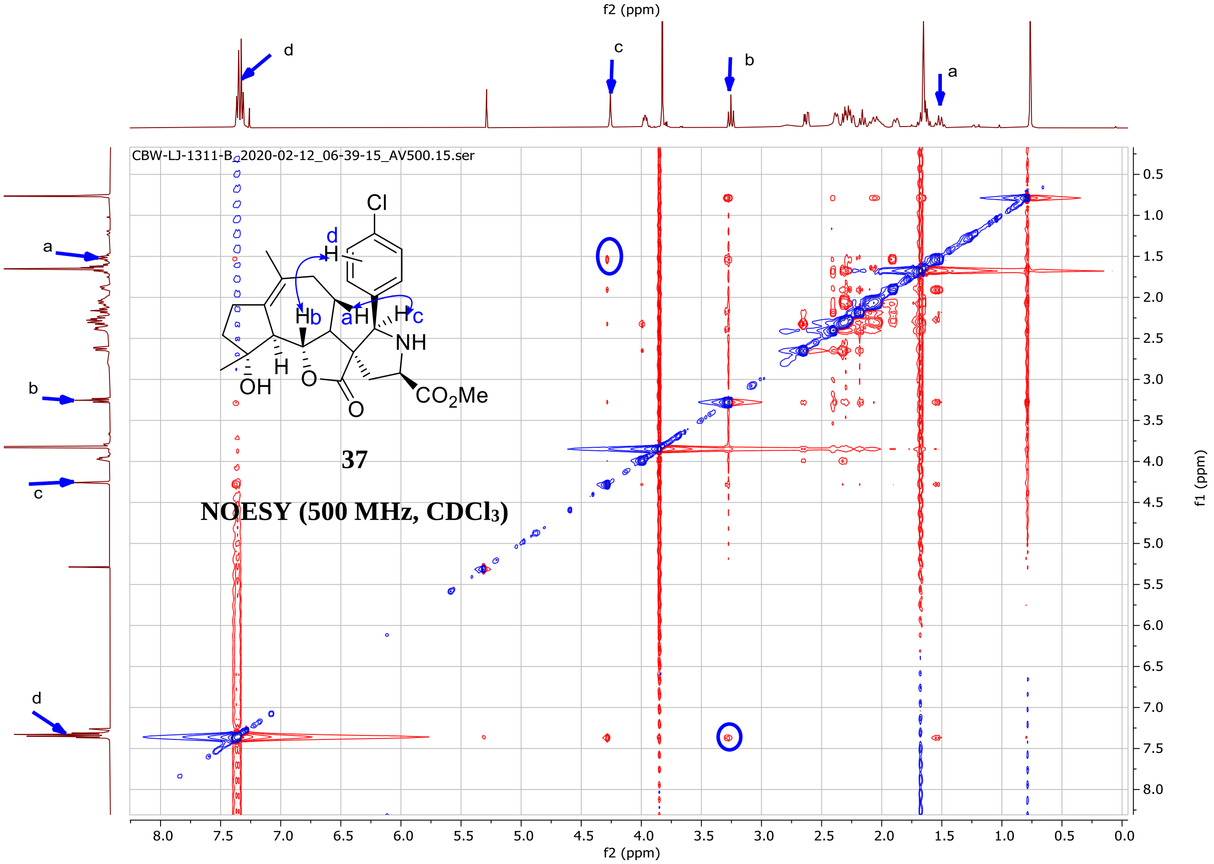
CBW-LJ-1311-B_2020-02-12_06-39-15_AV500.15.ser
Cl
d
H
Hb
aH
Hc
NH
H
OH
O
O
CO2Me
37
NOESY (500 MHz, CDCl3)
a
b
c
d
f2 (ppm)
f1 (ppm)

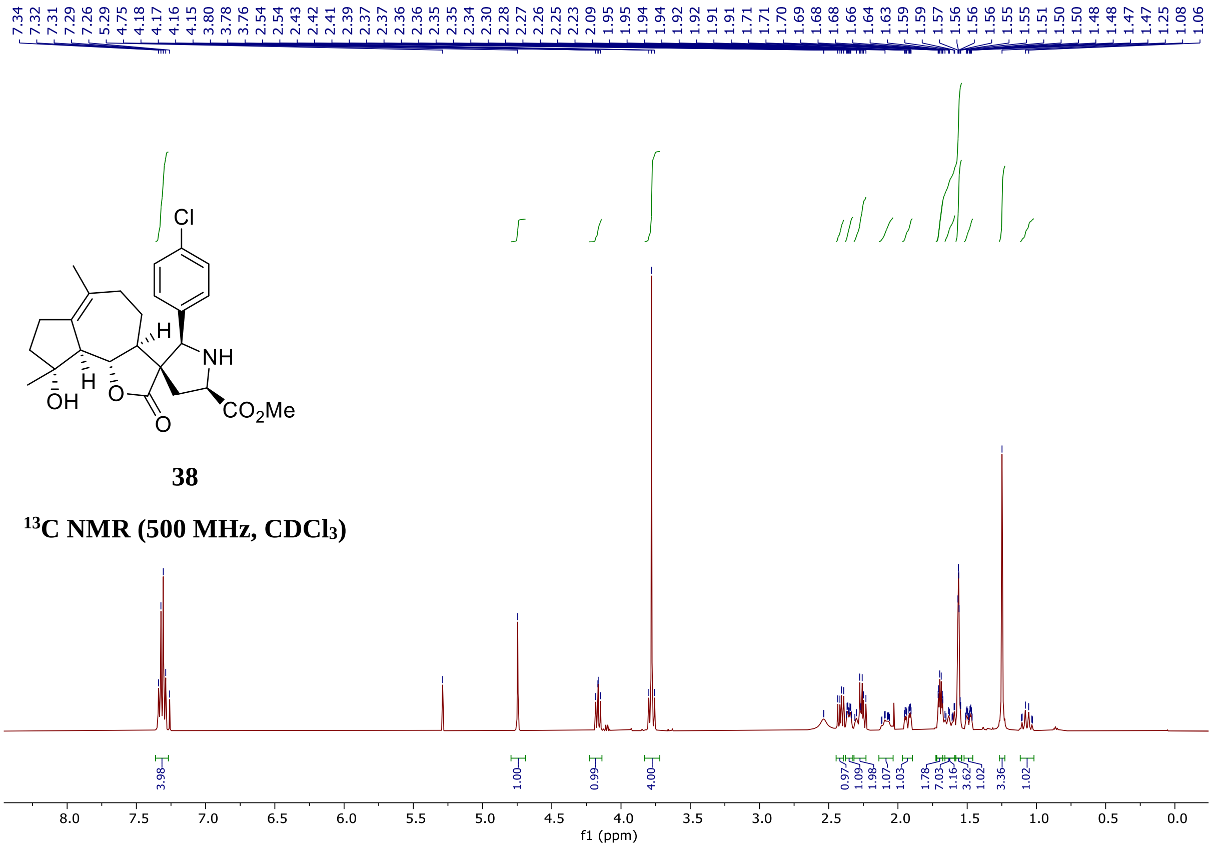

38

^{13}C NMR (500 MHz, CDCl$_3$)

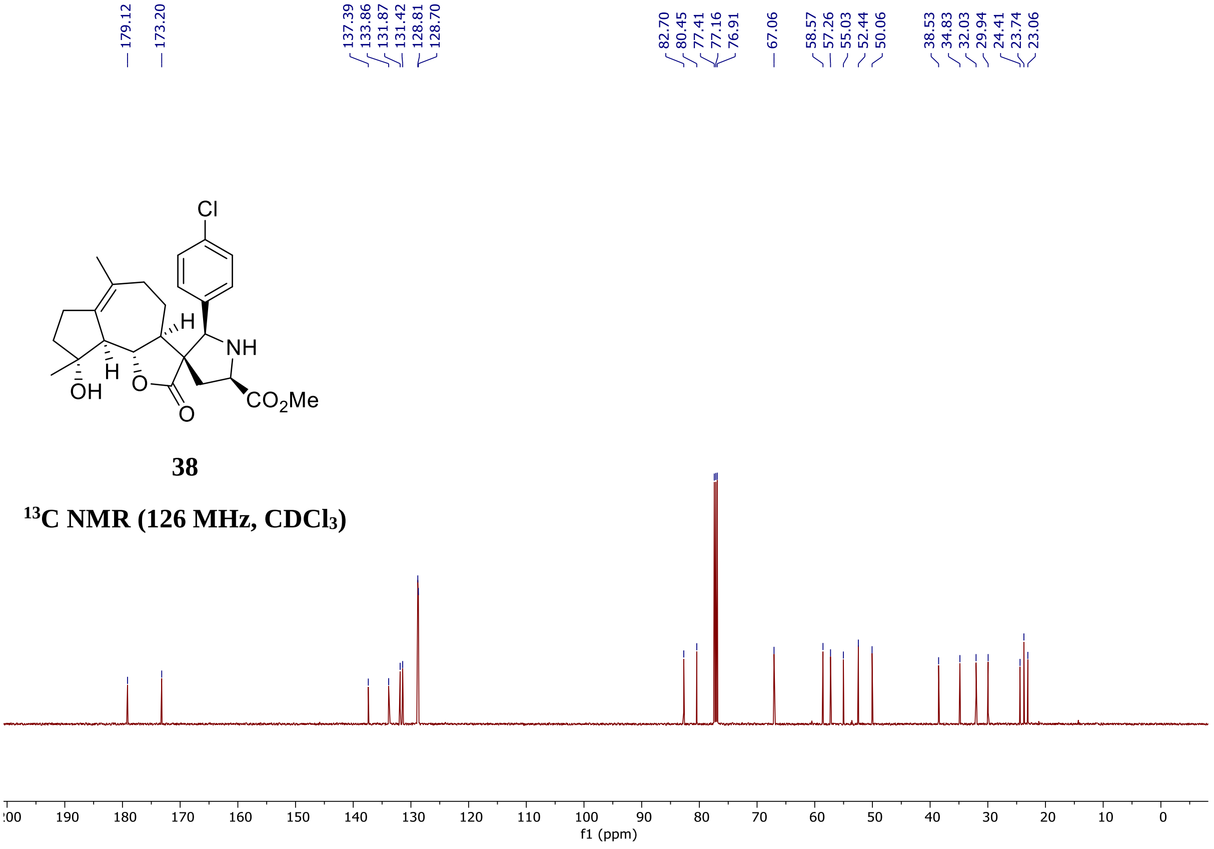

38

^{13}C NMR (126 MHz, CDCl$_3$)

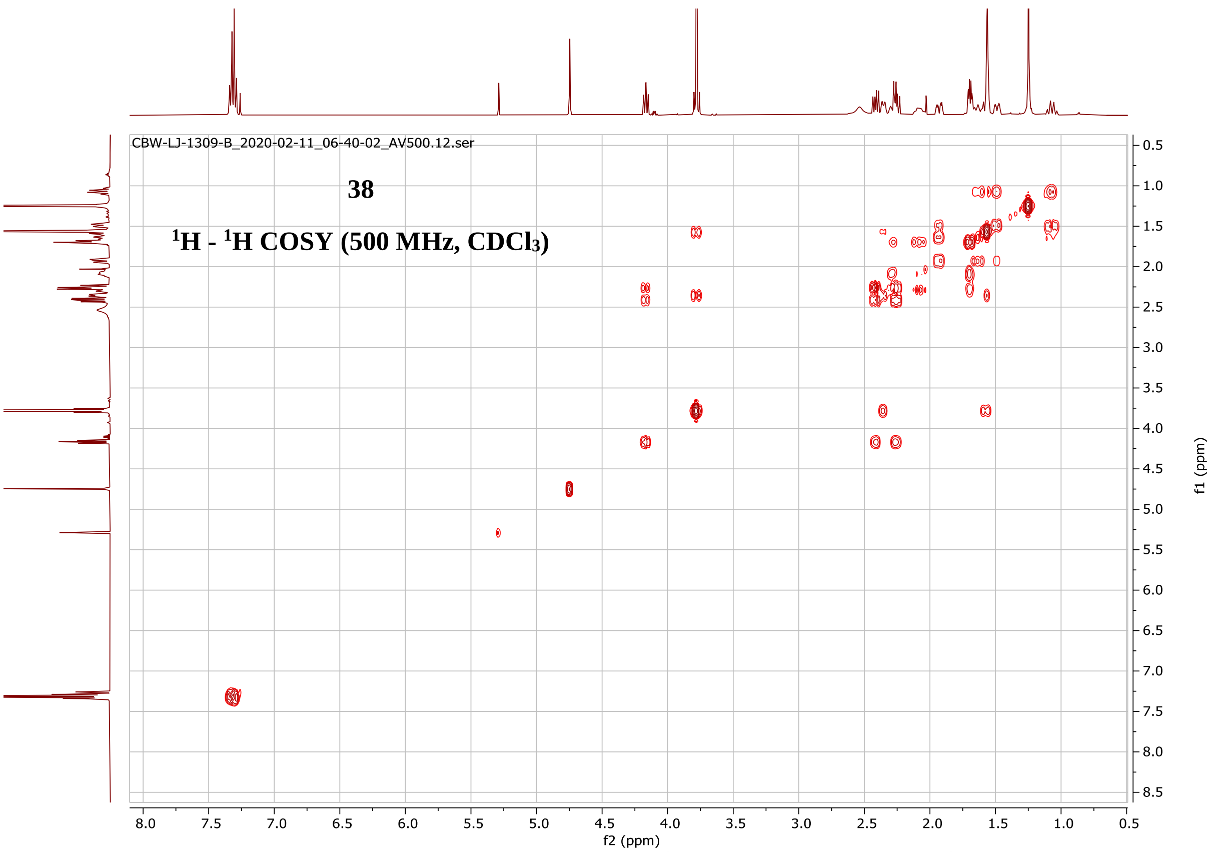
CBW-LJ-1309-B_2020-02-11_06-40-02_AV500.12.ser
38
¹H - ¹H COSY (500 MHz, CDCl₃)
f2 (ppm)
f1 (ppm)

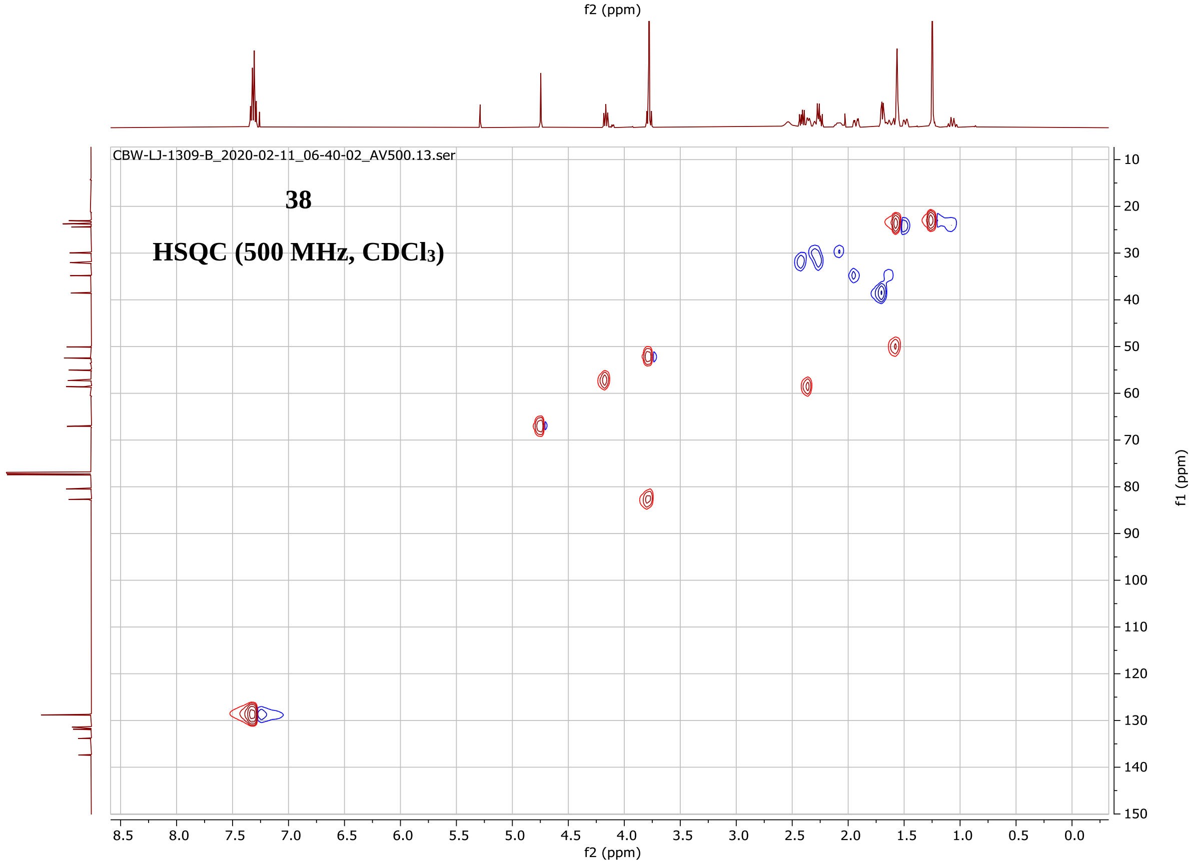
CBW-LJ-1309-B_2020-02-11_06-40-02_AV500.13.ser
38
HSQC (500 MHz, CDCl₃)
f2 (ppm)
f1 (ppm)

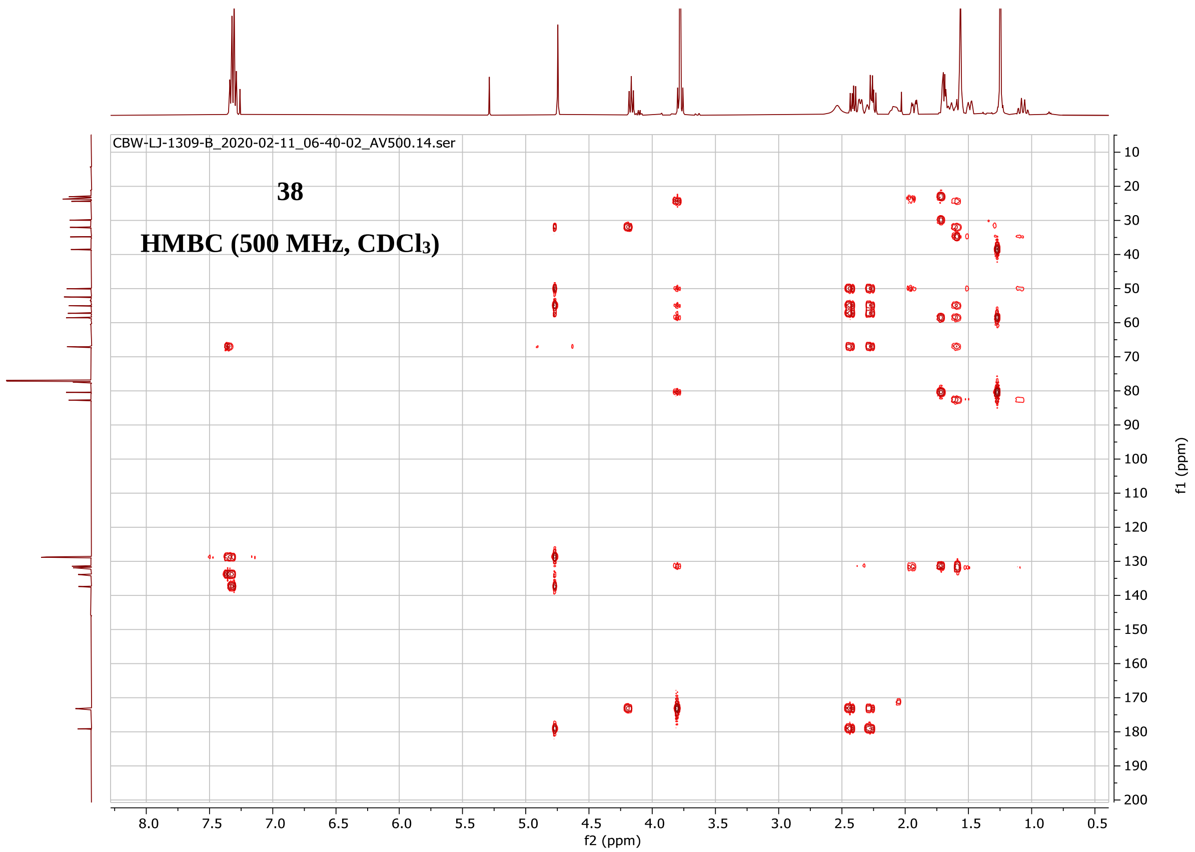

CBW-LJ-1309-B_2020-02-11_06-40-02_AV500.14.ser
38
HMBC (500 MHz, CDCl3)
f2 (ppm)
f1 (ppm)

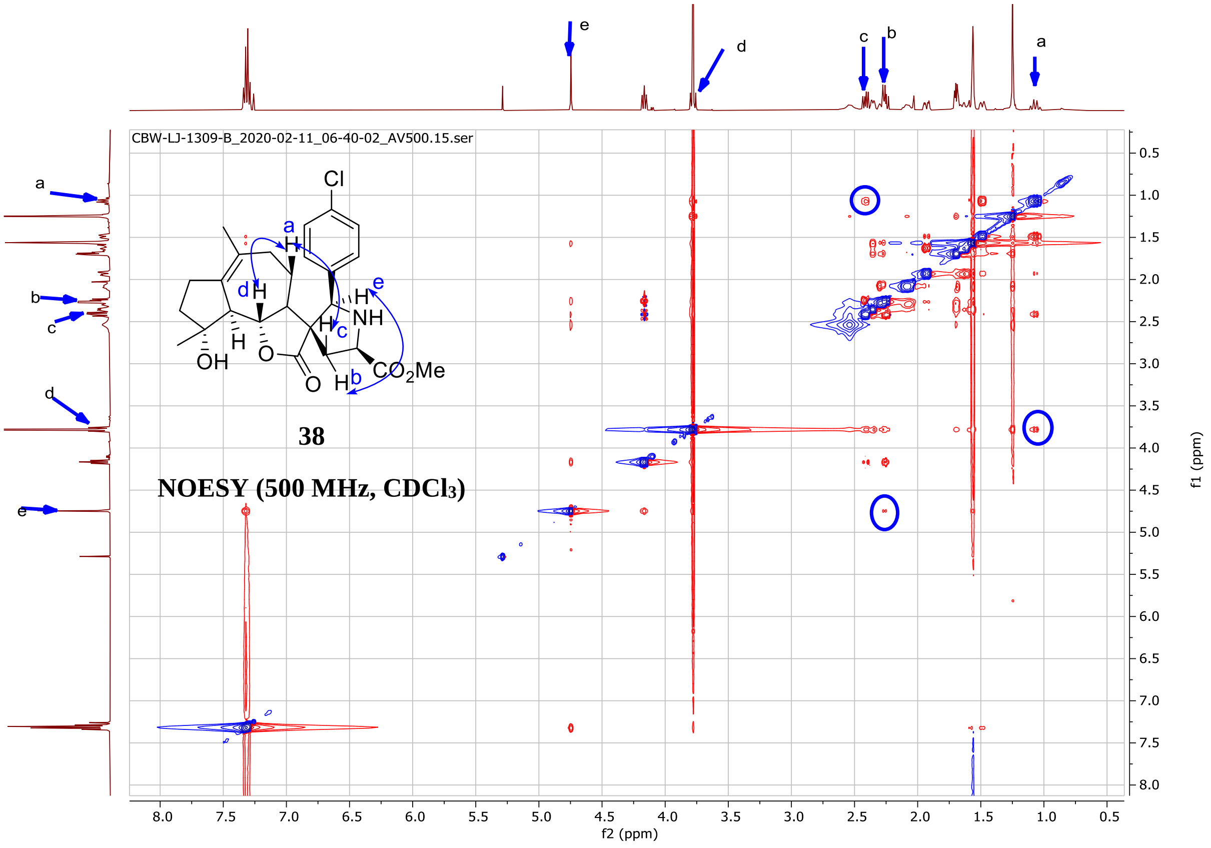

CBW-LJ-1309-B_2020-02-11_06-40-02_AV500.15.ser
a
b
c
d
e
Cl
OH
NH
CO_2Me
38
NOESY (500 MHz, CDCl3)
f2 (ppm)
f1 (ppm)

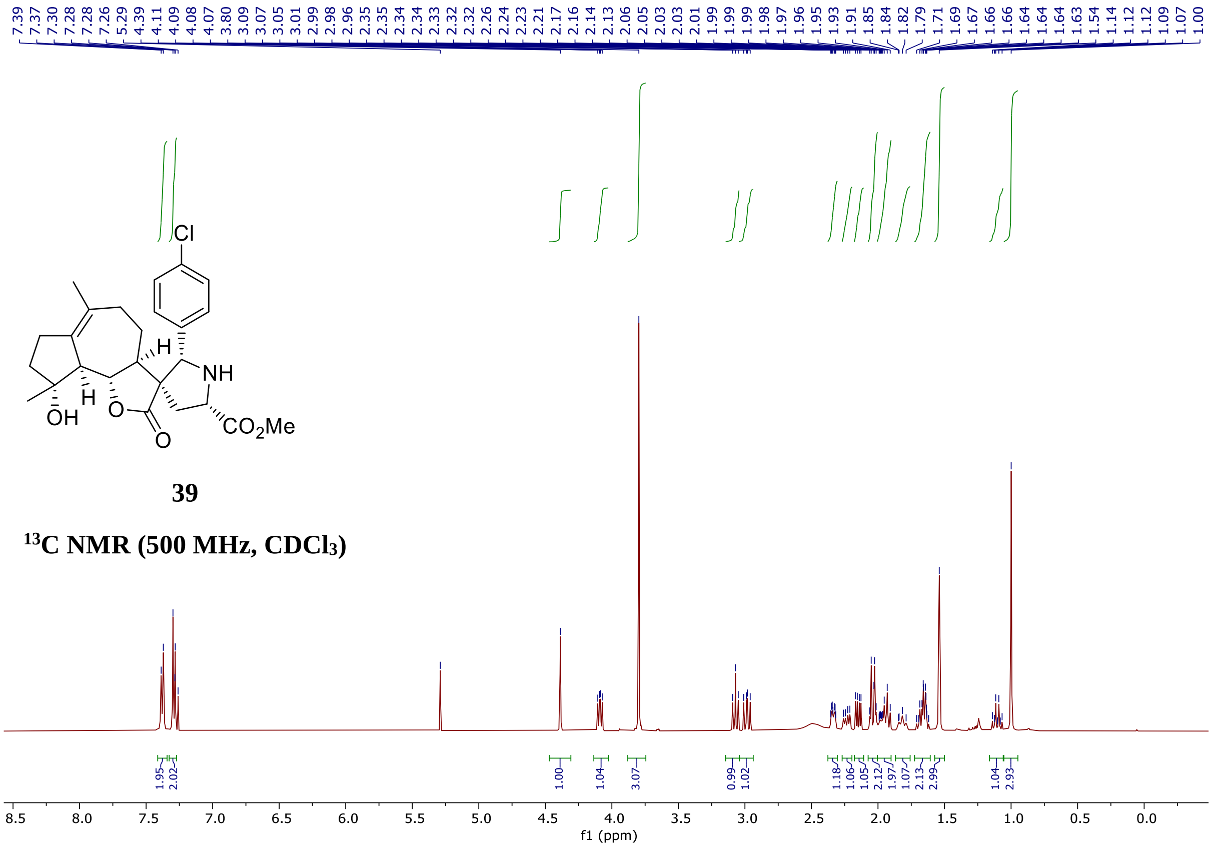

39

^{13}C NMR (500 MHz, CDCl$_3$)

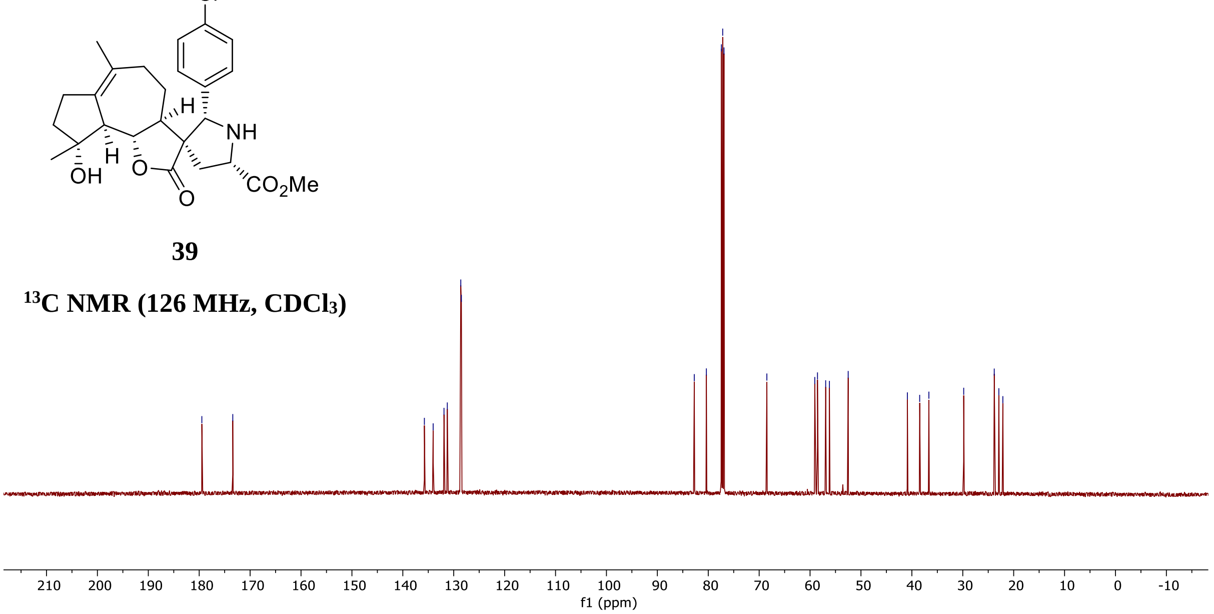

39

^{13}C NMR (126 MHz, CDCl$_3$)

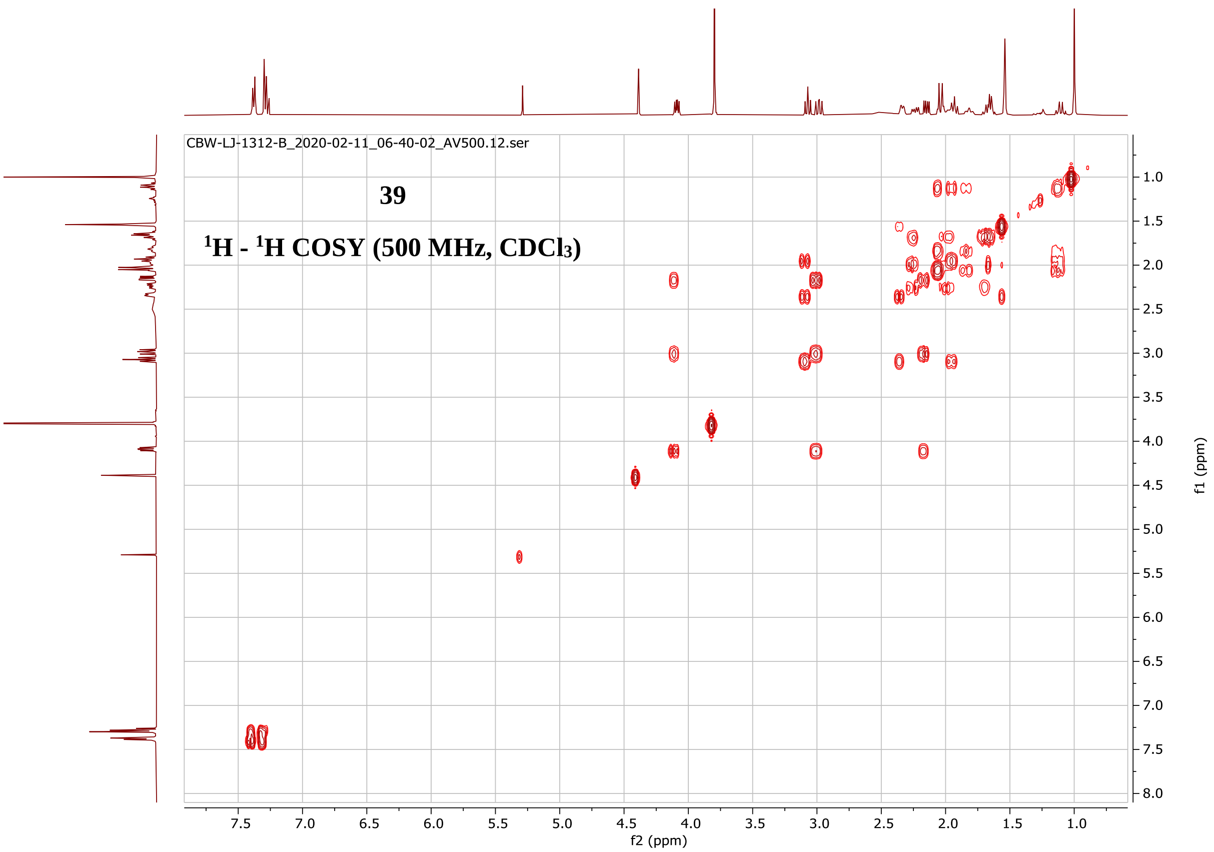
CBW-LJ-1312-B_2020-02-11_06-40-02_AV500.12.ser
39
¹H - ¹H COSY (500 MHz, CDCl3)
f2 (ppm)
f1 (ppm)

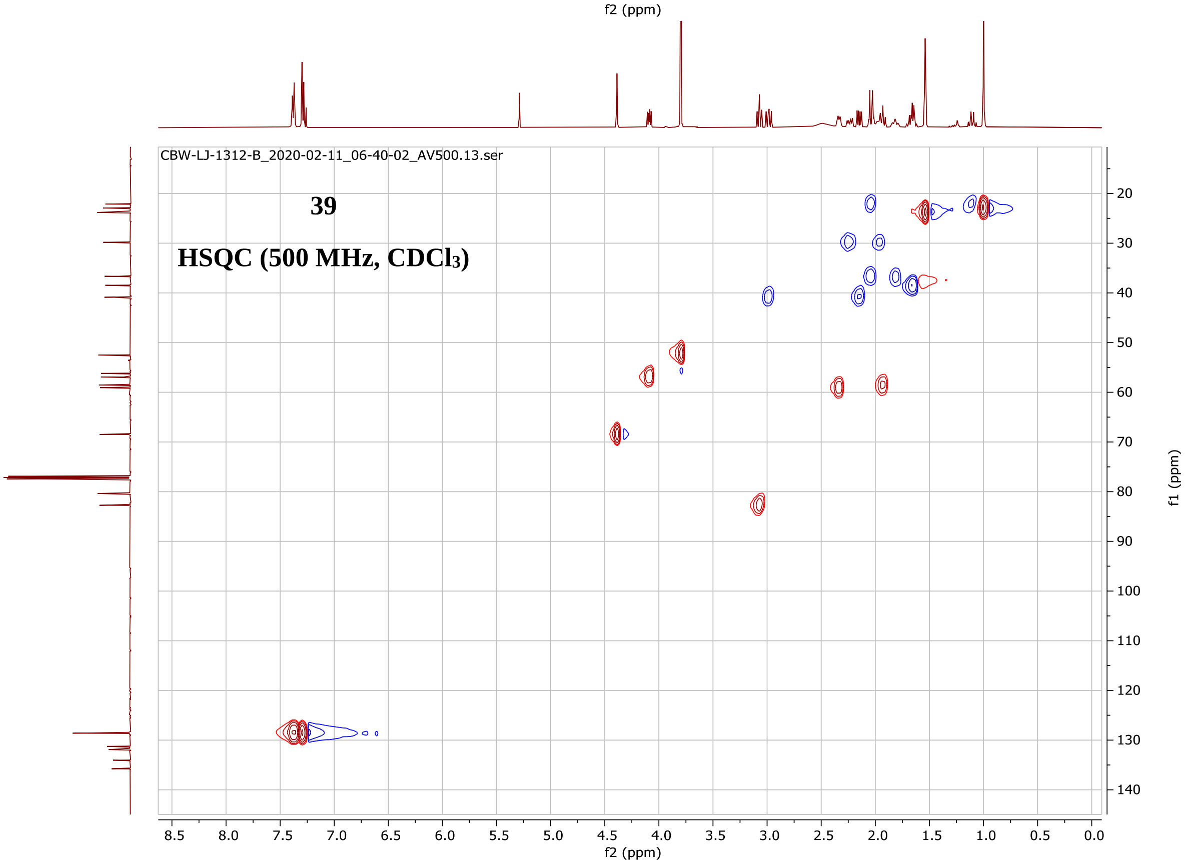
CBW-LJ-1312-B_2020-02-11_06-40-02_AV500.13.ser
39
HSQC (500 MHz, CDCl3)
f2 (ppm)
f1 (ppm)

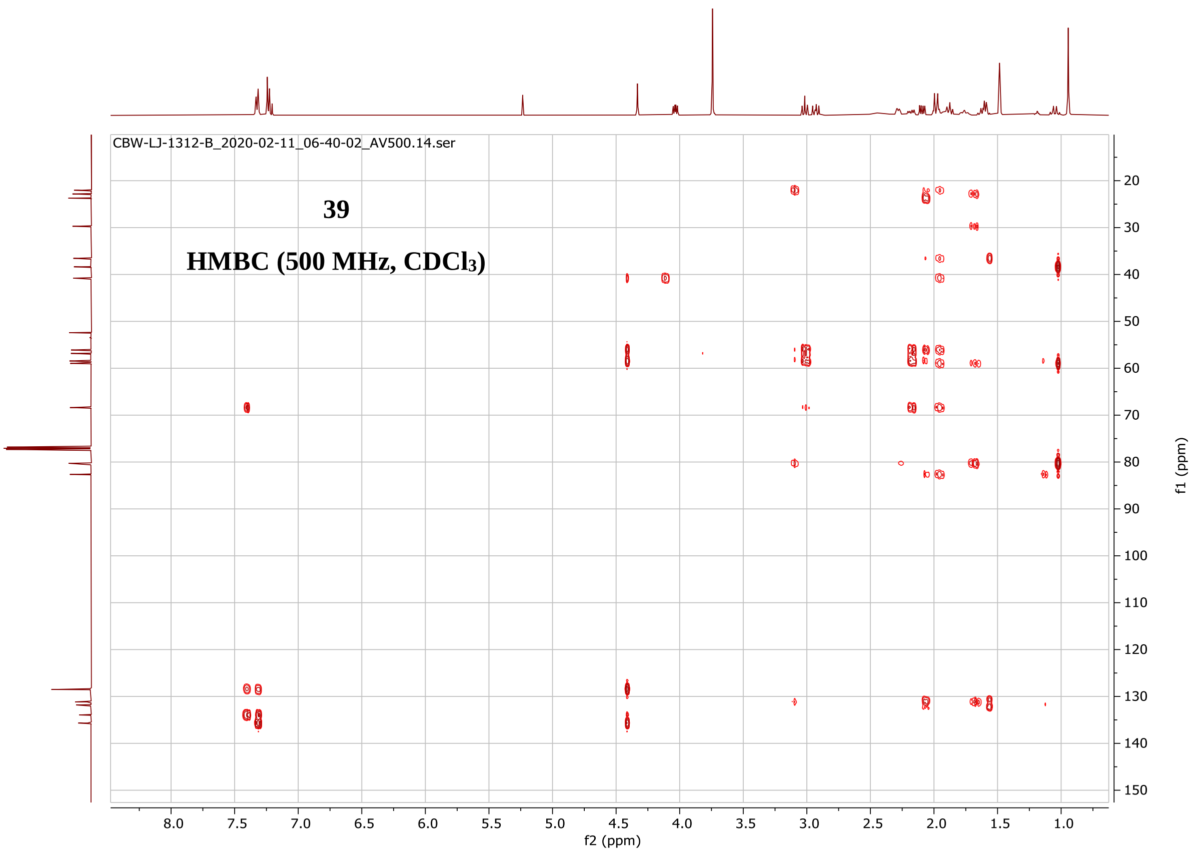
CBW-LJ-1312-B_2020-02-11_06-40-02_AV500.14.ser
39
HMBC (500 MHz, CDCl3)
f1 (ppm)
f2 (ppm)

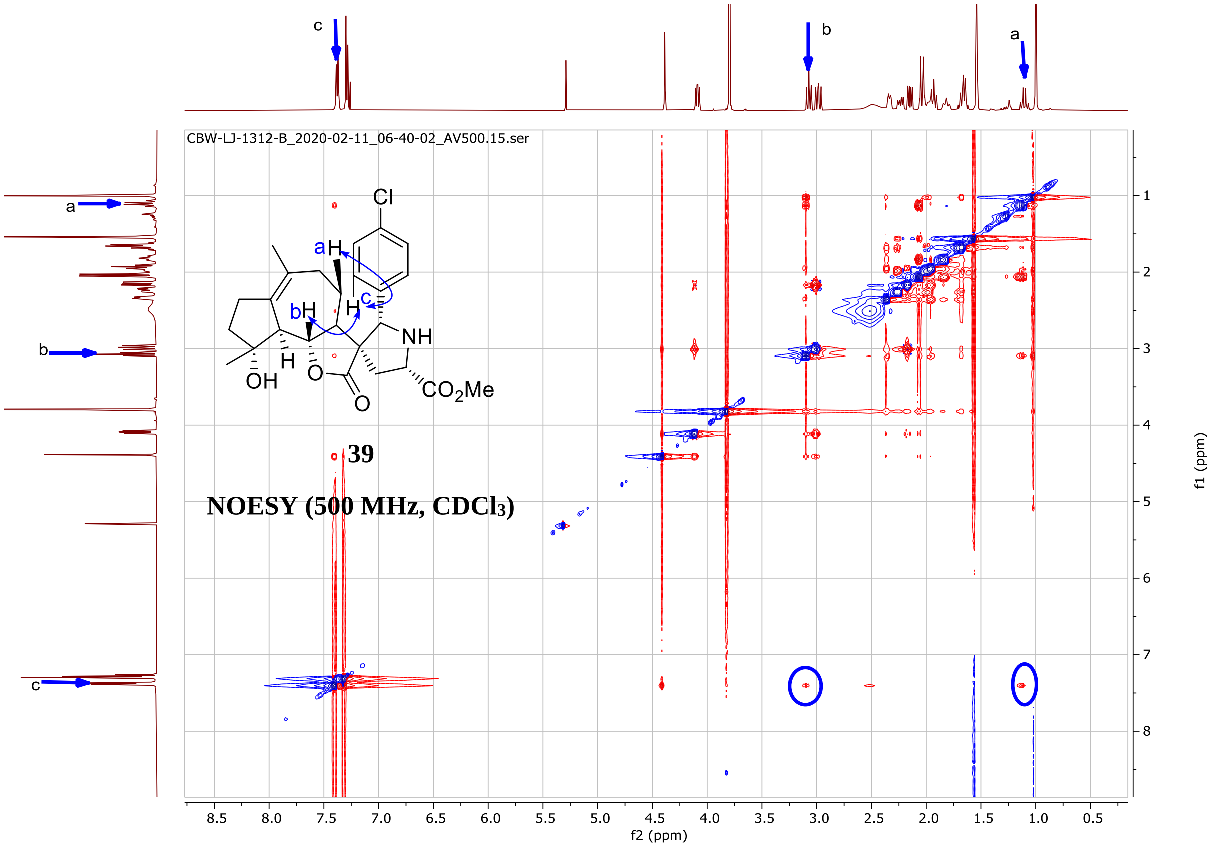
c
b
a
CBW-LJ-1312-B_2020-02-11_06-40-02_AV500.15.ser
Cl
NH
OH
CO2Me
39
NOESY (500 MHz, CDCl3)
f1 (ppm)
f2 (ppm)

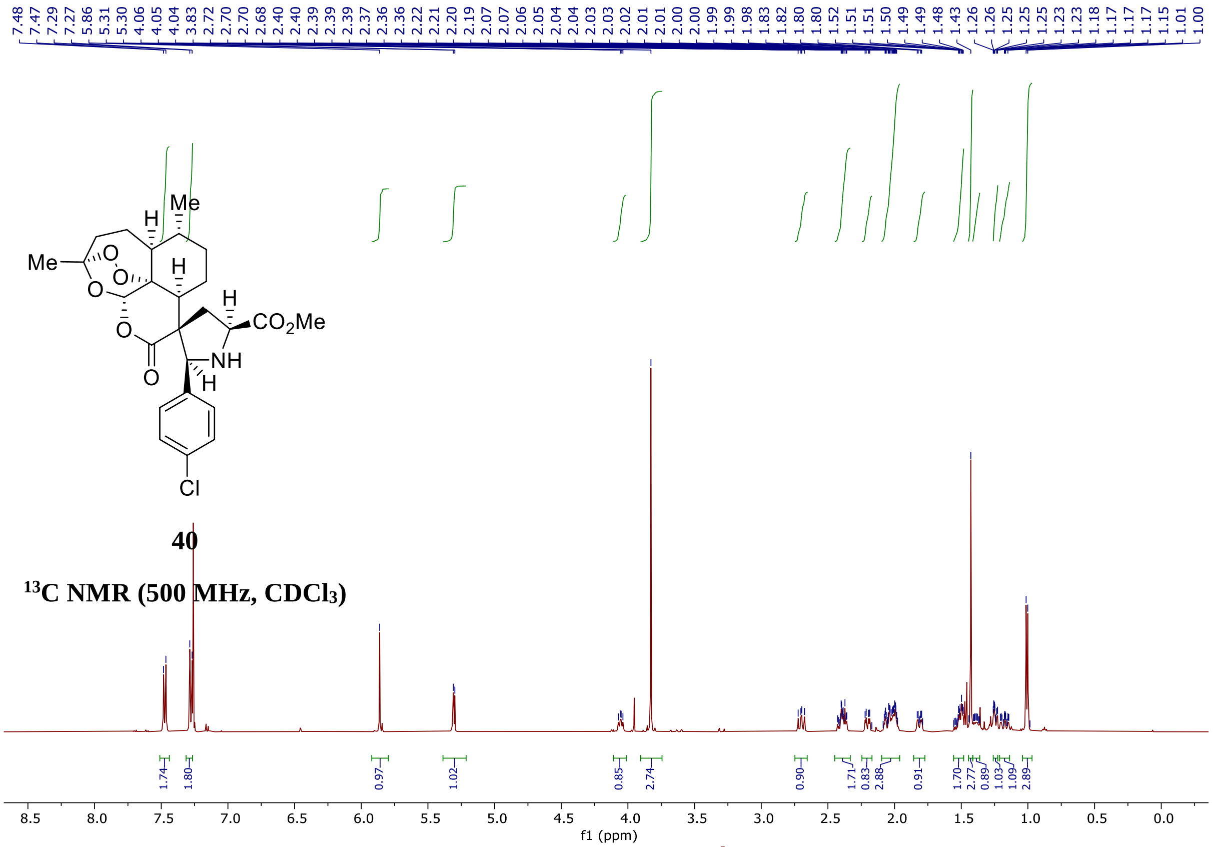

40

^{13}C NMR (500 MHz, CDCl$_3$)

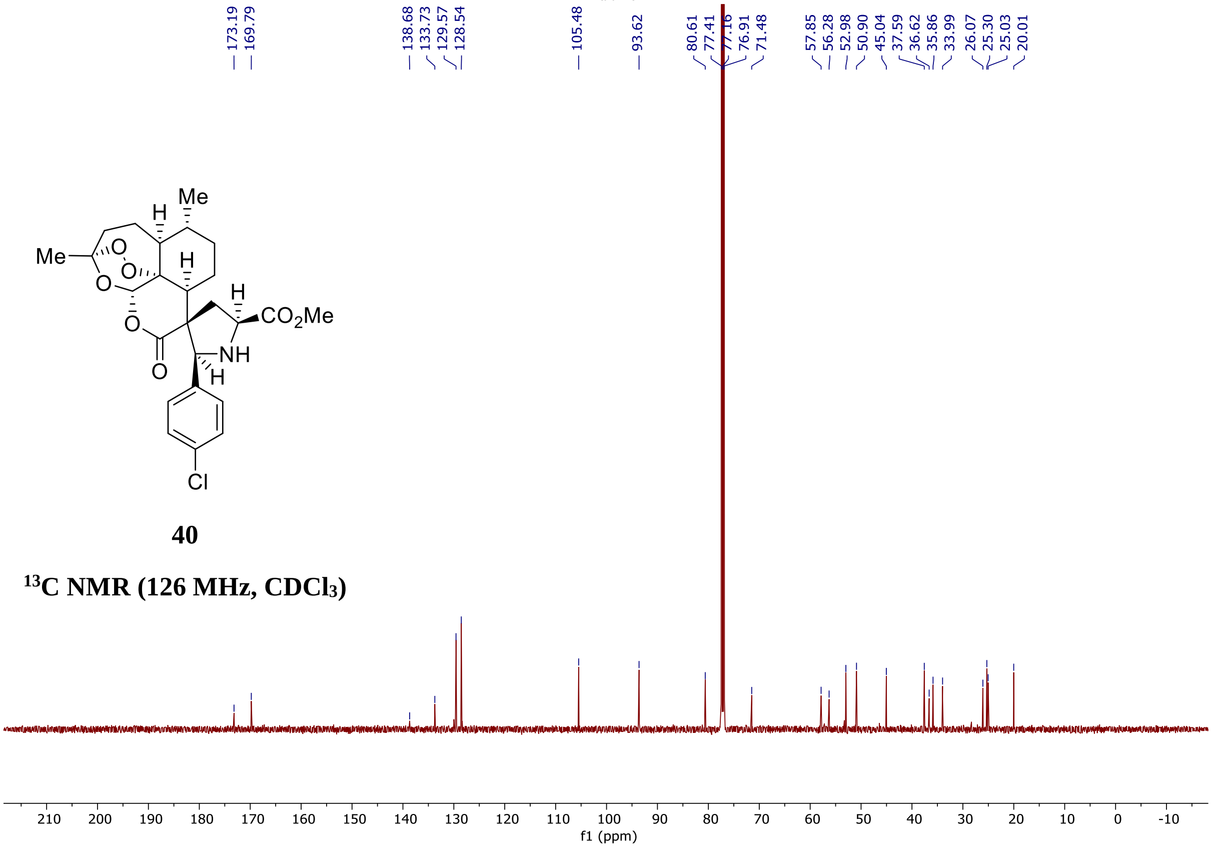

40

^{13}C NMR (126 MHz, CDCl$_3$)

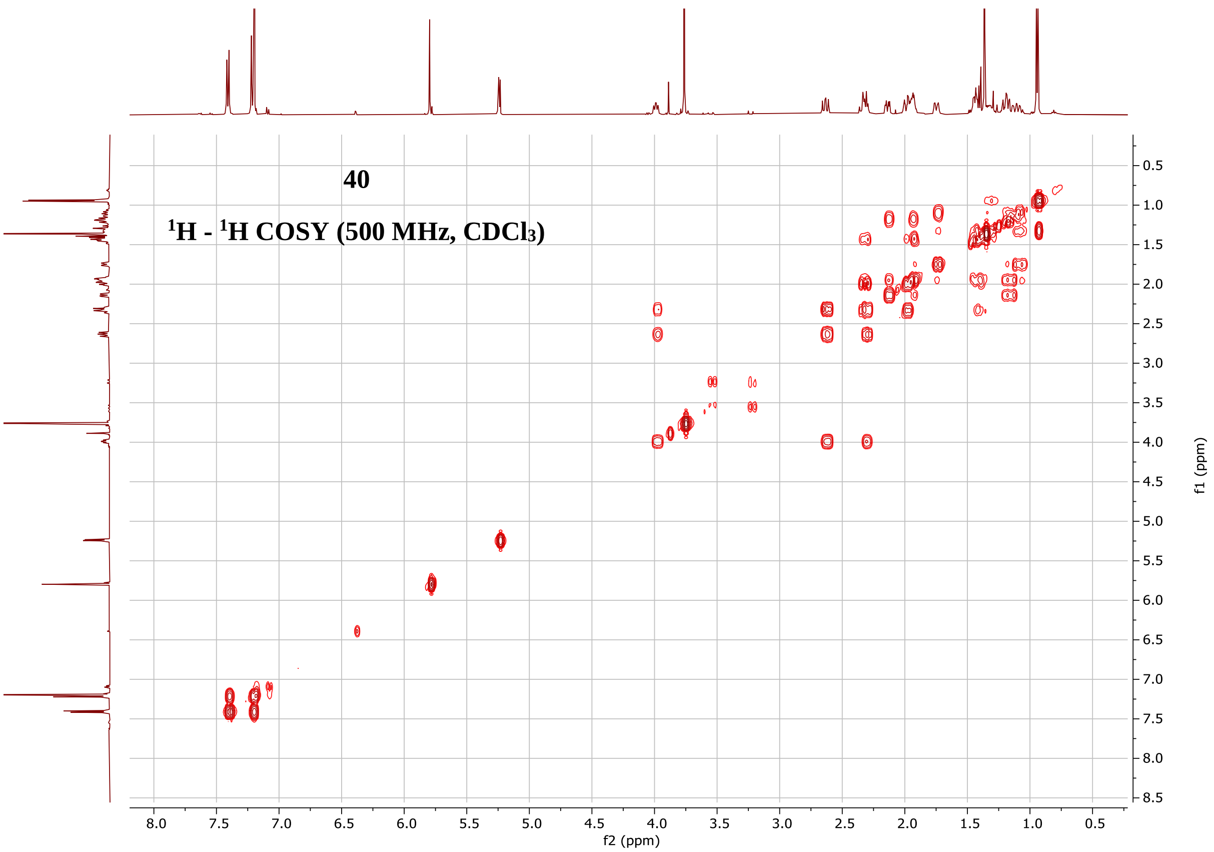
40
^1H - ^1H COSY (500 MHz, $CDCl_3$)
f2 (ppm)
f1 (ppm)

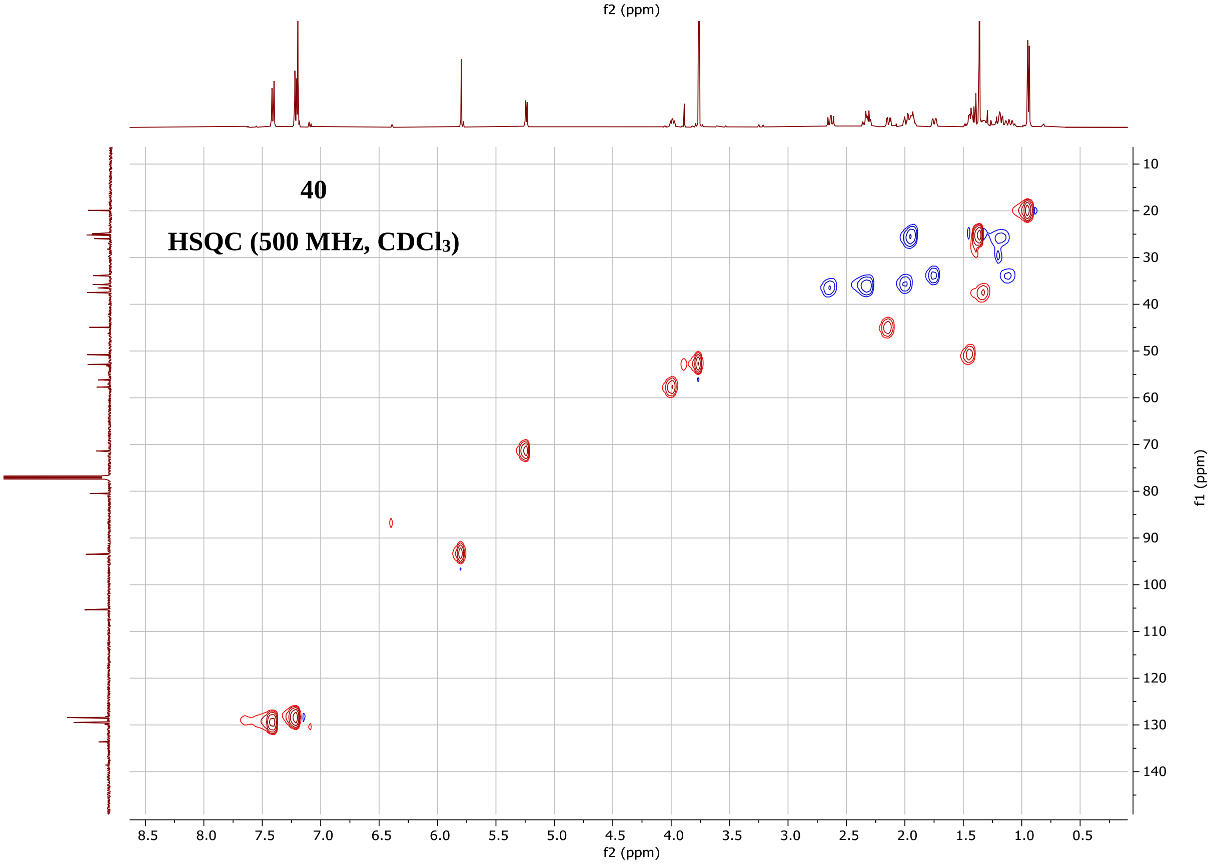
40
HSQC (500 MHz, $CDCl_3$)
f2 (ppm)
f1 (ppm)

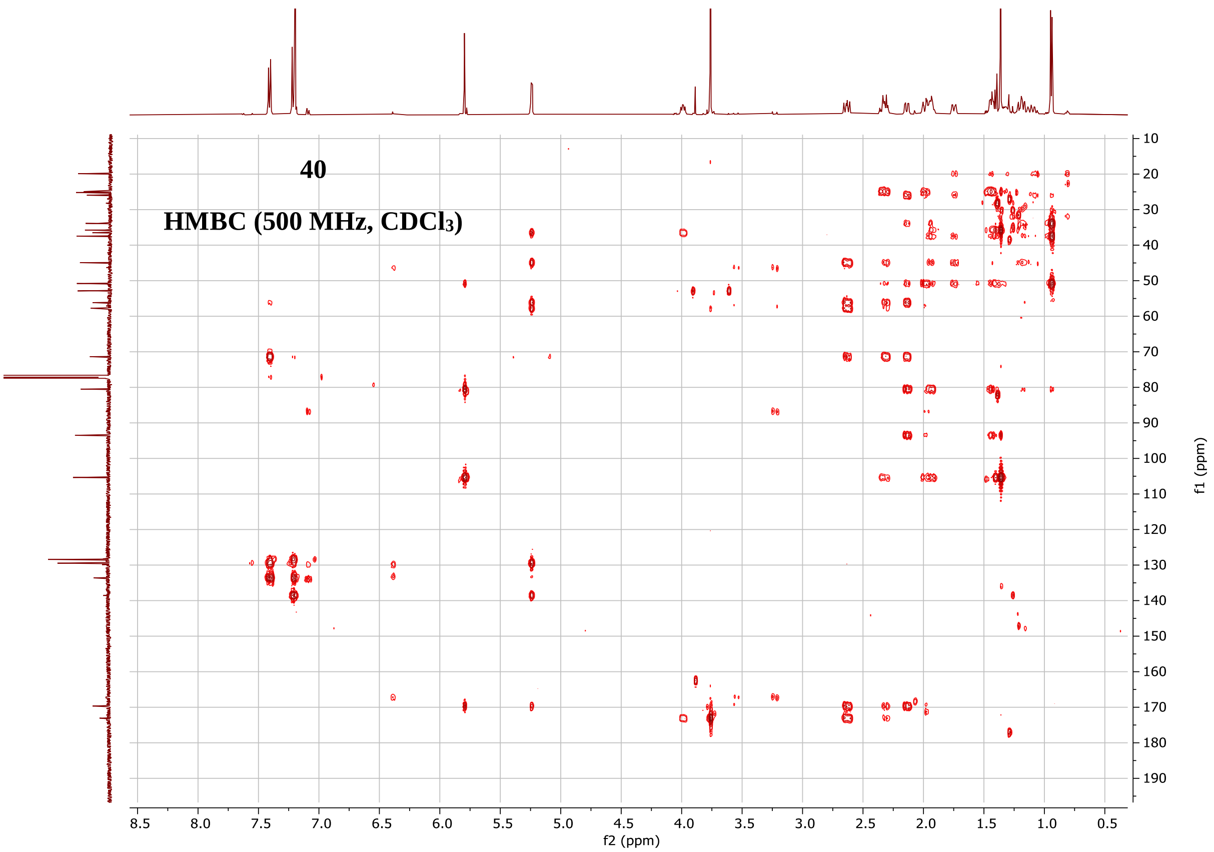
40
HMBC (500 MHz, CDCl3)
f2 (ppm)
f1 (ppm)

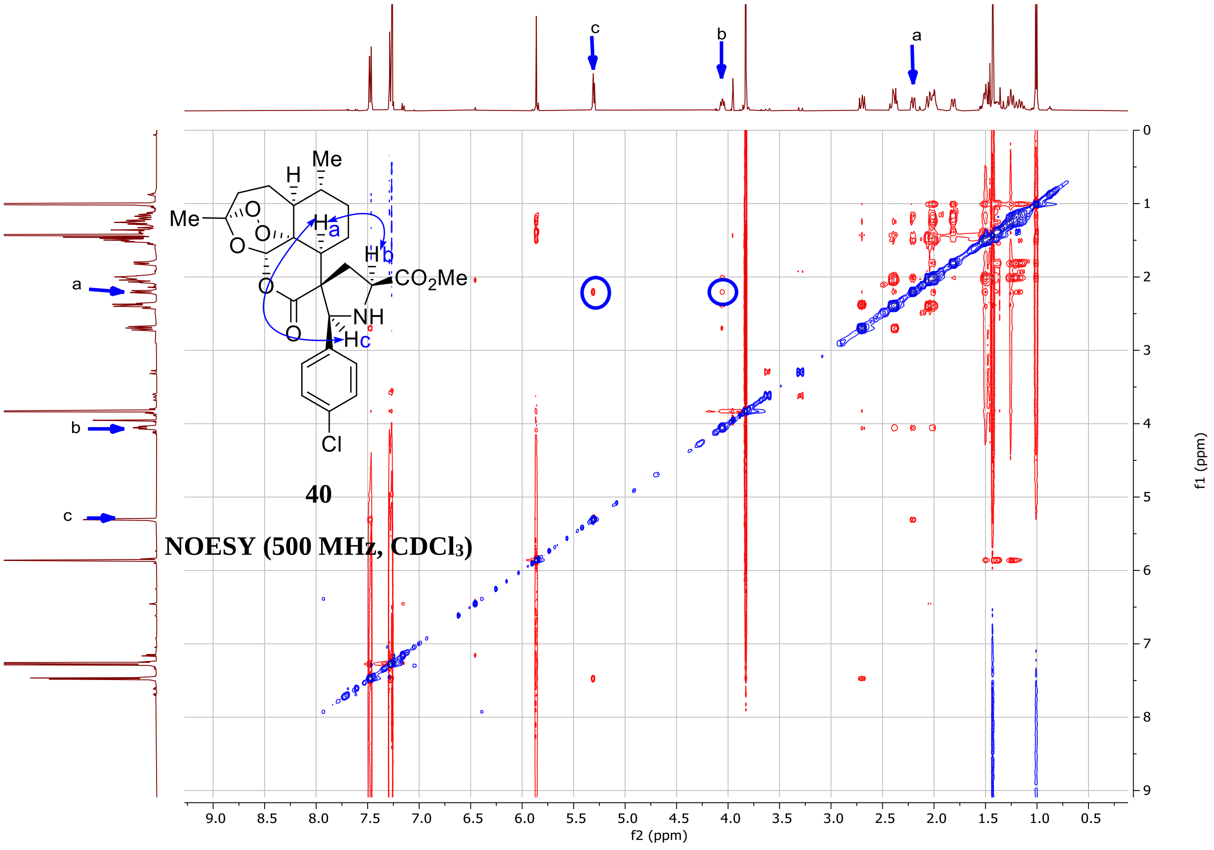
c
b
a
Me
H
Me
O
O
O
O
Ha
Hb
CO2Me
NH
O
Hc
Cl
40
NOESY (500 MHz, CDCl3)
f2 (ppm)
f1 (ppm)

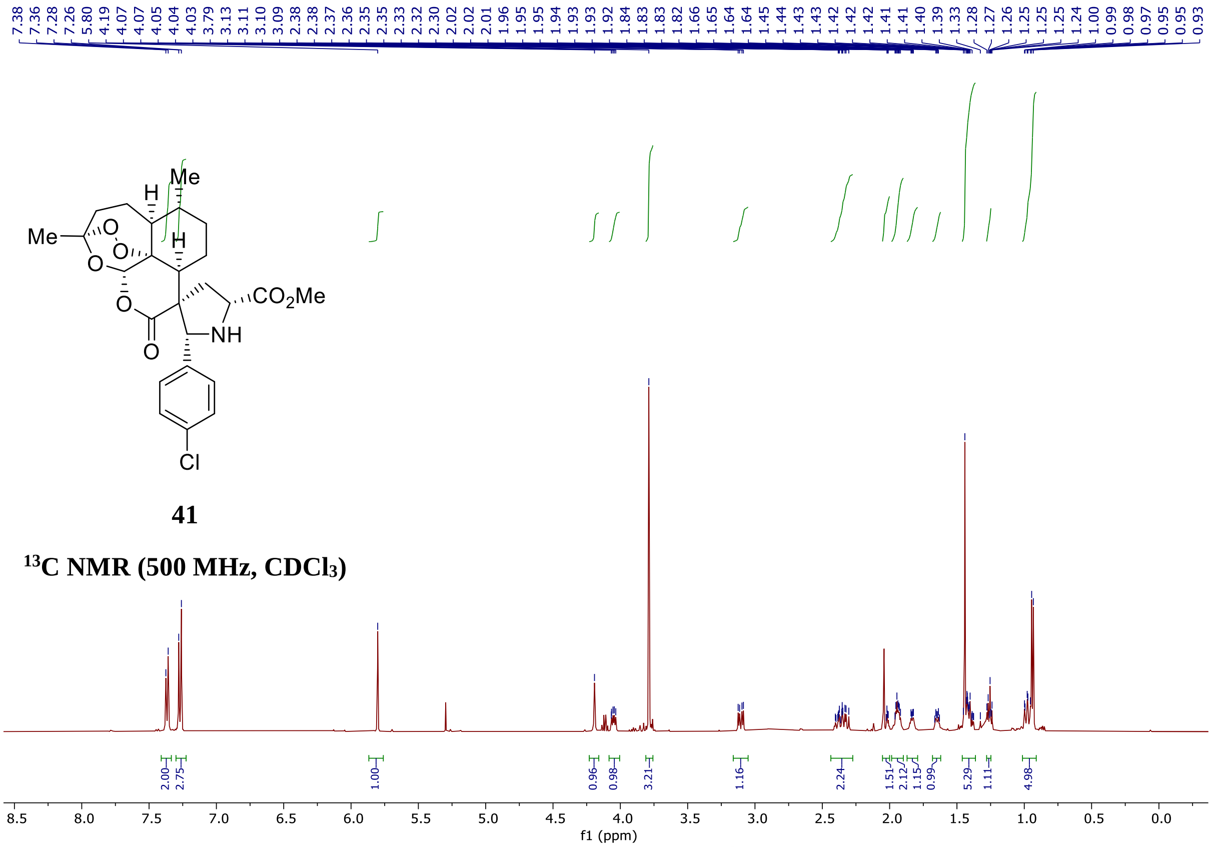

41

^{13}C NMR (500 MHz, $CDCl_3$)

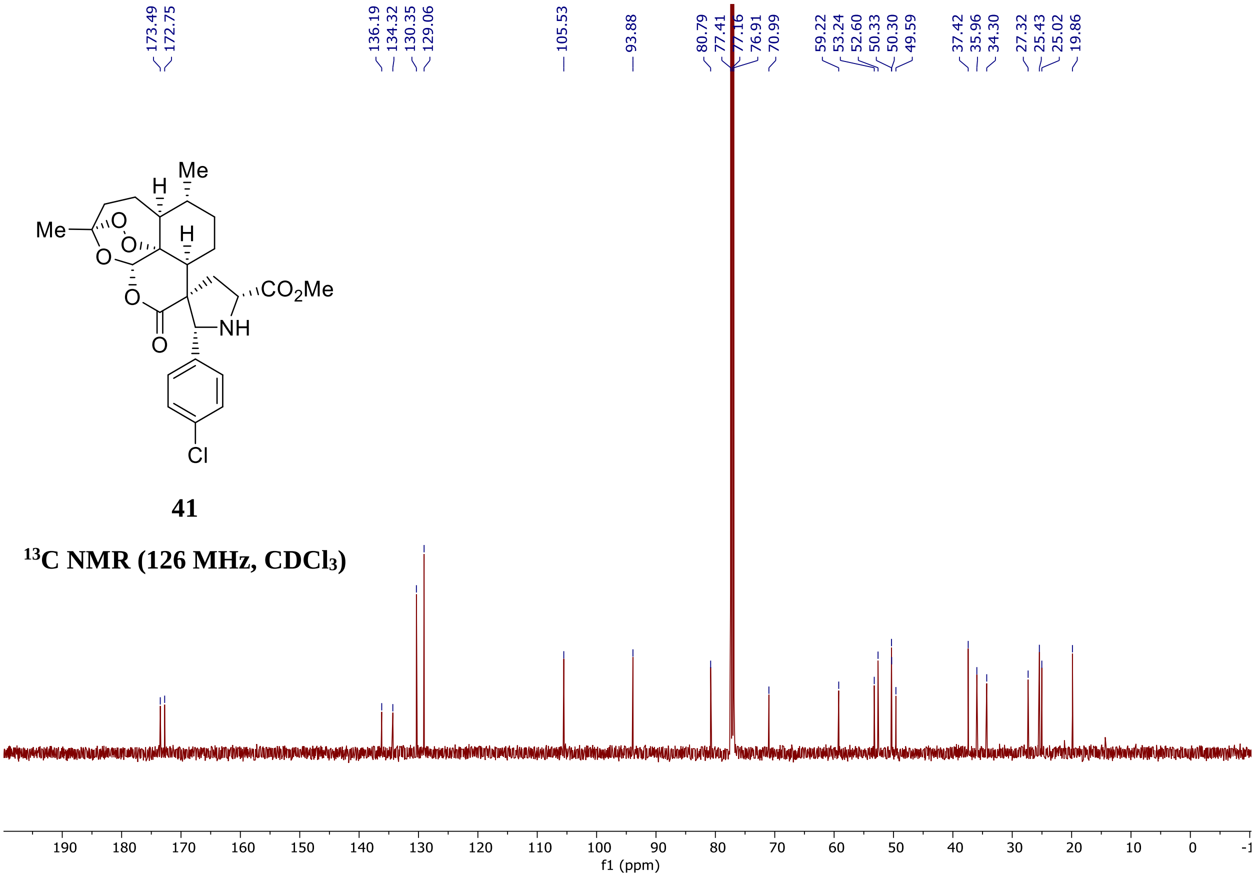

41

^{13}C NMR (126 MHz, $CDCl_3$)

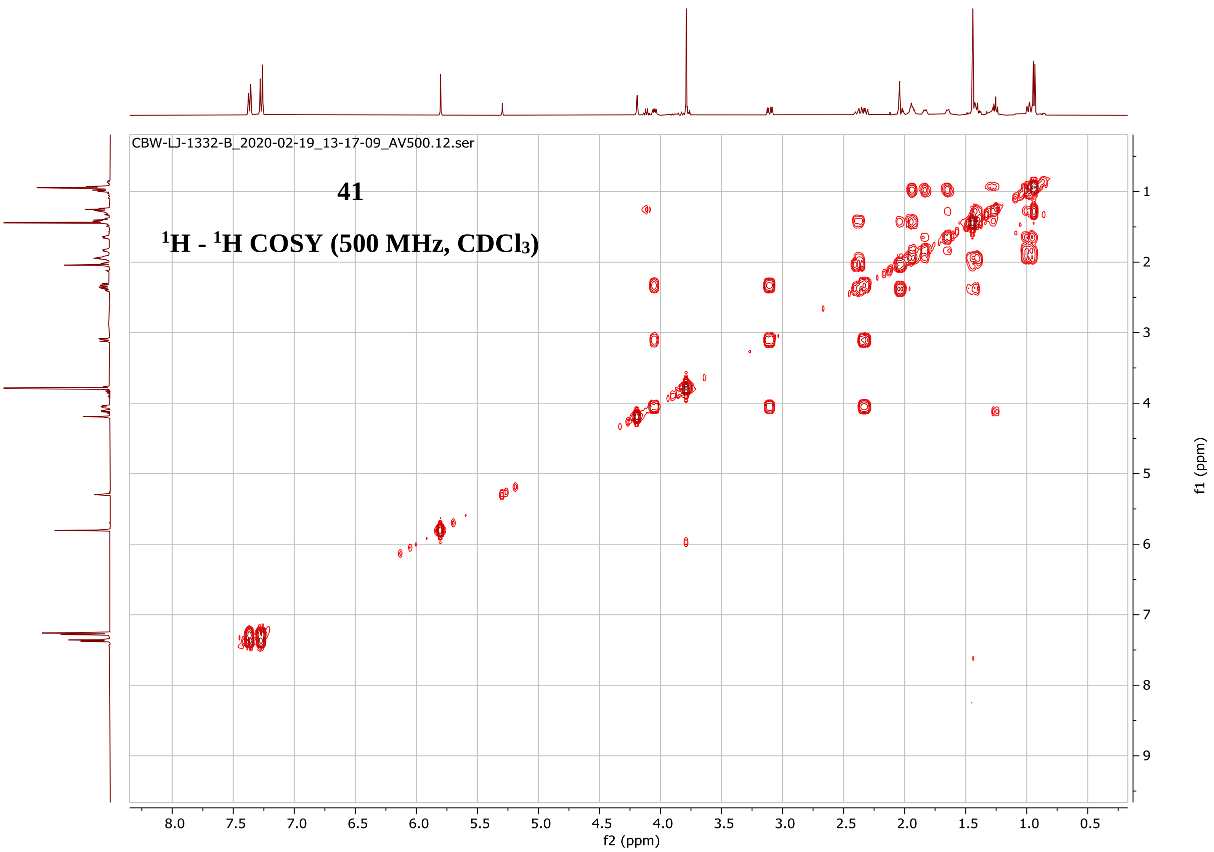
CBW-LJ-1332-B_2020-02-19_13-17-09_AV500.12.ser
41
^{1}H - ^{1}H COSY (500 MHz, CDCl$_3$)
f2 (ppm)
f1 (ppm)

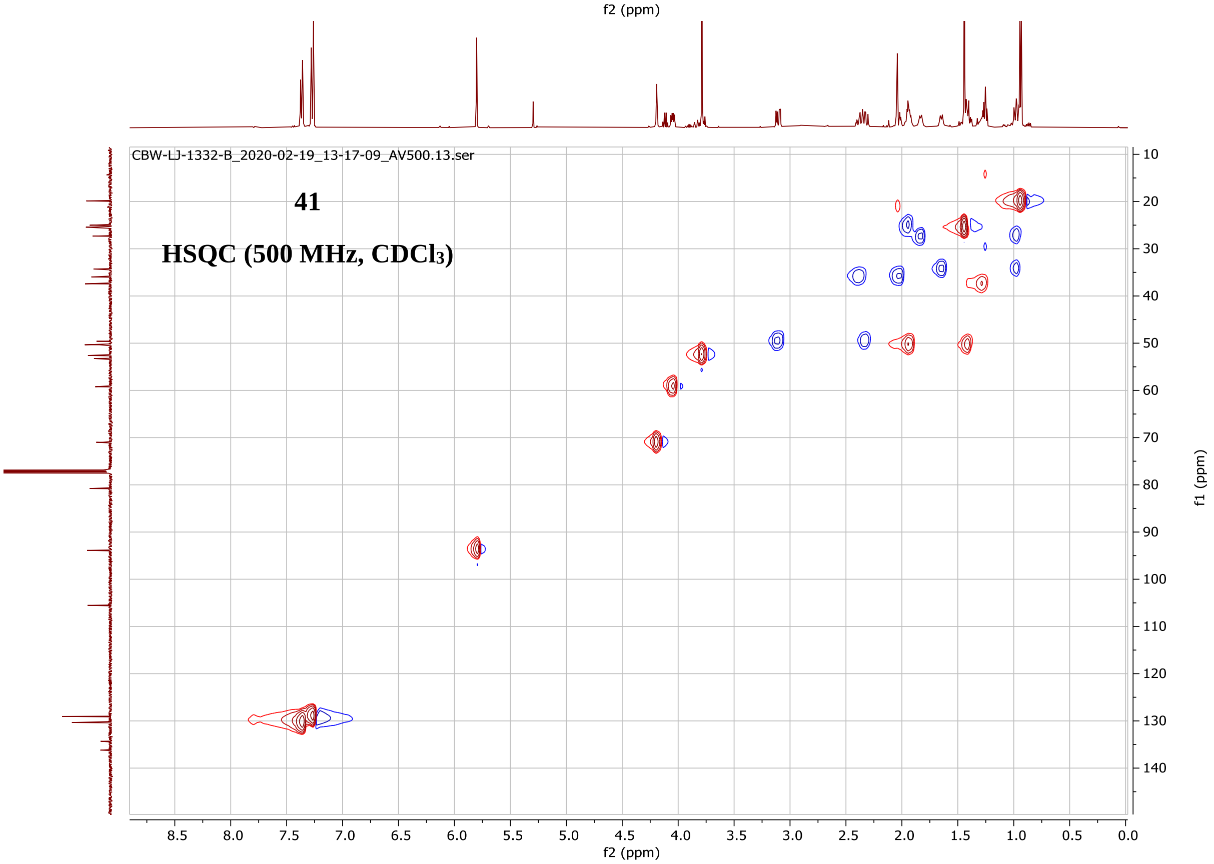
CBW-LJ-1332-B_2020-02-19_13-17-09_AV500.13.ser
41
HSQC (500 MHz, CDCl$_3$)
f2 (ppm)
f1 (ppm)

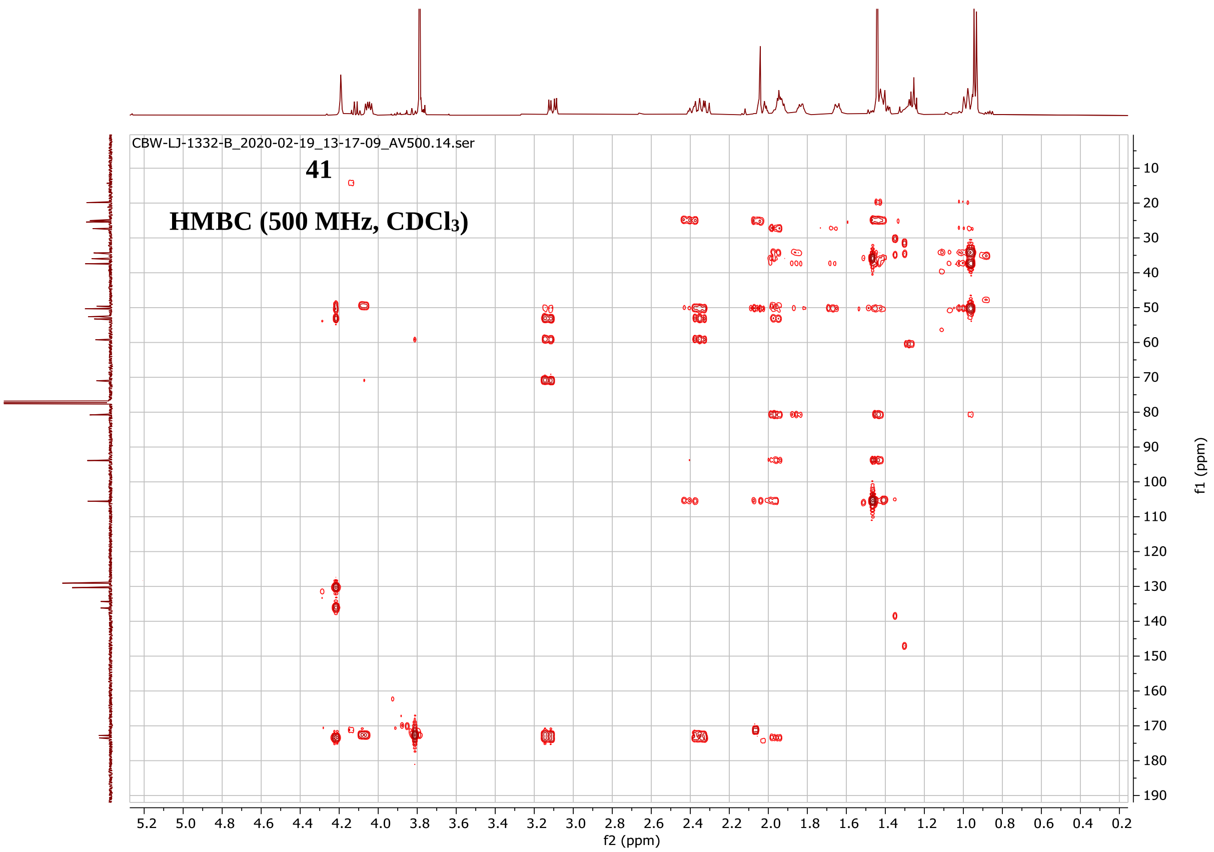

CBW-LJ-1332-B_2020-02-19_13-17-09_AV500.14.ser
41
HMBC (500 MHz, CDCl3)
f2 (ppm)
f1 (ppm)

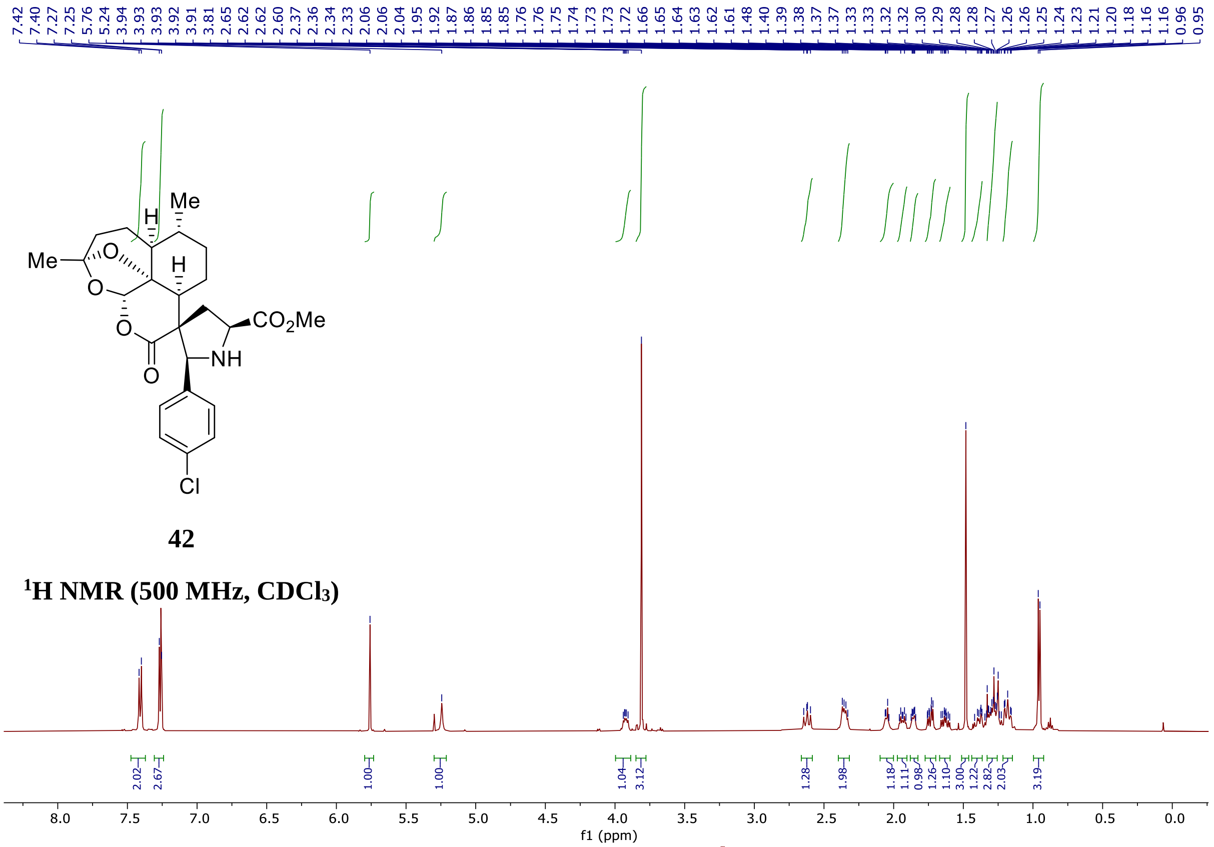

42

^{1}H NMR (500 MHz, $CDCl_3$)

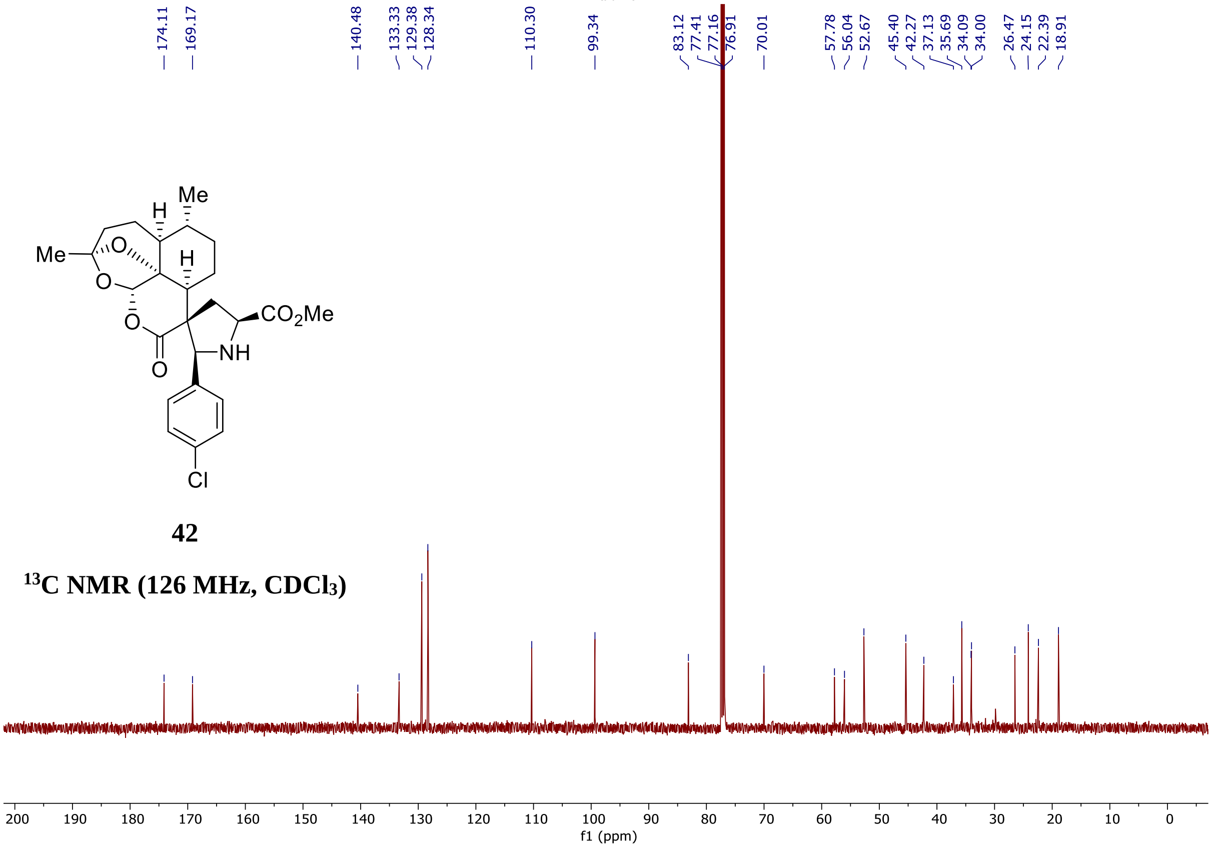

42

^{13}C NMR (126 MHz, $CDCl_3$)

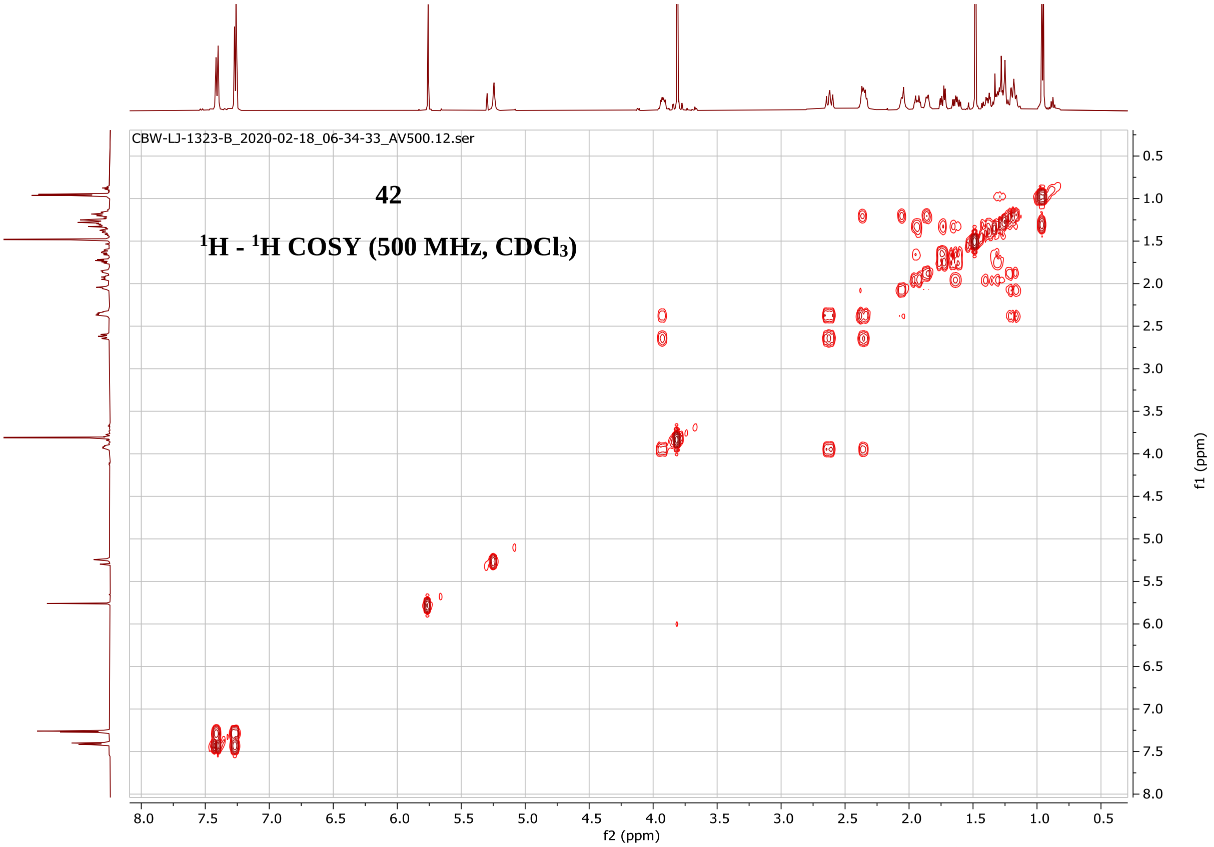
CBW-LJ-1323-B_2020-02-18_06-34-33_AV500.12.ser
42
^{1}H - ^{1}H COSY (500 MHz, CDCl$_3$)
f2 (ppm)
f1 (ppm)

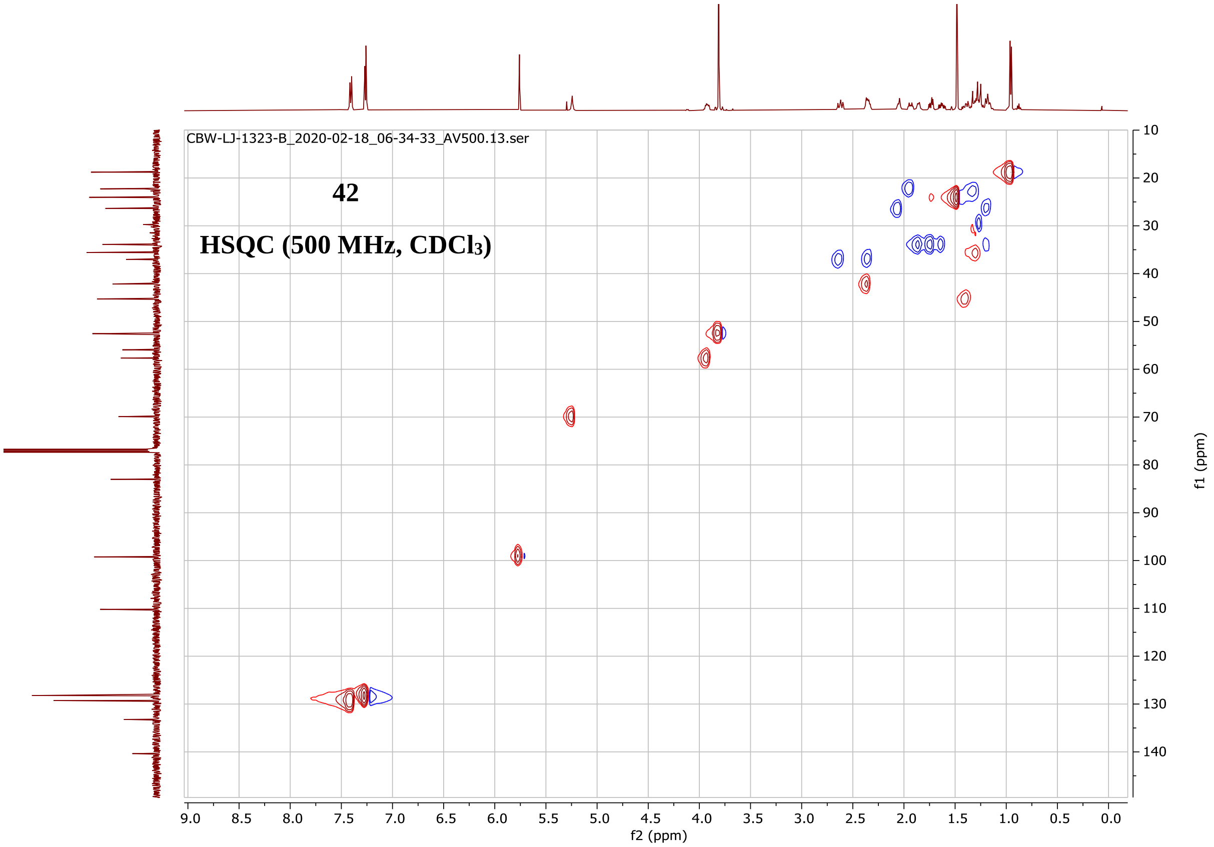
CBW-LJ-1323-B_2020-02-18_06-34-33_AV500.13.ser
42
HSQC (500 MHz, CDCl$_3$)
f2 (ppm)
f1 (ppm)

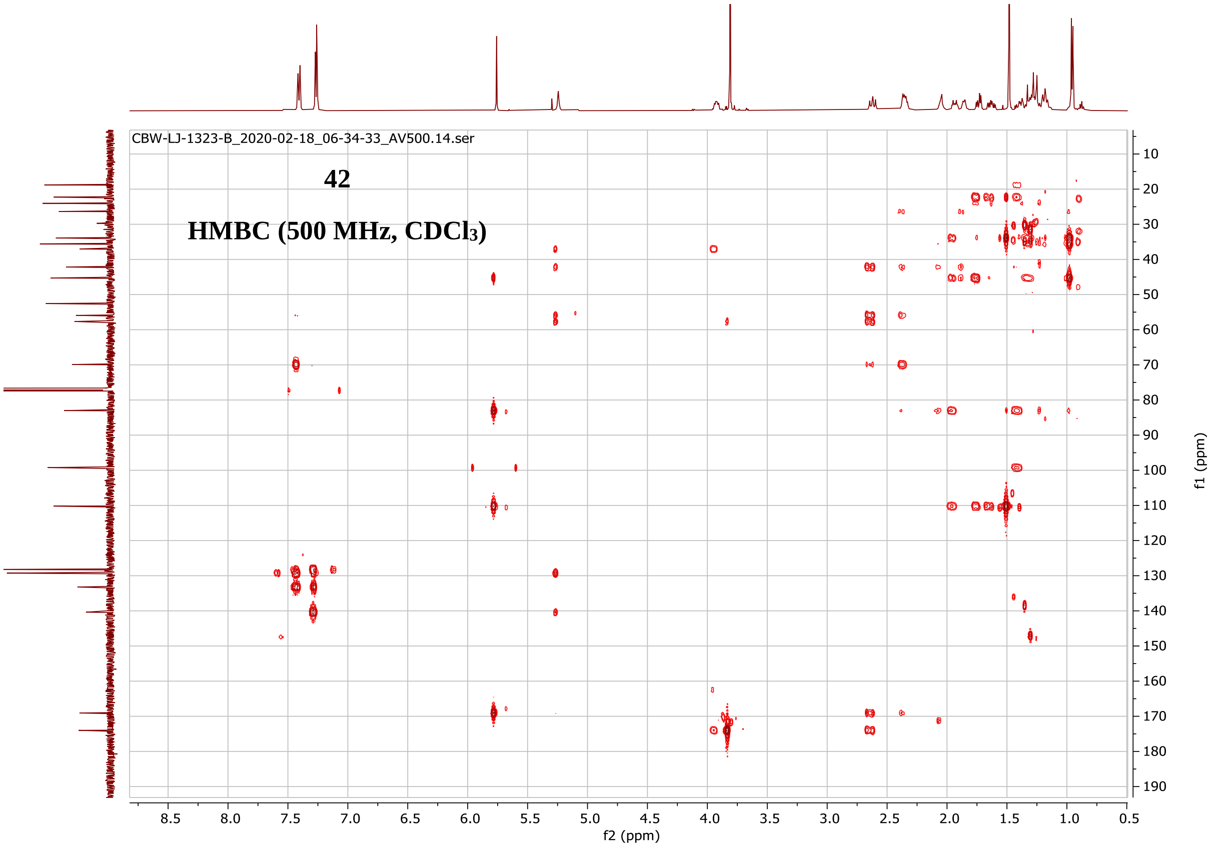
CBW-LJ-1323-B_2020-02-18_06-34-33_AV500.14.ser
42
HMBC (500 MHz, CDCl3)
f1 (ppm)
f2 (ppm)

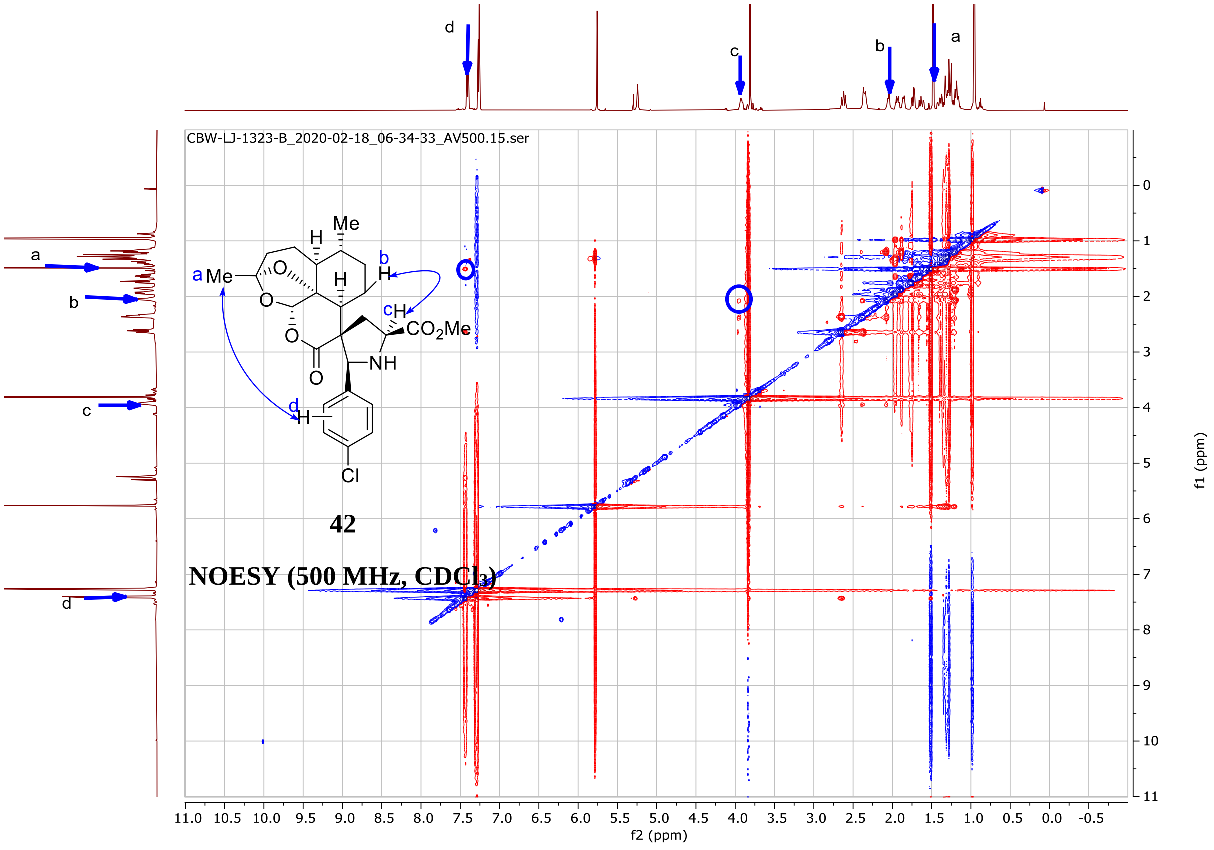
CBW-LJ-1323-B_2020-02-18_06-34-33_AV500.15.ser
a
b
c
d
Me
H
H
Cl
NH
CO2Me
42
NOESY (500 MHz, CDCl3)
f1 (ppm)
f2 (ppm)

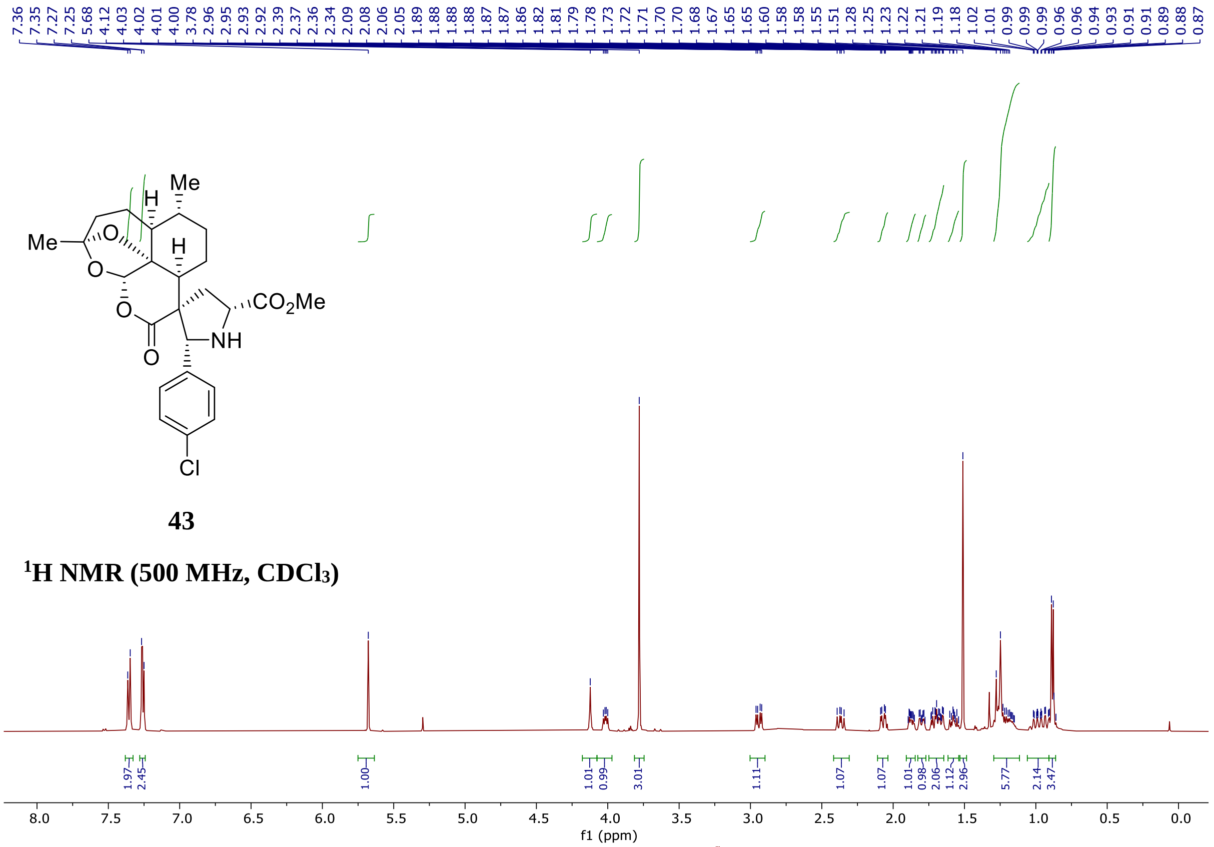

43

^{1}H NMR (500 MHz, $CDCl_3$)

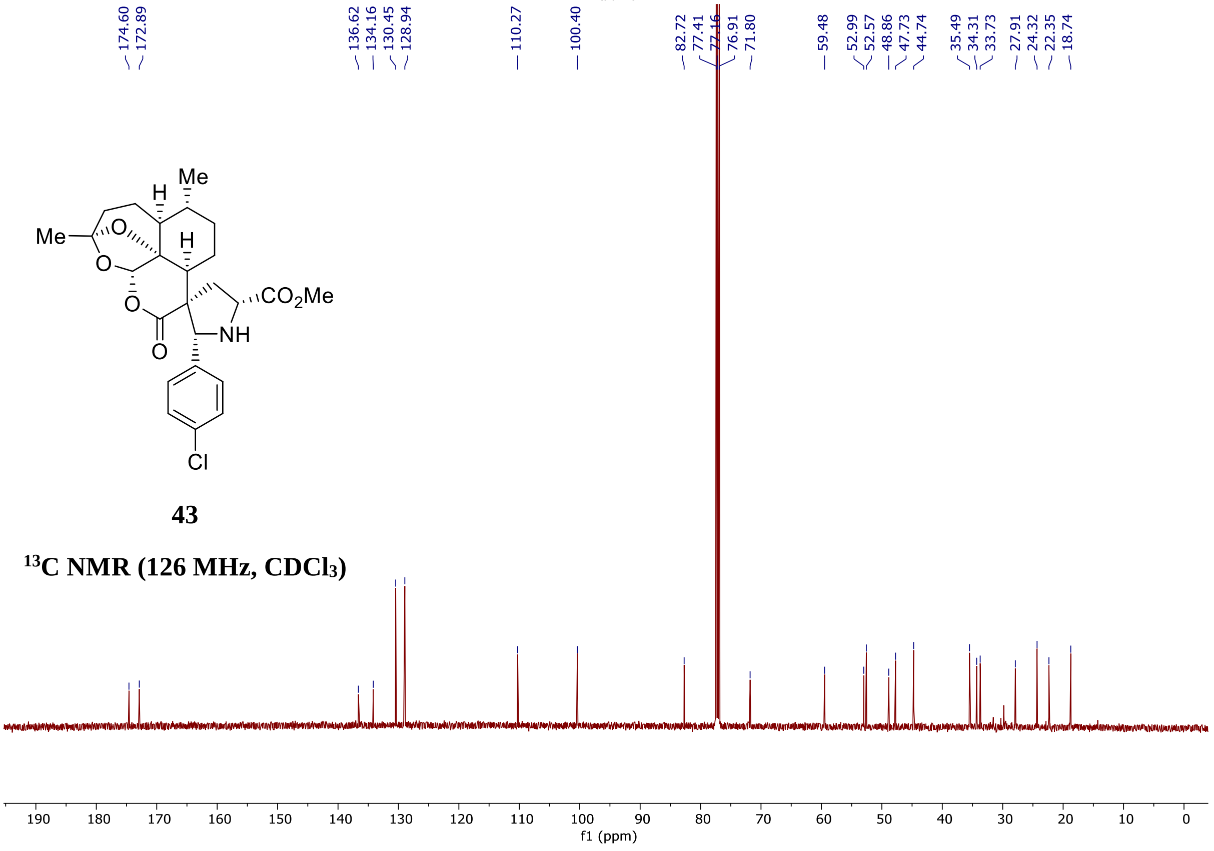

43

^{13}C NMR (126 MHz, $CDCl_3$)

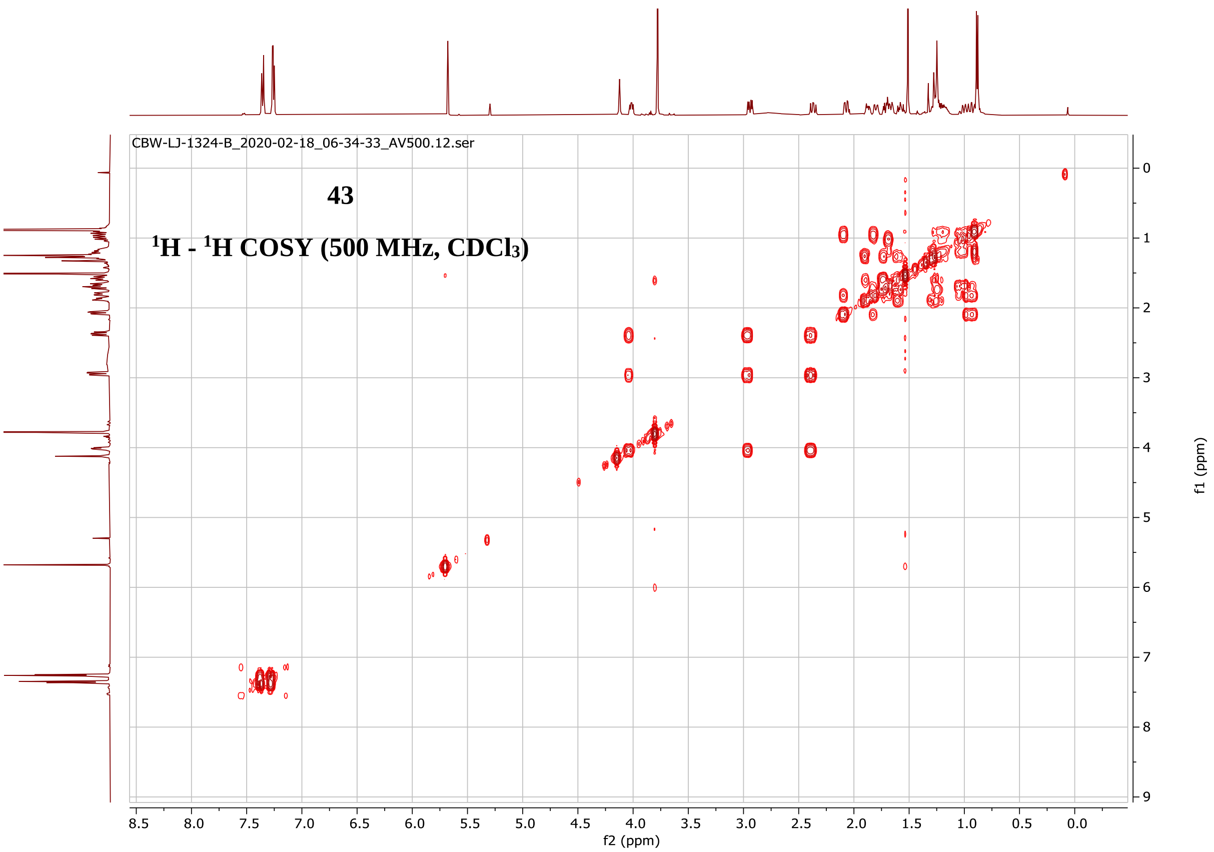
CBW-LJ-1324-B_2020-02-18_06-34-33_AV500.12.ser
43
^{1}H - ^{1}H COSY (500 MHz, CDCl3)
f2 (ppm)
f1 (ppm)

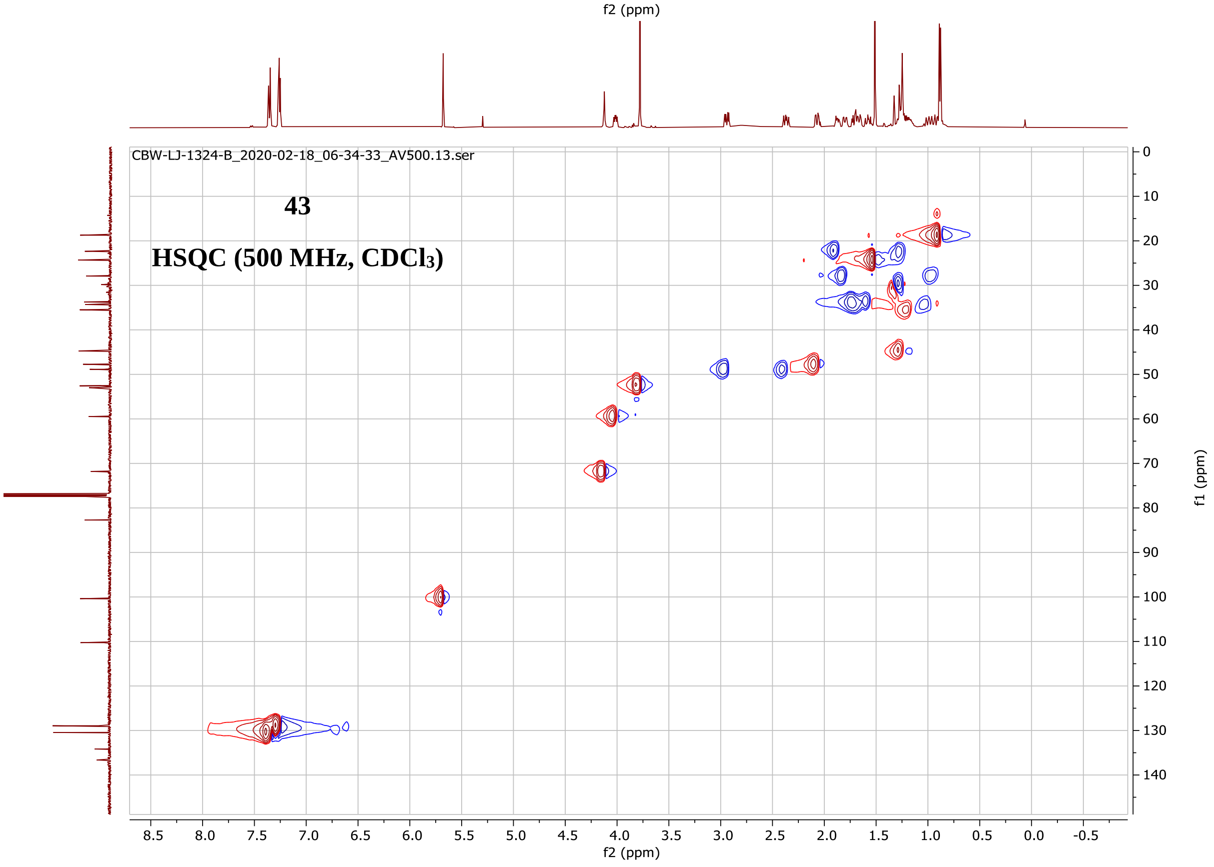
CBW-LJ-1324-B_2020-02-18_06-34-33_AV500.13.ser
43
HSQC (500 MHz, CDCl3)
f2 (ppm)
f1 (ppm)

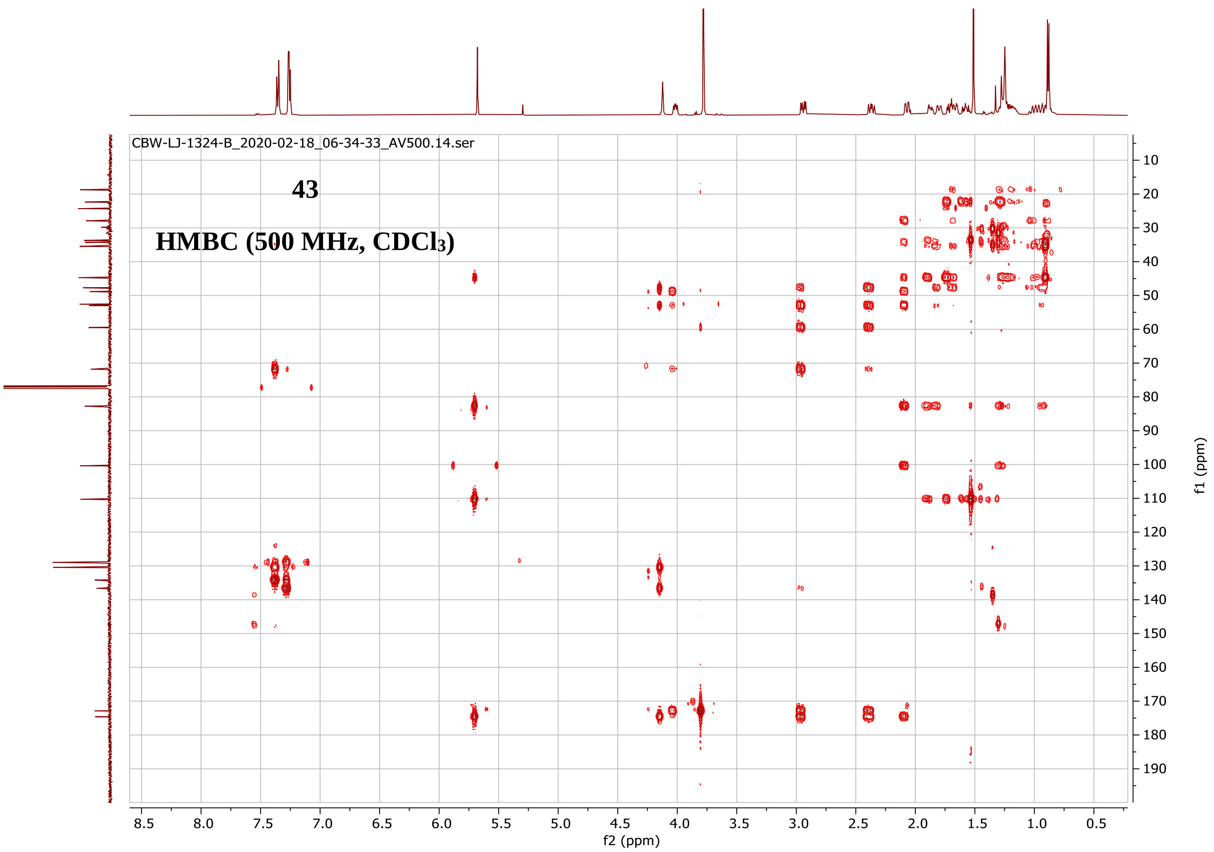
CBW-LJ-1324-B_2020-02-18_06-34-33_AV500.14.ser
43
HMBC (500 MHz, CDCl3)
f2 (ppm)
f1 (ppm)

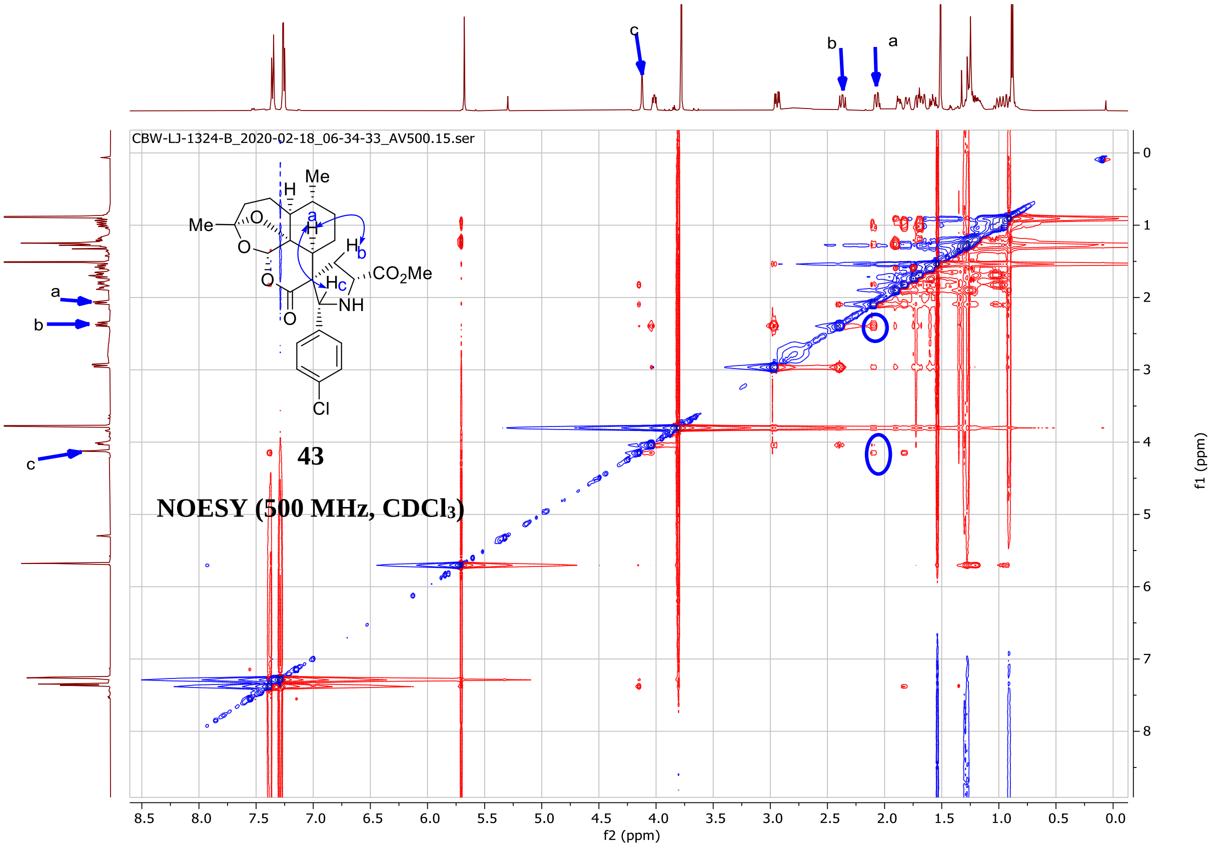
CBW-LJ-1324-B_2020-02-18_06-34-33_AV500.15.ser
a
b
c
Me
Me
H
H
Hb
Hc
O
NH
CO2Me
Cl
43
NOESY (500 MHz, CDCl3)
f2 (ppm)
f1 (ppm)

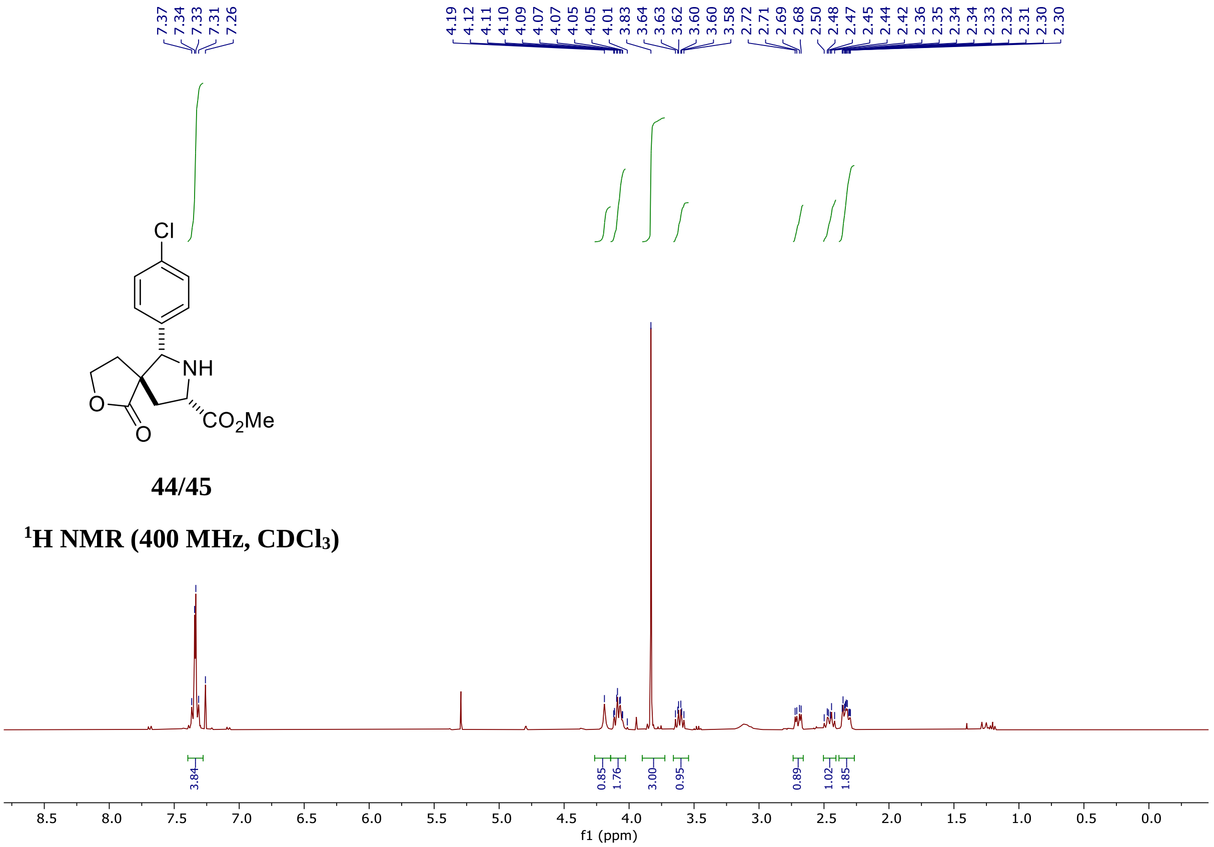

44/45

^{1}H NMR (400 MHz, CDCl$_3$)

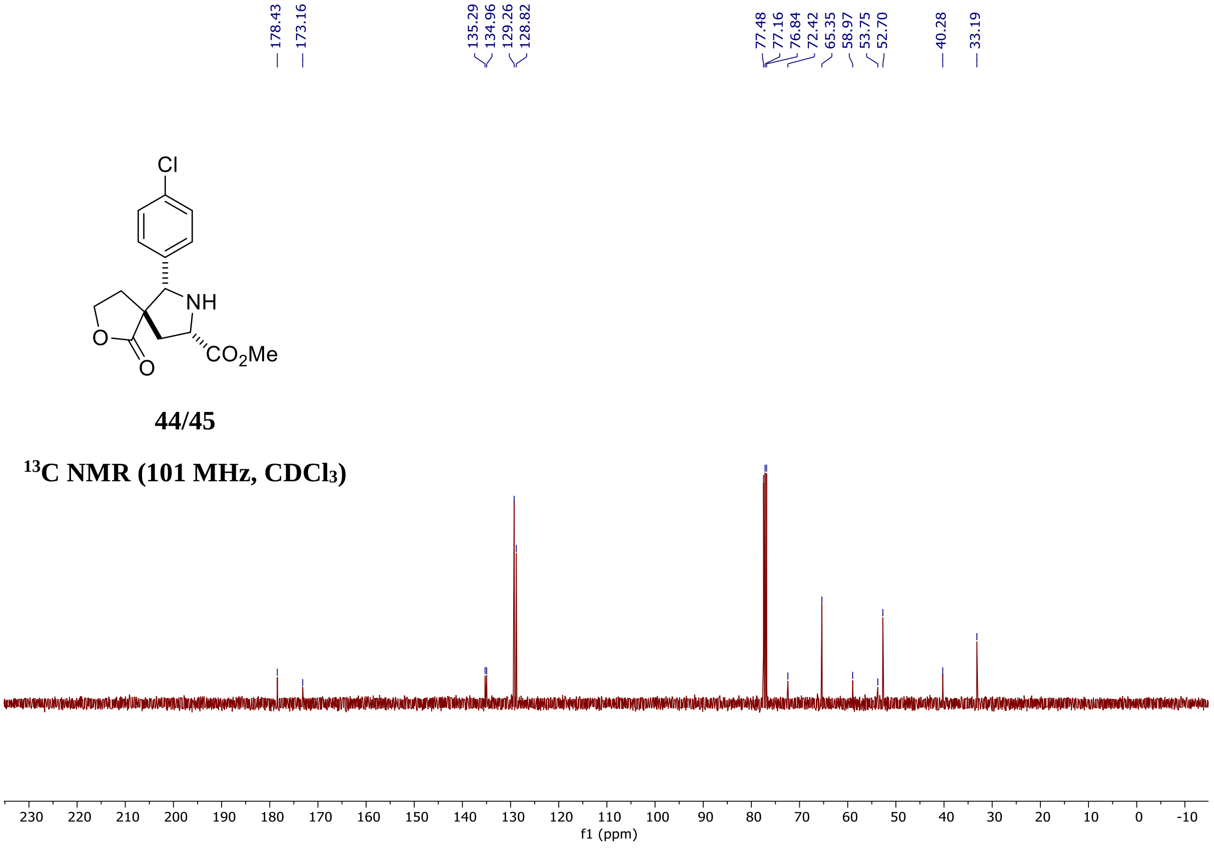

44/45

^{13}C NMR (101 MHz, CDCl$_3$)

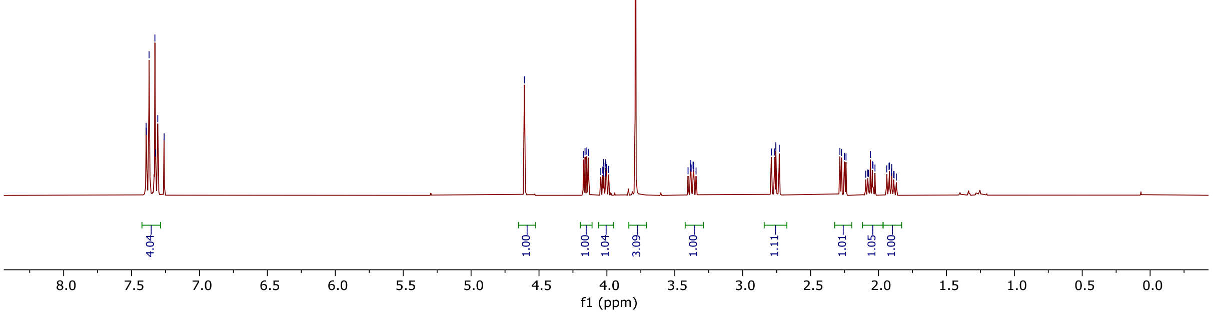

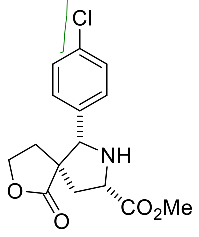

46/47

^{1}H NMR (400 MHz, CDCl$_3$)

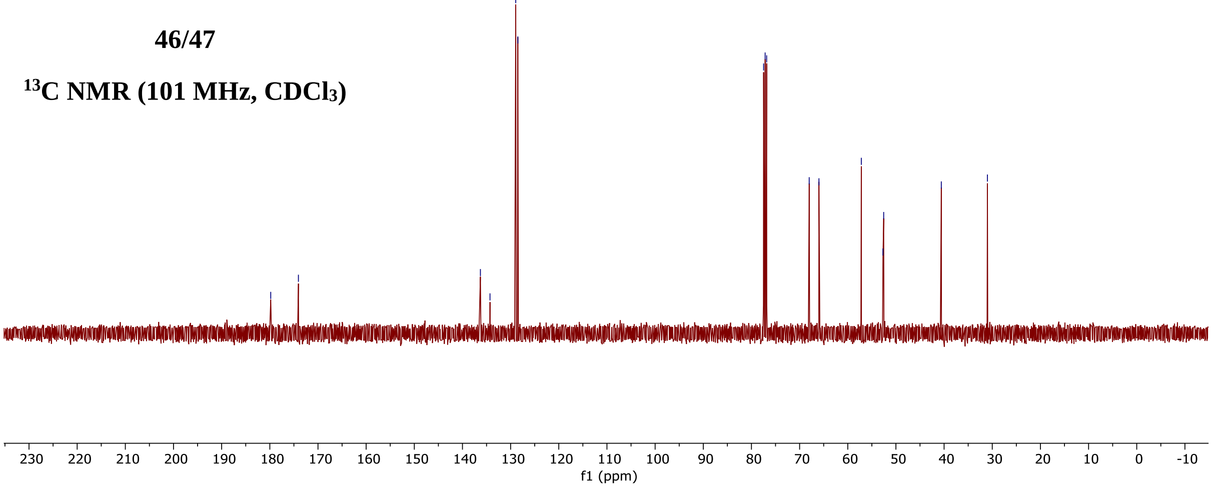

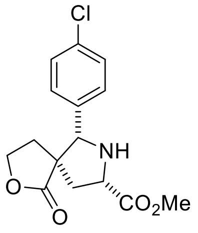

46/47

^{13}C NMR (101 MHz, CDCl$_3$)

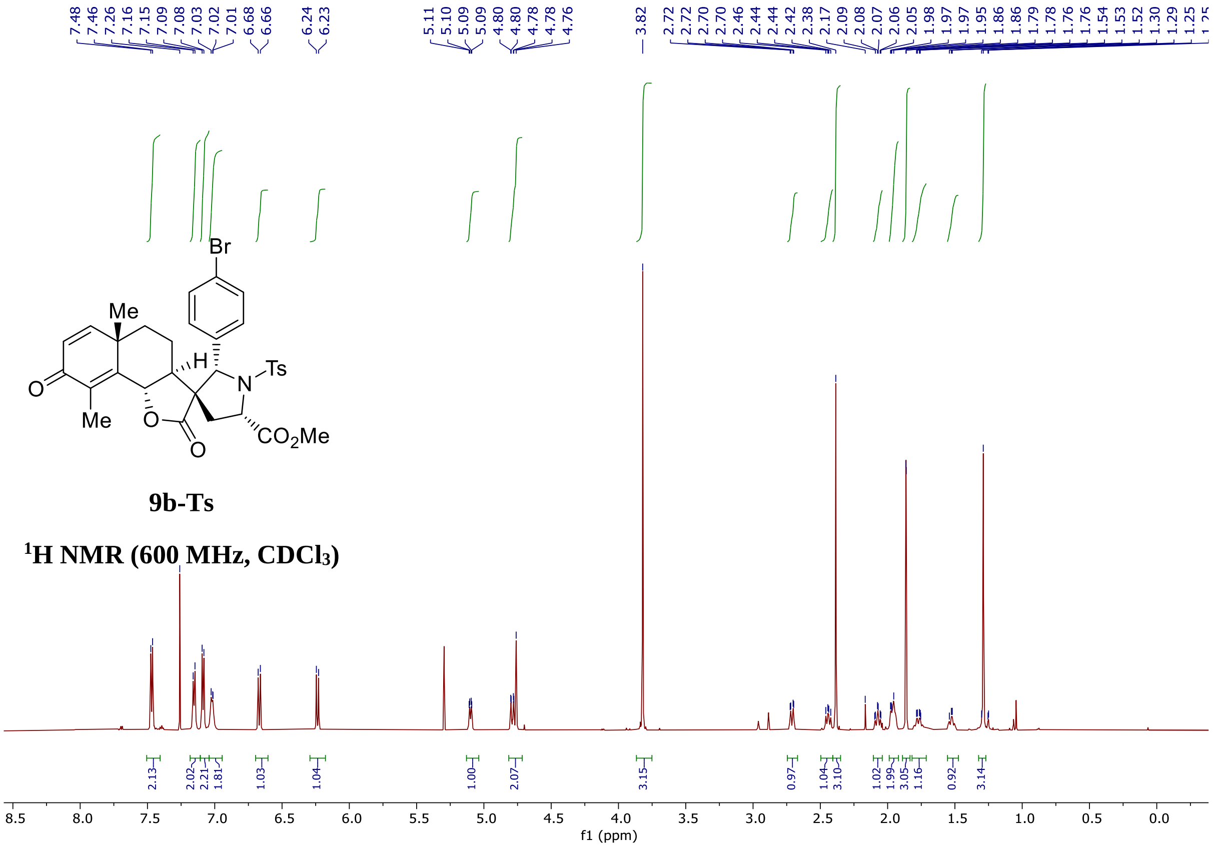
7.48
7.46
7.26
7.16
7.15
7.09
7.08
7.03
7.02
7.01
6.68
6.66
6.24
6.23
5.11
5.10
5.09
5.09
4.80
4.80
4.78
4.78
4.76
3.82
2.72
2.72
2.70
2.70
2.46
2.44
2.44
2.42
2.38
2.17
2.09
2.08
2.07
2.06
2.05
1.98
1.97
1.97
1.95
1.86
1.79
1.78
1.76
1.76
1.54
1.53
1.52
1.30
1.29
1.25
Br
Me
H
N
Ts
O
Me
O
O
CO2Me
9b-Ts
1H NMR (600 MHz, CDCl3)
2.13
2.02
2.21
1.81
1.03
1.04
1.00
2.07
3.15
0.97
1.04
3.10
1.02
1.99
3.05
1.16
0.92
3.14
8.5
8.0
7.5
7.0
6.5
6.0
5.5
5.0
4.5
4.0
3.5
3.0
2.5
2.0
1.5
1.0
0.5
0.0
f1 (ppm)

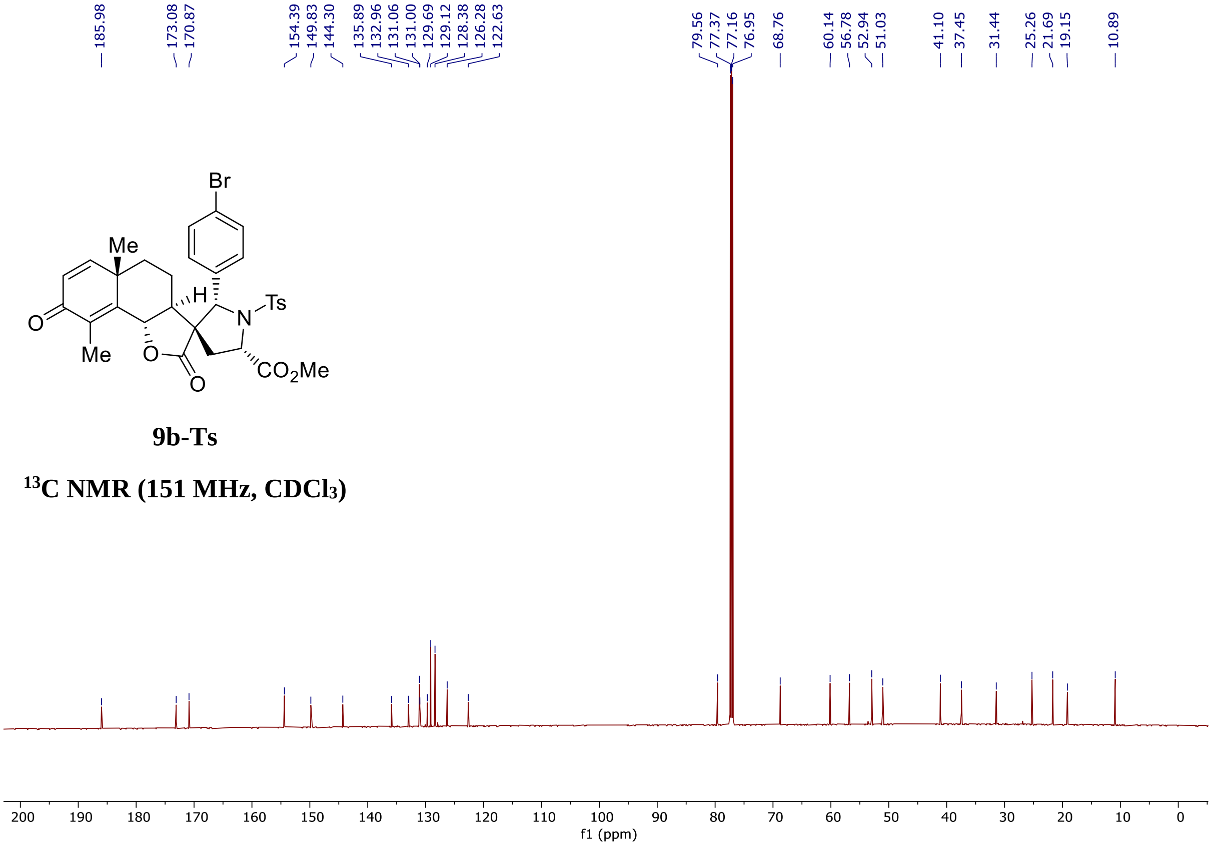
185.98
173.08
170.87
154.39
149.83
144.30
135.89
132.96
131.06
131.00
129.69
129.12
128.38
126.28
122.63
79.56
77.37
77.16
76.95
68.76
60.14
56.78
52.94
51.03
41.10
37.45
31.44
25.26
21.69
19.15
10.89
Br
Me
H
N
Ts
O
Me
O
O
CO2Me
9b-Ts
13C NMR (151 MHz, CDCl3)
200
190
180
170
160
150
140
130
120
110
100
90
80
70
60
50
40
30
20
10
0
f1 (ppm)

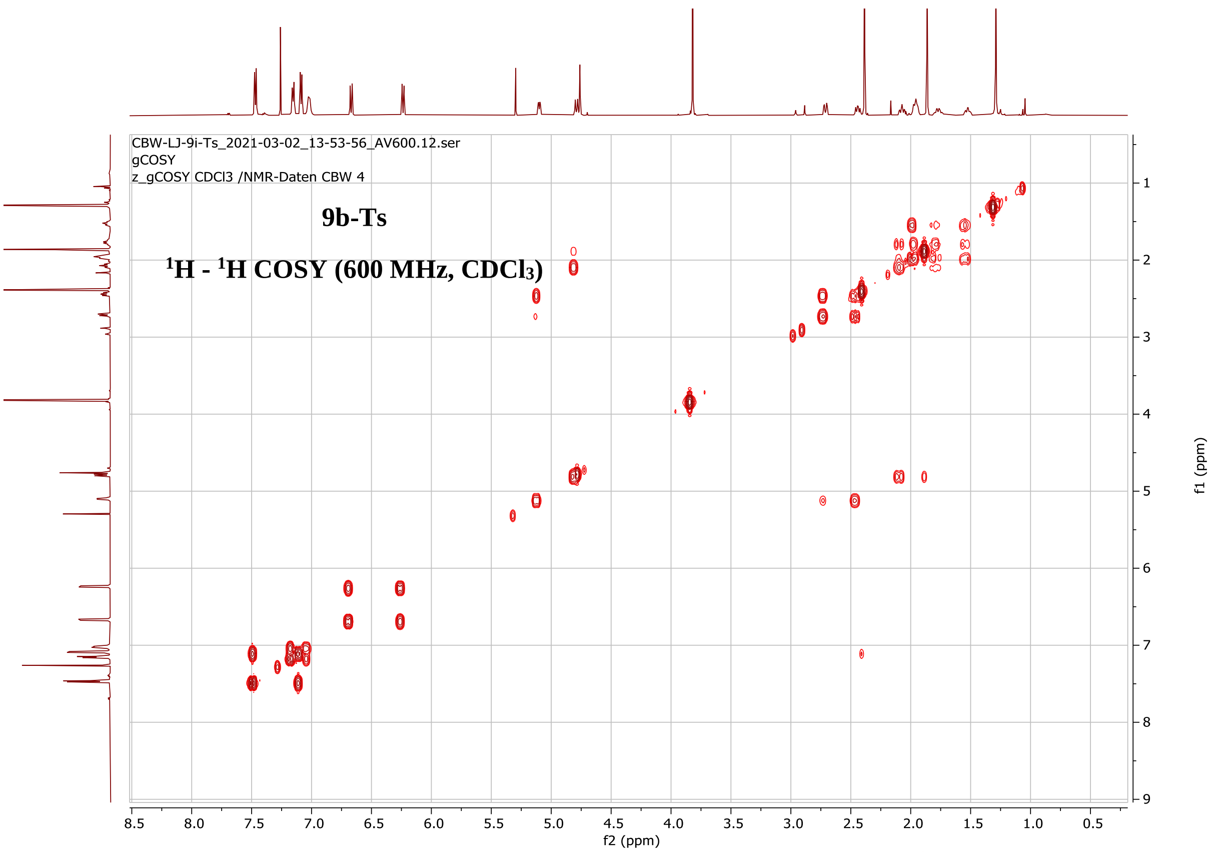
CBW-LJ-9i-Ts_2021-03-02_13-53-56_AV600.12.ser
gCOSY
z_gCOSY CDCl3 /NMR-Daten CBW 4
9b-Ts
1H - 1H COSY (600 MHz, CDCl3)
f2 (ppm)
f1 (ppm)

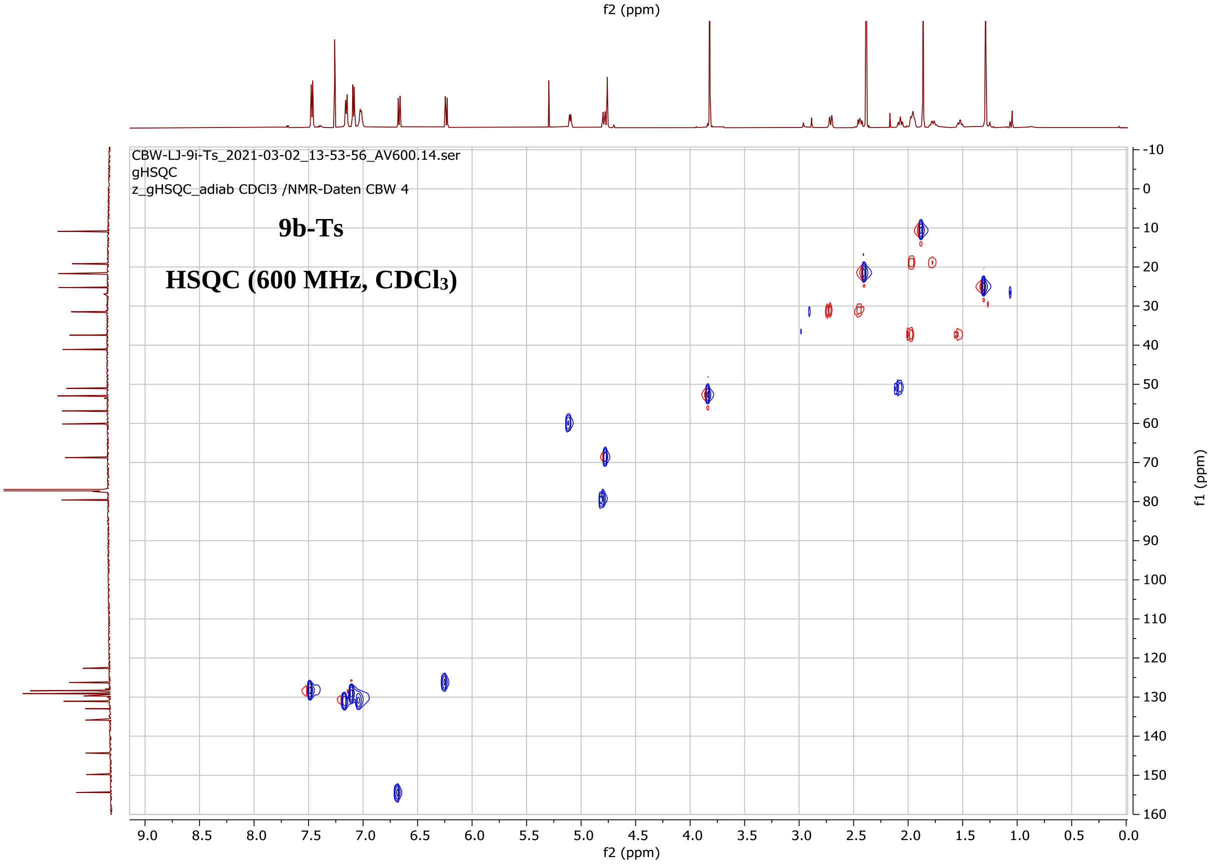
CBW-LJ-9i-Ts_2021-03-02_13-53-56_AV600.14.ser
gHSQC
z_gHSQC_adiab CDCl3 /NMR-Daten CBW 4
9b-Ts
HSQC (600 MHz, CDCl3)
f2 (ppm)
f1 (ppm)

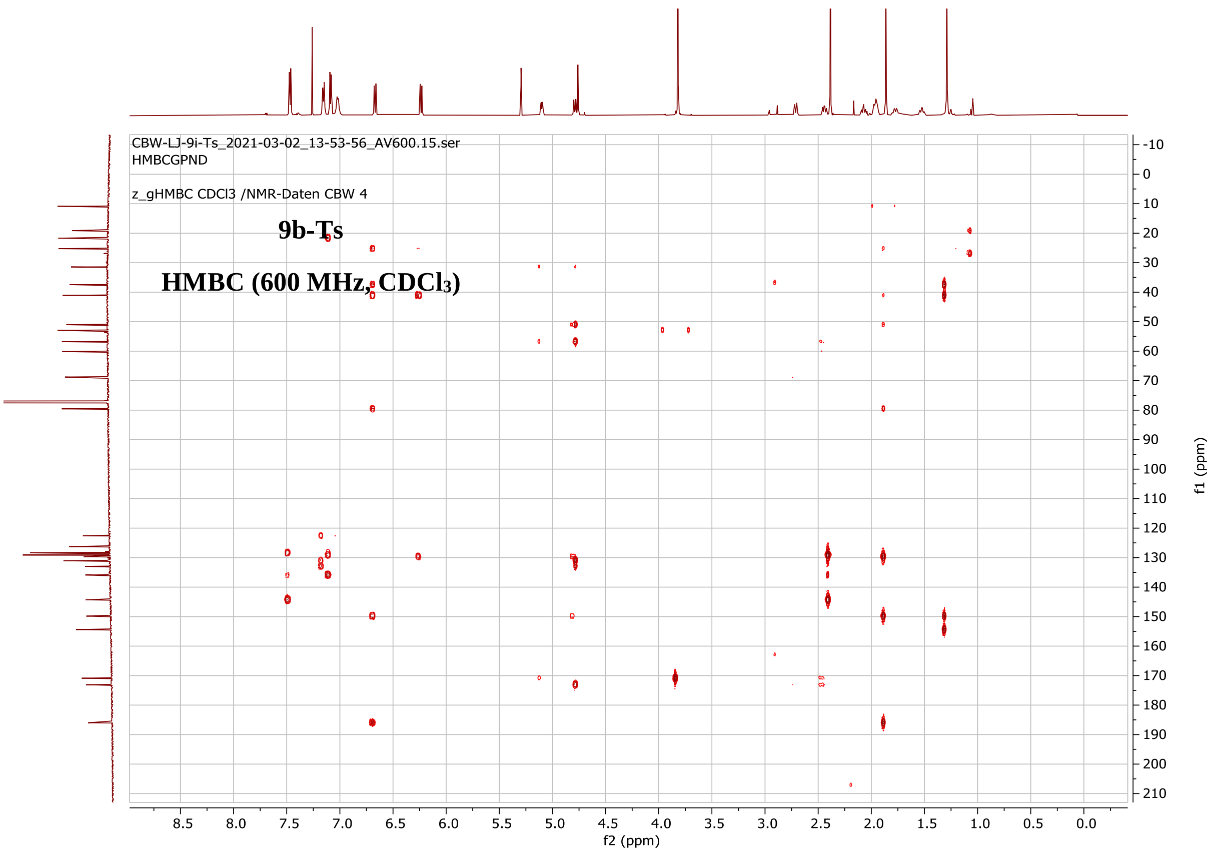
CBW-LJ-9i-Ts_2021-03-02_13-53-56_AV600.15.ser
HMBCGPND
z_gHMBC CDCl3 /NMR-Daten CBW 4
9b-Ts
HMBC (600 MHz, CDCl3)
f2 (ppm)
f1 (ppm)

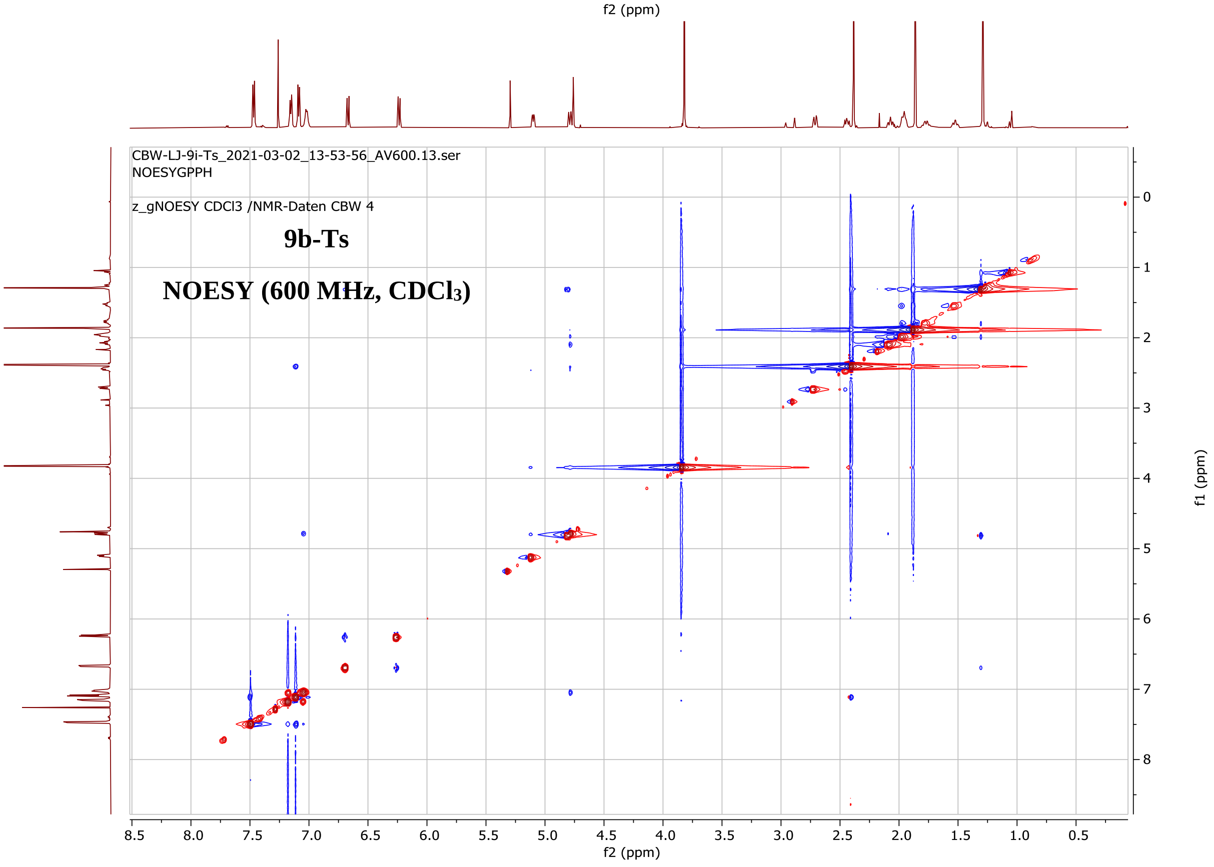
CBW-LJ-9i-Ts_2021-03-02_13-53-56_AV600.13.ser
NOESYGPPH
z_gNOESY CDCl3 /NMR-Daten CBW 4
9b-Ts
NOESY (600 MHz, CDCl3)
f2 (ppm)
f1 (ppm)

8. Biology experiment

Reagents

Purmorphamine was purchased from Cayman Chemical (#10009634) and DMSO from (Sigma Aldrich, #67685). The compound collection was synthesized as described in the paper. All other reagents used are mentioned in the respective protocols below, with the corresponding sources.

Cell lines

The murine mesenchymal stem cell line C3H10T1/2 (ATCC, CCL-226) was cultured in Dulbecco's Modified Eagle's medium (DMEM, high glucose, PAN, #P04-03550) supplemented with 10% of fetal calf serum (FCS, Fisher Scientific, #10136253, heat inactivated), 1 mM sodium pyruvate (PAN, #P04-43100) and 2 mM L-glutamine (PAN, #P0480100). The cells were cultured at 37°C and 5% CO_2 in a humidified atmosphere. Checks for mycoplasma contaminations were performed regularly and cells were always found to be free of any contaminations.

Osteoblast differentiation and viability assay

The osteoblast differentiation assay and the viability assay were performed using C3H10T1/2 cells. For the screening and IC_{50} determinations 800 C3H10T1/2 cells per well were seeded in white 384-well plates. After incubation overnight, cells were treated with 1.5 µM purmorphamine and different concentrations of the compounds or DMSO as a control. After 96 h the cell culture medium was aspirated and the commercial luminogenic ALP substrate CDP-Star (Roche, #11685627001) was added. The cells were incubated for one hour at room temperature and in absence of light. Afterwards the luminescence signal was read. To identify and exclude toxic compounds, which would also lead to a reduced luminescent signal, cell viability measurements were conducted in parallel. For this purpose, C3H10T1/2 cells were seeded and treated as described above. The cellular ATP content was determined as a measure of cell viability using the Cell Titer Glo reagent (Promega). Compounds were considered as hit compounds if they caused at least 50% reduction in the luminescent signal in the osteoblast differentiation assay while retaining cell viability $\geqslant$ 80% at a concentration of 10 µM. To determine IC_{50} curves for hit compounds, three-fold dilution curves starting from 10 µM, were used. Calculations of the IC_{50} values were conducted, using GraphPad Prism 7. (GraphPad Software, USA).
